# Transcriptional signature associated with early rheumatoid arthritis and healthy individuals at high risk to develop the disease

**DOI:** 10.1371/journal.pone.0194205

**Published:** 2018-03-27

**Authors:** N. Macías-Segura, J. E. Castañeda-Delgado, Y. Bastian, D. Santiago-Algarra, J. D. Castillo-Ortiz, A. L. Alemán-Navarro, E. Jaime-Sánchez, M. Gomez-Moreno, C. A. Saucedo-Toral, Edgar E. Lara-Ramírez, M. Zapata-Zuñiga, L. Enciso-Moreno, R. González-Amaro, C. Ramos-Remus, J. A. Enciso-Moreno

**Affiliations:** 1 Unidad de Investigación Biomédica de Zacatecas, IMSS, Zacatecas, México; 2 Departamento de Inmunología, Facultad de Medicina, Universidad Autónoma de San Luis Potosí, San Luis Potosí, México; 3 Catedras-CONACyT, Unidad de Investigación Biomédica de Zacatecas-IMSS, Zacatecas, México; 4 Instituto Potosino de Investigaciones Científicas y Tecnológicas, San Luis Potosí, México; 5 Unidad de Investigación en Enfermedades Crónico-Degenerativas, Guadalajara, México; 6 Facultad de Medicina Humana y Ciencias de la Salud, Universidad Autonoma de Zacatecas, Hospital Rural #51, IMSS, Villanueva, Zacatecas, México; 7 Universidad Autónoma de Guadalajara, Guadalajara, México; Mayo Clinic Rochester, UNITED STATES

## Abstract

**Background:**

Little is known regarding the mechanisms underlying the loss of tolerance in the early and preclinical stages of autoimmune diseases. The aim of this work was to identify the transcriptional profile and signaling pathways associated to non-treated early rheumatoid arthritis (RA) and subjects at high risk. Several biomarker candidates for early RA are proposed.

**Methods:**

Whole blood total RNA was obtained from non-treated early RA patients with <1 year of evolution as well as from healthy first-degree relatives of patients with RA (FDR) classified as ACCP+ and ACCP- according to their antibodies serum levels against cyclic citrullinated peptides. Complementary RNA (cRNA) was synthetized and hybridized to high-density microarrays. Data was analyzed in Genespring Software and functional categories were assigned to a specific transcriptome identified in subjects with RA and FDR ACCP positive. Specific signaling pathways for genes associated to RA were identified. Gene expression was evaluated by qPCR. Receiver operating characteristic (ROC) analysis was used to evaluate these genes as biomarkers.

**Results:**

A characteristic transcriptome of 551 induced genes and 4,402 repressed genes were identified in early RA patients. Bioinformatics analysis of the data identified a specific transcriptome in RA patients. Moreover, some overlapped transcriptional profiles between patients with RA and ACCP+ were identified, suggesting an up-regulated distinctive transcriptome from the preclinical stages up to progression to an early RA state. A total of 203 pathways have up-regulated genes that are shared between RA and ACCP+. Some of these genes show potential to be used as progression biomarkers for early RA with area under the curve of ROC > 0.92. These genes come from several functional categories associated to inflammation, Wnt signaling and type I interferon pathways.

**Conclusion:**

The presence of a specific transcriptome in whole blood of RA patients suggests the activation of a specific inflammatory transcriptional signature in early RA development. The set of overexpressed genes in early RA patients that are shared with ACCP+ subjects but not with ACCP- subjects, can represent a transcriptional signature involved with the transition of a preclinical to a clinical RA stage. Some of these particular up-regulated and down-regulated genes are related to inflammatory processes and could be considered as biomarker candidates for disease progression in subjects at risk to develop RA.

## Introduction

Rheumatoid arthritis (RA) has a multidimensional effect including progressive destruction of diarthrodial joints, development of comorbid conditions, family distress and high social costs [1–3]. Although the incidence of RA peaks during the fifth decade of life, in some countries close to 50% of patients develop symptoms before the age of 35 [4]. Early treatment can limit the overall impact of the disease [5, 6], including the prevention of joint damage and work absence [7–9]. Therefore, detection of RA at very early stages, or even during the pre-clinical phase of the disease, would have a meaningful impact on the patient outcome.

The genetic heritability of RA has been estimated at 12% to 60% [10]. Variation on these figures may be explained by differences in study design as well as in ethnicity and in characteristics such as duration of disease and treatments. A number of polymorphisms and variants in HLA molecules have been associated with the development of this disease, particularly the so-called “Shared epitope” [2, 4, 8]. Identification of a particular transcriptional signature in pre-clinical high-risk subjects would add insight on the molecular mechanisms involved in the development of RA, and may contribute to our knowledge of how self-tolerance is lost in autoimmune diseases. In addition, determination of transcriptional signatures may lead to the selection of biomarkers for the early detection of RA. Some reports in humans and murine models have identified transcriptional profiles associated with the development of some diseases (10, 11,12). For instance, in patients with arthralgia that latter progressed to RA, Verweij and colleagues [11] identified a transcriptional profile mainly composed of genes associated with Interferon (IFN)-mediated immunity, cytokine/chemokine activity and hematopoiesis. Moreover, another study showed that five interleukins were increased in the serum of subjects that later developed RA [9]. Nevertheless, the molecular events that trigger the development of RA in healthy subjects at high-risk are still not clear.

Our group recently reported that 58% of healthy relatives of RA patients develop the disease during the following 5-year period after the serum levels of anti-citrullinated protein antibodies (anti-CCP) >25 UI/ml were detected. Thus, the detection of anti-CCP antibodies allows the identification of healthy subjects at high risk of developing RA [12] In this context, the aim of this study was to assess the differences in transcriptional profiles between healthy subjects at high-risk of RA and patients with early-RA by means of microarray analysis followed by qPCR validation. Analysis of the differentially expressed pathways is explored and discussed in the context of RA physiopathology and the potential of several genes as biomarkers is also analyzed.

## Material and methods

### Subjects

This is a cross-sectional study to assess the differences in transcriptional profile between three groups of subjects described as follows: The group 1 (early-RA; RA) was comprised of RA patients classified according to the criteria of the EULAR/ACR 2010 (26), all of them with less than 1 year of disease evolution (defined from the date of the first swollen joint) and whom were off disease-modifying anti-rheumatic drugs (DMARDs) or glucocorticoids, but were attending a secondary-care rheumatologic clinic as outpatients. Group 2 (high-risk RA; ACCP+) (1) was comprised of healthy first-degree relatives of patients with established RA. All members in this group were older than 18 years, positive to anti-CCP, with no arthritis as per history or any other rheumatic disease. Group 3 (low risk controls; ACCP-) comprised healthy first-degree relatives of patients with established RA. All of them were older than 18 years and anti-CCP negative with no arthritis or any other rheumatic disease as it was described before [13]. All subjects were clinically examined in an independent way by two different certified rheumatologists. In all subjects, anti-CCP antibody levels were assessed using a second-generation IgG anti-CCP2 (ELISA, CCPlus Immunoscan kit, Euro Diagnostica AB, Switzerland) whereas IgM rheumatoid factor (RF) was assessed using nephelometry. According to manufacturer’s instructions, anti-CCP2 levels were considered positive if >25 IU/ml.

The Ethics Committee of the “Instituto Mexicano del Seguro Social” approved the protocol (IMSS, registry number R-20013-785-009). All of the participants enrolled in the study signed a written informed consent letter prior to any procedure.

### Collection of the biological material

For microarray analysis (discovery set) the following number of subjects was included: 6 ACCP+, 7 early RA and 7ACCP- group. For the validation set a total of 34 subjects were included (which comprised also the discovery set) as follows: twelve ACCP+, twelve from the reference group (ACCP-), and ten from the early-RA group (RA), all of which were similar in age and gender. Clinical and serologic variables of the studied subjects are shown in Table 1. Individual serum samples were obtained and stored at −20°C until analysis. Whole blood samples were collected using Vacutainer tubes with EDTA (Becton-Dickinson, USA). Samples were then mixed with 1 ml of RNAlater (Invitrogene, USA), homogenized and frozen at −70°C until use. Additional samples for Receiver Operating Characteristic (ROC analysis) were included for validation purposes; a subset of genes was evaluated in these samples as potential biomarkers for RA progression.

**Table 1:** Clinical and serologic variables of the studied subjects.

	Healthy Relatives		RA patients	*P* value
	Anti-CCP2+ N = 12	Anti-CCP2- N = 12	N = 10	
Age, yrs., mean ± SD	35 ± 8.7	43 ± 14.8	41 ± 9.8	0.257
Female (%)	75	91	80	0.534
Anti-CCP2 (U/ml), mean ± SD)	35 ± 5.4*	13 ± 1.7	194 ± 238.9*	0.0001
FR (IU/ml, mean ± SD)	6 ±3.5	7 ± 9.1	181 ± 178.2*	<0.0001
DAS28	NA	NA	5.8 ± 1.2	-

*Indicates significant differences for the anti-CCP2- group.

### RNA extraction and cDNA synthesis

Total RNA was extracted using a standardized protocol in our laboratory. Briefly, blood samples were thawed to room temperature and mixed with a volume of TRIzol^®^ (Invitrogen, USA). Samples were then mixed with one-tenth of chloroform and centrifuged at 13,000 rpm during 15 min at 4°C to obtain an aqueous phase with the nucleic acids material. One volume of 70% ethanol was added to the aqueous phase and homogenized. Samples were then processed in QIAmp RNA blood mini-columns (Qiagen, USA) according to the supplier’s instructions [14]. RNA concentration and integrity was evaluated in BioAnalyzer 2100 with the RNA 6000 Nano kit (Agilent Technologies, USA). Only samples with an RNA integrity number (RIN) >8 were considered for the synthesis of complementary Cy3-labeled copy RNA (cRNA). For RT-qPCR determinations 2.5 μg of total RNA from each sample were converted to cDNA using the Superscript II enzyme (Invitrogene, USA), following the supplier’s instructions [15].

### Hybridization and analysis of the Agilent GE 4X44 Expression Microarrays

Transcriptional analysis profiles from Cy3-cRNA samples were assessed using seven samples from the early RA patients group, six samples from ACCP+ group, and seven samples from ACCP- group. Microarray hybridization was performed using the Agilent 4X44K platform containing a total of 27,958 genes (Agilent Technologies, USA). Two hundred ng of total RNA were processed with one-color marker protocol, and sample microarray hybridization was carried out following the supplier’s instructions (Agilent Technologies, USA). Mean fluorescence intensities (MFI) from microarrays readings were obtained by use of the *Agilent G4900DA SureScan Microarray Scanner* (Agilent Technologies, USA). The *Agilent Feature Extraction* program (Agilent Technologies, USA) was used for individual samples data extraction. A non-supervised analysis of the data was generated with the *Agilent GeneSpring* software (12.6 ver, Agilent Technologies, USA). Fold change of induced and/or repressed genes between groups was determined by the GeneSpring program through matrix analysis (cluster analysis) and analysis of variance (ANOVA) using a linear threshold of 2 and a *P* value of less than 0.05 with the Benjamini Hochberg false discovery rate correction. We used *Gene Ontology* (GO) and *Pathway* analysis of the *Agilent GeneSpring Software* platform (Agilent Technologies, USA) to identify the biological function and interaction cascades of identified genes with a significance value for such GO association of P<0.05. Data is available at Gene Expression Omnibus under GSE100191.

### Gene validation and amplification by real-time PCR

To corroborate microarray results by qPCR, we considered some of the up-regulated genes in the RA group (early RA), which were associated to innate immune response, humoral innate immune response and inflammatory response. Nine induced genes were selected and primers sequences for qPCR were designed using the Roche online platform [16], (S1 Table). Fifty ng of cDNA per gene in a total volume of 10 μl per-reactions were run by triplicate for each sample. Amplification of cDNA samples was carried out using the SsoFast enzyme EvaGreen (Bio-Rad, USA). We used 45 cycles consisting of 5 seconds of denaturalization at 95°C, 10 seconds of alignment at 60°C, and 10 seconds of extension at 72°C. The qPCR was conducted employing Roche LightCycler^®^ 480 (Roche Diagnostics, USA). Relative expression of each gene was evaluated by the previously described 2-(ΔΔCt) formula [17]. Normalization of qPCR results was done with the housekeeping gene HPRT.

### Statistical analysis

For statistical analysis in qRT-PCR experiments, GraphPad Prism 5.0 statistical software was used. Significant differences between groups were determined by one-way ANOVA with Tukey *post-hoc* test if normal distribution was determined, and Kruskal-Wallis test with Dunn *post hoc* test for non-parametric data. Categorical variables were compared utilizing the χ^2^ test. A ROC curve analysis was performed to evaluate the potential of a subset of genes as biomarkers, AUC (area under the curve) and p values are reported. In all cases the Two-tailed *p* values of <0.05 were considered significant.

## Results

### Gene expression profiles of ACCP- and relatives ACCP+ compared to early RA patients

**Fig 1:**
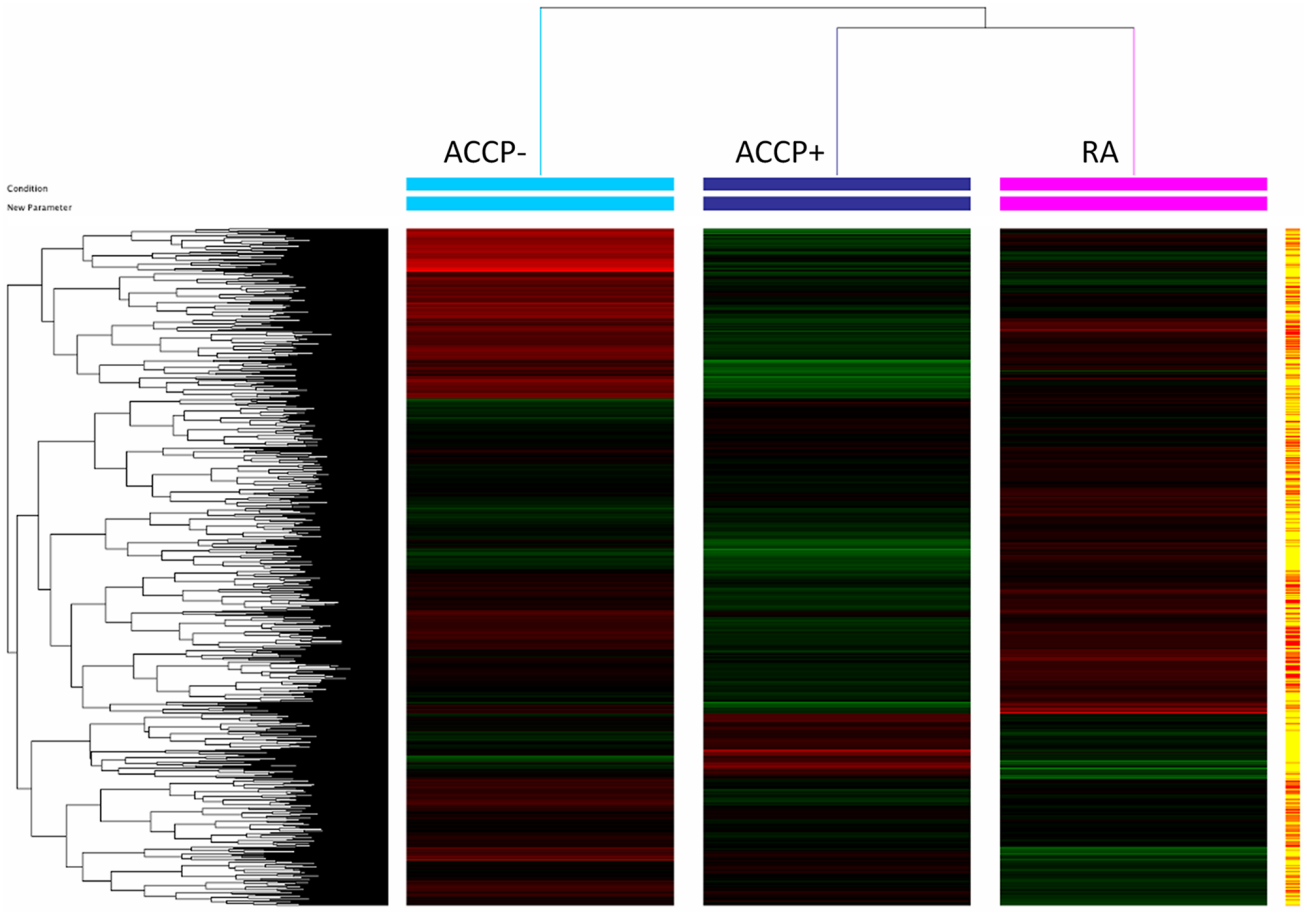
Cluster analysis of genes associated with early stages of rheumatoid arthritis (RA). Samples of total RNA from patients with RA (pink) and from healthy subjects with ACCP+ (dark blue) and ACCP- (light blue) were used to identify the transcriptional profile associated with each of the representative pre- and clinical stages in RA by High-density microarray (4X44k, Agilent, USA). The gene expression results were obtained by an unsupervised analysis using GeneSpring ver. 12.6 software (Agilent Technologies, USA).

**Fig 2:**
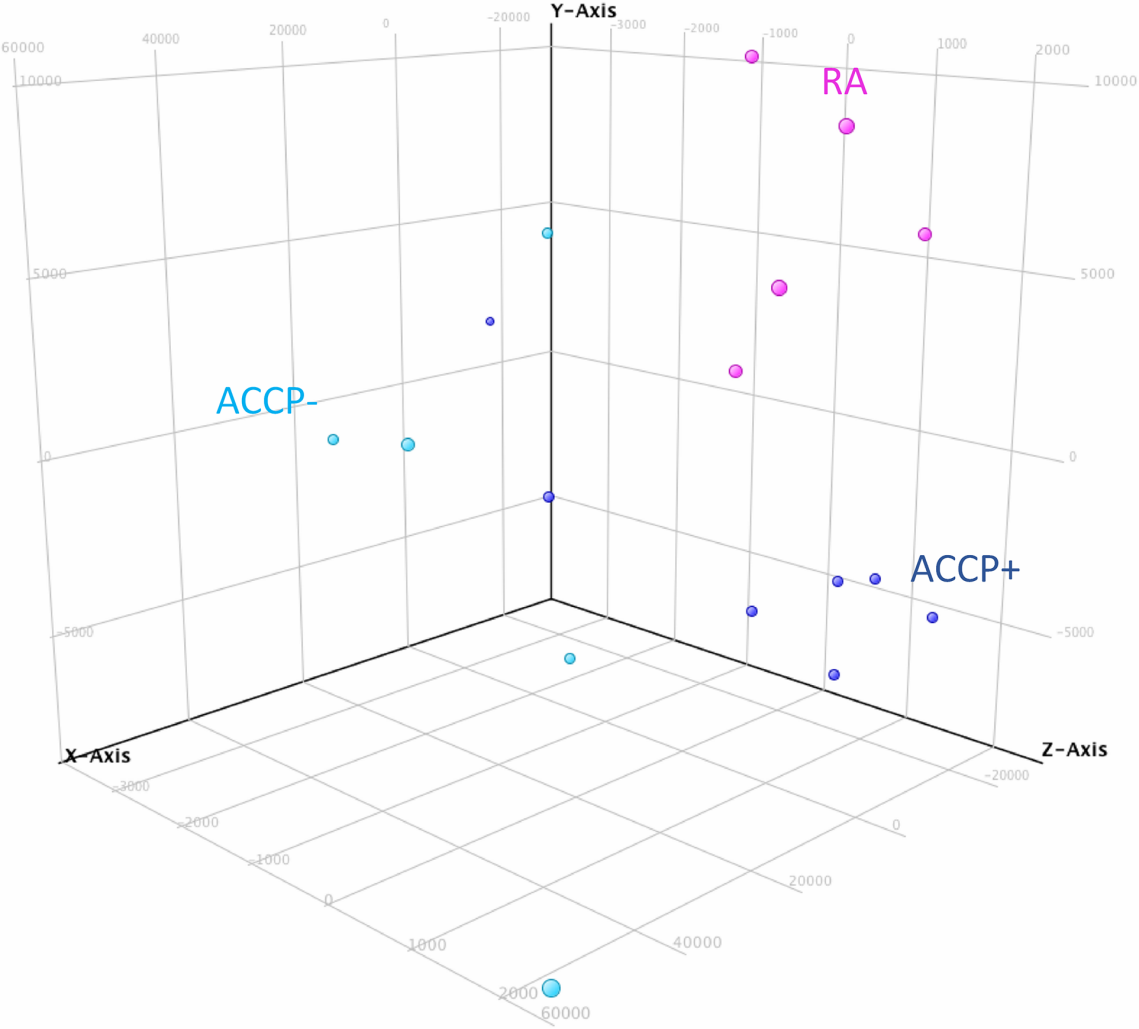
Principal component analysis for up and down-regulated genes from ACCP-, ACCP+ and rheumatoid arthritis (RA) groups. Microarray analysis data from RA (pink), ACCP- (light blue), and ACCP+ (black blue) were processed by a principal component analysis method by GeneSpring software.

In order to identify the molecular processes associated with RA development and the loss of tolerance, a discovery phase was undertaken in a carefully selected group of individuals by means of microarray data analysis. We identified which genes were differentially expressed between patients with early RA and high risk ACCP+ individual. We identified changes in the transcriptional profiles between the ACCP+ and RA groups *versus* the group of ACCP- relatives with an unsupervised analysis of the microarray data and a cluster analysis. From the set of 27,958 gene sequences present in the microarray, a specific transcriptome was identified for each group (Fig 1). Disease progression was associated to a list of 551 Up-regulated genes (URG) (S2 Table**)**. Interestingly, >4,000 down-regulated genes (DRG) (S3 Table) were exclusively observed in the early RA group versus the low risk ACCP- group. Considering that subjects with RA develop autoantibodies several years before the disease onset, the differences in transcriptional patterns are important for the understanding of the preclinical autoimmune phase of RA. For this purpose, comparisons between the high-risk ACCP+ group versus the low-risk ACCP- group identified a transcriptome of 876 URG and 7,531 DRG (S4 and S5 Tables). The capacity of such gene expression patterns to categorize individuals into such groups can be visualized in a principal Component Analysis (PCA), which also confirms the transcriptional differences among the three studied groups (Fig 2).

### Differentially expressed genes between ACCP+ and RA

**Fig 3:**
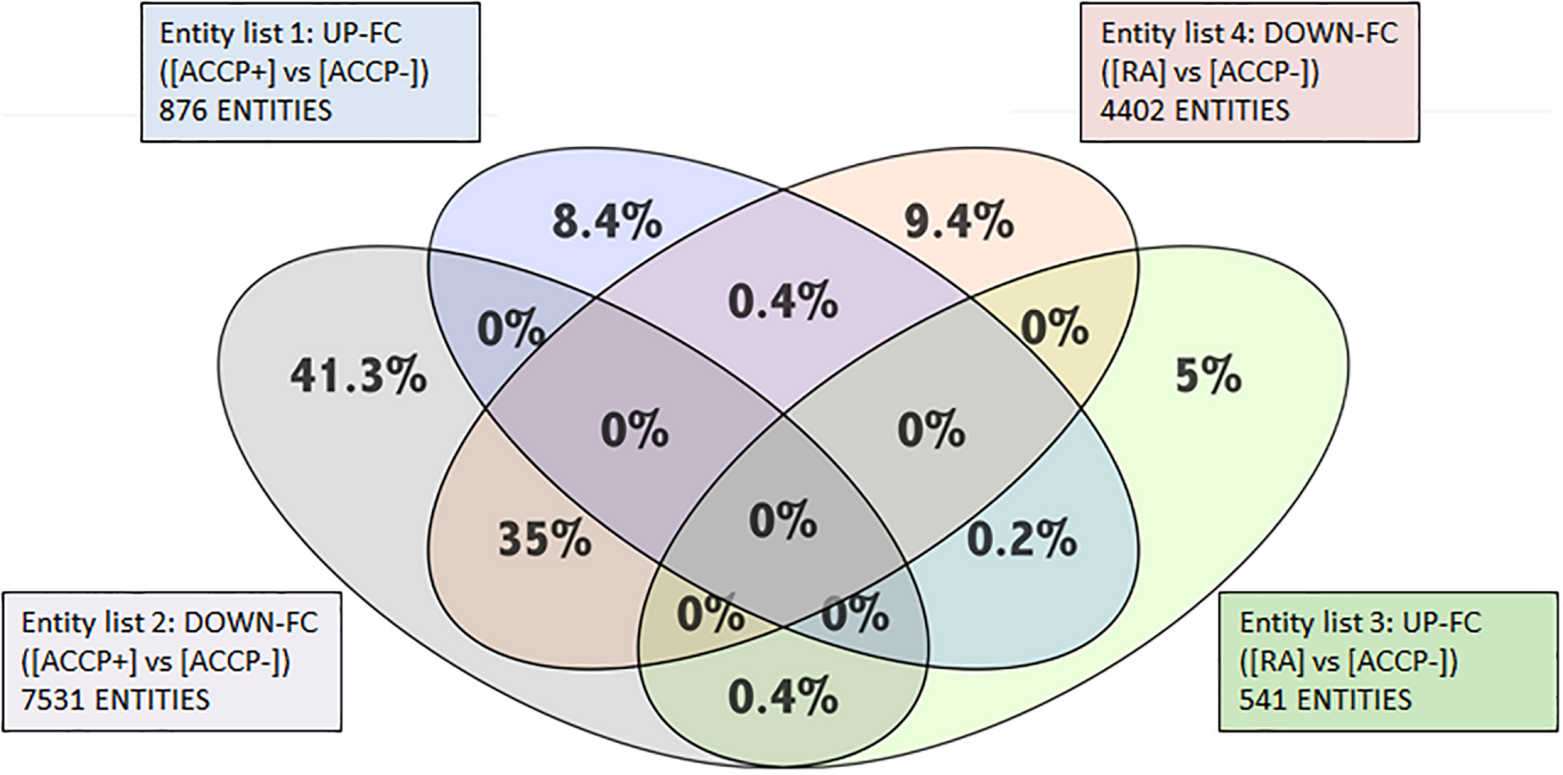
Venn diagram identifies shared and specific genes between ACCP+ and RA groups. Up regulated genes and down regulated genes in groups ACCP+ and RA were compared versus ACPP- group. Venn Diagram was performed using the GeneSpring Software ver.12.6 (Agilent Technologies, USA) to identify groups of genes up regulated exclusively in RA patients (blue, 8.4%) and up regulated exclusively in ACCP+ (green 5%); down regulated exclusively in RA patients (grey, 41.3) and in ACCP+ (orange, 9.4%). The diagram shows that there are a great number of down regulated genes shared between both groups (RA and ACCP+ vs ACCP-; merge in grey and orange, 35%) and a small number of genes shared between up-regulated genes (RA and ACCP+ vs ACCP-; merge in green and blue, 0.2%).

ACCP antibodies are highly specific for RA and are considered good predictors of the development of RA. To identify the variation in gene expression of the full dataset and to search genes associated with the increased risk to develop RA, a Venn diagram was constructed using the data from the preclinical stage group (ACCP+) and the early RA patients (Fig 3). Shared genes between RA and ACCP+ were identified: 0.2% (17 genes) was URG, mainly related to signal transduction, transcription factors and cytoskeleton, and 35% were DRG genes associated to innate immune response, cell growth, signal transduction and chemical response (full list in S6 and S7 Tables).

Furthermore, 0.4% was URG in the ACCP+ but DRG in RA (S8 Table); A similar 0.4% of the gene repertoire was URG in RA patients but DRG in ACCP+ (S9 Table). Noteworthy, out of the total number of differentially expressed genes, 35% are DRG shared between the high risk ACCP+ and early RA patients. However, 41% of the DRG were exclusive to the ACCP+ group, and only 9.4% of the DRG were exclusive to the early RA patients. Likewise, 8.4% are URG that belongs specifically to ACCP+ group whereas 5% of the URG are specific to the early RA patients.

### Identification of functional categories of the URG in ACCP+ and RA

In order to identify the functional categories of the differentially expressed genes and to have a better understanding of their participation in the physiopathology of RA, a gene ontology (GO) analysis was carried out. As shown in Table 2 a subgroup of up-regulated genes shared between the high risk ACCP+ and early RA patients that are associated to the establishment/development of the disease and the loss of tolerance were identified. These shared gene belong to the innate immune response, IFN type I signature and to leukocyte activation/migration, among others. A full list of genes in each associated biological process in the high-risk group and the early RA patients can be found in S10 and S11 Tables respectively. In particular, GO analysis of the genes overexpressed in RA patients shows that they are associated to inflammation, leukocyte activation, migration and differentiation as well as to hormones and cytokines. Furthermore, the distinctive transcriptome of RA patients is associated to cellular response to chemical stimuli, stress response and genes associated to regulation of metabolic-processes. Also, some of the URG fall under categories such as signal transduction and cell surface receptor signaling pathways (S11 Table).

**Table 2:** Biological processes in ACCP+ and rheumatoid arthritis (RA) groups of up-regulated genes by Gene ontology (GO) analysis.

Up-regulated GO in relatives with ACCP+	*N*, entities
• Innate immune response	33
• Interferon type 1 response	5
• Interferon gamma response	10
• Leukocyte activation	17
• Leukocyte migration	12
• Myeloid lineage differentiation	12
• Leukocyte differentiation	10
• Purine-containing compound catabolic process	24
• Cellular response to cytokine stimulus	25
• Cellular response to organonitrogen compounds	18
• Cellular response to hormones	20
• Chemotaxis	23
• Cellular response to insulin stimulus	10
• Cellular response to chemical stimulus	88
Up-regulated GO in patients with RA,	*N*, entities
• Inflammatory response	8
• Cell projection organization	21
• Signal transduction	89
• Cellular response to chemical stimulus	41
• Defense response	27
• Immune response	19
• Cellular response to stress	29
• Regulation of metabolic process	122
• Positive regulation of immune system process	17
• Cell surface receptor signaling pathway	49
• Cellular protein modification process	56
• Brain development	21
• Cellular nitrogen compound metabolic process	72

### Confirmation of microarray data by qPCR

Given the wide descriptive nature of microarray data, to provide stronger evidence that such massive data are consistent (according to the MIAME guidelines) we used qPCR to confirm microarrays results. For this purpose, additional samples were included as a validation set (including those used in the microarray hybridization experiments). A subset of 9 randomly selected up regulated genes from S11 Table were analyzed by RT-qPCR. Similar results to those obtained with microarray data analysis are shown in Table 3, showing a good overall concordance between the two methods and further confirming the validity of the microarray analysis so far.

**Table 3:** Relative expression by RT-qPCR of 9 inflammatory up-regulated genes.

	First-degree relatives ACCP-(n = 12)	First-degree relatives ACCP+(n = 12)	RA(n = 7)	P-Value
BCL2	0.45±0.36	0.48±0.43	0.87±0.30	0.0593
SERPINGB9	0.45±0.40*	0.50±0.20*	1.02±0.53	0.0075
SERPING1	0.84±0.91	0.96±1.25	0.25±0.32	0.3059
SNCA	0.37±0.28	0.72±0.74	1.32±0.88	0.1560
MS4A1	0.31±0.34*	0.40±0.24*	1.45±0.88	<0.0001
ETS1	0.53±0.47*	0.61±0.35*	1.3±0.38	<0.011
EGR1	0.51±0.50*	1.61±2.03*	5.01±4.9	0.0042
CX3CL1	5.66±7.49	1.79±2.04	0.07±0.09	0.0460
MEF2A	0.3292±0.31*	0.4733±0.31*	1.048±0.69	0.0041

Values represent mean ± SD; comparison between groups was performed using the Kruskal-Wallis test or ANOVA when necessary. Two-tail p value less than 0.05 was considered significant.

* indicates differences when compared with RA group.

### Pathway analysis shows a specific set of genes in RA and a shared set of Up-regulated genes in ACCP+ and RA

To associate biological functions of the up regulated genes to define the active pathways that are regulated during the progression process from ACCP + into RA, we elaborated an overall picture of the biological interactions of the transcriptomes in the context of the studied groups. In Table 4, we show that 8 signaling pathways are specific for early RA URG. Importantly these pathways include genes whose products are involved in the metabolism of phosphatidylinositol, cellular and migration processes as well as immune response against intracellular pathogens such as viruses. The signal pathways deduced from URG in high-risk individuals (ACCP+) and early RA patients were analyzed to identify the molecular interaction between induced genes in our groups. Nineteen induced pathways deduced from URG genes in ACCP+ high-risk individuals were identified (S12 Table). Our analysis shows the presence of induced pathways shared between the ACCP+ group and RA patients (S13 Table). These pathways are mainly related to the WNT signaling pathways, regulation of cytokines synthesis, clotting and the complement system, TCR signaling pathway and the Type I interferon-signaling pathway.

**Table 4:** Specific induced pathways in RA patients compared to relatives ACCP- and ACCP+.

Pathway	P Value	Matched Entities	Pathway Entities of Experiment Type
Hs_Unfolded_Protein_Response_WP1939_77024	0.006660059	2	9
Hs_Nucleotide_GPCRs_WP80_68938	0.012675031	2	11
Hs_Inositol_phosphate_metabolism_WP2741_76978	0.012989963	3	31
Hs_BMP_Signalling_and_Regulation_WP1425_74390	0.015936501	1	12
Hs_Metastatic_brain_tumor_WP2249_76471	0.015936501	1	27
Hs_Influenza_A_virus_infection_WP1438_73327	0.015936501	1	12
Hs_Heart_Development_WP1591_73381	0.032906506	3	47
Hs_Blood_Clotting_Cascade_WP272_71361	0.047459427	2	22

### Assessment of genes from the type I interferon pathway, WNT pathway and inflammation as biomarkers for RA

**Fig 4:**
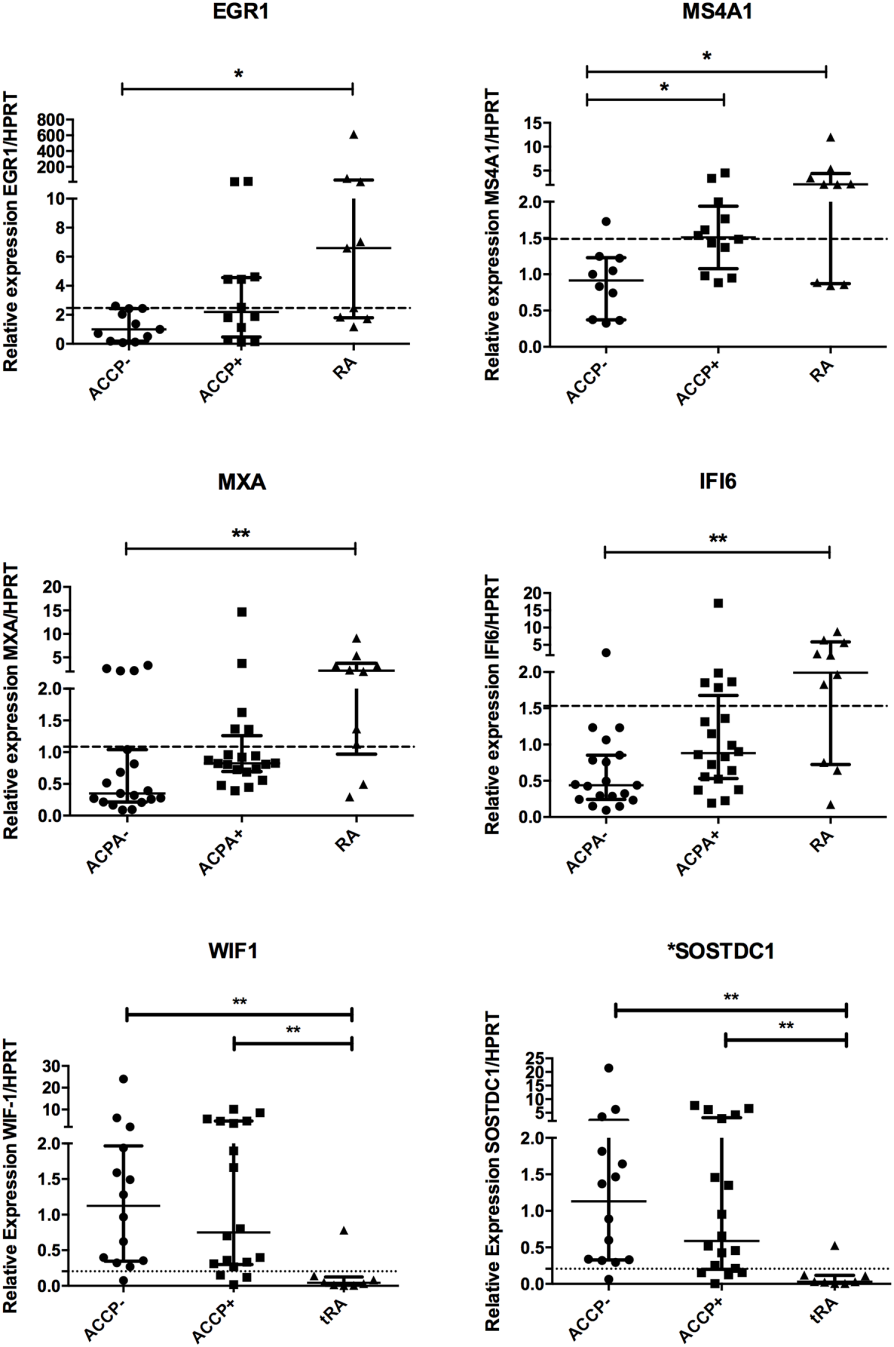
Gene expression of EGR1, MS4A1, MXA, IFI6, WIF1 and SOSTDC1. Gene expression analysis was carried out in cDNA from blood total RNA to assess the relative gene expression profile of selected genes in three groups ACCP-, anti-citrullinated peptide antibodies negative; ACCP+, anti-citrullinated peptide antibodies positive and eRA, early Rheumatoid Arthritis. The graphs depict median ± IQR as descriptive statistics. All relative values used the HPRT expression as reference gene in the 2-ΔΔCt equations. Multiple comparisons tests were made by means of the non-parametric Kruskal-Wallis test. The non-continuous line represents the best cut-off value from ROC analysis. P values of less than 0.05 were considered statistically significant. The * represent significant differences, ** very significant differences and *** extremely significant differences.

**Fig 5:**
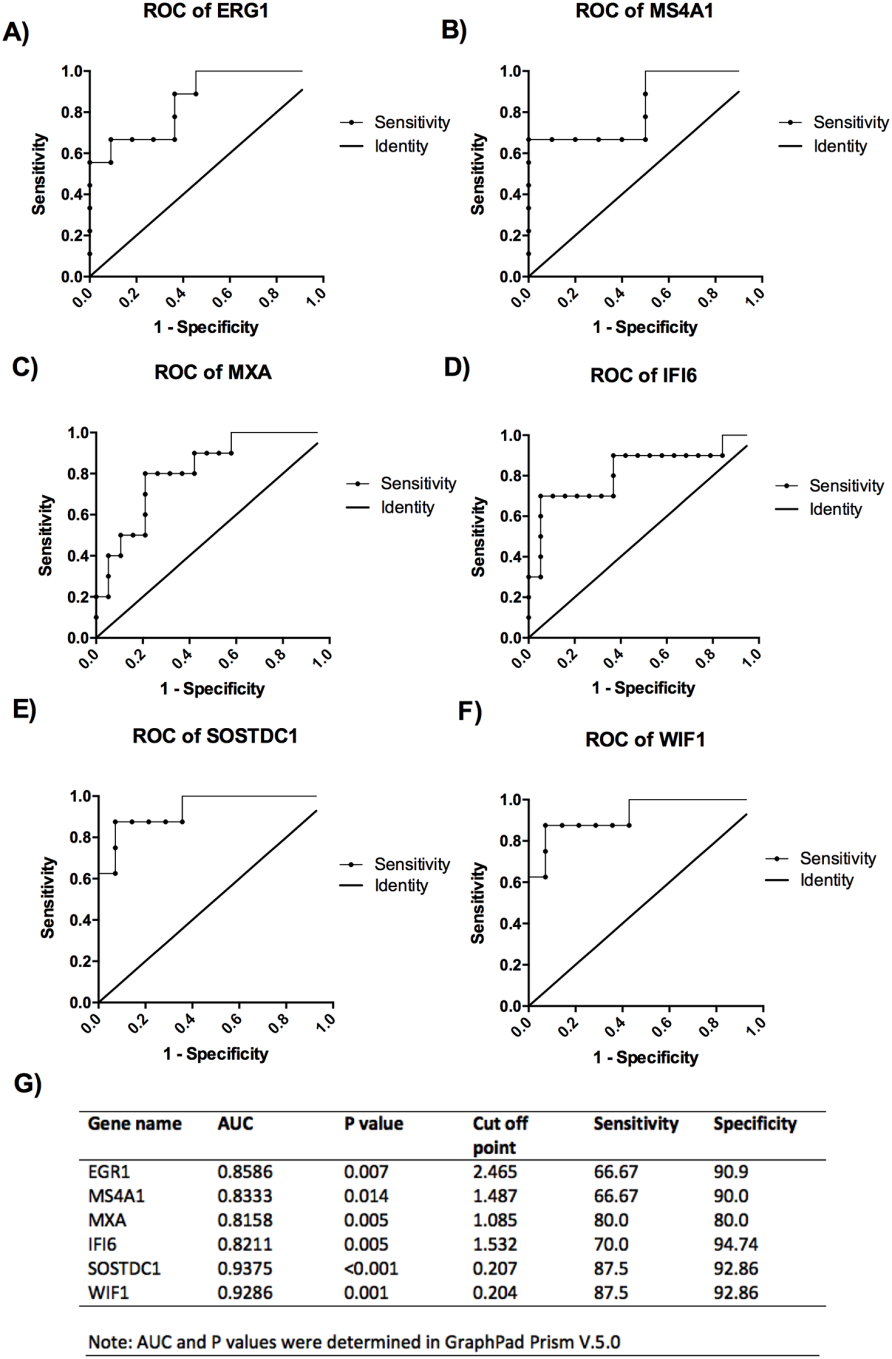
Receiver operating characteristic (ROC) curves of potential biomarkers. The ROC analysis and curves are shown comparing between ACCP- and early RA subjects for all mRNAs A) ERG1, B) MS4A1, C) MXA, D) IFI6, E) SOSTDC1 F) WIF1, additionally in G) a table summarizing the best cut off points and diagnostic performance of such mRNAs is shown. The line represents the line of no discrimination. P values of less than 0.05 were considered statistically significant.

Once that the most relevant pathways were identified, several genes were selected based on their values of fold change (under GSE100191 in NCBI, NIH, Gene Expression Omnibus). Given that several hurdles still remain in RA diagnosis particularly in the early phases of disease. True diagnosis of RA requires identification of biomarkers related to disease progression. Considering that the up-regulation of genes from the high risk ACCP+ to the early RA patients may reflect the progression the disease from a pre-clinical stage of the disease, we analyzed the expression of 6 genes to evaluate their utility as biomarkers. First, as shown in Fig 4, significant differences were found in a multiple comparisons test for the next genes: *EGR1 and MS4A1* (inflammatory pathway); *MXA and IFI6* (Type 1 interferon signature); *SOSTDC* and *WIF1* (Wnt signaling). In order to evaluate the usefulness of some of these genes as biomarkers to identify the RA patients, or high-risk ACCP+ on disease progression, we performed a Receiver Operating Characteristic (ROC) curves. In Fig 5A–5F the ROC analysis showed significant differences compared to the line of no discrimination. Moreover, the AUC for all 6 genes was above 0.8, suggesting their utility as biomarkers. A suggested Cut off point is shown within Fig 5G, with sensitivities similar to those achieved by the immune testing of ACCP and RF (approx. 90%). Remarkably, SOSTDC1 and WIF1, genes of the WNT pathway had the highest AUC (>.92), sensitivity and specificity, and thus may serve as good biomarkers for RA.

## Discussion

In this work we evaluated the transcription profiles associated with the development of RA. Thus, using microarray analysis, we compared healthy individuals at high risk (ACCP+), healthy relatives at low risk (ACCP-) and patients with early RA (RA). We identified an important transcriptional arrest on both the ACCP+ and the early RA groups. This suggests that a strong decrease in transcriptional levels precedes the preclinical stage (ACCP+ relatives) and continues up to the development of RA. Similar results had been described previously in a mouse RA progression model, in which mice that develop RA have a greater number of DRG in the previous stages compared to mice that do not develop RA [18]. We hypothesized that the development of RA could be associated with the loss of tolerance promoted by this strong down regulation event, in which many key regulators that control inflammation and suppress pro-inflammatory molecules are reduced. Our data on the increased expression of inflammatory-associated genes support this view. Microarray analysis was confirmed by qPCR. Several major pathways associated with broad regulation of gene expression were identified: Immune response, MAPK signaling, metabolism, Wnt signaling and type I interferon signature. Their possible participation in such phenomenon is discussed below.

MAPK signaling is involved in several cellular communication pathways associated to chronic inflammatory diseases such as RA, Crohn’s disease and psoriasis [19]. In the group of high-risk ACCP+ relatives, we observed an increased expression of genes involved in the MAPK signal pathway, highlighting PTPN7, DUSP4, STMN1, MKNK1, and FOS. This signal pathway participates directly in the proliferation and differentiation of T and B cells [20, 21], giving rise to a downward trend in the activation of TNFα and IL-1β [20–22]. Other genes, such as PTPN7, which are regulated by ERK1/2 and p38 [23], could be associated with chronic proliferation and avoidance of cell death through apoptosis, as has been reported for the PTPN22 gene, whose high expression has been associated directly with the inflammation and with loss of tolerance in RA [24, 25]. Further research is needed to confirm the role of these signaling pathways in the tolerance loss and clinical autoimmunity onset in RA.

As described before, we also observed in the high-risk ACCP+ subjects a set of genes that belongs to the Wnt signals (wingless-type). It has been reported that Wnt signals participate importantly in both synovial inflammation and in bone remodeling [26, 27]. Ligands of the Wnt pathway, such as wnt4, wnt3, and wnt5a, which participate in cellular proliferation, are also highly expressed in RA [28]. wnt5a induce the expression of pro-inflammatory cytokines such as IL-6, IL-8, IL-15, RANKL, fibronectin and MMP3 [29, 30]. Interestingly, it has been demonstrated that the WNT5A gene has a binding site for STAT3, and that its expression is regulated by this transcription factor [31], playing a very important role in the maintenance of a chronic inflammation state, as well as in the destruction of cartilage in arthritis [32–34]. Furthermore, the Wnt/β-catenin signaling pathway regulates the osteoblast and chondroblast metabolism through the DKK1 and Sclerostin, both upstream inhibitors of the Wnt/β-catenin pathway [35]. DKK1 has been proposed as a biomarker of arthritis in early stages of the disease [36]. Our results clearly show a down regulation of the expression of the negative regulators of the WNT signaling pathway, thus suggesting a higher activity in this signaling pathway. In regards to SOSTDC1 (Sclerostin domain-containing protein 1), a member of the family of sclerostin proteins, little information is known about the functions or the regulation of this gene. WIF1 (Wnt inhibitory factor 1) is a member of the WNT regulators and its role in RA pathogenesis is unknown but it has been suggested that might be implicated in cartilage balance and bone anabolism [37]. To our knowledge, this is the first time that WNT regulators are proposed to be used as biomarkers. Further investigation is needed to clarify the role of WNT signaling in the early stages of the disease and for validation of such biomarkers.

The participation of interferon has been widely reported in autoimmune diseases [38] such as Systemic Lupus Erythematous (SLE) and RA [39, 40]. In our group of ACCP+ high levels of expression occur in genes related to both, type I (IFN-α and IFN-β) and type 2 interferon (IFN-γ). This set includes genes that respond to IFN gamma, such as SOCS3, SOCS1, and MT2A. Those genes have been implicated as important negative regulators of the proliferation and release of inflammatory cytokines such as IL-6 [41, 42]. Recently our group found an important association between mRNA gene expression of the type I interferon signature and the production of autoantibodies, providing strong evidence of the participation of these signaling pathways in the physiopathology of RA even before symptoms onset [13]. Particularly, MXA (Interferon-induced dynamin-like GTPase) and IFI6 (Interferon alpha-inducible protein 6) show a modest potential to be used as biomarkers of early RA. Further research is needed in experimental models of RA to establish a causal link between activation of the type I interferon signature and the production of antibodies and B cell activation.

It is widely known that several cytokines such as tumor necrosis factor alpha (TNF-α), Interleukin (IL)-6, IL-17, IL-2 and others play a pivotal role in the RA inflammatory response [41, 43–49]. Our microarray data is consistent with this widely described RA pathological phenomenon. However, little is known regarding the expression of IL-2, IL-7, and IL-9 in preclinical stages of the disease and in promoting inflammation [50, 51]. Although, recent reports suggest that IL-2 production increases in seropositive arthralgia [52], suggesting that it might be associated to the early stage of the inflammatory process in RA. All these molecules are increased in the established disease [50, 51, 53].

MS4A1 (CD20, also known as membrane-spanning 4-domains subfamily A member) has an increased expression in sinovium and correlates with an increased erosion of bone structures in very early RA [54]. This suggests that the elevation of such molecules could be associated with the expansion of B cell populations responsible for autoantibodies production. This, given that ACPAs have been associated with bone erosions even before clinical symptoms onset [55]. To our knowledge this is the first time that gene expression of MS4A1 is measured in whole blood and proposed as diagnostic biomarker.

EGR-1 is a mammalian transcription factor. Several studies suggest that this molecule is involved in the transcription of metalloproteinases and their specific inhibitors. Also, several reports indicate that EGR1 show an increased expression in sinovial fibroblasts [56] and in infiltrating mononuclear cells in the sinovium regulating proinflammatory molecules production. This depends on the target cells. In mononuclear cells a regulation of PGE2 mediated production of TNF-a [57] was reported. In chondrocytes EGF-1 mediates a decreased expression of extracellular matrix proteins [58] and therefore, it could be regulating the matrix degradation of the sinovium.

The study has several limitations. It is descriptive in nature and the pathways with differential expression need further experimental corroboration. Animal model experiments exploring several candidate molecules and pathways are going to define the role of several of these signaling pathways and their role in RA. The evidence provided in ROC analysis of candidate biomarkers is limited but provides an encouraging prospect for a bigger sample size necessary to verify the utility of the identified genes as biomarker of early stages of RA. In summary, we describe some novel and uncharacterized pathways that could be implicated in the massive down regulation of gene expression that trigger the development of RA. Moreover, from such pathways we identified and evaluated a set of distinctive genes with increased or impaired expression that correlate with the progression of RA with good values of sensitivity and specificity, providing evidence of their potential use as biomarkers of RA. Further studies will be needed to clarify the functional roles of these pathways in the physiopathology of RA as well as to assess the potential use of the identified genes as biomarkers for the early stages of the disease.

## Supporting information

S1 Table Oligonucleotide sequences of the sense and antisense primers for realtime PCR Analysis. (PDF)

S2 Table Up regulated genes in patients with RA vs relatives ACCP-. (PDF)

S3 Table Down regulated genes in patients with RA vs relatives ACCP-. (PDF)

S4 Table Up regulated genes in relatives with ACCP+ vs relatives ACCP-. (PDF)

S5 Table Down regulated genes in relatives with ACCP+ vs relatives with ACCP-. (PDF)

S6 Table Up regulated genes in AR and ACCP+ groups according Venn diagram. (PDF)

S7 Table Down regulated genes in AR and ACCP+ groups according Venn diagram. (PDF)

S8 Table Down regulated genes in AR and up regulated genes in ACCP+ groups according Venn diagram. (PDF)

S9 Table Up regulated genes in AR and down regulated genes in ACCP+ groups according Venn diagram. (PDF)

S10 Table Biological function of the 2 fold change Up-regulated genes according GO analisis in relatives with ACCP+. (PDF)

S11 Table Biological function of the 2 fold change Up-regulated genes according GO analisis in RA. (PDF)

S12 Table Specific induced pathways in relatives with ACCP+ compared to relatives ACCP-. (PDF)

S13 Table Induced pathways in preclinical and clinical AR represented by relatives with ACCP+ and AR patients compared to relatives ACCP-. (PDF)
