# Supplementary material for: Transcriptional signature associated with early rheumatoid arthritis and healthy individuals at high risk to develop the disease
Source: PLoS One. 2018 Mar 27;13(3):e0194205. doi: 10.1371/journal.pone.0194205 (PMC5870959; doi:10.1371/journal.pone.0194205)
Supplement: S1 Table — (PDF) [file pone.0194205.s001.pdf]

Supplementary Table 1. Oligonucleotide sequences of the sense and antisense primers for real-time PCR Analysis

Gene	**Accession number**	**Forward Primer Sequence**	**Reverse Primer Sequence**	**Product Size**
BCL2	NM_000633	gcacctgcacacctggat	agggccaaactgagcaga	126
SERPINB9	BC002538	agataacccttcgcacaacg	cccgatgaatgtcttcctct	143
SERPING1	NM_000062	gaccctgggggactctctac	tcagcggactccaaacttct	69
SNCA	NM_000345	gagtggccattcgacgac	ccctgtttggttttctcagc	119
CX3CL1	NM_002996.3	ccacccctgaaaggctct	gtgggagtgggtccaatg	64
MS4A1	NM_152866.2	agaacaaaatctctactttgatggaac	ggcaaggcctactgctga	96
ETS1	NM_001143820.1	ctgcaggctgttgaaagatg	gggaggaccagtcgtggta	120
EGR1	NM_001964.2	ggtttggctggggtaactg	agccctacgagcacctgac	92
MEF2A	NM_005587.2	tgatgcggaatcataaaatcg	tggaactgtgacagacattgaa	68
SOSTDC1	*NM 015464.2*	*gattttgtaggtgcgtgtgct*	*gattggaggaggctatggaac*	*133*
WIF1	*NM 007191.4*	*gctgatttcacactgctctcc*	*gacaaagcaaactgctcaacc*	*105*
MXA	*NM_002462.3*	*atccagccaccattccaa*	*caacaagttaaatggtatcacagagc*	*61*
IFI6	*NM_002038.3*	*tgcttctcttctctcctccaa*	*gctctccgagcactttttctt*	*147*
