# Supplementary material for: Transcriptional signature associated with early rheumatoid arthritis and healthy individuals at high risk to develop the disease
Source: PLoS One. 2018 Mar 27;13(3):e0194205. doi: 10.1371/journal.pone.0194205 (PMC5870959; doi:10.1371/journal.pone.0194205)
Supplement: S2 Table — (PDF) [file pone.0194205.s002.pdf]

Supplementary Table 2. Up regulated genes in patients with RA vs relatives ACCP-

Gene Symbol	Genbank Accession	Gene Name	Fold Change	Regulation
IRF2BP2	NM_182972	interferon regulatory factor 2 binding protein 2	2.2283545	up
DNM3	NM_015569	dynamin 3	2.179819	up
WDR93	NM_020212	WD repeat domain 93	2.9332693	up
CAST	NM_001042440	calpastatin	2.0139737	up
DHRS7	NM_016029	dehydrogenase/reductase (SDR family) member 7	2.0166013	up
LOC644366	XM_932149	uncharacterized LOC644366	2.5573158	up
SUMO1	NM_003352	SMT3 suppressor of mif two 3 homolog 1 (S. cerevisiae)	2.1390934	up
FAM55C	NM_001134456	family with sequence similarity 55, member C	2.027618	up
			2.0528383	up
SNCA	NM_007308	synuclein, alpha (non A4 component of amyloid precursor)	3.070638	up
ETV4	NM_001079675	ets variant 4	2.078938	up
	DB073168		2.5304694	up
DDX24	NM_020414	DEAD (Asp-Glu-Ala-Asp) box polypeptide 24	2.1985526	up
BCL2	NM_000633	B-cell CLL/lymphoma 2	2.48382	up
TMF1	NM_007114	TATA element modulatory factor 1	2.0867357	up
OC90	NM_001080399	otoconin 90	2.187347	up
MORC3	NM_015358	MORC family CW-type zinc finger 3	2.0230103	up
AFTPH	NM_203437	aftiphilin	2.162457	up
SRPK3	NM_014370	SRSF protein kinase 3	2.1222854	up
FAM101B	NM_182705	family with sequence similarity 101, member B	2.2707503	up
LYRM2	NM_020466	LYR motif containing 2	2.2003713	up
	XM_003119905		2.442962	up
MRTO4	NM_016183	mRNA turnover 4 homolog (S. cerevisiae)	2.3046987	up
TNS1	NM_022648	tensin 1	2.028129	up
XK	NM_021083	X-linked Kx blood group (McLeod syndrome)	4.1446037	up
ACTR3	NM_005721	ARP3 actin-related protein 3 homolog (yeast)	2.036796	up
LOC654433	NR_015377	uncharacterized LOC654433	2.5371802	up
DMXL1	NM_005509	Dmx-like 1	2.0011044	up
C6orf105	NM_032744	chromosome 6 open reading frame 105	2.0007164	up
CTNNB1	NM_001904	catenin (cadherin-associated protein), beta 1, 88kDa	2.138463	up
VNN1	NM_004666	vanin 1	2.690406	up
SPRR2A	NM_005988	small proline-rich protein 2A	2.2807808	up
KNDC1	NM_152643	kinase non-catalytic C-lobe domain (KIND) containing 1	2.3655996	up
CSN1S2AP	NR_003720	casein alpha s2-like A, pseudogene	2.8103547	up
ETS1	NM_005238	v-ets erythroblastosis virus E26 oncogene homolog 1 (avian)	2.106572	up
CPNE6	NM_006032	copine VI (neuronal)	2.5055895	up
NCK2	NM_003581	NCK adaptor protein 2	2.4129615	up
EFCAB3	NM_173503	EF-hand calcium binding domain 3	3.4859395	up

			2.2170436	up
LOC729305	XR_132809	uncharacterized LOC729305	2.516636	up
ADAM21	NM_003813	ADAM metallopeptidase domain 21	2.5039046	up
LOC643529	NR_038382	hCG2024094	2.3166313	up
	AF469204		2.0560994	up
OTP	NM_032109	orthopedia homeobox	2.885809	up
LOC100131138	NR_036513	uncharacterized LOC100131138	2.896989	up
ABCC13	NR_003088	ATP-binding cassette, sub-family C (CFTR/MRP), member 13, pseudogene	2.7825346	up
			2.3469405	up
CD99L2	BC025729	CD99 molecule-like 2	3.9024413	up
PBX1	NM_002585	pre-B-cell leukemia homeobox 1	2.5033636	up
TRAM1	NM_014294	translocation associated membrane protein 1	2.0305283	up
C6orf97	NM_025059	chromosome 6 open reading frame 97	2.0603268	up
KCNK17	NM_031460	potassium channel, subfamily K, member 17	2.0954254	up
LPAR6	NM_005767	lysophosphatidic acid receptor 6	2.3590717	up
TAF1B	NM_005680	TATA box binding protein (TBP)-associated factor, RNA polymerase I, B, 63kDa	2.0075934	up
EPHB2	NM_004442	EPH receptor B2	2.2783122	up
CLEC7A	NM_197947	C-type lectin domain family 7, member A	2.0963047	up
TCEA1	NM_006756	transcription elongation factor A (SII), 1	2.1890926	up
			2.4983416	up
CNTNAP2	NM_014141	contactin associated protein-like 2	2.3479564	up
TRUB1	NM_139169	TruB pseudouridine (psi) synthase homolog 1 (E. coli)	2.062541	up
CLK1	NM_001162407	CDC-like kinase 1	2.8655984	up
LOC401127	NR_026854	WD repeat domain 5 pseudogene	2.8764637	up
	AK093119		2.2098558	up
GGH	NM_003878	gamma-glutamyl hydrolase (conjugase, folylpolygammaglutamyl hydrolase)	2.1032088	up
CBLL1	NM_024814	Cas-Br-M (murine) ecotropic retroviral transforming sequence-like 1	2.181801	up
PMEL	NM_006928	premelanosome protein	2.0764756	up
TRAT1	NM_016388	T cell receptor associated transmembrane adaptor 1	2.0102658	up
ERBB3	NM_001982	v-erb-b2 erythroblastic leukemia viral oncogene homolog 3 (avian)	2.1594281	up
	CU677518		2.5904014	up
NXF3	NM_022052	nuclear RNA export factor 3	2.1772964	up
RASGEF1B	NM_152545	RasGEF domain family, member 1B	2.379134	up
PRKAA1	NM_206907	protein kinase, AMP-activated, alpha 1 catalytic subunit	2.0089328	up
NDFIP2	NM_019080	Nedd4 family interacting protein 2	2.0371866	up
			3.2509692	up
SYT15	NM_181519	synaptotagmin XV	2.228985	up
DSCR8	NR_026838	Down syndrome critical region gene 8	2.3851957	up
MRC2	NM_006039	mannose receptor, C type 2	2.2330055	up
VNN2	NM_004665	vanin 2	2.119297	up

RB1	NM_000321	retinoblastoma 1	2.1439729	up
CXorf57	NM_018015	chromosome X open reading frame 57	2.147257	up
LOC100653325	XM_003403750	uncharacterized LOC100653325	2.908699	up
PRSS33	NM_152891	protease, serine, 33	2.6216602	up
CASD1	NM_022900	CAS1 domain containing 1	2.2140183	up
LOC149837	NR_015406	uncharacterized LOC149837	2.6367295	up
BNIP3L	NM_004331	BCL2/adenovirus E1B 19kDa interacting protein 3-like	3.1700666	up
LINC00240	NR_026775	long intergenic non-protein coding RNA 240	2.9120626	up
ANP32E	NM_030920	acidic (leucine-rich) nuclear phosphoprotein 32 family, member E	2.136219	up
ARHGAP40	NM_001164431	Rho GTPase activating protein 40	2.0468392	up
ASPSCR1	AK127159	alveolar soft part sarcoma chromosome region, candidate 1	2.424498	up
PANK3	BX648891	pantothenate kinase 3	2.084829	up
ST7L	NM_138729	suppression of tumorigenicity 7 like	2.4821072	up
DNHD1	NM_144666	dynein heavy chain domain 1	2.0509136	up
SUV420H1	NM_017635	suppressor of variegation 4-20 homolog 1 (Drosophila)	2.4649494	up
GPR78	NM_080819	G protein-coupled receptor 78	2.1594136	up
LYZL6	NM_020426	lysozyme-like 6	2.1683214	up
FZD6	NM_003506	frizzled family receptor 6	2.0556123	up
GUSBP1	NR_027027	glucuronidase, beta pseudogene 1	2.0721304	up
ZBTB6	NM_006626	zinc finger and BTB domain containing 6	2.0950403	up
A2ML1	NM_144670	alpha-2-macroglobulin-like 1	2.2553496	up
PCGEM1	NR_002769	prostate-specific transcript 1 (non-protein coding)	2.5509214	up
	DA372173		2.2742875	up
SLC25A32	NM_030780	solute carrier family 25, member 32	2.44969	up
UBE3A	NM_130839	ubiquitin protein ligase E3A	2.2440147	up
KLHDC8A	NM_018203	kelch domain containing 8A	2.1684434	up
DNAJC6	NM_014787	DnaJ (Hsp40) homolog, subfamily C, member 6	2.1866822	up
DNAJA2	NM_005880	DnaJ (Hsp40) homolog, subfamily A, member 2	2.431748	up
HRH4	NM_021624	histamine receptor H4	2.6878061	up
CYP2B6	NM_000767	cytochrome P450, family 2, subfamily B, polypeptide 6	2.3794148	up
LOC646851		putative uncharacterized protein LOC388900	2.0696096	up
DPPA5	NM_001025290	developmental pluripotency associated 5	2.4035685	up
VSIG10	NM_019086	V-set and immunoglobulin domain containing 10	2.181112	up
LRP12	NM_013437	low density lipoprotein receptor-related protein 12	2.2275164	up
USP1	NM_003368	ubiquitin specific peptidase 1	2.1401176	up
PPCS		phosphopantothenoylcysteine synthetase	2.4874487	up
SKAP2	NM_003930	src kinase associated phosphoprotein 2	2.0402973	up
E2F2	NM_004091	E2F transcription factor 2	3.4562614	up
			2.1896882	up
CX3CL1	NM_002996	chemokine (C-X3-C motif) ligand 1	2.070115	up
RSRC1	NM_016625	arginine/serine-rich coiled-coil 1	2.0188031	up

ICA1	NM_004968	islet cell autoantigen 1, 69kDa	2.4965518	up
PLEKHF2	NM_024613	pleckstrin homology domain containing, family F (with FYVE domain) member 2	2.0311363	up
ASB7	NM_198243	ankyrin repeat and SOCS box containing 7	2.0143614	up
EGR2	NM_000399	early growth response 2	3.0035136	up
ZNF235	NM_004234	zinc finger protein 235	2.211763	up
MYH7	NM_000257	myosin, heavy chain 7, cardiac muscle, beta	2.426603	up
LYRM5	NM_001001660	LYR motif containing 5	2.1336875	up
MKRN1	NM_013446	makorin ring finger protein 1	2.173403	up
TAS2R50	NM_176890	taste receptor, type 2, member 50	2.1007218	up
PGGT1B	NM_005023	protein geranylgeranyltransferase type I, beta subunit	2.152535	up
F13A1	NM_000129	coagulation factor XIII, A1 polypeptide	2.1604183	up
			2.987308	up
C1S	NM_001734	complement component 1, s subcomponent	2.8285618	up
HLF	NM_002126	hepatic leukemia factor	2.1526155	up
TREML4	NM_198153	triggering receptor expressed on myeloid cells-like 4	2.3565435	up
ECHDC1	NM_018479	enoyl CoA hydratase domain containing 1	2.1612232	up
HIST2H2BF	NM_001161334	histone cluster 2, H2bf	2.5397558	up
RBM44	NM_001080504	RNA binding motif protein 44	2.0121586	up
DIXDC1	NM_001037954	DIX domain containing 1	2.7631285	up
ITPKB	NM_002221	inositol-trisphosphate 3-kinase B	2.588736	up
OLFM4	NM_006418	olfactomedin 4	2.48169	up
	XR_109175		2.015282	up
SPATA21	NM_198546	spermatogenesis associated 21	2.326341	up
ATP6V0E1	NM_003945	ATPase, H+ transporting, lysosomal 9kDa, V0 subunit e1	2.058492	up
			2.0634086	up
RAB39B	NM_171998	RAB39B, member RAS oncogene family	2.1268976	up
			2.250161	up
WBP5	NM_016303	WW domain binding protein 5	2.0082836	up
	DB462629		3.4639966	up
UHRF1	NM_013282	ubiquitin-like with PHD and ring finger domains 1	2.0640435	up
THAP9	NM_024672	THAP domain containing 9	2.0142074	up
TFF3	NM_003226	trefoil factor 3 (intestinal)	2.442851	up
CXCL12	NM_001033886	chemokine (C-X-C motif) ligand 12	2.420027	up
LARP1B	NM_032239	La ribonucleoprotein domain family, member 1B	2.1858144	up
ZC3H14	NM_024824	zinc finger CCCH-type containing 14	2.1468008	up
FAM105B	NM_138348	family with sequence similarity 105, member B	2.0286233	up
BCAT1	NM_005504	branched chain amino-acid transaminase 1, cytosolic	2.249215	up
EMP1	NM_001423	epithelial membrane protein 1	2.1554358	up
			2.6047971	up
XPR1	NM_004736	xenotropic and polytropic retrovirus receptor 1	2.2743802	up

WBSCR17	NM_022479	Williams-Beuren syndrome chromosome region 17	3.189225	up
GEMIN2	NM_003616	gem (nuclear organelle) associated protein 2	2.307731	up
FLJ43663	NR_015431	uncharacterized LOC378805	2.073444	up
MAMSTR	NM_182574	MEF2 activating motif and SAP domain containing transcriptional regulator	2.463689	up
TTTY12	NR_001551	testis-specific transcript, Y-linked 12 (non-protein coding)	2.6459787	up
NOV	NM_002514	nephroblastoma overexpressed gene	2.0665514	up
			2.8055496	up
LOC100505876	NR_037879	uncharacterized LOC100505876	2.0519989	up
FAM76B	NM_144664	family with sequence similarity 76, member B	2.1129181	up
	XM_931148		2.4586365	up
YEATS4	NM_006530	YEATS domain containing 4	2.0349157	up
PHC3	NM_024947	polyhomeotic homolog 3 (Drosophila)	2.1305237	up
SERPING1		serpin peptidase inhibitor, clade G (C1 inhibitor), member 1	2.3713517	up
HAS1	NM_001523	hyaluronan synthase 1	2.2748744	up
AFAP1	NM_001134647	actin filament associated protein 1	2.338585	up
RGS12	NM_002926	regulator of G-protein signaling 12	2.4563916	up
			2.6360404	up
KRT1	NM_006121	keratin 1	3.2459288	up
MFAP3L	NM_021647	microfibrillar-associated protein 3-like	2.8052254	up
PPP1R3G	NM_001145115	protein phosphatase 1, regulatory subunit 3G	2.0523548	up
KIAA0664	NM_015229	KIAA0664	3.1315942	up
NPPA	NM_006172	natriuretic peptide A	2.326938	up
LRTM1	NM_020678	leucine-rich repeats and transmembrane domains 1	2.12895	up
TAF1	NM_004606	TAF1 RNA polymerase II, TATA box binding protein (TBP)-associated factor, 250kDa	2.2413568	up
TAF4B	NM_005640	TAF4b RNA polymerase II, TATA box binding protein (TBP)-associated factor, 105kDa	2.303319	up
DCT	NM_001922	dopachrome tautomerase (dopachrome delta-isomerase, tyrosine-related protein 2)	2.9082742	up
PTPRK	NM_002844	protein tyrosine phosphatase, receptor type, K	2.1001608	up
			2.8254147	up
ATXN1	NM_000332	ataxin 1	2.852338	up
FEM1B	NM_015322	fem-1 homolog b (C. elegans)	2.2095041	up
STC1	NM_003155	stanniocalcin 1	2.8113954	up
ERAP1	NM_001040458	endoplasmic reticulum aminopeptidase 1	2.2850757	up
SRSF8	NM_032102	serine/arginine-rich splicing factor 8	2.1073203	up
TGM4	NM_003241	transglutaminase 4 (prostate)	2.0748248	up
ACTR2	NM_001005386	ARP2 actin-related protein 2 homolog (yeast)	2.082664	up
DKFZP434H168	NR_026889	uncharacterized LOC26077	2.367622	up
NT5C1A	NM_032526	5'-nucleotidase, cytosolic IA	3.2178488	up
DACT1	NM_016651	dapper, antagonist of beta-catenin, homolog 1 (Xenopus laevis)	2.8586888	up
			2.6001701	up

C18orf10	NM_015476	chromosome 18 open reading frame 10	2.4458992	up
TBC1D9	NM_015130	TBC1 domain family, member 9 (with GRAM domain)	2.0756447	up
			2.2269216	up
ZC3H12B	NM_001010888	zinc finger CCCH-type containing 12B	2.7539322	up
KRTAP15-1	NM_181623	keratin associated protein 15-1	2.3450232	up
PVR	NM_006505	poliovirus receptor	2.329061	up
NDUFA5	NM_005000	NADH dehydrogenase (ubiquinone) 1 alpha subcomplex, 5, 13kDa	2.07942	up
FSD1	NM_024333	fibronectin type III and SPRY domain containing 1	3.3686712	up
ZNF334	NM_199441	zinc finger protein 334	2.1063576	up
RPIA	NM_144563	ribose 5-phosphate isomerase A	2.1208103	up
DCAF16	NM_017741	DDB1 and CUL4 associated factor 16	2.0078003	up
UGDH	NM_003359	UDP-glucose 6-dehydrogenase	2.3007631	up
C8orf31	NM_173687	chromosome 8 open reading frame 31	2.0861464	up
LRRD1	NM_001161528	leucine-rich repeats and death domain containing 1	2.6135151	up
			2.67043	up
SCP2	NM_002979	sterol carrier protein 2	2.0640256	up
INADL	NM_176877	InaD-like (Drosophila)	2.3543868	up
JKAMP	NM_016475	JNK1/MAPK8-associated membrane protein	3.0562344	up
COL9A2	BC065715	collagen, type IX, alpha 2	2.650093	up
CDC5L	NM_001253	CDC5 cell division cycle 5-like (S. pombe)	2.244164	up
			2.273086	up
S100P	NM_005980	S100 calcium binding protein P	4.51571	up
FLJ40536	AK097855	FLJ40536 protein	3.1316957	up
TMEM212	NM_001164436	transmembrane protein 212	2.082301	up
C1orf185	NM_001136508	chromosome 1 open reading frame 185	2.2304368	up
	AL831934		2.4530811	up
TRIOBP	NM_138632	TRIO and F-actin binding protein	3.1698763	up
RAB2B	NM_032846	RAB2B, member RAS oncogene family	2.0913925	up
SRSF1	NM_001078166	serine/arginine-rich splicing factor 1	2.0052543	up
TCL6	BC041075	T-cell leukemia/lymphoma 6 (non-protein coding)	3.3149445	up
KLKB1	NM_000892	kallikrein B, plasma (Fletcher factor) 1	2.353181	up
PPP6C	NM_002721	protein phosphatase 6, catalytic subunit	2.0964527	up
KANK2	NM_015493	KN motif and ankyrin repeat domains 2	2.0405536	up
EIF5	NM_001969	eukaryotic translation initiation factor 5	2.0252223	up
LOC644192	XR_111553	uncharacterized LOC644192	2.560938	up
QRSL1	NM_018292	glutaminyl-tRNA synthase (glutamine-hydrolyzing)-like 1	2.1386185	up
LOC100130503	XM_001717149	uncharacterized LOC100130503	3.0229201	up
C15orf54	NM_207445	chromosome 15 open reading frame 54	2.5744312	up
PCDH9	BC150296	protocadherin 9	2.9098659	up
OLFM3	NM_058170	olfactomedin 3	2.0711882	up
ERV18-1	AK126787	endogenous retrovirus group 18, member 1	2.1447368	up

BNIP3L	NM_004331	BCL2/adenovirus E1B 19kDa interacting protein 3-like	2.0753558	up
KIAA1199	NM_018689	KIAA1199	3.0143433	up
			2.2138634	up
C9orf30-TMEFF1	NM_001198812	C9orf30-TMEFF1 readthrough	3.3958824	up
MYZAP	NM_001018100	myocardial zonula adherens protein	2.1927118	up
CALM2	NM_001743	calmodulin 2 (phosphorylase kinase, delta)	2.0738442	up
COX11	NM_004375	COX11 cytochrome c oxidase assembly homolog (yeast)	2.217919	up
PTEN	NM_000314	phosphatase and tensin homolog	2.2400694	up
LCN1	NM_002297	lipocalin 1	2.2093573	up
RCAN2	NM_005822	regulator of calcineurin 2	2.8625188	up
PAQR3	NM_001040202	progestin and adipoQ receptor family member III	3.2449074	up
KCND3	NM_004980	potassium voltage-gated channel, Shal-related subfamily, member 3	2.0554535	up
PTGFRN	NM_020440	prostaglandin F2 receptor negative regulator	3.1173081	up
SP4	NM_003112	Sp4 transcription factor	2.0376666	up
RBM3	NM_006743	RNA binding motif (RNP1, RRM) protein 3	2.1024055	up
NR3C1	NM_001018077	nuclear receptor subfamily 3, group C, member 1 (glucocorticoid receptor)	2.006029	up
HACE1	NM_020771	HECT domain and ankyrin repeat containing, E3 ubiquitin protein ligase 1	2.025491	up
PPP1CB	NM_002709	protein phosphatase 1, catalytic subunit, beta isozyme	2.2256777	up
ZBTB8A	NM_001040441	zinc finger and BTB domain containing 8A	2.1779	up
LHFPL5	NM_182548	lipoma HMGIC fusion partner-like 5	2.1454184	up
LOC146336	NR_027242	uncharacterized LOC146336	2.5623827	up
MEF2A	NM_001171894	myocyte enhancer factor 2A	2.020954	up
OR51E1	NM_152430	olfactory receptor, family 51, subfamily E, member 1	2.3279693	up
COLEC10	NM_006438	collectin sub-family member 10 (C-type lectin)	2.115921	up
MAP7	NM_003980	microtubule-associated protein 7	2.1426651	up
MS4A1	NM_152866	membrane-spanning 4-domains, subfamily A, member 1	2.8324542	up
AKR1CL1	NR_027916	aldo-keto reductase family 1, member C-like 1	2.9109092	up
TSPYL6	NM_001003937	TSPY-like 6	3.3759797	up
C2orf69	NM_153689	chromosome 2 open reading frame 69	2.2670038	up
ZFHX4	NM_024721	zinc finger homeobox 4	3.385703	up
ROBO3	NM_022370	roundabout, axon guidance receptor, homolog 3 (Drosophila)	2.0279005	up
AKD1	NM_145025	adenylate kinase domain containing 1	2.197917	up
RGSL1	NM_001137669	regulator of G-protein signaling like 1	3.459836	up
SRP72	NM_006947	signal recognition particle 72kDa	2.125636	up
GRIK1-AS1	NR_027021	GRIK1 antisense RNA 1 (non-protein coding)	2.1970606	up
WSB2	NM_018639	WD repeat and SOCS box containing 2	2.034205	up
TPD52L3	NM_001001874	tumor protein D52-like 3	3.119155	up
NR3C1	NM_001018077	nuclear receptor subfamily 3, group C, member 1 (glucocorticoid receptor)	2.1705637	up
WNT4	NM_030761	wingless-type MMTV integration site family, member 4	2.174202	up
IGFL2	NM_001002915	IGF-like family member 2	2.4721038	up

ANKRD62	XM_003118756	ankyrin repeat domain 62	2.1289215	up
ADORA1	NM_000674	adenosine A1 receptor	2.4411824	up
HSPA5	NM_005347	heat shock 70kDa protein 5 (glucose-regulated protein, 78kDa)	2.031998	up
EGR1	NM_001964	early growth response 1	7.548753	up
APOBEC4	NM_203454	apolipoprotein B mRNA editing enzyme, catalytic polypeptide-like 4 (putative)	2.7338746	up
			2.409002	up
AGTR1	NM_031850	angiotensin II receptor, type 1	2.2404194	up
EIF2S1	NM_004094	eukaryotic translation initiation factor 2, subunit 1 alpha, 35kDa	2.2701647	up
ZIC1	NM_003412	Zic family member 1	2.0940816	up
LDHA	NM_005566	lactate dehydrogenase A	2.2790577	up
PNMA2	NM_007257	paraneoplastic antigen MA2	2.2493417	up
LMLN	NM_033029	leishmanolysin-like (metallopeptidase M8 family)	3.6547103	up
ZMAT1	NM_001011657	zinc finger, matrin-type 1	2.0963748	up
GNN	NR_027249	Grp94 neighboring nucleotidase pseudogene	2.4237833	up
PPP4R1L	NR_003505	protein phosphatase 4, regulatory subunit 1-like	2.1227238	up
POLR2F		polymerase (RNA) II (DNA directed) polypeptide F	2.1393638	up
HMGCS1	NM_002130	3-hydroxy-3-methylglutaryl-CoA synthase 1 (soluble)	2.22892	up
PWWP2A	NM_052927	PWWP domain containing 2A	2.0591938	up
FGF1	NM_000800	fibroblast growth factor 1 (acidic)	2.0427027	up
WNT5A	NM_003392	wingless-type MMTV integration site family, member 5A	2.52388	up
SEMA5B	NM_001031702	sema domain, seven thrombospondin repeats (type 1 and type 1-like), transmembrane domain (TM) and short cytoplasmic domain, (semaphorin) 5B	3.1140442	up
			2.5068834	up
TANK	NM_133484	TRAF family member-associated NFKB activator	2.1689553	up
PTPRJ	NM_001098503	protein tyrosine phosphatase, receptor type, J	2.8290167	up
INSIG1	NM_005542	insulin induced gene 1	2.4062417	up
HCAR2	NM_177551	hydroxycarboxylic acid receptor 2	2.0337234	up
LGALSL	NM_014181	lectin, galactoside-binding-like	2.2940598	up
GPX1	NM_201397	glutathione peroxidase 1	2.0866086	up
LOC100128278	XR_110306	uncharacterized LOC100128278	2.0862908	up
PAGE2B	NM_001015038	P antigen family, member 2B	2.3100486	up
NR2C1	NM_001032287	nuclear receptor subfamily 2, group C, member 1	2.2078183	up
BBS7	NM_018190	Bardet-Biedl syndrome 7	2.3186517	up
LOC254312	NR_015413	uncharacterized LOC254312	2.2718897	up
LRRN1	NM_020873	leucine rich repeat neuronal 1	2.6343048	up
LOC100505648	NR_040058	uncharacterized LOC100505648	2.584224	up
ATP6V1E1	NM_001696	ATPase, H+ transporting, lysosomal 31kDa, V1 subunit E1	2.3589697	up
ARGLU1	AK304751	arginine and glutamate rich 1	2.158685	up
LINC00239	NR_026774	long intergenic non-protein coding RNA 239	2.4558783	up
TFPI	NM_006287	tissue factor pathway inhibitor (lipoprotein-associated coagulation inhibitor)	2.8298364	up

MMAB	NM_052845	methylmalonic aciduria (cobalamin deficiency) cblB type	2.0586534	up
			2.0243075	up
ARID5B	NM_032199	AT rich interactive domain 5B (MRF1-like)	2.1512454	up
FAM149B1	NM_173348	family with sequence similarity 149, member B1	2.105633	up
MOB4	NM_015387	MOB family member 4, phocein	2.1700034	up
TXNDC17	NM_032731	thioredoxin domain containing 17	2.0698392	up
			2.062969	up
METTL21A	NM_001127395	methyltransferase like 21A	2.144304	up
HNRNPA2B1	NM_002137	heterogeneous nuclear ribonucleoprotein A2/B1	2.6643212	up
FLI1	NM_002017	Friend leukemia virus integration 1	2.0435832	up
LDOC1	NM_012317	leucine zipper, down-regulated in cancer 1	2.162084	up
	JF834318		2.0780642	up
ANO6	NM_001025356	anoctamin 6	2.251815	up
SPATA18	NM_145263	spermatogenesis associated 18 homolog (rat)	2.1164663	up
TRIP12	NM_004238	thyroid hormone receptor interactor 12	2.0887654	up
STK17B	NM_004226	serine/threonine kinase 17b	2.2759771	up
TRPA1	NM_007332	transient receptor potential cation channel, subfamily A, member 1	2.3513865	up
HTN1	NM_002159	histatin 1	2.5871925	up
C20orf141	NM_080739	chromosome 20 open reading frame 141	2.1620407	up
			2.3585575	up
HNRPDL	NM_031372	heterogeneous nuclear ribonucleoprotein D-like	2.0329182	up
C17orf42	NM_024683	chromosome 17 open reading frame 42	2.3261764	up
DEFB126	NM_030931	defensin, beta 126	2.0526075	up
CLIP4	NM_024692	CAP-GLY domain containing linker protein family, member 4	2.490263	up
NCBP1		nuclear cap binding protein subunit 1, 80kDa	2.448397	up
OR5AN1	NM_001004729	olfactory receptor, family 5, subfamily AN, member 1	2.1693134	up
DNAH14		dynein, axonemal, heavy chain 14	2.2328424	up
CHST9	NM_031422	carbohydrate (N-acetylgalactosamine 4-0) sulfotransferase 9	2.297745	up
PRSS36	NM_173502	protease, serine, 36	2.410336	up
ZNF804B	NM_181646	zinc finger protein 804B	2.3050272	up
SERPINB9	NM_004155	serpin peptidase inhibitor, clade B (ovalbumin), member 9	2.2028115	up
ARPP21	NM_016300	cAMP-regulated phosphoprotein, 21kDa	3.719478	up
ZNF271	NR_024565	zinc finger protein 271	2.343139	up
			2.427121	up
WDR49	AK097556	WD repeat domain 49	2.124233	up
AKAP6	NM_004274	A kinase (PRKA) anchor protein 6	3.412468	up
RIN2	NM_018993	Ras and Rab interactor 2	2.0434923	up
MKL2	NM_014048	MKL/myocardin-like 2	2.0342753	up
ZNF673	AK097159	zinc finger family member 673	2.2941186	up
SLC12A5	NM_020708	solute carrier family 12 (potassium/chloride transporter), member 5	2.1169405	up
FAM171B	NM_177454	family with sequence similarity 171, member B	2.3244078	up

POU2AF1	NM_006235	POU class 2 associating factor 1	2.0351043	up
LRRTM2	NM_015564	leucine rich repeat transmembrane neuronal 2	2.0155501	up
	XM_001721393		2.6143386	up
SPTA1	NM_003126	spectrin, alpha, erythrocytic 1 (elliptocytosis 2)	2.798148	up
			3.8620672	up
DEFA4	NM_001925	defensin, alpha 4, corticostatin	2.1196764	up
RBM12	NM_006047	RNA binding motif protein 12	2.0118887	up
			2.006014	up
METAP2	NM_006838	methionyl aminopeptidase 2	2.1589792	up
FONG	NR_034096	uncharacterized LOC348751	2.1759725	up
CLIC4	NM_013943	chloride intracellular channel 4	2.7739673	up
			4.2431383	up
EVI2A	NM_001003927	ecotropic viral integration site 2A	2.1364188	up
			2.3581214	up
CLCN4	NM_001830	chloride channel 4	2.0227542	up
PPAPDC3		phosphatidic acid phosphatase type 2 domain containing 3	2.159848	up
CNGA2	NM_005140	cyclic nucleotide gated channel alpha 2	2.0578973	up
CCL11	NM_002986	chemokine (C-C motif) ligand 11	2.0526867	up
CPEB4	NM_030627	cytoplasmic polyadenylation element binding protein 4	2.160189	up
MCAT	NM_173467	malonyl CoA:ACP acyltransferase (mitochondrial)	2.3976038	up
MB21D2	NM_178496	Mab-21 domain containing 2	2.573574	up
TDH	NR_001578	L-threonine dehydrogenase	2.354822	up
LIMD1	NM_014240	LIM domains containing 1	2.399504	up
NAP1L1	NM_139207	nucleosome assembly protein 1-like 1	2.209823	up
YTHDF3	NM_152758	YTH domain family, member 3	2.0574346	up
SENP6	NM_015571	SUMO1/sentrin specific peptidase 6	2.0189803	up
			3.1574576	up
XG	NM_175569	Xg blood group	2.3177845	up
C6orf64	NM_018322	chromosome 6 open reading frame 64	2.220312	up
SLITRK5	NM_015567	SLIT and NTRK-like family, member 5	2.0472965	up
CPNE1	NM_003915	copine I	2.5574238	up
PPP2R3C	NM_017917	protein phosphatase 2, regulatory subunit B'', gamma	2.1267047	up
CNTNAP3	NM_033655	contactin associated protein-like 3	2.08096	up
ZNF367	NM_153695	zinc finger protein 367	2.1370528	up
C20orf54	NM_033409	chromosome 20 open reading frame 54	2.5945551	up
			3.6786578	up
MYO3A	BC036079	myosin IIIA	2.5491035	up
ZNF664	NM_152437	zinc finger protein 664	2.3150718	up
GCNT1	NM_001097634	glucosaminyl (N-acetyl) transferase 1, core 2	2.0211456	up
ANXA1	NM_000700	annexin A1	2.0005891	up
TTC25	NM_031421	tetratricopeptide repeat domain 25	2.5326748	up

APOL6	NM_030641	apolipoprotein L, 6	2.4373572	up
YIPF6	NM_173834	Yip1 domain family, member 6	2.0629535	up
GSTA7P	NR_033760	glutathione S-transferase alpha 7, pseudogene	9.545928	up
	BX103737		2.3013823	up
KRTDAP	NM_207392	keratinocyte differentiation-associated protein	2.9482884	up
ZNF365	NM_014951	zinc finger protein 365	2.1590126	up
CYP39A1	NM_016593	cytochrome P450, family 39, subfamily A, polypeptide 1	2.3996668	up
WFDC2	NM_006103	WAP four-disulfide core domain 2	4.569528	up
ZCCHC7	NM_032226	zinc finger, CCHC domain containing 7	2.0827682	up
LOC645195	AK123450	uncharacterized LOC645195	2.0309558	up
HEMGN	NM_018437	hemogen	2.504413	up
F5	NM_000130	coagulation factor V (proaccelerin, labile factor)	2.1552105	up
DNAJC15	NM_013238	DnaJ (Hsp40) homolog, subfamily C, member 15	2.3551683	up
FAM46C	NM_017709	family with sequence similarity 46, member C	2.6403832	up
DNAJC21	NM_194283	DnaJ (Hsp40) homolog, subfamily C, member 21	2.2258818	up
CHTOP	AK097870	chromatin target of PRMT1	2.005364	up
MSL2	NM_018133	male-specific lethal 2 homolog (Drosophila)	2.1028452	up
HP	NM_005143	haptoglobin	2.1156824	up
TRIM58	NM_015431	tripartite motif containing 58	2.4587033	up
FECH	NM_001012515	ferrochelatase	3.7838116	up
	AK124695		3.2575479	up
WNK1	NM_001184985	WNK lysine deficient protein kinase 1	2.0270514	up
SSB	NM_003142	Sjogren syndrome antigen B (autoantigen La)	2.0013916	up
HNRNPH3	NM_012207	heterogeneous nuclear ribonucleoprotein H3 (2H9)	2.3336275	up
			2.0423963	up
ADD2	NM_017482	adducin 2 (beta)	2.0718493	up
SLC14A1	NM_001146037	solute carrier family 14 (urea transporter), member 1 (Kidd blood group)	3.0283325	up
FIG4		FIG4 homolog, SAC1 lipid phosphatase domain containing (S. cerevisiae)	2.0093048	up
TMBIM4	NM_016056	transmembrane BAX inhibitor motif containing 4	2.522177	up
NR1D2	NM_005126	nuclear receptor subfamily 1, group D, member 2	4.0018487	up
LOC147727	NR_024333	uncharacterized LOC147727	2.1628058	up
PRDX3	NM_006793	peroxiredoxin 3	2.8585997	up
EPHB4	NM_004444	EPH receptor B4	2.2087057	up
MS4A6A	NM_152852	membrane-spanning 4-domains, subfamily A, member 6A	2.3323429	up
ACER1	NM_133492	alkaline ceramidase 1	2.0135407	up
RBM12	NM_006047	RNA binding motif protein 12	2.4134982	up
LIPA	NM_000235	lipase A, lysosomal acid, cholesterol esterase	2.1935859	up
NDUFV2	NM_021074	NADH dehydrogenase (ubiquinone) flavoprotein 2, 24kDa	2.0734174	up
MRPL1	NM_020236	mitochondrial ribosomal protein L1	2.054657	up
CA1	NM_001738	carbonic anhydrase I	2.6069458	up
COL8A2	NM_005202	collagen, type VIII, alpha 2	2.1654181	up

MEAF6	NM_022756	MYST/Esa1-associated factor 6	2.2747684	up
STAP1	NM_012108	signal transducing adaptor family member 1	2.0867808	up
ECHDC1	NM_018479	enoyl CoA hydratase domain containing 1	2.2329905	up
ATF1	NM_005171	activating transcription factor 1	2.1539748	up
STK17B	BC052561	serine/threonine kinase 17b	2.6169624	up
WDR26	NM_025160	WD repeat domain 26	2.102758	up
SNAP23	NM_003825	synaptosomal-associated protein, 23kDa	2.47934	up
MAB21L2	NM_006439	mab-21-like 2 (C. elegans)	3.562848	up
ANAPC16	NM_001242546	anaphase promoting complex subunit 16	2.0838695	up
			2.8251603	up
ZDHHC2	NM_016353	zinc finger, DHHC-type containing 2	2.258542	up
TWSG1	NM_020648	twisted gastrulation homolog 1 (Drosophila)	2.0730171	up
			2.439557	up
IFT81	NM_014055	intraflagellar transport 81 homolog (Chlamydomonas)	2.318921	up
C15orf29	NM_024713	chromosome 15 open reading frame 29	2.0358288	up
TUBBP5	NR_027156	tubulin, beta pseudogene 5	3.9768343	up
BRD1	NM_014577	bromodomain containing 1	2.194385	up
LYPLAL1	NM_138794	lysophospholipase-like 1	2.179327	up
SOX13	NM_005686	SRY (sex determining region Y)-box 13	2.096142	up
FLJ31306	NR_029434	uncharacterized LOC379025	2.131765	up
CTDSPL2	NM_016396	CTD (carboxy-terminal domain, RNA polymerase II, polypeptide A) small phosphatase like 2	2.0725737	up
MZT1	NM_001071775	mitotic spindle organizing protein 1	2.2675748	up
OSBPL8	NM_020841	oxysterol binding protein-like 8	2.103212	up
LOC153577	AK054653	uncharacterized LOC153577	2.297152	up
LOC151009	NR_027244	uncharacterized LOC151009	2.0207531	up
DDX21	NM_004728	DEAD (Asp-Glu-Ala-Asp) box polypeptide 21	2.1099486	up
SCAI	NM_173690	suppressor of cancer cell invasion	2.0355842	up
SLFN11	NM_001104587	schlafen family member 11	2.0337663	up
CD69	AK303383	CD69 molecule	2.662265	up
ARMCX2	NM_014782	armadillo repeat containing, X-linked 2	2.0339794	up
C20orf196	NM_152504	chromosome 20 open reading frame 196	2.2117243	up
			2.466904	up
SCOC	NM_032547	short coiled-coil protein	2.552416	up
SLC44A5	NM_152697	solute carrier family 44, member 5	2.0434926	up
SETD7	BC066361	SET domain containing (lysine methyltransferase) 7	2.5649555	up
BMP2K	NM_017593	BMP2 inducible kinase	2.03252	up
ZNF217	NM_006526	zinc finger protein 217	2.3352828	up
PLA2G12A	NM_030821	phospholipase A2, group XIIA	2.042286	up
CFL2	NM_021914	cofilin 2 (muscle)	2.5331726	up
CLEC2D	NM_001004419	C-type lectin domain family 2, member D	2.0176551	up

			2.5991683	up
UBXN4	NM_014607	UBX domain protein 4	2.1819606	up
FAR1	NM_032228	fatty acyl CoA reductase 1	2.0757563	up
SKA2	NM_182620	spindle and kinetochore associated complex subunit 2	2.2772539	up
TBCEL	NM_152715	tubulin folding cofactor E-like	3.0788116	up
			2.1227462	up
PIK3R1	NM_181523	phosphoinositide-3-kinase, regulatory subunit 1 (alpha)	2.1363208	up
IFIT1B	NM_001010987	interferon-induced protein with tetratricopeptide repeats 1B	3.2180007	up
EXOC8	NM_175876	exocyst complex component 8	2.1484337	up
FAM125A	XR_111839	family with sequence similarity 125, member A	2.2122724	up
BMPR2	NM_001204	bone morphogenetic protein receptor, type II (serine/threonine kinase)	2.355234	up
JMY	NM_152405	junction mediating and regulatory protein, p53 cofactor	2.211525	up
BPGM	NM_199186	2,3-bisphosphoglycerate mutase	2.9273324	up
SSX2IP	NM_014021	synovial sarcoma, X breakpoint 2 interacting protein	2.7748337	up
LOC729678	NR_027183	uncharacterized LOC729678	2.121622	up
OBSL1	NM_015311	obscurin-like 1	2.6144726	up
LEKR1	AK094480	leucine, glutamate and lysine rich 1	3.026911	up
PLCL2	NM_015184	phospholipase C-like 2	2.233596	up
OR4D1	NM_012374	olfactory receptor, family 4, subfamily D, member 1	3.1983545	up
AHRR	NM_020731	aryl-hydrocarbon receptor repressor	4.9523463	up
GJA10	NM_032602	gap junction protein, alpha 10, 62kDa	2.4125774	up
MRGPRX2	NM_054030	MAS-related GPR, member X2	3.2090268	up
CPB2	NM_016413	carboxypeptidase B2 (plasma)	3.2224495	up
CCP110	NM_014711	centriolar coiled coil protein 110kDa	2.1312978	up
TMEM202	NM_001080462	transmembrane protein 202	3.7996936	up
NSUN3	NM_022072	NOP2/Sun domain family, member 3	2.3972085	up
SGTB	NM_019072	small glutamine-rich tetratricopeptide repeat (TPR)-containing, beta	2.080579	up
PGM3	NM_015599	phosphoglucomutase 3	2.1007688	up
TMEM63A	NM_014698	transmembrane protein 63A	2.4857924	up
TMEM220	NM_001004313	transmembrane protein 220	3.3820732	up
TSC22D2	NM_014779	TSC22 domain family, member 2	2.316937	up
C3orf55	NM_001243729	chromosome 3 open reading frame 55	2.1544988	up
SCAMP1	NM_004866	secretory carrier membrane protein 1	2.1405213	up
VMA21	NM_001017980	VMA21 vacuolar H+-ATPase homolog (S. cerevisiae)	2.0026705	up
KRTAP19-2	NM_181608	keratin associated protein 19-2	3.180922	up
			3.7708602	up
DPY19L2	NM_173812	dpy-19-like 2 (C. elegans)	2.218427	up
CTSL2	NM_001333	cathepsin L2	2.8008616	up
LOC199897	XM_001718592	uncharacterized LOC199897	2.5137637	up
MOSPD1	NM_019556	motile sperm domain containing 1	2.2056162	up
HNRNPH1	NM_005520	heterogeneous nuclear ribonucleoprotein H1 (H)	2.1390495	up

FBXO24	NM_033506	F-box protein 24	3.42086	up
L3MBTL3	NM_032438	l(3)mbt-like 3 (Drosophila)	2.725612	up
ARHGAP28	NM_001010000	Rho GTPase activating protein 28	3.630918	up
CCT6B	NM_006584	chaperonin containing TCP1, subunit 6B (zeta 2)	2.0370011	up
NCS1	NM_014286	neuronal calcium sensor 1	2.0359647	up
SBF2	NM_030962	SET binding factor 2	2.0668836	up
SHOC2	NM_007373	soc-2 suppressor of clear homolog (C. elegans)	2.1174717	up
CDADC1	NM_030911	cytidine and dCMP deaminase domain containing 1	2.978757	up
GCG	NM_002054	glucagon	2.2334974	up
AKAP2	NM_001004065	A kinase (PRKA) anchor protein 2	2.231729	up
TGM2	NM_198951	transglutaminase 2 (C polypeptide, protein-glutamine-gamma-glutamyltransferase)	2.7073684	up
DNAAF1	NM_178452	dynein, axonemal, assembly factor 1	2.1368246	up
WDR43	NM_015131	WD repeat domain 43	2.169946	up
SLAIN2	NM_020846	SLAIN motif family, member 2	2.322515	up
ARHGAP18	NM_033515	Rho GTPase activating protein 18	2.1493456	up
CBX1	NM_006807	chromobox homolog 1	2.0253344	up
FABP4	NM_001442	fatty acid binding protein 4, adipocyte	2.1605802	up
SLC25A26	NM_173471	solute carrier family 25, member 26	2.3715482	up
AMD1	NM_001634	adenosylmethionine decarboxylase 1	2.2836063	up
