# Supplementary material for: Transcriptional signature associated with early rheumatoid arthritis and healthy individuals at high risk to develop the disease
Source: PLoS One. 2018 Mar 27;13(3):e0194205. doi: 10.1371/journal.pone.0194205 (PMC5870959; doi:10.1371/journal.pone.0194205)
Supplement: S3 Table — (PDF) [file pone.0194205.s003.pdf]

Supplementary Table 3. Down regulated genes in patients with RA vs relatives ACCP-

Gene Symbol	Genbank Accession	Gene Name	Fold Change	Regulation
FGF6	NM_020996	fibroblast growth factor 6	-2.4612348	down
DCDC1	NM_181807	doublecortin domain containing 1	-5.908167	down
RSPH1	NM_080860	radial spoke head 1 homolog (Chlamydomonas)	-2.359272	down
IZUMO2	NM_152358	IZUMO family member 2	-2.1440845	down
			-3.5735257	down
FAM205B	AL080137	transmembrane protein C9orf144B pseudogene	-3.9766746	down
C1orf226	NM_001085375	chromosome 1 open reading frame 226	-2.8179643	down
LOC201617	NR_038221	uncharacterized LOC201617	-2.27644	down
RBMS3	NM_014483	RNA binding motif, single stranded interacting protein 3	-2.1848514	down
C7orf45	NM_145268	chromosome 7 open reading frame 45	-2.457924	down
LOC100128402	AK124574	uncharacterized LOC100128402	-3.337858	down
IPO7	NM_006391	importin 7	-2.0830383	down
ETV2	NM_014209	ets variant 2	-2.1907458	down
FOXF1	NM_001451	forkhead box F1	-2.2418375	down
CCKAR	NM_000730	cholecystokinin A receptor	-2.1643167	down
			-2.3475528	down
NBPF15	NM_001170755	neuroblastoma breakpoint family, member 15	-2.9943056	down
KDELR3	NM_016657	KDEL (Lys-Asp-Glu-Leu) endoplasmic reticulum protein retention receptor 3	-2.1010847	down
CLDND2	NM_152353	claudin domain containing 2	-2.2147286	down
	DR007925		-3.0071445	down
C1S	NM_201442	complement component 1, s subcomponent	-2.2595763	down
CYP27B1	NM_000785	cytochrome P450, family 27, subfamily B, polypeptide 1	-2.2186098	down
RIIAD1	NM_001144956	regulatory subunit of type II PKA R-subunit (RIIa) domain containing 1	-2.486339	down
LARP6	NM_197958	La ribonucleoprotein domain family, member 6	-3.1811519	down
STYXL1	NM_016086	serine/threonine/tyrosine interacting-like 1	-2.2973912	down
GRP	NM_002091	gastrin-releasing peptide	-2.2236316	down
GPR135	NM_022571	G protein-coupled receptor 135	-2.2938108	down
FAM75D3	NM_207416	family with sequence similarity 75, member D3	-2.0161974	down
IDO1	NM_002164	indoleamine 2,3-dioxygenase 1	-2.333675	down
GHRHR	NM_000823	growth hormone releasing hormone receptor	-2.1087446	down
ARHGAP33	NM_052948	Rho GTPase activating protein 33	-2.0564446	down
PGPEP1L	NM_001167902	pyroglutamyl-peptidase I-like	-3.1144521	down
			-2.114345	down
			-2.061469	down
	BX107671		-2.2888033	down
HRASLS5	NM_054108	HRAS-like suppressor family, member 5	-3.3872874	down
DEFB103A	NM_001081551	defensin, beta 103A	-2.1911187	down

PLEKHD1	NM_001161498	pleckstrin homology domain containing, family D (with coiled-coil domains) member 1	-2.6186786	down
LCE2C	NM_178429	late cornified envelope 2C	-2.5052247	down
TAS2R14	NM_023922	taste receptor, type 2, member 14	-2.5433955	down
MYO15B	BC128044	myosin XVB pseudogene	-4.016065	down
ASAP3	NM_017707	ArfGAP with SH3 domain, ankyrin repeat and PH domain 3	-2.8068252	down
LOC442028	NR_037597	uncharacterized LOC442028	-2.8493435	down
			-2.9503536	down
			-4.625366	down
TBX6	NM_004608	T-box 6	-6.1043735	down
HS3ST4	NM_006040	heparan sulfate (glucosamine) 3-O-sulfotransferase 4	-2.0810814	down
NGEF	NM_019850	neuronal guanine nucleotide exchange factor	-4.2346206	down
			-2.5424607	down
GTF2IRD1	NM_005685	GTF2I repeat domain containing 1	-3.1708293	down
SOST	NM_025237	sclerostin	-3.002744	down
FLJ40292	NR_024341	uncharacterized LOC643210	-2.1003883	down
KCNC1	NM_004976	potassium voltage-gated channel, Shaw-related subfamily, member 1	-2.2664573	down
			-3.8360286	down
	X13953		-3.1475484	down
POU3F2	NM_005604	POU class 3 homeobox 2	-2.2795503	down
KRT79	NM_175834	keratin 79	-2.656418	down
SCARF2	NM_153334	scavenger receptor class F, member 2	-2.2257302	down
LOC100133331	NR_028327	uncharacterized LOC100133331	-3.773689	down
			-3.9486558	down
LOC145694	XR_109210	uncharacterized LOC145694	-2.4219522	down
INHBA	NM_002192	inhibin, beta A	-4.1262827	down
TERT	NM_198253	telomerase reverse transcriptase	-3.9636452	down
CCL1	NM_002981	chemokine (C-C motif) ligand 1	-2.4017992	down
RARRES2	NM_002889	retinoic acid receptor responder (tazarotene induced) 2	-4.8614817	down
RPS26	NM_001029	ribosomal protein S26	-2.5013144	down
SRL	NM_001098814	sarcalumenin	-2.1878123	down
LOC729668	NR_003524	golgi autoantigen, golgin subfamily a, 6 pseudogene	-3.2774725	down
UGT2B11	NM_001073	UDP glucuronosyltransferase 2 family, polypeptide B11	-6.346666	down
SSX2	NM_175698	synovial sarcoma, X breakpoint 2	-2.273672	down
RDM1	NM_001034836	RAD52 motif 1	-2.9162483	down
THBS2	NM_003247	thrombospondin 2	-2.9432383	down
WTH3DI	NM_001077637	RAB6C-like	-3.3727872	down
DCST1	NM_152494	DC-STAMP domain containing 1	-2.4203007	down
			-2.901084	down
MUC17	NM_001040105	mucin 17, cell surface associated	-2.5495353	down
			-3.2851312	down

			-7.0911107	down
DPCR1	NM_080870	diffuse panbronchiolitis critical region 1	-5.749843	down
	BC101978		-2.9334037	down
CNIH2	NM_182553	cornichon homolog 2 (Drosophila)	-2.1542048	down
			-2.005743	down
SLC35G5	NM_054028	solute carrier family 35, member G5	-3.3485498	down
	XR_108874		-2.557444	down
	AK025047		-2.2335503	down
LOC285484	NR_037863	uncharacterized LOC285484	-2.2150912	down
			-2.0048096	down
MYPN	NM_032578	myopalladin	-2.060425	down
	BC031250		-4.2014008	down
	DA144211		-2.0575192	down
PLEC	NM_201380	plectin	-2.3104613	down
CALCR	NM_001164737	calcitonin receptor	-3.1915514	down
KANK1	AL832454	KN motif and ankyrin repeat domains 1	-2.398038	down
PTCH2		patched 2	-4.3255506	down
MAST4	AK299538	microtubule associated serine/threonine kinase family member 4	-7.23512	down
RLN3	NM_080864	relaxin 3	-2.0871866	down
ULBP3	NM_024518	UL16 binding protein 3	-2.414395	down
LOC100132774	NR_033827	uncharacterized LOC100132774	-3.0804498	down
GABRB1	NM_000812	gamma-aminobutyric acid (GABA) A receptor, beta 1	-2.02682	down
DLG4	NM_001365	discs, large homolog 4 (Drosophila)	-2.8535936	down
KRT33B	NM_002279	keratin 33B	-3.0449245	down
			-2.5631251	down
			-3.190821	down
	XM_002344257		-4.031147	down
FLJ25758	NR_024372	uncharacterized locus FLJ25758	-2.172661	down
CEACAM16	NM_001039213	carcinoembryonic antigen-related cell adhesion molecule 16	-2.5336459	down
LOC100130930	AK126579	uncharacterized LOC100130930	-2.7929077	down
CYP4F2	NM_001082	cytochrome P450, family 4, subfamily F, polypeptide 2	-3.4355886	down
ZNF746	NM_152557	zinc finger protein 746	-2.468108	down
FLJ39653	BC132927	uncharacterized FLJ39653	-2.1538723	down
GOLGA8F	NR_033351	golgin A8 family, member F	-3.772159	down
IQSEC3	BC024764	IQ motif and Sec7 domain 3	-2.3883328	down
	AK131313		-2.7875314	down
SCARA5	AY337579	scavenger receptor class A, member 5 (putative)	-2.2809887	down
TMEM146	NM_152784	transmembrane protein 146	-2.8033879	down
GRIK3	NM_000831	glutamate receptor, ionotropic, kainate 3	-2.8780453	down
EMR4P	NR_024075	egf-like module containing, mucin-like, hormone receptor-like 4 pseudogene	-3.8318007	down

	AK128867		-2.339463	down
LOC399875	BC040220	uncharacterized LOC399875	-3.5178869	down
DKK4	NM_014420	dickkopf homolog 4 (Xenopus laevis)	-2.437393	down
CSMD2	NM_052896	CUB and Sushi multiple domains 2	-2.3411067	down
LOC100128348	AK128128	uncharacterized LOC100128348	-3.5356994	down
			-2.6931462	down
P2RX2	NM_170683	purinergic receptor P2X, ligand-gated ion channel, 2	-2.557772	down
			-2.678121	down
LOC100128869	XM_001719518	uncharacterized LOC100128869	-3.5131736	down
KCTD11	NM_001002914	potassium channel tetramerisation domain containing 11	-2.2699606	down
	AF072164		-4.9391103	down
TCHHL1	NM_001008536	trichohyalin-like 1	-2.0612605	down
LOC728503	XM_001127575	uncharacterized LOC728503	-4.228413	down
GATA4	NM_002052	GATA binding protein 4	-2.033399	down
LOC100133131	AF289593	uncharacterized LOC100133131	-3.0451298	down
LOC729159	XM_001129515	UPF0607 protein ENSP00000381418-like	-2.8732407	down
			-2.1575031	down
OTOP1	NM_177998	otopetrin 1	-2.1152172	down
OR2C1	NM_012368	olfactory receptor, family 2, subfamily C, member 1	-2.673744	down
			-4.1162167	down
LOC389834	NR_027420	ankyrin repeat domain 57 pseudogene	-2.426158	down
CDX2	NM_001265	caudal type homeobox 2	-2.2728572	down
DNM1P46	NR_003260	DNM1 pseudogene 46	-2.2783635	down
GPAT2	NM_207328	glycerol-3-phosphate acyltransferase 2, mitochondrial	-2.6531255	down
HABP2	NM_004132	hyaluronan binding protein 2	-2.9742222	down
LOC100130093	NR_024485	uncharacterized LOC100130093	-2.198175	down
LOC100129393	AK124631	uncharacterized LOC100129393	-3.2786276	down
DNAH17	NM_173628	dynein, axonemal, heavy chain 17	-2.5355463	down
	AF289615		-2.060981	down
FAM106CP	NR_026810	family with sequence similarity 106, member C pseudogene	-2.1905699	down
DNPEP	NM_012100	aspartyl aminopeptidase	-2.3839345	down
			-2.4030042	down
LOC283728	AK095617	uncharacterized LOC283728	-3.7149692	down
TRIM36	BC017346	tripartite motif containing 36	-5.316765	down
DDX3Y	NM_001122665	DEAD (Asp-Glu-Ala-Asp) box polypeptide 3, Y-linked	-2.8573813	down
LOC729305	XR_132809	uncharacterized LOC729305	-5.7991824	down
FOXI2	NM_207426	forkhead box I2	-2.0110466	down
C13orf33	NM_032849	chromosome 13 open reading frame 33	-2.6747673	down
LOC100132247	NM_001135865	nuclear pore complex interacting protein related gene	-2.2041225	down
			-2.0018158	down
FREM2	NM_207361	FRAS1 related extracellular matrix protein 2	-2.993317	down

CHRM5	NM_012125	cholinergic receptor, muscarinic 5	-2.0895228	down
	AK124041		-2.1995711	down
LOC389332	NR_024418	uncharacterized LOC389332	-2.1874394	down
RNU105A	NR_004404	RNA, U105A small nucleolar	-2.374022	down
	XM_001717980		-3.5069368	down
GINS2	NM_016095	GINS complex subunit 2 (Psf2 homolog)	-8.626691	down
TPD52L1	NM_001003395	tumor protein D52-like 1	-2.068876	down
			-2.7930276	down
SERPINB3	NM_006919	serpin peptidase inhibitor, clade B (ovalbumin), member 3	-2.4826992	down
GABRQ	NM_018558	gamma-aminobutyric acid (GABA) receptor, theta	-2.6876967	down
RASSF4		Ras association (RalGDS/AF-6) domain family member 4	-2.224959	down
DOC2B	NM_003585	double C2-like domains, beta	-2.4832351	down
	AK025312		-2.0665631	down
HRASLS2	NM_017878	HRAS-like suppressor 2	-2.728009	down
IL36G	NM_019618	interleukin 36, gamma	-2.6372962	down
AKAP4	NM_003886	A kinase (PRKA) anchor protein 4	-2.4355173	down
PRR7	AK125417	proline rich 7 (synaptic)	-4.424351	down
LOC728671	XM_001128100	uncharacterized LOC728671	-5.382475	down
LOC642852	NR_026943	uncharacterized LOC642852	-3.6159494	down
PCDHB11	NM_018931	protocadherin beta 11	-2.79214	down
C5orf64	NM_173667	chromosome 5 open reading frame 64	-4.768939	down
LINGO2	NM_152570	leucine rich repeat and Ig domain containing 2	-4.4788895	down
NUDT8	NM_001243750	nudix (nucleoside diphosphate linked moiety X)-type motif 8	-3.5824828	down
			-7.947017	down
AOC4	NR_002773	AOC3 pseudogene	-4.0430007	down
RNU4ATAC	DW419002	RNA, U4atac small nuclear (U12-dependent splicing)	-2.3195674	down
MCART1	NR_024872	mitochondrial carrier triple repeat 1	-2.30282	down
LOC100128657	XR_132562	coiled-coil-helix-coiled-coil-helix domain containing 4 pseudogene	-2.5478723	down
			-2.5708065	down
	Z18843		-2.689854	down
LOC729966	NR_036575	uncharacterized LOC729966	-2.4443069	down
LOC100132686	BC020894	uncharacterized LOC100132686	-2.4803283	down
NR2E3	NM_014249	nuclear receptor subfamily 2, group E, member 3	-2.0687454	down
LRRC37BP1	AK125981	leucine rich repeat containing 37B pseudogene 1	-2.5252504	down
ATP8B3	NM_138813	ATPase, aminophospholipid transporter, class I, type 8B, member 3	-2.2276285	down
	AK057820		-3.4689136	down
ZNF562	NM_017656	zinc finger protein 562	-2.0377355	down
POMZP3	NM_152992	POM121 and ZP3 fusion	-2.5042193	down
GGA1		golgi-associated, gamma adaptin ear containing, ARF binding protein 1	-2.6645577	down
CYP3A4	NM_017460	cytochrome P450, family 3, subfamily A, polypeptide 4	-2.4023285	down
LAMB1	BC044633	laminin, beta 1	-2.4714444	down

GPR35	NM_001195381	G protein-coupled receptor 35	-2.187888	down
COPE	AK023303	coatomer protein complex, subunit epsilon	-2.519379	down
MLLT4	NM_001207008	myeloid/lymphoid or mixed-lineage leukemia (trithorax homolog, Drosophila); translocated to, 4	-3.0376167	down
LOC144817	NR_038433	uncharacterized LOC144817	-2.9228003	down
SERTM1	NM_203451	serine-rich and transmembrane domain containing 1	-3.9387424	down
IGFL3	NM_207393	IGF-like family member 3	-4.200748	down
C19orf55	NM_001039887	chromosome 19 open reading frame 55	-2.3949616	down
	BM477328		-2.3815846	down
BMP15	NM_005448	bone morphogenetic protein 15	-2.9643145	down
ZNF705A	NM_001004328	zinc finger protein 705A	-2.8314395	down
	AK024188		-2.5607195	down
	BX538057		-2.7868655	down
SFRP1	NM_003012	secreted frizzled-related protein 1	-3.3551188	down
PCDHGB7	NM_032101	protocadherin gamma subfamily B, 7	-3.278794	down
SH2D4A	NM_022071	SH2 domain containing 4A	-2.4754755	down
LOC572558	NR_015423	uncharacterized LOC572558	-2.2824948	down
LZTS2	AK097997	leucine zipper, putative tumor suppressor 2	-2.420541	down
			-3.2616613	down
LOC729995	XR_110198	hCG1817208	-2.5670633	down
LOC100129316	NR_033912	uncharacterized LOC100129316	-2.3271677	down
LOC100506533	XM_003118505	putative uncharacterized protein FLJ35883-like	-2.8542833	down
LOC389043	NR_036499	uncharacterized LOC389043	-2.151608	down
PLAGL2	NM_002657	pleiomorphic adenoma gene-like 2	-2.3623219	down
PPP1R26	NM_014811	protein phosphatase 1, regulatory subunit 26	-2.507344	down
MICU1		mitochondrial calcium uptake 1	-2.1282022	down
	DA734158		-3.0927353	down
NRSN2	NM_024958	neurensin 2	-2.9623015	down
PMFBP1	NM_031293	polyamine modulated factor 1 binding protein 1	-2.312073	down
			-2.4092784	down
C20orf151	NM_080833	chromosome 20 open reading frame 151	-2.3318949	down
KIFC2	NM_145754	kinesin family member C2	-3.0426295	down
NANOS2	NM_001029861	nanos homolog 2 (Drosophila)	-2.1999478	down
LAMC3	NM_006059	laminin, gamma 3	-4.0606203	down
LOC145837	NR_026979	uncharacterized LOC145837	-3.6114953	down
BARX2	NM_003658	BARX homeobox 2	-2.2483613	down
KYNU		kynureninase	-2.0048492	down
B3GNT8	NM_198540	UDP-GlcNAc:betaGal beta-1,3-N-acetylglucosaminyltransferase 8	-2.0302181	down
LOC254057	AK024653	uncharacterized LOC254057	-2.1027448	down
			-3.454256	down
LOC100128333	AK127681	uncharacterized LOC100128333	-2.4480226	down

INCENP	NM_001040694	inner centromere protein antigens 135/155kDa	-2.3008037	down
MAGEB1	NM_002363	melanoma antigen family B, 1	-2.6537116	down
RXFP2	NM_130806	relaxin/insulin-like family peptide receptor 2	-2.364773	down
COL13A1	NM_080801	collagen, type XIII, alpha 1	-2.4663982	down
LOC100190940	NR_024457	uncharacterized LOC100190940	-2.2174277	down
			-4.6378016	down
NODAL	NM_018055	nodal homolog (mouse)	-3.1945043	down
			-2.900224	down
	XM_003118552		-6.7279897	down
CYP7A1	NM_000780	cytochrome P450, family 7, subfamily A, polypeptide 1	-2.5759485	down
C3orf35	NM_178339	chromosome 3 open reading frame 35	-2.1795719	down
			-2.8362055	down
	AK098235		-2.2333887	down
ZNF280A	NM_080740	zinc finger protein 280A	-2.409288	down
GSG1	NM_001080554	germ cell associated 1	-5.013273	down
	AF090909		-3.1378796	down
CYTL1	NM_018659	cytokine-like 1	-2.2124548	down
C15orf60	NM_001042367	chromosome 15 open reading frame 60	-2.0673096	down
	CR989582		-2.0196733	down
LOC392364	NR_040117	chromosome 15 open reading frame 2 pseudogene	-2.0676107	down
IL36A	NM_014440	interleukin 36, alpha	-2.8281875	down
LOC389834	NR_027420	ankyrin repeat domain 57 pseudogene	-2.187541	down
AOX1	NM_001159	aldehyde oxidase 1	-2.6017566	down
			-2.4459352	down
MLL5	NM_182931	myeloid/lymphoid or mixed-lineage leukemia 5 (trithorax homolog, Drosophila)	-2.8510187	down
AVPR2	NM_000054	arginine vasopressin receptor 2	-2.8083498	down
C11orf52	NM_080659	chromosome 11 open reading frame 52	-3.7092638	down
			-2.9572113	down
LINC00467	NR_026761	long intergenic non-protein coding RNA 467	-3.2555642	down
			-2.092665	down
	AK024162		-5.4059706	down
SRRM3	NM_001110199	serine/arginine repetitive matrix 3	-3.6287715	down
ELK4	NM_021795	ELK4, ETS-domain protein (SRF accessory protein 1)	-2.4673357	down
GGT8P	NR_003503	gamma-glutamyltransferase 8 pseudogene	-4.8570437	down
GRIA3	NM_000828	glutamate receptor, ionotrophic, AMPA 3	-3.609577	down
LOC80054	NR_026887	uncharacterized LOC80054	-2.1593254	down
S100A14	NM_020672	S100 calcium binding protein A14	-4.210981	down
RAPGEF3	U78169	Rap guanine nucleotide exchange factor (GEF) 3	-2.2429178	down
LRP2	NM_004525	low density lipoprotein receptor-related protein 2	-10.938198	down
OR52I2	NM_001005170	olfactory receptor, family 52, subfamily I, member 2	-2.2155972	down

			-2.5089257	down
			-4.0312285	down
COL27A1	BC007696	collagen, type XXVII, alpha 1	-2.0218265	down
LOC339316	BC043286	uncharacterized LOC339316	-2.3005366	down
LRPPRC	NM_133259	leucine-rich PPR-motif containing	-3.1235523	down
FOXQ1	NM_033260	forkhead box Q1	-3.5405126	down
KCNA7	NM_031886	potassium voltage-gated channel, shaker-related subfamily, member 7	-2.1563544	down
SLC24A5	NM_205850	solute carrier family 24, member 5	-5.512019	down
ACTN4	NM_004924	actinin, alpha 4	-2.0808754	down
			-3.6623635	down
LOC100131107	XM_001715471	UPF0607 protein ENSP00000383783-like	-2.9852264	down
			-2.290563	down
	DR007930		-3.9309983	down
CACNA1B		calcium channel, voltage-dependent, N type, alpha 1B subunit	-2.0461526	down
PRSS56	NM_001195129	protease, serine, 56	-2.0246875	down
	BC040680		-2.9862437	down
GCNT4	NM_016591	glucosaminyl (N-acetyl) transferase 4, core 2	-2.5956194	down
			-2.8009536	down
MDH1B	NM_001039845	malate dehydrogenase 1B, NAD (soluble)	-2.4499521	down
GNA11	NM_002067	guanine nucleotide binding protein (G protein), alpha 11 (Gq class)	-2.5387928	down
LOC151171	NR_037809	uncharacterized LOC151171	-2.4732323	down
KCP	NM_199349	kielin/chordin-like protein	-2.190662	down
HCN3	NM_020897	hyperpolarization activated cyclic nucleotide-gated potassium channel 3	-2.6795626	down
NXF5	NM_032946	nuclear RNA export factor 5	-2.1164608	down
	AK126351		-3.5204198	down
			-3.6131644	down
ATP10D	NM_020453	ATPase, class V, type 10D	-2.5520403	down
MAGEA2B	NM_153488	melanoma antigen family A, 2B	-3.4460616	down
CCDC60	NM_178499	coiled-coil domain containing 60	-3.1452763	down
C8orf71	NR_026772	chromosome 8 open reading frame 71	-3.3366601	down
PTK6	NM_005975	PTK6 protein tyrosine kinase 6	-4.342905	down
FAM71A	NM_153606	family with sequence similarity 71, member A	-2.6628368	down
RASSF6	NM_201431	Ras association (RalGDS/AF-6) domain family member 6	-3.694323	down
AGAP1	NM_001037131	ArfGAP with GTPase domain, ankyrin repeat and PH domain 1	-3.9957428	down
			-2.6536207	down
TPD52	XM_001716081	tumor protein D52	-2.0229888	down
ARHGAP44	NM_014859	Rho GTPase activating protein 44	-2.3134844	down
			-2.7637691	down
LOC400743	AK127830	uncharacterized LOC400743	-3.1892774	down
TSPAN14	NM_030927	tetraspanin 14	-2.2708437	down
GAGE7	NM_021123	G antigen 7	-4.559126	down

TTC30A	NM_152275	tetratricopeptide repeat domain 30A	-2.0577908	down
			-2.0418484	down
			-3.1730504	down
			-6.8913774	down
LAT2	BC006080	linker for activation of T cells family, member 2	-3.0589664	down
			-3.1212816	down
LOC100134040	AL162052	uncharacterized LOC100134040	-3.6147633	down
LOC647070	AK001442	uncharacterized LOC647070	-2.4439862	down
AFF3		AF4/FMR2 family, member 3	-2.695566	down
C1orf51	NM_144697	chromosome 1 open reading frame 51	-2.4202318	down
CSRP2	NM_001321	cysteine and glycine-rich protein 2	-22.980167	down
MDM4	NM_001204171	Mdm4 p53 binding protein homolog (mouse)	-3.1185024	down
SLC38A1	DC378344	solute carrier family 38, member 1	-2.378194	down
S100A7	NM_002963	S100 calcium binding protein A7	-2.3794377	down
			-4.3220015	down
	AK097471		-3.5886817	down
OR51G1	NM_001005237	olfactory receptor, family 51, subfamily G, member 1	-2.8088446	down
LOC100128811	NR_027333	uncharacterized LOC100128811	-3.0326595	down
GOLGA6L5	NR_003246	golgin A6 family-like 5 (pseudogene)	-4.1409206	down
MAEL	NM_032858	maelstrom homolog (Drosophila)	-2.2612245	down
S100A1	NM_006271	S100 calcium binding protein A1	-2.3622494	down
LOC728339	BC087857	uncharacterized LOC728339	-2.338064	down
			-2.6913917	down
SOBP	NM_018013	sine oculis binding protein homolog (Drosophila)	-2.1060286	down
SH2D6	BC110073	SH2 domain containing 6	-2.6259198	down
ST8SIA1	NM_003034	ST8 alpha-N-acetyl-neuraminide alpha-2,8-sialyltransferase 1	-4.096914	down
RPL23AP64	NR_003040	ribosomal protein L23a pseudogene 64	-2.6580968	down
PRB4	NM_002723	proline-rich protein BstNI subfamily 4	-2.559516	down
DEPDC5	NM_001007188	DEP domain containing 5	-2.033664	down
LOC158960	CR613749	uncharacterized protein BC009467	-2.4126246	down
ANKRD13B	NM_152345	ankyrin repeat domain 13B	-5.5920157	down
TEX13A	NM_031274	testis expressed 13A	-2.1771133	down
WDR87	NM_031951	WD repeat domain 87	-6.350717	down
TPTE2P3	NR_002793	transmembrane phosphoinositide 3-phosphatase and tensin homolog 2 pseudogene 3	-2.8170989	down
			-3.350116	down
COL5A3	NM_015719	collagen, type V, alpha 3	-2.3167112	down
TULP1	NM_003322	tubby like protein 1	-2.0884333	down
TRPC2	NR_002720	transient receptor potential cation channel, subfamily C, member 2, pseudogene	-2.4366245	down
KRTAP4-2	NM_033062	keratin associated protein 4-2	-2.0248802	down
FTCD	NM_206965	formiminotransferase cyclodeaminase	-2.5420594	down

LINC00371	XR_132731	long intergenic non-protein coding RNA 371	-2.6738272	down
GAS2L2	NM_139285	growth arrest-specific 2 like 2	-2.6415405	down
			-2.568622	down
ERF	NM_006494	Ets2 repressor factor	-2.1893613	down
OR5F1	NM_003697	olfactory receptor, family 5, subfamily F, member 1	-2.3779263	down
NEK10	AK057247	NIMA (never in mitosis gene a)- related kinase 10	-2.1835463	down
			-2.1255703	down
DUOXA1	EU927394	dual oxidase maturation factor 1	-3.0396867	down
USP17L2	NM_201402	ubiquitin specific peptidase 17-like 2	-2.5991228	down
LOC100131581	AK092544	uncharacterized LOC100131581	-8.233938	down
RAX	NM_013435	retina and anterior neural fold homeobox	-2.3905017	down
CSTL1	NM_138283	cystatin-like 1	-2.3075707	down
HECTD2	NM_173497	HECT domain containing 2	-2.4544797	down
VSTM2A	NM_182546	V-set and transmembrane domain containing 2A	-2.1677985	down
COG4	NM_001195139	component of oligomeric golgi complex 4	-3.183384	down
ABCD3	NM_001122674	ATP-binding cassette, sub-family D (ALD), member 3	-2.609972	down
GPR61	NM_031936	G protein-coupled receptor 61	-2.4386528	down
ZNF467	NM_207336	zinc finger protein 467	-2.057597	down
LAMA5	BC015386	laminin, alpha 5	-2.5294468	down
			-2.0559359	down
DISC1	NM_001164550	disrupted in schizophrenia 1	-4.0900855	down
LOC100130542	AK127083	uncharacterized LOC100130542	-2.8147304	down
KIAA1755	NM_001029864	KIAA1755	-2.2032206	down
	AK095621		-3.285275	down
			-2.8335786	down
LOC100132249	XR_132823	uncharacterized LOC100132249	-2.9292002	down
			-6.4335713	down
LOC284551	NR_027085	uncharacterized LOC284551	-2.3071935	down
C6orf10	NM_006781	chromosome 6 open reading frame 10	-2.1796014	down
			-4.9856873	down
TUBB8	NM_177987	tubulin, beta 8 class VIII	-3.1990798	down
KIR2DS4	NM_012314	killer cell immunoglobulin-like receptor, two domains, short cytoplasmic tail, 4	-2.028281	down
SLC9A3R2	NM_004785	solute carrier family 9 (sodium/hydrogen exchanger), member 3 regulator 2	-2.225512	down
MAP3K4	NM_006724	mitogen-activated protein kinase kinase kinase 4	-2.7849026	down
			-2.9401739	down
TTTY13	NR_001537	testis-specific transcript, Y-linked 13 (non-protein coding)	-2.388504	down
OR51I2	NM_001004754	olfactory receptor, family 51, subfamily I, member 2	-2.84366	down
CERCAM	NM_016174	cerebral endothelial cell adhesion molecule	-7.717456	down
	XR_112969		-2.3091383	down
FAM5C	NM_199051	family with sequence similarity 5, member C	-4.288718	down

KLHL4	NM_057162	kelch-like 4 (Drosophila)	-2.8599212	down
			-4.3714232	down
			-2.9069355	down
C1orf52	NM_198077	chromosome 1 open reading frame 52	-2.9682403	down
KCNQ2	AK293727	potassium voltage-gated channel, KQT-like subfamily, member 2	-2.6751084	down
	AB305786		-2.3179505	down
KIAA1161	NM_020702	KIAA1161	-2.6965714	down
OR9G9	NM_001013358	olfactory receptor, family 9, subfamily G, member 9	-2.3524234	down
GDAP1L1	NM_024034	ganglioside-induced differentiation-associated protein 1-like 1	-2.310358	down
SCARNA5	NR_003008	small Cajal body-specific RNA 5	-3.9698277	down
KRT6A	NM_005554	keratin 6A	-2.9003875	down
			-3.1290665	down
ZNF526	NM_133444	zinc finger protein 526	-2.008108	down
TMEM201	NM_001130924	transmembrane protein 201	-2.8604376	down
XAGE-4	XR_113307	XAGE-4 protein	-2.8020458	down
			-3.4956048	down
OR4F21	NM_001005504	olfactory receptor, family 4, subfamily F, member 21	-2.3006544	down
			-2.1157777	down
			-2.303116	down
			-2.5150135	down
BARHL2	NM_020063	BarH-like homeobox 2	-2.1635277	down
PCDHB2	NM_018936	protocadherin beta 2	-2.6112478	down
USP21	NM_001014443	ubiquitin specific peptidase 21	-2.2606707	down
UPB1	NM_016327	ureidopropionase, beta	-2.5708623	down
			-2.3648338	down
HMGA2	NM_003484	high mobility group AT-hook 2	-2.9009023	down
	CR936830		-2.0233212	down
HNF1B	NM_000458	HNF1 homeobox B	-2.5154667	down
GSG1	NM_031289	germ cell associated 1	-2.3850048	down
			-3.1702468	down
CALY		calcyon neuron-specific vesicular protein	-2.565098	down
	AK074492		-2.8202822	down
SLC16A13	NM_201566	solute carrier family 16, member 13 (monocarboxylic acid transporter 13)	-2.192794	down
EPHB4	NM_004444	EPH receptor B4	-2.2023368	down
THBS3	CR933610	thrombospondin 3	-2.482915	down
LOC389199	XR_112616	uncharacterized LOC389199	-5.2141843	down
ACCN3	NM_020322	amiloride-sensitive cation channel 3	-2.2609878	down
LOC285768	NR_027115	uncharacterized LOC285768	-2.4689662	down
FLJ22184	NM_001190467	putative uncharacterized protein FLJ22184	-2.1675034	down
	AK090448		-4.2891116	down

GOLGA2		golgin A2	-2.2291768	down
COL6A2	NM_058175	collagen, type VI, alpha 2	-2.7809207	down
			-2.6206617	down
	AK123308		-2.0549405	down
SORCS3	NM_014978	sortilin-related VPS10 domain containing receptor 3	-2.2625854	down
LOC100130051	AK127296	uncharacterized LOC100130051	-2.4956615	down
NPIP	NM_006985	nuclear pore complex interacting protein	-2.167525	down
OR2T5	NM_001004697	olfactory receptor, family 2, subfamily T, member 5	-2.1197217	down
			-2.175076	down
LOC157273	NR_040039	uncharacterized LOC157273	-2.7618835	down
	AK126938		-2.921644	down
NNMT	NM_006169	nicotinamide N-methyltransferase	-13.12961	down
			-2.3312879	down
LINC00310	NR_027267	long intergenic non-protein coding RNA 310	-2.173869	down
LPHN1	NM_001008701	latrophilin 1	-2.3900933	down
ATPAF1-AS1	NM_001145474	ATPAF1 antisense RNA 1 (non-protein coding)	-4.49347	down
			-3.5526977	down
DEFB134	NM_001033019	defensin, beta 134	-2.3584015	down
RETSAT		retinol saturase (all-trans-retinol 13,14-reductase)	-2.0712445	down
CYP4Z1	NM_178134	cytochrome P450, family 4, subfamily Z, polypeptide 1	-2.041899	down
C22orf34	NR_026997	chromosome 22 open reading frame 34	-3.4166331	down
FAM163B	NM_001080515	family with sequence similarity 163, member B	-4.526807	down
CDK18	NM_212503	cyclin-dependent kinase 18	-2.4427114	down
			-2.2805216	down
DEFB107A	NM_001037668	defensin, beta 107A	-6.540771	down
EMP2	NM_001424	epithelial membrane protein 2	-3.9792604	down
KRTAP10-6	NM_198688	keratin associated protein 10-6	-2.2442586	down
			-2.4281075	down
UFSP1	NM_001015072	UFM1-specific peptidase 1 (non-functional)	-2.1328275	down
KLK13	NM_015596	kallikrein-related peptidase 13	-2.421634	down
MOG	NM_206809	myelin oligodendrocyte glycoprotein	-2.8477802	down
			-2.42982	down
FOXP3	NM_014009	forkhead box P3	-2.1542158	down
IQCF2	NM_203424	IQ motif containing F2	-3.2830222	down
PCDHGB1	NM_032095	protocadherin gamma subfamily B, 1	-2.044309	down
LOC100131742	AK130172	uncharacterized LOC100131742	-2.585999	down
			-3.7484388	down
POTEM	NM_001145442	POTE ankyrin domain family, member M	-7.468435	down
FOXA3	NM_004497	forkhead box A3	-2.5469534	down
LINC00271	NR_026805	long intergenic non-protein coding RNA 271	-2.036642	down
			-3.0407228	down

			-2.9879882	down
MYBPC1	NM_206819	myosin binding protein C, slow type	-2.3334785	down
FLJ45482	AK127393	uncharacterized LOC645566	-2.0237968	down
RAD21-AS1	NR_033886	RAD21 antisense RNA 1 (non-protein coding)	-2.5131156	down
			-3.9413996	down
ZCCHC23	AK127166	zinc finger, CCHC domain containing 23	-5.029593	down
C10orf58	NM_001243780	chromosome 10 open reading frame 58	-2.2744062	down
CBX1	NM_006807	chromobox homolog 1	-2.1077194	down
NT5DC4	BC041437	5'-nucleotidase domain containing 4	-2.1735709	down
CCNT1	NM_001240	cyclin T1	-2.7882442	down
KLK8	NM_144505	kallikrein-related peptidase 8	-3.1755412	down
			-2.2669382	down
			-3.81752	down
MAGIX	DQ884401	MAGI family member, X-linked	-3.233026	down
			-4.2233114	down
EDA	NM_001005610	ectodysplasin A	-2.1785462	down
NAT8	NM_003960	N-acetyltransferase 8 (GCN5-related, putative)	-2.8716145	down
LOC442421	NR_024496	uncharacterized LOC442421	-2.1895514	down
SERPINI2	NM_006217	serpin peptidase inhibitor, clade I (pancpin), member 2	-2.0928302	down
AGPHD1	NM_001083612	aminoglycoside phosphotransferase domain containing 1	-3.9076874	down
LOC728723	NR_024398	uncharacterized LOC728723	-3.2932596	down
OR13A1	NM_001004297	olfactory receptor, family 13, subfamily A, member 1	-2.099428	down
ADAMTS15	NM_139055	ADAM metallopeptidase with thrombospondin type 1 motif, 15	-2.1439404	down
ZNF813	NM_001004301	zinc finger protein 813	-2.342992	down
ADARB1	NR_027673	adenosine deaminase, RNA-specific, B1	-2.1330729	down
LOC100233156	AK126241	tektin 4 pseudogene	-3.8529954	down
PPP2R3B-AS1	NR_027232	PPP2R3B antisense RNA 1 (non-protein coding)	-2.9998403	down
	BC040680		-2.1774023	down
LOC154822	NR_024394	uncharacterized LOC154822	-2.2514052	down
PRKD1	NM_002742	protein kinase D1	-3.865807	down
RAX2	NM_032753	retina and anterior neural fold homeobox 2	-4.128754	down
ZNF169	NM_194320	zinc finger protein 169	-2.108995	down
A1CF	NM_138933	APOBEC1 complementation factor	-3.7475994	down
GIPC3	NM_133261	GIPC PDZ domain containing family, member 3	-2.9935844	down
LOC642620	AK057320	hCG1999814	-3.0802457	down
			-2.9055536	down
SLCO5A1	NM_030958	solute carrier organic anion transporter family, member 5A1	-2.3006828	down
TAF5L	NM_014409	TAF5-like RNA polymerase II, p300/CBP-associated factor (PCAF)-associated factor, 65kDa	-3.531544	down
GPC1	NM_002081	glypican 1	-2.2077737	down
HHIPL1	NM_032425	HHIP-like 1	-2.093702	down

HECTD2	NM_182765	HECT domain containing 2	-4.0942264	down
			-2.8668354	down
CSHL1	NM_022579	chorionic somatomammotropin hormone-like 1	-2.6213083	down
BAI2	NM_001703	brain-specific angiogenesis inhibitor 2	-2.0643744	down
FAM106A	NR_026809	family with sequence similarity 106, member A	-3.5205219	down
			-2.9638157	down
	AK127893		-4.8544517	down
VTCN1	BC065717	V-set domain containing T cell activation inhibitor 1	-2.031538	down
LOC100132529	XR_109319	uncharacterized LOC100132529	-2.9600005	down
LOC100127904	BC065739	uncharacterized LOC100127904	-5.4386525	down
LOC440131	NR_033889	uncharacterized LOC440131	-2.5443115	down
	AF218021		-2.8649302	down
LOC728903	AK093722	uncharacterized LOC728903	-2.5341508	down
ZDHHC23	AK127260	zinc finger, DHHC-type containing 23	-6.0998616	down
FAM107B	NM_031453	family with sequence similarity 107, member B	-2.022441	down
MZT2A	BC017694	mitotic spindle organizing protein 2A	-3.1391382	down
TMEM151B	NM_001137560	transmembrane protein 151B	-2.982902	down
TNFRSF6B	NM_003823	tumor necrosis factor receptor superfamily, member 6b, decoy	-3.1570582	down
	XR_108937		-2.4181159	down
	AK300203		-2.6573431	down
EXOC3L2	NM_138568	exocyst complex component 3-like 2	-2.0027194	down
PRSS16	NM_005865	protease, serine, 16 (thymus)	-11.002375	down
ABCG4	NM_022169	ATP-binding cassette, sub-family G (WHITE), member 4	-2.0086153	down
LOC100233209	NR_026544	uncharacterized LOC100233209	-2.3936365	down
OR7E5P	NR_027688	olfactory receptor, family 7, subfamily E, member 5 pseudogene	-3.4216955	down
	BC042181		-3.1926172	down
			-3.2652285	down
			-2.2247903	down
VSIG10L	NM_001163922	V-set and immunoglobulin domain containing 10 like	-2.73466	down
FAM57B	NM_031478	family with sequence similarity 57, member B	-2.1494594	down
PHYHIP	NM_014759	phytanoyl-CoA 2-hydroxylase interacting protein	-3.9368682	down
FLJ42022	AK124016	uncharacterized LOC646748	-2.330798	down
LOC100507410	NR_040018	uncharacterized LOC100507410	-2.4516547	down
GOLGA6L9	NM_198181	golgin A6 family-like 9	-2.619375	down
NACAD	NM_001146334	NAC alpha domain containing	-2.9412565	down
POTED	NM_174981	POTE ankyrin domain family, member D	-3.989508	down
	DQ786249		-3.532068	down
ZNF471	NM_020813	zinc finger protein 471	-2.1810136	down
MTSS1L	NM_138383	metastasis suppressor 1-like	-3.9568074	down
ZNF169	NM_194320	zinc finger protein 169	-2.2940533	down
LOC100130894	NR_034083	uncharacterized LOC100130894	-2.3949747	down

LGALS14	NM_203471	lectin, galactoside-binding, soluble, 14	-2.9478471	down
KRT34	NM_021013	keratin 34	-3.3958266	down
TLE6	NM_001143986	transducin-like enhancer of split 6 (E(sp1) homolog, Drosophila)	-3.1661563	down
DNAH7	AK094515	dynein, axonemal, heavy chain 7	-2.0253835	down
			-4.0448604	down
ENGASE	NM_001042573	endo-beta-N-acetylglucosaminidase	-3.5386775	down
PLA2G6	NM_001004426	phospholipase A2, group VI (cytosolic, calcium-independent)	-2.614979	down
	DB063160		-2.2317536	down
HSPB3	NM_006308	heat shock 27kDa protein 3	-2.325979	down
PGM5P2	NR_002836	phosphoglucomutase 5 pseudogene 2	-2.2196925	down
FLJ43879	NR_034162	FLJ43879 protein	-2.1957731	down
RIPK4	NM_020639	receptor-interacting serine-threonine kinase 4	-2.3816807	down
MMP21	NM_147191	matrix metallopeptidase 21	-2.1670947	down
NOL3	NM_003946	nucleolar protein 3 (apoptosis repressor with CARD domain)	-2.2851138	down
DARC	NM_002036	Duffy blood group, chemokine receptor	-2.721878	down
REM1	NM_014012	RAS (RAD and GEM)-like GTP-binding 1	-2.496624	down
CALHM3	NM_001129742	calcium homeostasis modulator 3	-2.9816327	down
EPS8L1	NM_133180	EPS8-like 1	-2.1542614	down
FAM179A		family with sequence similarity 179, member A	-2.0284348	down
			-3.6973152	down
	BX457161		-3.0089655	down
RIMKLB	NM_020734	ribosomal modification protein rimK-like family member B	-2.3866327	down
LUZP1	BC051733	leucine zipper protein 1	-2.8773108	down
NEDD4L	NM_001144967	neural precursor cell expressed, developmentally down-regulated 4-like	-2.0620732	down
	AK026812		-2.6088536	down
CHRNA4	NM_000744	cholinergic receptor, nicotinic, alpha 4	-2.7507045	down
			-2.6687324	down
	CB962925		-6.2553062	down
ZNF135	NM_003436	zinc finger protein 135	-2.5344617	down
	DA571569		-2.8202133	down
CDC42EP1	NM_152243	CDC42 effector protein (Rho GTPase binding) 1	-2.139466	down
FAM123C	NM_152698	family with sequence similarity 123C	-2.7864103	down
PGA3	NM_001079807	pepsinogen 3, group I (pepsinogen A)	-2.4115167	down
ETV6	NM_001987	ets variant 6	-2.0668702	down
GRHL3	NM_198174	grainyhead-like 3 (Drosophila)	-4.2577024	down
LOC100130872	NR_024569	uncharacterized LOC100130872	-2.1362271	down
TMEM51	NM_001136216	transmembrane protein 51	-2.8962402	down
LOC255411	NR_029448	uncharacterized LOC255411	-2.5803335	down
IRX4	NM_016358	iroquois homeobox 4	-2.227684	down
ADARB2	NM_018702	adenosine deaminase, RNA-specific, B2	-2.1071332	down
	XM_003403511		-5.0659294	down

LINC00483	AK000701	long intergenic non-protein coding RNA 483	-3.1257324	down
	AK057884		-2.1409512	down
EIF4E1B	NM_001099408	eukaryotic translation initiation factor 4E family member 1B	-3.3814898	down
	AK096159		-3.352346	down
PDE8B	NM_003719	phosphodiesterase 8B	-5.3174696	down
			-2.2083623	down
NRXN3	NM_004796	neurexin 3	-2.1737566	down
C6orf154	NM_001012974	chromosome 6 open reading frame 154	-2.7284353	down
OR51A2	NM_001004748	olfactory receptor, family 51, subfamily A, member 2	-2.214204	down
SLC16A10	NM_018593	solute carrier family 16, member 10 (aromatic amino acid transporter)	-2.1475823	down
			-2.2522726	down
CT47A11	NM_173571	cancer/testis antigen family 47, member A11	-2.2315848	down
C2orf81	NM_001145054	chromosome 2 open reading frame 81	-2.946376	down
TBX3	NM_016569	T-box 3	-3.092287	down
LOC284454	NR_036515	uncharacterized LOC284454	-2.3870957	down
SEC61A2	NM_018144	Sec61 alpha 2 subunit (S. cerevisiae)	-2.6974995	down
COL8A1	NM_001850	collagen, type VIII, alpha 1	-3.1609988	down
SYN3	NM_133633	synapsin III	-2.006292	down
SLC22A25	NM_199352	solute carrier family 22, member 25	-2.47531	down
PLBD2	NM_173542	phospholipase B domain containing 2	-2.3007019	down
VSX1	NM_199425	visual system homeobox 1	-2.512559	down
	XR_110849		-2.069769	down
			-2.5725393	down
			-3.1031451	down
C19orf69	NM_001130514	chromosome 19 open reading frame 69	-2.3431609	down
NFATC2IP	NM_032815	nuclear factor of activated T-cells, cytoplasmic, calcineurin-dependent 2 interacting protein	-2.4465878	down
BTNL2	NM_019602	butyrophilin-like 2 (MHC class II associated)	-2.7211523	down
HES3	NM_001024598	hairy and enhancer of split 3 (Drosophila)	-2.6749797	down
UBD	NM_006398	ubiquitin D	-4.809574	down
OR7D4	NM_001005191	olfactory receptor, family 7, subfamily D, member 4	-3.0526707	down
ENDOV	NM_173627	endonuclease V	-3.2813058	down
			-3.242217	down
S100B		S100 calcium binding protein B	-2.3076413	down
TRIM46	NM_025058	tripartite motif containing 46	-3.551807	down
CACNA1C	NM_199460	calcium channel, voltage-dependent, L type, alpha 1C subunit	-2.6821473	down
PRMT8	NM_019854	protein arginine methyltransferase 8	-2.2988193	down
MGAT4C	NM_013244	mannosyl (alpha-1,3-)-glycoprotein beta-1,4-N-acetylglucosaminyltransferase, isozyme C (putative)	-2.0604637	down
RBFOX2	NM_001031695	RNA binding protein, fox-1 homolog (C. elegans) 2	-6.778945	down
TMEM37		transmembrane protein 37	-2.0115738	down
SLC22A2	NM_003058	solute carrier family 22 (organic cation transporter), member 2	-2.0932717	down

	XR_110942		-6.1331234	down
	X81001		-2.7846134	down
CXorf28	NR_038428	chromosome X open reading frame 28	-2.0084805	down
			-2.9862378	down
LOC100129775	AK094853	uncharacterized LOC100129775	-3.8658996	down
MGC4859	BC002644	uncharacterized LOC79150	-2.8889797	down
SENP1	NM_014554	SUMO1/sentrin specific peptidase 1	-2.44317	down
CRB2	NM_173689	crumbs homolog 2 (Drosophila)	-3.0469072	down
BPIFA3	NM_178466	BPI fold containing family A, member 3	-2.1466963	down
SCN5A	NM_001099404	sodium channel, voltage-gated, type V, alpha subunit	-2.1593676	down
LOC283856	NR_027078	uncharacterized LOC283856	-3.9197576	down
GRIK2	NM_021956	glutamate receptor, ionotropic, kainate 2	-2.0101447	down
NPFFR1	NM_022146	neuropeptide FF receptor 1	-2.3120403	down
PARVB	AK309987	parvin, beta	-2.1484697	down
	AK130794		-2.8214889	down
			-2.4623194	down
KCNJ6	NM_002240	potassium inwardly-rectifying channel, subfamily J, member 6	-2.2140284	down
			-2.734938	down
FPGT-TNNI3K	BX640903	FPGT-TNNI3K readthrough	-3.0323894	down
LOC100130238	NR_024563	uncharacterized LOC100130238	-2.4025908	down
AK7	NM_152327	adenylate kinase 7	-2.3729737	down
	AK097143		-2.0021162	down
LOC202181	NR_026921	chromosome 5 open reading frame 25 pseudogene	-6.883093	down
			-4.0024467	down
ADAMTSL1	NM_052866	ADAMTS-like 1	-2.2870672	down
VWA5B1	AK125833	von Willebrand factor A domain containing 5B1	-4.0214868	down
ZNF205	NM_003456	zinc finger protein 205	-3.5070689	down
HAS3	NM_138612	hyaluronan synthase 3	-2.525828	down
SNX30	NM_001012994	sorting nexin family member 30	-2.224706	down
			-3.3599677	down
NKPD1	NM_198478	NTPase, KAP family P-loop domain containing 1	-4.0329967	down
P4HA3	NM_182904	prolyl 4-hydroxylase, alpha polypeptide III	-2.103879	down
SLC26A9	NM_052934	solute carrier family 26, member 9	-2.0560274	down
DEFB128	NM_001037732	defensin, beta 128	-2.638117	down
SPTSSB	BC130565	serine palmitoyltransferase, small subunit B	-2.507794	down
			-4.0071855	down
	BF106382		-2.3401406	down
TPSD1	NM_012217	tryptase delta 1	-2.5900114	down
ETV3	NM_001145312	ets variant 3	-2.7701516	down
LOC149134	NR_015422	uncharacterized LOC149134	-2.342262	down
NYNRIN	NM_025081	NYN domain and retroviral integrase containing	-2.2675173	down

VWA5A	NM_198315	von Willebrand factor A domain containing 5A	-2.3822362	down
			-2.776681	down
ZNF774	NM_001004309	zinc finger protein 774	-2.1446857	down
METTL21A	NM_001127395	methyltransferase like 21A	-3.6845732	down
FBXO43	NM_001029860	F-box protein 43	-2.5005777	down
	AK123993		-2.828009	down
DGCR9	NR_024159	DiGeorge syndrome critical region gene 9	-2.7433918	down
MUC5B	NM_002458	mucin 5B, oligomeric mucus/gel-forming	-6.858593	down
LOC100653259	XR_133099	e3 ubiquitin-protein ligase HERC2-like	-3.6758735	down
LOC440993	XR_110395	uncharacterized LOC440993	-2.7566223	down
PRAMEF2	NM_023014	PRAME family member 2	-2.755429	down
GCLM	NM_002061	glutamate-cysteine ligase, modifier subunit	-2.3291495	down
			-5.762676	down
SLC35A4	AF445025	solute carrier family 35, member A4	-2.3749065	down
TBCD	AL096745	tubulin folding cofactor D	-3.2921207	down
	XR_110925		-2.1033428	down
			-2.5059135	down
GRIN2D	NM_000836	glutamate receptor, ionotropic, N-methyl D-aspartate 2D	-2.127539	down
RAB7B	NM_177403	RAB7B, member RAS oncogene family	-2.7598479	down
			-2.2084846	down
XRCC2	NM_005431	X-ray repair complementing defective repair in Chinese hamster cells 2	-3.9412048	down
FAM24A	NM_001029888	family with sequence similarity 24, member A	-2.546498	down
SP6	NM_199262	Sp6 transcription factor	-2.0697722	down
ALX1	NM_006982	ALX homeobox 1	-2.365797	down
C10orf71	NM_001135196	chromosome 10 open reading frame 71	-2.0601904	down
KIAA0090		KIAA0090	-4.023962	down
GGT6	NM_153338	gamma-glutamyltransferase 6	-5.5791426	down
NSAP11	XR_110862	nervous system abundant protein 11	-2.004151	down
MASP2	NM_006610	mannan-binding lectin serine peptidase 2	-3.7483637	down
LOC644649	BX108667	apolipoprotein O pseudogene	-2.2276397	down
KIAA0087	NR_022006	KIAA0087	-2.3426142	down
LOC283693	NR_036446	actin, gamma pseudogene	-2.2174149	down
			-3.9314988	down
TTC7A	NM_020458	tetratricopeptide repeat domain 7A	-2.294366	down
MCF2L		MCF.2 cell line derived transforming sequence-like	-2.5119946	down
BGN	NM_001711	biglycan	-5.2434115	down
PADI3	NM_016233	peptidyl arginine deiminase, type III	-2.3341827	down
	AK125749		-2.8895736	down
SIGLEC6	NM_001245	sialic acid binding Ig-like lectin 6	-5.1416054	down
FAM92A3	NR_003612	family with sequence similarity 92, member A3	-5.862625	down
GTF2IRD2B		GTF2I repeat domain containing 2B	-3.0058002	down

C5orf58	NM_001102609	chromosome 5 open reading frame 58	-2.3745775	down
JAG2	NM_002226	jagged 2	-2.342133	down
S100A3	NM_002960	S100 calcium binding protein A3	-3.446978	down
LINC00277	NR_026949	long intergenic non-protein coding RNA 277	-2.0005004	down
CACNA1G	NM_018896	calcium channel, voltage-dependent, T type, alpha 1G subunit	-2.7417939	down
C1orf21	NM_030806	chromosome 1 open reading frame 21	-3.111706	down
	AF116661		-2.8661096	down
KIAA1161	NM_020702	KIAA1161	-2.7705297	down
			-2.3838866	down
CTNS		cystinosin, lysosomal cystine transporter	-2.8170528	down
C1QL2	NM_182528	complement component 1, q subcomponent-like 2	-4.247638	down
ATP13A2	NM_022089	ATPase type 13A2	-2.0642447	down
HERC2P7	XR_133065	hect domain and RLD 2 pseudogene 7	-2.542685	down
P2RY6	NM_176798	pyrimidinergic receptor P2Y, G-protein coupled, 6	-2.18919	down
			-3.347469	down
			-2.7305565	down
C19orf63	NM_206538	chromosome 19 open reading frame 63	-2.4077103	down
ILDR1	NM_175924	immunoglobulin-like domain containing receptor 1	-2.6286054	down
GAFA1	AF190748	FGF2-associated protein GAFA1	-2.0848186	down
LOC100499221	AK127052	uncharacterized LOC100499221	-4.90141	down
HERC2	NM_004667	hect domain and RLD 2	-2.2911088	down
VHL	NM_000551	von Hippel-Lindau tumor suppressor	-3.0923693	down
GRIA1	NM_000827	glutamate receptor, ionotropic, AMPA 1	-2.0847723	down
			-2.5602794	down
RGL1	NM_015149	ral guanine nucleotide dissociation stimulator-like 1	-5.1065435	down
MPV17L	NM_173803	MPV17 mitochondrial membrane protein-like	-3.6402934	down
			-2.7281613	down
	BC014063		-3.6603844	down
NRIP3	NM_020645	nuclear receptor interacting protein 3	-2.0406232	down
			-2.2177672	down
			-3.3741927	down
	BC040611		-2.7199779	down
	AF514420		-2.4019191	down
NTF3	NM_002527	neurotrophin 3	-2.3882217	down
ZNF667	NM_022103	zinc finger protein 667	-2.1410785	down
OR10A6	NM_001004461	olfactory receptor, family 10, subfamily A, member 6	-3.0233197	down
LOC284219	AK094436	uncharacterized LOC284219	-6.937428	down
SIX6	NM_007374	SIX homeobox 6	-3.0015733	down
CCDC9	NM_015603	coiled-coil domain containing 9	-2.431223	down
LAMA2	NM_000426	laminin, alpha 2	-2.1883686	down
OR4N4	NM_001005241	olfactory receptor, family 4, subfamily N, member 4	-4.0046897	down

DNM1P46	NR_003260	DNM1 pseudogene 46	-2.9753864	down
PDCL2	NM_152401	phosducin-like 2	-2.1179345	down
KIAA1751	NM_001080484	KIAA1751	-2.0209827	down
LOC338758	NR_028138	uncharacterized LOC338758	-2.1287215	down
	BM981407		-5.814292	down
E2F7	NM_203394	E2F transcription factor 7	-2.8482833	down
FAM181B	NM_175885	family with sequence similarity 181, member B	-2.804772	down
CLPTM1	NM_001294	cleft lip and palate associated transmembrane protein 1	-2.5246377	down
AZFP	XR_109896	AML-associated zinc finger protein	-2.305171	down
MDH1B	AK309144	malate dehydrogenase 1B, NAD (soluble)	-4.026212	down
PGAM5	NM_138575	phosphoglycerate mutase family member 5	-2.82386	down
	XM_002342821		-4.8995767	down
LOC729609	NR_024440	uncharacterized LOC729609	-2.7213964	down
C14orf167	NR_023921	chromosome 14 open reading frame 167	-3.4821274	down
GNG12	NM_018841	guanine nucleotide binding protein (G protein), gamma 12	-2.5622551	down
FLJ10661	NR_024362	family with sequence similarity 86, member A pseudogene	-3.8406982	down
ERVW-1	NM_014590	endogenous retrovirus group W, member 1	-4.2838273	down
RPH3AL	NM_006987	rabphilin 3A-like (without C2 domains)	-3.1777015	down
FAM201B	BC045732	family with sequence similarity 201, member B	-4.605173	down
	XR_108612		-5.1520066	down
			-3.4537048	down
ARHGAP42	NM_152432	Rho GTPase activating protein 42	-2.7685244	down
LOC84931	NR_027181	uncharacterized LOC84931	-2.1107287	down
C8orf74	NM_001040032	chromosome 8 open reading frame 74	-3.8976905	down
LOC100507547	NR_037169	uncharacterized LOC100507547	-3.1023297	down
CPLX2	NM_006650	complexin 2	-3.2364979	down
ND1	BC061915	NADH dehydrogenase, subunit 1 (complex I)	-2.0146625	down
HOMER3	NM_001145724	homer homolog 3 (Drosophila)	-2.2147903	down
PRODH2	NM_021232	proline dehydrogenase (oxidase) 2	-2.0751872	down
CD86	NM_006889	CD86 molecule	-6.0708394	down
LOC643719	NR_027620	uncharacterized LOC643719	-2.312933	down
TBX4	NM_018488	T-box 4	-2.0030367	down
FAM19A4	NM_182522	family with sequence similarity 19 (chemokine (C-C motif)-like), member A4	-2.1570458	down
POU4F3	NM_002700	POU class 4 homeobox 3	-2.1781178	down
	BC007749		-4.605416	down
	BC035135		-4.0899434	down
AMZ1	NM_133463	archaelysin family metallopeptidase 1	-3.0072765	down
PAR4	NR_022010	Prader-Willi/Angelman region gene 4	-7.4043956	down
TCFL5	BC046933	transcription factor-like 5 (basic helix-loop-helix)	-2.0931942	down
ALDH1L2	NM_001034173	aldehyde dehydrogenase 1 family, member L2	-2.5476327	down

			-21.25616	down
ADAMTS4	BC030812	ADAM metallopeptidase with thrombospondin type 1 motif, 4	-2.774159	down
VPS18	NM_020857	vacuolar protein sorting 18 homolog (S. cerevisiae)	-2.7445157	down
GRIA4	NM_001077243	glutamate receptor, ionotrophic, AMPA 4	-2.414727	down
BCL11B	NM_138576	B-cell CLL/lymphoma 11B (zinc finger protein)	-2.95319	down
FER1L5	NM_001113382	fer-1-like 5 (C. elegans)	-2.0715592	down
ZNF852	XM_001717544	zinc finger protein 852	-2.7356296	down
RND1	NM_014470	Rho family GTPase 1	-2.352077	down
LOC389906	NR_034031	zinc finger protein 839 pseudogene	-2.572595	down
TCEB3B	NM_016427	transcription elongation factor B polypeptide 3B (elongin A2)	-3.5261405	down
C7orf10	NM_024728	chromosome 7 open reading frame 10	-2.821668	down
C10orf47	NM_153256	chromosome 10 open reading frame 47	-2.5315373	down
			-3.031054	down
BNIP3L	AF370457	BCL2/adenovirus E1B 19kDa interacting protein 3-like	-2.8183804	down
MGC14436	NR_026661	uncharacterized LOC84983	-2.1342783	down
TBC1D3	AK122833	TBC1 domain family, member 3	-2.378262	down
MCC	NM_001085377	mutated in colorectal cancers	-2.6274388	down
LOC283403	NM_001242696	uncharacterized LOC283403	-2.4312103	down
IFFO1	NR_036467	intermediate filament family orphan 1	-2.5381324	down
			-2.6310046	down
LOC284373	BX537909	uncharacterized LOC284373	-2.0076368	down
KCNB2	NM_004770	potassium voltage-gated channel, Shab-related subfamily, member 2	-2.4696224	down
FLJ45831	XR_109367	uncharacterized FLJ45831	-2.4534793	down
KCTD19	NM_001100915	potassium channel tetramerisation domain containing 19	-2.0930295	down
BMPER	AB075845	BMP binding endothelial regulator	-2.4310462	down
			-3.5182805	down
	XR_111612		-5.910486	down
FAM22F	NM_017561	family with sequence similarity 22, member F	-2.226387	down
CCDC50	NM_178335	coiled-coil domain containing 50	-2.0695076	down
			-2.2071064	down
			-2.3678603	down
DNMT3A	NM_175629	DNA (cytosine-5-)-methyltransferase 3 alpha	-2.0584514	down
SPAG17	NM_206996	sperm associated antigen 17	-2.1135762	down
ARHGAP21	BX537570	Rho GTPase activating protein 21	-2.3107944	down
			-2.2358017	down
LOC100131066	AK126015	uncharacterized LOC100131066	-2.6286132	down
HDAC10	NM_032019	histone deacetylase 10	-2.5226002	down
LOC440386	BC029817	uncharacterized LOC440386	-2.2441561	down
LOC344967	NR_027277	acyl-CoA thioesterase 7 pseudogene	-2.1843812	down
SERPINB7	NM_001040147	serpin peptidase inhibitor, clade B (ovalbumin), member 7	-2.9745972	down
GABRB1	AK296023	gamma-aminobutyric acid (GABA) A receptor, beta 1	-2.3036993	down

ERVFRD-2	AK127846	endogenous retrovirus group FRD, member 2	-3.3377197	down
	AF074986		-2.3169346	down
LAMC2	NM_005562	laminin, gamma 2	-2.3459158	down
	AB306172		-2.4201303	down
YWHAE	AK296555	tyrosine 3-monooxygenase/tryptophan 5-monooxygenase activation protein, epsilon polypeptide	-2.908293	down
LOC100507547	NR_037169	uncharacterized LOC100507547	-2.4733112	down
MAP3K6	NM_004672	mitogen-activated protein kinase kinase kinase 6	-2.601848	down
LOC728158	AK125527	hCG2044975	-2.263453	down
C20orf85	NM_178456	chromosome 20 open reading frame 85	-2.8147066	down
			-3.5980184	down
PER2	NM_022817	period homolog 2 (Drosophila)	-2.9666555	down
CDH4	NM_001794	cadherin 4, type 1, R-cadherin (retinal)	-6.0878124	down
02-mar	NM_017898	mitochondrial amidoxime reducing component 2	-2.0407164	down
CDH2	NM_001792	cadherin 2, type 1, N-cadherin (neuronal)	-3.714384	down
C9orf131	NM_203299	chromosome 9 open reading frame 131	-2.3778877	down
PMS2L2	BC010535	postmeiotic segregation increased 2-like 2 pseudogene	-2.4203024	down
GH2	NM_022558	growth hormone 2	-2.3368669	down
MYO1C	NM_033375	myosin IC	-2.173801	down
GTF2IRD2	NM_173537	GTF2I repeat domain containing 2	-2.7550595	down
LOC100128703	AK128830	uncharacterized LOC100128703	-2.4644034	down
BICC1	NM_001080512	bicaudal C homolog 1 (Drosophila)	-3.3942635	down
LYPD3	NM_014400	LY6/PLAUR domain containing 3	-2.2153616	down
HFE2	NM_213653	hemochromatosis type 2 (juvenile)	-2.420331	down
PRDM15	NM_001040424	PR domain containing 15	-2.3053489	down
ENTPD8	NM_001033113	ectonucleoside triphosphate diphosphohydrolase 8	-3.736031	down
FAM40A	NM_033088	family with sequence similarity 40, member A	-2.442973	down
TECPR2	NM_001172631	tectonin beta-propeller repeat containing 2	-2.1858718	down
			-3.782386	down
VIT	NM_053276	vitrin	-2.0297961	down
			-2.0486994	down
			-4.886796	down
LOC284581	AK094426	uncharacterized LOC284581	-9.800617	down
THNSL2	NM_001244676	threonine synthase-like 2 (S. cerevisiae)	-3.135761	down
SPACA1	NM_030960	sperm acrosome associated 1	-3.5610242	down
MAD2L2		MAD2 mitotic arrest deficient-like 2 (yeast)	-3.2875857	down
FAM27A	NR_024060	family with sequence similarity 27, member A	-2.4772813	down
GRAP	NM_006613	GRB2-related adaptor protein	-2.0227585	down
FUT11	BC100994	fucosyltransferase 11 (alpha (1,3) fucosyltransferase)	-2.0068507	down
			-2.789328	down
			-2.1246498	down

SLC14A2	NM_007163	solute carrier family 14 (urea transporter), member 2	-2.5524795	down
	AK021797		-2.5752614	down
FAM3A	NM_001171134	family with sequence similarity 3, member A	-2.1456265	down
ARHGEF35	NM_001003702	Rho guanine nucleotide exchange factor (GEF) 35	-2.4864452	down
NS3BP	XR_109071	NS3BP	-2.2801344	down
HELT	NM_001029887	helt bHLH transcription factor	-2.0148091	down
C4orf19	NM_018302	chromosome 4 open reading frame 19	-2.4065623	down
POU6F2	NM_007252	POU class 6 homeobox 2	-2.414026	down
	XR_133518		-2.5860279	down
LOC63930	NR_033370	uncharacterized LOC63930	-2.8614426	down
BMP8A	NM_181809	bone morphogenetic protein 8a	-2.676179	down
PPP1R13L	NM_006663	protein phosphatase 1, regulatory subunit 13 like	-2.252408	down
LINC00301	NR_026946	long intergenic non-protein coding RNA 301	-4.916239	down
HIST3H2A	NM_033445	histone cluster 3, H2a	-2.0041134	down
COL6A5	NM_153264	collagen, type VI, alpha 5	-2.7317326	down
ZNF704	NM_001033723	zinc finger protein 704	-3.5323913	down
LOC727982	NR_034134	uncharacterized LOC727982	-2.262424	down
			-3.0192158	down
			-3.394756	down
MAGEE2	NM_138703	melanoma antigen family E, 2	-2.00022	down
			-3.1643271	down
EFCAB10	NR_027068	EF-hand calcium binding domain 10	-2.418079	down
FAM41C	NR_027055	family with sequence similarity 41, member C	-3.758045	down
			-3.4781518	down
CLDN14	NM_144492	claudin 14	-3.1466837	down
TMEM179	NM_207379	transmembrane protein 179	-2.9599962	down
KCTD3	NM_016121	potassium channel tetramerisation domain containing 3	-2.715921	down
PROKR1	NM_138964	prokineticin receptor 1	-2.236533	down
	DB155821		-2.791482	down
			-2.2949421	down
FLJ22763	NR_033977	uncharacterized LOC401081	-2.2647824	down
			-2.5410364	down
TMEM184A	NM_001097620	transmembrane protein 184A	-3.2429647	down
FERMT1	NM_017671	fermitin family member 1	-2.7994456	down
C14orf64	NR_015430	chromosome 14 open reading frame 64	-2.30505	down
PRKAR1B	NM_001164761	protein kinase, cAMP-dependent, regulatory, type I, beta	-2.5395684	down
B3GALNT2	NM_152490	beta-1,3-N-acetylgalactosaminyltransferase 2	-2.0110734	down
			-3.3423624	down
TNFRSF19	BC035796	tumor necrosis factor receptor superfamily, member 19	-6.1958547	down
SYT2	NM_001136504	synaptotagmin II	-3.063535	down
SLC15A1	AB001328	solute carrier family 15 (oligopeptide transporter), member 1	-2.1222937	down

HIST1H2BE	NM_003523	histone cluster 1, H2be	-2.0254223	down
MANEA	NM_024641	mannosidase, endo-alpha	-2.4143198	down
	AK093443		-2.3377457	down
LOC100128591	AK128705	uncharacterized LOC100128591	-3.0677636	down
VRTN	NM_018228	vertebrae development homolog (pig)	-3.349385	down
FAM149A	NM_015398	family with sequence similarity 149, member A	-2.9712298	down
FUBP3	NM_003934	far upstream element (FUSE) binding protein 3	-2.7376785	down
HSD11B2	NM_000196	hydroxysteroid (11-beta) dehydrogenase 2	-2.1148684	down
LOC157627	NR_024281	uncharacterized LOC157627	-3.7326648	down
TUSC5	NM_172367	tumor suppressor candidate 5	-2.5799549	down
NLGN4X	NM_020742	neuroligin 4, X-linked	-2.2987318	down
CLCF1	NM_013246	cardiotrophin-like cytokine factor 1	-2.1565404	down
TLCD2	NM_001164407	TLC domain containing 2	-2.198005	down
LOC284950	NR_038888	uncharacterized LOC284950	-2.9387305	down
SCRT2	NM_033129	scratch homolog 2, zinc finger protein (Drosophila)	-2.2713118	down
			-4.360089	down
ANGPTL2	NM_012098	angiopoietin-like 2	-2.3287864	down
C12orf28	XM_001716650	chromosome 12 open reading frame 28	-2.132765	down
KCNK4	NM_033310	potassium channel, subfamily K, member 4	-2.363637	down
RAD23B	NM_002874	RAD23 homolog B (S. cerevisiae)	-2.168622	down
DMKN	NM_033317	dermokine	-2.1554956	down
LOC389831	NM_001242480	uncharacterized LOC389831	-3.4519672	down
LOC100506310	XM_003118845	uncharacterized protein C1orf167-like	-2.6940622	down
C14orf178	NM_174943	chromosome 14 open reading frame 178	-2.2258563	down
MGC23270	NR_024396	uncharacterized LOC196872	-2.5431976	down
PDE4DIP	NM_022359	phosphodiesterase 4D interacting protein	-3.0068479	down
KRTAP10-5	NM_198694	keratin associated protein 10-5	-3.916554	down
MGP	NM_000900	matrix Gla protein	-2.1918366	down
KRTAP3-3	NM_033185	keratin associated protein 3-3	-2.7511568	down
LOC100129785	AK125285	uncharacterized LOC100129785	-7.7528396	down
IL29	NM_172140	interleukin 29 (interferon, lambda 1)	-2.1498632	down
SUV420H2	AK304562	suppressor of variegation 4-20 homolog 2 (Drosophila)	-2.475215	down
ZC3H12D	NM_207360	zinc finger CCCH-type containing 12D	-3.0985065	down
			-2.541297	down
			-2.0397563	down
ST3GAL4	AK021929	ST3 beta-galactoside alpha-2,3-sialyltransferase 4	-7.044811	down
LOC100506310	XR_132699	uncharacterized protein C1orf167-like	-2.4711325	down
LOC652586	XM_001719844	uncharacterized LOC652586	-2.2931592	down
			-3.1073565	down
			-2.364695	down
SLC30A2	NM_001004434	solute carrier family 30 (zinc transporter), member 2	-2.9452925	down

SLC24A1	NM_004727	solute carrier family 24 (sodium/potassium/calcium exchanger), member 1	-2.394274	down
ZNF468	NM_199132	zinc finger protein 468	-2.8107257	down
TMEM146	AK128088	transmembrane protein 146	-3.2647536	down
ARHGEF17	NM_014786	Rho guanine nucleotide exchange factor (GEF) 17	-2.1165524	down
			-2.2147217	down
HMGCS2	NM_005518	3-hydroxy-3-methylglutaryl-CoA synthase 2 (mitochondrial)	-2.0311007	down
SPATA13	NM_153023	spermatogenesis associated 13	-2.640243	down
GRXCR2	NM_001080516	glutaredoxin, cysteine rich 2	-2.2865386	down
ONECUT1	NM_004498	one cut homeobox 1	-2.0443244	down
OR13D1	NM_001004484	olfactory receptor, family 13, subfamily D, member 1	-2.1143165	down
OR51I1	NM_001005288	olfactory receptor, family 51, subfamily I, member 1	-3.1008372	down
KCNE4	NM_080671	potassium voltage-gated channel, Isk-related family, member 4	-5.6199284	down
	U66047		-4.487957	down
FGFR3	NM_000142	fibroblast growth factor receptor 3	-3.825862	down
FAM108A1	AK090438	family with sequence similarity 108, member A1	-2.768594	down
AFAP1-AS1	NR_026892	AFAP1 antisense RNA 1 (non-protein coding)	-2.1451848	down
ARPM1	NM_032487	actin related protein M1	-3.146077	down
			-2.0443058	down
			-2.325933	down
FLJ46066	NR_040105	uncharacterized LOC401103	-2.317953	down
BOD1P	BM559531	biorientation of chromosomes in cell division 1 pseudogene	-2.5467656	down
MAMSTR	NM_182574	MEF2 activating motif and SAP domain containing transcriptional regulator	-2.9105282	down
DHRS2	NM_182908	dehydrogenase/reductase (SDR family) member 2	-3.0704036	down
REEP6	NM_138393	receptor accessory protein 6	-2.875117	down
RCOR2	NM_173587	REST corepressor 2	-3.462535	down
KRTAP3-2	NM_031959	keratin associated protein 3-2	-2.2148068	down
FLJ21408	AK025061	uncharacterized LOC400512	-4.825887	down
FAT3	NM_001008781	FAT tumor suppressor homolog 3 (Drosophila)	-2.8852012	down
KRT222	NM_152349	keratin 222	-5.3459196	down
	BM930849		-3.6658337	down
SCN9A	NM_002977	sodium channel, voltage-gated, type IX, alpha subunit	-3.8044028	down
PROM2	NM_001165978	prominin 2	-7.005659	down
SRCIN1	NM_025248	SRC kinase signaling inhibitor 1	-4.1044054	down
PIP	NM_002652	prolactin-induced protein	-7.557286	down
KRTAP9-1	NM_001190460	keratin associated protein 9-1	-2.895403	down
FAM176A	NM_032181	family with sequence similarity 176, member A	-2.3179898	down
PCDHGA3	NM_032011	protocadherin gamma subfamily A, 3	-2.0073564	down
SHANK1	NM_016148	SH3 and multiple ankyrin repeat domains 1	-4.383421	down
STGC3	XR_110003	uncharacterized STGC3	-2.004961	down
DKFZp451A211	XM_003403663	DKFZp451A211 protein	-3.0595014	down

LOC100130452	NR_034036	uncharacterized LOC100130452	-2.2544987	down
NR2F1	NM_005654	nuclear receptor subfamily 2, group F, member 1	-2.361522	down
ARHGAP39	NM_025251	Rho GTPase activating protein 39	-2.9829621	down
LOC646498	NM_001080528	uncharacterized LOC646498	-3.8746393	down
SP9	NM_001145250	Sp9 transcription factor homolog (mouse)	-2.0920312	down
			-2.6245832	down
			-2.7872555	down
SNORA70B	BQ029156	small nucleolar RNA, H/ACA box 70B (retrotransposed)	-2.2352493	down
	AK128215		-2.1363661	down
	BX093647		-2.2324064	down
OR2T4	NM_001004696	olfactory receptor, family 2, subfamily T, member 4	-2.253095	down
TBC1D28	NM_001039397	TBC1 domain family, member 28	-2.87844	down
C3orf54	NM_203370	chromosome 3 open reading frame 54	-2.4272845	down
LOC100129048	AK128074	uncharacterized LOC100129048	-2.5689895	down
C1orf61	NM_006365	chromosome 1 open reading frame 61	-2.1435628	down
SSX4B	NM_001034832	synovial sarcoma, X breakpoint 4B	-4.047795	down
PDXDC2P	BX647358	pyridoxal-dependent decarboxylase domain containing 2, pseudogene	-4.094984	down
RNF150	NM_020724	ring finger protein 150	-5.1244707	down
LOC647983	AK126828	uncharacterized LOC647983	-4.8871355	down
GOLGA2P2Y	NR_001555	golgin A2 pseudogene 2, Y-linked	-2.5211184	down
LINC00314	NR_027246	long intergenic non-protein coding RNA 314	-2.2244236	down
LRRC46	NM_033413	leucine rich repeat containing 46	-2.0385394	down
DGCR10	NR_026651	DiGeorge syndrome critical region gene 10	-3.6058676	down
	AK094155		-5.0520654	down
LENG9	NM_198988	leukocyte receptor cluster (LRC) member 9	-2.007832	down
C16orf3	NM_001214	chromosome 16 open reading frame 3	-5.961076	down
CMKLR1	NM_004072	chemokine-like receptor 1	-2.3664968	down
RPL32P3	NR_003111	ribosomal protein L32 pseudogene 3	-2.1416872	down
UMODL1	NM_001199528	uromodulin-like 1	-4.0061846	down
PCDHB8	NM_019120	protocadherin beta 8	-2.515205	down
SIX3	NM_005413	SIX homeobox 3	-3.9609032	down
AGFG2	NM_006076	ArfGAP with FG repeats 2	-2.0649567	down
OR2AT4	NM_001005285	olfactory receptor, family 2, subfamily AT, member 4	-3.5410466	down
SEC24A	NM_001252231	SEC24 family, member A (S. cerevisiae)	-2.012962	down
LRRC19	NM_022901	leucine rich repeat containing 19	-3.5081592	down
TSSK1B	NM_032028	testis-specific serine kinase 1B	-2.1378126	down
			-2.7558503	down
ARMS2	NM_001099667	age-related maculopathy susceptibility 2	-2.514969	down
PAX2	NM_003990	paired box 2	-4.706431	down
LOC284014	AK095567	uncharacterized LOC284014	-3.6832788	down
			-3.682595	down

			-3.0128133	down
INMT	NM_001199219	indolethylamine N-methyltransferase	-4.5414267	down
KIAA1257	NM_020741	KIAA1257	-2.6012099	down
STRC	NM_153700	stereocilin	-2.3872378	down
			-2.4237964	down
			-2.1300905	down
C7orf61	NM_001004323	chromosome 7 open reading frame 61	-4.073289	down
RNASET2	AK124363	ribonuclease T2	-5.269457	down
PRIC285	BC104974	peroxisomal proliferator-activated receptor A interacting complex 285	-2.3447983	down
	AF220263		-2.933544	down
LOC339539	BC043541	uncharacterized LOC339539	-2.9785721	down
LOC100289079	XM_002344448	60S ribosomal protein L36-like	-2.9898376	down
OR7G2	NM_001005193	olfactory receptor, family 7, subfamily G, member 2	-2.5025396	down
PAPPA	NM_002581	pregnancy-associated plasma protein A, pappalysin 1	-2.2928572	down
KRTAP19-1	NM_181607	keratin associated protein 19-1	-6.9071665	down
			-3.9675622	down
			-2.0686378	down
SLC2A11		solute carrier family 2 (facilitated glucose transporter), member 11	-2.0024207	down
MAGEC2	NM_016249	melanoma antigen family C, 2	-3.9414883	down
ZNF248	BC021819	zinc finger protein 248	-2.340402	down
GAFA2	AF220234	FGF-2 activity-associated protein 2	-2.0031347	down
SULT4A1	NM_014351	sulfotransferase family 4A, member 1	-3.5427861	down
DCUN1D2	NM_001014283	DCN1, defective in cullin neddylation 1, domain containing 2 (S. cerevisiae)	-3.6211264	down
LOC283483	XR_109153	uncharacterized LOC283483	-2.9179118	down
			-2.3440614	down
LOC100506191	XM_003118995	uncharacterized protein LOC100506191	-2.304706	down
PRDX4		peroxiredoxin 4	-2.0758712	down
			-2.5028021	down
	XR_109614		-2.616706	down
GPSM1	NM_015597	G-protein signaling modulator 1	-3.4216862	down
C2CD4D	NM_001136003	C2 calcium-dependent domain containing 4D	-2.1858833	down
FAM171A1	NM_001010924	family with sequence similarity 171, member A1	-2.046398	down
HIST1H4E	NM_003545	histone cluster 1, H4e	-2.8773859	down
HCAR1	NM_032554	hydroxycarboxylic acid receptor 1	-2.687955	down
	BG957402		-2.1933568	down
ZDHHC8P1	NR_003950	zinc finger, DHHC-type containing 8 pseudogene 1	-3.9944594	down
IKBKB	AK309052	inhibitor of kappa light polypeptide gene enhancer in B-cells, kinase beta	-2.0707302	down
LOC727677	NR_024393	uncharacterized LOC727677	-2.752713	down
	XR_132803		-3.951643	down
CDHR5	NM_031264	cadherin-related family member 5	-2.1542735	down

TNFSF11	NM_003701	tumor necrosis factor (ligand) superfamily, member 11	-3.0199182	down
	AB529275		-2.7506073	down
	BC069659		-3.5412123	down
FFAR3	NM_005304	free fatty acid receptor 3	-2.4922762	down
BBS5	NM_152384	Bardet-Biedl syndrome 5	-3.3318424	down
LOC100133091	NR_029411	uncharacterized LOC100133091	-3.6314611	down
LOC100130256	AK127400	uncharacterized LOC100130256	-2.3596847	down
HCP5B	BC035659	HLA complex P5B (non-protein coding)	-2.2379448	down
MBL1P	NR_002724	mannose-binding lectin (protein A) 1, pseudogene	-2.250458	down
C17orf46	NM_152343	chromosome 17 open reading frame 46	-2.1616297	down
OR2D3	NM_001004684	olfactory receptor, family 2, subfamily D, member 3	-2.0092852	down
OR52W1	NM_001005178	olfactory receptor, family 52, subfamily W, member 1	-2.0155756	down
HIST1H4L	NM_003546	histone cluster 1, H4l	-2.3230183	down
POLR3G	NM_006467	polymerase (RNA) III (DNA directed) polypeptide G (32kD)	-4.865662	down
	AK128523		-3.2098026	down
			-2.2627273	down
SHROOM4	NM_020717	shroom family member 4	-2.698823	down
KIAA1598	NM_018330	KIAA1598	-2.2871034	down
TMEM31	NM_182541	transmembrane protein 31	-2.569111	down
			-3.6364934	down
ZNF81	BC039609	zinc finger protein 81	-2.6136913	down
CHN2	NM_004067	chimerin (chimaerin) 2	-3.0664074	down
ODF2L	NM_001007022	outer dense fiber of sperm tails 2-like	-2.6571915	down
	XR_109707		-3.4660625	down
FOXD1	NM_004472	forkhead box D1	-2.342332	down
LOC100131581	AK092544	uncharacterized LOC100131581	-3.0167692	down
GDF10	NM_004962	growth differentiation factor 10	-3.432062	down
SLC35F2	NM_017515	solute carrier family 35, member F2	-4.473814	down
ORAOV1	BC133014	oral cancer overexpressed 1	-2.090473	down
LOC729080	NR_033244	glycine cleavage system protein H (aminomethyl carrier) pseudogene	-4.1445174	down
TSPEAR	NM_144991	thrombospondin-type laminin G domain and EAR repeats	-2.1994097	down
GPR157	NM_024980	G protein-coupled receptor 157	-2.2411687	down
GOLGA6L10	NM_001164465	golgin A6 family-like 10	-6.690217	down
			-3.3173008	down
TIAL1	AB209260	TIA1 cytotoxic granule-associated RNA binding protein-like 1	-2.5260284	down
LOC100509196	XM_003119833	putative uncharacterized protein encoded by NCRNA00205-like	-2.068929	down
LOC100128653	XR_108727	uncharacterized LOC100128653	-2.6321714	down
GUCA2B	NM_007102	guanylate cyclase activator 2B (uroguanylin)	-2.9069526	down
LOC440461	NR_027283	Rho GTPase activating protein 27 pseudogene	-2.007196	down
LOC728073	AL833395	uncharacterized LOC728073	-4.232102	down
PMP22		peripheral myelin protein 22	-2.4203575	down

ADAM12	NM_003474	ADAM metallopeptidase domain 12	-5.1799927	down
PCBP3-OT1	AK126016	PCBP3 overlapping transcript (non-protein coding)	-4.1296663	down
			-3.3598876	down
KRTAP4-7	NM_033061	keratin associated protein 4-7	-2.7129662	down
			-2.2719307	down
FLJ14186	NR_037596	uncharacterized LOC401149	-2.1709328	down
GUSBP1	BC048193	glucuronidase, beta pseudogene 1	-6.1747327	down
UNC5B	NM_170744	unc-5 homolog B (C. elegans)	-2.8932836	down
			-3.1547675	down
DCAF8L1	NM_001017930	DDB1 and CUL4 associated factor 8-like 1	-2.2685955	down
GRIK4	NM_014619	glutamate receptor, ionotropic, kainate 4	-5.004351	down
CPLX2	NM_006650	complexin 2	-2.1575124	down
MYO3A	NM_017433	myosin IIIA	-6.171576	down
IL12RB2	NM_001559	interleukin 12 receptor, beta 2	-2.40887	down
VSX2	NM_182894	visual system homeobox 2	-2.4018989	down
PPYR1	NM_005972	pancreatic polypeptide receptor 1	-2.1601186	down
LIPH	NM_139248	lipase, member H	-2.4428647	down
KRTAP12-4	NM_198698	keratin associated protein 12-4	-2.1273403	down
			-2.495093	down
MARK4	NM_031417	MAP/microtubule affinity-regulating kinase 4	-2.6387324	down
TSPAN16	NM_012466	tetraspanin 16	-5.547237	down
LOC283112	AK096599	uncharacterized LOC283112	-2.2130513	down
HSD52	NR_027120	uncharacterized LOC729467	-2.3608165	down
TMEM229B	AK090706	transmembrane protein 229B	-2.2172356	down
LOC399939	NM_001206627	ring finger protein 18-like	-4.8642063	down
LOC100288884	AK124122	uncharacterized LOC100288884	-3.7163653	down
LOC729444	NR_038388	uncharacterized LOC729444	-2.3946958	down
AKAP14	NM_001008535	A kinase (PRKA) anchor protein 14	-3.7234235	down
ZDHHC14	NM_153746	zinc finger, DHHC-type containing 14	-2.61771	down
COL6A2	NM_058174	collagen, type VI, alpha 2	-2.2731335	down
PPP6R1	NM_014931	protein phosphatase 6, regulatory subunit 1	-3.7880533	down
LOC100507055	NM_001195520	uncharacterized LOC100507055	-2.3316307	down
LRRC2	NM_024512	leucine rich repeat containing 2	-4.936049	down
HAUS6		HAUS augmin-like complex, subunit 6	-2.1946075	down
	BC094791		-4.1076794	down
ABCC6	NM_001079528	ATP-binding cassette, sub-family C (CFTR/MRP), member 6	-2.3465993	down
COL23A1	NM_173465	collagen, type XXIII, alpha 1	-2.4980407	down
DSCR10	NR_027695	Down syndrome critical region gene 10 (non-protein coding)	-4.523501	down
ARNT2	NM_014862	aryl-hydrocarbon receptor nuclear translocator 2	-3.672476	down
WNT9A	NM_003395	wingless-type MMTV integration site family, member 9A	-2.4781842	down
CALY		calcyon neuron-specific vesicular protein	-3.2764416	down

TAS2R60	NM_177437	taste receptor, type 2, member 60	-7.17155	down
	AK091409		-2.1723459	down
LOC440297	NR_033579	chondroitin sulfate proteoglycan 4 pseudogene	-4.108693	down
FLJ34223	AK091542	uncharacterized LOC440479	-3.636388	down
			-2.0357	down
NXPH3	NM_007225	neurexophilin 3	-5.9469247	down
CDKN1B	NM_004064	cyclin-dependent kinase inhibitor 1B (p27, Kip1)	-2.750145	down
FOSL1	NM_005438	FOS-like antigen 1	-2.878679	down
ODAM	NM_017855	odontogenic, ameloblast asssociated	-2.316448	down
RPA4	NM_013347	replication protein A4, 30kDa	-5.7699113	down
	AK056269		-2.6885736	down
LOC151475	NR_040038	uncharacterized LOC151475	-2.0918717	down
BEND4	NM_207406	BEN domain containing 4	-2.7898242	down
	M15530		-3.8116748	down
B4GALNT1	NM_001478	beta-1,4-N-acetyl-galactosaminyl transferase 1	-4.1517744	down
LOC100131107	XM_001715471	UPF0607 protein ENSP00000383783-like	-2.6409407	down
INTU	NM_015693	inturned planar cell polarity effector homolog (Drosophila)	-2.0360618	down
GPT	NM_005309	glutamic-pyruvate transaminase (alanine aminotransferase)	-12.025615	down
POF1B	NM_024921	premature ovarian failure, 1B	-3.9651456	down
	XR_109175		-11.882804	down
FGF22	NM_020637	fibroblast growth factor 22	-2.0708148	down
HEATR4	NM_203309	HEAT repeat containing 4	-2.7644792	down
OR10R3P	AK122667	olfactory receptor, family 10, subfamily R, member 3 pseudogene	-2.766414	down
OR2M7	NM_001004691	olfactory receptor, family 2, subfamily M, member 7	-3.0194101	down
EMX1	BC037242	empty spiracles homeobox 1	-2.7129996	down
MST1P9	NR_002729	macrophage stimulating 1 (hepatocyte growth factor-like) pseudogene 9	-3.6798	down
RGMA	NM_020211	RGM domain family, member A	-2.797557	down
LHX1	NM_005568	LIM homeobox 1	-16.453512	down
			-3.8611047	down
CSDC2	NM_014460	cold shock domain containing C2, RNA binding	-2.2499437	down
GMNC	NM_001146686	geminin coiled-coil domain containing	-2.2894156	down
LOC100287314	NR_040245	uncharacterized LOC100287314	-2.8990226	down
GNN	NR_027249	Grp94 neighboring nucleotidase pseudogene	-3.0765047	down
	AK130724		-3.0144577	down
ZSCAN30	AK131291	zinc finger and SCAN domain containing 30	-2.432667	down
	AK130932		-2.50488	down
	DA666023		-5.5206895	down
PAX8	NM_003466	paired box 8	-2.3970957	down
IGFN1	NM_001164586	immunoglobulin-like and fibronectin type III domain containing 1	-2.936139	down
SNORA60	BF304636	small nucleolar RNA, H/ACA box 60	-24.101852	down
TAB3	AL832071	TGF-beta activated kinase 1/MAP3K7 binding protein 3	-2.0841086	down

DNAI1	NM_012144	dynein, axonemal, intermediate chain 1	-2.885911	down
DMTF1	NR_024549	cyclin D binding myb-like transcription factor 1	-2.3297913	down
OR2T6	NM_001005471	olfactory receptor, family 2, subfamily T, member 6	-2.9359596	down
			-2.2367468	down
	AK093659		-2.3107414	down
EYA2	NM_005244	eyes absent homolog 2 (Drosophila)	-3.1323988	down
APOA1	NM_000039	apolipoprotein A-I	-2.5555198	down
RNU105B	NR_004386	RNA, U105B small nucleolar	-2.052073	down
	XM_003118494		-2.7944076	down
GOLGA8A	NM_181077	golgin A8 family, member A	-3.1745539	down
ARHGEF26	NM_015595	Rho guanine nucleotide exchange factor (GEF) 26	-2.2591317	down
			-2.0300515	down
GOLT1A	NM_198447	golgi transport 1A	-2.2550058	down
LOC402160	NM_001193282	uncharacterized LOC402160	-2.94199	down
ZBTB8B	NM_001145720	zinc finger and BTB domain containing 8B	-2.5661407	down
MAGI2	NM_012301	membrane associated guanylate kinase, WW and PDZ domain containing 2	-2.0878015	down
MIR17HG	NR_027350	miR-17-92 cluster host gene (non-protein coding)	-4.278801	down
FAM166A	AK097419	family with sequence similarity 166, member A	-3.26692	down
NLRP7	NM_139176	NLR family, pyrin domain containing 7	-2.6873498	down
C1orf61	BC054045	chromosome 1 open reading frame 61	-4.1522026	down
ARHGAP23	NM_001199417	Rho GTPase activating protein 23	-2.0313954	down
CDHR3	NM_152750	cadherin-related family member 3	-2.9512844	down
OR1J1	NM_001004451	olfactory receptor, family 1, subfamily J, member 1	-2.8675408	down
PSG11	NM_002785	pregnancy specific beta-1-glycoprotein 11	-2.0961835	down
RREB1	NM_001003699	ras responsive element binding protein 1	-2.9915798	down
			-4.0586104	down
			-3.7115092	down
LOC100508383	XM_003119910	uncharacterized LOC100508383	-2.0231652	down
MACROD2	BC035876	MACRO domain containing 2	-3.7093298	down
LOC442132	NR_033906	golgin A6 family-like 1 pseudogene	-2.6582966	down
			-4.7340984	down
PITX2	NM_153426	paired-like homeodomain 2	-3.0472758	down
			-3.4793699	down
LOC100652742	XR_132763	uncharacterized LOC100652742	-4.4878373	down
LHX9	NM_020204	LIM homeobox 9	-3.2342193	down
GPR179	NM_001004334	G protein-coupled receptor 179	-3.5796883	down
TPH2	NM_173353	tryptophan hydroxylase 2	-7.6923847	down
MAPK15	NM_139021	mitogen-activated protein kinase 15	-2.5821118	down
MOB3B	NM_024761	MOB kinase activator 3B	-3.4244726	down
GPR98	NM_032119	G protein-coupled receptor 98	-4.3317847	down

AQP4	NM_001650	aquaporin 4	-3.0286992	down
MPL	NM_005373	myeloproliferative leukemia virus oncogene	-2.378023	down
FBLN1	NM_001996	fibulin 1	-6.499015	down
			-2.2055886	down
PPP1R27	NM_001007533	protein phosphatase 1, regulatory subunit 27	-5.269405	down
			-2.184193	down
LOC286442	NR_038968	uncharacterized LOC286442	-2.328914	down
SPEN	NM_015001	spen homolog, transcriptional regulator (Drosophila)	-5.6074247	down
			-2.3358502	down
FAM75A3	NM_001083124	family with sequence similarity 75, member A3	-3.3604574	down
OR4C6	NM_001004704	olfactory receptor, family 4, subfamily C, member 6	-11.45397	down
FBXW10	NM_031456	F-box and WD repeat domain containing 10	-4.3554344	down
			-3.2773993	down
			-4.1436296	down
CACNA1B	NM_000718	calcium channel, voltage-dependent, N type, alpha 1B subunit	-4.9678426	down
RNASE12	NM_001024822	ribonuclease, RNase A family, 12 (non-active)	-2.7973037	down
APC	NM_001127511	adenomatous polyposis coli	-2.182364	down
	CR625008		-4.1678476	down
SPON2	AK124606	spondin 2, extracellular matrix protein	-2.3445394	down
			-3.3159459	down
LOC731932	XM_001716667	uncharacterized LOC731932	-3.8089678	down
RGS8	NM_033345	regulator of G-protein signaling 8	-2.525994	down
			-2.0902019	down
LOC100130587	AK127527	uncharacterized LOC100130587	-2.3164892	down
LOC646482	BC015677	uncharacterized LOC646482	-2.8813436	down
RAP1GAP	NM_002885	RAP1 GTPase activating protein	-3.1807668	down
LOC100130539	XM_001724322	uncharacterized LOC100130539	-2.0935957	down
			-2.2228973	down
KCNJ14	NM_170720	potassium inwardly-rectifying channel, subfamily J, member 14	-2.4246068	down
ENTPD1	NM_001776	ectonucleoside triphosphate diphosphohydrolase 1	-2.13871	down
LOC286177	NR_038874	uncharacterized LOC286177	-2.1548452	down
			-2.7265215	down
FSHR	NM_000145	follicle stimulating hormone receptor	-2.267113	down
GPM6B	NM_001001995	glycoprotein M6B	-2.3533	down
ARHGAP23	NM_001199417	Rho GTPase activating protein 23	-3.3287895	down
			-3.0666175	down
			-3.3255405	down
PGM5	NM_021965	phosphoglucomutase 5	-2.2664988	down
KRT19P2	NR_036685	keratin 19 pseudogene 2	-2.1444669	down
CBX2	NM_032647	chromobox homolog 2	-2.165092	down
CERCAM	NM_016174	cerebral endothelial cell adhesion molecule	-2.907789	down

KAAG1	NM_181337	kidney associated antigen 1	-2.9201388	down
FBXL7	NM_012304	F-box and leucine-rich repeat protein 7	-2.2919905	down
LOC100130581	AK127427	uncharacterized LOC100130581	-2.4430544	down
ULBP2	NM_025217	UL16 binding protein 2	-3.1869807	down
SERPINA4	NM_006215	serpin peptidase inhibitor, clade A (alpha-1 antiproteinase, antitrypsin), member 4	-3.599843	down
ELK1	NM_001114123	ELK1, member of ETS oncogene family	-8.91259	down
ANKRD20A8P	NR_003366	ankyrin repeat domain 20 family, member A8, pseudogene	-4.333573	down
RCE1	NM_005133	RCE1 homolog, prenyl protein peptidase (S. cerevisiae)	-2.8645515	down
			-2.201137	down
PIP5K1A	NM_003557	phosphatidylinositol-4-phosphate 5-kinase, type I, alpha	-4.3902464	down
			-2.8960779	down
SPTBN4	NM_020971	spectrin, beta, non-erythrocytic 4	-3.9215827	down
			-3.4587607	down
DCD	NM_053283	dermcidin	-2.4074175	down
HHIP	NM_022475	hedgehog interacting protein	-3.1273031	down
SHC3	NM_016848	SHC (Src homology 2 domain containing) transforming protein 3	-2.0452006	down
CCDC108	NM_152389	coiled-coil domain containing 108	-2.321727	down
LOC283731	BC050067	uncharacterized LOC283731	-3.443976	down
TSGA10	NM_025244	testis specific, 10	-2.2358823	down
SLC25A10	NM_012140	solute carrier family 25 (mitochondrial carrier; dicarboxylate transporter), member 10	-2.608613	down
LINC00281	NR_027278	long intergenic non-protein coding RNA 281	-3.6202443	down
EXTL1	NM_004455	exostoses (multiple)-like 1	-4.033939	down
FAM22A	NM_001099338	family with sequence similarity 22, member A	-2.6953962	down
SOX3	NM_005634	SRY (sex determining region Y)-box 3	-3.300588	down
			-2.9207594	down
PHLDB3	AK097512	pleckstrin homology-like domain, family B, member 3	-2.2092724	down
			-3.443294	down
USH1C	NM_005709	Usher syndrome 1C (autosomal recessive, severe)	-2.880904	down
	AL117481		-2.611336	down
	AK123118		-6.6710963	down
ZNF331		zinc finger protein 331	-3.0107036	down
LRRC3	NM_030891	leucine rich repeat containing 3	-2.853224	down
PDGFRA	NM_006206	platelet-derived growth factor receptor, alpha polypeptide	-2.2322545	down
DPF3	AK024141	D4, zinc and double PHD fingers, family 3	-2.080736	down
FLJ38379	XR_108456	uncharacterized FLJ38379	-2.0375452	down
FAM27A	NR_024060	family with sequence similarity 27, member A	-4.4144225	down
			-2.9267063	down
			-2.0754888	down
	BC041650		-3.5388305	down
PRAMEF22	NM_001100631	PRAME family member 22	-2.1726177	down

NFASC	NM_001005388	neurofascin	-6.7971063	down
	AK022609		-2.005248	down
			-2.634412	down
			-2.4412186	down
	AK123308		-2.1073499	down
	AK056600		-2.2421494	down
	CU678159		-3.3637216	down
LOC441204	NR_015364	uncharacterized LOC441204	-3.853323	down
ABCB9	NM_019625	ATP-binding cassette, sub-family B (MDR/TAP), member 9	-2.612492	down
EML5	NM_183387	echinoderm microtubule associated protein like 5	-9.253154	down
LOC200726	NM_001102659	hCG1657980	-5.9616265	down
			-2.2873392	down
BSX	NM_001098169	brain-specific homeobox	-2.4991012	down
HSPA8		heat shock 70kDa protein 8	-3.1697426	down
CASKIN1	NM_020764	CASK interacting protein 1	-2.277006	down
DGCR5	NR_002733	DiGeorge syndrome critical region gene 5 (non-protein coding)	-2.143334	down
NEUROG1	NM_006161	neurogenin 1	-3.1397324	down
KIRREL3-AS3	NR_040078	KIRREL3 antisense RNA 3 (non-protein coding)	-5.0667768	down
			-2.6659617	down
DLK1	NM_003836	delta-like 1 homolog (Drosophila)	-2.7227533	down
RAPH1	NM_213589	Ras association (RalGDS/AF-6) and pleckstrin homology domains 1	-2.3589814	down
PHLDA3	NM_012396	pleckstrin homology-like domain, family A, member 3	-2.6204505	down
CIITA	NM_000246	class II, major histocompatibility complex, transactivator	-2.8290663	down
			-4.9868937	down
ZC3H11A	BX648271	zinc finger CCCH-type containing 11A	-3.0538406	down
NTRK2	NM_006180	neurotrophic tyrosine kinase, receptor, type 2	-2.1302247	down
PAX5	NM_016734	paired box 5	-2.3719497	down
LOC116437	NR_026670	uncharacterized LOC116437	-4.1206765	down
NAT8L	NM_178557	N-acetyltransferase 8-like (GCN5-related, putative)	-2.6935322	down
GLRA1	NM_000171	glycine receptor, alpha 1	-2.484067	down
RNU105C	NR_004385	RNA, U105C small nucleolar	-7.9807844	down
KIF6	NM_145027	kinesin family member 6	-2.2739236	down
AZGP1	NM_001185	alpha-2-glycoprotein 1, zinc-binding	-2.3737552	down
IKZF2	NM_001079526	IKAROS family zinc finger 2 (Helios)	-2.5392673	down
			-3.2232668	down
			-2.107883	down
C1orf95	NM_001003665	chromosome 1 open reading frame 95	-2.5306077	down
CHEK2	NM_145862	checkpoint kinase 2	-2.01323	down
			-3.9305325	down
SCN8A	NM_014191	sodium channel, voltage gated, type VIII, alpha subunit	-2.4663725	down
FLJ44054	NR_024609	uncharacterized LOC643365	-2.4761572	down

C6orf138	NM_001013732	chromosome 6 open reading frame 138	-2.5933256	down
			-4.5568705	down
PDE4DIP	NM_001198832	phosphodiesterase 4D interacting protein	-5.6383185	down
LOC401188	AK091013	uncharacterized LOC401188	-2.4214504	down
GOLGA6L10	NM_001164465	golgin A6 family-like 10	-7.4029164	down
CEP55	NM_018131	centrosomal protein 55kDa	-2.0650635	down
SH3GL1P2	NR_033420	SH3-domain GRB2-like 1 pseudogene 2	-4.760231	down
MYO18B	NM_032608	myosin XVIIIB	-2.263577	down
	XM_003119553		-2.3404934	down
			-2.827184	down
			-3.1189828	down
AACSP1	NR_024035	acetoacetyl-CoA synthetase pseudogene 1	-8.845006	down
			-2.2812047	down
POM121L9P	NR_003714	POM121 membrane glycoprotein-like 9, pseudogene	-3.0508938	down
FLJ16779	NR_024389	uncharacterized LOC100192386	-2.0264542	down
GALNT5	NM_014568	UDP-N-acetyl-alpha-D-galactosamine:polypeptide N-acetylgalactosaminyltransferase 5 (GalNAc-T5)	-3.2798953	down
	AK075182		-2.2564356	down
ANKRD20A4	XM_003403822	ankyrin repeat domain 20 family, member A4	-5.3436694	down
LOC441179	XR_112948	uncharacterized LOC441179	-4.5668626	down
C19orf44	NM_032207	chromosome 19 open reading frame 44	-2.042961	down
			-2.1753078	down
MPP3	NM_001932	membrane protein, palmitoylated 3 (MAGUK p55 subfamily member 3)	-3.2927377	down
SLC38A8	NM_001080442	solute carrier family 38, member 8	-2.4864526	down
MYADML2	NM_001145113	myeloid-associated differentiation marker-like 2	-2.3721561	down
TBC1D3B	NM_001001417	TBC1 domain family, member 3B	-2.961522	down
	AF068294		-3.0097098	down
NPB	NM_148896	neuropeptide B	-2.6334124	down
RUNX1	NM_001122607	runt-related transcription factor 1	-4.8424187	down
			-3.8398585	down
GLB1L3	NM_001080407	galactosidase, beta 1-like 3	-2.0269883	down
LOC143286	AL049428	uncharacterized LOC143286	-3.7336493	down
	AF495723		-3.4110782	down
	BC039440		-4.5986185	down
			-2.2632632	down
LINC00111	NR_024367	long intergenic non-protein coding RNA 111	-6.9616804	down
LOC644246	NR_034172	uncharacterized LOC644246	-2.919609	down
MCART6	NM_001012755	mitochondrial carrier triple repeat 6	-2.0241315	down
BSPRY	NM_017688	B-box and SPRY domain containing	-2.912585	down
NFATC2	NM_173091	nuclear factor of activated T-cells, cytoplasmic, calcineurin-dependent 2	-3.1096048	down
	AF116680		-3.4316304	down

MGC15705	BC007304	uncharacterized protein MGC15705	-2.3447604	down
DAZ1	NM_004081	deleted in azoospermia 1	-2.696044	down
BAALC		brain and acute leukemia, cytoplasmic	-2.5718036	down
			-2.9526112	down
			-2.9310122	down
			-3.3041184	down
PRSS47	XR_108978	protease, serine, 47	-2.9740458	down
LOC100130278	AK094114	uncharacterized LOC100130278	-2.6810272	down
MYLK3	NM_182493	myosin light chain kinase 3	-2.6045861	down
	CD674797		-2.2103498	down
KCNH6	NM_173092	potassium voltage-gated channel, subfamily H (eag-related), member 6	-3.789919	down
C7orf31	NM_138811	chromosome 7 open reading frame 31	-2.5165324	down
LOC441268	AK125166	uncharacterized LOC441268	-2.662963	down
ASB15	NM_080928	ankyrin repeat and SOCS box containing 15	-2.018217	down
LOC100128501	AK092192	uncharacterized LOC100128501	-2.9409704	down
TWIST2	BC103755	twist homolog 2 (Drosophila)	-2.13133	down
CHN2	AK294216	chimerin (chimaerin) 2	-2.1265008	down
OPRL1	NM_182647	opiate receptor-like 1	-3.7429655	down
	CR597597		-2.713801	down
FAM83F	NM_138435	family with sequence similarity 83, member F	-2.2759414	down
TRIM11	AK074623	tripartite motif containing 11	-2.0618768	down
LOC389033	NR_026740	placenta-specific 9 pseudogene	-3.116613	down
PANX2	NM_052839	pannexin 2	-3.5234835	down
LOC100129148	NR_033999	uncharacterized LOC100129148	-3.8475778	down
VPS37D	NM_001077621	vacuolar protein sorting 37 homolog D (S. cerevisiae)	-2.6878357	down
ZC3H12D	NM_207360	zinc finger CCCH-type containing 12D	-6.3925047	down
LOC440346	BC035163	uncharacterized LOC440346	-2.5741751	down
GPR133	NM_198827	G protein-coupled receptor 133	-3.547708	down
			-2.664155	down
NCAPG	NM_022346	non-SMC condensin I complex, subunit G	-2.4537015	down
NTNG2	AY358165	netrin G2	-6.8432245	down
HS1BP3	BC057389	HCLS1 binding protein 3	-3.9806798	down
	XM_003119477		-2.4544106	down
FAM123A	NM_152704	family with sequence similarity 123A	-2.0918944	down
ADRA1B	NM_000679	adrenergic, alpha-1B-, receptor	-2.8089519	down
EXOC7	AK022397	exocyst complex component 7	-2.4090416	down
GJD4	BC035898	gap junction protein, delta 4, 40.1kDa	-6.991357	down
CRYBA2	NM_005209	crystallin, beta A2	-2.7679296	down
C22orf42	NM_001010859	chromosome 22 open reading frame 42	-3.8820403	down
LOC100128105	XM_001721678	uncharacterized LOC100128105	-2.01934	down
RUFY4	NM_198483	RUN and FYVE domain containing 4	-3.057439	down

CPNE1	NM_003915	copine I	-2.9326484	down
LOC285300	AK095655	uncharacterized LOC285300	-3.0888019	down
TSG1	NR_015362	tumor suppressor TSG1	-2.380016	down
			-4.532669	down
			-2.3408945	down
	AF130091		-2.0109074	down
REG3A	NM_138938	regenerating islet-derived 3 alpha	-2.559145	down
NPLOC4	AB040932	nuclear protein localization 4 homolog (S. cerevisiae)	-2.1306121	down
ONECUT3	NM_001080488	one cut homeobox 3	-3.3143384	down
PRSS8	NM_002773	protease, serine, 8	-4.0148997	down
C17orf105	NM_001136483	chromosome 17 open reading frame 105	-2.9773674	down
LYZL1	NM_032517	lysozyme-like 1	-4.044266	down
	AB305689		-2.2967505	down
ZNF682	NM_033196	zinc finger protein 682	-3.902928	down
TTLL6	NM_173623	tubulin tyrosine ligase-like family, member 6	-3.057029	down
CYP2C18	NM_000772	cytochrome P450, family 2, subfamily C, polypeptide 18	-2.4119344	down
	AK124325		-2.5298648	down
C2orf53	NM_178553	chromosome 2 open reading frame 53	-2.0435889	down
TSPY26P	NR_002781	testis specific protein, Y-linked 26, pseudogene	-2.7128167	down
			-2.9023128	down
POTEB	NM_207355	POTE ankyrin domain family, member B	-4.1299067	down
LARP4B	NM_015155	La ribonucleoprotein domain family, member 4B	-2.2183692	down
PROX2	NM_001243007	prospero homeobox 2	-5.0145664	down
	AF390550		-2.703168	down
FAM188B	AK090826	family with sequence similarity 188, member B	-2.0660553	down
FLJ42392	NR_033877	uncharacterized LOC400123	-2.267029	down
HMGB3P1	NR_002165	high mobility group box 3 pseudogene 1	-3.4973514	down
SPC24	AK075287	SPC24, NDC80 kinetochore complex component, homolog (S. cerevisiae)	-2.3197775	down
LOC646508	NM_001195187	family with sequence similarity 90, member A1-like	-3.0373304	down
MEGF11	NM_032445	multiple EGF-like-domains 11	-4.04248	down
ZP4	NM_021186	zona pellucida glycoprotein 4	-2.7163396	down
MAT1A	NM_000429	methionine adenosyltransferase I, alpha	-3.4326124	down
SH2D4B	NM_207372	SH2 domain containing 4B	-3.135691	down
GH1	NM_000515	growth hormone 1	-2.542353	down
ERO1LB		ERO1-like beta (S. cerevisiae)	-2.0460145	down
CYCSP52	NR_001560	cytochrome c, somatic pseudogene 52	-2.7928193	down
			-3.0524366	down
LOC729739	BC013681	peptidylprolyl isomerase A (cyclophilin A) pseudogene	-3.0480518	down
			-2.0485036	down
ARHGEF4	NM_015320	Rho guanine nucleotide exchange factor (GEF) 4	-2.0614185	down
MAGEC1	NM_005462	melanoma antigen family C, 1	-3.344572	down

			-2.0052109	down
			-3.5126047	down
LOC100130372	AK127532	uncharacterized LOC100130372	-2.0686522	down
LOC402779	NR_038372	uncharacterized LOC402779	-2.2035089	down
STAG2	NM_001042751	stromal antigen 2	-2.3683817	down
CALCA	NM_001033952	calcitonin-related polypeptide alpha	-2.9556026	down
LOC100131170	AK098403	uncharacterized LOC100131170	-3.704955	down
UCP3	NM_022803	uncoupling protein 3 (mitochondrial, proton carrier)	-3.1229603	down
S100A7A	AY189119	S100 calcium binding protein A7A	-3.0437043	down
TMEM99	NM_001195386	transmembrane protein 99	-2.2920058	down
SATB2	NM_015265	SATB homeobox 2	-2.0754857	down
			-2.615217	down
VSTM2L	NM_080607	V-set and transmembrane domain containing 2 like	-6.580755	down
LOC100128176	NR_038408	uncharacterized LOC100128176	-2.3471353	down
C4orf50	XM_003118524	chromosome 4 open reading frame 50	-3.3959832	down
			-3.8274066	down
			-8.7195015	down
MATN4	NM_003833	matrilin 4	-2.2093909	down
MYH7B	NM_020884	myosin, heavy chain 7B, cardiac muscle, beta	-2.909175	down
			-3.4789088	down
TMEM132C	NM_001136103	transmembrane protein 132C	-2.8326926	down
GPC6	NM_005708	glypican 6	-2.7232473	down
LOC100128950	AK124198	uncharacterized LOC100128950	-3.310135	down
			-8.349956	down
LOC729911	NR_038997	uncharacterized LOC729911	-3.3559012	down
C2orf16	NM_032266	chromosome 2 open reading frame 16	-7.9584894	down
STON2	NM_033104	stonin 2	-2.5440195	down
CEP72	NM_018140	centrosomal protein 72kDa	-2.883138	down
PLA2G2D	NM_012400	phospholipase A2, group IID	-2.4678793	down
MAGEA11	NM_001011544	melanoma antigen family A, 11	-2.2949226	down
	AJ276252		-2.196858	down
TRPM3	NM_206948	transient receptor potential cation channel, subfamily M, member 3	-4.561398	down
FICD	NM_007076	FIC domain containing	-3.2580085	down
LOC100132356	NR_034127	uncharacterized LOC100132356	-2.5004866	down
HTRA3	NM_053044	HtrA serine peptidase 3	-2.380205	down
			-4.2568326	down
BTBD19	NM_001136537	BTB (POZ) domain containing 19	-3.4949558	down
			-2.3657763	down
PRAMEF4	NM_001009611	PRAME family member 4	-5.2520337	down
LOC442366	XM_001714957	uncharacterized LOC442366	-2.0368047	down
FCER1G		Fc fragment of IgE, high affinity I, receptor for; gamma polypeptide	-2.0450554	down

OPRM1	NM_001008504	opioid receptor, mu 1	-2.3947942	down
FLJ13224	NR_026806	uncharacterized LOC79857	-2.504551	down
CELSR2	NM_001408	cadherin, EGF LAG seven-pass G-type receptor 2 (flamingo homolog, Drosophila)	-2.1288154	down
UBE2I		ubiquitin-conjugating enzyme E2I	-3.1578462	down
SLC5A7	NM_021815	solute carrier family 5 (choline transporter), member 7	-2.2359223	down
FLJ44674	AK128747	FLJ44674 protein	-2.497909	down
LOC100506485	XR_110000	uncharacterized LOC100506485	-2.507636	down
PCSK1N	NM_013271	proprotein convertase subtilisin/kexin type 1 inhibitor	-4.017537	down
			-2.2224689	down
OR52E2	NM_001005164	olfactory receptor, family 52, subfamily E, member 2	-12.850661	down
			-2.2948582	down
	AK128779		-3.1691606	down
			-2.450457	down
ANKRD30BL	NR_027019	ankyrin repeat domain 30B-like	-3.9171348	down
MGC72080	NR_002822	MGC72080 pseudogene	-2.4167578	down
C7orf54	NR_027330	chromosome 7 open reading frame 54	-5.7121572	down
ZNF295-AS1	NR_027273	ZNF295 antisense RNA 1 (non-protein coding)	-2.1244316	down
LOC284276	NR_015417	uncharacterized LOC284276	-2.149846	down
C9orf170	NM_001001709	chromosome 9 open reading frame 170	-2.194299	down
KRT18	NM_000224	keratin 18	-2.544059	down
LOC284939	AK094442	uncharacterized LOC284939	-2.5421364	down
	XR_110101		-2.663046	down
KRT24	NM_019016	keratin 24	-3.9864826	down
CBWD7	AK097639	COBW domain containing 7	-2.050406	down
			-3.2997575	down
IL23R	NM_144701	interleukin 23 receptor	-2.009516	down
LYPD1	NM_144586	LY6/PLAUR domain containing 1	-3.1973336	down
LOC100131257	NR_034022	zinc finger protein 655 pseudogene	-2.0795376	down
ZP3	NM_007155	zona pellucida glycoprotein 3 (sperm receptor)	-3.2205937	down
ELMOD3	NM_001135021	ELMO/CED-12 domain containing 3	-6.2250795	down
BBOX1	NM_003986	butyrobetaine (gamma), 2-oxoglutarate dioxygenase (gamma-butyrobetaine hydroxylase) 1	-2.249721	down
			-3.4104838	down
NRIP2	NM_031474	nuclear receptor interacting protein 2	-3.1240985	down
PLEKHH3	NM_024927	pleckstrin homology domain containing, family H (with MyTH4 domain) member 3	-6.09917	down
CAMKK1	NM_172207	calcium/calmodulin-dependent protein kinase kinase 1, alpha	-2.2565734	down
SPEG	NM_005876	SPEG complex locus	-4.6148615	down
ELOVL3	NM_152310	ELOVL fatty acid elongase 3	-2.1939065	down
CYP11B1	NM_000497	cytochrome P450, family 11, subfamily B, polypeptide 1	-4.0354156	down
ALDH3B2	NM_000695	aldehyde dehydrogenase 3 family, member B2	-2.7727742	down
			-2.5169075	down

LOC390660	AK131095	FLJ00317 protein	-2.0108864	down
			-2.4875305	down
LOC100130009	XM_001718914	high mobility group protein HMG-I/HMG-Y-like	-2.1222732	down
PRKCG	NM_002739	protein kinase C, gamma	-2.5764086	down
GRID2IP	NM_001145118	glutamate receptor, ionotropic, delta 2 (Grid2) interacting protein	-2.5105624	down
ALDH4A1	NM_003748	aldehyde dehydrogenase 4 family, member A1	-2.0557659	down
NEU2	NM_005383	sialidase 2 (cytosolic sialidase)	-2.152409	down
FLJ42200	AK124194	FLJ42200 protein	-2.5985951	down
PSG1	NM_006905	pregnancy specific beta-1-glycoprotein 1	-2.1910615	down
	CR745051		-3.053713	down
LINC00302	CU449054	long intergenic non-protein coding RNA 302	-2.149412	down
DKKL1	NM_014419	dickkopf-like 1	-2.145298	down
ACACB	NM_001093	acetyl-CoA carboxylase beta	-2.1251402	down
			-3.4992864	down
C1orf140	NR_024236	uncharacterized LOC400804	-5.214493	down
EMILIN3	NM_052846	elastin microfibril interfacer 3	-2.0795846	down
PPIAL4G	AK123006	peptidylprolyl isomerase A (cyclophilin A)-like 4G	-3.2630737	down
CLDN24	NM_001185149	claudin 24	-2.027146	down
EFHC2	NM_025184	EF-hand domain (C-terminal) containing 2	-2.1857426	down
	XR_109271		-3.1493418	down
C14orf41	AK055876	chromosome 14 open reading frame 41	-4.465653	down
ESYT3	NM_031913	extended synaptotagmin-like protein 3	-2.027996	down
ANKRD1	NM_014391	ankyrin repeat domain 1 (cardiac muscle)	-2.058246	down
	XM_001727011		-2.1015105	down
GJA8	NM_005267	gap junction protein, alpha 8, 50kDa	-2.649914	down
			-2.8283353	down
OR5AS1	NM_001001921	olfactory receptor, family 5, subfamily AS, member 1	-6.8833327	down
MGC12982	NR_026878	uncharacterized protein MGC12982	-2.1788409	down
PRAMEF15	NM_001098376	PRAME family member 15	-3.3328235	down
	DA797466		-3.3040235	down
EGFL6	NM_001167890	EGF-like-domain, multiple 6	-2.9807382	down
KALRN	AK131379	kalirin, RhoGEF kinase	-7.722789	down
HSD17B2	NM_002153	hydroxysteroid (17-beta) dehydrogenase 2	-2.275334	down
RS1	NM_000330	retinoschisin 1	-2.5032353	down
RAI1	NM_030665	retinoic acid induced 1	-2.3887513	down
TTLL13	NM_001029964	tubulin tyrosine ligase-like family, member 13	-2.9083288	down
ALPK1	AK026323	alpha-kinase 1	-2.2010942	down
SERPING1	NM_000062	serpin peptidase inhibitor, clade G (C1 inhibitor), member 1	-4.2205234	down
NXN	BC104634	nucleoredoxin	-2.1891823	down
C2orf27B	NM_214461	chromosome 2 open reading frame 27B	-3.303043	down
LOC284561	XR_112106	uncharacterized LOC284561	-4.23857	down

AOX1	NM_001159	aldehyde oxidase 1	-2.0797276	down
GPRIN1	NM_052899	G protein regulated inducer of neurite outgrowth 1	-2.8827758	down
			-2.142824	down
LOC100127946	XM_001717040	uncharacterized LOC100127946	-2.1503267	down
KNCN	NM_001097611	kinocilin	-2.725823	down
LOC100128682	NR_040046	uncharacterized LOC100128682	-2.454104	down
BTC	NM_001729	betacellulin	-2.8819747	down
MLNR	NM_001507	motilin receptor	-2.2022471	down
LINC00265	NR_026999	long intergenic non-protein coding RNA 265	-10.263503	down
FAM46B	NM_052943	family with sequence similarity 46, member B	-2.3069835	down
LOC100131242	AK124483	uncharacterized LOC100131242	-3.5512571	down
	CR627384		-2.2860222	down
C22orf24	NM_015372	chromosome 22 open reading frame 24	-3.5211573	down
SLC2A12	NM_145176	solute carrier family 2 (facilitated glucose transporter), member 12	-3.3910832	down
LONRF2	NM_198461	LON peptidase N-terminal domain and ring finger 2	-3.2625039	down
XAGE1A	NM_001097592	X antigen family, member 1A	-3.8157604	down
SLC35E2	NM_182838	solute carrier family 35, member E2	-2.1596966	down
			-3.477825	down
KISS1	NM_002256	KiSS-1 metastasis-suppressor	-2.3801632	down
LOC728613	NR_003713	programmed cell death 6 pseudogene	-4.9963565	down
SPATA13	AK092754	spermatogenesis associated 13	-3.1779885	down
LIPG	NM_006033	lipase, endothelial	-2.2022915	down
MATN1	NM_002379	matrilin 1, cartilage matrix protein	-2.150094	down
LOC283553	NR_038358	uncharacterized LOC283553	-2.2213464	down
LOC203274	BC110369	uncharacterized LOC203274	-2.9399838	down
			-2.74192	down
C6orf170	NM_152730	chromosome 6 open reading frame 170	-2.1800516	down
TNFAIP8L1	NM_001167942	tumor necrosis factor, alpha-induced protein 8-like 1	-2.8801103	down
ATOH7	NM_145178	atonal homolog 7 (Drosophila)	-3.0680246	down
LOC400706	XR_111876	uncharacterized LOC400706	-2.6725647	down
DHX30	NM_014966	DEAH (Asp-Glu-Ala-His) box polypeptide 30	-2.0170038	down
KRBA2	NM_213597	KRAB-A domain containing 2	-2.636628	down
			-3.1327965	down
	XR_111602		-2.1665819	down
LOC285758	NR_038863	uncharacterized LOC285758	-6.7411222	down
BEGAIN	NM_001159531	brain-enriched guanylate kinase-associated homolog (rat)	-2.8482418	down
IFITM5	NM_001025295	interferon induced transmembrane protein 5	-3.7229087	down
PDXDC2P	NR_003610	pyridoxal-dependent decarboxylase domain containing 2, pseudogene	-4.043652	down
			-4.615257	down
SRPX	NM_006307	sushi-repeat containing protein, X-linked	-2.3670316	down
			-2.0965304	down

C4orf38	NR_024008	chromosome 4 open reading frame 38	-2.3429992	down
HMBOX1		homeobox containing 1	-4.5809073	down
CDH24	NM_022478	cadherin 24, type 2	-8.422184	down
SIGLEC16	NR_002825	sialic acid binding Ig-like lectin 16 (gene/pseudogene)	-2.8121698	down
ICMT	BC017037	isoprenylcysteine carboxyl methyltransferase	-2.7361953	down
PRSS50	NM_013270	protease, serine, 50	-2.275281	down
			-2.977569	down
			-2.0973783	down
FRMD4A	AK001072	FERM domain containing 4A	-5.5562153	down
RYR2	NM_001035	ryanodine receptor 2 (cardiac)	-2.3227167	down
GPR114		G protein-coupled receptor 114	-2.4407423	down
	AF251048		-2.819039	down
ANKMY2	NM_020319	ankyrin repeat and MYND domain containing 2	-2.540638	down
UBAP2L	NM_014847	ubiquitin associated protein 2-like	-2.8346689	down
	XR_110878		-2.3498874	down
			-3.8640091	down
C5orf25	AK299336	chromosome 5 open reading frame 25	-2.1877909	down
PGC	NM_002630	progastricsin (pepsinogen C)	-3.787712	down
ANKRD33B	NM_001164440	ankyrin repeat domain 33B	-2.1035476	down
LOC392364	NR_040117	chromosome 15 open reading frame 2 pseudogene	-4.0242825	down
SH3BGRL3	NM_031286	SH3 domain binding glutamic acid-rich protein like 3	-2.6298208	down
TMSB4Y	NM_004202	thymosin beta 4, Y-linked	-2.4894464	down
			-4.641126	down
C19orf80	NM_018687	chromosome 19 open reading frame 80	-2.9006255	down
PRELID2	NM_138492	PRELI domain containing 2	-2.567545	down
AQP2	NM_000486	aquaporin 2 (collecting duct)	-2.2700171	down
C21orf116	AF130090	chromosome 21 open reading frame 116	-2.12819	down
	AK002210		-2.419726	down
PARP10	NM_032789	poly (ADP-ribose) polymerase family, member 10	-3.0117638	down
LOC728558	NR_038444	uncharacterized LOC728558	-2.0298798	down
	DA967691		-3.8911455	down
C1orf95	NM_001003665	chromosome 1 open reading frame 95	-2.8651948	down
PLAC4	NM_182832	placenta-specific 4	-2.899971	down
MUC22	NM_001198815	mucin 22	-2.2997468	down
ASS1	NM_000050	argininosuccinate synthase 1	-2.3587267	down
FBXO44	NM_001014765	F-box protein 44	-2.8412106	down
HSPA12B	NM_052970	heat shock 70kD protein 12B	-6.8582325	down
MREG	NM_018000	melanoregulin	-2.5230293	down
AIM1L	NM_001039775	absent in melanoma 1-like	-2.0896115	down
CLDN9	NM_020982	claudin 9	-2.3201125	down
	AK055281		-6.856401	down

			-3.6521168	down
RARB	NM_000965	retinoic acid receptor, beta	-2.0805387	down
HOXA2	NM_006735	homeobox A2	-7.2102246	down
SCNN1D	NM_001130413	sodium channel, nonvoltage-gated 1, delta	-2.8304255	down
	AK126599		-2.2630346	down
			-2.512548	down
ADSSL1	NM_199165	adenylosuccinate synthase like 1	-2.6370478	down
ADH4	NM_000670	alcohol dehydrogenase 4 (class II), pi polypeptide	-5.839934	down
LOC153811	AK021734	uncharacterized LOC153811	-2.077881	down
ZNF750	NM_024702	zinc finger protein 750	-2.2620246	down
GDA	NM_004293	guanine deaminase	-2.3238697	down
DKFZP547J0410	AL050263	DKFZP547J0410 protein	-2.1034982	down
ANKRD20A5P	NR_040113	ankyrin repeat domain 20 family, member A5, pseudogene	-3.301266	down
LOC100132474	AK125160	uncharacterized LOC100132474	-4.3550954	down
			-2.1240373	down
TEKT5	NM_144674	tektin 5	-2.1575022	down
	AK055264		-4.298498	down
CYTH1	AK123894	cytohesin 1	-3.669139	down
C14orf23	NR_026731	chromosome 14 open reading frame 23	-2.0129092	down
			-3.1985557	down
NPHS2	NM_014625	nephrosis 2, idiopathic, steroid-resistant (podocin)	-2.0273662	down
PLCE1	NM_016341	phospholipase C, epsilon 1	-4.449349	down
			-2.488993	down
			-3.2373679	down
HOXD3	NM_006898	homeobox D3	-4.82493	down
C16orf78	NM_144602	chromosome 16 open reading frame 78	-2.8015893	down
			-3.0303025	down
	XR_133518		-6.628003	down
	AK130638		-3.8447871	down
TRIM49	NM_020358	tripartite motif containing 49	-2.952903	down
CARD10	NM_014550	caspase recruitment domain family, member 10	-2.5354111	down
CDK20	NM_001039803	cyclin-dependent kinase 20	-2.9887044	down
ITGB1BP2	NM_012278	integrin beta 1 binding protein (melusin) 2	-3.6831837	down
HILS1	NR_024193	histone linker H1 domain, spermatid-specific 1	-2.1705287	down
MTHFR	NM_005957	methylenetetrahydrofolate reductase (NAD(P)H)	-2.239424	down
	AK096952		-4.0018263	down
FIGNL2	NM_001013690	fidgetin-like 2	-7.0376697	down
FLJ16126	XR_109873	uncharacterized LOC645010	-2.6852047	down
	AK094078		-2.536136	down
			-2.242781	down

DDX51	NM_175066	DEAD (Asp-Glu-Ala-Asp) box polypeptide 51	-2.1429496	down
	AK093172		-2.1346881	down
			-3.0330968	down
FLJ45079	NR_028337	FLJ45079 protein	-2.9329846	down
FAM188B	NM_032222	family with sequence similarity 188, member B	-2.7789786	down
			-2.2357159	down
TTC9B	NM_152479	tetratricopeptide repeat domain 9B	-5.811311	down
	XM_003403546		-2.5914798	down
BAALC		brain and acute leukemia, cytoplasmic	-3.4983406	down
GJB1	NM_000166	gap junction protein, beta 1, 32kDa	-2.311607	down
	AK129520		-3.1529033	down
LOC255177	BC031230	uncharacterized LOC255177	-5.7813587	down
CACNG7	NM_031896	calcium channel, voltage-dependent, gamma subunit 7	-2.189293	down
SEC14L3	NM_174975	SEC14-like 3 (S. cerevisiae)	-2.3337786	down
			-2.272156	down
AQP7P1	NR_002817	aquaporin 7 pseudogene 1	-5.191415	down
			-4.2666173	down
			-2.1274254	down
EVPLL	NM_001145127	envoplakin-like	-2.7900255	down
MYB	AJ616798	v-myb myeloblastosis viral oncogene homolog (avian)	-2.402128	down
	AY043127		-3.5069249	down
CYP3A7	NM_000765	cytochrome P450, family 3, subfamily A, polypeptide 7	-2.7026408	down
SLC4A11	NM_032034	solute carrier family 4, sodium borate transporter, member 11	-3.8291397	down
DDX11	NM_152438	DEAD/H (Asp-Glu-Ala-Asp/His) box polypeptide 11	-2.8795123	down
			-6.0088816	down
TNR	NM_003285	tenascin R (restrictin, janusin)	-3.688181	down
CDH26	AF169690	cadherin 26	-2.5530965	down
WEE2	NM_001105558	WEE1 homolog 2 (S. pombe)	-2.0671835	down
LOC283140	AK095275	uncharacterized LOC283140	-2.5002172	down
STARD13	AY082592	StAR-related lipid transfer (START) domain containing 13	-6.3124475	down
KRTAP22-1	BC101686	keratin associated protein 22-1	-2.193338	down
HIP1	AY358103	huntingtin interacting protein 1	-4.580367	down
SPINT3	NM_006652	serine peptidase inhibitor, Kunitz type, 3	-2.1773279	down
TRAIP	NM_005879	TRAF interacting protein	-2.61517	down
			-2.0892658	down
MSLNL	NM_001025190	mesothelin-like	-2.3094718	down
			-4.216011	down
			-3.1852612	down
RSG1	NM_030907	REM2 and RAB-like small GTPase 1	-2.7162266	down
KATNAL2	NM_031303	katanin p60 subunit A-like 2	-3.7840579	down
LRRC27	NM_001143757	leucine rich repeat containing 27	-2.0007784	down

			-3.4921243	down
CASC5	NM_170589	cancer susceptibility candidate 5	-2.6278677	down
LOC100287326	XM_002343311	uncharacterized LOC100287326	-2.037475	down
	BC007984		-3.7753386	down
SLC6A7	NM_014228	solute carrier family 6 (neurotransmitter transporter, L-proline), member 7	-2.0562637	down
LOC284628	AK094692	uncharacterized LOC284628	-2.0210927	down
			-2.4951174	down
ZBTB46	NM_025224	zinc finger and BTB domain containing 46	-2.4187608	down
OR4P4	NM_001004124	olfactory receptor, family 4, subfamily P, member 4	-2.0618207	down
BPIFA1	NM_130852	BPI fold containing family A, member 1	-2.1941106	down
			-2.262546	down
			-2.0420282	down
ARHGAP33	AY044864	Rho GTPase activating protein 33	-3.8457866	down
			-2.1764355	down
JSRP1	NM_144616	junctional sarcoplasmic reticulum protein 1	-2.1416838	down
KRTAP21-1	NM_181619	keratin associated protein 21-1	-2.2723818	down
DCDC2B	NM_001099434	doublecortin domain containing 2B	-3.0515325	down
			-2.458312	down
LANCL3	NM_198511	LanC lantibiotic synthetase component C-like 3 (bacterial)	-3.9324112	down
			-2.147342	down
POTEA	NM_001005365	POTE ankyrin domain family, member A	-2.153182	down
PPP1R12B	NM_002481	protein phosphatase 1, regulatory subunit 12B	-2.9945374	down
FXYD5	AF177940	FXYD domain containing ion transport regulator 5	-3.351646	down
ZNF833P	NR_028594	zinc finger protein 833, pseudogene	-3.2071152	down
			-3.576794	down
CD70	NM_001252	CD70 molecule	-2.6214747	down
LOC254128	NR_037857	uncharacterized LOC254128	-2.4693296	down
FUT5	NM_002034	fucosyltransferase 5 (alpha (1,3) fucosyltransferase)	-5.0115747	down
LOC440910	NR_030728	uncharacterized LOC440910	-2.178331	down
C22orf36	NM_207644	chromosome 22 open reading frame 36	-5.2006183	down
LOC100130581	AK127427	uncharacterized LOC100130581	-2.6120749	down
	XM_003118969		-2.0362318	down
MIR137HG	AK092728	MIR137 host gene (non-protein coding)	-3.9423654	down
FUT7	NM_004479	fucosyltransferase 7 (alpha (1,3) fucosyltransferase)	-2.336332	down
RDH8	NM_015725	retinol dehydrogenase 8 (all-trans)	-2.9215565	down
RBP5	NM_031491	retinol binding protein 5, cellular	-3.0047588	down
			-3.5357096	down
ASPN	NM_001193335	asporin	-3.1003358	down
OSBPL1A	NM_080597	oxysterol binding protein-like 1A	-2.3265214	down
FILIP1	NM_015687	filamin A interacting protein 1	-4.686489	down

C8G	NM_000606	complement component 8, gamma polypeptide	-2.109857	down
CLCN2	NM_004366	chloride channel 2	-3.5596416	down
LOC100128946	NR_038944	uncharacterized LOC100128946	-2.0029652	down
TUBB3	NM_006086	tubulin, beta 3 class III	-2.9997253	down
PNPLA5	NM_138814	patatin-like phospholipase domain containing 5	-2.1336887	down
			-2.929605	down
SSC5D	NM_001144950	scavenger receptor cysteine rich domain containing (5 domains)	-4.202606	down
			-2.1304648	down
PDE1C	NM_005020	phosphodiesterase 1C, calmodulin-dependent 70kDa	-2.0257459	down
C10orf57	NM_025125	chromosome 10 open reading frame 57	-2.027727	down
STAR	NM_000349	steroidogenic acute regulatory protein	-2.6234725	down
ZNF496	NM_032752	zinc finger protein 496	-2.1253622	down
KCNK17	NM_031460	potassium channel, subfamily K, member 17	-2.324354	down
RASGEF1C	NM_175062	RasGEF domain family, member 1C	-2.5676343	down
LINC00479	NR_027272	long intergenic non-protein coding RNA 479	-4.0778666	down
	AK127451		-2.596568	down
LOC730755	NM_001165252	keratin associated protein 2-4-like	-2.2337527	down
AKR1B15	NM_001080538	aldo-keto reductase family 1, member B15	-2.1337974	down
ADAMTS16	NM_139056	ADAM metallopeptidase with thrombospondin type 1 motif, 16	-3.2559235	down
RABEPK	AL832249	Rab9 effector protein with kelch motifs	-2.7702084	down
SLC4A10	NM_022058	solute carrier family 4, sodium bicarbonate transporter, member 10	-2.6917078	down
OXCT2	NM_022120	3-oxoacid CoA transferase 2	-2.0726116	down
FAM101A	NM_181709	family with sequence similarity 101, member A	-5.0124717	down
AGSK1	AK026448	golgin subfamily A member 2-like	-2.4807687	down
TRIP13	NM_004237	thyroid hormone receptor interactor 13	-2.0441926	down
MYO5A	NM_000259	myosin VA (heavy chain 12, myoxin)	-2.0242338	down
FAM184A	BX640728	family with sequence similarity 184, member A	-2.818335	down
SLC4A8	AK128321	solute carrier family 4, sodium bicarbonate cotransporter, member 8	-2.0290713	down
			-6.231032	down
MCF2L	NM_024979	MCF.2 cell line derived transforming sequence-like	-2.4111705	down
	AK130019		-2.4094837	down
			-2.2229578	down
OBSCN	NM_052843	obscurin, cytoskeletal calmodulin and titin-interacting RhoGEF	-2.9657788	down
			-2.9328835	down
			-7.210703	down
			-2.40387	down
PRSS38	NM_183062	protease, serine, 38	-4.308838	down
ZDHHC20	BC034944	zinc finger, DHHC-type containing 20	-2.653478	down
OR5I1	NM_006637	olfactory receptor, family 5, subfamily I, member 1	-3.0074112	down
INE1	NR_024616	inactivation escape 1 (non-protein coding)	-2.4841604	down
			-2.0379684	down

DRD2	NM_000795	dopamine receptor D2	-2.098226	down
MGAM		maltase-glucoamylase (alpha-glucosidase)	-2.9981947	down
LOC643327	AK096549	uncharacterized LOC643327	-2.3396132	down
CHST9-AS1	NR_026908	CHST9 antisense RNA 1 (non-protein coding)	-2.2112362	down
HOXB13	NM_006361	homeobox B13	-3.7711306	down
	AK126601		-3.1251743	down
LOC400752	NR_024270	uncharacterized LOC400752	-2.4061265	down
LOC100131043	AK124222	uncharacterized LOC100131043	-3.286456	down
IMMT	NM_001100170	inner membrane protein, mitochondrial	-2.6078925	down
SCNN1G	NM_001039	sodium channel, nonvoltage-gated 1, gamma	-2.1228693	down
PRKXP1	AI016765	protein kinase, X-linked, pseudogene 1	-3.530172	down
GFM1	AK022724	G elongation factor, mitochondrial 1	-3.4136693	down
TEAD3	NM_003214	TEA domain family member 3	-2.3171246	down
	AK098012		-2.547695	down
CNGA4	NM_001037329	cyclic nucleotide gated channel alpha 4	-2.4353645	down
C19orf47	NM_178830	chromosome 19 open reading frame 47	-2.2270744	down
TTTY5	NR_001541	testis-specific transcript, Y-linked 5 (non-protein coding)	-3.821448	down
NEK11	NM_145910	NIMA (never in mitosis gene a)- related kinase 11	-2.245152	down
HSP90AB4P	NR_002927	heat shock protein 90kDa alpha (cytosolic), class B member 4, pseudogene	-2.056503	down
IL26	NM_018402	interleukin 26	-5.722428	down
FAM197Y2P	NR_001553	family with sequence similarity 197, Y-linked, member 2, pseudogene	-2.8376663	down
FLJ41455	AK123449	uncharacterized LOC441441	-3.128099	down
DIO2	NM_013989	deiodinase, iodothyronine, type II	-2.5414531	down
AVPR1B	NM_000707	arginine vasopressin receptor 1B	-3.216929	down
FBXL18	NM_024963	F-box and leucine-rich repeat protein 18	-2.1857722	down
	CR605662		-2.1526978	down
TYMP	NM_001113755	thymidine phosphorylase	-2.5097065	down
	DB053889		-3.9369462	down
EBPL	BC073152	emopamil binding protein-like	-3.1389692	down
LOC100128001	AK125720	uncharacterized LOC100128001	-5.13357	down
	AK095132		-2.3890495	down
OR10G2	NM_001005466	olfactory receptor, family 10, subfamily G, member 2	-3.315201	down
KRT8P41	NR_027713	keratin 8 pseudogene 41	-2.871	down
MGC16275	NR_026914	uncharacterized protein MGC16275	-2.3972409	down
HES2	BC012091	hairy and enhancer of split 2 (Drosophila)	-2.0149393	down
NPPC	NM_024409	natriuretic peptide C	-2.2403631	down
LOC389831	NM_001242480	uncharacterized LOC389831	-5.603441	down
			-2.6453834	down
LINC00478	NR_027790	long intergenic non-protein coding RNA 478	-2.1834679	down
TMEM177	AK311734	transmembrane protein 177	-2.816161	down

HSFX1	NM_016153	heat shock transcription factor family, X linked 1	-2.0969775	down
GOLGA6L9	NM_198181	golgin A6 family-like 9	-2.6304502	down
OR2L2	NM_001004686	olfactory receptor, family 2, subfamily L, member 2	-2.3532882	down
POFUT1	NM_172236	protein O-fucosyltransferase 1	-2.8667874	down
LOC126536	NR_026828	uncharacterized LOC126536	-2.3260567	down
			-2.3419116	down
PARP1		poly (ADP-ribose) polymerase 1	-2.5167377	down
REXO1L1	NM_172239	REX1, RNA exonuclease 1 homolog (S. cerevisiae)-like 1	-6.7877603	down
ZNF771	NM_016643	zinc finger protein 771	-2.7068307	down
			-3.81782	down
			-3.6396644	down
TRIM77P	NM_001146162	tripartite motif containing 77, pseudogene	-2.2468913	down
			-2.0192826	down
BK250D10.8	NR_024355	uncharacterized LOC339674	-2.6002984	down
ITPK1-AS1	NR_002808	ITPK1 antisense RNA 1 (non-protein coding)	-3.1966882	down
CHN2		chimerin (chimaerin) 2	-3.3470228	down
C19orf26		chromosome 19 open reading frame 26	-2.6392431	down
KRTAP26-1	NM_203405	keratin associated protein 26-1	-2.1406884	down
			-2.2138312	down
			-2.482147	down
STXBP5L	NM_014980	syntaxin binding protein 5-like	-2.0890753	down
LOC648044	XM_001131322	guanine nucleotide binding protein (G protein), gamma 12-like	-2.072355	down
			-5.4164205	down
LOC285103	BC036597	uncharacterized LOC285103	-3.2048664	down
ZNF503-AS2	NR_024421	ZNF503 antisense RNA 2 (non-protein coding)	-2.3035274	down
LOC650293	NM_001040071	seven transmembrane helix receptor	-6.540019	down
	BC040619		-5.4872065	down
TAF5L		TAF5-like RNA polymerase II, p300/CBP-associated factor (PCAF)-associated factor, 65kDa	-2.3891914	down
MGC39545	XR_110535	uncharacterized LOC403312	-2.1363657	down
B4GALNT2	NM_153446	beta-1,4-N-acetyl-galactosaminyl transferase 2	-2.0449708	down
			-2.1958797	down
POM121L8P	NR_024583	POM121 membrane glycoprotein-like 8 pseudogene	-7.818869	down
LOC100506816	XR_108400	uncharacterized LOC100506816	-2.01689	down
			-2.0610745	down
CTPS2	NM_001144002	CTP synthase II	-2.0952806	down
HEATR7B1	XM_001721240	HEAT repeat containing 7B1	-2.594823	down
AKAP1	NM_003488	A kinase (PRKA) anchor protein 1	-6.650966	down
			-3.730161	down
PAK3	NM_002578	p21 protein (Cdc42/Rac)-activated kinase 3	-3.365554	down
FBLN1	NM_006486	fibulin 1	-3.6865642	down

SPRR2E	NM_001024209	small proline-rich protein 2E	-2.9355419	down
			-2.0585797	down
C2CD4B	NM_001007595	C2 calcium-dependent domain containing 4B	-3.2585046	down
CHRNA1	NM_001039523	cholinergic receptor, nicotinic, alpha 1 (muscle)	-4.53592	down
CRHR1	NM_001145148	corticotropin releasing hormone receptor 1	-2.5290244	down
TJP2		tight junction protein 2 (zona occludens 2)	-2.354647	down
TAS2R30	NM_001097643	taste receptor, type 2, member 30	-2.4881923	down
VAX1	NM_001112704	ventral anterior homeobox 1	-3.3857303	down
			-5.241445	down
			-2.1355317	down
HTR3A	NM_213621	5-hydroxytryptamine (serotonin) receptor 3A	-2.1413627	down
C6orf164	NR_026784	chromosome 6 open reading frame 164	-2.4579763	down
ACAN	NM_001135	aggrecan	-3.8159482	down
BCAS1	NM_003657	breast carcinoma amplified sequence 1	-2.7943482	down
			-6.458739	down
LRRTM4	NM_024993	leucine rich repeat transmembrane neuronal 4	-2.0386956	down
	BC050023		-4.641401	down
LOC100128402	AK124574	uncharacterized LOC100128402	-3.4809797	down
KRTAP5-5	NM_001001480	keratin associated protein 5-5	-2.3037202	down
MAP3K10	NM_002446	mitogen-activated protein kinase kinase kinase 10	-3.0484006	down
MAGEB10	NM_182506	melanoma antigen family B, 10	-2.435996	down
	U80770		-2.6299403	down
DRGX		dorsal root ganglia homeobox	-2.05292	down
SHOX	NM_006883	short stature homeobox	-4.3361726	down
LOC147004	AK057317	uncharacterized LOC147004	-3.4306297	down
CXorf64	NM_001122716	chromosome X open reading frame 64	-2.1719856	down
KRT26	NM_181539	keratin 26	-2.0922265	down
MUCL1	NM_058173	mucin-like 1	-3.1079416	down
			-2.6670797	down
PTN	NM_002825	pleiotrophin	-2.4462154	down
PTPRH	NM_002842	protein tyrosine phosphatase, receptor type, H	-2.4729233	down
C17orf82	NM_203425	chromosome 17 open reading frame 82	-3.0048323	down
CSH1	NM_001317	chorionic somatomammotropin hormone 1 (placental lactogen)	-3.4446065	down
OR6P1	NM_001160325	olfactory receptor, family 6, subfamily P, member 1	-2.0816424	down
HAB1	BU190374	B1 for mucin	-2.7543426	down
SERINC2	NM_178865	serine incorporator 2	-2.1549928	down
	AK293046		-2.1472292	down
PPP1R1C	NM_001080545	protein phosphatase 1, regulatory (inhibitor) subunit 1C	-2.2610533	down
MAP3K13	NM_004721	mitogen-activated protein kinase kinase kinase 13	-2.3992116	down
LOC100128126	NR_038406	uncharacterized LOC100128126	-2.489578	down
SEZ6L	NM_021115	seizure related 6 homolog (mouse)-like	-3.1217635	down

SLC2A10	NM_030777	solute carrier family 2 (facilitated glucose transporter), member 10	-2.0907114	down
DEFB1	NM_005218	defensin, beta 1	-2.3869355	down
PDILT	NM_174924	protein disulfide isomerase-like, testis expressed	-4.672183	down
	XM_003403800		-2.4421797	down
PIKFYVE	NM_001178000	phosphoinositide kinase, FYVE finger containing	-2.231356	down
LOC729020	NM_001143909	rcRPE	-2.6608815	down
OAS3	NM_006187	2'-5'-oligoadenylate synthetase 3, 100kDa	-2.073909	down
GTSE1	NM_016426	G-2 and S-phase expressed 1	-2.2025383	down
OTC	NM_000531	ornithine carbamoyltransferase	-3.4451053	down
CAPN13	NM_144575	calpain 13	-3.1888301	down
LOC286299	BX648501	uncharacterized LOC286299	-2.9855356	down
TMEM132E	NM_207313	transmembrane protein 132E	-4.284696	down
TRIM17	NM_016102	tripartite motif containing 17	-3.620156	down
			-2.441919	down
MBD3L1	NM_145208	methyl-CpG binding domain protein 3-like 1	-3.1835573	down
TP53TG3	NM_016212	TP53 target 3	-2.0361729	down
PDE4DIP	AK024906	phosphodiesterase 4D interacting protein	-2.464693	down
AP1S2	AK299921	adaptor-related protein complex 1, sigma 2 subunit	-2.379591	down
			-2.3792312	down
FLJ42289	NR_028139	uncharacterized LOC388182	-2.0919814	down
ODF3B	NM_001014440	outer dense fiber of sperm tails 3B	-3.0685577	down
LOC100128366	XR_132755	uncharacterized LOC100128366	-2.9917042	down
OLFM2	NM_058164	olfactomedin 2	-2.5303257	down
LOC100133319	AF132201	PRO1804	-2.7913573	down
CYP46A1	AB209749	cytochrome P450, family 46, subfamily A, polypeptide 1	-2.2400594	down
			-2.6587827	down
CT45A5	NM_001007551	cancer/testis antigen family 45, member A5	-8.094015	down
C6orf118	NM_144980	chromosome 6 open reading frame 118	-2.1295283	down
			-3.1050115	down
NFASC	NM_001005388	neurofascin	-2.6506538	down
ARCN1	NM_001655	archain 1	-3.026219	down
EMR4P	AY181245	egf-like module containing, mucin-like, hormone receptor-like 4 pseudogene	-2.3522377	down
C17orf66	NM_152781	chromosome 17 open reading frame 66	-2.6208339	down
			-3.6261485	down
LINC00328	AF172850	long intergenic non-protein coding RNA 328	-3.3823345	down
LOC100130480	NM_001243517	uncharacterized LOC100130480	-5.265971	down
OR4M1	NM_001005500	olfactory receptor, family 4, subfamily M, member 1	-2.6218333	down
EPX	NM_000502	eosinophil peroxidase	-2.1721497	down
LOC280665	AF547222	anti-CNG alpha 1 cation channel translation product-like	-2.2615285	down
			-2.6959102	down

PLK1	NM_005030	polo-like kinase 1	-2.1234255	down
IGSF21	NM_032880	immunoglobin superfamily, member 21	-2.7440553	down
LOC100507003	NM_001195256	uncharacterized protein LOC100507003	-4.1581097	down
LOC152286	AL117431	uncharacterized LOC152286	-2.150858	down
SELV	NM_182704	selenoprotein V	-4.503737	down
LINC00256A	NR_024366	long intergenic non-protein coding RNA 256A	-2.2850823	down
			-2.3124392	down
LRRC48	NM_031294	leucine rich repeat containing 48	-3.5324757	down
PEX7	NM_000288	peroxisomal biogenesis factor 7	-3.168864	down
CADPS	NM_183393	Ca++-dependent secretion activator	-2.1044981	down
			-2.3382058	down
			-4.4931135	down
	AK000950		-4.49499	down
OR51L1	NM_001004755	olfactory receptor, family 51, subfamily L, member 1	-2.3236735	down
			-2.1920462	down
IAPP	NM_000415	islet amyloid polypeptide	-2.7572558	down
TPO	NM_175721	thyroid peroxidase	-2.2086618	down
ILDR1	NM_001199799	immunoglobulin-like domain containing receptor 1	-3.69501	down
PPP1R12B	NM_001167858	protein phosphatase 1, regulatory subunit 12B	-2.428456	down
POM121L4P	NR_024592	POM121 membrane glycoprotein-like 4 pseudogene	-6.7370973	down
			-3.391712	down
ALPK3	NM_020778	alpha-kinase 3	-3.1536317	down
HOXD12	NM_021193	homeobox D12	-3.730177	down
SLC22A24	NM_001136506	solute carrier family 22, member 24	-2.218759	down
CLEC4M	NM_001144911	C-type lectin domain family 4, member M	-3.3323767	down
PLXDC1	NM_020405	plexin domain containing 1	-2.8136203	down
MMP2	NM_004530	matrix metallopeptidase 2 (gelatinase A, 72kDa gelatinase, 72kDa type IV collagenase)	-3.0168788	down
LOC100130713	AK096566	uncharacterized LOC100130713	-2.4770176	down
RHO	NM_000539	rhodopsin	-2.0070555	down
KRT15	NM_002275	keratin 15	-2.0723236	down
	AK056856		-2.1946433	down
LOC100128851	AK127423	uncharacterized LOC100128851	-3.5658264	down
LYPD4	BC034629	LY6/PLAUR domain containing 4	-2.368074	down
			-3.4043262	down
SIM1	NM_005068	single-minded homolog 1 (Drosophila)	-3.516522	down
ERBB4	NM_005235	v-erb-a erythroblastic leukemia viral oncogene homolog 4 (avian)	-3.0228953	down
TTC39A	AB007921	tetratricopeptide repeat domain 39A	-4.0860405	down
SSC5D	NM_001144950	scavenger receptor cysteine rich domain containing (5 domains)	-3.9298322	down
psiTPTE22	AK226145	TPTE pseudogene	-2.7495475	down
			-2.1346862	down

	AK130024		-2.1361983	down
FAM20A	NM_017565	family with sequence similarity 20, member A	-2.5970764	down
C21orf62	NM_019596	chromosome 21 open reading frame 62	-2.2702794	down
YY2	NM_206923	YY2 transcription factor	-2.745018	down
SH2D4B	NM_207372	SH2 domain containing 4B	-2.143373	down
			-2.1196024	down
KLK3	NM_001030050	kallikrein-related peptidase 3	-3.9587147	down
	XR_133518		-2.1441925	down
FAM75C2	NM_001166137	family with sequence similarity 75, member C2	-2.105384	down
HEYL	NM_014571	hairy/enhancer-of-split related with YRPW motif-like	-2.776064	down
WDR6	NM_018031	WD repeat domain 6	-3.2026353	down
VTCN1	NM_024626	V-set domain containing T cell activation inhibitor 1	-2.519322	down
	DA380926		-2.8633425	down
FZD10	NM_007197	frizzled family receptor 10	-2.1467643	down
RSPO1	NM_001038633	R-spondin 1	-2.2965157	down
ATP7A	NM_000052	ATPase, Cu++ transporting, alpha polypeptide	-2.302463	down
PBOV1	NM_021635	prostate and breast cancer overexpressed 1	-2.8565507	down
DLL4	NM_019074	delta-like 4 (Drosophila)	-2.6120486	down
TBX15	BX537778	T-box 15	-2.572243	down
LOC100130370	XM_001714096	uncharacterized LOC100130370	-2.462087	down
GGN	NM_152657	gametogenetin	-3.048785	down
			-2.323868	down
LOC391764	XM_001713926	putative TAF11-like protein ENSP00000332601-like	-2.0393913	down
STXBP1	NM_003165	syntaxin binding protein 1	-11.575024	down
PRSS35	NM_153362	protease, serine, 35	-3.7475495	down
ARMC4	NM_018076	armadillo repeat containing 4	-2.4722443	down
FAM182A	NR_026713	family with sequence similarity 182, member A	-3.3738337	down
GPHA2	NM_130769	glycoprotein hormone alpha 2	-2.9351585	down
ERVH-3	AB128832	endogenous retrovirus group H, member 3	-2.152782	down
	XR_132502		-3.025131	down
	DA827940		-2.9747903	down
C11orf44	XR_110537	chromosome 11 open reading frame 44	-2.7863092	down
	XR_132808		-2.5082736	down
C9orf53	NR_024274	chromosome 9 open reading frame 53	-2.208523	down
LOC390705	NR_033866	protein phosphatase 2, regulatory subunit B'', beta pseudogene	-3.3711836	down
ALDH3A1	NM_001135168	aldehyde dehydrogenase 3 family, member A1	-2.2598565	down
FAM115A	NM_014719	family with sequence similarity 115, member A	-2.575283	down
LOC100130027	XR_110587	uncharacterized LOC100130027	-2.3434985	down
GPR143	NM_000273	G protein-coupled receptor 143	-2.0001326	down
			-2.7141237	down
O3FAR1	NM_181745	omega-3 fatty acid receptor 1	-3.5376074	down

EIF4EBP1	NM_004095	eukaryotic translation initiation factor 4E binding protein 1	-2.128559	down
CCL20	NM_004591	chemokine (C-C motif) ligand 20	-5.3729663	down
ARHGAP29	NM_004815	Rho GTPase activating protein 29	-2.3029828	down
LCE3A	NM_178431	late cornified envelope 3A	-4.215301	down
			-2.4659622	down
LINC00469	NR_027146	long intergenic non-protein coding RNA 469	-3.5327144	down
PDZD2	AK128686	PDZ domain containing 2	-2.0075896	down
FPGS	NM_004957	folylpolyglutamate synthase	-2.7402375	down
LOC100505873	XR_132638	putative apoptosis-related protein 2-like	-2.0176792	down
GHRH	NM_021081	growth hormone releasing hormone	-2.4451466	down
PLOD3	NM_001084	procollagen-lysine, 2-oxoglutarate 5-dioxygenase 3	-2.2753787	down
	XR_110159		-2.81897	down
	XM_001723179		-3.3416808	down
			-3.2798095	down
			-2.6721013	down
TMEM2		transmembrane protein 2	-3.3218708	down
FLJ42102	NR_038862	uncharacterized LOC399923	-3.0024512	down
GSC	NM_173849	goosecoid homeobox	-2.2491138	down
			-2.5129466	down
	BE468260		-2.3224392	down
GDF5	NM_000557	growth differentiation factor 5	-2.5101643	down
			-5.8776093	down
SARDH	NM_007101	sarcosine dehydrogenase	-2.4933617	down
AHI1		Abelson helper integration site 1	-2.2153018	down
LOC100288018	XM_002342495	putative TAF11-like protein ENSP00000332601-like	-2.6748283	down
KIF21B	NM_001252102	kinesin family member 21B	-2.2452161	down
TRPV1	NM_080706	transient receptor potential cation channel, subfamily V, member 1	-4.1230664	down
			-3.051634	down
POLM	AK092801	polymerase (DNA directed), mu	-6.1459208	down
PGF	NM_002632	placental growth factor	-4.1989174	down
HOGA1	AK094791	4-hydroxy-2-oxoglutarate aldolase 1	-2.9133787	down
	BX350880		-3.5348084	down
			-2.3641286	down
FARP2	NM_014808	FERM, RhoGEF and pleckstrin domain protein 2	-3.1892345	down
OR4C13	NM_001001955	olfactory receptor, family 4, subfamily C, member 13	-2.7805126	down
LOC652276	NR_015441	potassium channel tetramerisation domain containing 5 pseudogene	-3.7802067	down
RPRM	NM_019845	reprimo, TP53 dependent G2 arrest mediator candidate	-3.009193	down
C17orf104	NM_001145080	chromosome 17 open reading frame 104	-2.2853482	down
C1orf110	AK123573	chromosome 1 open reading frame 110	-2.212149	down
MIA	NM_006533	melanoma inhibitory activity	-3.0630538	down
C11orf91	NM_001166692	chromosome 11 open reading frame 91	-3.2158985	down

LINC00112	NR_024028	long intergenic non-protein coding RNA 112	-3.696973	down
MAP3K9	NM_033141	mitogen-activated protein kinase kinase kinase 9	-2.248949	down
KREMEN1	NM_032045	kringle containing transmembrane protein 1	-3.0111268	down
RAB3C	NM_138453	RAB3C, member RAS oncogene family	-2.6397803	down
SLC26A1	NM_022042	solute carrier family 26 (sulfate transporter), member 1	-2.140201	down
			-4.1089587	down
PRRG3		proline rich Gla (G-carboxyglutamic acid) 3 (transmembrane)	-4.0721474	down
LOC440518	NR_033899	golgin A2 pseudogene	-2.8164687	down
RAET1E	NM_139165	retinoic acid early transcript 1E	-2.6243067	down
			-2.1660411	down
			-3.950393	down
LRCH3	NM_032773	leucine-rich repeats and calponin homology (CH) domain containing 3	-2.3075314	down
CLIC5	NM_016929	chloride intracellular channel 5	-2.813209	down
PCDH8	NM_002590	protocadherin 8	-2.3611255	down
	AK057379		-5.463425	down
	AK098828		-2.1194508	down
REP15	NM_001029874	RAB15 effector protein	-3.6371796	down
	BC069683		-3.0403626	down
LOC339685	NR_038922	uncharacterized LOC339685	-2.120838	down
PATE1	NM_138294	prostate and testis expressed 1	-2.601538	down
SLC19A3		solute carrier family 19, member 3	-2.89024	down
MYCT1	NM_025107	myc target 1	-3.1453035	down
LOC200261	NR_034149	uncharacterized LOC200261	-2.534903	down
AK4	NM_001005353	adenylate kinase 4	-4.0319743	down
			-2.2034023	down
FAM116B	NM_001001794	family with sequence similarity 116, member B	-2.6293757	down
RTN4RL1	NM_178568	reticulon 4 receptor-like 1	-2.4262722	down
SEPN1	NM_020451	selenoprotein N, 1	-2.6878133	down
RBMY1B	NM_001006121	RNA binding motif protein, Y-linked, family 1, member B	-2.2463882	down
			-5.8662295	down
			-2.0807428	down
			-3.1178367	down
	XM_003403554		-3.155046	down
LOC100130442	AK131364	uncharacterized LOC100130442	-4.7179313	down
L2HGDH	NM_024884	L-2-hydroxyglutarate dehydrogenase	-3.6938467	down
			-3.3330872	down
MUC12	NM_001164462	mucin 12, cell surface associated	-2.31573	down
LUZP4	NM_016383	leucine zipper protein 4	-2.3685749	down
	DA825750		-2.1884382	down
LY6D	NM_003695	lymphocyte antigen 6 complex, locus D	-2.1063852	down
ABHD12B	NM_001206673	abhydrolase domain containing 12B	-2.2948968	down

C7	NM_000587	complement component 7	-4.3760676	down
			-2.1417725	down
			-2.1340694	down
MAGEB16	NM_001099921	melanoma antigen family B, 16	-3.1281996	down
LOC339788	NR_015405	uncharacterized LOC339788	-3.0976439	down
TERF1	AK128828	telomeric repeat binding factor (NIMA-interacting) 1	-2.939499	down
LOC100131650	AK124894	uncharacterized LOC100131650	-2.3067143	down
FLJ32955	XR_108437	uncharacterized protein FLJ32955	-2.436399	down
			-2.263893	down
HIGD1B	NM_016438	HIG1 hypoxia inducible domain family, member 1B	-4.7088146	down
	AK127494		-2.2153118	down
C8orf66	AL834492	chromosome 8 open reading frame 66	-5.335049	down
GGNBP1	NR_028361	gametogenetin binding protein 1	-2.296233	down
	XR_108962		-2.131698	down
GJA4	NM_002060	gap junction protein, alpha 4, 37kDa	-2.754091	down
FLJ45482	AK127393	uncharacterized LOC645566	-5.4473696	down
ZNF221	NM_013359	zinc finger protein 221	-2.2103717	down
	BM264234		-2.0487006	down
RIBC1	NM_144968	RIB43A domain with coiled-coils 1	-2.9950383	down
WNK2	NM_006648	WNK lysine deficient protein kinase 2	-2.8913014	down
CNTN6	NM_014461	contactin 6	-2.5210204	down
			-2.5062912	down
CALML6	NM_138705	calmodulin-like 6	-2.3114624	down
APOC4	NM_001646	apolipoprotein C-IV	-2.2767167	down
OR13H1	NM_001004486	olfactory receptor, family 13, subfamily H, member 1	-2.2315154	down
LOC401480	AK056998	uncharacterized LOC401480	-2.558823	down
ATF7IP	NM_018179	activating transcription factor 7 interacting protein	-2.179154	down
LRRC3B	NM_052953	leucine rich repeat containing 3B	-2.304273	down
IGSF11	NM_152538	immunoglobulin superfamily, member 11	-2.044415	down
LOC442122	AK128759	uncharacterized LOC442122	-3.912592	down
ZFHX2	NM_033400	zinc finger homeobox 2	-2.0057359	down
SNX29	BC029857	sorting nexin 29	-2.550967	down
TAF7L	NM_024885	TAF7-like RNA polymerase II, TATA box binding protein (TBP)-associated factor, 50kDa	-4.237088	down
B9D1	NM_015681	B9 protein domain 1	-2.1498072	down
	XR_109115		-2.995904	down
			-5.541071	down
C11orf94	NM_001080446	chromosome 11 open reading frame 94	-2.2475994	down
LOC100128644	XR_110032	LMNE6487	-2.3389635	down
LOC340094	NR_026994	uncharacterized LOC340094	-2.321115	down
LPA	NM_005577	lipoprotein, Lp(a)	-2.062975	down

SLC22A7	NM_153320	solute carrier family 22 (organic anion transporter), member 7	-2.5640233	down
DCN	NM_001920	decorin	-2.601472	down
			-2.8931408	down
ADAMTSL5	NM_213604	ADAMTS-like 5	-3.1528556	down
LOC400682	XM_003118492	zinc finger protein 100-like	-3.1319497	down
LOC645586	AK057937	uncharacterized LOC645586	-3.1844053	down
LAMA3	BC093406	laminin, alpha 3	-2.5552838	down
FAM83H	NM_198488	family with sequence similarity 83, member H	-2.2783282	down
RLIM	NM_183353	ring finger protein, LIM domain interacting	-2.0225472	down
TBXA2R	NM_001060	thromboxane A2 receptor	-2.2187736	down
ZNRF3	NM_001206998	zinc and ring finger 3	-4.799175	down
SH3TC1	BC068094	SH3 domain and tetratricopeptide repeats 1	-2.5559738	down
OR7E12P	NR_044999	olfactory receptor, family 7, subfamily E, member 12 pseudogene	-2.3326104	down
CCL15	NM_032965	chemokine (C-C motif) ligand 15	-2.2070825	down
LOC440082	AK131474	uncharacterized LOC440082	-2.0122592	down
MGC11082	BC094703	uncharacterized LOC84777	-2.0489757	down
	AK127982		-2.0305517	down
TRPM4	NM_017636	transient receptor potential cation channel, subfamily M, member 4	-2.555945	down
MYOZ3	NM_133371	myozenin 3	-3.1188323	down
TAS2R8	NM_023918	taste receptor, type 2, member 8	-2.297789	down
			-2.9971247	down
			-2.0324469	down
EPM2A	NM_001018041	epilepsy, progressive myoclonus type 2A, Lafora disease (laforin)	-2.4355779	down
ZNF365	NM_199451	zinc finger protein 365	-3.8610733	down
			-2.2933345	down
PSG10P	L14723	pregnancy specific beta-1-glycoprotein 10, pseudogene	-5.8618336	down
PCDH10	NM_032961	protocadherin 10	-2.4410305	down
OTOG	XM_001717531	otogelin	-2.0625606	down
BCOR		BCL6 corepressor	-2.0180728	down
LOC100130433	AK096255	uncharacterized LOC100130433	-2.5512693	down
			-7.8933845	down
C9orf169	NM_199001	chromosome 9 open reading frame 169	-2.0445554	down
MEX3D	NM_001174118	mex-3 homolog D (C. elegans)	-2.4165623	down
DUOXA2	BX537581	dual oxidase maturation factor 2	-2.217342	down
PGP	NM_001042371	phosphoglycolate phosphatase	-2.0516772	down
			-3.0303755	down
PKD2L1	NM_016112	polycystic kidney disease 2-like 1	-2.9406118	down
OR2A5	NM_012365	olfactory receptor, family 2, subfamily A, member 5	-3.9019973	down
IVL	NM_005547	involucrin	-3.6366699	down
C18orf15	AK055900	chromosome 18 open reading frame 15	-4.244676	down
PCDHA9	NM_014005	protocadherin alpha 9	-2.320552	down

ATP2A1	NM_173201	ATPase, Ca++ transporting, cardiac muscle, fast twitch 1	-2.6509368	down
C10orf67	NM_153714	chromosome 10 open reading frame 67	-2.49525	down
NEUROD2	NM_006160	neurogenic differentiation 2	-2.582318	down
	DA292495		-4.4575987	down
KLF17	NM_173484	Kruppel-like factor 17	-2.9071062	down
			-2.0764837	down
LINC00471	NR_024079	long intergenic non-protein coding RNA 471	-2.558882	down
LOC400950		uncharacterized LOC400950	-2.6039388	down
SRRM2	NM_016333	serine/arginine repetitive matrix 2	-2.4846544	down
ITPKA	NM_002220	inositol-trisphosphate 3-kinase A	-2.3765798	down
ZNF710	NM_198526	zinc finger protein 710	-3.2708488	down
HIC2	NM_015094	hypermethylated in cancer 2	-3.6411602	down
			-2.0564435	down
ARHGEF25	NM_182947	Rho guanine nucleotide exchange factor (GEF) 25	-2.2068572	down
LOC100128640	NR_028389	uncharacterized LOC100128640	-2.6750498	down
CENPM	NM_001002876	centromere protein M	-3.0034392	down
	AK126822		-2.144103	down
	XR_109126		-2.3793402	down
SNAR-A3	NR_024214	small ILF3/NF90-associated RNA A3	-2.610968	down
ARTN	NM_057090	artemin	-2.9977477	down
IKZF3	NM_012481	IKAROS family zinc finger 3 (Aiolos)	-2.4724152	down
C1orf187	NM_198545	chromosome 1 open reading frame 187	-4.297741	down
GOLGA8IP	NR_024074	golgin A8 family, member I, pseudogene	-2.3570118	down
KERA	NM_007035	keratocan	-2.166617	down
CCDC18	NM_206886	coiled-coil domain containing 18	-2.0145316	down
			-5.5404215	down
TREM2	NM_018965	triggering receptor expressed on myeloid cells 2	-2.5101337	down
OR51V1	NM_001004760	olfactory receptor, family 51, subfamily V, member 1	-3.1856377	down
AGXT	NM_000030	alanine-glyoxylate aminotransferase	-2.421701	down
LOC440297	NR_033579	chondroitin sulfate proteoglycan 4 pseudogene	-2.7088027	down
SRGAP1	NM_020762	SLIT-ROBO Rho GTPase activating protein 1	-5.9805984	down
LOC100128184	AK128032	uncharacterized LOC100128184	-3.726653	down
C9orf79	NM_178828	chromosome 9 open reading frame 79	-3.0858095	down
PAQR9	NM_198504	progestin and adipoQ receptor family member IX	-2.3362849	down
	BC137370		-3.0560966	down
			-3.1110375	down
C12orf42	BC039352	chromosome 12 open reading frame 42	-2.0324252	down
OR2Z1	NM_001004699	olfactory receptor, family 2, subfamily Z, member 1	-4.651697	down
KRTAP5-7	NM_001012503	keratin associated protein 5-7	-3.689695	down
AKR1C1		aldo-keto reductase family 1, member C1 (dihydrodiol dehydrogenase 1; 20-alpha (3-alpha)-hydroxysteroid dehydrogenase)	-2.9251955	down

KIAA1804	NM_032435	mixed lineage kinase 4	-3.1814885	down
			-2.0366514	down
			-3.0949423	down
PRSS37	NM_001008270	protease, serine, 37	-2.7350087	down
	AB529249		-2.4436464	down
TCF25	NM_014972	transcription factor 25 (basic helix-loop-helix)	-2.0621836	down
IL19	NM_153758	interleukin 19	-2.0626311	down
MS4A4E	XM_003119183	membrane-spanning 4-domains, subfamily A, member 4E	-2.944113	down
HNF1A	X71347	HNF1 homeobox A	-9.948551	down
PRDM5	NM_018699	PR domain containing 5	-2.291478	down
SPATC1	NM_198572	spermatogenesis and centriole associated 1	-3.3295693	down
FGL1	NM_201553	fibrinogen-like 1	-3.7432191	down
ACBD7	NM_001039844	acyl-CoA binding domain containing 7	-3.5007224	down
OR7E91P	NR_002185	olfactory receptor, family 7, subfamily E, member 91 pseudogene	-2.177752	down
			-3.9875796	down
	CR599065		-3.7671514	down
ANKDD1A	NM_182703	ankyrin repeat and death domain containing 1A	-2.6914237	down
	DW451783		-3.93076	down
			-2.163832	down
			-2.5405533	down
SLC12A1	NM_000338	solute carrier family 12 (sodium/potassium/chloride transporters), member 1	-2.1636744	down
ARHGAP32	NM_014715	Rho GTPase activating protein 32	-2.3476632	down
CHRFAM7A	NM_139320	CHRNA7 (cholinergic receptor, nicotinic, alpha 7, exons 5-10) and FAM7A (family with sequence similarity 7A, exons A-E) fusion	-2.266517	down
			-4.634769	down
			-5.431114	down
			-4.826088	down
SYTL4	AL832596	synaptotagmin-like 4	-3.0938442	down
TRIM66	NM_014818	tripartite motif containing 66	-2.0310817	down
FAM153A	NM_173663	family with sequence similarity 153, member A	-2.7518253	down
MYL7	NM_021223	myosin, light chain 7, regulatory	-3.0365138	down
TSSK6	NM_032037	testis-specific serine kinase 6	-3.1816342	down
KRT78	NM_173352	keratin 78	-3.6873376	down
MGC45800	NR_027107	uncharacterized LOC90768	-2.265468	down
PRKACG	NM_002732	protein kinase, cAMP-dependent, catalytic, gamma	-2.1536882	down
GPX3	NM_002084	glutathione peroxidase 3 (plasma)	-26.24971	down
	AB529247		-2.674829	down
MAGI1	NM_004742	membrane associated guanylate kinase, WW and PDZ domain containing 1	-2.219465	down
			-2.26418	down
C5orf56	AK096941	chromosome 5 open reading frame 56	-2.3479493	down
	XM_003119194		-2.187194	down

PGF	AK023843	placental growth factor	-3.2633703	down
GNG4	NM_001098722	guanine nucleotide binding protein (G protein), gamma 4	-2.2099357	down
WT1-AS	NR_023920	WT1 antisense RNA (non-protein coding)	-2.638942	down
	AK095662		-3.7249205	down
THRSP	NM_003251	thyroid hormone responsive	-2.5471523	down
NPY5R	NM_006174	neuropeptide Y receptor Y5	-2.4194074	down
C6orf191	NM_001010876	chromosome 6 open reading frame 191	-2.596214	down
LOC100287879	NR_033978	uncharacterized LOC100287879	-2.8703406	down
LOC100130876	XR_109361	uncharacterized LOC100130876	-2.5611365	down
KCNG1	BC006367	potassium voltage-gated channel, subfamily G, member 1	-2.251751	down
ADRA2B	NM_000682	adrenergic, alpha-2B-, receptor	-2.0081847	down
CDH23	NM_022124	cadherin-related 23	-2.3900423	down
CACNG7	NM_031896	calcium channel, voltage-dependent, gamma subunit 7	-3.4514198	down
ANKRD36B	NM_025190	ankyrin repeat domain 36B	-2.42802	down
	AK097428		-2.2840698	down
			-8.08274	down
DDC	NM_001242890	dopa decarboxylase (aromatic L-amino acid decarboxylase)	-2.8398564	down
C1orf98	NR_040064	chromosome 1 open reading frame 98	-3.9664595	down
			-4.1982193	down
LOC100132495	AK127414	uncharacterized LOC100132495	-2.2449865	down
			-5.6128917	down
C12orf34	NM_032829	chromosome 12 open reading frame 34	-2.464742	down
MGC24103	XR_108934	uncharacterized MGC24103	-3.5006068	down
GIGYF2		GRB10 interacting GYF protein 2	-2.1976812	down
LOC415056	NR_024369	uncharacterized LOC415056	-2.0007253	down
			-2.2938197	down
			-2.466728	down
			-3.697126	down
CRHR1	NM_001145146	corticotropin releasing hormone receptor 1	-3.3979523	down
ADCK3	AK123980	aarF domain containing kinase 3	-2.3748455	down
CRX	NM_000554	cone-rod homeobox	-3.6322737	down
C2orf65	NM_138804	chromosome 2 open reading frame 65	-5.0430565	down
LOC147670	NM_001193628	uncharacterized LOC147670	-3.9596071	down
GRM4	NM_000841	glutamate receptor, metabotropic 4	-3.4541683	down
CNBD1	NM_173538	cyclic nucleotide binding domain containing 1	-2.9723847	down
MFSD2B	NM_001080473	major facilitator superfamily domain containing 2B	-2.5988963	down
			-3.6310484	down
FAM95B1	NR_026759	family with sequence similarity 95, member B1	-6.888975	down
FOXL1	NM_005250	forkhead box L1	-2.4489553	down
PKD1L2	NM_052892	polycystic kidney disease 1-like 2	-3.6406822	down
ASS1	NM_000050	argininosuccinate synthase 1	-4.2798576	down

MSTO2P	NR_024117	misato homolog 2 pseudogene	-3.1386647	down
			-8.059803	down
DOT1L	NM_032482	DOT1-like, histone H3 methyltransferase (S. cerevisiae)	-2.0727584	down
PLAC9		placenta-specific 9	-2.4713573	down
SMARCA1	NM_003069	SWI/SNF related, matrix associated, actin dependent regulator of chromatin, subfamily a, member 1	-2.0854108	down
			-2.085765	down
LINC00085	NR_024330	long intergenic non-protein coding RNA 85	-2.171319	down
DCAKD	NM_024819	dephospho-CoA kinase domain containing	-3.5462472	down
GABRB3	BC038837	gamma-aminobutyric acid (GABA) A receptor, beta 3	-2.5747182	down
LOC340335	AK074459	uncharacterized LOC340335	-2.6379824	down
			-2.2797046	down
BRD7		bromodomain containing 7	-8.3824215	down
	CB962925		-5.0996275	down
CETN1	NM_004066	centrin, EF-hand protein, 1	-2.6012702	down
RBPJL	NM_014276	recombination signal binding protein for immunoglobulin kappa J region-like	-2.5140555	down
	AY358259		-2.1031651	down
C12orf33	AK123808	chromosome 12 open reading frame 33	-2.3952613	down
CREG2	NM_153836	cellular repressor of E1A-stimulated genes 2	-3.1792367	down
CRABP2	NM_001878	cellular retinoic acid binding protein 2	-2.2594383	down
CPS1-IT1	NR_002763	CPS1 intronic transcript 1 (non-protein coding)	-2.132304	down
NRXN2	NM_138732	neurexin 2	-2.7401717	down
			-3.6120808	down
			-4.3502536	down
CROCCP2	AK123337	ciliary rootlet coiled-coil, rootletin pseudogene 2	-2.0799756	down
LOC648691	NR_027426	uncharacterized LOC648691	-2.7376802	down
ZNF365	NM_199451	zinc finger protein 365	-2.3908486	down
LOC100129213	NR_038419	uncharacterized LOC100129213	-4.172857	down
			-2.5002108	down
GCNT7	NM_080615	glucosaminyl (N-acetyl) transferase family member 7	-2.0400364	down
	CR997556		-2.563053	down
			-3.6988819	down
LRRC37A3	NM_199340	leucine rich repeat containing 37, member A3	-3.8995996	down
C11orf20	NM_001039496	chromosome 11 open reading frame 20	-2.8640788	down
			-2.521992	down
GPR27	NM_018971	G protein-coupled receptor 27	-2.7395535	down
CCNB3	NM_033031	cyclin B3	-2.4409332	down
MOK	NM_014226	MOK protein kinase	-3.1339493	down
			-6.421004	down
C15orf2	NM_018958	chromosome 15 open reading frame 2	-5.384669	down
LOC284395	NR_040029	uncharacterized LOC284395	-3.5200908	down

MOBP	NM_182935	myelin-associated oligodendrocyte basic protein	-2.4987159	down
RNF151	NM_174903	ring finger protein 151	-6.9431434	down
			-5.995757	down
FLJ46300	NR_038365	FLJ46300 protein	-2.3785203	down
XIRP1	NM_194293	xin actin-binding repeat containing 1	-2.0768394	down
HTR1A	NM_000524	5-hydroxytryptamine (serotonin) receptor 1A	-2.294744	down
TRIM15	NM_033229	tripartite motif containing 15	-2.6229405	down
			-3.247132	down
PRDM8	NM_020226	PR domain containing 8	-2.211857	down
	DB452078		-5.68079	down
ATMIN	AK302556	ATM interactor	-3.0260386	down
CHRDL1	NM_145234	chordin-like 1	-4.456384	down
CIB4	NM_001029881	calcium and integrin binding family member 4	-2.0584834	down
NRG1	AF176921	neuregulin 1	-2.4161296	down
			-2.4606175	down
CAND1	NM_018448	cullin-associated and neddylation-dissociated 1	-2.76554	down
LOC728208	XR_108453	uncharacterized LOC728208	-2.0081418	down
NWD1	NM_001007525	NACHT and WD repeat domain containing 1	-2.9914217	down
ITLN2	NM_080878	intelectin 2	-2.399439	down
LOC100128386	AK124326	uncharacterized LOC100128386	-3.6855512	down
BMP8B	NM_001720	bone morphogenetic protein 8b	-3.4113386	down
			-2.8334656	down
			-2.3038237	down
C6orf132	NM_001164446	chromosome 6 open reading frame 132	-2.0614069	down
IGF2-AS	NR_028044	insulin-like growth factor 2 antisense (non-protein coding)	-2.2078357	down
			-2.2797542	down
KRTAP5-8	NM_021046	keratin associated protein 5-8	-2.9945402	down
LRRC37BP1	AK125981	leucine rich repeat containing 37B pseudogene 1	-2.0038986	down
DLGAP1	BC040718	discs, large (Drosophila) homolog-associated protein 1	-2.4041307	down
			-2.3075495	down
IGLON5	NM_001101372	IgLON family member 5	-4.5871277	down
CXorf36	NM_176819	chromosome X open reading frame 36	-3.6356258	down
			-3.9116843	down
OR1D5	NM_014566	olfactory receptor, family 1, subfamily D, member 5	-2.5481603	down
	BG192758		-4.4178777	down
SPG7	AF090912	spastic paraplegia 7 (pure and complicated autosomal recessive)	-2.546172	down
LOC401164	AK095968	uncharacterized LOC401164	-2.3773453	down
NAV1	NM_020443	neuron navigator 1	-2.265468	down
ECM2	NM_001197296	extracellular matrix protein 2, female organ and adipocyte specific	-2.1066232	down
TMED7-TICAM2	NM_001164469	TMED7-TICAM2 readthrough	-4.454804	down
CCL13	NM_005408	chemokine (C-C motif) ligand 13	-2.7365208	down

CNTN1	NM_001843	contactin 1	-2.6254642	down
TMEM136	NM_174926	transmembrane protein 136	-3.2309813	down
SPRY1		sprouty homolog 1, antagonist of FGF signaling (Drosophila)	-3.9324708	down
HES2	NM_019089	hairy and enhancer of split 2 (Drosophila)	-2.1838522	down
OR5M1	NM_001004740	olfactory receptor, family 5, subfamily M, member 1	-2.0258088	down
LOC643669	NM_001243212	uncharacterized LOC643669	-2.9784377	down
EFNB3	NM_001406	ephrin-B3	-2.0737786	down
			-2.2057874	down
NTM	NM_001144058	neurotrimin	-3.3258417	down
FAM115C	AK090395	family with sequence similarity 115, member C	-2.8501523	down
ZNF735	NM_001159524	zinc finger protein 735	-2.6174467	down
			-3.1889184	down
CD276	NM_001024736	CD276 molecule	-3.9181426	down
KIR3DL3	NM_153443	killer cell immunoglobulin-like receptor, three domains, long cytoplasmic tail, 3	-5.4592304	down
CROT	NM_021151	carnitine O-octanoyltransferase	-2.5305808	down
GPR172B	NM_017986	G protein-coupled receptor 172B	-3.277146	down
KCNK10	NM_021161	potassium channel, subfamily K, member 10	-3.266088	down
	XM_003118656		-6.7563615	down
DNAH10OS	AK127211	dynein, axonemal, heavy chain 10 opposite strand	-2.3963795	down
			-2.272889	down
LOC100128675	NR_024561	uncharacterized LOC100128675	-3.4931052	down
FAM180B	NM_001164379	family with sequence similarity 180, member B	-3.0560126	down
SNTG1		syntrophin, gamma 1	-4.626544	down
LOC400043	NR_026656	uncharacterized LOC400043	-2.0180123	down
LOC100653108	XR_133081	uncharacterized LOC100653108	-3.705672	down
ARHGAP42	NM_152432	Rho GTPase activating protein 42	-5.0928216	down
	BC044628		-2.6442804	down
GRID2	NM_001510	glutamate receptor, ionotropic, delta 2	-2.403945	down
TAF1B		TATA box binding protein (TBP)-associated factor, RNA polymerase I, B, 63kDa	-2.3717206	down
SF3B2	CN273640	splicing factor 3b, subunit 2, 145kDa	-3.7307885	down
HIST2H3A	NM_001005464	histone cluster 2, H3a	-4.079533	down
	AK127863		-4.8730435	down
NKAIN4	NM_152864	Na+/K+ transporting ATPase interacting 4	-2.1338558	down
LOC642776	BC003645	uncharacterized LOC642776	-3.8382444	down
	AA489744		-3.2260072	down
PAK6	NM_020168	p21 protein (Cdc42/Rac)-activated kinase 6	-2.4176488	down
VAPB	NM_004738	VAMP (vesicle-associated membrane protein)-associated protein B and C	-2.6637015	down
C10orf112	XM_003403619	chromosome 10 open reading frame 112	-5.223857	down
LOC284191	AK097198	uncharacterized LOC284191	-3.6086152	down
PDLIM2	NM_176871	PDZ and LIM domain 2 (mystique)	-2.5667872	down

TACC2	NM_206862	transforming, acidic coiled-coil containing protein 2	-2.6267865	down
PIGQ	NM_148920	phosphatidylinositol glycan anchor biosynthesis, class Q	-2.2098374	down
SCXA	NM_001008271	scleraxis homolog A (mouse)	-2.4852164	down
DLK2	NM_206539	delta-like 2 homolog (Drosophila)	-2.4141169	down
RGMB	NM_001012761	RGM domain family, member B	-2.0258934	down
CCBL2	NM_001008661	cysteine conjugate-beta lyase 2	-4.073024	down
			-7.913347	down
			-2.8316476	down
ZNF556	NM_024967	zinc finger protein 556	-4.8221555	down
			-3.5852954	down
LOC400927	NR_002821	TPTE and PTEN homologous inositol lipid phosphatase pseudogene	-2.7839723	down
			-3.0954583	down
HOXB6	NM_018952	homeobox B6	-3.160428	down
KRTAP4-8	NM_031960	keratin associated protein 4-8	-2.6519606	down
CNDP2	NM_018235	CNDP dipeptidase 2 (metallopeptidase M20 family)	-2.2151976	down
LINC00371	XR_132731	long intergenic non-protein coding RNA 371	-2.1707833	down
LOC388387	NR_027254	uncharacterized LOC388387	-2.6735916	down
DNAJC5B	NM_033105	DnaJ (Hsp40) homolog, subfamily C, member 5 beta	-2.5006452	down
BAGE	NM_001187	B melanoma antigen	-2.2087772	down
			-2.5467663	down
SPINK14	NM_001001325	serine peptidase inhibitor, Kazal type 14 (putative)	-2.6004455	down
LOC442122	AK128759	uncharacterized LOC442122	-3.5038638	down
OR5H1	NM_001005338	olfactory receptor, family 5, subfamily H, member 1	-3.2964878	down
			-2.5070555	down
OR51M1	NM_001004756	olfactory receptor, family 51, subfamily M, member 1	-2.374214	down
PTGR2	NM_152444	prostaglandin reductase 2	-8.441116	down
LOC338694	BC043531	uncharacterized LOC338694	-2.4193122	down
LOC100506514	AK124637	uncharacterized LOC100506514	-5.316796	down
			-4.2262087	down
KRTAP10-1	NM_198691	keratin associated protein 10-1	-2.0510616	down
EFR3B	AB023170	EFR3 homolog B (S. cerevisiae)	-5.4527555	down
C12orf56	NM_001170633	chromosome 12 open reading frame 56	-2.3829932	down
			-2.6616418	down
ANKRD19P	AK292218	ankyrin repeat domain 19, pseudogene	-2.4562414	down
	BX404796		-6.1796365	down
EBF2	NM_022659	early B-cell factor 2	-4.952757	down
			-2.0120137	down
			-4.454665	down
	BM925287		-2.3079445	down
LINC00312	NR_024065	long intergenic non-protein coding RNA 312	-2.5898385	down
			-3.0176022	down

	AK124642		-2.088488	down
SCARF2	NM_153334	scavenger receptor class F, member 2	-3.2063382	down
KRT18P55	NR_028334	keratin 18 pseudogene 55	-3.00817	down
C21orf56	NM_032261	chromosome 21 open reading frame 56	-2.1654463	down
ZNF503-AS1	NR_038223	ZNF503 antisense RNA 1 (non-protein coding)	-3.3589785	down
	FV367791		-2.6291692	down
LOC100130849	NR_038450	phosphorylase kinase, gamma 1 (muscle) pseudogene	-2.6875086	down
ZDHHC11	NM_024786	zinc finger, DHHC-type containing 11	-2.5308278	down
RABL2B	BC020495	RAB, member of RAS oncogene family-like 2B	-3.8184621	down
LOC100132731	XM_003403421	uncharacterized LOC100132731	-2.2677796	down
	DB153536		-2.5775142	down
PDPR	NM_017990	pyruvate dehydrogenase phosphatase regulatory subunit	-3.9326153	down
C1orf138	AK127688	chromosome 1 open reading frame 138	-4.0490417	down
AADACL3	NM_001103170	arylacetamide deacetylase-like 3	-4.7020364	down
REXO1L1	NM_172239	REX1, RNA exonuclease 1 homolog (S. cerevisiae)-like 1	-3.3646264	down
TRIM61	NM_001012414	tripartite motif containing 61	-2.6063843	down
	BX115986		-2.9335191	down
RBM43	NM_198557	RNA binding motif protein 43	-2.1906834	down
LOC284379	NR_002938	solute carrier family 7 (cationic amino acid transporter, y+ system), member 3 pseudogene	-2.6562598	down
FAM78B	AB593134	family with sequence similarity 78, member B	-2.043585	down
	AK128457		-2.3191981	down
			-9.558157	down
CCL25	NM_005624	chemokine (C-C motif) ligand 25	-2.8125806	down
STK32A	NM_145001	serine/threonine kinase 32A	-2.125329	down
LOC100128988	AK023614	uncharacterized LOC100128988	-2.5201375	down
OR3A4P	NR_024128	olfactory receptor, family 3, subfamily A, member 4 pseudogene	-2.0444016	down
REG1B	NM_006507	regenerating islet-derived 1 beta	-2.0526686	down
LOC100132005	AK092662	uncharacterized LOC100132005	-2.2618313	down
MIR143HG	NR_027180	MIR143 host gene (non-protein coding)	-2.3195164	down
	BC012174		-2.0057304	down
GPR21	NM_005294	G protein-coupled receptor 21	-3.0188227	down
RAPGEF1	NM_198679	Rap guanine nucleotide exchange factor (GEF) 1	-3.2454271	down
SLC39A5	NM_001135195	solute carrier family 39 (metal ion transporter), member 5	-2.177772	down
OR51D1	NM_001004751	olfactory receptor, family 51, subfamily D, member 1	-2.7234256	down
LOC100128792	AK128181	uncharacterized LOC100128792	-2.9169078	down
PRR15	NM_175887	proline rich 15	-2.8473809	down
ZSCAN5D	XM_001725568	zinc finger and SCAN domain containing 5D	-2.763367	down
AANAT	NM_001166579	aralkylamine N-acetyltransferase	-4.4548736	down
KRT8	BC007663	keratin 8	-2.224495	down
SPATA4	NM_144644	spermatogenesis associated 4	-2.0024173	down

UPK1A	NM_007000	uroplakin 1A	-3.35515	down
			-2.079903	down
LOC728093	XR_133401	putative POM121-like protein 1-like	-2.1696646	down
SPPL2B	NM_152988	signal peptide peptidase-like 2B	-2.3410263	down
SLC38A7	NM_018231	solute carrier family 38, member 7	-3.6438258	down
	AY129018		-2.1229484	down
LRRC41	NM_006369	leucine rich repeat containing 41	-2.4657407	down
OPA1	NM_130831	optic atrophy 1 (autosomal dominant)	-2.2762234	down
IL18R1	NM_003855	interleukin 18 receptor 1	-4.775212	down
C19orf73	NM_018111	chromosome 19 open reading frame 73	-2.770989	down
CKMT1A	NM_001015001	creatine kinase, mitochondrial 1A	-3.4198341	down
C20orf166-AS1	NR_033263	C20orf166 antisense RNA 1 (non-protein coding)	-3.677217	down
SLC22A11	NM_018484	solute carrier family 22 (organic anion/urate transporter), member 11	-2.3128831	down
	XM_003119179		-2.2614367	down
HTR6	NM_000871	5-hydroxytryptamine (serotonin) receptor 6	-4.801688	down
TRDN	NM_001251987	triadin	-2.4237154	down
COLQ	NM_080538	collagen-like tail subunit (single strand of homotrimer) of asymmetric acetylcholinesterase	-2.2697773	down
LOC729732	XR_132886	uncharacterized LOC729732	-5.959142	down
GOLGA6L1	NM_001001413	golgin A6 family-like 1	-3.3031168	down
OR5C1	NM_001001923	olfactory receptor, family 5, subfamily C, member 1	-2.0499384	down
C6orf123	NR_026773	chromosome 6 open reading frame 123	-3.0128644	down
CD300LG	NM_145273	CD300 molecule-like family member g	-4.8576713	down
CD37	AK058093	CD37 molecule	-2.8741121	down
NXPH3	NM_007225	neurexophilin 3	-2.8111084	down
	AW245926		-2.7772305	down
HOXB3	NM_002146	homeobox B3	-3.075062	down
			-2.4572413	down
TCTE1	NM_182539	t-complex-associated-testis-expressed 1	-2.600335	down
RUSC1-AS1	NM_001039517	RUSC1 antisense RNA 1 (non-protein coding)	-4.7683234	down
LOC344887	NR_033752	NmrA-like family domain containing 1 pseudogene	-2.1797147	down
CEND1	NM_016564	cell cycle exit and neuronal differentiation 1	-2.1468523	down
	BC126309		-2.3907735	down
RIPK3	NM_006871	receptor-interacting serine-threonine kinase 3	-2.0493836	down
C3	NM_000064	complement component 3	-3.0477736	down
SYCN	NM_001080468	syncollin	-4.126801	down
SUPT3H		suppressor of Ty 3 homolog (S. cerevisiae)	-2.5391817	down
			-2.6321282	down
			-2.601751	down
TCP10L	NM_144659	t-complex 10 (mouse)-like	-2.1274908	down
LOC285084	NR_038897	uncharacterized LOC285084	-3.2387424	down

ODF4	NM_153007	outer dense fiber of sperm tails 4	-2.342248	down
GATA4	FJ169610	GATA binding protein 4	-3.4079113	down
L2HGDH	NM_024884	L-2-hydroxyglutarate dehydrogenase	-7.3986907	down
LOC200772	NR_033841	uncharacterized LOC200772	-2.3504179	down
			-2.8248224	down
IKZF2	NM_001079526	IKAROS family zinc finger 2 (Helios)	-2.096539	down
FOXB2	NM_001013735	forkhead box B2	-2.2824314	down
LINC00162	NR_024089	long intergenic non-protein coding RNA 162	-2.2422283	down
EPHA10	NM_001099439	EPH receptor A10	-2.0703175	down
LOC400891	NR_027006	chromosome 14 open reading frame 166B pseudogene	-3.673029	down
DMBT1	NM_007329	deleted in malignant brain tumors 1	-5.0211787	down
TTBK1	NM_032538	tau tubulin kinase 1	-3.3152597	down
			-4.1136	down
LOC151009	NR_027244	uncharacterized LOC151009	-2.214634	down
CA10	NM_020178	carbonic anhydrase X	-2.1008806	down
PTCH1	AB214500	patched 1	-5.1449695	down
KLK13	AY923173	kallikrein-related peptidase 13	-2.6436265	down
TNFRSF13C	NM_052945	tumor necrosis factor receptor superfamily, member 13C	-2.2379334	down
SLC22A7	NM_153320	solute carrier family 22 (organic anion transporter), member 7	-3.2391326	down
			-3.094894	down
	XR_109862		-2.6796117	down
SPRR1A	NM_005987	small proline-rich protein 1A	-2.1431	down
			-2.2449286	down
OTOP3	NM_178233	otopetrin 3	-2.1958504	down
LOC100127885	XM_001721771	uncharacterized LOC100127885	-3.6513202	down
PLEKHN1	NM_032129	pleckstrin homology domain containing, family N member 1	-4.4371614	down
TNP1	NM_003284	transition protein 1 (during histone to protamine replacement)	-3.239564	down
C14orf48	NR_024182	chromosome 14 open reading frame 48	-13.302271	down
			-3.5029771	down
PEX11G	NM_080662	peroxisomal biogenesis factor 11 gamma	-4.0354238	down
PREX2	NM_025170	phosphatidylinositol-3,4,5-trisphosphate-dependent Rac exchange factor 2	-2.5224254	down
NTRK3	AK094929	neurotrophic tyrosine kinase, receptor, type 3	-3.1561337	down
			-6.5622582	down
PDPN	NM_198389	podoplanin	-2.4606566	down
			-3.6976383	down
LOC653061	NR_038843	golgin A8 family, member B pseudogene	-6.2678037	down
PDE4D	NM_001197218	phosphodiesterase 4D, cAMP-specific	-3.2914772	down
			-2.5822186	down
AP4M1	NM_004722	adaptor-related protein complex 4, mu 1 subunit	-2.6249983	down
C9orf62	XR_109856	chromosome 9 open reading frame 62	-2.6012628	down

SALL4	NM_020436	sal-like 4 (Drosophila)	-7.585921	down
	AK128018		-2.0155284	down
NUP43	NM_198887	nucleoporin 43kDa	-2.0761888	down
PCDH10	NM_020815	protocadherin 10	-3.8829024	down
			-4.033067	down
			-2.1923654	down
MUC2	NM_002457	mucin 2, oligomeric mucus/gel-forming	-2.0623684	down
NME9	NM_178130	NME gene family member 9	-2.1936576	down
SYT6	NM_205848	synaptotagmin VI	-2.5053356	down
LOC283038	NR_033848	uncharacterized LOC283038	-3.2461724	down
PDDC1	NM_182612	Parkinson disease 7 domain containing 1	-2.0845392	down
HES6	NM_018645	hairy and enhancer of split 6 (Drosophila)	-3.6653175	down
YIPF7	NM_182592	Yip1 domain family, member 7	-2.478259	down
C20orf173	NM_001145350	chromosome 20 open reading frame 173	-2.5379503	down
HMCN1	NM_031935	hemicentin 1	-4.2775693	down
TMCO5A	NM_152453	transmembrane and coiled-coil domains 5A	-2.5085406	down
FAM22D	NM_001009610	family with sequence similarity 22, member D	-2.1489017	down
H2AFB2	NM_001017991	H2A histone family, member B2	-2.1886191	down
OR8H2	NM_001005200	olfactory receptor, family 8, subfamily H, member 2	-6.4763327	down
C2orf27A	NM_013310	chromosome 2 open reading frame 27A	-2.1131563	down
LOC389607	XR_110088	uncharacterized LOC389607	-2.7484398	down
SLC25A16	BC001407	solute carrier family 25 (mitochondrial carrier; Graves disease autoantigen), member 16	-4.07135	down
FLJ41649	NR_038887	uncharacterized LOC401260	-2.1117842	down
POMT2	NM_013382	protein-O-mannosyltransferase 2	-2.5679996	down
FLG	NM_002016	filaggrin	-4.5268593	down
CREB3L3	NM_032607	cAMP responsive element binding protein 3-like 3	-5.5571895	down
LOC283335	NR_033854	uncharacterized LOC283335	-2.1808505	down
ERBB3	NM_001005915	v-erb-b2 erythroblastic leukemia viral oncogene homolog 3 (avian)	-2.7754562	down
OR2A7	NM_001005328	olfactory receptor, family 2, subfamily A, member 7	-2.298693	down
FANCA	NM_001018112	Fanconi anemia, complementation group A	-2.0050056	down
DCAF8	NM_015726	DDB1 and CUL4 associated factor 8	-2.1286006	down
GPR123	NM_001083909	G protein-coupled receptor 123	-2.4919996	down
HIST1H2APS1	NR_045125	histone cluster 1, H2a, pseudogene 1	-2.6281953	down
TPTE2P3	NR_002793	transmembrane phosphoinositide 3-phosphatase and tensin homolog 2 pseudogene 3	-2.5212119	down
TMEM139	NM_153345	transmembrane protein 139	-2.4233544	down
TINAG	NM_014464	tubulointerstitial nephritis antigen	-2.2303288	down
SNCAIP	NM_005460	synuclein, alpha interacting protein	-2.3968954	down
			-12.246122	down
			-3.070447	down
FOXA1		forkhead box A1	-3.9074488	down

OR51E1	NM_152430	olfactory receptor, family 51, subfamily E, member 1	-2.1475463	down
			-3.3969452	down
	XR_110942		-3.2308414	down
TPBG	NM_006670	trophoblast glycoprotein	-3.1373882	down
SLC5A6	NM_021095	solute carrier family 5 (sodium-dependent vitamin transporter), member 6	-2.4534147	down
PDXK	AK094630	pyridoxal (pyridoxine, vitamin B6) kinase	-2.0311298	down
FGF17	NM_003867	fibroblast growth factor 17	-4.1033063	down
CFLAR	NM_001202519	CASP8 and FADD-like apoptosis regulator	-4.6703625	down
			-2.7066426	down
RP1L1	AK127545	retinitis pigmentosa 1-like 1	-2.7269533	down
TNXB	NM_019105	tenascin XB	-4.3601375	down
OR1F1	NM_012360	olfactory receptor, family 1, subfamily F, member 1	-4.514842	down
MRGPRD	NM_198923	MAS-related GPR, member D	-3.5890746	down
LRRN4	NM_152611	leucine rich repeat neuronal 4	-2.4662676	down
C1QTNF7	NM_031911	C1q and tumor necrosis factor related protein 7	-2.151715	down
ZNF812	NM_001199814	zinc finger protein 812	-2.6769469	down
C21orf30	XR_109680	chromosome 21 open reading frame 30	-2.4871452	down
LOC399829	XR_109033	uncharacterized LOC399829	-3.4007292	down
NRN1	NM_016588	neuritin 1	-2.3697023	down
YJEFN3	NM_198537	YjeF N-terminal domain containing 3	-2.1582272	down
NDP	NM_000266	Norrie disease (pseudoglioma)	-2.5220702	down
PFKL	NM_002626	phosphofructokinase, liver	-4.5982337	down
			-6.0992327	down
NRG1	NM_004495	neuregulin 1	-3.5246947	down
MYLK2	NM_033118	myosin light chain kinase 2	-3.6064427	down
TTLL9	NM_001008409	tubulin tyrosine ligase-like family, member 9	-3.0298066	down
			-3.7075272	down
BEST3	NM_032735	bestrophin 3	-4.591873	down
GSG1	NM_001080554	germ cell associated 1	-4.001791	down
	AK094832		-2.579876	down
FAM27L	NR_028336	family with sequence similarity 27-like	-3.7409658	down
			-3.6939983	down
			-2.902639	down
MLPH	NM_024101	melanophilin	-2.6889625	down
FBXW8	NM_153348	F-box and WD repeat domain containing 8	-2.5005636	down
COL5A1	AK057231	collagen, type V, alpha 1	-4.067668	down
	XM_001725354		-2.533428	down
LMOD3	NM_198271	leiomodin 3 (fetal)	-2.187952	down
			-3.7325935	down
GRAMD2	NM_001012642	GRAM domain containing 2	-2.04428	down

DMC1	NM_007068	DMC1 dosage suppressor of mck1 homolog, meiosis-specific homologous recombination (yeast)	-2.5993614	down
ANKMY1	NM_017844	ankyrin repeat and MYND domain containing 1	-3.5635881	down
GNRHR	NM_000406	gonadotropin-releasing hormone receptor	-2.2511566	down
CDC14C	NR_003595	CDC14 cell division cycle 14 homolog C (S. cerevisiae)	-2.0562453	down
DNM1P46	NR_003260	DNM1 pseudogene 46	-3.9727767	down
MERTK		c-mer proto-oncogene tyrosine kinase	-2.5086474	down
SYNE2	NM_015180	spectrin repeat containing, nuclear envelope 2	-2.7720377	down
PLSCR4	NM_020353	phospholipid scramblase 4	-2.0200844	down
CSF1	NM_000757	colony stimulating factor 1 (macrophage)	-2.3409243	down
YEATS2	AK090720	YEATS domain containing 2	-2.1613255	down
PTGR1	NM_012212	prostaglandin reductase 1	-2.6007707	down
			-3.0213652	down
KCTD1	NM_198991	potassium channel tetramerisation domain containing 1	-2.3955479	down
ATE1	NM_001001976	arginyltransferase 1	-2.19486	down
TMEM174	NM_153217	transmembrane protein 174	-5.9497805	down
FLJ25363	NM_001145553	uncharacterized LOC401082	-2.0607967	down
GRM6		glutamate receptor, metabotropic 6	-4.476083	down
			-3.219986	down
ZNF3	NM_017715	zinc finger protein 3	-4.0247526	down
DBC1	BC021560	deleted in bladder cancer 1	-2.245867	down
C8orf44-SGK3	NM_001204173	C8orf44-SGK3 readthrough	-2.4135082	down
			-2.2181242	down
CRMP1	NM_001014809	collapsin response mediator protein 1	-4.3283453	down
			-2.093263	down
SSTR5	NM_001053	somatostatin receptor 5	-2.121615	down
LOC728208	XR_108453	uncharacterized LOC728208	-4.2462087	down
RGR	BC008094	retinal G protein coupled receptor	-2.877186	down
HAND2	NM_021973	heart and neural crest derivatives expressed 2	-2.0427072	down
PRH2	NM_005042	proline-rich protein HaeIII subfamily 2	-2.0849574	down
	JN120858		-2.7122898	down
FAM48B2	NM_001136233	family with sequence similarity 48, member B2	-3.7483592	down
NPAS1	NM_002517	neuronal PAS domain protein 1	-2.5409536	down
COL6A4P2	NR_027898	collagen, type VI, alpha 4 pseudogene 2	-2.4015937	down
PODNL1	NM_024825	podocan-like 1	-5.9561896	down
DERL3	NM_198440	Der1-like domain family, member 3	-2.358331	down
GPR115	NM_153838	G protein-coupled receptor 115	-2.7746212	down
DND1	NM_194249	dead end homolog 1 (zebrafish)	-2.0638418	down
LOC100129125	AK125712	uncharacterized LOC100129125	-3.7789106	down
CDH16	NM_004062	cadherin 16, KSP-cadherin	-2.9937773	down
LOC100132014	AK092445	uncharacterized LOC100132014	-3.343256	down

LOC644093	XR_132708	hCG2040054	-2.7517478	down
	BC010426		-3.6751964	down
ABCA9	NM_080283	ATP-binding cassette, sub-family A (ABC1), member 9	-2.8953872	down
			-2.0809124	down
			-2.2386808	down
SMCR7L	NM_019008	Smith-Magenis syndrome chromosome region, candidate 7-like	-2.1880252	down
TCL6	NR_028288	T-cell leukemia/lymphoma 6 (non-protein coding)	-3.5812922	down
MMACHC	NM_015506	methylmalonic aciduria (cobalamin deficiency) cblC type, with homocystinuria	-2.6416502	down
FARP1	NM_005766	FERM, RhoGEF (ARHGEF) and pleckstrin domain protein 1 (chondrocyte-derived)	-2.2835436	down
LSM14B	NM_144703	LSM14B, SCD6 homolog B (S. cerevisiae)	-2.4017031	down
			-3.2970088	down
RCL1		RNA terminal phosphate cyclase-like 1	-3.0049343	down
DPP6	NM_001039350	dipeptidyl-peptidase 6	-4.514639	down
CD209	NM_021155	CD209 molecule	-2.4687397	down
FOXP3	NM_014009	forkhead box P3	-2.0533419	down
LOC100270679	NR_038394	uncharacterized LOC100270679	-2.6754577	down
TMEM89	NM_001008269	transmembrane protein 89	-2.730447	down
GALNT9	NM_021808	UDP-N-acetyl-alpha-D-galactosamine:polypeptide N-acetylgalactosaminyltransferase 9 (GalNAc-T9)	-2.3163168	down
SLC19A3	NM_025243	solute carrier family 19, member 3	-2.0669942	down
TMEM235	NM_001204210	transmembrane protein 235	-10.200586	down
			-4.1268883	down
ARHGEF38	NM_017700	Rho guanine nucleotide exchange factor (GEF) 38	-2.9027936	down
PRODH	BC036534	proline dehydrogenase (oxidase) 1	-4.231664	down
GJD4	NM_153368	gap junction protein, delta 4, 40.1kDa	-2.0357091	down
VPS37A	AL834189	vacuolar protein sorting 37 homolog A (S. cerevisiae)	-2.7135425	down
GDNF	NM_001190468	glial cell derived neurotrophic factor	-2.4422011	down
LOC283392	NR_026836	uncharacterized LOC283392	-2.1587815	down
	AW593215		-2.9488413	down
GHRLOS	NR_004431	ghrelin opposite strand/antisense RNA (non-protein coding)	-2.9478412	down
LOC345051	XR_109844	hCG38984	-3.3789592	down
			-2.1716497	down
LOC100144602	NR_034138	uncharacterized LOC100144602	-3.4381387	down
PLEKHG5	NM_198681	pleckstrin homology domain containing, family G (with RhoGef domain) member 5	-2.3795633	down
CHI3L1	NM_001276	chitinase 3-like 1 (cartilage glycoprotein-39)	-2.1526206	down
	DA797466		-4.107947	down
RNF165	NM_152470	ring finger protein 165	-2.897544	down
MORN1	NM_024848	MORN repeat containing 1	-4.779135	down
USHBP1	NM_031941	Usher syndrome 1C binding protein 1	-5.4821634	down
GOLGA6A	NM_001038640	golgin A6 family, member A	-3.289856	down

SCARA5	NM_173833	scavenger receptor class A, member 5 (putative)	-3.1757426	down
ASIP	NM_001672	agouti signaling protein	-3.1825237	down
			-2.0611525	down
SPRR2B	NM_001017418	small proline-rich protein 2B	-2.376864	down
LINC00087	NR_024493	long intergenic non-protein coding RNA 87	-2.7784214	down
PXMP2	BC009836	peroxisomal membrane protein 2, 22kDa	-2.032861	down
AIF1L		allograft inflammatory factor 1-like	-2.1419098	down
FRG2	NM_001005217	FSHD region gene 2	-3.2779982	down
LYZL2	NM_183058	lysozyme-like 2	-2.972997	down
			-3.2739453	down
GREB1	BC071853	growth regulation by estrogen in breast cancer 1	-3.4169838	down
PNMA6A	NM_032882	paraneoplastic antigen like 6A	-2.3811274	down
C8orf37	NM_177965	chromosome 8 open reading frame 37	-2.026593	down
	AJ003549		-3.9372993	down
SLC26A7	NM_052832	solute carrier family 26, member 7	-3.6869466	down
			-3.2151647	down
LOC285972	NR_034033	uncharacterized LOC285972	-2.487889	down
			-5.557467	down
			-2.5298479	down
MPPED1	NM_001044370	metallophosphoesterase domain containing 1	-5.874022	down
DBIL5P2	NR_036635	diazepam binding inhibitor-like 5 pseudogene 2	-3.0367873	down
MED1	NM_004774	mediator complex subunit 1	-4.8572793	down
GUCY2D	NM_000180	guanylate cyclase 2D, membrane (retina-specific)	-2.2980378	down
MGC21881	AK127789	uncharacterized locus MGC21881	-2.4019768	down
	AK026667		-3.575991	down
	CD694834		-6.289534	down
CASS4	NM_001164115	Cas scaffolding protein family member 4	-2.1462932	down
FLJ32224	AK056786	uncharacterized LOC440584	-3.794238	down
BLACE	AY166699	B-cell acute lymphoblastic leukemia expressed	-2.6677935	down
SLC9A7	NM_032591	solute carrier family 9 (sodium/hydrogen exchanger), member 7	-2.1704664	down
CCBE1	NM_133459	collagen and calcium binding EGF domains 1	-2.804313	down
OPN5	NM_181744	opsin 5	-3.137884	down
INADL	AJ001306	InaD-like (Drosophila)	-2.2827427	down
CCDC40	NM_001243342	coiled-coil domain containing 40	-2.201744	down
LOC389602	XR_108709	uncharacterized LOC389602	-2.602459	down
	BX648392		-2.781433	down
LOC440297	NR_033579	chondroitin sulfate proteoglycan 4 pseudogene	-3.6204724	down
CHDH	NM_018397	choline dehydrogenase	-2.5915337	down
NFKBIL1	NM_001144961	nuclear factor of kappa light polypeptide gene enhancer in B-cells inhibitor-like 1	-2.2580805	down
ZNF717	NM_001128223	zinc finger protein 717	-2.0979207	down

TMEM190	NM_139172	transmembrane protein 190	-2.5993617	down
TSGA10	NM_025244	testis specific, 10	-2.0500958	down
	DA992326		-4.113459	down
DEFB121	NM_001171832	defensin, beta 121	-5.5644264	down
LINC00028	NR_024358	long intergenic non-protein coding RNA 28	-2.429259	down
TRIM36	NM_001017397	tripartite motif containing 36	-2.3733952	down
ITIH3	NM_002217	inter-alpha-trypsin inhibitor heavy chain 3	-4.883746	down
TRPC6	NM_004621	transient receptor potential cation channel, subfamily C, member 6	-2.5640135	down
LRIT2	NM_001017924	leucine-rich repeat, immunoglobulin-like and transmembrane domains 2	-2.9736469	down
	BC029571		-3.9679463	down
CCL19	NM_006274	chemokine (C-C motif) ligand 19	-2.143954	down
TRIM53P	NR_028346	tripartite motif containing 53, pseudogene	-2.2950032	down
ARHGEF33	NM_001145451	Rho guanine nucleotide exchange factor (GEF) 33	-4.3038073	down
BPIFC	NM_174932	BPI fold containing family C	-2.704431	down
LOC100130015	NR_027335	5-hydroxyisourate hydrolase pseudogene	-2.9379911	down
ACTR3C	BX640643	ARP3 actin-related protein 3 homolog C (yeast)	-2.0208564	down
PEX26	NM_001199319	peroxisomal biogenesis factor 26	-2.410133	down
EFR3B	NM_014971	EFR3 homolog B (S. cerevisiae)	-2.909654	down
HYAL4	NM_012269	hyaluronoglucosaminidase 4	-2.448535	down
ANGPTL4	NM_139314	angiopoietin-like 4	-2.9992814	down
SLC38A4	NM_018018	solute carrier family 38, member 4	-3.050788	down
C1QA	NM_015991	complement component 1, q subcomponent, A chain	-2.0644414	down
RAB19	NM_001008749	RAB19, member RAS oncogene family	-5.7299867	down
	AK307375		-4.9440103	down
LY6K	NM_017527	lymphocyte antigen 6 complex, locus K	-3.219032	down
	DA858408		-2.912551	down
FAT2	NM_001447	FAT tumor suppressor homolog 2 (Drosophila)	-2.0715022	down
			-2.9836261	down
FLJ40039	AK097358	uncharacterized LOC647662	-7.3407874	down
ASRGL1	NM_001083926	asparaginase like 1	-2.5058482	down
ZFP64	NM_199427	zinc finger protein 64 homolog (mouse)	-2.336841	down
HPSE2	NM_021828	heparanase 2	-2.1212192	down
			-2.7351577	down
PROX2	NM_001243007	prospero homeobox 2	-2.7758756	down
	A25493		-4.0344954	down
LOC100128402	AK124574	uncharacterized LOC100128402	-2.0488076	down
GABRR3	NM_001105580	gamma-aminobutyric acid (GABA) receptor, rho 3	-2.021903	down
SIX1	NM_005982	SIX homeobox 1	-6.201456	down
	AK097701		-6.688405	down
C15orf42	NM_152259	chromosome 15 open reading frame 42	-2.2031682	down
PRR23B	NM_001013650	proline rich 23B	-2.3621576	down

LBH	NM_030915	limb bud and heart development homolog (mouse)	-2.0417814	down
C17orf107	NM_001145536	chromosome 17 open reading frame 107	-2.8604891	down
PROM2	NM_144707	prominin 2	-2.370941	down
ANP32A	AK127498	acidic (leucine-rich) nuclear phosphoprotein 32 family, member A	-2.457489	down
C14orf165	NR_024081	chromosome 14 open reading frame 165	-2.379098	down
LOC100129620	NR_033940	uncharacterized LOC100129620	-2.074432	down
LEPR	NM_001003680	leptin receptor	-2.1868777	down
RDX	DQ916738	radixin	-3.0268786	down
HTATSF1P2	BX648511	HIV-1 Tat specific factor 1 pseudogene 2	-2.7865615	down
SPIRE1	NM_001128626	spire homolog 1 (Drosophila)	-2.4888685	down
LOC283888	NR_037158	uncharacterized LOC283888	-2.1284125	down
			-3.9829996	down
C15orf59	NM_001039614	chromosome 15 open reading frame 59	-3.7594361	down
TNC	NM_002160	tenascin C	-3.2533302	down
MAP1LC3C	NM_001004343	microtubule-associated protein 1 light chain 3 gamma	-2.7137017	down
SDCBP2	NM_080489	syndecan binding protein (syntenin) 2	-4.48174	down
			-2.434551	down
FLJ44385	AK126356	uncharacterized FLJ44385	-2.2554266	down
			-2.793236	down
ETV3	NM_001145312	ets variant 3	-3.0406468	down
LPA	NM_005577	lipoprotein, Lp(a)	-2.0264592	down
			-2.1148758	down
ERC2	NM_015576	ELKS/RAB6-interacting/CAST family member 2	-2.3619015	down
TMEM52	NM_178545	transmembrane protein 52	-5.5612707	down
C1orf49		chromosome 1 open reading frame 49	-2.3776698	down
CYP2G1P	NR_040249	cytochrome P450, family 2, subfamily G, polypeptide 1 pseudogene	-2.275448	down
LOC100289094	BC025775	uncharacterized LOC100289094	-3.3646216	down
			-4.065298	down
PTPRQ	NM_001145026	protein tyrosine phosphatase, receptor type, Q	-3.1092496	down
SEMA6C	NM_030913	sema domain, transmembrane domain (TM), and cytoplasmic domain, (semaphorin) 6C	-5.4617624	down
LOC100130954	NR_034016	uncharacterized LOC100130954	-6.72411	down
			-3.1852295	down
DIO3	NM_001362	deiodinase, iodothyronine, type III	-4.2080483	down
	XM_001129515		-2.254708	down
IFNA4	NM_021068	interferon, alpha 4	-2.1906059	down
			-3.3933635	down
PRAMEF5	NM_001013407	PRAME family member 5	-2.5051455	down
ANO7	NM_001001891	anoctamin 7	-3.3846624	down
LOC100130800	XM_001719118	uncharacterized LOC100130800	-5.701493	down
	XR_109597		-4.041795	down

PTH1R	AK094114	parathyroid hormone 1 receptor	-2.1353686	down
LOC728196	BC021736	uncharacterized LOC728196	-3.0604086	down
CDH23	NM_052836	cadherin-related 23	-3.5766797	down
TP53AIP1	NM_001195195	tumor protein p53 regulated apoptosis inducing protein 1	-5.6300135	down
ZNF521	NM_015461	zinc finger protein 521	-4.9204555	down
STRC	NM_153700	stereocilin	-2.8344922	down
LOC100652869	XM_003403453	uncharacterized LOC100652869	-2.2566817	down
TMEM54	NM_033504	transmembrane protein 54	-3.4771028	down
LOC100128081	NR_034097	uncharacterized LOC100128081	-2.2433896	down
FAM115A	NM_014719	family with sequence similarity 115, member A	-2.120631	down
			-2.7800899	down
C16orf89	NM_152459	chromosome 16 open reading frame 89	-2.626226	down
FBLL1	NR_024356	fibrillarin-like 1	-2.3722076	down
	M27336		-3.7871387	down
C1orf146		chromosome 1 open reading frame 146	-2.549752	down
ELK1	NM_001114123	ELK1, member of ETS oncogene family	-2.2374234	down
FAM157A	NM_001145248	family with sequence similarity 157, member A	-3.2728245	down
SLC45A2	NM_016180	solute carrier family 45, member 2	-2.7646308	down
LOC645206	NR_038840	uncharacterized LOC645206	-2.0339217	down
FSTL3	NM_005860	follistatin-like 3 (secreted glycoprotein)	-3.5463393	down
MRPL38	AK024058	mitochondrial ribosomal protein L38	-2.3964107	down
			-3.3816366	down
			-4.0391006	down
MC5R	NM_005913	melanocortin 5 receptor	-2.533881	down
CELF6	NM_001172684	CUGBP, Elav-like family member 6	-2.6516817	down
LOC100131048	AK125976	uncharacterized LOC100131048	-2.0402286	down
FAM183B	NR_028347	acyloxyacyl hydrolase (neutrophil)	-2.2025957	down
USP37	NM_020935	ubiquitin specific peptidase 37	-2.4631536	down
DDI2	NM_032341	DNA-damage inducible 1 homolog 2 (S. cerevisiae)	-2.230267	down
OR2G2	NM_001001915	olfactory receptor, family 2, subfamily G, member 2	-2.0096073	down
POTEE	NM_001083538	POTE ankyrin domain family, member E	-4.067189	down
			-2.7782009	down
CCDC144A	NM_014695	coiled-coil domain containing 144A	-2.0681257	down
HLA-DPB1		major histocompatibility complex, class II, DP beta 1	-3.4620607	down
	XM_003118626		-2.1988246	down
GRID1	NM_017551	glutamate receptor, ionotropic, delta 1	-3.1634269	down
HNRNPA3	NM_194247	heterogeneous nuclear ribonucleoprotein A3	-2.4045832	down
INSC	NM_001031853	inscuteable homolog (Drosophila)	-2.2724304	down
APOB	BC051278	apolipoprotein B (including Ag(x) antigen)	-2.2254267	down
			-3.0753853	down
LINC00256B	NR_024376	long intergenic non-protein coding RNA 256B	-2.7571151	down

			-5.587338	down
			-3.195272	down
FAM167A	NM_053279	family with sequence similarity 167, member A	-2.0159037	down
LOC146795	AK057377	uncharacterized LOC146795	-5.6406894	down
C9orf84	NM_173521	chromosome 9 open reading frame 84	-2.2580492	down
C2orf43		chromosome 2 open reading frame 43	-2.4308124	down
ZNF577	NR_024181	zinc finger protein 577	-2.9011743	down
BCL9L	NM_182557	B-cell CLL/lymphoma 9-like	-2.2818851	down
	CR627426		-4.066877	down
GAMT	NM_138924	guanidinoacetate N-methyltransferase	-3.1134667	down
	D43770		-3.5790865	down
PYCR1	NM_006907	pyrroline-5-carboxylate reductase 1	-2.8095202	down
LOC100127885	XM_001721771	uncharacterized LOC100127885	-4.0095925	down
IQCA1	NM_024726	IQ motif containing with AAA domain 1	-2.4238458	down
PRODH	NM_016335	proline dehydrogenase (oxidase) 1	-3.0909398	down
CLDN3	NM_001306	claudin 3	-2.3020046	down
PDZRN3	BC014432	PDZ domain containing ring finger 3	-2.4092207	down
FFAR1	NM_005303	free fatty acid receptor 1	-2.0572886	down
ZPBP	NM_007009	zona pellucida binding protein	-2.4437635	down
EXPH5	NM_015065	exophilin 5	-2.3969522	down
			-3.677437	down
			-2.5446966	down
IL24	NM_001185156	interleukin 24	-14.694345	down
AQP12A	NM_198998	aquaporin 12A	-2.184617	down
FLJ41327	AK123321	FLJ41327 protein	-2.5390434	down
	XR_132470		-2.3826046	down
PRAMEF13	NM_001024661	PRAME family member 13	-3.3176877	down
			-2.4075794	down
C21orf88	NR_026543	chromosome 21 open reading frame 88	-3.1465907	down
ZNF502	NM_033210	zinc finger protein 502	-2.3460853	down
	AF116680		-2.1041095	down
PARD3		par-3 partitioning defective 3 homolog (C. elegans)	-3.5658984	down
			-2.1084313	down
ZNF7	AK096025	zinc finger protein 7	-2.479293	down
PTPRN	NM_002846	protein tyrosine phosphatase, receptor type, N	-2.3494031	down
RAP1GAP2	NM_015085	RAP1 GTPase activating protein 2	-2.077722	down
KCNE1	NM_000219	potassium voltage-gated channel, Isk-related family, member 1	-2.353031	down
LRRC9	XR_110295	leucine rich repeat containing 9	-2.3282945	down
GAFA1	AF190748	FGF2-associated protein GAFA1	-3.424499	down
CHSY3	NM_175856	chondroitin sulfate synthase 3	-2.0585327	down
LOC339874	NR_038976	uncharacterized LOC339874	-3.359486	down

C3orf39	NM_032806	chromosome 3 open reading frame 39	-2.050156	down
IGFL2	AK125754	IGF-like family member 2	-2.1914747	down
			-2.1889653	down
			-2.1629236	down
MSLN	NM_005823	mesothelin	-2.3855743	down
SYNGAP1	NM_006772	synaptic Ras GTPase activating protein 1	-2.4489183	down
CADPS2	NM_001167940	Ca++-dependent secretion activator 2	-2.124017	down
SYNE1	NM_033071	spectrin repeat containing, nuclear envelope 1	-2.3212101	down
			-3.853116	down
			-3.0360734	down
DEFB109P1	NR_024044	defensin, beta 109, pseudogene 1	-2.6331418	down
WDR96	NM_025145	WD repeat domain 96	-3.6573246	down
HSPG2	NM_005529	heparan sulfate proteoglycan 2	-2.9772706	down
USP49	AJ586139	ubiquitin specific peptidase 49	-2.0703204	down
SPSB4	NM_080862	splA/ryanodine receptor domain and SOCS box containing 4	-2.0252662	down
	XR_110582		-2.3271604	down
FGD5	NM_152536	FYVE, RhoGEF and PH domain containing 5	-3.1576493	down
SGSM2	AK124883	small G protein signaling modulator 2	-4.649883	down
HNRNPKP3	NR_033868	heterogeneous nuclear ribonucleoprotein K pseudogene 3	-2.1605213	down
			-3.6328452	down
LOC388796	BC012894	uncharacterized LOC388796	-2.437358	down
FLT4	AB209637	fms-related tyrosine kinase 4	-2.8877766	down
RNF216	AK128551	ring finger protein 216	-3.6984475	down
ESRP1	NM_017697	epithelial splicing regulatory protein 1	-2.6823573	down
VSX1	DQ854811	visual system homeobox 1	-3.3461142	down
NRG3	NM_001010848	neuregulin 3	-2.1122186	down
FLJ32742	AK057304	uncharacterized locus FLJ32742	-2.26879	down
STK16	NM_001008910	serine/threonine kinase 16	-5.1239147	down
ZNF792	NM_175872	zinc finger protein 792	-2.0244782	down
LINC00410	NR_027039	long intergenic non-protein coding RNA 410	-3.2167509	down
	AK098530		-2.0207627	down
CD209	NM_021155	CD209 molecule	-4.00072	down
	AK127178		-3.5016816	down
NADKD1	NM_001085411	NAD kinase domain containing 1	-4.36624	down
FAM22A	NM_001099338	family with sequence similarity 22, member A	-3.300521	down
C9orf68	NM_001039395	chromosome 9 open reading frame 68	-2.6702895	down
RHOV	NM_133639	ras homolog gene family, member V	-2.0145276	down
	AK309505		-3.4554362	down
CRYAB	NM_001885	crystallin, alpha B	-2.4514074	down
LY6G5C	NM_025262	lymphocyte antigen 6 complex, locus G5C	-9.029468	down
PSMB11	NM_001099780	proteasome (prosome, macropain) subunit, beta type, 11	-2.034291	down

			-3.7783194	down
LOC100240734	NR_026657	uncharacterized LOC100240734	-2.6423094	down
TAF1L	NM_153809	TAF1 RNA polymerase II, TATA box binding protein (TBP)-associated factor, 210kDa-like	-5.5881977	down
FAM196A	NM_001039762	family with sequence similarity 196, member A	-2.5060225	down
	AF289570		-4.904358	down
C1orf210	NM_182517	chromosome 1 open reading frame 210	-3.2689347	down
KLB	NM_175737	klotho beta	-2.7674081	down
LINC00273	NR_038368	long intergenic non-protein coding RNA 273	-11.5935135	down
LOC348840	NR_003291	uncharacterized LOC348840	-2.1356416	down
TRIM3	NM_006458	tripartite motif containing 3	-2.016826	down
XKR6	NM_173683	XK, Kell blood group complex subunit-related family, member 6	-2.8963418	down
KALRN	NM_003947	kalirin, RhoGEF kinase	-4.946721	down
C21orf7	AY033900	chromosome 21 open reading frame 7	-3.5229876	down
NEBL		nebulette	-2.6540837	down
RPS6KL1	NM_031464	ribosomal protein S6 kinase-like 1	-2.1336749	down
C3orf51	NR_024615	chromosome 3 open reading frame 51	-3.9506292	down
MYLPF	NM_013292	myosin light chain, phosphorylatable, fast skeletal muscle	-2.8975496	down
BHLHE23	NM_080606	basic helix-loop-helix family, member e23	-2.953399	down
MAGEA2B	NM_153488	melanoma antigen family A, 2B	-3.042379	down
GRRP1	NM_024869	glycine/arginine rich protein 1	-2.0276973	down
LOC100130342	BC114619	secretoglobin-like pseudogene	-2.5438118	down
DEFB130	NM_001037804	defensin, beta 130	-2.711161	down
HSPG2	NM_005529	heparan sulfate proteoglycan 2	-3.7379262	down
NPEPPS	AK311414	aminopeptidase puromycin sensitive	-3.2359207	down
TRPM3	NM_001007471	transient receptor potential cation channel, subfamily M, member 3	-2.509547	down
CCBP2	NM_001296	chemokine binding protein 2	-2.5091977	down
LRRIQ3	AK131476	leucine-rich repeats and IQ motif containing 3	-2.322823	down
	AK092228		-2.1858025	down
	AK054980		-2.2069337	down
MAGEA4	NM_002362	melanoma antigen family A, 4	-2.1924	down
MYOD1	NM_002478	myogenic differentiation 1	-7.1367836	down
RBM10	AK098115	RNA binding motif protein 10	-2.5255873	down
SLC28A1	NM_201651	solute carrier family 28 (sodium-coupled nucleoside transporter), member 1	-2.9468334	down
FLJ25917	AK098783	uncharacterized LOC401585	-4.1608076	down
			-2.0808046	down
ANKRD30BP2	NR_026916	ankyrin repeat domain 30B pseudogene 2	-2.7637517	down
KDM2B	AB031230	lysine (K)-specific demethylase 2B	-2.3524652	down
			-2.8000383	down
			-2.2860525	down
UBE2Q2P3	NR_024474	ubiquitin-conjugating enzyme E2Q family member 2 pseudogene 3	-2.3396132	down

	AY927536		-2.0427396	down
DMRT3	NM_021240	doublesex and mab-3 related transcription factor 3	-3.0700583	down
	BC032956		-3.3692966	down
GALP	NM_033106	galanin-like peptide	-2.3076105	down
LCE6A	NM_001128600	late cornified envelope 6A	-2.1669328	down
KCNJ13	NM_002242	potassium inwardly-rectifying channel, subfamily J, member 13	-2.1590295	down
FBXO44	NM_001014765	F-box protein 44	-3.7377772	down
SDK2	NM_001144952	sidekick homolog 2 (chicken)	-2.5141318	down
DEFA7P	AY746432	defensin, alpha 7 pseudogene	-2.4262056	down
			-2.526869	down
			-3.1192315	down
LRRC3C	NM_001195545	leucine rich repeat containing 3C	-2.158264	down
C9orf117	BC133027	chromosome 9 open reading frame 117	-3.2173495	down
LOC442421	NR_024496	uncharacterized LOC442421	-3.0705323	down
			-2.3721492	down
LOC144486	NR_027035	uncharacterized LOC144486	-2.7160625	down
			-2.6186666	down
ATRNL1	NM_207303	attractin-like 1	-2.0279331	down
KCNK7	NM_005714	potassium channel, subfamily K, member 7	-2.4519825	down
L3MBTL2	NM_031488	l(3)mbt-like 2 (Drosophila)	-2.4604206	down
			-2.5506198	down
C19orf73	NM_018111	chromosome 19 open reading frame 73	-2.1735058	down
	CU677870		-2.3017318	down
UCA1	NR_015379	urothelial cancer associated 1 (non-protein coding)	-2.8168876	down
			-4.000587	down
AKNA	NM_030767	AT-hook transcription factor	-5.454889	down
ATP4A	NM_000704	ATPase, H+/K+ exchanging, alpha polypeptide	-2.286891	down
LOC100128402	AK124574	uncharacterized LOC100128402	-2.8967838	down
NOS1AP	NM_014697	nitric oxide synthase 1 (neuronal) adaptor protein	-3.179233	down
	BI056255		-2.2621713	down
DEFB132	NM_207469	defensin, beta 132	-2.4618776	down
SNX31	NM_152628	sorting nexin 31	-2.1604326	down
LOC283501	AK093292	uncharacterized LOC283501	-3.187564	down
SEMA6B	NM_032108	sema domain, transmembrane domain (TM), and cytoplasmic domain, (semaphorin) 6B	-2.0456653	down
PTCHD2	NM_020780	patched domain containing 2	-2.1074114	down
			-5.123204	down
ESPNL	NM_194312	espin-like	-2.7577095	down
PTPRF	BC048416	protein tyrosine phosphatase, receptor type, F	-6.87335	down
SPRR2A	NM_005988	small proline-rich protein 2A	-3.136551	down
ZNF550	NM_001039654	zinc finger protein 550	-2.3173168	down

HYALP1	NR_002731	hyaluronoglucosaminidase pseudogene 1	-4.87453	down
MUC16	NM_024690	mucin 16, cell surface associated	-2.1008573	down
PASD1	NM_173493	PAS domain containing 1	-3.107486	down
	AF289601		-2.6873312	down
			-2.1281114	down
DUOXA1	NM_144565	dual oxidase maturation factor 1	-2.868307	down
	AK096239		-2.659365	down
	AK310283		-2.2905316	down
ANKRD20A5P	BC022023	ankyrin repeat domain 20 family, member A5, pseudogene	-3.3703258	down
GOLGA6L6	NM_001145004	golgin A6 family-like 6	-2.0424128	down
	AK131548		-2.9386191	down
			-2.3039758	down
	BU659163		-2.7154207	down
	XM_001717762		-2.004759	down
KCNQ2	NM_004518	potassium voltage-gated channel, KQT-like subfamily, member 2	-2.6802337	down
			-4.353743	down
OR56A4	NM_001005179	olfactory receptor, family 56, subfamily A, member 4	-3.442417	down
COL9A1	NM_001851	collagen, type IX, alpha 1	-2.5184052	down
MYCBPAP	NM_032133	MYCBP associated protein	-2.5466025	down
KCNV2	NM_133497	potassium channel, subfamily V, member 2	-3.276973	down
PRAMEF3	NM_001013692	PRAME family member 3	-2.3842094	down
ARHGAP36	NM_144967	Rho GTPase activating protein 36	-2.4338784	down
MYCL1	NM_005376	v-myc myelocytomatosis viral oncogene homolog 1, lung carcinoma derived (avian)	-2.5123694	down
POU5F1	NM_002701	POU class 5 homeobox 1	-2.5848484	down
IPO7	NM_006391	importin 7	-2.1834571	down
NYNRIN	NM_025081	NYN domain and retroviral integrase containing	-2.4519982	down
PRG2	NM_002728	proteoglycan 2, bone marrow (natural killer cell activator, eosinophil granule major basic protein)	-2.9523873	down
LOC100132071	AK128253	uncharacterized LOC100132071	-2.3124185	down
FLJ37786	XR_108343	uncharacterized LOC642691	-4.214188	down
FLJ43903	AK125891	uncharacterized LOC401471	-17.184885	down
			-4.050142	down
OR2Y1	NM_001001657	olfactory receptor, family 2, subfamily Y, member 1	-2.3281872	down
			-4.3974977	down
TLX1	NM_005521	T-cell leukemia homeobox 1	-2.0746644	down
SLC30A3	NM_003459	solute carrier family 30 (zinc transporter), member 3	-3.165658	down
BDNF-AS1	NR_002832	BDNF antisense RNA 1 (non-protein coding)	-3.3081436	down
CDH18	NM_004934	cadherin 18, type 2	-3.1158633	down
SGCD	NM_000337	sarcoglycan, delta (35kDa dystrophin-associated glycoprotein)	-5.716335	down
ABCB11	NM_003742	ATP-binding cassette, sub-family B (MDR/TAP), member 11	-2.027744	down
FAM71F1	NM_032599	family with sequence similarity 71, member F1	-2.8818603	down

SZT2	AK131107	seizure threshold 2 homolog (mouse)	-2.162768	down
PRDM15	NM_022115	PR domain containing 15	-2.5705073	down
LOC439990	NR_038464	uncharacterized LOC439990	-2.0570042	down
C8orf80	NM_001010906	chromosome 8 open reading frame 80	-2.86587	down
CYP24A1	NM_000782	cytochrome P450, family 24, subfamily A, polypeptide 1	-2.2484956	down
MUM1	NM_032853	melanoma associated antigen (mutated) 1	-2.1086583	down
			-2.3870096	down
CLIP2	NM_003388	CAP-GLY domain containing linker protein 2	-2.791784	down
PRIC285	NM_001037335	peroxisomal proliferator-activated receptor A interacting complex 285	-6.1510987	down
			-3.532509	down
			-3.6665282	down
SYNDIG1L	NM_001105579	synapse differentiation inducing 1-like	-4.2930007	down
VSX1	NM_014588	visual system homeobox 1	-2.0050478	down
CECR2	AF411609	cat eye syndrome chromosome region, candidate 2	-4.5544186	down
CCIN	NM_005893	calicin	-3.3939779	down
LOC286154	AK096739	uncharacterized LOC286154	-2.5067275	down
LOC100128361	NR_036505	uncharacterized LOC100128361	-2.050467	down
	AK172763		-3.33453	down
			-2.059746	down
RBM14	AB209007	RNA binding motif protein 14	-2.191927	down
ALG1L2	NM_001136152	asparagine-linked glycosylation 1-like 2	-2.4297338	down
SRPX2	NM_014467	sushi-repeat containing protein, X-linked 2	-2.1659663	down
TRIM43	NM_138800	tripartite motif containing 43	-2.4103746	down
LOC147646	NM_001193623	uncharacterized LOC147646	-4.1407604	down
POM121L10P	NR_024593	POM121 membrane glycoprotein-like 10, pseudogene	-2.423049	down
			-4.5689235	down
CPNE7	NM_014427	copine VII	-2.0932019	down
OR4F6	NM_001005326	olfactory receptor, family 4, subfamily F, member 6	-2.498925	down
			-2.6798487	down
LOC283738	NR_038229	uncharacterized LOC283738	-3.1906116	down
HIP1R	AK090537	huntingtin interacting protein 1 related	-3.9575086	down
LOC283270	AK091052	uncharacterized LOC283270	-6.2427974	down
C6orf226	NM_001008739	chromosome 6 open reading frame 226	-2.0317554	down
			-3.0007043	down
GRIK2	NM_001166247	glutamate receptor, ionotropic, kainate 2	-3.7389605	down
PRB1	NM_005039	proline-rich protein BstNI subfamily 1	-2.585292	down
ADAMDEC1	NM_001145271	ADAM-like, decysin 1	-2.8370197	down
			-4.2263117	down
RNF157	NM_052916	ring finger protein 157	-5.696053	down
MAPKBP1	NM_014994	mitogen-activated protein kinase binding protein 1	-2.318445	down
PER3	NM_016831	period homolog 3 (Drosophila)	-2.7912817	down

C10orf129	NM_207321	chromosome 10 open reading frame 129	-3.9808633	down
			-2.2515256	down
CDV3	NM_017548	CDV3 homolog (mouse)	-2.1465824	down
POM121L1P	NR_024591	POM121 membrane glycoprotein-like 1, pseudogene	-2.0600715	down
			-4.2592173	down
OR2G3	NM_001001914	olfactory receptor, family 2, subfamily G, member 3	-2.050878	down
SYT8	NM_138567	synaptotagmin VIII	-2.3531735	down
OR7E37P	NR_002163	olfactory receptor, family 7, subfamily E, member 37 pseudogene	-5.2708173	down
LOC100130741	AK127222	uncharacterized LOC100130741	-3.072275	down
ALDH1L1	NM_012190	aldehyde dehydrogenase 1 family, member L1	-2.478089	down
FAM64A	NM_001195228	family with sequence similarity 64, member A	-4.513279	down
LOC100130285	XR_109235	uncharacterized LOC100130285	-3.3794758	down
GUCA1B	NM_002098	guanylate cyclase activator 1B (retina)	-2.0673616	down
CHRNG	NM_005199	cholinergic receptor, nicotinic, gamma	-4.1138744	down
SEMA6C	NM_001178061	sema domain, transmembrane domain (TM), and cytoplasmic domain, (semaphorin) 6C	-3.451388	down
GOLGA8E	NR_033350	golgin A8 family, member E	-2.189271	down
SUSD4		sushi domain containing 4	-2.1846018	down
NALCN	AK094390	sodium leak channel, non-selective	-3.0156343	down
C12orf50	NM_152589	chromosome 12 open reading frame 50	-3.3261697	down
LRRC52	NM_001005214	leucine rich repeat containing 52	-2.5712633	down
			-3.2312324	down
EBF4	NM_001110514	early B-cell factor 4	-2.5946763	down
GJB7		gap junction protein, beta 7, 25kDa	-2.0394537	down
XAGE-4	XR_109760	XAGE-4 protein	-2.3084831	down
			-2.8844619	down
LOC284412	NR_029390	uncharacterized LOC284412	-2.5562716	down
	DA613845		-3.3073323	down
OR11H6	NM_001004480	olfactory receptor, family 11, subfamily H, member 6	-2.2350492	down
LOC120824	NM_001206625	tripartite motif-containing protein ENSP00000309378-like	-2.4483905	down
DQX1	NM_133637	DEAQ box RNA-dependent ATPase 1	-2.619572	down
GLP2R	NM_004246	glucagon-like peptide 2 receptor	-2.654922	down
AMMECR1	NM_001171689	Alport syndrome, mental retardation, midface hypoplasia and elliptocytosis chromosomal region gene 1	-3.9276004	down
LLGL2	NM_001015002	lethal giant larvae homolog 2 (Drosophila)	-2.3091593	down
KIAA0087	NR_022006	KIAA0087	-7.228069	down
LOC100131581	AK092544	uncharacterized LOC100131581	-2.8613105	down
DRGX	NM_001080520	dorsal root ganglia homeobox	-2.0302548	down
KRTAP12-2	NM_181684	keratin associated protein 12-2	-2.7637467	down
SLC34A2	NM_006424	solute carrier family 34 (sodium phosphate), member 2	-2.3184993	down
			-2.6399198	down
			-2.5514452	down

	XR_109635		-2.7173836	down
	AB529268		-2.2521598	down
ARHGEF1	AK090448	Rho guanine nucleotide exchange factor (GEF) 1	-2.1621234	down
RARRES1	NM_002888	retinoic acid receptor responder (tazarotene induced) 1	-2.3380191	down
PTH1R	NM_000316	parathyroid hormone 1 receptor	-2.3096282	down
			-2.1962855	down
	BC028053		-3.2160168	down
GRM7	NM_181874	glutamate receptor, metabotropic 7	-3.1569126	down
MYH14	NM_001077186	myosin, heavy chain 14, non-muscle	-3.9846447	down
ZNF621	NM_198484	zinc finger protein 621	-3.3787754	down
SNAR-C3	BF570763	small ILF3/NF90-associated RNA C3	-5.299424	down
SPATA13	NM_001166271	spermatogenesis associated 13	-2.9004962	down
			-4.396531	down
WDR64	NM_144625	WD repeat domain 64	-3.1141214	down
SSTR4	NM_001052	somatostatin receptor 4	-2.2098005	down
CHRM2	NM_001006630	cholinergic receptor, muscarinic 2	-2.6866453	down
WFIKKN2	NM_175575	WAP, follistatin/kazal, immunoglobulin, kunitz and netrin domain containing 2	-4.5092516	down
			-2.4341285	down
SLC22A24	NM_001136506	solute carrier family 22, member 24	-2.6459508	down
C17orf104	BC035159	chromosome 17 open reading frame 104	-2.8974319	down
SUSD2	NM_019601	sushi domain containing 2	-2.9208345	down
AKAP12	NM_144497	A kinase (PRKA) anchor protein 12	-2.039172	down
	BX090553		-2.4411469	down
CEACAM7	NM_006890	carcinoembryonic antigen-related cell adhesion molecule 7	-2.2503421	down
DUSP18	NM_152511	dual specificity phosphatase 18	-2.2181687	down
			-2.8659291	down
LOC100130557	NR_024567	uncharacterized LOC100130557	-2.0409	down
CXCL14	NM_004887	chemokine (C-X-C motif) ligand 14	-2.5581894	down
			-2.7704477	down
MEGF8	NM_001410	multiple EGF-like-domains 8	-3.1479897	down
EXD1	NM_152596	exonuclease 3'-5' domain containing 1	-4.3647523	down
			-2.8587263	down
MAML3	NM_018717	mastermind-like 3 (Drosophila)	-15.053548	down
LAMA4	NM_001105207	laminin, alpha 4	-4.858686	down
FIGN		fidgetin	-2.472739	down
FLJ13773	AK023835	FLJ13773	-2.103998	down
NLGN2	NM_020795	neuroligin 2	-2.5072322	down
	BC043411		-2.7703223	down
MUC6	AK092533	mucin 6, oligomeric mucus/gel-forming	-2.2798593	down
STH	NM_001007532	saitohin	-4.316842	down

	AK127966		-2.099907	down
			-2.496098	down
HAAO		3-hydroxyanthranilate 3,4-dioxygenase	-5.9729013	down
DLX6-AS1	NR_015448	DLX6 antisense RNA 1 (non-protein coding)	-2.107493	down
ZNF208	NM_007153	zinc finger protein 208	-2.3921297	down
	AF068294		-4.3153763	down
PLA2G1B	NM_000928	phospholipase A2, group IB (pancreas)	-3.544727	down
GNAL	NM_002071	guanine nucleotide binding protein (G protein), alpha activating activity polypeptide, olfactory type	-2.8628473	down
PPP6R2	AK024148	protein phosphatase 6, regulatory subunit 2	-5.953941	down
UGT2B7	NM_001074	UDP glucuronosyltransferase 2 family, polypeptide B7	-4.2031784	down
DBX1	NM_001029865	developing brain homeobox 1	-2.674685	down
BUB1B	NM_001211	budding uninhibited by benzimidazoles 1 homolog beta (yeast)	-4.010965	down
KCNK9	NM_016601	potassium channel, subfamily K, member 9	-3.212436	down
ATP1B2	NM_001678	ATPase, Na+/K+ transporting, beta 2 polypeptide	-2.2162814	down
DDB1		damage-specific DNA binding protein 1, 127kDa	-2.6218011	down
LZTS1	NM_021020	leucine zipper, putative tumor suppressor 1	-2.11839	down
HN1L	NM_144570	hematological and neurological expressed 1-like	-3.0376556	down
TEF	NM_003216	thyrotrophic embryonic factor	-2.6184251	down
			-3.8491814	down
IMP5	NM_175882	intramembrane protease 5	-3.3487146	down
CLEC6A	NM_001007033	C-type lectin domain family 6, member A	-3.0251372	down
PHKA1	NM_002637	phosphorylase kinase, alpha 1 (muscle)	-2.113557	down
GAK	AK131464	cyclin G associated kinase	-2.2029355	down
C12orf48	NM_017915	chromosome 12 open reading frame 48	-7.67942	down
TRIM61	NM_001012414	tripartite motif containing 61	-2.1925528	down
			-2.2568557	down
LMX1B	NM_002316	LIM homeobox transcription factor 1, beta	-2.5086687	down
PRM2	NM_002762	protamine 2	-2.4658096	down
PNMA6C	NM_001170944	paraneoplastic antigen like 6C	-2.9828613	down
FSIP2	NM_173651	fibrous sheath interacting protein 2	-2.5490985	down
	DB453801		-2.191657	down
PFKFB3	NM_004566	6-phosphofructo-2-kinase/fructose-2,6-biphosphatase 3	-2.1264164	down
			-2.3124542	down
LOC729032	XR_132715	ribosomal protein L36 pseudogene	-2.1807523	down
TWIST1	NM_000474	twist homolog 1 (Drosophila)	-2.3576634	down
CELA3A	NM_005747	chymotrypsin-like elastase family, member 3A	-2.2357085	down
TCEB3C	NM_145653	transcription elongation factor B polypeptide 3C (elongin A3)	-8.311952	down
NRG2	AK124504	neuregulin 2	-2.7202995	down
C6orf201	NM_001085401	chromosome 6 open reading frame 201	-2.1229281	down
ADAMTSL1	NM_052866	ADAMTS-like 1	-2.3660681	down

			-2.5416183	down
CIDEA	NM_001279	cell death-inducing DFFA-like effector a	-5.287442	down
CHD5	NM_015557	chromodomain helicase DNA binding protein 5	-2.7657988	down
KRTAP10-4	NM_198687	keratin associated protein 10-4	-4.6243157	down
			-2.7799406	down
KIF1C	NM_006612	kinesin family member 1C	-2.0738742	down
LMOD2	NM_207163	leiomodin 2 (cardiac)	-2.570554	down
C19orf20	NM_033513	chromosome 19 open reading frame 20	-2.420084	down
NPL	BC034966	N-acetylneuraminate pyruvate lyase (dihydrodipicolinate synthase)	-2.38428	down
ZNF783	AK303492	zinc finger family member 783	-3.6931887	down
C17orf69	NR_026906	chromosome 17 open reading frame 69	-2.249554	down
OVOL2	NM_021220	ovo-like 2 (Drosophila)	-3.4602869	down
CXorf1	NM_004709	chromosome X open reading frame 1	-2.5168185	down
			-3.6310387	down
DEFB136	NM_001033018	defensin, beta 136	-2.5463066	down
GDPD5	NM_030792	glycerophosphodiester phosphodiesterase domain containing 5	-2.1044037	down
CDRT8	AI150443	CMT1A duplicated region transcript 8	-3.3121119	down
SH3PXD2B	NM_001017995	SH3 and PX domains 2B	-2.0006588	down
ALOX12P2	NR_002710	arachidonate 12-lipoxygenase pseudogene 2	-3.0843325	down
NCR1	NM_004829	natural cytotoxicity triggering receptor 1	-2.14373	down
	BG722090		-2.2055364	down
MRP63	NM_024026	mitochondrial ribosomal protein 63	-2.5852232	down
LOC100190939	NR_024458	uncharacterized LOC100190939	-2.055494	down
NTF4	NM_006179	neurotrophin 4	-2.2685297	down
FADS6	NM_178128	fatty acid desaturase domain family, member 6	-5.106219	down
			-2.7674096	down
			-2.5182302	down
	AK074630		-3.5783901	down
ZBTB12	NM_181842	zinc finger and BTB domain containing 12	-2.2155778	down
CRYBB2P1	NR_033733	crystallin, beta B2 pseudogene 1	-2.578034	down
AGMO	NM_001004320	alkylglycerol monooxygenase	-2.9283755	down
SLC13A4	NM_012450	solute carrier family 13 (sodium/sulfate symporters), member 4	-2.5506876	down
BRSK1	NM_032430	BR serine/threonine kinase 1	-3.3366559	down
	CU677925		-2.1556482	down
NPW	NM_001099456	neuropeptide W	-2.8928888	down
CLCN5	NM_001127899	chloride channel 5	-3.778924	down
PKD1	NM_001009944	polycystic kidney disease 1 (autosomal dominant)	-2.2537096	down
LMX1A	NM_177398	LIM homeobox transcription factor 1, alpha	-2.7074177	down
GJA5	NM_005266	gap junction protein, alpha 5, 40kDa	-2.4397182	down
TNNI3	NM_000363	troponin I type 3 (cardiac)	-2.0047987	down
			-3.1679533	down

LOC151760	XR_108461	uncharacterized LOC151760	-2.2375312	down
GNAO1	NM_138736	guanine nucleotide binding protein (G protein), alpha activating activity polypeptide O	-2.0468462	down
OR4F4	NM_001004195	olfactory receptor, family 4, subfamily F, member 4	-2.2719133	down
STOX2	NM_020225	storkhead box 2	-2.6534352	down
C15orf55	NM_175741	chromosome 15 open reading frame 55	-3.0860043	down
LOC100130071	XR_109863	GSQS6193	-2.1335576	down
VSIG2	NM_014312	V-set and immunoglobulin domain containing 2	-2.8821256	down
PLA2R1	NM_007366	phospholipase A2 receptor 1, 180kDa	-2.0699399	down
LRP11		low density lipoprotein receptor-related protein 11	-2.8726544	down
			-3.8446653	down
DNAAF2	NM_001083908	dynein, axonemal, assembly factor 2	-2.1570506	down
LOC100287225	NR_040075	uncharacterized LOC100287225	-2.439124	down
GVINP1	NR_003945	GTPase, very large interferon inducible pseudogene 1	-2.1592197	down
HOMER1		homer homolog 1 (Drosophila)	-3.095869	down
ROBO2	NM_002942	roundabout, axon guidance receptor, homolog 2 (Drosophila)	-4.242307	down
PALM	NM_002579	paralemmin	-2.1597066	down
LOC100131792	AK093561	uncharacterized LOC100131792	-2.9254284	down
TPRXL	NR_002223	tetra-peptide repeat homeobox-like	-2.2759128	down
	XR_132758		-3.2619371	down
GLI2	NM_005270	GLI family zinc finger 2	-2.0886974	down
TGM7	NM_052955	transglutaminase 7	-2.6584141	down
ZNF749	NM_001023561	zinc finger protein 749	-2.2963426	down
FGF7	NM_002009	fibroblast growth factor 7	-3.1798081	down
LOC100129518	NR_037166	uncharacterized LOC100129518	-3.0518596	down
VWA5B2	NM_138345	von Willebrand factor A domain containing 5B2	-2.3341541	down
			-2.6388333	down
	DB229023		-2.2753153	down
MUC22	NM_001198815	mucin 22	-2.1166081	down
NAG20	XR_108823	NAG20	-10.061569	down
C21orf104	AJ011409	chromosome 21 open reading frame 104	-3.939667	down
ASB18	NM_212556	ankyrin repeat and SOCS box containing 18	-2.563251	down
	AK129565		-4.287017	down
MPP2	NM_005374	membrane protein, palmitoylated 2 (MAGUK p55 subfamily member 2)	-2.9756818	down
SOX10	NM_006941	SRY (sex determining region Y)-box 10	-2.019094	down
LACTBL1	XM_002342035	lactamase, beta-like 1	-2.4899201	down
FLJ46361	NR_003570	deleted in malignant brain tumors 1 pseudogene	-2.379761	down
LOC650293	NM_001040071	seven transmembrane helix receptor	-3.1572661	down
ACSS3	NM_024560	acyl-CoA synthetase short-chain family member 3	-2.8812094	down
	AF384996		-3.1008523	down
P4HA2	NM_001017973	prolyl 4-hydroxylase, alpha polypeptide II	-2.0589087	down

	AK128101		-3.4896889	down
VIPR2	NM_003382	vasoactive intestinal peptide receptor 2	-2.1175394	down
RHBDL2	NM_017821	rhomboid, veinlet-like 2 (Drosophila)	-2.0552807	down
OR1S2	NM_001004459	olfactory receptor, family 1, subfamily S, member 2	-2.427515	down
SGK110	NM_001199824	putative uncharacterized serine/threonine-protein kinase SgK110-like	-2.0057232	down
BCL2L10	NM_020396	BCL2-like 10 (apoptosis facilitator)	-5.0572643	down
LINC00235	NR_024121	long intergenic non-protein coding RNA 235	-2.086108	down
	X57818		-3.4062817	down
LOC100128498	AK126742	uncharacterized LOC100128498	-2.821145	down
FAM132B	XM_003119882	family with sequence similarity 132, member B	-3.4506288	down
HORMAD2	NM_152510	HORMA domain containing 2	-3.3447893	down
RBM33	NM_053043	RNA binding motif protein 33	-2.5258825	down
			-3.0081782	down
CDX1	NM_001804	caudal type homeobox 1	-2.900254	down
			-3.316415	down
TXLNB		taxilin beta	-2.9084172	down
TDRD6	NM_001010870	tudor domain containing 6	-2.0006807	down
LINC00163	NR_033840	long intergenic non-protein coding RNA 163	-2.4099782	down
GLT25D2	NM_015101	glycosyltransferase 25 domain containing 2	-2.9312768	down
LOC440330	BC004968	uncharacterized LOC440330	-14.663246	down
CATSPER2		cation channel, sperm associated 2	-5.412927	down
FAM9B	NM_205849	family with sequence similarity 9, member B	-2.0680704	down
PHKG1	NM_006213	phosphorylase kinase, gamma 1 (muscle)	-3.820593	down
VSX1	NM_199425	visual system homeobox 1	-2.3420432	down
	BC015443		-3.1130645	down
SYPL2	NM_001040709	synaptophysin-like 2	-5.0311785	down
PTPRC	NM_002838	protein tyrosine phosphatase, receptor type, C	-2.4676294	down
PVRL4	NM_030916	poliovirus receptor-related 4	-2.3491104	down
			-3.6696165	down
			-3.500627	down
SPRR2D	NM_006945	small proline-rich protein 2D	-2.2251267	down
FLJ13224	NR_026806	uncharacterized LOC79857	-3.3484845	down
SPATA12	NM_181727	spermatogenesis associated 12	-2.4145296	down
CELSR1	NM_014246	cadherin, EGF LAG seven-pass G-type receptor 1 (flamingo homolog, Drosophila)	-2.0354974	down
GRIP1	NM_021150	glutamate receptor interacting protein 1	-3.6491559	down
FGF3	NM_005247	fibroblast growth factor 3	-13.7881565	down
BCL2	NM_000657	B-cell CLL/lymphoma 2	-2.037091	down
ADCY1	NM_021116	adenylate cyclase 1 (brain)	-3.6959312	down
MRPS16	NM_016065	mitochondrial ribosomal protein S16	-2.248775	down
GPR113	NM_153835	G protein-coupled receptor 113	-2.231668	down

			-2.9753563	down
CSNK1E	NM_152221	casein kinase 1, epsilon	-2.6681604	down
CPN2	NM_001080513	carboxypeptidase N, polypeptide 2	-3.211302	down
			-3.5383708	down
HOXA7	NM_006896	homeobox A7	-3.1903841	down
FAM48B1	NM_001136234	family with sequence similarity 48, member B1	-4.0409393	down
DNAL4		dynein, axonemal, light chain 4	-4.13544	down
NBR2	NR_003108	neighbor of BRCA1 gene 2 (non-protein coding)	-2.2720811	down
ELAVL4	NM_021952	ELAV (embryonic lethal, abnormal vision, Drosophila)-like 4 (Hu antigen D)	-2.26844	down
			-2.275369	down
RGS21	NM_001039152	regulator of G-protein signaling 21	-3.2906437	down
EML2	NM_012155	echinoderm microtubule associated protein like 2	-2.432665	down
LOC100272216	NR_027439	uncharacterized LOC100272216	-3.7766654	down
			-2.5882592	down
PPAPDC1A	NM_001030059	phosphatidic acid phosphatase type 2 domain containing 1A	-2.0184798	down
MYL10	NM_138403	myosin, light chain 10, regulatory	-2.9560215	down
DPM3	NM_018973	dolichyl-phosphate mannosyltransferase polypeptide 3	-2.4301288	down
SYTL5	NM_138780	synaptotagmin-like 5	-2.0588367	down
AWAT2	NM_001002254	acyl-CoA wax alcohol acyltransferase 2	-2.237796	down
			-6.600277	down
ANP32A-IT1	NR_026808	ANP32A intronic transcript 1 (non-protein coding)	-2.7897952	down
DST	NM_001723	dystonin	-3.4785645	down
TP53INP1	NM_033285	tumor protein p53 inducible nuclear protein 1	-2.3441136	down
LOC100128531	NR_038941	uncharacterized LOC100128531	-3.5386827	down
			-2.1866016	down
	S60780		-2.0055678	down
HOPX	NM_001145460	HOP homeobox	-2.0832694	down
CBLN4	NM_080617	cerebellin 4 precursor	-3.8885832	down
CFHR4	NM_006684	complement factor H-related 4	-2.4945016	down
KIAA1486	NM_020864	KIAA1486	-2.1306896	down
	AK094885		-2.02908	down
SLC13A3	NM_001193339	solute carrier family 13 (sodium-dependent dicarboxylate transporter), member 3	-2.5012276	down
CNTN4	NM_175613	contactin 4	-2.3370209	down
LOC84856	NR_026827	uncharacterized LOC84856	-2.203354	down
LRRC71	NM_144702	leucine rich repeat containing 71	-2.2184744	down
C1orf101	NM_173807	chromosome 1 open reading frame 101	-2.1297073	down
			-2.8130136	down
RFPL3-AS1	NR_001450	RFPL3 antisense RNA 1 (non-protein coding)	-2.4599001	down
PYY	NM_004160	peptide YY	-2.8745625	down
NELF		nasal embryonic LHRH factor	-2.5614614	down

	BX106347		-4.6487794	down
			-2.4754813	down
CCDC64B	NM_001103175	coiled-coil domain containing 64B	-8.239313	down
PDZD7	NM_001195263	PDZ domain containing 7	-2.757152	down
SORCS3	NM_014978	sortilin-related VPS10 domain containing receptor 3	-4.1854196	down
LOC100129027	NR_038876	uncharacterized LOC100129027	-2.3324537	down
CDC27	NM_001114091	cell division cycle 27 homolog (S. cerevisiae)	-3.5207305	down
LOC400682	XM_001726878	zinc finger protein 100-like	-2.0223794	down
OR52K2	NM_001005172	olfactory receptor, family 52, subfamily K, member 2	-2.5208087	down
PSMD10		proteasome (prosome, macropain) 26S subunit, non-ATPase, 10	-4.215822	down
ZNF160	BC000807	zinc finger protein 160	-2.535495	down
KCNK13	NM_022054	potassium channel, subfamily K, member 13	-4.140283	down
STAM	NM_003473	signal transducing adaptor molecule (SH3 domain and ITAM motif) 1	-3.2446482	down
MPZ	NM_000530	myelin protein zero	-2.3164196	down
TMEM231	NM_001077419	transmembrane protein 231	-2.027912	down
			-4.506873	down
IL1RAPL1	NM_014271	interleukin 1 receptor accessory protein-like 1	-4.7195106	down
AKD1	AK124171	adenylate kinase domain containing 1	-2.4501972	down
C14orf135	AK095489	chromosome 14 open reading frame 135	-3.2413363	down
FLNC	NM_001458	filamin C, gamma	-2.333547	down
GRK1	NM_002929	G protein-coupled receptor kinase 1	-2.9543786	down
C17orf72	NM_001164257	chromosome 17 open reading frame 72	-4.932738	down
LOC286238	NM_001100111	uncharacterized LOC286238	-3.359222	down
CFL1P1	NR_028492	cofilin 1 (non-muscle) pseudogene 1	-4.1626306	down
SCARNA7	NR_003001	small Cajal body-specific RNA 7	-2.134681	down
LOC100128869	XM_001719518	uncharacterized LOC100128869	-14.568469	down
ST8SIA3	NM_015879	ST8 alpha-N-acetyl-neuraminide alpha-2,8-sialyltransferase 3	-2.969064	down
C7orf13	NR_026865	chromosome 7 open reading frame 13	-3.7078924	down
SUSD2	NM_019601	sushi domain containing 2	-3.070956	down
			-4.772648	down
			-6.020332	down
SNAI2	NM_003068	snail homolog 2 (Drosophila)	-3.1396892	down
LOC651337	XR_132727	uncharacterized LOC651337	-2.0341203	down
TSSK3	NM_052841	testis-specific serine kinase 3	-2.4604442	down
KCNQ1DN	NR_024627	KCNQ1 downstream neighbor (non-protein coding)	-2.7120717	down
DAAM2	BC078153	dishevelled associated activator of morphogenesis 2	-3.612623	down
OSTBETA	NM_178859	organic solute transporter beta	-3.0954852	down
DDX11	NM_030653	DEAD/H (Asp-Glu-Ala-Asp/His) box polypeptide 11	-2.3358989	down
UPK3A	NM_006953	uroplakin 3A	-4.2959294	down
OPALIN	NM_001040103	oligodendrocytic myelin paranodal and inner loop protein	-2.941597	down
HNRNPU-AS1	NR_026778	HNRNPU antisense RNA 1 (non-protein coding)	-2.758113	down

LOC100129119	XR_109159	uncharacterized LOC100129119	-4.649454	down
ADAMTS7	NM_014272	ADAM metallopeptidase with thrombospondin type 1 motif, 7	-2.1989279	down
KRTAP5-3	NM_001012708	keratin associated protein 5-3	-3.4099417	down
SPTBN5	NM_016642	spectrin, beta, non-erythrocytic 5	-2.499378	down
			-3.400303	down
CCDC166	NM_001162914	coiled-coil domain containing 166	-2.1435456	down
C2orf61	NM_173649	chromosome 2 open reading frame 61	-4.651796	down
KCNT1	AK123276	potassium channel, subfamily T, member 1	-3.4384925	down
	AK093210		-3.3209505	down
CEACAM5	NM_004363	carcinoembryonic antigen-related cell adhesion molecule 5	-2.1425428	down
C19orf26	NM_152769	chromosome 19 open reading frame 26	-2.3175488	down
KCTD5	NM_018992	potassium channel tetramerisation domain containing 5	-2.0377405	down
			-3.2922227	down
			-2.3078423	down
DKFZp686O1327	NR_033870	uncharacterized LOC401014	-2.6219852	down
NEURL1B	NM_001142651	neuralized homolog 1B (Drosophila)	-2.0261936	down
CD164L2		CD164 sialomucin-like 2	-2.5628273	down
			-4.1499066	down
LOC100130193	AK096102	uncharacterized LOC100130193	-4.886785	down
ADAMTSL1	NM_001040272	ADAMTS-like 1	-2.7540135	down
NEO1	NM_002499	neogenin 1	-2.2549496	down
SLC35F1	NM_001029858	solute carrier family 35, member F1	-2.722175	down
PLAC8L1	NM_001029869	PLAC8-like 1	-2.2947977	down
			-2.1359377	down
MMP24	NM_006690	matrix metallopeptidase 24 (membrane-inserted)	-2.19764	down
NFIB	NM_005596	nuclear factor I/B	-2.010619	down
BTBD9	NM_052893	BTB (POZ) domain containing 9	-3.1878254	down
	AY203961		-2.5608068	down
C1orf86	AK054818	chromosome 1 open reading frame 86	-4.3577604	down
			-4.784889	down
GAST	NM_000805	gastrin	-4.0819	down
MGC34800	XR_110135	uncharacterized protein MGC34800	-2.3042676	down
	AK128007		-4.192814	down
	AK127623		-2.6761942	down
USP2	NM_004205	ubiquitin specific peptidase 2	-2.1656077	down
LOC284930	AK093107	uncharacterized LOC284930	-2.3239396	down
TUBG2	NM_016437	tubulin, gamma 2	-3.9754875	down
			-2.9490306	down
MEP1A	NM_005588	meprin A, alpha (PABA peptide hydrolase)	-4.245633	down
SDC3	NM_014654	syndecan 3	-2.348735	down

PTPRK	NM_002844	protein tyrosine phosphatase, receptor type, K	-2.455539	down
MATN1	NM_002379	matrilin 1, cartilage matrix protein	-2.4572616	down
BET3L	AK002042	BET3 like (S. cerevisiae)	-2.6196525	down
NPVF	NM_022150	neuropeptide VF precursor	-2.2994633	down
FCRL5	NM_001195388	Fc receptor-like 5	-5.0250344	down
	AK026419		-2.4700818	down
			-2.9643085	down
LOC100131195	AK097743	uncharacterized LOC100131195	-4.797982	down
CLSTN3	NM_014718	calsyntenin 3	-3.9137201	down
			-3.0460346	down
LEPROTL1	NM_001128208	leptin receptor overlapping transcript-like 1	-4.4890656	down
			-2.522849	down
			-5.2356024	down
			-2.2561421	down
GLRA4	NM_001024452	glycine receptor, alpha 4	-2.072405	down
RTKN	NM_033046	rhotekin	-2.2317336	down
			-4.5816	down
DOK7	AK075037	docking protein 7	-4.1738076	down
BIRC7	NM_022161	baculoviral IAP repeat containing 7	-3.8651798	down
ZDHHC9	NM_016032	zinc finger, DHHC-type containing 9	-2.7043614	down
CHRNA2	NM_000742	cholinergic receptor, nicotinic, alpha 2 (neuronal)	-4.3793488	down
			-2.3732464	down
IGFALS	NM_004970	insulin-like growth factor binding protein, acid labile subunit	-3.3129983	down
			-3.6051116	down
LOC388882	XR_109751	uncharacterized LOC388882	-3.3496366	down
HPX-2	X74861	homeobox HPX-2	-3.8764646	down
AADACL3	NM_001103170	arylacetamide deacetylase-like 3	-2.3056881	down
			-4.3805804	down
GPR52	NM_005684	G protein-coupled receptor 52	-2.719857	down
UNC45A	NM_001039675	unc-45 homolog A (C. elegans)	-2.9157367	down
ANKRD63	NM_001190479	ankyrin repeat domain 63	-3.1105874	down
LOC401176	XR_108565	uncharacterized LOC401176	-2.3629441	down
	XR_132593		-2.2071903	down
C17orf88	NR_026770	chromosome 17 open reading frame 88	-2.3708618	down
TNRC6C	NM_001142640	trinucleotide repeat containing 6C	-3.187599	down
CDRT1	NM_006382	CMT1A duplicated region transcript 1	-2.9550984	down
			-2.8204217	down
PRDM15	NM_022115	PR domain containing 15	-2.4915326	down
	XR_110564		-2.68227	down
IP6K3	NM_054111	inositol hexakisphosphate kinase 3	-2.1338544	down
SETMAR	NM_006515	SET domain and mariner transposase fusion gene	-3.6181812	down

NGF	NM_002506	nerve growth factor (beta polypeptide)	-2.1390355	down
DDX31	NM_138620	DEAD (Asp-Glu-Ala-Asp) box polypeptide 31	-2.0697954	down
OVOS	XM_003403834	ovostatin	-3.0591376	down
NANOS2	NM_001029861	nanos homolog 2 (Drosophila)	-3.2480094	down
LOC100130417	NR_026874	uncharacterized LOC100130417	-2.8546896	down
LOC649201	XM_001127211	paraneoplastic antigen like 6A-like	-2.022891	down
WFS1	NM_006005	Wolfram syndrome 1 (wolframin)	-2.50929	down
PRR25	NM_001013638	proline rich 25	-2.71824	down
TRIM48	NM_024114	tripartite motif containing 48	-3.0246205	down
KIAA1644	NM_001099294	KIAA1644	-2.8583632	down
SERHL2	NM_014509	serine hydrolase-like 2	-2.3131166	down
PRPF18	CU692621	PRP18 pre-mRNA processing factor 18 homolog (S. cerevisiae)	-2.4917176	down
HMGB3	NM_005342	high mobility group box 3	-2.2783196	down
	AK127494		-3.993354	down
SETBP1	NM_001130110	SET binding protein 1	-2.84234	down
FBXL22	NM_203373	F-box and leucine-rich repeat protein 22	-3.2706625	down
			-2.1899219	down
A4GALT	NM_017436	alpha 1,4-galactosyltransferase	-4.01797	down
PHGR1	NM_001145643	proline/histidine/glycine-rich 1	-2.3261058	down
LOC729159	XM_001129515	UPF0607 protein ENSP00000381418-like	-4.1811337	down
LOC100134868	NR_004846	uncharacterized LOC100134868	-2.9018774	down
ADARB2	AK289895	adenosine deaminase, RNA-specific, B2	-2.1572196	down
SLC22A13	NM_004256	solute carrier family 22 (organic anion transporter), member 13	-8.378844	down
KLHL14	BC021267	kelch-like 14 (Drosophila)	-2.8605113	down
FAM151A	NM_176782	family with sequence similarity 151, member A	-2.6073656	down
MAP4	NM_030885	microtubule-associated protein 4	-3.695181	down
SCT	NM_021920	secretin	-2.1631072	down
SNORA26	AI792523	small nucleolar RNA, H/ACA box 26	-3.5881493	down
ATAD3C	NM_001039211	ATPase family, AAA domain containing 3C	-2.9793437	down
DNASE1	NM_005223	deoxyribonuclease I	-2.3602126	down
GATA4	NM_002052	GATA binding protein 4	-2.032713	down
FRG2C	NM_001124759	FSHD region gene 2 family, member C	-4.7455025	down
	BC015643		-2.6952899	down
SEMA3G	NM_020163	sema domain, immunoglobulin domain (Ig), short basic domain, secreted, (semaphorin) 3G	-2.105367	down
CPAMD8	NM_015692	C3 and PZP-like, alpha-2-macroglobulin domain containing 8	-2.3752642	down
HM13	NM_178582	histocompatibility (minor) 13	-4.8412814	down
			-3.7661602	down
			-2.5732157	down
SNORA80	DB304787	small nucleolar RNA, H/ACA box 80	-4.2207484	down
ZNF720		zinc finger protein 720	-2.0350087	down

PDE1C	AK126467	phosphodiesterase 1C, calmodulin-dependent 70kDa	-2.7776697	down
			-3.7098117	down
			-3.0234857	down
PDLIM4	NM_003687	PDZ and LIM domain 4	-3.443663	down
GLTPD1	NM_001029885	glycolipid transfer protein domain containing 1	-4.349214	down
			-3.0453532	down
GALNTL6	NM_001034845	UDP-N-acetyl-alpha-D-galactosamine:polypeptide N-acetylgalactosaminyltransferase-like 6	-2.4416559	down
LOC100128946	NR_038944	uncharacterized LOC100128946	-2.120539	down
C10orf91	NM_173541	chromosome 10 open reading frame 91	-2.202203	down
			-2.1661677	down
SH3GL3	AF036272	SH3-domain GRB2-like 3	-3.3240488	down
GABRE	NM_004961	gamma-aminobutyric acid (GABA) A receptor, epsilon	-4.76355	down
LOC284240	AL390159	uncharacterized LOC284240	-2.0733082	down
BRD7P3	NR_002730	bromodomain containing 7 pseudogene 3	-3.635799	down
ZNF329	AK090893	zinc finger protein 329	-2.483955	down
LOC441179	XR_112948	uncharacterized LOC441179	-2.3863375	down
			-2.5900695	down
CA6	NM_001215	carbonic anhydrase VI	-2.0829291	down
C5orf20	NM_130848	chromosome 5 open reading frame 20	-2.086026	down
FZD4	NM_012193	frizzled family receptor 4	-2.6502554	down
			-2.7675278	down
OLFML3	NM_020190	olfactomedin-like 3	-2.7830617	down
SH3BP5L	AK125138	SH3-binding domain protein 5-like	-4.087898	down
TECRL	NM_001010874	trans-2,3-enoyl-CoA reductase-like	-3.893835	down
MAGIX	AK025340	MAGI family member, X-linked	-2.5316963	down
LOC541473	NR_003602	FK506 binding protein 6, 36kDa pseudogene	-2.1551108	down
SLC2A6	NM_017585	solute carrier family 2 (facilitated glucose transporter), member 6	-2.6380157	down
MDM2	NM_002392	Mdm2 p53 binding protein homolog (mouse)	-2.5344481	down
CNGA3	NM_001298	cyclic nucleotide gated channel alpha 3	-2.9793782	down
AP1S1	NM_001283	adaptor-related protein complex 1, sigma 1 subunit	-3.3726618	down
SLC18A1	NM_001142325	solute carrier family 18 (vesicular monoamine), member 1	-3.7324808	down
HOXA5	NM_019102	homeobox A5	-2.3764973	down
DNAJB3	NM_001001394	DnaJ (Hsp40) homolog, subfamily B, member 3	-4.1020045	down
CELF1	NM_198700	CUGBP, Elav-like family member 1	-2.375127	down
PTGER1	NM_000955	prostaglandin E receptor 1 (subtype EP1), 42kDa	-5.4921627	down
CAPN5	NM_004055	calpain 5	-2.5296283	down
			-2.2140472	down
	CR979835		-3.381196	down
			-3.1748285	down
LOC399744	NR_024497	uncharacterized LOC399744	-3.1626635	down

			-2.0138168	down
POLR2H	CR590527	polymerase (RNA) II (DNA directed) polypeptide H	-2.8910644	down
NYNRIN	NM_025081	NYN domain and retroviral integrase containing	-2.9919755	down
			-4.1788774	down
ACTL7B	NM_006686	actin-like 7B	-2.1480823	down
LOC441493	XR_113304	uncharacterized LOC441493	-2.3391201	down
KRTAP4-5	NM_033188	keratin associated protein 4-5	-2.0711207	down
RGL3	NM_001161616	ral guanine nucleotide dissociation stimulator-like 3	-2.3689911	down
RUNX2	NM_001015051	runt-related transcription factor 2	-5.1165733	down
OR5W2	NM_001001960	olfactory receptor, family 5, subfamily W, member 2	-2.1485484	down
CHIC1	NM_001039840	cysteine-rich hydrophobic domain 1	-2.7092931	down
DNAJC14	NM_032364	DnaJ (Hsp40) homolog, subfamily C, member 14	-2.3243752	down
RLTPR	NM_001013838	RGD motif, leucine rich repeats, tropomodulin domain and proline-rich containing	-2.5516832	down
NPPB	NM_002521	natriuretic peptide B	-2.1878586	down
LOC284242	BC035844	uncharacterized LOC284242	-5.0251207	down
LOC442421	NR_024496	uncharacterized LOC442421	-3.8294752	down
SCN4B	NM_174934	sodium channel, voltage-gated, type IV, beta	-4.41341	down
	XR_110674		-2.8677733	down
POLE2	NM_002692	polymerase (DNA directed), epsilon 2 (p59 subunit)	-2.2603612	down
ANKRD20A2	NM_001012421	ankyrin repeat domain 20 family, member A2	-3.1776767	down
DPYSL3	NM_001387	dihydropyrimidinase-like 3	-7.8794856	down
			-2.1261191	down
LOC100131434	AK310119	uncharacterized LOC100131434	-2.4553752	down
	AK091705		-2.938854	down
SPIRE2	NM_032451	spire homolog 2 (Drosophila)	-3.050261	down
NPAS3	NM_022123	neuronal PAS domain protein 3	-2.5736332	down
RAP1GAP		RAP1 GTPase activating protein	-3.8229792	down
FOXD4L2	NM_001099279	forkhead box D4-like 2	-4.9400506	down
ROR1	NM_001083592	receptor tyrosine kinase-like orphan receptor 1	-3.349082	down
JPH2	NM_020433	junctophilin 2	-2.6826003	down
LOC100134368	NR_024453	uncharacterized LOC100134368	-3.5742295	down
SERP1	AK125413	stress-associated endoplasmic reticulum protein 1	-2.0096798	down
FAM159A	AK128236	family with sequence similarity 159, member A	-2.5474887	down
ZNF667	NM_022103	zinc finger protein 667	-3.8127968	down
RIMS1	NM_014989	regulating synaptic membrane exocytosis 1	-2.0362244	down
			-2.629157	down
			-3.5300465	down
ASCL1	NM_004316	achaete-scute complex homolog 1 (Drosophila)	-2.3816438	down
KIF26B	NM_018012	kinesin family member 26B	-2.444543	down
KIAA1984	NM_001039374	KIAA1984	-4.78533	down

UROC1	NM_144639	urocanase domain containing 1	-2.5256565	down
DUOX2	NM_014080	dual oxidase 2	-2.9561653	down
CDK13	NM_031267	cyclin-dependent kinase 13	-2.6361322	down
KY	NM_178554	kyphoscoliosis peptidase	-2.494125	down
			-2.5735772	down
MMD2	NM_198403	monocyte to macrophage differentiation-associated 2	-2.3638217	down
CXorf49B	NM_001145139	chromosome X open reading frame 49B	-2.1022503	down
	AK074886		-3.186019	down
HAND1	NM_004821	heart and neural crest derivatives expressed 1	-2.063052	down
RNASE7	NM_032572	ribonuclease, RNase A family, 7	-3.0744746	down
FAM83G	NM_001039999	family with sequence similarity 83, member G	-3.3051016	down
NR4A3	NM_173200	nuclear receptor subfamily 4, group A, member 3	-2.4256346	down
SPACA4	NM_133498	sperm acrosome associated 4	-2.8658245	down
	AK131247		-3.077039	down
WDFY4	NM_020945	WDFY family member 4	-6.073584	down
	AK124259		-2.2198312	down
NRXN1	NM_004801	neurexin 1	-6.327814	down
PGAM2	NM_000290	phosphoglycerate mutase 2 (muscle)	-2.2873666	down
MLANA	NM_005511	melan-A	-2.224407	down
	XR_110084		-3.0428755	down
MAS1L	NM_052967	MAS1 oncogene-like	-2.6141255	down
			-2.8040004	down
			-2.8989537	down
C1orf94	NM_032884	chromosome 1 open reading frame 94	-3.756665	down
EVC	NM_153717	Ellis van Creveld syndrome	-2.7697384	down
PLA2G2F	NM_022819	phospholipase A2, group IIF	-3.403952	down
TGM6	NM_198994	transglutaminase 6	-2.2753196	down
MAST4	NM_198828	microtubule associated serine/threonine kinase family member 4	-3.4127192	down
MGC10814	BC004943	uncharacterized protein MGC10814	-2.439593	down
CHKA	NM_001277	choline kinase alpha	-3.3115492	down
			-3.0070133	down
LOC729770	XR_112442	uncharacterized LOC729770	-6.183721	down
			-2.9896858	down
	AK130290		-3.0822678	down
PREPL	NM_006036	prolyl endopeptidase-like	-2.0861392	down
FLJ20712	XR_108766	uncharacterized FLJ20712	-4.678123	down
GDNF	NM_000514	glial cell derived neurotrophic factor	-2.1684034	down
DYNC1I1	NM_004411	dynein, cytoplasmic 1, intermediate chain 1	-3.1733766	down
			-2.6779542	down
POLQ	NM_199420	polymerase (DNA directed), theta	-3.7286987	down
ZDHHC14	NM_153746	zinc finger, DHHC-type containing 14	-2.8085394	down

CYP2C9	NM_000771	cytochrome P450, family 2, subfamily C, polypeptide 9	-2.0049195	down
HSD3B1	NM_000862	hydroxy-delta-5-steroid dehydrogenase, 3 beta- and steroid delta-isomerase 1	-2.3151848	down
TSPAN15	NM_012339	tetraspanin 15	-3.7497544	down
GOLGA6L5	NR_003246	golgin A6 family-like 5 (pseudogene)	-3.574765	down
LOC254099	NR_038869	uncharacterized LOC254099	-2.1128137	down
			-3.0458848	down
LOC100132147	BC036435	uncharacterized LOC100132147	-2.5759373	down
PTK2B	AK128371	PTK2B protein tyrosine kinase 2 beta	-5.003921	down
			-2.0228636	down
TMEM213	NM_001085429	transmembrane protein 213	-2.7899535	down
ATP6V0A4	NM_020632	ATPase, H+ transporting, lysosomal V0 subunit a4	-2.221564	down
CENPT	NM_025082	centromere protein T	-3.2732708	down
ZNF236	AF085243	zinc finger protein 236	-3.1913104	down
	XR_109245		-2.5847692	down
PAQR3	BC031256	progestin and adipoQ receptor family member III	-4.154528	down
FBXO48	NM_001024680	F-box protein 48	-2.45437	down
AP3B2	NM_004644	adaptor-related protein complex 3, beta 2 subunit	-3.4030423	down
PLA2G4F	NM_213600	phospholipase A2, group IVF	-2.6701288	down
PAX3	NM_013942	paired box 3	-3.6595964	down
LOC100133306	AK125136	uncharacterized LOC100133306	-2.4206388	down
BARHL1	NM_020064	BarH-like homeobox 1	-2.5366955	down
LRRTM2	NM_015564	leucine rich repeat transmembrane neuronal 2	-2.1928859	down
GLOD5	NM_001080489	glyoxalase domain containing 5	-3.307993	down
CHAC1	NM_024111	ChaC, cation transport regulator homolog 1 (E. coli)	-3.0818245	down
ENTPD3	NM_001248	ectonucleoside triphosphate diphosphohydrolase 3	-3.190444	down
HOXD10	NM_002148	homeobox D10	-2.7860587	down
IGSF1	NM_205833	immunoglobulin superfamily, member 1	-2.2437158	down
CCDC147		coiled-coil domain containing 147	-2.0214283	down
GPR151	NM_194251	G protein-coupled receptor 151	-2.0616794	down
KRTAP7-1	NM_181606	keratin associated protein 7-1 (gene/pseudogene)	-2.0595503	down
FHL2	CR936682	four and a half LIM domains 2	-3.5324337	down
ZSWIM5	NM_020883	zinc finger, SWIM-type containing 5	-2.3930855	down
PEG3-AS1	NR_023847	PEG3 antisense RNA 1 (non-protein coding)	-2.824169	down
C7orf60	NM_152556	chromosome 7 open reading frame 60	-2.3955445	down
			-2.2365222	down
KRTAP9-4	NM_033191	keratin associated protein 9-4	-2.282601	down
MS4A2	NM_000139	membrane-spanning 4-domains, subfamily A, member 2 (Fc fragment of IgE, high affinity I, receptor for; beta polypeptide)	-2.3528442	down
LINC00482	NR_038080	long intergenic non-protein coding RNA 482	-2.0731435	down
OTOG	AK128214	otogelin	-2.0336635	down
MOGAT1	NM_058165	monoacylglycerol O-acyltransferase 1	-2.0775344	down

DCDC5	NM_020869	doublecortin domain containing 5	-2.1005309	down
SGSM1	NM_001039948	small G protein signaling modulator 1	-4.0712523	down
	BI224516		-4.1411424	down
H1FOO	NM_153833	H1 histone family, member O, oocyte-specific	-2.195511	down
HHIPL1	NM_001127258	HHIP-like 1	-2.9411142	down
TNK2	NM_001010938	tyrosine kinase, non-receptor, 2	-3.161386	down
PRSS27	NM_031948	protease, serine 27	-2.892147	down
ADAD2	NM_139174	adenosine deaminase domain containing 2	-2.6538084	down
EGFLAM	NM_152403	EGF-like, fibronectin type III and laminin G domains	-2.8712275	down
IL21	NM_021803	interleukin 21	-2.505085	down
			-3.3438714	down
C12orf77	NM_001101339	chromosome 12 open reading frame 77	-2.3369946	down
PRPF40B	NM_001031698	PRP40 pre-mRNA processing factor 40 homolog B (S. cerevisiae)	-2.1412349	down
OR2AG1	NM_001004489	olfactory receptor, family 2, subfamily AG, member 1	-2.0516229	down
KRTAP13-3	NM_181622	keratin associated protein 13-3	-2.4094756	down
SHANK2	NM_012309	SH3 and multiple ankyrin repeat domains 2	-3.990878	down
LOC100129840	AK126633	uncharacterized LOC100129840	-2.2003484	down
SAMM50	AK124895	sorting and assembly machinery component 50 homolog (S. cerevisiae)	-2.794607	down
CLIC3	NM_004669	chloride intracellular channel 3	-2.174027	down
SLC10A7	NM_001029998	solute carrier family 10 (sodium/bile acid cotransporter family), member 7	-2.0299864	down
			-4.5553555	down
GRIK1-AS1	NR_027021	GRIK1 antisense RNA 1 (non-protein coding)	-4.8278446	down
IGF1	NM_000618	insulin-like growth factor 1 (somatomedin C)	-2.6714737	down
SMPDL3B	NM_001009568	sphingomyelin phosphodiesterase, acid-like 3B	-2.5115447	down
DEFB115	NM_001037730	defensin, beta 115	-2.3854241	down
LOC731789	NR_026794	uncharacterized LOC731789	-2.6800928	down
IDI2	NM_033261	isopentenyl-diphosphate delta isomerase 2	-2.9759207	down
MGC16121	NR_024607	uncharacterized protein MGC16121	-2.8313148	down
FAM99A	AK124823	family with sequence similarity 99, member A (non-protein coding)	-2.148725	down
			-3.501977	down
TAS2R13	NM_023920	taste receptor, type 2, member 13	-6.0611205	down
CHRDL2	NM_015424	chordin-like 2	-2.5211065	down
			-2.2791998	down
C1QTNF1	NM_198594	C1q and tumor necrosis factor related protein 1	-3.01527	down
RNF212	NM_001131034	ring finger protein 212	-2.3526158	down
KIAA0889	NM_199181	KIAA0889	-3.131229	down
YIF1B	NM_033557	Yip1 interacting factor homolog B (S. cerevisiae)	-2.184969	down
FAM134A	NM_024293	family with sequence similarity 134, member A	-2.2635784	down
			-3.0650012	down
ACTN2	NM_001103	actinin, alpha 2	-2.0038512	down

TMPRSS13	NM_001077263	transmembrane protease, serine 13	-2.1636193	down
SOX21	NM_007084	SRY (sex determining region Y)-box 21	-3.1146472	down
OR4F29	NM_001005221	olfactory receptor, family 4, subfamily F, member 29	-2.3857095	down
TREH	NM_007180	trehalase (brush-border membrane glycoprotein)	-3.3082974	down
LOC440040	NR_027044	glutamate receptor, metabotropic 5 pseudogene	-2.1251934	down
SERPINB4	NM_002974	serpin peptidase inhibitor, clade B (ovalbumin), member 4	-2.2896538	down
DMBX1	NM_147192	diencephalon/mesencephalon homeobox 1	-2.8457117	down
	AK054875		-3.8827298	down
			-2.4108953	down
CASR	NM_001178065	calcium-sensing receptor	-2.2855563	down
EGFR	NM_201282	epidermal growth factor receptor	-3.1577954	down
LOC653712	NR_034179	intraflagellar transport 122 homolog (Chlamydomonas) pseudogene	-4.171725	down
GRHL1	NM_198182	grainyhead-like 1 (Drosophila)	-2.2964911	down
AGBL1	NM_152336	ATP/GTP binding protein-like 1	-2.045469	down
	BC031238		-2.8013902	down
DEFB123	NM_153324	defensin, beta 123	-2.784746	down
FN1	NM_054034	fibronectin 1	-2.2919369	down
RBM39	NM_001242600	RNA binding motif protein 39	-2.0929222	down
EPHA10	NM_173641	EPH receptor A10	-2.3768528	down
CTSL3	NR_027917	cathepsin L family member 3	-3.0742848	down
ANKRD36BP1	NR_026844	ankyrin repeat domain 36B pseudogene 1	-2.0090399	down
			-3.0410738	down
FRG2B	NM_001080998	FSHD region gene 2 family, member B	-3.0783799	down
	AK128036		-2.6380951	down
PYY2	NR_003064	peptide YY, 2 (seminalplasmin)	-2.9844809	down
			-3.6973255	down
BACE1	NM_012104	beta-site APP-cleaving enzyme 1	-4.215377	down
KCNIP2	NM_173197	Kv channel interacting protein 2	-2.0455768	down
TBC1D24	NM_020705	TBC1 domain family, member 24	-2.0904098	down
PCA3	NR_015342	prostate cancer antigen 3 (non-protein coding)	-3.2699623	down
PLA2G4D	NM_178034	phospholipase A2, group IVD (cytosolic)	-2.7543018	down
LOC729626	XM_001133811	uncharacterized LOC729626	-4.814678	down
GJC3	NM_181538	gap junction protein, gamma 3, 30.2kDa	-7.2663884	down
			-2.6411629	down
WFDC5	NM_145652	WAP four-disulfide core domain 5	-5.2364945	down
LRRC14B	NM_001080478	leucine rich repeat containing 14B	-4.1470437	down
GUCA2A	NM_033553	guanylate cyclase activator 2A (guanylin)	-2.3264434	down
LOC388849	NM_001243537	uncharacterized LOC388849	-2.9330778	down
			-3.5745382	down
BAD	AK309150	BCL2-associated agonist of cell death	-2.5891025	down
			-3.2103443	down

SPRR2C	NR_003062	small proline-rich protein 2C (pseudogene)	-5.268663	down
COPS7B	NM_022730	COP9 constitutive photomorphogenic homolog subunit 7B (Arabidopsis)	-3.5340283	down
CHAT	NM_020549	choline O-acetyltransferase	-2.5076835	down
CYP2D6	NM_000106	cytochrome P450, family 2, subfamily D, polypeptide 6	-2.136789	down
	BC029043		-2.434501	down
LOC100132363	AK096512	uncharacterized LOC100132363	-2.4933207	down
PTH2	NM_178449	parathyroid hormone 2	-2.7637966	down
	DQ786249		-4.879596	down
TUBG1	NM_001070	tubulin, gamma 1	-2.647618	down
LOC100128320	AK125438	uncharacterized LOC100128320	-5.204045	down
MCCD1	NM_001011700	mitochondrial coiled-coil domain 1	-2.6316607	down
SLC22A12	NM_144585	solute carrier family 22 (organic anion/urate transporter), member 12	-2.382098	down
GPRC6A	NM_148963	G protein-coupled receptor, family C, group 6, member A	-2.0084155	down
	CN283607		-2.0692704	down
LOC257152	AK001439	uncharacterized LOC257152	-4.5231023	down
LOC644083	XR_112044	uncharacterized LOC644083	-2.031323	down
KIF18B	NM_001080443	kinesin family member 18B	-2.4329178	down
	AK127601		-7.013296	down
FAM182B	NR_027061	family with sequence similarity 182, member B	-4.446895	down
			-2.805535	down
LOC645427	AK094407	uncharacterized LOC645427	-2.940977	down
LOC646034	AK125175	uncharacterized LOC646034	-2.8623116	down
SLC22A6	NM_153277	solute carrier family 22 (organic anion transporter), member 6	-3.6926575	down
OR2L3	NM_001004687	olfactory receptor, family 2, subfamily L, member 3	-3.0904346	down
LOC284379	NR_002938	solute carrier family 7 (cationic amino acid transporter, y+ system), member 3 pseudogene	-2.5896008	down
LOC728093	XR_133401	putative POM121-like protein 1-like	-3.1270144	down
LOC339240	NR_001443	keratin 17 pseudogene	-2.251825	down
DNM1	AB209124	dynamin 1	-5.9209957	down
OR1K1	NM_080859	olfactory receptor, family 1, subfamily K, member 1	-2.29827	down
TMEM239	NM_001167670	transmembrane protein 239	-2.354593	down
LOC646743	NR_033930	uncharacterized LOC646743	-6.9335494	down
TNNT2	NM_000364	troponin T type 2 (cardiac)	-3.6965022	down
KLK15	NM_017509	kallikrein-related peptidase 15	-2.576642	down
C9orf152	NM_001012993	chromosome 9 open reading frame 152	-2.067756	down
SCGB1D2	NM_006551	secretoglobin, family 1D, member 2	-2.367468	down
PRKG2	NM_006259	protein kinase, cGMP-dependent, type II	-2.108568	down
	XR_110942		-6.3074183	down
LOC100130175	AK125829	uncharacterized LOC100130175	-2.659356	down
			-4.5740514	down
			-4.9121447	down

C9orf147	XR_133532	chromosome 9 open reading frame 147	-2.159597	down
			-2.1852236	down
SRCRB4D	NM_080744	scavenger receptor cysteine rich domain containing, group B (4 domains)	-2.0075288	down
FLJ30403	NR_034159	uncharacterized LOC729975	-5.3173976	down
SNX21	NM_152897	sorting nexin family member 21	-2.4536612	down
PRR20B	NM_001130404	proline rich 20B	-2.647472	down
	AW002507		-3.341208	down
			-2.7077096	down
			-2.3534725	down
MUC13	NM_033049	mucin 13, cell surface associated	-6.926307	down
TTTY14	NR_001543	testis-specific transcript, Y-linked 14 (non-protein coding)	-2.7651021	down
NT5E	NM_002526	5'-nucleotidase, ecto (CD73)	-2.1316285	down
C2orf66	NM_213608	chromosome 2 open reading frame 66	-2.7817643	down
RFPL4A	NM_001145014	ret finger protein-like 4A	-2.625456	down
	AK125780		-2.2565725	down
CELA3A	AK308514	chymotrypsin-like elastase family, member 3A	-5.3999424	down
			-3.0245314	down
DKK3	NM_015881	dickkopf 3 homolog (Xenopus laevis)	-5.5479383	down
C14orf162	NR_024630	chromosome 14 open reading frame 162	-2.351535	down
MYL3	NM_000258	myosin, light chain 3, alkali; ventricular, skeletal, slow	-3.9522247	down
			-3.6487744	down
SLC8A2	NM_015063	solute carrier family 8 (sodium/calcium exchanger), member 2	-3.599214	down
			-2.4550724	down
			-2.405829	down
			-2.266612	down
ASTL	NM_001002036	astacin-like metallo-endopeptidase (M12 family)	-5.6348124	down
FAM47A	NM_203408	family with sequence similarity 47, member A	-2.873728	down
OR2T1	NM_030904	olfactory receptor, family 2, subfamily T, member 1	-2.457143	down
OR5E1P	AF309699	olfactory receptor, family 5, subfamily E, member 1 pseudogene	-2.6311245	down
OR52R1	NM_001005177	olfactory receptor, family 52, subfamily R, member 1	-2.3003714	down
COL23A1	CU692082	collagen, type XXIII, alpha 1	-2.252874	down
KCNJ13	NM_002242	potassium inwardly-rectifying channel, subfamily J, member 13	-3.466033	down
LOC440131	NR_033889	uncharacterized LOC440131	-3.2540586	down
	BM457408		-2.5434105	down
HSPB1	NM_001540	heat shock 27kDa protein 1	-2.020122	down
	AK123300		-2.0487065	down
SLIT3	NM_003062	slit homolog 3 (Drosophila)	-2.226543	down
LAMA4	NM_001105206	laminin, alpha 4	-5.1228747	down
BCL2L15	NM_001010922	BCL2-like 15	-3.1335475	down
SIGLECP3	NR_002804	sialic acid binding Ig-like lectin, pseudogene 3	-5.5102215	down
			-2.6297312	down

USP21	NM_001014443	ubiquitin specific peptidase 21	-2.4280882	down
OR1C1	NM_012353	olfactory receptor, family 1, subfamily C, member 1	-2.6557124	down
SLC4A2	NM_003040	solute carrier family 4, anion exchanger, member 2 (erythrocyte membrane protein band 3-like 1)	-2.6451623	down
DOC2B	NM_003585	double C2-like domains, beta	-2.101427	down
VKORC1	AK125618	vitamin K epoxide reductase complex, subunit 1	-3.1096578	down
			-5.4172783	down
LOC100652824	XM_003403420	uncharacterized LOC100652824	-2.028815	down
ACSM5	NM_017888	acyl-CoA synthetase medium-chain family member 5	-3.1996808	down
LOC100131434	NR_027455	uncharacterized LOC100131434	-3.4121733	down
			-4.2110543	down
LOC391767	XM_001715028	putative TAF11-like protein ENSP00000332601-like	-3.2585142	down
			-2.1952505	down
CHST14	NM_130468	carbohydrate (N-acetylgalactosamine 4-0) sulfotransferase 14	-2.205755	down
SLC9A5	NM_004594	solute carrier family 9 (sodium/hydrogen exchanger), member 5	-2.5307686	down
			-3.3831615	down
SPANXA1	NM_013453	sperm protein associated with the nucleus, X-linked, family member A1	-3.1174965	down
MYH15	NM_014981	myosin, heavy chain 15	-2.0230348	down
NTN5	NM_145807	netrin 5	-3.8881257	down
SGPP2	NM_152386	sphingosine-1-phosphate phosphatase 2	-2.0176077	down
ATP4B	NM_000705	ATPase, H+/K+ exchanging, beta polypeptide	-3.2286546	down
			-5.982001	down
C9orf96	NM_153710	chromosome 9 open reading frame 96	-2.5803359	down
FHAD1	NM_052929	forkhead-associated (FHA) phosphopeptide binding domain 1	-2.1333544	down
ATM	BC007023	ataxia telangiectasia mutated	-2.143438	down
ZNRD1-AS1	NR_026751	ZNRD1 antisense RNA 1 (non-protein coding)	-5.032544	down
			-3.5694723	down
BMP10	NM_014482	bone morphogenetic protein 10	-2.414793	down
PPAN-P2RY11	NM_001040664	PPAN-P2RY11 readthrough	-4.0220838	down
SPRY4	NM_030964	sprouty homolog 4 (Drosophila)	-2.0099323	down
HAPLN1	NM_001884	hyaluronan and proteoglycan link protein 1	-2.2465894	down
C9orf153	NM_001010907	chromosome 9 open reading frame 153	-2.542598	down
LYPD6	NM_001195685	LY6/PLAUR domain containing 6	-3.7093213	down
			-2.8566647	down
CST1	NM_001898	cystatin SN	-2.5339808	down
SYNGAP1	NM_006772	synaptic Ras GTPase activating protein 1	-2.971704	down
LOC647983	AK126828	uncharacterized LOC647983	-4.001189	down
			-2.1137738	down
SMPX	NM_014332	small muscle protein, X-linked	-2.355111	down
TMC8	NM_152468	transmembrane channel-like 8	-2.5862494	down
PPIL4	NM_139126	peptidylprolyl isomerase (cyclophilin)-like 4	-2.300738	down

NIPAL2	NM_024759	NIPA-like domain containing 2	-2.0928764	down
TMEM191B	NM_001242313	transmembrane protein 191B	-2.428717	down
TRIM31	NM_007028	tripartite motif containing 31	-2.069862	down
	BC066548		-2.1639297	down
GCM2	NM_004752	glial cells missing homolog 2 (Drosophila)	-4.5928836	down
PTAR1	AK127511	protein prenyltransferase alpha subunit repeat containing 1	-2.4225144	down
SLC25A35	NM_201520	solute carrier family 25, member 35	-2.069909	down
SPDYE3	NM_001004351	speedy homolog E3 (Xenopus laevis)	-5.2403007	down
EMILIN1	NM_007046	elastin microfibril interfacer 1	-2.524348	down
C17orf47	NM_001038704	chromosome 17 open reading frame 47	-4.134124	down
C20orf112	NM_080616	chromosome 20 open reading frame 112	-3.826094	down
OLIG3	NM_175747	oligodendrocyte transcription factor 3	-3.3683784	down
CELF5	NM_021938	CUGBP, Elav-like family member 5	-2.1728947	down
LOC100131831	AK129685	uncharacterized LOC100131831	-2.1375268	down
FLJ25694	AK127969	uncharacterized protein FLJ25694	-7.518144	down
SAMD5	NM_001030060	sterile alpha motif domain containing 5	-2.6627824	down
OR2M5	NM_001004690	olfactory receptor, family 2, subfamily M, member 5	-5.429307	down
TMEM52	NM_178545	transmembrane protein 52	-2.336586	down
	AK125883		-2.1502578	down
FGF18	NM_003862	fibroblast growth factor 18	-2.57209	down
HOXB5	NM_002147	homeobox B5	-3.2419593	down
LOC731656	NR_027454	uncharacterized LOC731656	-2.717006	down
FAM59B	NM_001191033	family with sequence similarity 59, member B	-2.231845	down
MS4A15	NM_001098835	membrane-spanning 4-domains, subfamily A, member 15	-2.134461	down
FLT4	NM_002020	fms-related tyrosine kinase 4	-2.6600595	down
BAI2	NM_001703	brain-specific angiogenesis inhibitor 2	-2.055258	down
	AF416714		-2.80043	down
CNTN2	NM_005076	contactin 2 (axonal)	-4.524108	down
SMAD5-AS1	NR_026763	SMAD5 antisense RNA 1 (non-protein coding)	-2.0423934	down
GPR133	NM_198827	G protein-coupled receptor 133	-3.0372365	down
SLC7A14	NM_020949	solute carrier family 7 (orphan transporter), member 14	-2.4758465	down
	CU687439		-2.1815228	down
COL4A1	NM_001845	collagen, type IV, alpha 1	-4.864076	down
			-2.1866405	down
ZC3H10	NM_032786	zinc finger CCCH-type containing 10	-2.6616583	down
TGFB2	NM_003238	transforming growth factor, beta 2	-2.0163758	down
LOC644662	XM_933903	uncharacterized LOC644662	-2.0723808	down
C3orf20	NM_032137	chromosome 3 open reading frame 20	-3.0207899	down
SVOPL	NM_174959	SVOP-like	-2.2200868	down
PSMB7		proteasome (prosome, macropain) subunit, beta type, 7	-2.0117671	down
TMEM194B		transmembrane protein 194B	-2.1395192	down

NEAT1	AF001893	nuclear paraspeckle assembly transcript 1 (non-protein coding)	-2.8376615	down
LOC441956	XR_109656	uncharacterized LOC441956	-3.221395	down
AHNAK2	NM_138420	AHNAK nucleoprotein 2	-3.2539978	down
SELV	NM_182704	selenoprotein V	-3.2637463	down
GYG2	NM_001184703	glycogenin 2	-2.4951386	down
C11orf95	NM_001144936	chromosome 11 open reading frame 95	-2.8758984	down
SSX4B	NM_001034832	synovial sarcoma, X breakpoint 4B	-5.78702	down
TDRD3	BC020604	tudor domain containing 3	-3.1269019	down
TOX2	NM_032883	TOX high mobility group box family member 2	-2.1438344	down
LOC100130865	AK123130	uncharacterized LOC100130865	-3.081819	down
	AK093166		-2.3832526	down
MGC20647	BC008289	uncharacterized protein MGC20647	-3.140553	down
			-2.009179	down
			-3.9271653	down
LENG8	NM_052925	leukocyte receptor cluster (LRC) member 8	-2.405218	down
MIXL1	NM_031944	Mix paired-like homeobox	-2.4086452	down
CES3	NM_024922	carboxylesterase 3	-3.7508008	down
			-5.4234653	down
CXorf38	AL832829	chromosome X open reading frame 38	-2.774094	down
TSPAN8	NM_004616	tetraspanin 8	-2.351582	down
LOC100289650	NR_036584	uncharacterized LOC100289650	-5.134286	down
HULC	NR_004855	highly up-regulated in liver cancer (non-protein coding)	-2.8277068	down
CDKAL1	NM_017774	CDK5 regulatory subunit associated protein 1-like 1	-2.3557155	down
CP	NM_000096	ceruloplasmin (ferroxidase)	-2.0804539	down
TTC28-AS1	NR_026963	TTC28 antisense RNA 1 (non-protein coding)	-5.1564555	down
			-3.246623	down
IGFL1	NM_198541	IGF-like family member 1	-2.6515749	down
HES5	NM_001010926	hairy and enhancer of split 5 (Drosophila)	-4.611401	down
MYO5B	NM_001080467	myosin VB	-2.735534	down
LINC00302	AF005082	long intergenic non-protein coding RNA 302	-4.5198603	down
GORAB	NM_001146039	golgin, RAB6-interacting	-2.1386724	down
RP11-165H20.1	NR_003928	CHIA-like pseudogene	-2.6871252	down
LOC100129223	XR_111243	uncharacterized LOC100129223	-3.0323548	down
	BC064144		-2.941225	down
SPRYD5	NM_032681	SPRY domain containing 5	-3.7017303	down
FAM160B2	NM_022749	family with sequence similarity 160, member B2	-2.9236286	down
PRSS54	NM_001080492	protease, serine, 54	-2.1586802	down
PBLD	NM_001033083	phenazine biosynthesis-like protein domain containing	-2.2117817	down
			-3.143149	down
TMEM44	NM_001011655	transmembrane protein 44	-2.4158256	down
			-2.0613482	down

			-2.052461	down
LOC730139	BG168244	uncharacterized LOC730139	-8.081863	down
SGK494	NM_001174103	uncharacterized serine/threonine-protein kinase SgK494	-4.712771	down
UGT1A6	NM_001072	UDP glucuronosyltransferase 1 family, polypeptide A6	-2.7758641	down
LOC100130453	AK123920	uncharacterized LOC100130453	-3.266371	down
RUFY4	NM_198483	RUN and FYVE domain containing 4	-4.517506	down
ARHGAP35	NM_004491	Rho GTPase activating protein 35	-2.017066	down
DEFT1P	NR_036686	defensin, theta 1 pseudogene	-2.2326682	down
TGIF1		TGFB-induced factor homeobox 1	-6.2166343	down
CCDC144A	NM_014695	coiled-coil domain containing 144A	-2.1359017	down
FLJ37786	XR_108343	uncharacterized LOC642691	-2.85832	down
FTCD	NM_206965	formiminotransferase cyclodeaminase	-2.718331	down
ADAT2	NM_182503	adenosine deaminase, tRNA-specific 2	-2.2314584	down
TMEM25	NM_032780	transmembrane protein 25	-2.5767536	down
INPP5F	NM_001243195	inositol polyphosphate-5-phosphatase F	-2.517566	down
			-4.5056696	down
ADAMTSL2	NM_014694	ADAMTS-like 2	-2.3844516	down
MPDU1	AK027742	mannose-P-dolichol utilization defect 1	-2.5096831	down
			-2.5572865	down
GSTM5	NM_000851	glutathione S-transferase mu 5	-2.880986	down
ENDOV	NM_001164638	endonuclease V	-2.1511707	down
LOC440742	XR_110857	uncharacterized LOC440742	-4.881606	down
CCDC11	NM_145020	coiled-coil domain containing 11	-2.6664994	down
SPACA5	NM_205856	sperm acrosome associated 5	-3.5503838	down
PTPRG	NM_002841	protein tyrosine phosphatase, receptor type, G	-2.7297883	down
LOC100131894	AK092825	uncharacterized LOC100131894	-3.298152	down
HTR4	NM_001040173	5-hydroxytryptamine (serotonin) receptor 4	-2.7669418	down
			-2.4836795	down
ZNF763	NM_001012753	zinc finger protein 763	-2.4495506	down
			-2.4366586	down
LINC00222	NR_033376	long intergenic non-protein coding RNA 222	-2.7653391	down
HIBCH	NM_198047	3-hydroxyisobutyryl-CoA hydrolase	-2.5109205	down
LOC100130348	AK124141	uncharacterized LOC100130348	-2.9576893	down
KIAA1244	NM_020340	KIAA1244	-2.0615048	down
LINC00263	NR_026762	long intergenic non-protein coding RNA 263	-2.3385224	down
	CR622587		-2.3800116	down
			-3.0835307	down
	AK127957		-2.077758	down
	XR_110433		-3.4432583	down
ADAM21	NM_003813	ADAM metallopeptidase domain 21	-2.1352837	down
SHISA9	NM_001145205	shisa homolog 9 (Xenopus laevis)	-2.3256302	down

NR1D2	BC070035	nuclear receptor subfamily 1, group D, member 2	-2.9418225	down
	AK126625		-4.3313055	down
CHRNA5	NM_000745	cholinergic receptor, nicotinic, alpha 5	-3.8311088	down
GOLGA6A	NM_001038640	golgin A6 family, member A	-4.515959	down
KRTAP4-1	NM_033060	keratin associated protein 4-1	-7.800764	down
DEFB108B	NM_001002035	defensin, beta 108B	-3.193814	down
SLFN5	NM_144975	schlafen family member 5	-2.7624593	down
RAMP1	NM_005855	receptor (G protein-coupled) activity modifying protein 1	-2.639751	down
LOC727993	AK090681	uncharacterized LOC727993	-7.231693	down
SH3GL1P3	X99663	SH3-domain GRB2-like 1 pseudogene 3	-2.930762	down
NKAIN4	NM_152864	Na+/K+ transporting ATPase interacting 4	-2.099535	down
SLC3A1	DQ023513	solute carrier family 3 (cystine, dibasic and neutral amino acid transporters, activator of cystine, dibasic and neutral amino acid transport), member 1	-2.5089746	down
PRRC2B	NM_013318	proline-rich coiled-coil 2B	-3.4010828	down
LOC646719	XR_112726	uncharacterized LOC646719	-2.1726642	down
CCDC104	AK309764	coiled-coil domain containing 104	-4.960236	down
	BC039333		-2.258889	down
IFNW1	NM_002177	interferon, omega 1	-2.479682	down
DEDD	AF064605	death effector domain containing	-3.5773826	down
ZNF662	NM_207404	zinc finger protein 662	-3.2179415	down
C1orf227	NM_001024601	chromosome 1 open reading frame 227	-2.218295	down
LRRC49	NM_017691	leucine rich repeat containing 49	-2.1868796	down
MFSD9	NM_032718	major facilitator superfamily domain containing 9	-2.0727165	down
C21orf88	NR_026542	chromosome 21 open reading frame 88	-2.2156951	down
	AK055155		-2.4456036	down
RPRML	NM_203400	reprimo-like	-2.5461533	down
PLA2G4F	NM_213600	phospholipase A2, group IVF	-3.569584	down
TAS2R41	NM_176883	taste receptor, type 2, member 41	-2.5078769	down
TBX10	NM_005995	T-box 10	-2.1203413	down
CACNA2D1	NM_000722	calcium channel, voltage-dependent, alpha 2/delta subunit 1	-2.2805147	down
H2AFB2	NM_001017991	H2A histone family, member B2	-3.7219415	down
	AK127935		-2.2251754	down
LOC100128563	XR_132703	uncharacterized LOC100128563	-2.5026765	down
MC3R	NM_019888	melanocortin 3 receptor	-2.5158591	down
LOC440297	NR_033579	chondroitin sulfate proteoglycan 4 pseudogene	-2.8473406	down
PTTG1IP	NM_004339	pituitary tumor-transforming 1 interacting protein	-2.2359407	down
MAN1A2	NM_006699	mannosidase, alpha, class 1A, member 2	-2.2242367	down
OR2B6	NM_012367	olfactory receptor, family 2, subfamily B, member 6	-2.1525958	down
LBP	NM_004139	lipopolysaccharide binding protein	-2.1001062	down
CYP1A2	NM_000761	cytochrome P450, family 1, subfamily A, polypeptide 2	-2.5994556	down
LOC286272	AK093004	uncharacterized LOC286272	-2.0242498	down

	BC132839		-2.8446035	down
			-3.5111523	down
SPRED3	NM_001039616	sprouty-related, EVH1 domain containing 3	-3.9820526	down
	BC130558		-2.5987318	down
TARM1	NM_001135686	T cell-interacting, activating receptor on myeloid cells 1	-2.3951137	down
	BG216262		-2.2752378	down
RAI1	AJ271790	retinoic acid induced 1	-2.4362583	down
VCX2	NM_016378	variable charge, X-linked 2	-4.6333914	down
	BE244176		-3.5649886	down
FLG	NM_002016	filaggrin	-3.173141	down
			-2.520661	down
SLC17A6	NM_020346	solute carrier family 17 (sodium-dependent inorganic phosphate cotransporter), member 6	-6.0544634	down
LACRT	NM_033277	lacritin	-2.9059248	down
LOC100126784	NR_015384	uncharacterized LOC100126784	-3.4552524	down
TRIP13	NM_001166260	thyroid hormone receptor interactor 13	-2.2269318	down
LOC100127909	XR_111781	uncharacterized LOC100127909	-3.1288	down
DUSP8	NM_004420	dual specificity phosphatase 8	-2.5769916	down
LOC400548	NR_033984	uncharacterized LOC400548	-2.40036	down
			-4.2483263	down
CSNK1A1P1	NR_027320	casein kinase 1, alpha 1 pseudogene 1	-2.1933153	down
LOC440297	NR_033579	chondroitin sulfate proteoglycan 4 pseudogene	-2.009342	down
ZNRF4	NM_181710	zinc and ring finger 4	-3.69466	down
OR2L8	NM_001001963	olfactory receptor, family 2, subfamily L, member 8	-2.1067717	down
KRTAP1-5	NM_031957	keratin associated protein 1-5	-2.1074543	down
TMEM132A	NM_017870	transmembrane protein 132A	-2.3369367	down
LOC100131860	AK097109	uncharacterized LOC100131860	-2.086315	down
SPRR2F	NM_001014450	small proline-rich protein 2F	-2.3916743	down
SLC3A1	NM_000341	solute carrier family 3 (cystine, dibasic and neutral amino acid transporters, activator of cystine, dibasic and neutral amino acid transport), member 1	-9.613231	down
BEST3	AF440758	bestrophin 3	-3.3493996	down
TMEM35	NM_021637	transmembrane protein 35	-4.691395	down
OPN1MW	NM_000513	opsin 1 (cone pigments), medium-wave-sensitive	-3.7852883	down
KLK2	NM_001002231	kallikrein-related peptidase 2	-3.8603814	down
NCKAP5L	BC110599	NCK-associated protein 5-like	-3.3476906	down
CAV1	NM_001753	caveolin 1, caveolae protein, 22kDa	-2.6581335	down
LOC285095	XM_001722643	uncharacterized LOC285095	-2.771608	down
IQCH	NM_001031715	IQ motif containing H	-2.1537576	down
FMO2	NM_001460	flavin containing monooxygenase 2 (non-functional)	-2.3110301	down
			-2.2918015	down
CRYGN	NM_144727	crystallin, gamma N	-2.383322	down

HSD3B7	NM_001142777	hydroxy-delta-5-steroid dehydrogenase, 3 beta- and steroid delta-isomerase 7	-2.4414687	down
TBX2	NM_005994	T-box 2	-2.4387703	down
ALDOAP2	M21191	aldolase A, fructose-bisphosphate pseudogene 2	-2.0807843	down
			-2.3149915	down
NRAP	NM_198060	nebulin-related anchoring protein	-2.4597619	down
C6orf132	NM_001164446	chromosome 6 open reading frame 132	-9.727346	down
DNAH12	NM_178504	dynein, axonemal, heavy chain 12	-2.3318284	down
ABCG8	NM_022437	ATP-binding cassette, sub-family G (WHITE), member 8	-4.009081	down
			-3.3536286	down
ANKRD20A9P	NR_027995	ankyrin repeat domain 20 family, member A9, pseudogene	-2.7862835	down
MRO	NM_031939	maestro	-2.1969876	down
RHBDD1		rhomboid domain containing 1	-4.3730392	down
	BX104084		-10.683768	down
FAM25A	NM_001146157	family with sequence similarity 25, member A	-5.3189464	down
DPYSL5	NM_020134	dihydropyrimidinase-like 5	-3.9440286	down
LOC100509091	XM_003403877	uncharacterized LOC100509091	-2.1500516	down
SPACA5	NM_205856	sperm acrosome associated 5	-2.5995908	down
LOC644285	AK126853	uncharacterized LOC644285	-2.3458006	down
LDHAL6A	NM_144972	lactate dehydrogenase A-like 6A	-2.3466198	down
LOC100128857	XR_132709	beta-1,3-N-acetylgalactosaminyltransferase 1 (globoside blood group) pseudogene	-2.3515322	down
LOC100131000	AK124361	uncharacterized LOC100131000	-3.218077	down
			-2.2598424	down
DHH	NM_021044	desert hedgehog	-2.851316	down
FLJ12825	NR_026655	uncharacterized LOC440101	-2.1450946	down
ZAR1	NM_175619	zygote arrest 1	-4.3487625	down
RPL28	NM_001136137	ribosomal protein L28	-2.2861476	down
			-2.0454698	down
KRT6B	NM_005555	keratin 6B	-2.2352533	down
PRSS55	NM_198464	protease, serine, 55	-3.3137004	down
SEC14L6	NM_001193336	SEC14-like 6 (S. cerevisiae)	-3.011952	down
LOC728061	AK025151	hCG2003663	-2.5644073	down
BTN2A3P	NR_027795	butyrophilin, subfamily 2, member A3, pseudogene	-2.238426	down
FAM198A	AK091001	family with sequence similarity 198, member A	-3.992717	down
LOC100130849	NR_038450	phosphorylase kinase, gamma 1 (muscle) pseudogene	-3.8647861	down
			-4.554587	down
	AK096324		-2.1652827	down
OR14A16	NM_001001966	olfactory receptor, family 14, subfamily A, member 16	-2.6017656	down
			-2.4328039	down
FLJ46906	NR_033896	uncharacterized LOC441172	-2.533102	down
LOC100128496	CR738909	uncharacterized LOC100128496	-2.1220982	down

	DB066855		-5.1644583	down
INMT	NM_001199219	indolethylamine N-methyltransferase	-2.257948	down
SLC7A8	NM_182728	solute carrier family 7 (amino acid transporter light chain, L system), member 8	-2.5715306	down
PKDCC	AK311155	protein kinase domain containing, cytoplasmic homolog (mouse)	-2.9054477	down
TAS2R19	NM_176888	taste receptor, type 2, member 19	-2.273835	down
			-3.831292	down
ZNF81	NM_007137	zinc finger protein 81	-3.3331683	down
			-2.6774642	down
ZNF511	AK091711	zinc finger protein 511	-2.297694	down
	CR748640		-2.3447924	down
			-5.732493	down
	AK126080		-4.7672544	down
			-2.010095	down
RAB43P1	BM917410	RAB43 pseudogene 1	-4.0891805	down
PPP1R14D	NM_017726	protein phosphatase 1, regulatory (inhibitor) subunit 14D	-2.2565925	down
			-9.106207	down
ABI3BP	NM_015429	ABI family, member 3 (NESH) binding protein	-2.7335498	down
	AK127417		-2.0540762	down
CMIP	NM_198390	c-Maf inducing protein	-2.3066244	down
			-2.3209527	down
	AL137562		-3.1207476	down
LOC100130857	AK097050	uncharacterized LOC100130857	-2.1709507	down
FLJ11710	AK021772	uncharacterized protein FLJ11710	-3.912566	down
LOC100129098	XM_001714893	uncharacterized LOC100129098	-2.7728262	down
CPXM2	NM_198148	carboxypeptidase X (M14 family), member 2	-3.7511039	down
CCDC103	NM_213607	coiled-coil domain containing 103	-2.7786374	down
INO80	NM_017553	INO80 homolog (S. cerevisiae)	-2.2209868	down
AQP7P3	NR_026558	aquaporin 7 pseudogene 3	-2.6258645	down
PRRT2	NM_145239	proline-rich transmembrane protein 2	-2.5783308	down
DNAJC5	NM_025219	DnaJ (Hsp40) homolog, subfamily C, member 5	-2.2478328	down
LCNL1	NM_207510	lipocalin-like 1	-3.0202014	down
CFHR1	NM_002113	complement factor H-related 1	-2.6748908	down
PRAMEF7	NM_001012277	PRAME family member 7	-2.3781807	down
CNTNAP4	NM_138994	contactin associated protein-like 4	-2.8021007	down
	AK097770		-2.039524	down
OR5T3	NM_001004747	olfactory receptor, family 5, subfamily T, member 3	-2.5306466	down
HCN2	NM_001194	hyperpolarization activated cyclic nucleotide-gated potassium channel 2	-2.650218	down
RASA4	NM_001079877	RAS p21 protein activator 4	-2.1796172	down
			-2.3137233	down
MYCNOS	NR_026766	MYCN opposite strand/antisense RNA (non-protein coding)	-2.9692016	down

SLC11A1	NM_000578	solute carrier family 11 (proton-coupled divalent metal ion transporters), member 1	-2.4163134	down
ADAM19	NM_033274	ADAM metallopeptidase domain 19	-3.0050838	down
OR2H2	NM_007160	olfactory receptor, family 2, subfamily H, member 2	-3.0151265	down
SOD3	NM_003102	superoxide dismutase 3, extracellular	-2.8318462	down
AVP	NM_000490	arginine vasopressin	-3.2853966	down
MFSD6L	NM_152599	major facilitator superfamily domain containing 6-like	-3.7301908	down
LOC100131607	XR_108637	uncharacterized LOC100131607	-2.7394753	down
	AK090928		-2.9178474	down
OR6C74	NM_001005490	olfactory receptor, family 6, subfamily C, member 74	-2.3116274	down
UNQ9370	XR_111551	IFMQ9370	-6.3807073	down
AP4B1	NM_006594	adaptor-related protein complex 4, beta 1 subunit	-2.28458	down
			-2.7157795	down
SLC5A4	NM_014227	solute carrier family 5 (low affinity glucose cotransporter), member 4	-2.34644	down
LOC157860	AK025743	uncharacterized LOC157860	-2.5894132	down
CREB3L4	NM_130898	cAMP responsive element binding protein 3-like 4	-2.0981402	down
PTPRG	NM_002841	protein tyrosine phosphatase, receptor type, G	-2.2563598	down
PDE4DIP		phosphodiesterase 4D interacting protein	-3.227337	down
TEX19	NM_207459	testis expressed 19	-2.0959647	down
SOX13	BC040649	SRY (sex determining region Y)-box 13	-2.0764039	down
SPC24	NM_182513	SPC24, NDC80 kinetochore complex component, homolog (S. cerevisiae)	-2.0852225	down
LINC00114	AY204748	long intergenic non-protein coding RNA 114	-2.791678	down
LOC100130354	BC069735	uncharacterized LOC100130354	-3.9027586	down
PSG5	BG354573	pregnancy specific beta-1-glycoprotein 5	-3.4516673	down
FAM19A5		family with sequence similarity 19 (chemokine (C-C motif)-like), member A5	-2.7488794	down
C21orf58	NM_058180	chromosome 21 open reading frame 58	-2.3386693	down
FLJ10661	NR_024361	family with sequence similarity 86, member A pseudogene	-3.0564413	down
			-6.469771	down
FAM84A	NM_145175	family with sequence similarity 84, member A	-2.6488738	down
			-5.801462	down
TMCO4	NM_181719	transmembrane and coiled-coil domains 4	-2.375089	down
C6orf58	NM_001010905	chromosome 6 open reading frame 58	-2.2836833	down
LOC100129917	NR_036511	uncharacterized LOC100129917	-2.1845236	down
SMC1B	NM_148674	structural maintenance of chromosomes 1B	-2.4287293	down
OR13C4	NM_001001919	olfactory receptor, family 13, subfamily C, member 4	-2.8245487	down
IL20RA	AK098312	interleukin 20 receptor, alpha	-2.0692644	down
S100A5	NM_002962	S100 calcium binding protein A5	-3.9728982	down
	DB511925		-3.0654693	down
GGT5	NM_001099781	gamma-glutamyltransferase 5	-2.857171	down
GLIS3	DQ438879	GLIS family zinc finger 3	-3.84525	down
LOC100131581	AK092544	uncharacterized LOC100131581	-6.2694902	down

	AK097463		-2.0526426	down
NEK2	NM_002497	NIMA (never in mitosis gene a)-related kinase 2	-2.6656015	down
GPM6B	NM_001001996	glycoprotein M6B	-3.0386808	down
RP9P	NR_003500	retinitis pigmentosa 9 pseudogene	-4.1858306	down
	AF289562		-2.5417893	down
			-2.1793094	down
			-2.4414911	down
PTGER3	NM_198715	prostaglandin E receptor 3 (subtype EP3)	-2.3230703	down
	XR_108734		-3.5592332	down
			-4.4264274	down
CPNE9	NM_153635	copine family member IX	-2.8515382	down
DCLK1	NM_004734	doublecortin-like kinase 1	-4.0360823	down
WFIKKN2	NM_175575	WAP, follistatin/kazal, immunoglobulin, kunitz and netrin domain containing 2	-2.9805276	down
MUC3	AF007191	intestinal mucin-like	-2.2538464	down
DNM3	NM_001136127	dynamin 3	-2.1572034	down
	CU692315		-2.4623742	down
TBX1	NM_080647	T-box 1	-3.7338393	down
WNK4	NM_032387	WNK lysine deficient protein kinase 4	-2.4359827	down
	DA067175		-3.1556063	down
MFSD3	NM_138431	major facilitator superfamily domain containing 3	-2.2270577	down
FAM189A2	NM_004816	family with sequence similarity 189, member A2	-2.1782188	down
KRBOX1	NM_001205272	KRAB box domain containing 1	-2.0611854	down
08-sep	NM_001098811	septin 8	-2.7487297	down
			-3.4421222	down
LOC285740	NR_027114	uncharacterized LOC285740	-3.6962438	down
BMP3	NM_001201	bone morphogenetic protein 3	-4.1398873	down
KCNQ2	NM_172109	potassium voltage-gated channel, KQT-like subfamily, member 2	-2.9891136	down
	BC051343		-2.9513226	down
LOC100132197	AK125954	uncharacterized LOC100132197	-3.079703	down
FAM138E	NR_026819	family with sequence similarity 138, member E	-2.0997684	down
L3MBTL4	NM_173464	l(3)mbt-like 4 (Drosophila)	-2.481974	down
			-3.3554232	down
TBCB		tubulin folding cofactor B	-2.6268542	down
CCDC114	NM_144577	coiled-coil domain containing 114	-2.90382	down
MAGEC3	NM_138702	melanoma antigen family C, 3	-3.0491858	down
FRAS1	AK092082	Fraser syndrome 1	-2.0997248	down
FBXO27	NM_178820	F-box protein 27	-3.283798	down
GPR101	NM_054021	G protein-coupled receptor 101	-2.0174198	down
LOC100131581	AK092544	uncharacterized LOC100131581	-4.8780975	down
