# Supplementary material for: Transcriptional signature associated with early rheumatoid arthritis and healthy individuals at high risk to develop the disease
Source: PLoS One. 2018 Mar 27;13(3):e0194205. doi: 10.1371/journal.pone.0194205 (PMC5870959; doi:10.1371/journal.pone.0194205)
Supplement: S4 Table — (PDF) [file pone.0194205.s004.pdf]

Supplementary Table 4. Up regulated genes in relatives with ACCP+ vs relatives ACCP-

Gene Symbol	Genbank Accession	Gene Name	Fold Change	Regulation
LOC100288292	XR_132538	putative uncharacterized protein FLJ44672-like	2.789923	up
CAV1	NM_001753	caveolin 1, caveolae protein, 22kDa	3.946695	up
CCDC129		coiled-coil domain containing 129	2.1985881	up
ABTB1	NM_032548	ankyrin repeat and BTB (POZ) domain containing 1	2.2613204	up
WSCD2	NM_014653	WSC domain containing 2	2.075865	up
PPP1R14A	NM_033256	protein phosphatase 1, regulatory (inhibitor) subunit 14A	2.6415465	up
ATP6V0D1	NM_004691	ATPase, H+ transporting, lysosomal 38kDa, V0 subunit d1	2.4900744	up
KCNK15	NM_022358	potassium channel, subfamily K, member 15	2.2245746	up
SP2	NM_003110	Sp2 transcription factor	2.6386456	up
KIAA0319L	NM_024874	KIAA0319-like	2.1558642	up
C19orf59	NM_174918	chromosome 19 open reading frame 59	2.3626502	up
SCARF2	NM_153334	scavenger receptor class F, member 2	2.360706	up
Unknown			2.6564271	up
ACBD4	NM_024722	acyl-CoA binding domain containing 4	2.818159	up
NARF	NM_001038618	nuclear prelamin A recognition factor	2.008297	up
	AK094541		2.4117448	up
PCP4	NM_006198	Purkinje cell protein 4	2.489627	up
CIC	NM_015125	capicua homolog (Drosophila)	2.3857553	up
	AK098147		4.7182083	up
IL1R2	NM_004633	interleukin 1 receptor, type II	2.1484475	up
KRTAP10-8	NM_198695	keratin associated protein 10-8	2.4531016	up
TARP	NM_001003799	TCR gamma alternate reading frame protein	2.415881	up
TUBA3D	NM_080386	tubulin, alpha 3d	2.273618	up
LOC283547	NR_039982	uncharacterized LOC283547	2.015791	up
RNASET2	AK001769	ribonuclease T2	4.057167	up
ZNF24	NM_006965	zinc finger protein 24	3.4035223	up
LOC389834	NR_027420	ankyrin repeat domain 57 pseudogene	2.7860982	up
LOC100132247	NM_001135865	nuclear pore complex interacting protein related gene	2.1316926	up
PARP12	NM_022750	poly (ADP-ribose) polymerase family, member 12	2.4150546	up
SH3BP4	NM_014521	SH3-domain binding protein 4	2.079753	up
DDX3Y	NM_001122665	DEAD (Asp-Glu-Ala-Asp) box polypeptide 3, Y-linked	2.0400486	up
SIK1	NM_173354	salt-inducible kinase 1	2.1804388	up
CSN1S1	NM_001890	casein alpha s1	2.5260406	up
ACSL1	NM_001995	acyl-CoA synthetase long-chain family member 1	2.0690696	up
MCOLN3	NM_018298	mucolipin 3	3.3666747	up
LOC100129931	NR_033828	uncharacterized LOC100129931	10.931081	up
Unknown			2.0839288	up
ND3	AY423734	NADH dehydrogenase, subunit 3 (complex I)	2.6191425	up
LCE1A	NM_178348	late cornified envelope 1A	2.3914135	up
APOBEC3C	NM_014508	apolipoprotein B mRNA editing enzyme, catalytic polypeptide-like 3C	2.0509124	up

LOC400662	AK055411	uncharacterized LOC400662	3.3198295	up
ARAP1	NM_001040118	ArfGAP with RhoGAP domain, ankyrin repeat and PH domain 1	2.3279066	up
TNXB	NM_019105	tenascin XB	2.3959095	up
SPOCK2	NM_001134434	sparc/osteonectin, cwcv and kazal-like domains proteoglycan (testican) 2	2.3886817	up
TIPRL	NM_152902	TIP41, TOR signaling pathway regulator-like (S. cerevisiae)	2.6131291	up
ZFP36L1	AK097098	zinc finger protein 36, C3H type-like 1	2.0590303	up
CPSF3L	NM_017871	cleavage and polyadenylation specific factor 3-like	3.3125765	up
ACSS2	NM_018677	acyl-CoA synthetase short-chain family member 2	2.2859967	up
SLC12A9	NM_020246	solute carrier family 12 (potassium/chloride transporters), member 9	2.2835226	up
PLEKHG5	NM_198681	pleckstrin homology domain containing, family G (with RhoGef domain) member 5	2.0799766	up
ARHGEF16	NM_014448	Rho guanine nucleotide exchange factor (GEF) 16	2.4696991	up
TLE4	NM_007005	transducin-like enhancer of split 4 (E(sp1) homolog, Drosophila)	2.0882056	up
HPS1	NM_000195	Hermansky-Pudlak syndrome 1	2.6783736	up
ACTN4	NM_004924	actinin, alpha 4	2.3923786	up
MORN1		MORN repeat containing 1	2.925553	up
ACAP3	NM_030649	ArfGAP with coiled-coil, ankyrin repeat and PH domains 3	2.5301104	up
Unknown			2.5941215	up
COL27A1	NM_032888	collagen, type XXVII, alpha 1	2.9623253	up
CYP4F30P	NR_023391	cytochrome P450, family 4, subfamily F, polypeptide 30, pseudogene	4.3726916	up
LOC647086	XR_132608	chromosome 20 open reading frame 27 pseudogene	2.084827	up
Unknown			2.1183016	up
STAT3	NM_213662	signal transducer and activator of transcription 3 (acute-phase response factor)	2.6298807	up
KRTAP10-12	NM_198699	keratin associated protein 10-12	3.3742194	up
AHSG	NM_001622	alpha-2-HS-glycoprotein	2.4957964	up
ACAP1	NM_014716	ArfGAP with coiled-coil, ankyrin repeat and PH domains 1	2.449008	up
PORCN	NM_203473	porcupine homolog (Drosophila)	3.0370867	up
	DR731407		4.1535273	up
USP5	NM_003481	ubiquitin specific peptidase 5 (isopeptidase T)	2.2778535	up
RLTPR	NM_001013838	RGD motif, leucine rich repeats, tropomodulin domain and proline-rich containing	2.5468485	up
H3F3B	NM_005324	H3 histone, family 3B (H3.3B)	2.164711	up
LOC729305	XR_132809	uncharacterized LOC729305	4.0573115	up
FOXB1	NM_012182	forkhead box B1	2.3309827	up
GDI1	NM_001493	GDP dissociation inhibitor 1	2.2780993	up
DPYSL4	NM_006426	dihydropyrimidinase-like 4	2.5227687	up
Unknown			4.7101107	up
MAP1LC3A	NM_032514	microtubule-associated protein 1 light chain 3 alpha	3.7433934	up
RAB1B	NM_030981	RAB1B, member RAS oncogene family	2.3512714	up
	AK097701		2.2802246	up
LOC143188	NR_015409	uncharacterized LOC143188	2.7468412	up
USP21	NM_001014443	ubiquitin specific peptidase 21	2.651004	up
HMGA2	NM_003484	high mobility group AT-hook 2	3.1459346	up
ST7	NM_018412	suppression of tumorigenicity 7	2.0580974	up

GPR15	NM_005290	G protein-coupled receptor 15	2.6640446	up
GABARAP	NM_007278	GABA(A) receptor-associated protein	2.1371005	up
CHKB	NM_005198	choline kinase beta	2.2299933	up
CD3E	NM_000733	CD3e molecule, epsilon (CD3-TCR complex)	2.1550918	up
NSUN4	NM_199044	NOP2/Sun domain family, member 4	2.6492007	up
LOC100652762	XM_003403544	uncharacterized LOC100652762	2.0573764	up
FAM65A	NM_024519	family with sequence similarity 65, member A	3.2714615	up
BPIFB2	NM_025227	BPI fold containing family B, member 2	4.1397	up
INPPL1	NM_001567	inositol polyphosphate phosphatase-like 1	2.0888195	up
SIRT2	NM_012237	sirtuin 2	2.4678743	up
Unknown			2.8905916	up
KRTAP5-11		keratin associated protein 5-11	2.0622194	up
LINC00494	NR_026958	long intergenic non-protein coding RNA 494	3.0265074	up
LOC401357	XR_132811	uncharacterized LOC401357	2.5916228	up
MON1B	NM_014940	MON1 homolog B (yeast)	2.590523	up
	AY010113		2.2281902	up
CYC1	NM_001916	cytochrome c-1	2.0117774	up
NPPA		natriuretic peptide A	2.9338233	up
EIF1AY	NM_004681	eukaryotic translation initiation factor 1A, Y-linked	5.6645055	up
	BE266556		2.068938	up
TMEM151B	NM_001137560	transmembrane protein 151B	2.4972787	up
LINC00106	BQ009527	long intergenic non-protein coding RNA 106	2.212271	up
AKT1	NM_005163	v-akt murine thymoma viral oncogene homolog 1	2.404629	up
SMAP2	NM_022733	small ArfGAP2	2.2495065	up
REREP3	NR_033735	arginine-glutamic acid dipeptide (RE) repeats pseudogene 3	3.242693	up
Unknown			2.3068695	up
CT47A11	NM_173571	cancer/testis antigen family 47, member A11	2.8958516	up
Unknown			2.2935717	up
EPHX3	NM_024794	epoxide hydrolase 3	2.7596948	up
C1orf198	NM_032800	chromosome 1 open reading frame 198	2.0539234	up
	XM_003118719		2.7333615	up
ACHE	NM_000665	acetylcholinesterase	2.6459744	up
Unknown			2.1197147	up
NASP	NM_002482	nuclear autoantigenic sperm protein (histone-binding)	3.7578866	up
NIP7	NM_016101	nuclear import 7 homolog (S. cerevisiae)	3.1747224	up
COX1	AK074742	cytochrome c oxidase subunit I	2.3615606	up
LOC100131150	AK096194	uncharacterized LOC100131150	2.1258461	up
AUP1	NM_181575	ancient ubiquitous protein 1	2.105954	up
TMEM219	NM_194280	transmembrane protein 219	2.1054459	up
MESTIT1	NR_004382	MEST intronic transcript 1, antisense RNA (non-protein coding)	2.8080826	up
MOV10L1	NM_018995	Mov10l1, Moloney leukemia virus 10-like 1, homolog (mouse)	2.9715996	up
LOC100130238	NR_024563	uncharacterized LOC100130238	2.1249104	up

LOC645261	AF258587	PP565	3.8613176	up
FAM100A	NM_145253	family with sequence similarity 100, member A	2.6317227	up
ZNF205	NM_003456	zinc finger protein 205	3.350588	up
OR5V1	NM_030876	olfactory receptor, family 5, subfamily V, member 1	2.0663216	up
WNT10A	NM_025216	wingless-type MMTV integration site family, member 10A	2.2642982	up
HNRNPUL1	NM_007040	heterogeneous nuclear ribonucleoprotein U-like 1	2.525899	up
	DA197111		2.4724066	up
GUK1	NM_001242840	guanylate kinase 1	2.5769215	up
ZNF266	NM_006631	zinc finger protein 266	2.2760646	up
NKAIN2	NM_001040214	Na+/K+ transporting ATPase interacting 2	2.0606914	up
HMG20B	NM_006339	high mobility group 20B	2.4731197	up
HSP90B1	NM_003299	heat shock protein 90kDa beta (Grp94), member 1	2.1757808	up
TRAM2	NM_012288	translocation associated membrane protein 2	3.66912	up
P4HTM	NM_177938	prolyl 4-hydroxylase, transmembrane (endoplasmic reticulum)	2.2450502	up
TMPRSS9	NM_182973	transmembrane protease, serine 9	3.1652484	up
RHBDL1	NM_003961	rhomboid, veinlet-like 1 (Drosophila)	2.7976906	up
FRMD1	NM_024919	FERM domain containing 1	3.5627968	up
Unknown			2.4173417	up
MRPL45	NM_032351	mitochondrial ribosomal protein L45	2.4917097	up
IFITM3	NM_021034	interferon induced transmembrane protein 3	2.0669284	up
LALBA	NM_002289	lactalbumin, alpha-	7.8349533	up
OAS1	NM_002534	2'-5'-oligoadenylate synthetase 1, 40/46kDa	2.666262	up
CLPTM1	NM_001294	cleft lip and palate associated transmembrane protein 1	2.178986	up
FLJ14186	NR_037596	uncharacterized LOC401149	2.880895	up
KRTAP5-4	NM_001012709	keratin associated protein 5-4	2.263062	up
ARHGAP40	NM_001164431	Rho GTPase activating protein 40	2.3690147	up
CAMK2G	NM_172171	calcium/calmodulin-dependent protein kinase II gamma	2.0102026	up
FAM108A1	NM_001130111	family with sequence similarity 108, member A1	2.0615745	up
NSUN5	NM_148956	NOP2/Sun domain family, member 5	2.051235	up
C18orf12	XR_109479	chromosome 18 open reading frame 12	2.4328592	up
CMTM7	NM_138410	CKLF-like MARVEL transmembrane domain containing 7	3.5308428	up
	AK126423		2.4049344	up
	BC166633		2.3559139	up
KCTD19	NM_001100915	potassium channel tetramerisation domain containing 19	3.0291438	up
FAM73B	NM_032809	family with sequence similarity 73, member B	2.0745895	up
ATG4B	NM_013325	ATG4 autophagy related 4 homolog B (S. cerevisiae)	3.0301833	up
RBAK-LOC389458	NM_001204513	RBAK-LOC389458 readthrough	2.9922888	up
CMA1	NM_001836	chymase 1, mast cell	2.499754	up
OSCAR	NM_206818	osteoclast associated, immunoglobulin-like receptor	2.6062565	up
	BF515046		2.459267	up
ZFP41	NM_173832	zinc finger protein 41 homolog (mouse)	5.1692195	up
TMEM189	NM_199129	transmembrane protein 189	2.7031746	up

AMN	NM_030943	amnionless homolog (mouse)	2.6318595	up
Unknown			2.2459526	up
STX5	NM_003164	syntaxin 5	2.0274286	up
PRDX5	NM_012094	peroxiredoxin 5	2.0816474	up
C19orf29	NM_021231	chromosome 19 open reading frame 29	2.722241	up
CRLF1	NM_004750	cytokine receptor-like factor 1	2.3008592	up
ARHGEF10	BC040474	Rho guanine nucleotide exchange factor (GEF) 10	2.261707	up
ENTPD6	NM_001247	ectonucleoside triphosphate diphosphohydrolase 6 (putative)	2.2502723	up
POLR2J4	NR_003655	polymerase (RNA) II (DNA directed) polypeptide J4, pseudogene	2.494902	up
MLLT11	NM_006818	myeloid/lymphoid or mixed-lineage leukemia (trithorax homolog, Drosophila); transl	2.338549	up
TCOF1	NM_001008657	Treacher Collins-Franceschetti syndrome 1	3.5125248	up
SPATA2L	NM_152339	spermatogenesis associated 2-like	2.2859683	up
SCGB3A2	NM_054023	secretoglobin, family 3A, member 2	3.76579	up
C8orf58	NM_001013842	chromosome 8 open reading frame 58	2.2809741	up
VMO1	NM_182566	vitelline membrane outer layer 1 homolog (chicken)	2.418349	up
HIST1H2BN	NM_003520	histone cluster 1, H2bn	2.3483684	up
EFNA1	NM_004428	ephrin-A1	3.2253997	up
SLC34A1	NM_003052	solute carrier family 34 (sodium phosphate), member 1	2.3626764	up
CORO1A	NM_007074	coronin, actin binding protein, 1A	2.4794939	up
DHRS13	NM_144683	dehydrogenase/reductase (SDR family) member 13	2.491425	up
RPP25	NM_017793	ribonuclease P/MRP 25kDa subunit	2.4927213	up
IL32	NM_001012631	interleukin 32	2.104205	up
GPR97	NM_170776	G protein-coupled receptor 97	2.0263214	up
KCP	NM_001135914	kielin/chordin-like protein	2.9855702	up
Unknown			2.128056	up
GNG8	NM_033258	guanine nucleotide binding protein (G protein), gamma 8	2.0878649	up
CDX1	NM_001804	caudal type homeobox 1	4.2568593	up
OR10H2	NM_013939	olfactory receptor, family 10, subfamily H, member 2	2.1559143	up
PQLC3	NM_152391	PQ loop repeat containing 3	3.0017273	up
BLOC1S1	NM_001487	biogenesis of lysosomal organelles complex-1, subunit 1	2.6000597	up
S100A11	NM_005620	S100 calcium binding protein A11	2.9087212	up
LOC646743	NR_033930	uncharacterized LOC646743	4.124948	up
PNPLA2	NM_020376	patatin-like phospholipase domain containing 2	2.6759987	up
FMO5	NM_001461	flavin containing monooxygenase 5	2.0558357	up
Unknown			3.1459734	up
LOC339807	NR_034023	uncharacterized LOC339807	2.0697885	up
LSS	NM_002340	lanosterol synthase (2,3-oxidosqualene-lanosterol cyclase)	2.2321827	up
Unknown			2.0370793	up
ZNF703	NM_025069	zinc finger protein 703	2.1090167	up
APBB3	NM_006051	amyloid beta (A4) precursor protein-binding, family B, member 3	2.0174665	up
ERGIC3	NM_015966	ERGIC and golgi 3	2.0251486	up
CD37	NM_001774	CD37 molecule	2.4798875	up

DUSP8	NM_004420	dual specificity phosphatase 8	2.4004533	up
ALDH3B1	NM_001161473	aldehyde dehydrogenase 3 family, member B1	2.4100623	up
TMUB2	NM_177441	transmembrane and ubiquitin-like domain containing 2	2.2567334	up
Unknown			3.281102	up
F13A1	NM_000129	coagulation factor XIII, A1 polypeptide	2.5725837	up
AGPAT3	NM_020132	1-acylglycerol-3-phosphate O-acyltransferase 3	2.2452831	up
TTTY15	NR_001545	testis-specific transcript, Y-linked 15 (non-protein coding)	2.273323	up
ASNA1	NM_004317	arsA arsenite transporter, ATP-binding, homolog 1 (bacterial)	2.1891809	up
COL6A2	NM_058174	collagen, type VI, alpha 2	2.97253	up
TMCO6	NM_018502	transmembrane and coiled-coil domains 6	2.09654	up
AK1	NM_000476	adenylate kinase 1	2.3696666	up
TMEM40	NM_018306	transmembrane protein 40	2.4684763	up
MAEA	NM_001017405	macrophage erythroblast attacher	2.2102544	up
CERS3	NM_178842	ceramide synthase 3	2.8725007	up
HTRA2	NM_145074	HtrA serine peptidase 2	2.392647	up
FBXL17	NM_001163315	F-box and leucine-rich repeat protein 17	3.862536	up
GPX1	NM_201397	glutathione peroxidase 1	2.6742394	up
POLR2C	NM_032940	polymerase (RNA) II (DNA directed) polypeptide C, 33kDa	2.3571212	up
C16orf7	NM_004913	chromosome 16 open reading frame 7	2.50614	up
FTL	NM_000146	ferritin, light polypeptide	2.1800663	up
OR11A1	NM_013937	olfactory receptor, family 11, subfamily A, member 1	3.688992	up
ASB16	NM_080863	ankyrin repeat and SOCS box containing 16	2.987806	up
ATP6V0B	NM_004047	ATPase, H+ transporting, lysosomal 21kDa, V0 subunit b	2.1722252	up
FLJ34208	NR_033929	uncharacterized LOC401106	3.5345564	up
SH3GL1	NM_003025	SH3-domain GRB2-like 1	2.5025542	up
FBXL6	NM_012162	F-box and leucine-rich repeat protein 6	2.7470727	up
TNIP1	NM_006058	TNFAIP3 interacting protein 1	2.1397786	up
FTL	NM_000146	ferritin, light polypeptide	2.0166714	up
ZNF282	NM_003575	zinc finger protein 282	3.1631253	up
TMEM146	NM_152784	transmembrane protein 146	2.2819207	up
BCKDK	NM_005881	branched chain ketoacid dehydrogenase kinase	2.3312452	up
KIFC2	NM_145754	kinesin family member C2	2.4249732	up
GNB1	NM_002074	guanine nucleotide binding protein (G protein), beta polypeptide 1	2.0907006	up
ITGAL	NM_002209	integrin, alpha L (antigen CD11A (p180), lymphocyte function-associated antigen 1; a	2.0606594	up
MICU1	NM_006077	mitochondrial calcium uptake 1	2.126319	up
FCER1G	NM_004106	Fc fragment of IgE, high affinity I, receptor for; gamma polypeptide	2.0405056	up
SHISA4	NM_198149	shisa homolog 4 (Xenopus laevis)	2.6683297	up
SLC26A11	NM_001166347	solute carrier family 26, member 11	3.1420128	up
LOC390660	XR_109177	FLJ00317 protein	2.6243894	up
LYL1	NM_005583	lymphoblastic leukemia derived sequence 1	2.8418229	up
			2.385252	up
CDCA4	NM_017955	cell division cycle associated 4	2.8596375	up

C2CD4C	NM_001136263	C2 calcium-dependent domain containing 4C	3.567688	up
AP2B1	NM_001030006	adaptor-related protein complex 2, beta 1 subunit	2.070988	up
ARMC6	NM_033415	armadillo repeat containing 6	2.8686852	up
EVX1	NM_001989	even-skipped homeobox 1	2.6414528	up
	BC033227		2.5524182	up
PARVG	NM_022141	parvin, gamma	2.0445724	up
SLC14A1	NM_001146037	solute carrier family 14 (urea transporter), member 1 (Kidd blood group)	2.0789602	up
DVL2	NM_004422	dishevelled, dsh homolog 2 (Drosophila)	2.0782108	up
GAS7	NM_201433	growth arrest-specific 7	2.1753223	up
CISD3	NM_001136498	CDGSH iron sulfur domain 3	2.2889295	up
ZMYND8	NM_183047	zinc finger, MYND-type containing 8	2.519038	up
LRRC33	NM_198565	leucine rich repeat containing 33	2.1911287	up
CSF1	NM_172212	colony stimulating factor 1 (macrophage)	2.5548365	up
RHBDD2	NM_001040457	rhomboid domain containing 2	2.2276585	up
DUSP4	NM_001394	dual specificity phosphatase 4	3.9018173	up
NXF1	NM_006362	nuclear RNA export factor 1	2.2717564	up
NPB	NM_148896	neuropeptide B	2.888444	up
Unknown			2.6223218	up
AGBL3	NM_178563	ATP/GTP binding protein-like 3	2.486592	up
ALG1	NM_019109	asparagine-linked glycosylation 1, beta-1,4-mannosyltransferase homolog (S. cerevis	2.02872	up
SSH1	NM_001161331	slingshot homolog 1 (Drosophila)	2.13739	up
LTK	NM_002344	leukocyte receptor tyrosine kinase	2.1800156	up
SH3GLB2	NM_020145	SH3-domain GRB2-like endophilin B2	2.5247235	up
ALKBH5	NM_017758	alkB, alkylation repair homolog 5 (E. coli)	2.9174528	up
SYTL1	NM_032872	synaptotagmin-like 1	2.5048254	up
NOA1	NM_032313	nitric oxide associated 1	2.5846655	up
SREBF1	NM_001005291	sterol regulatory element binding transcription factor 1	2.2380853	up
Unknown			2.786228	up
DOT1L	AB058717	DOT1-like, histone H3 methyltransferase (S. cerevisiae)	3.5372963	up
MYH14	NM_001077186	myosin, heavy chain 14, non-muscle	2.6250458	up
NOTO	NM_001134462	notochord homeobox	3.2267942	up
VWA1	NM_022834	von Willebrand factor A domain containing 1	2.404687	up
Unknown			2.374378	up
KLHDC7B	NM_138433	kelch domain containing 7B	2.8690758	up
FAM108A1	NM_031213	family with sequence similarity 108, member A1	2.2581694	up
PCSK1N	NM_013271	proprotein convertase subtilisin/kexin type 1 inhibitor	3.5376828	up
C20orf123	NM_080721	chromosome 20 open reading frame 123	2.2385967	up
CYTB	HV444967	cytochrome b	3.181024	up
Unknown			2.3991928	up
HTT	NM_002111	huntingtin	2.2714274	up
PRB3	NM_006249	proline-rich protein BstNI subfamily 3	4.755131	up
C9orf173	NM_001004353	chromosome 9 open reading frame 173	2.6211097	up

RTDR1		rhabdoid tumor deletion region gene 1	2.0407968	up
AFAP1	NM_001134647	actin filament associated protein 1	2.3948164	up
LOC221814	AL122087	uncharacterized LOC221814	3.0290005	up
DUSP15	NM_080611	dual specificity phosphatase 15	2.0299642	up
PDLIM2	NM_198042	PDZ and LIM domain 2 (mystique)	2.1478093	up
RAE1	NM_001015885	RAE1 RNA export 1 homolog (S. pombe)	3.5306275	up
RAVER1	NM_133452	ribonucleoprotein, PTB-binding 1	2.488028	up
MYO1G	NM_033054	myosin IG	2.3639238	up
BAG6	NM_004639	BCL2-associated athanogene 6	2.1358933	up
LRG1	NM_052972	leucine-rich alpha-2-glycoprotein 1	2.6347952	up
TLE1	NM_005077	transducin-like enhancer of split 1 (E(sp1) homolog, Drosophila)	4.284191	up
SPAG1	NM_003114	sperm associated antigen 1	2.3289213	up
RNF40	NM_014771	ring finger protein 40	2.1602867	up
	XR_132817		2.1748853	up
DHCR24	NM_014762	24-dehydrocholesterol reductase	2.3491774	up
FLJ14186	NR_037596	uncharacterized LOC401149	2.4611003	up
LOC100131094	NM_001242901	uncharacterized LOC100131094	2.031078	up
PSMC4	NM_006503	proteasome (prosome, macropain) 26S subunit, ATPase, 4	2.2124834	up
KRT73	NM_175068	keratin 73	2.2522876	up
PPP5C	NM_006247	protein phosphatase 5, catalytic subunit	2.1869056	up
ENTPD7	NM_020354	ectonucleoside triphosphate diphosphohydrolase 7	2.025124	up
Unknown			2.2636058	up
IDH3G	NM_004135	isocitrate dehydrogenase 3 (NAD+) gamma	2.0241475	up
LINC00265	NR_026999	long intergenic non-protein coding RNA 265	2.215186	up
TMEM8A	NM_021259	transmembrane protein 8A	2.0625715	up
GPR132	NM_013345	G protein-coupled receptor 132	5.3407106	up
TPP1	NM_000391	tripeptidyl peptidase I	3.2587173	up
KRT85	NM_002283	keratin 85	4.8867793	up
SMCR2	AI821758	Smith-Magenis syndrome chromosome region, candidate 2 (non-protein coding)	2.1187375	up
SNED1	NM_001080437	sushi, nidogen and EGF-like domains 1	2.4987593	up
ETV3L	NM_001004341	ets variant 3-like	2.391817	up
CREB3L1	NM_052854	cAMP responsive element binding protein 3-like 1	3.4514906	up
POLD4	NM_021173	polymerase (DNA-directed), delta 4	2.0392158	up
ITGB2	L78790	integrin, beta 2 (complement component 3 receptor 3 and 4 subunit)	2.0947294	up
PPP1R14B	NM_138689	protein phosphatase 1, regulatory (inhibitor) subunit 14B	2.1118708	up
TAAR2	NM_001033080	trace amine associated receptor 2	2.1933281	up
TNFRSF4	NM_003327	tumor necrosis factor receptor superfamily, member 4	2.6528015	up
DPM2	NM_003863	dolichyl-phosphate mannosyltransferase polypeptide 2, regulatory subunit	2.3329062	up
CST3	NM_000099	cystatin C	3.2337716	up
NDUFB7	NM_004146	NADH dehydrogenase (ubiquinone) 1 beta subcomplex, 7, 18kDa	3.8067162	up
RASGRP4	NM_170604	RAS guanyl releasing protein 4	2.0746405	up
MTMR14	AK128312	myotubularin related protein 14	2.923553	up

MOCS3	NM_014484	molybdenum cofactor synthesis 3	3.040179	up
FLJ25694	AK127969	uncharacterized protein FLJ25694	2.9436	up
UNC93B1	NM_030930	unc-93 homolog B1 (C. elegans)	2.018595	up
ZNF641	NM_152320	zinc finger protein 641	2.0046716	up
JUNB	NM_002229	jun B proto-oncogene	3.4909914	up
OR2H1	NM_030883	olfactory receptor, family 2, subfamily H, member 1	2.346983	up
FLJ36000	NR_027084	uncharacterized FLJ36000	2.0665047	up
RPRD1B	NM_021215	regulation of nuclear pre-mRNA domain containing 1B	2.3433013	up
RPP30	NM_006413	ribonuclease P/MRP 30kDa subunit	2.0619528	up
HERC6	NM_017912	hect domain and RLD 6	3.3713746	up
ZNF771	NM_016643	zinc finger protein 771	2.0332878	up
SLC25A39	NM_016016	solute carrier family 25, member 39	2.6531897	up
PACS1	NM_018026	phosphofurin acidic cluster sorting protein 1	2.386689	up
LOC157740	AJ291676	uncharacterized protein C8orf9	2.9273436	up
OR5AP2	NM_001002925	olfactory receptor, family 5, subfamily AP, member 2	4.2860036	up
SRPR	NM_003139	signal recognition particle receptor (docking protein)	2.3204548	up
TEX261	NM_144582	testis expressed 261	2.8641853	up
SSR2	NM_003145	signal sequence receptor, beta (translocon-associated protein beta)	2.021808	up
FLJ45445	NR_028324	uncharacterized LOC399844	2.2271838	up
SLC22A23	NM_021945	solute carrier family 22, member 23	2.4369624	up
TAPBP	NM_003190	TAP binding protein (tapasin)	2.0152535	up
SPAG8	NM_001039592	sperm associated antigen 8	2.289541	up
B4GALT5	NM_004776	UDP-Gal:betaGlcNAc beta 1,4- galactosyltransferase, polypeptide 5	2.082269	up
REPIN1	NM_014374	replication initiator 1	2.045336	up
MLL4	NM_014727	myeloid/lymphoid or mixed-lineage leukemia 4	2.1333902	up
	AK093659		2.1559415	up
CST7	NM_003650	cystatin F (leukocystatin)	2.5456247	up
FKBP1A	NM_000801	FK506 binding protein 1A, 12kDa	2.498522	up
CDA	NM_001785	cytidine deaminase	3.051612	up
CHD1L	NM_004284	chromodomain helicase DNA binding protein 1-like	2.700532	up
CPLX2		complexin 2	2.4863415	up
CUX1	NM_001913	cut-like homeobox 1	2.3591845	up
SLC22A31	NM_001242757	solute carrier family 22, member 31	2.099208	up
C10orf27	NM_152710	chromosome 10 open reading frame 27	5.202161	up
FLJ14186	NR_037596	uncharacterized LOC401149	3.195986	up
POLR2J	NM_006234	polymerase (RNA) II (DNA directed) polypeptide J, 13.3kDa	2.1569586	up
C19orf56	NM_016145	chromosome 19 open reading frame 56	2.082174	up
FTSJD2	NM_015050	FtsJ methyltransferase domain containing 2	2.420377	up
FLJ45445	NR_028324	uncharacterized LOC399844	2.1531484	up
STAG3L2	NR_040584	stromal antigen 3-like 2	2.5088859	up
LOC100132593	AK098270	uncharacterized LOC100132593	2.0571446	up
			2.6352627	up

JKAMP	NM_016475	JNK1/MAPK8-associated membrane protein	2.2222292	up
	XM_001719321		3.041666	up
TAGLN	NM_001001522	transgelin	3.3945303	up
KIF3B	NM_004798	kinesin family member 3B	2.1783493	up
OTOA	BC040551	otoancorin	2.5205932	up
UBE2G2	NM_182688	ubiquitin-conjugating enzyme E2G 2	2.1712408	up
AIF1	NM_004847	allograft inflammatory factor 1	2.4536178	up
EIF4G1	NM_182917	eukaryotic translation initiation factor 4 gamma, 1	2.2555962	up
GLIS1	NM_147193	GLIS family zinc finger 1	2.0432203	up
HPS1	NM_182639	Hermansky-Pudlak syndrome 1	2.915498	up
PQBP1	NM_001167989	polyglutamine binding protein 1	4.7224183	up
S100P	NM_005980	S100 calcium binding protein P	3.8260586	up
	D13077		2.2005086	up
TMC8	NM_152468	transmembrane channel-like 8	2.0550494	up
	CR737729		2.1551185	up
GAPDH	NM_002046	glyceraldehyde-3-phosphate dehydrogenase	2.036496	up
	AK021933		4.567646	up
PRPS1	NM_002764	phosphoribosyl pyrophosphate synthetase 1	3.0220182	up
LOC284926	CR624447	uncharacterized LOC284926	2.06371	up
ATP1A3	NM_152296	ATPase, Na+/K+ transporting, alpha 3 polypeptide	2.8927448	up
SPNS1	NM_032038	spinster homolog 1 (Drosophila)	2.1965334	up
ACADVL	NM_000018	acyl-CoA dehydrogenase, very long chain	2.009936	up
WLS	NM_024911	wntless homolog (Drosophila)	2.294251	up
LOC100132249	XR_132823	uncharacterized LOC100132249	2.4884002	up
FLOT2	NM_004475	flotillin 2	2.794187	up
UBL7	NM_032907	ubiquitin-like 7 (bone marrow stromal cell-derived)	2.6817155	up
SIRT3	AK074992	sirtuin 3	2.433562	up
Unknown			2.1971831	up
AHSA2	BC050395	AHA1, activator of heat shock 90kDa protein ATPase homolog 2 (yeast)	4.2361674	up
LRRC16B	NM_138360	leucine rich repeat containing 16B	2.6115358	up
C19orf66	NM_018381	chromosome 19 open reading frame 66	2.6281729	up
HIF3A	AB118749	hypoxia inducible factor 3, alpha subunit	2.4027464	up
			3.4199228	up
DENND2A	NM_015689	DENN/MADD domain containing 2A	2.766787	up
FLJ45445	NR_028324	uncharacterized LOC399844	2.2747633	up
ACADS	NM_000017	acyl-CoA dehydrogenase, C-2 to C-3 short chain	2.0657194	up
DNAJC27-AS1	NR_034113	DNAJC27 antisense RNA 1 (non-protein coding)	2.3628697	up
Unknown			2.5952158	up
CEP104	BC050721	centrosomal protein 104kDa	6.9731812	up
DCTN3	NM_007234	dynactin 3 (p22)	2.1977496	up
C2orf63		chromosome 2 open reading frame 63	2.1694813	up
LOC727721	DB238770	uncharacterized LOC727721	3.2233636	up

CHST13	NM_152889	carbohydrate (chondroitin 4) sulfotransferase 13	2.4717147	up
TTC39A	NM_001080494	tetratricopeptide repeat domain 39A	2.0910904	up
CDC34	NM_004359	cell division cycle 34 homolog (S. cerevisiae)	2.3891041	up
SSBP3	NM_001009955	single stranded DNA binding protein 3	2.6869538	up
C4orf40	NM_214711	chromosome 4 open reading frame 40	3.4621024	up
CABP5	NM_019855	calcium binding protein 5	2.693108	up
	BX398892		2.6134462	up
LOC100288293	XR_132539	putative uncharacterized protein FLJ44672-like	2.608739215	up
CAV2	NM_001753	caveolin 1, caveolae protein, 22kDa	2.608559004	up
CCDC130		coiled-coil domain containing 130	2.608378792	up
ABTB2	NM_032548	ankyrin repeat and BTB (POZ) domain containing 2	2.608198581	up
WSCD3	NM_014653	WSC domain containing 3	2.608018369	up
PPP1R14A	NM_033256	protein phosphatase 1, regulatory (inhibitor) subunit 14A	2.607838158	up
ATP6V0D2	NM_004691	ATPase, H+ transporting, lysosomal 38kDa, V0 subunit d2	2.607657946	up
KCNK16	NM_022358	potassium channel, subfamily K, member 16	2.607477735	up
SP3	NM_003110	Sp2 transcription factor	2.607297523	up
KIAA0319L	NM_024874	KIAA0319-like	2.607117312	up
C19orf60	NM_174918	chromosome 19 open reading frame 60	2.606937101	up
SCARF3	NM_153334	scavenger receptor class F, member 3	2.606756889	up
Unknown			2.606576678	up
ACBD5	NM_024722	acyl-CoA binding domain containing 5	2.606396466	up
NARF	NM_001038618	nuclear prelamin A recognition factor	2.606216255	up
	AK094542		2.606036043	up
PCP5	NM_024052	Purkinje cell protein 5	2.605855832	up
CIC	NM_032979	capicua homolog (Drosophila)	2.605675621	up
	AK098148		2.605495409	up
IL1R3	NM_004633	interleukin 1 receptor, type II	2.605315198	up
KRTAP10-9	NM_198695	keratin associated protein 10-9	2.605134986	up
TARP	NM_001003799	TCR gamma alternate reading frame protein	2.604954775	up
TUBA3D	NM_080386	tubulin, alpha 3d	2.604774563	up
LOC283548	NR_039983	uncharacterized LOC283548	2.604594352	up
RNASET3	AK001770	ribonuclease T3	2.60441414	up
ZNF25	NM_006966	zinc finger protein 25	2.604233929	up
LOC199874660	NR_027421	ankyrin repeat domain 57 pseudogene	2.604053718	up
LOC299617073	NM_001135865	nuclear pore complex interacting protein related gene	2.603873506	up
PARP13	NM_022750	poly (ADP-ribose) polymerase family, member 13	2.603693295	up
SH3BP5	NM_014521	SH3-domain binding protein 5	2.603513083	up
DDX3Y	NM_001122665	DEAD (Asp-Glu-Ala-Asp) box polypeptide 3, Y-linked	2.603332872	up
SIK2	NM_173354	salt-inducible kinase 2	2.60315266	up
CSN1S2	NM_001890	casein alpha s2	2.602972449	up
ACSL2	NM_001995	acyl-CoA synthetase long-chain family member 2	2.602792238	up
MCOLN4	NM_018298	mucolipin 4	2.602612026	up

LOC100129932	NR_033829	uncharacterized LOC100129932	2.602431815	up
			2.602251603	up
ND4	AY423735	NADH dehydrogenase, subunit 3 (complex I)	2.602071392	up
LCE1A	NM_149332	late cornified envelope 1A	2.60189118	up
APOBEC3C	NM_313172	apolipoprotein B mRNA editing enzyme, catalytic polypeptide-like 3C	2.601710969	up
LOC400663	AK055412	uncharacterized LOC400663	2.601530757	up
ARAP2	NM_001040118	ArfGAP with RhoGAP domain, ankyrin repeat and PH domain 2	2.601350546	up
TNXB	NM_019105	tenascin XB	2.601170335	up
SPOCK3	NM_001134434	sparc/osteonectin, cwcv and kazal-like domains proteoglycan (testican) 3	2.600990123	up
TIPRL	NM_152902	TIP41, TOR signaling pathway regulator-like (S. cerevisiae)	2.600809912	up
ZFP36L2	AK097099	zinc finger protein 36, C3H type-like 2	2.6006297	up
CPSF3L	NM_017871	cleavage and polyadenylation specific factor 3-like	2.600449489	up
ACSS3	NM_018677	acyl-CoA synthetase short-chain family member 3	2.600269277	up
SLC12A10	NM_020246	solute carrier family 12 (potassium/chloride transporters), member 10	2.600089066	up
PLEKHG6	NM_198681	pleckstrin homology domain containing, family G (with RhoGef domain) member 6	2.599908855	up
ARHGEF17	NM_014448	Rho guanine nucleotide exchange factor (GEF) 17	2.599728643	up
TLE5	NM_007005	transducin-like enhancer of split 4 (E(sp1) homolog, Drosophila)	2.599548432	up
HPS2	NM_000195	Hermansky-Pudlak syndrome 2	2.59936822	up
ACTN5	NM_004924	actinin, alpha 5	2.599188009	up
MORN2		MORN repeat containing 2	2.599007797	up
ACAP4	NM_030649	ArfGAP with coiled-coil, ankyrin repeat and PH domains 4	2.598827586	up
Unknown			2.598647374	up
COL27A2	NM_032888	collagen, type XXVII, alpha 2	2.598467163	up
CYP4F30P	NR_023392	cytochrome P450, family 4, subfamily F, polypeptide 30, pseudogene	2.598286952	up
LOC647087	XR_132609	chromosome 20 open reading frame 27 pseudogene	2.59810674	up
Unknown			2.597926529	up
STAT4	NM_213662	signal transducer and activator of transcription 3 (acute-phase response factor)	2.597746317	up
KRTAP10-13	NM_198699	keratin associated protein 10-13	2.597566106	up
AHSG	NM_001622	alpha-2-HS-glycoprotein	2.597385894	up
ACAP2	NM_014716	ArfGAP with coiled-coil, ankyrin repeat and PH domains 2	2.597205683	up
PORCN	NM_203473	porcupine homolog (Drosophila)	2.597025472	up
	DR731408		2.59684526	up
USP6	NM_003481	ubiquitin specific peptidase 5 (isopeptidase T)	2.596665049	up
RLTPR	NM_001013838	RGD motif, leucine rich repeats, tropomodulin domain and proline-rich containing	2.596484837	up
H3F3B	NM_005324	H3 histone, family 3B (H3.3B)	2.596304626	up
LOC729306	XR_132810	uncharacterized LOC729306	2.596124414	up
FOXB2	NM_012182	forkhead box B2	2.595944203	up
GDI2	NM_001493	GDP dissociation inhibitor 2	2.595763991	up
DPYSL5	NM_006426	dihydropyrimidinase-like 5	2.59558378	up
			2.595403569	up
MAP1LC3A	NM_032514	microtubule-associated protein 1 light chain 3 alpha	2.595223357	up
RAB1B	NM_030981	RAB1B, member RAS oncogene family	2.595043146	up

	AK097702		2.594862934	up
LOC143189	NR_015410	uncharacterized LOC143189	2.594682723	up
USP22	NM_001014443	ubiquitin specific peptidase 22	2.594502511	up
HMGA3	NM_003484	high mobility group AT-hook 3	2.5943223	up
ST8	NM_018412	suppression of tumorigenicity 8	2.594142089	up
GPR16	NM_005290	G protein-coupled receptor 16	2.593961877	up
GABARAP	NM_007278	GABA(A) receptor-associated protein	2.593781666	up
CHKB	NM_005198	choline kinase beta	2.593601454	up
CD3E	NM_000733	CD3e molecule, epsilon (CD3-TCR complex)	2.593421243	up
NSUN5	NM_199044	NOP2/Sun domain family, member 5	2.593241031	up
LOC100652763	XM_003403545	uncharacterized LOC100652763	2.59306082	up
FAM65A	NM_024519	family with sequence similarity 65, member A	2.592880608	up
BPIFB3	NM_025227	BPI fold containing family B, member 3	2.592700397	up
INPPL2	NM_001567	inositol polyphosphate phosphatase-like 2	2.592520186	up
SIRT3	NM_012237	sirtuin 3	2.592339974	up
Unknown			2.592159763	up
KRTAP5-12		keratin associated protein 5-12	2.591979551	up
LINC00495	NR_026959	long intergenic non-protein coding RNA 495	2.59179934	up
LOC401358	XR_132812	uncharacterized LOC401358	2.591619128	up
MON1B	NM_014941	MON1 homolog B (yeast)	2.591438917	up
	AY010114		2.591258705	up
CYC2	NM_007446	cytochrome c-2	2.591078494	up
NPPA		natriuretic peptide A	2.590898283	up
EIF1AY	NM_010211	eukaryotic translation initiation factor 1A, Y-linked	2.590718071	up
	BE266557		2.59053786	up
TMEM151B	NM_001137561	transmembrane protein 151B	2.590357648	up
LINC00107	BQ009528	long intergenic non-protein coding RNA 107	2.590177437	up
AKT2	NM_040303	v-akt murine thymoma viral oncogene homolog 2	2.589997225	up
SMAP3	NM_057873	small ArfGAP3	2.589817014	up
REREP4	NR_033736	arginine-glutamic acid dipeptide (RE) repeats pseudogene 4	2.589636803	up
Unknown			2.589456591	up
CT47A12	NM_173571	cancer/testis antigen family 47, member A12	2.58927638	up
Unknown			2.589096168	up
EPHX4	NM_024794	epoxide hydrolase 4	2.588915957	up
C1orf199	NM_032800	chromosome 1 open reading frame 199	2.588735745	up
	XM_003118720		2.588555534	up
ACHE	NM_000665	acetylcholinesterase	2.588375322	up
Unknown			2.588195111	up
NASP	NM_002482	nuclear autoantigenic sperm protein (histone-binding)	2.5880149	up
NIP8	NM_016101	nuclear import 7 homolog (S. cerevisiae)	2.587834688	up
COX2	AK117646	cytochrome c oxidase subunit I	2.587654477	up
LOC100131151	AK139098	uncharacterized LOC100131151	2.587474265	up

AUP2	NM_206985	ancient ubiquitous protein 2	2.587294054	up
TMEM220	NM_219690	transmembrane protein 220	2.587113842	up
MESTIT2	NR_004383	MEST intronic transcript 1, antisense RNA (non-protein coding)	2.586933631	up
MOV10L2	NM_018996	Mov10l1, Moloney leukemia virus 10-like 1, homolog (mouse)	2.58675342	up
LOC98839716	NR_024564	uncharacterized LOC100130239	2.586573208	up
LOC198324693	AF258588	PP566	2.586392997	up
FAM100A	NM_145253	family with sequence similarity 100, member A	2.586212785	up
ZNF206	NM_003456	zinc finger protein 206	2.586032574	up
OR5V2	NM_030876	olfactory receptor, family 5, subfamily V, member 2	2.585852362	up
WNT10A	NM_025216	wingless-type MMTV integration site family, member 10A	2.585672151	up
HNRNPUL2	NM_007040	heterogeneous nuclear ribonucleoprotein U-like 2	2.585491939	up
	DA197112		2.585311728	up
GUK2	NM_001242840	guanylate kinase 2	2.585131517	up
ZNF267	NM_006631	zinc finger protein 267	2.584951305	up
NKAIN3	NM_001040214	Na+/K+ transporting ATPase interacting 3	2.584771094	up
HMG20B	NM_006339	high mobility group 20B	2.584590882	up
HSP90B2	NM_003299	heat shock protein 90kDa beta (Grp94), member 2	2.584410671	up
TRAM3	NM_012288	translocation associated membrane protein 3	2.584230459	up
P4HTM	NM_177938	prolyl 4-hydroxylase, transmembrane (endoplasmic reticulum)	2.584050248	up
TMPRSS10	NM_182973	transmembrane protease, serine 10	2.583870037	up
RHBDL2	NM_003961	rhomboid, veinlet-like 1 (Drosophila)	2.583689825	up
FRMD2	NM_024919	FERM domain containing 2	2.583509614	up
Unknown			2.583329402	up
MRPL46	NM_032351	mitochondrial ribosomal protein L46	2.583149191	up
IFITM4	NM_021034	interferon induced transmembrane protein 4	2.582968979	up
LALBA	NM_002289	lactalbumin, alpha-	2.582788768	up
OAS2	NM_002534	2'-5'-oligoadenylate synthetase 1, 40/46kDa	2.582608556	up
CLPTM2	NM_001294	cleft lip and palate associated transmembrane protein 2	2.582428345	up
FLJ14187	NR_037597	uncharacterized LOC401150	2.582248134	up
KRTAP5-5	NM_001012709	keratin associated protein 5-5	2.582067922	up
ARHGAP41	NM_001164431	Rho GTPase activating protein 41	2.581887711	up
CAMK2G	NM_172171	calcium/calmodulin-dependent protein kinase II gamma	2.581707499	up
FAM108A2	NM_001130111	family with sequence similarity 108, member A2	2.581527288	up
NSUN6	NM_148956	NOP2/Sun domain family, member 6	2.581347076	up
C18orf13	XR_109480	chromosome 18 open reading frame 13	2.581166865	up
CMTM8	NM_138411	CKLF-like MARVEL transmembrane domain containing 8	2.580986654	up
	AK126424		2.580806442	up
	BC166634		2.580626231	up
KCTD20	NM_001100915	potassium channel tetramerisation domain containing 20	2.580446019	up
FAM73B	NM_032809	family with sequence similarity 73, member B	2.580265808	up
ATG4B	NM_013325	ATG4 autophagy related 4 homolog B (S. cerevisiae)	2.580085596	up
RBAK-LOC389459	NM_001204513	RBAK-LOC389458 readthrough	2.579905385	up

CMA2	NM_001836	chymase 1, mast cell	2.579725173	up
OSCAR	NM_206818	osteoclast associated, immunoglobulin-like receptor	2.579544962	up
	BF515047		2.579364751	up
ZFP42	NM_173832	zinc finger protein 41 homolog (mouse)	2.579184539	up
TMEM190	NM_199129	transmembrane protein 190	2.579004328	up
AMN	NM_030943	amnionless homolog (mouse)	2.578824116	up
Unknown			2.578643905	up
STX6	NM_003164	syntaxin 6	2.578463693	up
PRDX6	NM_012094	peroxiredoxin 6	2.578283482	up
C19orf30	NM_021231	chromosome 19 open reading frame 30	2.578103271	up
CRLF2	NM_004750	cytokine receptor-like factor 2	2.577923059	up
ARHGEF11	BC040475	Rho guanine nucleotide exchange factor (GEF) 11	2.577742848	up
ENTPD7	NM_001248	ectonucleoside triphosphate diphosphohydrolase 6 (putative)	2.577562636	up
POLR2J5	NR_003656	polymerase (RNA) II (DNA directed) polypeptide J4, pseudogene	2.577382425	up
MLLT12	NM_006818	myeloid/lymphoid or mixed-lineage leukemia (trithorax homolog, Drosophila); transl	2.577202213	up
TCOF2	NM_001008657	Treacher Collins-Franceschetti syndrome 2	2.577022002	up
SPATA2L	NM_152339	spermatogenesis associated 2-like	2.57684179	up
SCGB3A3	NM_054023	secretoglobin, family 3A, member 3	2.576661579	up
C8orf59	NM_001013842	chromosome 8 open reading frame 59	2.576481368	up
VMO2	NM_182566	vitelline membrane outer layer 1 homolog (chicken)	2.576301156	up
HIST1H2BN	NM_003520	histone cluster 1, H2bn	2.576120945	up
EFNA2	NM_004428	ephrin-A2	2.575940733	up
SLC34A2	NM_003052	solute carrier family 34 (sodium phosphate), member 2	2.575760522	up
CORO1A	NM_007074	coronin, actin binding protein, 1A	2.57558031	up
DHRS14	NM_144683	dehydrogenase/reductase (SDR family) member 14	2.575400099	up
RPP26	NM_017793	ribonuclease P/MRP 25kDa subunit	2.575219888	up
IL33	NM_001012631	interleukin 33	2.575039676	up
GPR98	NM_170776	G protein-coupled receptor 98	2.574859465	up
KCP	NM_001135914	kielin/chordin-like protein	2.574679253	up
Unknown			2.574499042	up
GNG9	NM_033258	guanine nucleotide binding protein (G protein), gamma 9	2.57431883	up
CDX2	NM_001804	caudal type homeobox 2	2.574138619	up
OR10H3	NM_013939	olfactory receptor, family 10, subfamily H, member 3	2.573958407	up
PQLC4	NM_152391	PQ loop repeat containing 4	2.573778196	up
BLOC1S2	NM_001487	biogenesis of lysosomal organelles complex-1, subunit 2	2.573597985	up
S100A12	NM_005620	S100 calcium binding protein A12	2.573417773	up
LOC646744	NR_033931	uncharacterized LOC646744	2.573237562	up
PNPLA3	NM_017454	patatin-like phospholipase domain containing 3	2.57305735	up
FMO6	NM_036369	flavin containing monooxygenase 6	2.572877139	up
Unknown			2.572696927	up
LOC339808	NR_034024	uncharacterized LOC339808	2.572516716	up
LSS	NM_002340	lanosterol synthase (2,3-oxidosqualene-lanosterol cyclase)	2.572336505	up

			2.572156293	up
ZNF704	NM_025069	zinc finger protein 704	2.571976082	up
APBB4	NM_006051	amyloid beta (A4) precursor protein-binding, family B, member 4	2.57179587	up
ERGIC4	NM_015966	ERGIC and golgi 4	2.571615659	up
CD38	NM_001774	CD37 molecule	2.571435447	up
DUSP9	NM_004420	dual specificity phosphatase 9	2.571255236	up
ALDH3B2	NM_001161473	aldehyde dehydrogenase 3 family, member B2	2.571075024	up
TMUB3	NM_177441	transmembrane and ubiquitin-like domain containing 3	2.570894813	up
Unknown			2.570714602	up
F13A2	NM_000129	coagulation factor XIII, A1 polypeptide	2.57053439	up
AGPAT4	NM_020132	1-acylglycerol-3-phosphate O-acyltransferase 4	2.570354179	up
TTTY16	NR_001546	testis-specific transcript, Y-linked 15 (non-protein coding)	2.570173967	up
ASNA2	NM_004317	arsA arsenite transporter, ATP-binding, homolog 1 (bacterial)	2.569993756	up
COL6A3	NM_058174	collagen, type VI, alpha 3	2.569813544	up
TMCO7	NM_018502	transmembrane and coiled-coil domains 7	2.569633333	up
AK2	NM_000476	adenylate kinase 2	2.569453121	up
TMEM41	NM_018306	transmembrane protein 41	2.56927291	up
MAEA	NM_001017405	macrophage erythroblast attacher	2.569092699	up
CERS4	NM_178842	ceramide synthase 4	2.568912487	up
HTRA3	NM_145074	HtrA serine peptidase 3	2.568732276	up
FBXL18	NM_001163315	F-box and leucine-rich repeat protein 18	2.568552064	up
GPX2	NM_201397	glutathione peroxidase 2	2.568371853	up
POLR2C	NM_032940	polymerase (RNA) II (DNA directed) polypeptide C, 33kDa	2.568191641	up
C16orf8	NM_004913	chromosome 16 open reading frame 8	2.56801143	up
FTL	NM_000146	ferritin, light polypeptide	2.567831219	up
OR11A2	NM_013937	olfactory receptor, family 11, subfamily A, member 2	2.567651007	up
ASB17	NM_080863	ankyrin repeat and SOCS box containing 17	2.567470796	up
ATP6V0B	NM_004047	ATPase, H+ transporting, lysosomal 21kDa, V0 subunit b	2.567290584	up
FLJ34209	NR_033930	uncharacterized LOC401107	2.567110373	up
SH3GL2	NM_003025	SH3-domain GRB2-like 2	2.566930161	up
FBXL7	NM_012162	F-box and leucine-rich repeat protein 7	2.56674995	up
TNIP2	NM_006058	TNFAIP3 interacting protein 2	2.566569738	up
FTL	NM_000146	ferritin, light polypeptide	2.566389527	up
ZNF283	NM_003575	zinc finger protein 283	2.566209316	up
TMEM147	NM_152784	transmembrane protein 147	2.566029104	up
BCKDK	NM_005881	branched chain ketoacid dehydrogenase kinase	2.565848893	up
KIFC3	NM_145754	kinesin family member C3	2.565668681	up
GNB2	NM_002074	guanine nucleotide binding protein (G protein), beta polypeptide 2	2.56548847	up
ITGAL	NM_002209	integrin, alpha L (antigen CD11A (p180), lymphocyte function-associated antigen 1; a	2.565308258	up
MICU2	NM_006077	mitochondrial calcium uptake 2	2.565128047	up
FCER1G	NM_004106	Fc fragment of IgE, high affinity I, receptor for; gamma polypeptide	2.564947836	up
SHISA5	NM_198149	shisa homolog 4 (Xenopus laevis)	2.564767624	up

SLC26A12	NM_001166347	solute carrier family 26, member 12	2.564587413	up
LOC390661	XR_109178	FLJ00317 protein	2.564407201	up
LYL2	NM_005583	lymphoblastic leukemia derived sequence 2	2.56422699	up
Unknown			2.564046778	up
CDCA5	NM_017955	cell division cycle associated 5	2.563866567	up
C2CD4C	NM_001136263	C2 calcium-dependent domain containing 4C	2.563686355	up
AP2B2	NM_001030006	adaptor-related protein complex 2, beta 1 subunit	2.563506144	up
ARMC7	NM_033415	armadillo repeat containing 7	2.563325933	up
EVX2	NM_001989	even-skipped homeobox 2	2.563145721	up
	BC033228		2.56296551	up
PARVG	NM_022141	parvin, gamma	2.562785298	up
SLC14A2	NM_001146037	solute carrier family 14 (urea transporter), member 1 (Kidd blood group)	2.562605087	up
DVL3	NM_004422	dishevelled, dsh homolog 2 (Drosophila)	2.562424875	up
GAS8	NM_201433	growth arrest-specific 8	2.562244664	up
CISD4	NM_001136498	CDGSH iron sulfur domain 4	2.562064453	up
ZMYND9	NM_183047	zinc finger, MYND-type containing 9	2.561884241	up
LRRC34	NM_198565	leucine rich repeat containing 34	2.56170403	up
CSF2	NM_172212	colony stimulating factor 1 (macrophage)	2.561523818	up
RHBDD3	NM_001040457	rhomboid domain containing 3	2.561343607	up
DUSP5	NM_001394	dual specificity phosphatase 5	2.561163395	up
NXF2	NM_006362	nuclear RNA export factor 2	2.560983184	up
NPB	NM_148896	neuropeptide B	2.560802972	up
Unknown			2.560622761	up
AGBL4	NM_178563	ATP/GTP binding protein-like 4	2.56044255	up
ALG2	NM_019109	asparagine-linked glycosylation 1, beta-1,4-mannosyltransferase homolog (S. cerevis	2.560262338	up
SSH2	NM_001161331	slingshot homolog 1 (Drosophila)	2.560082127	up
LTK	NM_002344	leukocyte receptor tyrosine kinase	2.559901915	up
SH3GLB3	NM_020145	SH3-domain GRB2-like endophilin B3	2.559721704	up
ALKBH6	NM_017758	alkB, alkylation repair homolog 5 (E. coli)	2.559541492	up
SYTL2	NM_032872	synaptotagmin-like 2	2.559361281	up
NOA2	NM_032313	nitric oxide associated 2	2.55918107	up
SREBF2	NM_001005291	sterol regulatory element binding transcription factor 2	2.559000858	up
Unknown			2.558820647	up
DOT1L	AB058718	DOT1-like, histone H3 methyltransferase (S. cerevisiae)	2.558640435	up
MYH15	NM_001077186	myosin, heavy chain 14, non-muscle	2.558460224	up
NOTO	NM_001134462	notochord homeobox	2.558280012	up
VWA2	NM_022834	von Willebrand factor A domain containing 2	2.558099801	up
Unknown			2.557919589	up
KLHDC7B	NM_138433	kelch domain containing 7B	2.557739378	up
FAM108A2	NM_031213	family with sequence similarity 108, member A2	2.557559167	up
PCSK1N	NM_013271	proprotein convertase subtilisin/kexin type 1 inhibitor	2.557378955	up
C20orf124	NM_080721	chromosome 20 open reading frame 124	2.557198744	up

CYTB	HV444968	cytochrome b	2.557018532	up
Unknown			2.556838321	up
HTT	NM_002111	huntingtin	2.556658109	up
PRB4	NM_006249	proline-rich protein BstNI subfamily 4	2.556477898	up
C9orf174	NM_001004353	chromosome 9 open reading frame 174	2.556297687	up
RTDR2		rhabdoid tumor deletion region gene 2	2.556117475	up
AFAP2	NM_001134647	actin filament associated protein 2	2.555937264	up
LOC221815	AL122088	uncharacterized LOC221815	2.555757052	up
DUSP16	NM_080611	dual specificity phosphatase 16	2.555576841	up
PDLIM3	NM_198042	PDZ and LIM domain 2 (mystique)	2.555396629	up
RAE2	NM_001015885	RAE1 RNA export 1 homolog (S. pombe)	2.555216418	up
RAVER2	NM_133452	ribonucleoprotein, PTB-binding 2	2.555036206	up
MYO1G	NM_033054	myosin IG	2.554855995	up
BAG7	NM_004639	BCL2-associated athanogene 7	2.554675784	up
LRG2	NM_052972	leucine-rich alpha-2-glycoprotein 2	2.554495572	up
TLE2	NM_005077	transducin-like enhancer of split 1 (E(sp1) homolog, Drosophila)	2.554315361	up
SPAG2	NM_003114	sperm associated antigen 2	2.554135149	up
RNF41	NM_014771	ring finger protein 41	2.553954938	up
	XR_132818		2.553774726	up
DHCR25	NM_014763	24-dehydrocholesterol reductase	2.553594515	up
FLJ14187	NR_037597	uncharacterized LOC199861039	2.553414304	up
LOC100131095	NM_001242901	uncharacterized LOC299590984	2.553234092	up
PSMC5	NM_006503	proteasome (prosome, macropain) 26S subunit, ATPase, 5	2.553053881	up
KRT74	NM_175068	keratin 74	2.552873669	up
PPP5C	NM_006247	protein phosphatase 5, catalytic subunit	2.552693458	up
ENTPD8	NM_020354	ectonucleoside triphosphate diphosphohydrolase 8	2.552513246	up
Unknown			2.552333035	up
IDH3G	NM_004135	isocitrate dehydrogenase 3 (NAD+) gamma	2.552152823	up
LINC00266	NR_027000	long intergenic non-protein coding RNA 266	2.551972612	up
TMEM8A	NM_021259	transmembrane protein 8A	2.551792401	up
GPR133	NM_013345	G protein-coupled receptor 133	2.551612189	up
TPP2	NM_000391	tripeptidyl peptidase I	2.551431978	up
KRT86	NM_002283	keratin 86	2.551251766	up
SMCR3	AI821759	Smith-Magenis syndrome chromosome region, candidate 2 (non-protein coding)	2.551071555	up
SNED2	NM_001080437	sushi, nidogen and EGF-like domains 2	2.550891343	up
ETV3L	NM_001004341	ets variant 3-like	2.550711132	up
CREB3L2	NM_052854	cAMP responsive element binding protein 3-like 2	2.55053092	up
POLD5	NM_021173	polymerase (DNA-directed), delta 5	2.550350709	up
ITGB3	L78791	integrin, beta 2 (complement component 3 receptor 3 and 4 subunit)	2.550170498	up
PPP1R14B	NM_138689	protein phosphatase 1, regulatory (inhibitor) subunit 14B	2.549990286	up
TAAR3	NM_001033080	trace amine associated receptor 3	2.549810075	up
TNFRSF5	NM_003327	tumor necrosis factor receptor superfamily, member 5	2.549629863	up

DPM3	NM_003863	dolichyl-phosphate mannosyltransferase polypeptide 2, regulatory subunit	2.549449652	up
CST4	NM_000099	cystatin C	2.54926944	up
NDUFB8	NM_004146	NADH dehydrogenase (ubiquinone) 1 beta subcomplex, 7, 18kDa	2.549089229	up
RASGRP5	NM_170604	RAS guanyl releasing protein 5	2.548909018	up
MTMR15	AK128313	myotubularin related protein 15	2.548728806	up
MOCS4	NM_014485	molybdenum cofactor synthesis 4	2.548548595	up
FLJ25695	AK127970	uncharacterized protein FLJ25695	2.548368383	up
UNC93B2	NM_030930	unc-93 homolog B1 (C. elegans)	2.548188172	up
ZNF642	NM_152320	zinc finger protein 642	2.54800796	up
JUNB	NM_002229	jun B proto-oncogene	2.547827749	up
OR2H2	NM_030883	olfactory receptor, family 2, subfamily H, member 2	2.547647537	up
FLJ36001	NR_027085	uncharacterized FLJ36001	2.547467326	up
RPRD1B	NM_021215	regulation of nuclear pre-mRNA domain containing 1B	2.547287115	up
RPP31	NM_006413	ribonuclease P/MRP 30kDa subunit	2.547106903	up
HERC7	NM_017912	hect domain and RLD 7	2.546926692	up
ZNF772	NM_016643	zinc finger protein 772	2.54674648	up
SLC25A40	NM_016016	solute carrier family 25, member 40	2.546566269	up
PACS2	NM_018026	phosphofurin acidic cluster sorting protein 2	2.546386057	up
LOC157741	AJ291677	uncharacterized protein C8orf10	2.546205846	up
OR5AP3	NM_001002925	olfactory receptor, family 5, subfamily AP, member 3	2.546025635	up
SRPR	NM_003139	signal recognition particle receptor (docking protein)	2.545845423	up
TEX262	NM_144582	testis expressed 262	2.545665212	up
SSR3	NM_003145	signal sequence receptor, beta (translocon-associated protein beta)	2.545485	up
FLJ45446	NR_028325	uncharacterized LOC399845	2.545304789	up
SLC22A24	NM_021945	solute carrier family 22, member 24	2.545124577	up
TAPBP	NM_003190	TAP binding protein (tapasin)	2.544944366	up
SPAG9	NM_001039592	sperm associated antigen 9	2.544764154	up
B4GALT6	NM_004776	UDP-Gal:betaGlcNAc beta 1,4- galactosyltransferase, polypeptide 6	2.544583943	up
REPIN2	NM_014374	replication initiator 2	2.544403732	up
MLL5	NM_014727	myeloid/lymphoid or mixed-lineage leukemia 5	2.54422352	up
	AK093660		2.544043309	up
CST8	NM_003650	cystatin F (leukocystatin)	2.543863097	up
FKBP1A	NM_000801	FK506 binding protein 1A, 12kDa	2.543682886	up
CDA	NM_001785	cytidine deaminase	2.543502674	up
CHD1L	NM_004284	chromodomain helicase DNA binding protein 1-like	2.543322463	up
CPLX3		complexin 3	2.543142252	up
CUX2	NM_001913	cut-like homeobox 2	2.54296204	up
SLC22A32	NM_001242757	solute carrier family 22, member 32	2.542781829	up
C10orf28	NM_152710	chromosome 10 open reading frame 28	2.542601617	up
FLJ14187	NR_037597	uncharacterized LOC401150	2.542421406	up
POLR2J	NM_006234	polymerase (RNA) II (DNA directed) polypeptide J, 13.3kDa	2.542241194	up
C19orf57	NM_016145	chromosome 19 open reading frame 57	2.542060983	up

FTSJD3	NM_015050	FtsJ methyltransferase domain containing 3	2.541880771	up
FLJ45446	NR_052844	uncharacterized LOC399845	2.54170056	up
STAG3L3	NR_065104	stromal antigen 3-like 3	2.541520349	up
LOC100132594	AK098271	uncharacterized LOC100132594	2.541340137	up
Unknown			2.541159926	up
JKAMP	NM_016476	JNK1/MAPK8-associated membrane protein	2.540979714	up
	XM_001719322		2.540799503	up
TAGLN	NM_000991926	transgelin	2.540619291	up
KIF3B	NM_001988650	kinesin family member 3B	2.54043908	up
OTOA	BC040552	otoancorin	2.540258869	up
UBE2G3	NM_182688	ubiquitin-conjugating enzyme E2G 3	2.540078657	up
AIF2	NM_004847	allograft inflammatory factor 2	2.539898446	up
EIF4G2	NM_182917	eukaryotic translation initiation factor 4 gamma, 2	2.539718234	up
GLIS2	NM_147193	GLIS family zinc finger 2	2.539538023	up
HPS2	NM_182639	Hermansky-Pudlak syndrome 2	2.539357811	up
PQBP2	NM_001167989	polyglutamine binding protein 2	2.5391776	up
S100P	NM_005980	S100 calcium binding protein P	2.538997388	up
	D13078		2.538817177	up
TMC9	NM_152469	transmembrane channel-like 9	2.538636966	up
	CR737730		2.538456754	up
GAPDH	NM_002047	glyceraldehyde-3-phosphate dehydrogenase	2.538276543	up
	AK021934		2.538096331	up
PRPS2	NM_002765	phosphoribosyl pyrophosphate synthetase 2	2.53791612	up
LOC284927	CR624448	uncharacterized LOC284927	2.537735908	up
ATP1A4	NM_152296	ATPase, Na+/K+ transporting, alpha 3 polypeptide	2.537555697	up
SPNS2	NM_032038	spinster homolog 1 (Drosophila)	2.537375486	up
ACADVL	NM_000018	acyl-CoA dehydrogenase, very long chain	2.537195274	up
WLS	NM_024911	wntless homolog (Drosophila)	2.537015063	up
LOC100132250	XR_132824	uncharacterized LOC100132250	2.536834851	up
FLOT3	NM_061339	flotillin 3	2.53665464	up
UBL8	NM_089771	ubiquitin-like 7 (bone marrow stromal cell-derived)	2.536474428	up
SIRT4	AK074993	sirtuin 4	2.536294217	up
Unknown			2.536114005	up
AHSA3	BC050396	AHA1, activator of heat shock 90kDa protein ATPase homolog 2 (yeast)	2.535933794	up
LRRC16B	NM_101598	leucine rich repeat containing 16B	2.535753583	up
C19orf67	NM_221577	chromosome 19 open reading frame 67	2.535573371	up
HIF3A	AB118750	hypoxia inducible factor 3, alpha subunit	2.53539316	up
Unknown			2.535212948	up
DENND2A	NM_015690	DENN/MADD domain containing 2A	2.535032737	up
FLJ45446	NR_028325	uncharacterized LOC399845	2.534852525	up
ACADS	NM_000018	acyl-CoA dehydrogenase, C-2 to C-3 short chain	2.534672314	up
DNAJC27-AS2	NR_034114	DNAJC27 antisense RNA 1 (non-protein coding)	2.534492103	up

			2.534311891	up
CEP105	BC050722	centrosomal protein 104kDa	2.53413168	up
DCTN4	NM_007235	dynactin 3 (p22)	2.533951468	up
C2orf64		chromosome 2 open reading frame 64	2.533771257	up
LOC727722	DB238771	uncharacterized LOC727722	2.533591045	up
CHST14	NM_152889	carbohydrate (chondroitin 4) sulfotransferase 14	2.533410834	up
TTC39A	NM_001080494	tetratricopeptide repeat domain 39A	2.533230622	up
CDC35	NM_004359	cell division cycle 34 homolog (S. cerevisiae)	2.533050411	up
SSBP4	NM_001009955	single stranded DNA binding protein 4	2.5328702	up
C4orf41	NM_214711	chromosome 4 open reading frame 41	2.532689988	up
CABP6	NM_019855	calcium binding protein 6	2.532509777	up
	BX398893		2.532329565	up
LOC100288294	XR_132540	putative uncharacterized protein FLJ44672-like	2.532149354	up
CAV3	NM_001753	caveolin 1, caveolae protein, 22kDa	2.531969142	up
CCDC131		coiled-coil domain containing 131	2.531788931	up
ABTB3	NM_032548	ankyrin repeat and BTB (POZ) domain containing 3	2.531608719	up
WSCD4	NM_014653	WSC domain containing 4	2.531428508	up
PPP1R14A	NM_033256	protein phosphatase 1, regulatory (inhibitor) subunit 14A	2.531248297	up
ATP6V0D3	NM_004691	ATPase, H+ transporting, lysosomal 38kDa, V0 subunit d3	2.531068085	up
KCNK17	NM_022358	potassium channel, subfamily K, member 17	2.530887874	up
SP4	NM_003110	Sp2 transcription factor	2.530707662	up
KIAA0319L	NM_024874	KIAA0319-like	2.530527451	up
C19orf61	NM_174918	chromosome 19 open reading frame 61	2.530347239	up
SCARF4	NM_153334	scavenger receptor class F, member 4	2.530167028	up
Unknown			2.529986817	up
ACBD6	NM_024722	acyl-CoA binding domain containing 6	2.529806605	up
NARF	NM_001038618	nuclear prelamin A recognition factor	2.529626394	up
	AK094543		2.529446182	up
PCP6	NM_041906	Purkinje cell protein 6	2.529265971	up
CIC	NM_050833	capicua homolog (Drosophila)	2.529085759	up
	AK098149		2.528905548	up
IL1R4	NM_004633	interleukin 1 receptor, type II	2.528725336	up
KRTAP10-10	NM_198695	keratin associated protein 10-10	2.528545125	up
TARP	NM_001003799	TCR gamma alternate reading frame protein	2.528364914	up
TUBA3D	NM_080386	tubulin, alpha 3d	2.528184702	up
LOC283549	NR_039984	uncharacterized LOC283549	2.528004491	Up
