# Supplementary material for: Transcriptional signature associated with early rheumatoid arthritis and healthy individuals at high risk to develop the disease
Source: PLoS One. 2018 Mar 27;13(3):e0194205. doi: 10.1371/journal.pone.0194205 (PMC5870959; doi:10.1371/journal.pone.0194205)
Supplement: S5 Table — (PDF) [file pone.0194205.s005.pdf]

Supplementary table 5. Down regulated genes in relatives with ACCP+ vs relatives with ACCP-

Gene Symbol	Genbank Accession	Gene Name	Fold Change	Regulation
	AK127601		-8.149225	down
TNR	NM_003285	tenascin R (restrictin, janusin)	-6.9211035	down
ZNF326	NM_182975///NM_182976	zinc finger protein 326	-6.9098153	down
	BC040619		-6.651993	down
KRTAP4-1	NM_033060	keratin associated protein 4-1	-6.053292	down
CCDC64B	NM_001103175	coiled-coil domain containing 64B	-5.9813466	down
AADACL3	NM_001103170	arylacetamide deacetylase-like 3	-5.93062	down
LOC143286	AL049428	uncharacterized LOC143286	-5.6371875	down
C17orf99	NM_001163075	chromosome 17 open reading frame 99	-5.471807	down
TMEM174	NM_153217	transmembrane protein 174	-5.4278245	down
LOC146795	AK057377	uncharacterized LOC146795	-5.4235826	down
GABRE	NM_004961	gamma-aminobutyric acid (GABA) A receptor, epsilon	-5.390086	down
MAGEC1	NM_005462	melanoma antigen family C, 1	-5.3627763	down
ACBD7	NM_001039844	acyl-CoA binding domain containing 7	-5.3035192	down
IGLON5	NM_001101372	IgLON family member 5	-5.2727575	down
COL4A1	NM_001845	collagen, type IV, alpha 1	-5.2538695	down
LOC284014	AK095567	uncharacterized LOC284014	-5.1718607	down
SPEG	NM_005876	SPEG complex locus	-5.171494	down
C7	NM_000587	complement component 7	-5.003119	down
	BE244176		-4.975176	down
LOC100130442	AK131364	uncharacterized LOC100130442	-4.9635677	down
TERT	NM_198253	telomerase reverse transcriptase	-4.9621606	down
LOC100128811	NR_027333	uncharacterized LOC100128811	-4.886698	down
DPYSL3	NM_001387	dihydropyrimidinase-like 3	-4.842905	down
RASGEF1C	NM_175062	RasGEF domain family, member 1C	-4.7974463	down
LOC729668	NR_003524	golgi autoantigen, golgin subfamily a, 6 pseudogene	-4.762927	down
RABL2B	BC020495	RAB, member of RAS oncogene family-like 2B	-4.7587266	down
LOC100133131	AF289593	uncharacterized LOC100133131	-4.7572374	down
KALRN	AK131379///AK125979	kalirin, RhoGEF kinase	-4.7004085	down
C15orf59	NM_001039614	chromosome 15 open reading frame 59	-4.6669188	down
LOC100287314	NR_040245	uncharacterized LOC100287314	-4.633044	down
ANKRD20A5P	BC022023	ankyrin repeat domain 20 family, member A5, pseudogene	-4.535587	down
	BC039333		-4.522702	down
	AK026667		-4.511659	down
	BM930849		-4.493437	down
	AF289570		-4.4800324	down
LOC283335	NR_033854	uncharacterized LOC283335	-4.4514327	down
OPN1MW	NM_000513	opsin 1 (cone pigments), medium-wave-sensitive	-4.419268	down
KCNT1	AK123276	potassium channel, subfamily T, member 1	-4.409153	down
LOC100130713	AK096566	uncharacterized LOC100130713	-4.406819	down
XRCC2	NM_005431	X-ray repair complementing defective repair in Chinese hamster cells 2	-4.3245616	down
LOC100129048	AK128074	uncharacterized LOC100129048	-4.314815	down
LOC731932	XM_001716667	uncharacterized LOC731932	-4.299716	down
PPIAL4G	AK123006	peptidylprolyl isomerase A (cyclophilin A)-like 4G	-4.292633	down

KRTAP10-5	NM_198694	keratin associated protein 10-5	-4.2823825	down
MSTO2P	NR_024117	misato homolog 2 pseudogene	-4.2730513	down
HOGA1	AK094791	4-hydroxy-2-oxoglutarate aldolase 1	-4.2575474	down
RASSF6	NM_201431	Ras association (RalGDS/AF-6) domain family member 6	-4.255986	down
SCNN1D	NM_001130413	sodium channel, nonvoltage-gated 1, delta	-4.190655	down
SPRR2E	NM_001024209	small proline-rich protein 2E	-4.182568	down
HTR6	NM_000871	5-hydroxytryptamine (serotonin) receptor 6	-4.152948	down
LOC284454	NR_036515	uncharacterized LOC284454	-4.148765	down
FAT3	NM_001008781	FAT tumor suppressor homolog 3 (Drosophila)	-4.1319385	down
ANP32A-IT1	NR_026808	ANP32A intronic transcript 1 (non-protein coding)	-4.1204963	down
FAM151A	NM_176782	family with sequence similarity 151, member A	-4.105673	down
BMP8B	NM_001720	bone morphogenetic protein 8b	-4.078451	down
LOC145820	NR_027132	uncharacterized LOC145820	-4.0527415	down
SRGAP1	NM_020762	SLIT-ROBO Rho GTPase activating protein 1	-4.021942	down
GOLGA1		golgin A1	-3.9849272	down
ZNF713	NM_182633	zinc finger protein 713	-3.9646027	down
CD70	NM_001252	CD70 molecule	-3.9595456	down
C20orf85	NM_178456	chromosome 20 open reading frame 85	-3.9237356	down
	BC035135		-3.9192657	down
LOC257152	AK001439	uncharacterized LOC257152	-3.8936198	down
TMEM211	NM_001001663	transmembrane protein 211	-3.8791683	down
LINC00162	NR_024089	long intergenic non-protein coding RNA 162	-3.876327	down
RIIAD1	NM_001144956	regulatory subunit of type II PKA R-subunit (RIIa) domain containing 1	-3.8571253	down
DEFB132	NM_207469	defensin, beta 132	-3.8566248	down
C3orf51	NR_024615	chromosome 3 open reading frame 51	-3.8433135	down
	AK126743		-3.83514	down
	AK124041		-3.813653	down
LOC728208	XR_108453	uncharacterized LOC728208	-3.8022668	down
KLK3	NM_001030050	kallikrein-related peptidase 3	-3.7934535	down
SLC22A7	NM_153320	solute carrier family 22 (organic anion transporter), member 7	-3.7900012	down
DKFZp451A211	XM_003403663	DKFZp451A211 protein	-3.7832575	down
ZNF2	NM_021088	zinc finger protein 2	-3.7799203	down
LOC541472	XR_108749	uncharacterized LOC541472	-3.7415555	down
MRP63	NM_024026	mitochondrial ribosomal protein 63	-3.7029822	down
EDA	NM_001005610	ectodysplasin A	-3.6996717	down
ABCD3	NM_001122674	ATP-binding cassette, sub-family D (ALD), member 3	-3.6944852	down
	AK097143		-3.682143	down
	XR_133518		-3.6721823	down
BAALC		brain and acute leukemia, cytoplasmic	-3.6676579	down
LOC284379	NR_002938	solute carrier family 7 (cationic amino acid transporter, y+ system), member 3 pseudogene	-3.6466064	down
	AF068294		-3.6458466	down
RHCG	NM_016321	Rh family, C glycoprotein	-3.6436305	down
MGC16121	NR_024607	uncharacterized protein MGC16121	-3.6432042	down
CERCAM	NM_016174	cerebral endothelial cell adhesion molecule	-3.6397061	down
LOC201477	NR_038895	uncharacterized LOC201477	-3.6162875	down

C2orf66	NM_213608	chromosome 2 open reading frame 66	-3.6038916	down
NEAT1	AF001893	nuclear paraspeckle assembly transcript 1 (non-protein coding)	-3.6036763	down
KRT74	NM_175053	keratin 74	-3.5976164	down
PRSS47	XR_108978	protease, serine, 47	-3.5894709	down
MGC4859	BC002644	uncharacterized LOC79150	-3.588737	down
ST8SIA1	NM_003034	ST8 alpha-N-acetyl-neuraminide alpha-2,8-sialyltransferase 1	-3.5464482	down
C9orf153	NM_001010907	chromosome 9 open reading frame 153	-3.539701	down
MRPS16	NM_016065	mitochondrial ribosomal protein S16	-3.5393963	down
SH3GL3	NM_003027	SH3-domain GRB2-like 3	-3.5385592	down
TRIP13	NM_004237	thyroid hormone receptor interactor 13	-3.5316992	down
LOC644649	BX108667	apolipoprotein O pseudogene	-3.5239089	down
GSG1	NM_001206842///NM_001080554	germ cell associated 1	-3.511323	down
MYOZ3	NM_133371	myozenin 3	-3.510613	down
LOC100127904	BC065739	uncharacterized LOC100127904	-3.5052238	down
LOC442366	XM_001714957	uncharacterized LOC442366	-3.4912412	down
LOC100652869	XM_003403453	uncharacterized LOC100652869	-3.4865127	down
ASIP	NM_001672	agouti signaling protein	-3.4822333	down
	AF390550		-3.4373658	down
LOC100130876	XR_109361	uncharacterized LOC100130876	-3.435041	down
COL5A1	AK057231	collagen, type V, alpha 1	-3.426531	down
CRYBB2P1	NR_033733	crystallin, beta B2 pseudogene 1	-3.4127192	down
POLR2J2	NM_032959	polymerase (RNA) II (DNA directed) polypeptide J2	-3.4098988	down
ZNF667	NM_022103	zinc finger protein 667	-3.4020307	down
LANCL3	NM_198511	LanC lantibiotic synthetase component C-like 3 (bacterial)	-3.3886886	down
LOC340073	NR_037895	uncharacterized LOC340073	-3.3809247	down
SCARA5	AY337579	scavenger receptor class A, member 5 (putative)	-3.3774571	down
LOC286154	AK096739	uncharacterized LOC286154	-3.3768787	down
INADL	AJ001306	InaD-like (Drosophila)	-3.3725493	down
	AK025047		-3.3656507	down
CCBL2	NM_001008661	cysteine conjugate-beta lyase 2	-3.3546028	down
CTPS2	NM_001144002	CTP synthase II	-3.3519897	down
LOC100128366	XR_132755	uncharacterized LOC100128366	-3.3333921	down
F8	NM_000132	coagulation factor VIII, procoagulant component	-3.3271666	down
SGSM2	NM_014853///AK124883	small G protein signaling modulator 2	-3.3230295	down
LOC729609	NR_024440	uncharacterized LOC729609	-3.3229191	down
PHLDA3	NM_012396	pleckstrin homology-like domain, family A, member 3	-3.3228786	down
	BC007984		-3.3201811	down
	AK130932		-3.317462	down
	XM_003403554		-3.3143845	down
TTTY14	NR_001543	testis-specific transcript, Y-linked 14 (non-protein coding)	-3.3106325	down
LDHAL6A	NM_144972	lactate dehydrogenase A-like 6A	-3.3089619	down
DRD3	NM_033663	dopamine receptor D3	-3.303985	down
CD276	NM_001024736	CD276 molecule	-3.3010466	down
GOLGA6L9	NM_198181	golgin A6 family-like 9	-3.2969959	down
MDH1B	AK309144	malate dehydrogenase 1B, NAD (soluble)	-3.292479	down
CLDN24	NM_001185149	claudin 24	-3.2797403	down

LOC100130015	NR_027335	5-hydroxyisourate hydrolase pseudogene	-3.2691438	down
FLJ42392	NR_033877	uncharacterized LOC400123	-3.263585	down
FAM180B	NM_001164379	family with sequence similarity 180, member B	-3.2609055	down
	AK309505		-3.242605	down
FLJ42022	AK124016	uncharacterized LOC646748	-3.2342017	down
BPIFA1	NM_130852	BPI fold containing family A, member 1	-3.233667	down
LOC100134868	NR_004846	uncharacterized LOC100134868	-3.2231336	down
IGSF21	NM_032880	immunoglobin superfamily, member 21	-3.220779	down
LINC00467	NR_026761	long intergenic non-protein coding RNA 467	-3.2171037	down
METTL21A	NM_145280///NM_001127395	methyltransferase like 21A	-3.2161102	down
VPS37D	NM_001077621	vacuolar protein sorting 37 homolog D (S. cerevisiae)	-3.208353	down
TTC28	NM_001145418	tetratricopeptide repeat domain 28	-3.207271	down
LOC100509196	XM_003119833	putative uncharacterized protein encoded by NCRNA00205-like	-3.2044818	down
LOC100130175	AK125829	uncharacterized LOC100130175	-3.199861	down
LOC100233209	NR_026544	uncharacterized LOC100233209	-3.1969006	down
UMODL1	AB096971	uromodulin-like 1	-3.1875017	down
	AY927536		-3.1818442	down
TNC	NM_002160	tenascin C	-3.1592815	down
CTNS		cystinosin, lysosomal cystine transporter	-3.1545734	down
ANO2	NM_020373	anoctamin 2	-3.1503232	down
TGM7	NM_052955	transglutaminase 7	-3.1474361	down
KLK10	NM_002776	kallikrein-related peptidase 10	-3.1438987	down
TOX2	NM_032883	TOX high mobility group box family member 2	-3.1425915	down
AMZ1	NM_133463	archaelysin family metallopeptidase 1	-3.1391256	down
LOC100130453	AK123920	uncharacterized LOC100130453	-3.137412	down
RAP1GAP	NM_002885	RAP1 GTPase activating protein	-3.1310093	down
TCEAL2	NM_080390	transcription elongation factor A (SII)-like 2	-3.1228952	down
ADAD2	NM_139174	adenosine deaminase domain containing 2	-3.1180933	down
	AK130724		-3.1152065	down
NPY	NM_000905	neuropeptide Y	-3.1149602	down
C9orf96	NM_153710	chromosome 9 open reading frame 96	-3.114468	down
LOC649294	AK091259	uncharacterized LOC649294	-3.1127348	down
LOC100128851	AK127423	uncharacterized LOC100128851	-3.1016989	down
HIP1	AY358103	huntingtin interacting protein 1	-3.0990822	down
KRBA2	NM_213597	KRAB-A domain containing 2	-3.0931094	down
LOC440910	NR_030728	uncharacterized LOC440910	-3.0913005	down
GH1	NM_000515	growth hormone 1	-3.0904567	down
SCN5A	NM_001160161///NM_001099404	sodium channel, voltage-gated, type V, alpha subunit	-3.0878847	down
OR10H1	NM_013940	olfactory receptor, family 10, subfamily H, member 1	-3.0812497	down
LOC151171	NR_037809	uncharacterized LOC151171	-3.0728755	down
SNAR-A3	NR_024214	small ILF3/NF90-associated RNA A3	-3.0648012	down
KLK6	NM_001012964	kallikrein-related peptidase 6	-3.0550961	down
	AK130019		-3.050063	down

	AK128820		-3.048331	down
SMARCA1	NM_003069	SWI/SNF related, matrix associated, actin dependent regulator of chromatin, subfamily a, member 1	-3.045075	down
RPL23AP64	NR_003040	ribosomal protein L23a pseudogene 64	-3.045053	down
LOC440461	NR_027283	Rho GTPase activating protein 27 pseudogene	-3.0416405	down
TAF5L		TAF5-like RNA polymerase II, p300/CBP-associated factor (PCAF)-associated factor, 65kDa	-3.0314069	down
	AK128523		-3.0263355	down
C21orf88	NR_026543///NR_026542	chromosome 21 open reading frame 88	-3.0261002	down
	XR_112969		-3.0252192	down
KCNQ1OT1	NR_002728	KCNQ1 opposite strand/antisense transcript 1 (non-protein coding)	-3.0212224	down
	AK094078		-3.0137305	down
CNTN6	NM_014461	contactin 6	-3.0039775	down
TPRX1	NM_198479	tetra-peptide repeat homeobox 1	-2.9983547	down
LOC100130987	NR_024469	uncharacterized LOC100130987	-2.9896526	down
C14orf167	NR_023921	chromosome 14 open reading frame 167	-2.9855416	down
	CD674797		-2.9718642	down
LOC100128644	XR_110032	LMNE6487	-2.963439	down
ANKRD36B	NM_025190	ankyrin repeat domain 36B	-2.963027	down
KRTAP12-2	NM_181684	keratin associated protein 12-2	-2.9618137	down
LOC644093	XR_132708	hCG2040054	-2.9559996	down
MAP6	NM_207577	microtubule-associated protein 6	-2.954796	down
LOC652586	XM_001719844	uncharacterized LOC652586	-2.9513712	down
LOC400752	NR_024270	uncharacterized LOC400752	-2.9489892	down
AMMECR1	NM_001171689	Alport syndrome, mental retardation, midface hypoplasia and elliptocytosis chromosomal region gene 1	-2.9474378	down
HYDIN2	XR_133104	hydrocephalus inducing homolog 2 (mouse)	-2.9408126	down
BOD1P	BM559531	biorientation of chromosomes in cell division 1 pseudogene	-2.9368014	down
CCDC149	NM_173463///AL834257	coiled-coil domain containing 149	-2.9340742	down
ADCYAP1R1	NM_001118	adenylate cyclase activating polypeptide 1 (pituitary) receptor type I	-2.9194582	down
GPC2	NM_152742	glypican 2	-2.9160316	down
psiTPTE22	NR_001591	TPTE pseudogene	-2.8998117	down
PCDHGC5	NM_032407	protocadherin gamma subfamily C, 5	-2.8901749	down
SPPL2B	NM_152988	signal peptide peptidase-like 2B	-2.889194	down
BEX1	NM_018476	brain expressed, X-linked 1	-2.880442	down
DLK2	NM_206539	delta-like 2 homolog (Drosophila)	-2.8770752	down
LOC100128818	XR_109738	LPEQ6126	-2.8560507	down
FLJ13773	AK023835	FLJ13773	-2.8553648	down
H2AFB2	NM_001017991	H2A histone family, member B2	-2.8528192	down
KRT35	NM_002280	keratin 35	-2.8473141	down
C10orf91	NM_173541	chromosome 10 open reading frame 91	-2.8469021	down
RP11-165H20.1	NR_003928	CHIA-like pseudogene	-2.8442988	down
CYP1A2	NM_000761	cytochrome P450, family 1, subfamily A, polypeptide 2	-2.843859	down
LPHN1	NM_001008701	latrophilin 1	-2.8404775	down

RAPGEF3	U78169	Rap guanine nucleotide exchange factor (GEF) 3	-2.8392982	down
	AJ295982		-2.8383412	down
KIAA0125	NR_026800	KIAA0125	-2.8363616	down
FRMD4A	NM_018027	FERM domain containing 4A	-2.824559	down
	XM_003118553		-2.8186138	down
GNAL	NM_002071	guanine nucleotide binding protein (G protein), alpha activating activity polypeptide, olfactory type	-2.8121302	down
IFNA4	NM_021068	interferon, alpha 4	-2.8103843	down
EFCAB10	BC062748	EF-hand calcium binding domain 10	-2.810281	down
	AK127505		-2.806459	down
	DB056001		-2.8032458	down
KRTAP21-1	NM_181619	keratin associated protein 21-1	-2.8026798	down
KSR2	NM_173598	kinase suppressor of ras 2	-2.8023064	down
LAMC1	NM_002293	laminin, gamma 1 (formerly LAMB2)	-2.7998624	down
FFAR1	NM_005303	free fatty acid receptor 1	-2.7981694	down
RORB	NM_006914	RAR-related orphan receptor B	-2.7963376	down
C14orf128	NR_027263	chromosome 14 open reading frame 128	-2.7945726	down
TCF23	NM_175769	transcription factor 23	-2.7914324	down
LOC729032	XR_132715	ribosomal protein L36 pseudogene	-2.788074	down
PSORS1C1	NM_014068	psoriasis susceptibility 1 candidate 1	-2.785542	down
LY6G6C	NM_025261	lymphocyte antigen 6 complex, locus G6C	-2.781667	down
KRTAP1-5	NM_031957	keratin associated protein 1-5	-2.7801375	down
SLC25A16	BC001407///NM_152707	solute carrier family 25 (mitochondrial carrier; Graves disease autoantigen), member 16	-2.7757733	down
DEFB121	NM_001011878	defensin, beta 121	-2.7725685	down
GABRB1	AK296023	gamma-aminobutyric acid (GABA) A receptor, beta 1	-2.7698011	down
CHKA	NM_001277	choline kinase alpha	-2.7688966	down
CRYGN	NM_144727	crystallin, gamma N	-2.7666614	down
ERVFRD-2	AK127846	endogenous retrovirus group FRD, member 2	-2.763434	down
FLJ31662	NR_033966	uncharacterized LOC440594	-2.7625144	down
DCLK2	NM_001040261	doublecortin-like kinase 2	-2.7607162	down
RASGRF2	NM_006909	Ras protein-specific guanine nucleotide-releasing factor 2	-2.7606442	down
HERC2P7	AF071178///XR_133065	hect domain and RLD 2 pseudogene 7	-2.7562346	down
APOC4	NM_001646	apolipoprotein C-IV	-2.753569	down
LOC442421	NR_024496	uncharacterized LOC442421	-2.753157	down
CEP72	NM_018140	centrosomal protein 72kDa	-2.7498465	down
NRSN1		neurensin 1	-2.7442598	down
IRX2	NM_033267	iroquois homeobox 2	-2.7434125	down
KLK5	NM_012427	kallikrein-related peptidase 5	-2.7384646	down
CBWD7	AK097639	COBW domain containing 7	-2.7370837	down
SLFN5	NM_144975	schlafen family member 5	-2.7370715	down
FLJ10661	NR_024362///NR_024361	family with sequence similarity 86, member A pseudogene	-2.728295	down
KGFLP2	NR_003670	keratinocyte growth factor-like protein 2	-2.7273145	down
PALM3	NM_001145028	paralemmin 3	-2.724795	down
ODF2L	NM_001007022	outer dense fiber of sperm tails 2-like	-2.72029	down
FLJ46906	NR_033896	uncharacterized LOC441172	-2.719882	down
HDGFRP2	NM_032631///AB208892	hepatoma-derived growth factor-related protein 2	-2.7197547	down
OR10J5	NM_001004469	olfactory receptor, family 10, subfamily J, member 5	-2.7189047	down

MUC6	NM_005961	mucin 6, oligomeric mucus/gel-forming	-2.7174659	down
	CU687439		-2.716379	down
MRGPRE	NM_001039165	MAS-related GPR, member E	-2.7119572	down
GRIA4	NM_001077243	glutamate receptor, ionotrophic, AMPA 4	-2.7108667	down
LZTS2	AK097997///NM_032429	leucine zipper, putative tumor suppressor 2	-2.710476	down
FAM155B	NM_015686	family with sequence similarity 155, member B	-2.709032	down
GPR144	NM_001161808	G protein-coupled receptor 144	-2.7072523	down
DEFB108B	NM_001002035	defensin, beta 108B	-2.7050786	down
C4orf39	NM_153027	chromosome 4 open reading frame 39	-2.704934	down
SLC16A13	NM_201566	solute carrier family 16, member 13 (monocarboxylic acid transporter 13)	-2.7010362	down
FAM27A	NR_024060	family with sequence similarity 27, member A	-2.6990807	down
CENPF	NM_016343	centromere protein F, 350/400kDa (mitosin)	-2.697138	down
LIPM	NM_001128215	lipase, family member M	-2.695125	down
HAUS5	NM_015302	HAUS augmin-like complex, subunit 5	-2.6927483	down
MGC10814	BC004943	uncharacterized protein MGC10814	-2.687813	down
	XR_108874		-2.684743	down
LOC284373	BX537909	uncharacterized LOC284373	-2.682722	down
FCN3	AK309540///NM_003665	ficolin (collagen/fibrinogen domain containing) 3 (Hakata antigen)	-2.6824653	down
GDF7	NM_182828	growth differentiation factor 7	-2.67323	down
MRGPRX4	NM_054032	MAS-related GPR, member X4	-2.6724622	down
	XR_109126		-2.672053	down
SUZ12P	BC047718	suppressor of zeste 12 homolog pseudogene	-2.6694243	down
LOC400043	NR_026656	uncharacterized LOC400043	-2.6686485	down
DNA2	NM_001080449	DNA replication helicase 2 homolog (yeast)	-2.6659276	down
O3FAR1	NM_181745	omega-3 fatty acid receptor 1	-2.664836	down
HMP19	NM_015980	HMP19 protein	-2.6647341	down
	AK093443		-2.6609523	down
CDC7	NM_003503	cell division cycle 7 homolog (S. cerevisiae)	-2.6598022	down
RUFY4	NM_198483	RUN and FYVE domain containing 4	-2.6572275	down
LOC100130236	AK128227	uncharacterized LOC100130236	-2.6548076	down
CDC6	NM_001254	cell division cycle 6 homolog (S. cerevisiae)	-2.6420462	down
TEKT2	NM_014466	tektin 2 (testicular)	-2.641182	down
CAPN5	NM_004055	calpain 5	-2.6409423	down
	AK125780		-2.637878	down
ZNF763	NM_001012753	zinc finger protein 763	-2.6329584	down
GLYCTK	NM_001144951	glycerate kinase	-2.6321416	down
GCG	NM_002054	glucagon	-2.631446	down
MAPK8	NM_139047	mitogen-activated protein kinase 8	-2.6304858	down
KRTAP10-2	NM_198693	keratin associated protein 10-2	-2.6304023	down
LACTBL1	XM_002342035	lactamase, beta-like 1	-2.6256578	down
TAAR8	NM_053278	trace amine associated receptor 8	-2.621925	down
ZNF562	NM_017656	zinc finger protein 562	-2.6172392	down
LOC100130768	AK128224	uncharacterized LOC100130768	-2.616366	down
MFSD2B	NM_001080473	major facilitator superfamily domain containing 2B	-2.6144173	down
VCX2	NM_016378	variable charge, X-linked 2	-2.6070209	down

NOL3	NM_003946	nucleolar protein 3 (apoptosis repressor with CARD domain)	-2.603689	down
GRIK5	NM_002088	glutamate receptor, ionotropic, kainate 5	-2.6029408	down
RXFP3	NM_016568	relaxin/insulin-like family peptide receptor 3	-2.6019537	down
SMTN	NM_134269	smoothelin	-2.598266	down
ZNF527	BC014325	zinc finger protein 527	-2.5981786	down
SH3GL1P3	X99663	SH3-domain GRB2-like 1 pseudogene 3	-2.5894454	down
LIN28B	NM_001004317	lin-28 homolog B (C. elegans)	-2.5886908	down
	XM_001129515		-2.5876904	down
ARL17A	NM_016632	ADP-ribosylation factor-like 17A	-2.5871973	down
VSIG7	XM_003403542	V-set and immunoglobulin domain containing 7	-2.5851786	down
GPR62	NM_080865	G protein-coupled receptor 62	-2.5824215	down
LOC283693	NR_036446	actin, gamma pseudogene	-2.582228	down
C6orf208	NR_026780	chromosome 6 open reading frame 208	-2.5815573	down
GCLM	NM_002061	glutamate-cysteine ligase, modifier subunit	-2.5814161	down
IGF2-AS	NR_028044	insulin-like growth factor 2 antisense (non-protein coding)	-2.5720792	down
LOC100130285	XR_109235	uncharacterized LOC100130285	-2.5712218	down
ACTL8	NM_030812	actin-like 8	-2.5686913	down
BEST4	NM_153274	bestrophin 4	-2.5667307	down
COL4A6	BT007228	collagen, type IV, alpha 6	-2.565813	down
LOC100144604	NR_021493	uncharacterized LOC100144604	-2.5642583	down
OR10C1	NM_013941	olfactory receptor, family 10, subfamily C, member 1	-2.558106	down
GPHA2	NM_130769	glycoprotein hormone alpha 2	-2.553818	down
MST1R	NM_002447	macrophage stimulating 1 receptor (c-met-related tyrosine kinase)	-2.5493414	down
	AK093659		-2.5483825	down
LOC200261	NR_034149	uncharacterized LOC200261	-2.5465016	down
BCMO1	NM_017429	beta-carotene 15,15'-monooxygenase 1	-2.544161	down
DPF1	NM_001135155	D4, zinc and double PHD fingers family 1	-2.543376	down
CACNA2D1	NM_000722	calcium channel, voltage-dependent, alpha 2/delta subunit 1	-2.541569	down
LOC440896	NR_015361	uncharacterized LOC440896	-2.540988	down
AUTS2	NM_015570///NM_001127232	autism susceptibility candidate 2	-2.5362935	down
GOLGA8IP	NR_024074	golgin A8 family, member I, pseudogene	-2.536184	down
KRTAP5-5	NM_001001480	keratin associated protein 5-5	-2.5331464	down
	AK056269		-2.532334	down
GLI2	NM_005270	GLI family zinc finger 2	-2.5322402	down
D21S2088E	NR_040254	D21S2088E	-2.5296218	down
LOC100131015	AK124509	uncharacterized LOC100131015	-2.527658	down
LOC100507205	NR_038309	uncharacterized LOC100507205	-2.5262938	down
LOC400499	XM_003118689	uncharacterized LOC400499	-2.5256298	down
ST8SIA3	NM_015879	ST8 alpha-N-acetyl-neuraminide alpha-2,8-sialyltransferase 3	-2.522339	down
CTSE	NM_001910	cathepsin E	-2.521859	down
SPRR2F	NM_001014450	small proline-rich protein 2F	-2.5202756	down
CELF6	NM_001172684	CUGBP, Elav-like family member 6	-2.5196774	down

CYP2F1	NM_000774	cytochrome P450, family 2, subfamily F, polypeptide 1	-2.5166054	down
LOC170425	NR_038220	uncharacterized LOC170425	-2.5158978	down
RGS11	NM_003834	regulator of G-protein signaling 11	-2.5146346	down
LOC494150	BC014228	prohibitin pseudogene	-2.5127451	down
ZNF683	NM_001114759	zinc finger protein 683	-2.5126276	down
CEACAM8	NM_001816	carcinoembryonic antigen-related cell adhesion molecule 8	-2.5116963	down
KIAA1919	NM_153369	KIAA1919	-2.5078835	down
LOC100130913	AK128355	uncharacterized LOC100130913	-2.5026677	down
OPRL1	NM_182647	opiate receptor-like 1	-2.4988651	down
LOC644285	AK126853	uncharacterized LOC644285	-2.4981222	down
KCTD11	NM_001002914	potassium channel tetramerisation domain containing 11	-2.4954853	down
FAM171A2	NM_198475	family with sequence similarity 171, member A2	-2.4954185	down
TCTE1	NM_182539	t-complex-associated-testis-expressed 1	-2.494768	down
LOC100130078	AK094659	uncharacterized LOC100130078	-2.4942832	down
C4BPA	NM_000715	complement component 4 binding protein, alpha	-2.4921403	down
AQP8	NM_001169	aquaporin 8	-2.48934	down
BHLHA15	NM_177455	basic helix-loop-helix family, member a15	-2.4891992	down
LOC100130539	XM_001724322	uncharacterized LOC100130539	-2.4865284	down
KRT18	NM_000224	keratin 18	-2.4853084	down
	AK124642		-2.4835212	down
SIX2	NM_016932	SIX homeobox 2	-2.4783657	down
	AK056856		-2.4768996	down
FHL2	NM_001039492///CR936682	four and a half LIM domains 2	-2.4763184	down
CDCA7	NM_031942	cell division cycle associated 7	-2.4757226	down
LHFPL4	NM_198560	lipoma HMGIC fusion partner-like 4	-2.4756093	down
SULT1C2P1	NR_037191	sulfotransferase family, cytosolic, 1C, member 2 pseudogene 1	-2.4747286	down
EFCAB5	NM_001145053	EF-hand calcium binding domain 5	-2.4736834	down
MACROD2	BC035876///NM_080676	MACRO domain containing 2	-2.4698927	down
TSL	AB050003	testis-expressed, seven-twelve, leukemia	-2.466147	down
FAM160A1	NM_001109977	family with sequence similarity 160, member A1	-2.465684	down
	XR_110878		-2.4619625	down
SPRR3	NM_005416	small proline-rich protein 3	-2.4597776	down
C9orf24	NM_032596	chromosome 9 open reading frame 24	-2.4568412	down
TNRC6C	NM_001142640	trinucleotide repeat containing 6C	-2.4550312	down
	AL832737		-2.454463	down
TRPM3	NM_001007471	transient receptor potential cation channel, subfamily M, member 3	-2.4535666	down
CSH1	NM_001317	chorionic somatomammotropin hormone 1 (placental lactogen)	-2.4519737	down
CYP2B6	NM_000767	cytochrome P450, family 2, subfamily B, polypeptide 6	-2.4499354	down
LOC393078	XR_108716	uncharacterized LOC393078	-2.4493845	down
ANKRD53	NM_024933	ankyrin repeat domain 53	-2.4476876	down
C12orf34	NM_032829	chromosome 12 open reading frame 34	-2.4452784	down
PPP6R1	NM_014931	protein phosphatase 6, regulatory subunit 1	-2.4439967	down
LCN8	BC042109	lipocalin 8	-2.4424543	down

LOC153811	AK021734	uncharacterized LOC153811	-2.4395025	down
PLAC9		placenta-specific 9	-2.4391804	down
C5orf49	NM_001089584	chromosome 5 open reading frame 49	-2.4347346	down
GEMIN2	NM_003616	gem (nuclear organelle) associated protein 2	-2.427462	down
	AK025312		-2.4272277	down
	BC043411		-2.426614	down
RAB40AL	NM_001031834	RAB40A, member RAS oncogene family-like	-2.425677	down
FAM187B	NM_152481	family with sequence similarity 187, member B	-2.425212	down
OR7C2	NM_012377	olfactory receptor, family 7, subfamily C, member 2	-2.4243827	down
PLCH2	NM_014638	phospholipase C, eta 2	-2.4240437	down
TBXA2R	NM_201636///NM_001060	thromboxane A2 receptor	-2.4230893	down
TEX101	NM_031451	testis expressed 101	-2.418484	down
RHBG	NM_020407	Rh family, B glycoprotein (gene/pseudogene)	-2.4132702	down
FAM57B	NM_031478	family with sequence similarity 57, member B	-2.4115717	down
KCNN2	NM_021614	potassium intermediate/small conductance calcium-activated channel, subfamily N, member 2	-2.408999	down
SPRY4	NM_030964	sprouty homolog 4 (Drosophila)	-2.407378	down
ZNF169	NM_194320	zinc finger protein 169	-2.4068968	down
FOXRED2	NM_024955	FAD-dependent oxidoreductase domain containing 2	-2.4057827	down
TYRO3	NM_006293	TYRO3 protein tyrosine kinase	-2.4029422	down
LCNL1	NM_207510	lipocalin-like 1	-2.402745	down
LOC644794	XR_133450	uncharacterized LOC644794	-2.401635	down
LOC152024	NR_026834	uncharacterized LOC152024	-2.401567	down
CHRNA6	NM_004198	cholinergic receptor, nicotinic, alpha 6	-2.3993444	down
F11	NM_000128	coagulation factor XI	-2.3980606	down
FLJ36777	AK094096	uncharacterized LOC730971	-2.3969283	down
KLK9	NM_012315	kallikrein-related peptidase 9	-2.3933306	down
YY2	NM_206923	YY2 transcription factor	-2.391668	down
KISS1	NM_002256	KiSS-1 metastasis-suppressor	-2.3910356	down
	AK127825		-2.3894765	down
C1QTNF9B	NM_001007537	C1q and tumor necrosis factor related protein 9B	-2.3884475	down
	AK092531		-2.3856747	down
ANKMY2	NM_020319	ankyrin repeat and MYND domain containing 2	-2.3828273	down
GHRLOS	NR_004431	ghrelin opposite strand/antisense RNA (non-protein coding)	-2.376792	down
INTU	NM_015693	inturned planar cell polarity effector homolog (Drosophila)	-2.3756533	down
DEFA4	NM_001925	defensin, alpha 4, corticostatin	-2.3721945	down
HIST1H2AB	NM_003513	histone cluster 1, H2ab	-2.3720171	down
DUSP9	NM_001395	dual specificity phosphatase 9	-2.3712244	down
SIGLEC16	NR_002825	sialic acid binding Ig-like lectin 16 (gene/pseudogene)	-2.3707201	down
MAP3K10	NM_002446	mitogen-activated protein kinase kinase kinase 10	-2.3705287	down
PTGER3	NM_198715	prostaglandin E receptor 3 (subtype EP3)	-2.3690603	down
LOC284628	AK094692	uncharacterized LOC284628	-2.3656733	down
KRTAP4-8	NM_031960	keratin associated protein 4-8	-2.362244	down
FOLR4	NM_001199206	folate receptor 4 (delta) homolog (mouse)	-2.3619516	down

	BF733045		-2.3607671	down
	BE613848		-2.3578959	down
	X81001		-2.3565495	down
LOC203274	BC110369	uncharacterized LOC203274	-2.3564928	down
	XM_001727011		-2.3555954	down
ZNF865	NM_001195605	zinc finger protein 865	-2.3541124	down
SLC9A3R2	NM_004785	solute carrier family 9 (sodium/hydrogen exchanger), member 3 regulator 2	-2.35131	down
GFRA2	NM_001495	GDNF family receptor alpha 2	-2.3501434	down
MTMR8	NM_017677	myotubularin related protein 8	-2.3499327	down
FAM179A		family with sequence similarity 179, member A	-2.3449762	down
C3orf54	NM_203370	chromosome 3 open reading frame 54	-2.3424883	down
FER1L6-AS1	NR_040044	FER1L6 antisense RNA 1 (non-protein coding)	-2.3423638	down
KRT26	NM_181539	keratin 26	-2.338993	down
TMEM9B	NM_020644	TMEM9 domain family, member B	-2.3321023	down
FOXD2	NM_004474	forkhead box D2	-2.3300383	down
SRCIN1	BC033233	SRC kinase signaling inhibitor 1	-2.329657	down
FAM22D	NM_001009610	family with sequence similarity 22, member D	-2.3294964	down
OR7E91P	NR_002185	olfactory receptor, family 7, subfamily E, member 91 pseudogene	-2.329453	down
GREB1	NM_148903	growth regulation by estrogen in breast cancer 1	-2.3273413	down
TP53TG3	NM_016212	TP53 target 3	-2.3255098	down
LOC728254	AB002446	uncharacterized LOC728254	-2.3216484	down
ARL5C	NM_001143968	ADP-ribosylation factor-like 5C	-2.3181145	down
TPD52	XM_001716081///NM_001025252	tumor protein D52	-2.3178868	down
	AK130324		-2.317654	down
GNN	NR_027249	Grp94 neighboring nucleotidase pseudogene	-2.3171926	down
CCR6	NM_031409	chemokine (C-C motif) receptor 6	-2.316728	down
LOC100190939	NR_024458	uncharacterized LOC100190939	-2.3166418	down
MDGA2	NM_001113498	MAM domain containing glycosylphosphatidylinositol anchor 2	-2.3162284	down
TSEN15	NM_052965	tRNA splicing endonuclease 15 homolog (S. cerevisiae)	-2.31487	down
	AK123797		-2.313963	down
	DA734585		-2.3134992	down
TRIM67	NM_001004342	tripartite motif containing 67	-2.3130445	down
GIN1	NM_017676	gypsy retrotransposon integrase 1	-2.3106217	down
HIST1H4B	NM_003544	histone cluster 1, H4b	-2.3097599	down
RPS10P7	NR_026667	ribosomal protein S10 pseudogene 7	-2.3089738	down
CEACAM18	NM_001080405	carcinoembryonic antigen-related cell adhesion molecule 18	-2.3075693	down
SLC12A5	AK098371	solute carrier family 12 (potassium/chloride transporter), member 5	-2.300993	down
FKSG2	AF300871	tumor protein, translationally-controlled 1 pseudogene	-2.2998195	down
LOC388387	NR_027254	uncharacterized LOC388387	-2.2971892	down
ERBB3	NM_001982	v-erb-b2 erythroblastic leukemia viral oncogene homolog 3 (avian)	-2.2963245	down
LOC100128551	AK125148	uncharacterized LOC100128551	-2.2947125	down
MGC11082	BC094703	uncharacterized LOC84777	-2.28615	down

AMBN	NM_016519	ameloblastin (enamel matrix protein)	-2.2857258	down
DEFB104B	NM_001040702	defensin, beta 104B	-2.2809145	down
ADAM7	NM_003817	ADAM metallopeptidase domain 7	-2.2803962	down
GPRC5B	NM_016235	G protein-coupled receptor, family C, group 5, member B	-2.2778149	down
REXO1L1	NM_172239	REX1, RNA exonuclease 1 homolog (S. cerevisiae)-like 1	-2.2776148	down
CHRND	NM_000751	cholinergic receptor, nicotinic, delta	-2.2767553	down
LOC100130872	NR_024569	uncharacterized LOC100130872	-2.2761073	down
XRCC3	NM_001100119	X-ray repair complementing defective repair in Chinese hamster cells 3	-2.2753365	down
HSCB	NM_172002	HscB iron-sulfur cluster co-chaperone homolog (E. coli)	-2.2748752	down
LOC100130745	AK128206	uncharacterized LOC100130745	-2.2734354	down
LRRTM1	NM_178839	leucine rich repeat transmembrane neuronal 1	-2.2716744	down
TSHB	NM_000549	thyroid stimulating hormone, beta	-2.2709882	down
DNPEP	NM_012100	aspartyl aminopeptidase	-2.2689023	down
CNPY1	NM_001103176	canopy 1 homolog (zebrafish)	-2.2679706	down
KRTAP10-6	NM_198688	keratin associated protein 10-6	-2.2661133	down
SLC2A10	NM_030777	solute carrier family 2 (facilitated glucose transporter), member 10	-2.265758	down
TMEM191C	AK308704	transmembrane protein 191C	-2.2651885	down
CUX2	NM_015267	cut-like homeobox 2	-2.264672	down
SMPDL3B	NM_001009568	sphingomyelin phosphodiesterase, acid-like 3B	-2.2633882	down
SYCE1L	NM_001129979	synaptonemal complex central element protein 1-like	-2.260511	down
LOC100128946	NR_038944	uncharacterized LOC100128946	-2.2601676	down
PDGFA	NM_033023	platelet-derived growth factor alpha polypeptide	-2.260119	down
SERHL2	NM_014509	serine hydrolase-like 2	-2.2583516	down
DKFZP564C152	AL049980	DKFZP564C152 protein	-2.2551537	down
LOC100652995	XR_132578	uncharacterized LOC100652995	-2.2550085	down
SLC6A16	NM_014037	solute carrier family 6, member 16	-2.2530563	down
ARHGEF26	NM_001251962	Rho guanine nucleotide exchange factor (GEF) 26	-2.2504292	down
RPL23AP7	NR_000029///NR_024530	ribosomal protein L23a pseudogene 7	-2.2488248	down
	XR_113267		-2.248518	down
CEND1	NM_016564	cell cycle exit and neuronal differentiation 1	-2.248288	down
LOC286186	NR_033893	uncharacterized LOC286186	-2.245334	down
LCE3B	NM_178433	late cornified envelope 3B	-2.2447402	down
	BC100776		-2.2444487	down
AFMID	NM_001145526	arylformamidase	-2.2444293	down
LOC100289255	NR_036581	uncharacterized LOC100289255	-2.2443118	down
SLC5A7	NM_021815	solute carrier family 5 (choline transporter), member 7	-2.2441318	down
UCMA	NM_145314	upper zone of growth plate and cartilage matrix associated	-2.2441084	down
MUC7	NM_152291	mucin 7, secreted	-2.2436132	down
OR5AK4P	AF309700	olfactory receptor, family 5, subfamily AK, member 4 pseudogene	-2.2369654	down

LINC00487	NR_038369	long intergenic non-protein coding RNA 487	-2.2349634	down
SLC30A3	NM_003459	solute carrier family 30 (zinc transporter), member 3	-2.2326071	down
ELFN2	NM_052906	extracellular leucine-rich repeat and fibronectin type III domain containing 2	-2.2323627	down
LOC100129840	AK126633	uncharacterized LOC100129840	-2.2323372	down
C5orf55	NM_138464	chromosome 5 open reading frame 55	-2.2312827	down
KRTAP19-7	NM_181614	keratin associated protein 19-7	-2.2309666	down
TELO2	NM_016111	TEL2, telomere maintenance 2, homolog (S. cerevisiae)	-2.230096	down
TAS2R9	NM_023917	taste receptor, type 2, member 9	-2.2295055	down
LINC00085	NR_024330	long intergenic non-protein coding RNA 85	-2.2285845	down
LOC728061	AK025151	hCG2003663	-2.2283885	down
GAL3ST4	NM_024637	galactose-3-O-sulfotransferase 4	-2.2270055	down
PPIP5K1	NM_001190214	diphosphoinositol pentakisphosphate kinase 1	-2.226299	down
	AY444749		-2.225928	down
PYCR1	NM_006907	pyrroline-5-carboxylate reductase 1	-2.2257733	down
NBPF3	NM_032264	neuroblastoma breakpoint family, member 3	-2.2252023	down
LCE3E	NM_178435	late cornified envelope 3E	-2.2242131	down
ZNF496	NM_032752	zinc finger protein 496	-2.2231312	down
KIRREL	NM_018240	kin of IRRE like (Drosophila)	-2.2196777	down
	AK127153		-2.2193358	down
STOX2	NM_020225	storkhead box 2	-2.2193117	down
TNFRSF13C	NM_052945	tumor necrosis factor receptor superfamily, member 13C	-2.217435	down
LINC00340	NR_015410	long intergenic non-protein coding RNA 340	-2.2140365	down
ANKRD45	NM_198493	ankyrin repeat domain 45	-2.214009	down
	XR_112173		-2.2131727	down
LOC157381	NR_027321	uncharacterized LOC157381	-2.2121453	down
MAPK15	NM_139021	mitogen-activated protein kinase 15	-2.2121444	down
LOC642980	AK131413	uncharacterized LOC642980	-2.2121215	down
CACNA1B	NM_001243812	calcium channel, voltage-dependent, N type, alpha 1B subunit	-2.2114873	down
	XR_108730		-2.21018	down
PPP1R11	NM_021959	protein phosphatase 1, regulatory (inhibitor) subunit 11	-2.2100499	down
LOC400464	XR_109229	uncharacterized LOC400464	-2.209847	down
PPYR1	NM_005972	pancreatic polypeptide receptor 1	-2.2096689	down
	BC044628		-2.2082832	down
ATP2B1	NM_001001323	ATPase, Ca++ transporting, plasma membrane 1	-2.2076125	down
OR5A1	NM_001004728	olfactory receptor, family 5, subfamily A, member 1	-2.2075155	down
LOC100130700	NR_034018	uncharacterized LOC100130700	-2.2069159	down
REN	NM_000537	renin	-2.2063065	down
EFCAB4A	NM_173584	EF-hand calcium binding domain 4A	-2.2058685	down
LOC90246	NR_026954	uncharacterized LOC90246	-2.204098	down
NPR1	NM_000906	natriuretic peptide receptor A/guanylate cyclase A (atrionatriuretic peptide receptor A)	-2.203831	down
ZNF502	NM_033210	zinc finger protein 502	-2.2026372	down
LOC729852	NR_034084	uncharacterized LOC729852	-2.2017443	down
NLGN2	NM_020795	neuroligin 2	-2.200254	down

LOC645202	XM_003403668	golgin A6 family-like	-2.197397	down
LOC283075	AK096372	uncharacterized LOC283075	-2.1970363	down
OR4X2	NM_001004727	olfactory receptor, family 4, subfamily X, member 2	-2.1962726	down
	AB529252		-2.1919396	down
CPSF4L	NM_001129885	cleavage and polyadenylation specific factor 4-like	-2.191141	down
LOC399753	AK024156	uncharacterized LOC399753	-2.1904354	down
FER1L4	NR_024377	fer-1-like 4 (C. elegans) pseudogene	-2.1879423	down
LOC100130463	AK124300	uncharacterized LOC100130463	-2.1838872	down
HPDL	NM_032756	4-hydroxyphenylpyruvate dioxygenase-like	-2.1826904	down
ALPI	NM_001631	alkaline phosphatase, intestinal	-2.1825242	down
UBE2DNL	NR_024062	ubiquitin-conjugating enzyme E2D N-terminal like (pseudogene)	-2.1790946	down
LRRC27	NM_001143757	leucine rich repeat containing 27	-2.1782818	down
LOC100132247	NM_001135865	nuclear pore complex interacting protein related gene	-2.1763976	down
GPR180	NM_180989	G protein-coupled receptor 180	-2.1761527	down
NXF5	NM_032946	nuclear RNA export factor 5	-2.1750615	down
LOC100129171	AK131268	uncharacterized LOC100129171	-2.1744747	down
C5orf63	NM_001164479	chromosome 5 open reading frame 63	-2.1723661	down
	AK090403		-2.1699007	down
ADAT2	NM_182503	adenosine deaminase, tRNA-specific 2	-2.1682281	down
DNM3	NM_015569///NM_001136127	dynamin 3	-2.1676588	down
DMKN	NM_001190348	dermokine	-2.1652613	down
FBXO36	NM_174899	F-box protein 36	-2.1638336	down
C1QTNF5	NM_015645	C1q and tumor necrosis factor related protein 5	-2.1635568	down
ZNF569	NM_152484	zinc finger protein 569	-2.1630597	down
C12orf53	NM_153685	chromosome 12 open reading frame 53	-2.1630168	down
CGB2	NM_033378	chorionic gonadotropin, beta polypeptide 2	-2.1627414	down
GSTT2B	NM_001080843	glutathione S-transferase theta 2B (gene/pseudogene)	-2.162295	down
	AK131315		-2.1620684	down
KRTAP6-1	NM_181602	keratin associated protein 6-1	-2.1597912	down
CELF5	NM_021938	CUGBP, Elav-like family member 5	-2.1591501	down
WFDC8	NM_130896	WAP four-disulfide core domain 8	-2.1588523	down
APLN	NM_017413	apelin	-2.157271	down
PTPN5	NM_006906	protein tyrosine phosphatase, non-receptor type 5 (striatum-enriched)	-2.1556683	down
TMEM117	NM_032256	transmembrane protein 117	-2.155298	down
CNTNAP3B	AB051501///NM_001201380///AK024257	contactin associated protein-like 3B	-2.1543336	down
TTTY13	NR_001537	testis-specific transcript, Y-linked 13 (non-protein coding)	-2.1502554	down
LINC00442	NR_026852	long intergenic non-protein coding RNA 442	-2.148959	down
IQCF5	NM_001145059	IQ motif containing F5	-2.148922	down
LOC100128081	NR_034097	uncharacterized LOC100128081	-2.1484575	down
LOH12CR2	NR_024061	loss of heterozygosity, 12, chromosomal region 2	-2.148283	down
GIPC3	NM_133261	GIPC PDZ domain containing family, member 3	-2.1461275	down
MYO10	NM_012334	myosin X	-2.139821	down

	CU688199		-2.1370525	down
PLBD2	NM_173542	phospholipase B domain containing 2	-2.1359034	down
CPNE7	NM_014427	copine VII	-2.1342006	down
CHCHD7	NM_001011667	coiled-coil-helix-coiled-coil-helix domain containing 7	-2.1291068	down
ZBTB7C	NM_001039360	zinc finger and BTB domain containing 7C	-2.1289392	down
CIB3	NM_054113	calcium and integrin binding family member 3	-2.1284103	down
C14orf166B	NM_194287	chromosome 14 open reading frame 166B	-2.1284084	down
PKDCC	NM_138370	protein kinase domain containing, cytoplasmic homolog (mouse)	-2.1258557	down
	AK302432		-2.1226811	down
MUC12	NM_001164462	mucin 12, cell surface associated	-2.1223652	down
BHLHA9	NM_001164405	basic helix-loop-helix family, member a9	-2.1212633	down
	BC012174		-2.120395	down
	AK026812		-2.1196752	down
	CB270075		-2.1195061	down
TSPAN1	NM_005727	tetraspanin 1	-2.1143034	down
NID2	BX648241	nidogen 2 (osteonidogen)	-2.1134534	down
UBQLN3	NM_017481	ubiquilin 3	-2.1133804	down
STMN4	NM_030795	stathmin-like 4	-2.112959	down
RMST	NR_024037	rhabdomyosarcoma 2 associated transcript (non-protein coding)	-2.112226	down
NLRP9	NM_176820	NLR family, pyrin domain containing 9	-2.1105695	down
HEPN1	NM_001037558	hepatocellular carcinoma, down-regulated 1	-2.1105409	down
CCDC9	NM_015603	coiled-coil domain containing 9	-2.1099033	down
LOC90784	NR_026984	uncharacterized LOC90784	-2.1059442	down
C16orf87	NM_001001436	chromosome 16 open reading frame 87	-2.1015024	down
ZIM2	NM_015363	zinc finger, imprinted 2	-2.1006057	down
LOC440131	NR_033889	uncharacterized LOC440131	-2.099816	down
PRSS2	NM_002770	protease, serine, 2 (trypsin 2)	-2.0979638	down
NAT8L	NM_178557	N-acetyltransferase 8-like (GCN5-related, putative)	-2.0978842	down
C14orf176	NM_001146683	chromosome 14 open reading frame 176	-2.0973334	down
CASP14	NM_012114	caspase 14, apoptosis-related cysteine peptidase	-2.0969706	down
PTK2B	NM_173174///AK128371	PTK2B protein tyrosine kinase 2 beta	-2.0965767	down
EPHA10	NM_173641	EPH receptor A10	-2.096572	down
C8orf44-SGK3	NM_001204173	C8orf44-SGK3 readthrough	-2.0951014	down
MIR143HG	NR_027180	MIR143 host gene (non-protein coding)	-2.0921316	down
LOC649201	XM_001127211	paraneoplastic antigen like 6A-like	-2.0900328	down
	AK093443		-2.0878046	down
SLC2A6	NM_017585	solute carrier family 2 (facilitated glucose transporter), member 6	-2.0866916	down
POM121L1P	NR_024591	POM121 membrane glycoprotein-like 1, pseudogene	-2.085631	down
ARNT2	NM_014862	aryl-hydrocarbon receptor nuclear translocator 2	-2.0834508	down
LBX2	NM_001009812	ladybird homeobox 2	-2.0829527	down
BCL11A	NM_022893	B-cell CLL/lymphoma 11A (zinc finger protein)	-2.0825357	down
SLC6A3	NM_001044	solute carrier family 6 (neurotransmitter transporter, dopamine), member 3	-2.0824823	down
CHODL	NM_024944	chondrolectin	-2.0823462	down

PRKAR1B	NM_001164761///NM_002735	protein kinase, cAMP-dependent, regulatory, type I, beta	-2.0816574	down
TAS2R31	NM_176885	taste receptor, type 2, member 31	-2.081264	down
LOC283888	NR_037158	uncharacterized LOC283888	-2.080609	down
TTC36	NM_001080441	tetratricopeptide repeat domain 36	-2.0795953	down
FMN1	NM_001103184	formin 1	-2.0783157	down
HBD	NM_000519	hemoglobin, delta	-2.0768418	down
FOXI3	NM_001135649	forkhead box I3	-2.0767267	down
SART1	NM_005146	squamous cell carcinoma antigen recognized by T cells	-2.0765228	down
REEP6	NM_138393	receptor accessory protein 6	-2.0735948	down
	XR_108282		-2.0734959	down
EXPH5	NM_015065	exophilin 5	-2.0723968	down
CHRNB3	NM_000749	cholinergic receptor, nicotinic, beta 3	-2.0709572	down
TFAP2A	M61156	transcription factor AP-2 alpha (activating enhancer binding protein 2 alpha)	-2.0709248	down
CACNG4	NM_014405	calcium channel, voltage-dependent, gamma subunit 4	-2.0699942	down
CST2	NM_001322	cystatin SA	-2.0673876	down
SPN	NM_001030288	sialophorin	-2.0671988	down
OMP	NM_006189	olfactory marker protein	-2.067044	down
ACCN1	NM_183377	amiloride-sensitive cation channel 1, neuronal	-2.0668747	down
ANKAR	NM_144708	ankyrin and armadillo repeat containing	-2.0664806	down
ESR1	NM_000125	estrogen receptor 1	-2.0664165	down
RABEPK	NM_005833///AL832249	Rab9 effector protein with kelch motifs	-2.065677	down
HIST1H4L	NM_003546	histone cluster 1, H4l	-2.0656586	down
	XR_110039		-2.06471	down
	AK075182		-2.062517	down
LOC440337	XR_111632	uncharacterized LOC440337	-2.0620723	down
FBXW12	NM_207102	F-box and WD repeat domain containing 12	-2.0591745	down
TTC39B		tetratricopeptide repeat domain 39B	-2.058892	down
FMR1-AS1	NR_024499	FMR1 antisense RNA 1 (non-protein coding)	-2.0567596	down
	AY203951		-2.055339	down
FAM99A	AK124823	family with sequence similarity 99, member A (non-protein coding)	-2.0544324	down
IKBKB	XR_109728///NM_001556	inhibitor of kappa light polypeptide gene enhancer in B-cells, kinase beta	-2.0532818	down
DNAJB13	NM_153614	DnaJ (Hsp40) homolog, subfamily B, member 13	-2.0516517	down
C3orf32	NM_015931	chromosome 3 open reading frame 32	-2.050691	down
DPRXP4	NR_002221	divergent-paired related homeobox pseudogene 4	-2.0499709	down
DKFZp686A05212	BX640722	uncharacterized LOC400505	-2.049751	down
	AK092418		-2.049159	down
GNAZ	NM_002073	guanine nucleotide binding protein (G protein), alpha z polypeptide	-2.048858	down
ATP2B3	NM_001001344	ATPase, Ca++ transporting, plasma membrane 3	-2.048586	down
NTRK2	NM_006180	neurotrophic tyrosine kinase, receptor, type 2	-2.0484884	down
	AB231740		-2.046863	down
MAGEA8	NM_005364	melanoma antigen family A, 8	-2.0468335	down
FAM120C	NM_017848	family with sequence similarity 120C	-2.0458806	down

FXYD3	NM_001136007	FXYD domain containing ion transport regulator 3	-2.0450728	down
LCE2A	NM_178428	late cornified envelope 2A	-2.0443916	down
ITGA11	NM_001004439	integrin, alpha 11	-2.042925	down
TMEM235	NM_001204210	transmembrane protein 235	-2.04137	down
CX3CL1	NM_002996	chemokine (C-X3-C motif) ligand 1	-2.040802	down
SH3D21	NM_001162530	SH3 domain containing 21	-2.0403612	down
VPS18	NM_020857	vacuolar protein sorting 18 homolog (S. cerevisiae)	-2.0402434	down
	XR_110925		-2.0384786	down
TMEM201	NM_001010866	transmembrane protein 201	-2.0381317	down
PABPN1L	NM_001080487	poly(A) binding protein, nuclear 1-like (cytoplasmic)	-2.0379143	down
	BX281397		-2.03724	down
HAP1	NM_177977	huntingtin-associated protein 1	-2.0368109	down
CTNND2	NM_001332	catenin (cadherin-associated protein), delta 2 (neural plakophilin-related arm-repeat protein)	-2.0353923	down
MARCH10	NM_152598	membrane-associated ring finger (C3HC4) 10	-2.0348182	down
EML6	AK131467	echinoderm microtubule associated protein like 6	-2.0318718	down
ZNF37A	NM_001007094	zinc finger protein 37A	-2.0318232	down
SDS	NM_006843	serine dehydratase	-2.0291767	down
TMEM37	NM_183240	transmembrane protein 37	-2.0291743	down
PTCH2	NM_003738	patched 2	-2.0286763	down
GAN	NM_022041	gigaxonin	-2.02859	down
TBX10	NM_005995	T-box 10	-2.0285823	down
ECHDC2	NM_018281	enoyl CoA hydratase domain containing 2	-2.0255418	down
OR7A17	NM_030901	olfactory receptor, family 7, subfamily A, member 17	-2.0245028	down
SLC10A7	NM_001029998	solute carrier family 10 (sodium/bile acid cotransporter family), member 7	-2.0240805	down
	AF416714		-2.0195365	down
	CR736467		-2.018983	down
	X58736		-2.0183103	down
JPH2	NM_175913	junctophilin 2	-2.0178995	down
ANKRD43	NM_175873	ankyrin repeat domain 43	-2.0168846	down
PPP1R13L	NM_006663	protein phosphatase 1, regulatory subunit 13 like	-2.0133767	down
TLX2	NM_016170	T-cell leukemia homeobox 2	-2.0096662	down
EXOG	NM_005107	endo/exonuclease (5'-3'), endonuclease G-like	-2.0095582	down
ARL9	NM_206919	ADP-ribosylation factor-like 9	-2.0084627	down
ITIH1	NM_002215	inter-alpha-trypsin inhibitor heavy chain 1	-2.0084305	down
OR4C15	NM_001001920	olfactory receptor, family 4, subfamily C, member 15	-2.0051599	down
ALPK1	NM_025144///AK026323	alpha-kinase 1	-2.0032086	down
IL1RL2	NM_003854	interleukin 1 receptor-like 2	-2.0026412	down
OK/SW-CL.58	AB064667	OK/SW-CL.58	-2.0018082	down
PRPH	NM_006262	peripherin	-2.0013936	down
C8orf85	NM_001025357	chromosome 8 open reading frame 85	-2.0013928	down
CCDC50	NM_178335	coiled-coil domain containing 50	-2.0002527	down
