# Supplementary material for: Transcriptional signature associated with early rheumatoid arthritis and healthy individuals at high risk to develop the disease
Source: PLoS One. 2018 Mar 27;13(3):e0194205. doi: 10.1371/journal.pone.0194205 (PMC5870959; doi:10.1371/journal.pone.0194205)
Supplement: S6 Table — (PDF) [file pone.0194205.s006.pdf]

Supplementary table 6. Up regulated genes in AR and ACCP+ groups acording Venn diagram

Gene Symbol	Genbank Accession	Gene Name	ACCP+		RA	
			Fold Change	Regulation	Fold Change	Regulation
LOC729305		uncharacterized LOC729305	4.0573115	up	2.516636	up
ARHGAP40		Rho GTPase activating protein 40	2.3690147	up	2.0468392	up
F13A1	NM_000129	coagulation factor XIII, A1 polypeptide	2.5725837	up	2.1604183	up
AFAP1	NM_001134647	actin filament associated protein 1	2.3948164	up	2.338585	up
JKAMP	NM_001284201	JNK1/MAPK8-associated membrane protein	2.2222292	up	3.0562344	up
S100P	NM_005980	S100 calcium binding protein P	3.8260586	up	4.51571	up
SSTR5-AS1	NR_027242	SSTR5 antisense RNA 1	2.1461265	up	2.5623827	up
ROBO3	NM_022370	roundabout, axon guidance receptor, homolog 3 (Drosophila)	2.1030178	up	2.0279005	up
PPP4R1L	NR_003505	protein phosphatase 4, regulatory subunit 1-like	2.8710845	up	2.1227238	up
LINC00239	NR_026774	long intergenic non-protein coding RNA 239	2.0563939	up	2.4558783	up
	JF834318		2.1773176	up	2.0780642	up
TEFM	NM_024683	transcription elongation factor, mitochondrial	2.1226554	up	2.3261764	up
ACER1	NM_133492	alkaline ceramidase 1	2.399555	up	2.0135407	up
STK17B	BC052561	serine/threonine kinase 17b	2.6423168	up	2.6169624	up
LINC01123	NR_046110	long intergenic non-protein coding RNA 1123	3.4334328	up	2.0207531	up
Unkown			2.7547994	up	2.466904	up
TGM2	NM_198951	transglutaminase 2	2.2009268	up	2.7073684	up
