# Supplementary material for: Transcriptional signature associated with early rheumatoid arthritis and healthy individuals at high risk to develop the disease
Source: PLoS One. 2018 Mar 27;13(3):e0194205. doi: 10.1371/journal.pone.0194205 (PMC5870959; doi:10.1371/journal.pone.0194205)
Supplement: S7 Table — (PDF) [file pone.0194205.s007.pdf]

Supplementary table 7. Down regulated genes in AR and ACCP+ groups acording Venn diagram

Gene Symbol	Genbank Accession	Gene Name	ACCP+		RA	
			Fold Change	Regulation	Fold Change	Regulation
FGF6	NM_020996	fibroblast growth factor 6	-2.7356706	down	-2.4612348	down
DCDC1	NM_181807	doublecortin domain containing 1	-10.398871	down	-5.908167	down
RSPH1	NM_080860	radial spoke head 1 homolog (Chlamydomonas)	-3.0131872	down	-2.359272	down
IZUMO2	NM_152358	IZUMO family member 2	-2.4402485	down	-2.1440845	down
Unknown			-5.4844894	down	-3.5735257	down
FAM205B	AL080137	transmembrane protein C9orf144B pseudogene	-6.756355	down	-3.9766746	down
C1orf226	NM_001085375	chromosome 1 open reading frame 226	-4.013202	down	-2.8179643	down
LINC00870		long intergenic non-protein coding RNA 870	-4.914358	down	-2.27644	down
RBMS3	NM_014483	RNA binding motif, single stranded interacting protein 3	-2.7508397	down	-2.1848514	down
SSMEM1	NM_145268	serine-rich single-pass membrane protein 1	-5.0435715	down	-2.457924	down
LOC100128402	AK124574	uncharacterized LOC100128402	-3.041526	down	-3.337858	down
IPO7	NM_006391	importin 7	-2.9247923	down	-2.0830383	down
ETV2	NM_014209	ets variant 2	-2.3904889	down	-2.1907458	down
CCKAR	NM_000730	cholecystokinin A receptor	-3.2449987	down	-2.1643167	down
SDAD1	AF132198	SDA1 domain containing 1	-3.088813	down	-2.3475528	down
KDELR3	NM_016657	KDEL (Lys-Asp-Glu-Leu) endoplasmic reticulum protein retention receptor 3	-2.8405354	down	-2.1010847	down
	DR007925		-2.7181683	down	-3.0071445	down
C1S	NM_201442	complement component 1, s subcomponent	-3.7875316	down	-2.2595763	down
CYP27B1	NM_000785	cytochrome P450, family 27, subfamily B, polypeptide 1	-2.458018	down	-2.2186098	down
RIIAD1	NM_001144956	regulatory subunit of type II PKA R-subunit (RIIa) domain containing 1	-3.7718177	down	-2.486339	down
LARP6	NM_197958	La ribonucleoprotein domain family, member 6	-2.0188422	down	-3.1811519	down
GRP	NM_002091	gastrin-releasing peptide	-2.6148796	down	-2.2236316	down
GPR135	NM_022571	G protein-coupled receptor 135	-3.2988245	down	-2.2938108	down
SPATA31D3	NM_207416	SPATA31 subfamily D, member 3	-3.8351433	down	-2.0161974	down
GHRHR	NM_000823	growth hormone releasing hormone receptor	-3.2994812	down	-2.1087446	down
PGPEP1L	NM_001167902	pyroglutamyl-peptidase I-like	-2.9867265	down	-3.1144521	down
Unknown			-4.23424	down	-2.114345	down
	BX107671		-4.1807947	down	-2.2888033	down
DEFB103A	NM_001081551	defensin, beta 103A	-3.5569656	down	-2.1911187	down
PLEKHD1	NM_001161498	pleckstrin homology domain containing, family D (with coiled-coil domains) member 1	-2.2023618	down	-2.6186786	down
LCE2C	NM_178429	late cornified envelope 2C	-5.6939993	down	-2.5052247	down
TAS2R14	NM_023922	taste receptor, type 2, member 14	-4.24592	down	-2.5433955	down
MYO15B	BC128044	myosin XVB pseudogene	-3.9022787	down	-4.016065	down
ASAP3	NM_017707	ArfGAP with SH3 domain, ankyrin repeat and PH domain 3	-3.2008781	down	-2.8068252	down
LOC442028	NR_037597	uncharacterized LOC442028	-5.5398593	down	-2.8493435	down
XLOC_014512			-6.898358	down	-2.9503536	down
Unknown			-3.1783042	down	-4.625366	down
TBX6	NM_004608	T-box 6	-5.7063303	down	-6.1043735	down
HS3ST4	NM_006040	heparan sulfate (glucosamine) 3-O-sulfotransferase 4	-2.5890305	down	-2.0810814	down
NGEF	NM_019850	neuronal guanine nucleotide exchange factor	-6.0617447	down	-4.2346206	down
GTF2IRD1	NM_005685	GTF2I repeat domain containing 1	-4.403517	down	-3.1708293	down
SOST	NM_025237	sclerostin	-4.5408583	down	-3.002744	down
EHMT1-IT1	NR_024341	EHMT1 intronic transcript 1	-2.2236643	down	-2.1003883	down
KCNC1	NM_004976	potassium voltage-gated channel, Shaw-related subfamily, member 1	-3.9483259	down	-2.2664573	down
Unknown			-6.285416	down	-3.8360286	down
	X13953		-3.6568775	down	-3.1475484	down
POU3F2	NM_005604	POU class 3 homeobox 2	-3.9604735	down	-2.2795503	down
KRT79	NM_175834	keratin 79	-3.5263817	down	-2.656418	down
Unknown			-4.829571	down	-3.9486558	down
LOC145694	AK056793	uncharacterized LOC145694	-2.6336966	down	-2.4219522	down
INHBA	NM_002192	inhibin, beta A	-7.26326	down	-4.1262827	down
TERT	NM_198253	telomerase reverse transcriptase	-5.4082713	down	-3.9636452	down
CCL1	NM_002981	chemokine (C-C motif) ligand 1	-3.7698221	down	-2.4017992	down
RARRES2	NM_002889	retinoic acid receptor responder (tazarotene induced) 2	-7.238941	down	-4.8614817	down
MTPAP	XR_171057	mitochondrial poly(A) polymerase	-5.255531	down	-3.2774725	down
UGT2B11	NM_001073	UDP glucuronosyltransferase 2 family, polypeptide B11	-4.501039	down	-6.346666	down
RDM1	NM_001034836	RAD52 motif 1	-3.3240294	down	-2.9162483	down

THBS2	NM_003247	thrombospondin 2	-3.0687163	down	-2.9432383	down
WTH3DI	NM_001077637	RAB6C-like	-7.232513	down	-3.3727872	down
MUC17	NM_001040105	mucin 17, cell surface associated	-2.6185644	down	-2.5495353	down
Unknown			-3.5285816	down	-3.2851312	down
	XR_247077		-4.0376334	down	-7.0911107	down
DPCR1	NM_080870	diffuse panbronchiolitis critical region 1	-4.074805	down	-5.749843	down
	XR_246934		-4.9533257	down	-2.9334037	down
USP17L7	NM_001256869	ubiquitin specific peptidase 17-like family member 7	-2.4822962	down	-2.005743	down
SLC35G5	NM_054028	solute carrier family 35, member G5	-2.6206124	down	-3.3485498	down
LOC100996457	XR_108874	uncharacterized LOC100996457	-2.7944608	down	-2.557444	down
	AK025047		-3.5295773	down	-2.2335503	down
LOC285484	NR_037863	uncharacterized LOC285484	-4.1093225	down	-2.2150912	down
Unknown			-3.814625	down	-2.0048096	down
MYPN	NM_032578	myopalladin	-4.1722775	down	-2.060425	down
	BC031250		-3.3856745	down	-4.2014008	down
	DA144211		-3.3923743	down	-2.0575192	down
CALCR	NM_001164737	calcitonin receptor	-4.8522997	down	-3.1915514	down
KANK1	AL832454	KN motif and ankyrin repeat domains 1	-3.8645802	down	-2.398038	down
PTCH2	XM_005271301	patched 2	-5.1223755	down	-4.3255506	down
MAST4	AK299538	microtubule associated serine/threonine kinase family member 4	-7.7951584	down	-7.23512	down
RLN3	NM_080864	relaxin 3	-4.7238793	down	-2.0871866	down
ULBP3	NM_024518	UL16 binding protein 3	-4.199691	down	-2.414395	down
KDM4A-AS1	NR_033827	KDM4A antisense RNA 1	-3.6983013	down	-3.0804498	down
GABRB1	NM_000812	gamma-aminobutyric acid (GABA) A receptor, beta 1	-3.4232345	down	-2.02682	down
KRT33B	NM_002279	keratin 33B	-3.127337	down	-3.0449245	down
Unknown			-4.4404907	down	-3.190821	down
	BC136790		-5.1337814	down	-4.031147	down
FLJ25758	NR_024372	MAP/microtubule affinity-regulating kinase 1 pseudogene	-3.8291676	down	-2.172661	down
CEACAM16	NM_001039213	carcinoembryonic antigen-related cell adhesion molecule 16	-4.22323	down	-2.5336459	down
LOC100130930	AK126579	uncharacterized LOC100130930	-2.2687356	down	-2.7929077	down
GOLGA8M	NM_001282468	golgin A8 family, member M	-3.0344326	down	-3.772159	down
IQSEC3	BC024764	IQ motif and Sec7 domain 3	-5.352266	down	-2.3883328	down
	AK131313		-4.142166	down	-2.7875314	down
SCARA5	AY337579	scavenger receptor class A, member 5 (putative)	-3.6457164	down	-2.2809887	down
CATSPERD	NM_152784	catsper channel auxiliary subunit delta	-4.006151	down	-2.8033879	down
GRIK3	NM_000831	glutamate receptor, ionotropic, kainate 3	-2.6034112	down	-2.8780453	down
EMR4P	NR_024075	egf-like module containing, mucin-like, hormone receptor-like 4 pseudogene	-5.591217	down	-3.8318007	down
STON1	AK128867	stonin 1	-2.3545156	down	-2.339463	down
NAV2-AS5	NR_049725	NAV2 antisense RNA 5	-3.8621438	down	-3.5178869	down
DKK4	NM_014420	dickkopf WNT signaling pathway inhibitor 4	-5.601517	down	-2.437393	down
CSMD2	NM_052896	CUB and Sushi multiple domains 2	-3.7003953	down	-2.3411067	down
	AK128128		-4.6943436	down	-3.5356994	down
Unknown			-4.7469234	down	-2.6931462	down
Unknown			-2.5339923	down	-2.678121	down
	BC131768		-4.7090745	down	-3.5131736	down
KCTD11	NM_001002914	potassium channel tetramerization domain containing 11	-2.3383977	down	-2.2699606	down
	AF072164		-5.8852663	down	-4.9391103	down
TCHHL1	NM_001008536	trichohyalin-like 1	-4.5610495	down	-2.0612605	down
LOC728503	XM_001127575	uncharacterized LOC728503	-2.6312406	down	-4.228413	down
GATA4	NM_002052	GATA binding protein 4	-4.3179	down	-2.033399	down
	AF289593		-4.6415443	down	-3.0451298	down
LOC729159	NM_001282301	UPF0607 protein ENSP00000381418-like	-3.9189873	down	-2.8732407	down
XLOC_014512			-2.6954098	down	-2.1575031	down
OTOP1	NM_177998	otopetrin 1	-4.9926395	down	-2.1152172	down
OR2C1	NM_012368	olfactory receptor, family 2, subfamily C, member 1	-5.64337	down	-2.673744	down
Unknown			-6.2617574	down	-4.1162167	down
CDX2	NM_001265	caudal type homeobox 2	-3.307679	down	-2.2728572	down
DNM1P46	NR_003260	DNM1 pseudogene 46	-2.3741019	down	-2.2783635	down
HABP2	NM_004132	hyaluronan binding protein 2	-7.531104	down	-2.9742222	down
LOC100129393	AK124631	uncharacterized LOC100129393	-5.76749	down	-3.2786276	down

FAM106CP	NR_026810	family with sequence similarity 106, member C pseudogene	-4.9130793	down	-2.1905699	down
DNPEP	NM_012100	aspartyl aminopeptidase	-3.647235	down	-2.3839345	down
Unknown			-2.6869447	down	-2.4030042	down
LOC283728	AK095617	uncharacterized LOC283728	-6.717494	down	-3.7149692	down
TRIM36	BC017346	tripartite motif containing 36	-2.6318202	down	-5.316765	down
LOC729305		uncharacterized LOC729305	-7.486693	down	-5.7991824	down
MEDAG	NM_032849	mesenteric estrogen-dependent adipogenesis	-2.7451317	down	-2.6747673	down
NPIPB5	NM_001135865	nuclear pore complex interacting protein family, member B5	-3.6593182	down	-2.2041225	down
Unknown			-5.1192384	down	-2.0018158	down
FREM2	NM_207361	FRAS1 related extracellular matrix protein 2	-3.0345447	down	-2.993317	down
	AK124041		-3.7615404	down	-2.1995711	down
LOC389332	NR_024418	uncharacterized LOC389332	-4.722308	down	-2.1874394	down
GINS2	NM_016095	GINS complex subunit 2 (Psf2 homolog)	-5.0490203	down	-8.626691	down
Unknown			-2.1159	down	-2.7930276	down
SERPINB3	NM_006919	serpin peptidase inhibitor, clade B (ovalbumin), member 3	-4.2726974	down	-2.4826992	down
GABRQ	NM_018558	gamma-aminobutyric acid (GABA) A receptor, theta	-4.7511625	down	-2.6876967	down
RASSF4		Ras association (RalGDS/AF-6) domain family member 4	-2.621052	down	-2.224959	down
DOC2B	NM_003585	double C2-like domains, beta	-3.6279755	down	-2.4832351	down
Unknown			-2.4468224	down	-2.0665631	down
HRASLS2	NM_017878	HRAS-like suppressor 2	-3.0453184	down	-2.728009	down
AKAP4	NM_003886	A kinase (PRKA) anchor protein 4	-4.6666627	down	-2.4355173	down
PRR7	AK125417	proline rich 7 (synaptic)	-7.072389	down	-4.424351	down
Unknown			-6.7537675	down	-5.382475	down
LOC642852		uncharacterized LOC642852	-5.164522	down	-3.6159494	down
PCDHB11	NM_018931	protocadherin beta 11	-3.6656606	down	-2.79214	down
C5orf64	NM_173667	chromosome 5 open reading frame 64	-4.4786873	down	-4.768939	down
NUDT8	NM_001243750	nudix (nucleoside diphosphate linked moiety X)-type motif 8	-2.9733312	down	-3.5824828	down
Unknown			-3.9522982	down	-7.947017	down
AOC4P	NR_002773	amine oxidase, copper containing 4, pseudogene	-4.6877036	down	-4.0430007	down
RNU4ATAC	DW419002	RNA, U4atac small nuclear (U12-dependent splicing)	-2.18159	down	-2.3195674	down
SLC25A51	NR_024872	solute carrier family 25, member 51	-4.5600233	down	-2.30282	down
Unknown			-3.9081805	down	-2.5478723	down
XLOC_014512			-5.7082195	down	-2.5708065	down
	Z18843		-3.573436	down	-2.689854	down
LOC729966	NR_036575	uncharacterized LOC729966	-4.5013766	down	-2.4443069	down
	BC020894		-3.053141	down	-2.4803283	down
NR2E3	NM_014249	nuclear receptor subfamily 2, group E, member 3	-3.1002529	down	-2.0687454	down
	AK125981		-2.166294	down	-2.5252504	down
ATP8B3	NM_138813	ATPase, aminophospholipid transporter, class I, type 8B, member 3	-4.734704	down	-2.2276285	down
	XR_247172		-2.9470575	down	-3.4689136	down
ZNF562	NM_017656	zinc finger protein 562	-2.8975852	down	-2.0377355	down
GGA1		golgi-associated, gamma adaptin ear containing, ARF binding protein 1	-3.1121163	down	-2.6645577	down
CYP3A4	NM_017460	cytochrome P450, family 3, subfamily A, polypeptide 4	-2.2372468	down	-2.4023285	down
LAMB1	BC044633	laminin, beta 1	-4.313568	down	-2.4714444	down
COPE	AK023303	coatomer protein complex, subunit epsilon	-3.761863	down	-2.519379	down
MLLT4	NM_001207008	myeloid/lymphoid or mixed-lineage leukemia (trithorax homolog, Drosophila); translocated to, 4	-4.9150195	down	-3.0376167	down
LINC00330		long intergenic non-protein coding RNA 330	-4.0354204	down	-2.9228003	down
SERTM1	NM_203451	serine-rich and transmembrane domain containing 1	-5.026436	down	-3.9387424	down
IGFL3	NM_207393	IGF-like family member 3	-5.951504	down	-4.200748	down
C19orf55	NM_001039887	chromosome 19 open reading frame 55	-2.8610198	down	-2.3949616	down
XLOC_001500			-2.8034983	down	-2.3815846	down
BMP15	NM_005448	bone morphogenetic protein 15	-4.061518	down	-2.9643145	down
	AK024188		-3.879919	down	-2.5607195	down
SFRP1	NM_003012	secreted frizzled-related protein 1	-5.789137	down	-3.3551188	down
PCDHGB7	NM_032101	protocadherin gamma subfamily B, 7	-4.946394	down	-3.278794	down
SH2D4A	NM_022071	SH2 domain containing 4A	-4.0896015	down	-2.4754755	down
PGM5-AS1	NR_015423	PGM5 antisense RNA 1	-5.4077306	down	-2.2824948	down
LZTS2	AK097997	leucine zipper, putative tumor suppressor 2	-3.541616	down	-2.420541	down

Unknown			-3.657871	down	-3.2616613	down
Unknown			-3.0938025	down	-2.5670633	down
LOC100129316		uncharacterized LOC100129316	-4.417311	down	-2.3271677	down
Unknown			-4.039256	down	-2.8542833	down
LINC01120		long intergenic non-protein coding RNA 1120	-3.914519	down	-2.151608	down
PPP1R26	NM_014811	protein phosphatase 1, regulatory subunit 26	-4.199215	down	-2.507344	down
MICU1	XM_005269387	mitochondrial calcium uptake 1	-3.7358396	down	-2.1282022	down
	DA734158		-5.4436846	down	-3.0927353	down
NRSN2	NM_024958	neurensin 2	-2.7972322	down	-2.9623015	down
PMFBP1	NM_031293	polyamine modulated factor 1 binding protein 1	-3.627016	down	-2.312073	down
Unknown			-3.438147	down	-2.4092784	down
RBBP8NL	NM_080833	RBBP8 N-terminal like	-2.546231	down	-2.3318949	down
KIFC2	NM_145754	kinesin family member C2	-2.2041512	down	-3.0426295	down
NANOS2	NM_001029861	nanos homolog 2 (Drosophila)	-3.345633	down	-2.1999478	down
LAMC3	NM_006059	laminin, gamma 3	-5.9667172	down	-4.0606203	down
LOC145837	NR_026979	uncharacterized LOC145837	-5.6042867	down	-3.6114953	down
BARX2	NM_003658	BARX homeobox 2	-3.737969	down	-2.2483613	down
Unknown			-2.2038448	down	-2.0048492	down
LOC254057	AK024653	uncharacterized LOC254057	-3.7843974	down	-2.1027448	down
Unknown			-2.1341243	down	-3.454256	down
LOC100128333	AK127681	uncharacterized LOC100128333	-4.274669	down	-2.4480226	down
MAGEB1	NM_002363	melanoma antigen family B, 1	-2.4571786	down	-2.6537116	down
RXFP2	NM_130806	relaxin/insulin-like family peptide receptor 2	-2.9666843	down	-2.364773	down
LOC100190940	AK127723	uncharacterized LOC100190940	-3.059862	down	-2.2174277	down
XLOC_006339			-4.4362917	down	-4.6378016	down
NODAL	NM_018055	nodal growth differentiation factor	-4.4517674	down	-3.1945043	down
Unknown			-2.1858826	down	-2.900224	down
	XM_003118552		-7.4920907	down	-6.7279897	down
CYP7A1	NM_000780	cytochrome P450, family 7, subfamily A, polypeptide 1	-5.9586134	down	-2.5759485	down
Unknown			-4.04905	down	-2.8362055	down
	AK098235		-2.4968948	down	-2.2333887	down
ZNF280A	NM_080740	zinc finger protein 280A	-5.2506285	down	-2.409288	down
GSG1	NM_001080554	germ cell associated 1	-5.0986567	down	-5.013273	down
	AF090909		-4.7112746	down	-3.1378796	down
C15orf60	NM_001042367	chromosome 15 open reading frame 60	-3.8633578	down	-2.0673096	down
	CR989582		-2.9639716	down	-2.0196733	down
LOC392364	NR_040117	nuclear pore associated protein 1 pseudogene	-2.1191466	down	-2.0676107	down
IL36A	NM_014440	interleukin 36, alpha	-2.9777825	down	-2.8281875	down
LOC389834	NR_027420	ankyrin repeat domain 57 pseudogene	-2.6569471	down	-2.187541	down
AOX1	NM_001159	aldehyde oxidase 1	-3.2718687	down	-2.6017566	down
Unknown			-3.7319307	down	-2.4459352	down
KMT2E	NM_182931	lysine (K)-specific methyltransferase 2E	-2.1595058	down	-2.8510187	down
AVPR2	NM_000054	arginine vasopressin receptor 2	-4.2036986	down	-2.8083498	down
C11orf52	NM_080659	chromosome 11 open reading frame 52	-7.6105685	down	-3.7092638	down
LINC00467	NR_026761	long intergenic non-protein coding RNA 467	-4.959096	down	-3.2555642	down
Unknown			-3.117215	down	-2.092665	down
	AK024162		-8.17975	down	-5.4059706	down
SRRM3	NM_001110199	serine/arginine repetitive matrix 3	-2.890344	down	-3.6287715	down
ELK4	NM_021795	ELK4, ETS-domain protein (SRF accessory protein 1)	-2.567827	down	-2.4673357	down
GGT8P		gamma-glutamyltransferase 8 pseudogene	-3.9339473	down	-4.8570437	down
GRIA3	NM_000828	glutamate receptor, ionotropic, AMPA 3	-6.105583	down	-3.609577	down
CEBPA-AS1	NR_026887	CEBPA antisense RNA 1 (head to head)	-2.609788	down	-2.1593254	down
S100A14	NM_020672	S100 calcium binding protein A14	-6.1257925	down	-4.210981	down
RAPGEF3	U78169	Rap guanine nucleotide exchange factor (GEF) 3	-2.9180486	down	-2.2429178	down
LRP2	NM_004525	low density lipoprotein receptor-related protein 2	-8.930958	down	-10.938198	down
OR52I2	NM_001005170	olfactory receptor, family 52, subfamily I, member 2	-4.631544	down	-2.2155972	down
Unknown			-2.473258	down	-2.5089257	down
Unknown			-4.810202	down	-4.0312285	down
COL27A1	BC007696	collagen, type XXVII, alpha 1	-3.8672407	down	-2.0218265	down
THEG5	NM_001278577	testis highly expressed protein 5	-3.1596646	down	-2.3005366	down
LRPPRC	NM_133259	leucine-rich pentatricopeptide repeat containing	-2.0540526	down	-3.1235523	down
FOXQ1	NM_033260	forkhead box Q1	-4.5112844	down	-3.5405126	down

KCNA7	NM_031886	potassium voltage-gated channel, shaker-related subfamily, member 7	-4.4486423	down	-2.1563544	down
SLC24A5	NM_205850	solute carrier family 24 (sodium/potassium/calcium exchanger), member 5	-9.810018	down	-5.512019	down
			-5.6948113	down	-3.6623635	down
LOC729159	NM_001282301	UPF0607 protein ENSP00000381418-like	-5.6992316	down	-2.9852264	down
			-4.064507	down	-2.290563	down
	DR007930		-6.077233	down	-3.9309983	down
CACNA1B		calcium channel, voltage-dependent, N type, alpha 1B subunit	-3.1216295	down	-2.0461526	down
	BC040680		-2.3249002	down	-2.9862437	down
GCNT4	NM_016591	glucosaminyl (N-acetyl) transferase 4, core 2	-4.082605	down	-2.5956194	down
Unknown			-2.9892886	down	-2.8009536	down
MDH1B	NM_001039845	malate dehydrogenase 1B, NAD (soluble)	-4.422717	down	-2.4499521	down
LINC01107		long intergenic non-protein coding RNA 1107	-2.787989	down	-2.4732323	down
KCP	NM_199349	kielin/chordin-like protein	-3.7424371	down	-2.190662	down
HCN3	NM_020897	hyperpolarization activated cyclic nucleotide-gated potassium channel 3	-2.589922	down	-2.6795626	down
	XR_241335		-6.037035	down	-3.5204198	down
Unknown			-5.662252	down	-3.6131644	down
ATP10D	NM_020453	ATPase, class V, type 10D	-2.8716946	down	-2.5520403	down
MAGEA2B	NM_153488	melanoma antigen family A, 2B	-7.9043283	down	-3.4460616	down
LINC00588	NR_026772	long intergenic non-protein coding RNA 588	-3.5732524	down	-3.3366601	down
PTK6	NM_005975	protein tyrosine kinase 6	-2.8881297	down	-4.342905	down
FAM71A	NM_153606	family with sequence similarity 71, member A	-4.6503105	down	-2.6628368	down
RASSF6	NM_177532	Ras association (RalGDS/AF-6) domain family member 6	-4.3898015	down	-3.694323	down
Unknown			-3.3953197	down	-2.6536207	down
TPD52		tumor protein D52	-2.5669	down	-2.0229888	down
ARHGAP44	NM_014859	Rho GTPase activating protein 44	-2.2771447	down	-2.3134844	down
Unknown			-3.4707677	down	-2.7637691	down
LOC400743		uncharacterized LOC400743	-4.7660003	down	-3.1892774	down
GAGE7	NM_021123	G antigen 7	-8.785406	down	-4.559126	down
Unknown			-2.0609853	down	-2.0418484	down
Unknown			-2.4924583	down	-3.1730504	down
Unknown			-3.928244	down	-6.8913774	down
LAT2	XM_005250562	linker for activation of T cells family, member 2	-3.56985	down	-3.0589664	down
Unknown			-2.1434238	down	-3.1212816	down
LOC101929998		uncharacterized LOC101929998	-2.7336268	down	-3.6147633	down
LOC647070	AK001442	uncharacterized LOC647070	-2.8765714	down	-2.4439862	down
AFF3		AF4/FMR2 family, member 3	-6.0185285	down	-2.695566	down
C1orf51	NM_144697	chromosome 1 open reading frame 51	-3.3635309	down	-2.4202318	down
CSRP2	NM_001321	cysteine and glycine-rich protein 2	-15.822509	down	-22.980167	down
MDM4	NM_001278518	Mdm4 p53 binding protein homolog (mouse)	-3.3000515	down	-3.1185024	down
	DC378344		-2.4340184	down	-2.378194	down
XLOC_014512			-3.158004	down	-4.3220015	down
	XR_245224		-4.198692	down	-3.5886817	down
OR51G1	NM_001005237	olfactory receptor, family 51, subfamily G, member 1	-2.267934	down	-2.8088446	down
GPR158-AS1	NR_027333	GPR158 antisense RNA 1	-5.1155467	down	-3.0326595	down
GOLGA6L5P		golgin A6 family-like 5, pseudogene	-5.956165	down	-4.1409206	down
MAEL	NM_032858	maelstrom spermatogenic transposon silencer	-2.0187945	down	-2.2612245	down
S100A1	NM_006271	S100 calcium binding protein A1	-3.8897114	down	-2.3622494	down
Unknown			-6.775108	down	-2.6913917	down
SOBP	NM_018013	sine oculis binding protein homolog (Drosophila)	-2.8791604	down	-2.1060286	down
SH2D6	XM_005264269	SH2 domain containing 6	-2.6459694	down	-2.6259198	down
ST8SIA1	NM_003034	ST8 alpha-N-acetyl-neuraminide alpha-2,8-sialyltransferase 1	-4.3716426	down	-4.096914	down
RPL23AP64	NR_003040	ribosomal protein L23a pseudogene 64	-3.2202754	down	-2.6580968	down
DEPDC5	NM_001007188	DEP domain containing 5	-3.2977488	down	-2.033664	down
Unknown			-4.1415133	down	-2.4126246	down
ANKRD13B	NM_152345	ankyrin repeat domain 13B	-9.177254	down	-5.5920157	down
TEX13A	NM_031274	testis expressed 13A	-3.496359	down	-2.1771133	down
WDR87	NM_031951	WD repeat domain 87	-6.4895234	down	-6.350717	down
TPTE2P3	NR_002793	transmembrane phosphoinositide 3-phosphatase and tensin homolog 2 pseudogene 3	-4.541015	down	-2.8170989	down
Unknown			-5.0733256	down	-3.350116	down

COL5A3	NM_015719	collagen, type V, alpha 3	-2.447098	down	-2.3167112	down
TULP1	NM_003322	tubby like protein 1	-3.1633475	down	-2.0884333	down
TRPC2		transient receptor potential cation channel, subfamily C, member 2, pseudogene	-3.6966984	down	-2.4366245	down
KRTAP4-2	NM_033062	keratin associated protein 4-2	-4.4064183	down	-2.0248802	down
FTCD	NM_206965	formimidoyltransferase cyclodeaminase	-4.3600597	down	-2.5420594	down
LINC00371	NR_102432	long intergenic non-protein coding RNA 371	-3.575458	down	-2.6738272	down
GAS2L2	NM_139285	growth arrest-specific 2 like 2	-3.9202523	down	-2.6415405	down
Unknown			-2.3967628	down	-2.568622	down
NEK10	XM_005265644	NIMA-related kinase 10	-3.131731	down	-2.1835463	down
Unknown			-5.649989	down	-2.1255703	down
DUOXA1	NM_001276266	dual oxidase maturation factor 1	-2.4777508	down	-3.0396867	down
USP17L8	NM_001256872	ubiquitin specific peptidase 17-like family member 8	-2.9799976	down	-2.5991228	down
LOC100131581	AK092544	uncharacterized LOC100131581	-8.597723	down	-8.233938	down
RAX	NM_013435	retina and anterior neural fold homeobox	-4.5225215	down	-2.3905017	down
CSTL1	NM_138283	cystatin-like 1	-4.9539375	down	-2.3075707	down
HECTD2	NM_173497	HECT domain containing E3 ubiquitin protein ligase 2	-5.39886	down	-2.4544797	down
VSTM2A	NM_182546	V-set and transmembrane domain containing 2A	-4.4006214	down	-2.1677985	down
ABCD3	NM_001122674	ATP-binding cassette, sub-family D (ALD), member 3	-4.1378484	down	-2.609972	down
GPR61	NM_031936	G protein-coupled receptor 61	-4.045343	down	-2.4386528	down
LAMA5	BC015386	laminin, alpha 5	-3.6840506	down	-2.5294468	down
Unknown			-3.5418634	down	-2.0559359	down
DISC1	NM_001164550	disrupted in schizophrenia 1	-3.5727456	down	-4.0900855	down
LOC100130542	AK127083	uncharacterized LOC100130542	-3.4345715	down	-2.8147304	down
KIAA1755	NM_001029864	KIAA1755	-2.851643	down	-2.2032206	down
	AK095621		-3.589049	down	-3.285275	down
Unknown			-3.0009613	down	-2.8335786	down
Unknown			-4.9574223	down	-2.9292002	down
	XM_005276704		-3.2809958	down	-6.4335713	down
LOC284551	NR_027085	uncharacterized LOC284551	-4.7185216	down	-2.3071935	down
C6orf10	NM_006781	chromosome 6 open reading frame 10	-3.8626244	down	-2.1796014	down
			-8.049088	down	-4.9856873	down
TUBB8	NM_177987	tubulin, beta 8 class VIII	-4.7999144	down	-3.1990798	down
SLC9A3R2	NM_004785	solute carrier family 9, subfamily A (NHE3, cation proton antiporter 3), member 3 regulator 2	-2.3047967	down	-2.225512	down
MAP3K4	NM_006724	mitogen-activated protein kinase kinase kinase 4	-3.8696704	down	-2.7849026	down
FLJ45743		uncharacterized LOC642484	-2.763872	down	-2.9401739	down
TTTY13		testis-specific transcript, Y-linked 13 (non-protein coding)	-2.0200555	down	-2.388504	down
OR51I2	NM_001004754	olfactory receptor, family 51, subfamily I, member 2	-4.278258	down	-2.84366	down
CERCAM	NM_016174	cerebral endothelial cell adhesion molecule	-5.9602847	down	-7.717456	down
LOC100128374	XR_112969	uncharacterized LOC100128374	-3.172776	down	-2.3091383	down
BRINP3	NM_199051	bone morphogenetic protein/retinoic acid inducible neural-specific 3	-3.4584541	down	-4.288718	down
KLHL4	NM_057162	kelch-like family member 4	-2.0216594	down	-2.8599212	down
LOC101930210	XR_246852	protein capicua homolog	-6.283665	down	-4.3714232	down
Unknown			-2.9050434	down	-2.9069355	down
C1orf52	NM_198077	chromosome 1 open reading frame 52	-3.0224564	down	-2.9682403	down
KCNQ2	AK293727	potassium voltage-gated channel, KQT-like subfamily, member 2	-3.7738152	down	-2.6751084	down
	AB305786		-3.074929	down	-2.3179505	down
KIAA1161	NM_020702	KIAA1161	-2.604228	down	-2.6965714	down
OR9G9	NM_001013358	olfactory receptor, family 9, subfamily G, member 9	-3.4962032	down	-2.3524234	down
GDAP1L1	NM_024034	ganglioside induced differentiation associated protein 1-like 1	-3.154506	down	-2.310358	down
SCARNA5	NR_003008	small Cajal body-specific RNA 5	-5.604073	down	-3.9698277	down
KRT6A	NM_005554	keratin 6A	-2.7788346	down	-2.9003875	down
Unknown			-3.849484	down	-3.1290665	down
ZNF526	NM_133444	zinc finger protein 526	-2.2385113	down	-2.008108	down
TMEM201	NM_001130924	transmembrane protein 201	-2.0417995	down	-2.8604376	down
XAGE-4	XR_113307	XAGE-4 protein	-3.8336847	down	-2.8020458	down
Unknown			-2.930544	down	-3.4956048	down
OR4F21	NM_001005504	olfactory receptor, family 4, subfamily F, member 21	-4.8957086	down	-2.3006544	down
Unknown			-2.5361595	down	-2.1157777	down
Unknown			-2.683017	down	-2.303116	down

CYP4F24P		cytochrome P450, family 4, subfamily F, polypeptide 24, pseudogene	-2.3393188	down	-2.5150135	down
BARHL2	NM_020063	BarH-like homeobox 2	-4.315287	down	-2.1635277	down
UPB1	NM_016327	ureidopropionase, beta	-2.7191365	down	-2.5708623	down
Unknown			-5.385691	down	-2.3648338	down
GSG1	NM_031289	germ cell associated 1	-4.493778	down	-2.3850048	down
Unknown			-5.0339055	down	-3.1702468	down
CALY		calcyon neuron-specific vesicular protein	-2.7939858	down	-2.565098	down
SLC16A13	NM_201566	solute carrier family 16, member 13	-2.9460757	down	-2.192794	down
THBS3	CR933610	thrombospondin 3	-2.1435308	down	-2.482915	down
LOC389199	XR_112616	uncharacterized LOC389199	-5.6941924	down	-5.2141843	down
ASIC3	NM_020322	acid-sensing (proton-gated) ion channel 3	-2.556778	down	-2.2609878	down
LOC285768		uncharacterized LOC285768	-2.9096715	down	-2.4689662	down
GOLGA2		golgin A2	-2.0631425	down	-2.2291768	down
LOC101060085	XM_003960901	uncharacterized LOC101060085	-4.2096252	down	-2.6206617	down
	AK123308		-3.471036	down	-2.0549405	down
SORCS3	NM_014978	sortilin-related VPS10 domain containing receptor 3	-4.1438384	down	-2.2625854	down
LOC100130051	AK127296	uncharacterized LOC100130051	-3.6706736	down	-2.4956615	down
OR2T5	NM_001004697	olfactory receptor, family 2, subfamily T, member 5	-4.3583646	down	-2.1197217	down
Unknown			-5.462452	down	-2.175076	down
LOC157273		uncharacterized LOC157273	-6.6521487	down	-2.7618835	down
	AK126938		-3.9056528	down	-2.921644	down
NNMT	NM_006169	nicotinamide N-methyltransferase	-9.808606	down	-13.12961	down
Unknown			-4.1117153	down	-2.3312879	down
LINC00310		long intergenic non-protein coding RNA 310	-4.5679903	down	-2.173869	down
LPHN1	NM_001008701	latrophilin 1	-2.548114	down	-2.3900933	down
TEX38	NM_001145474	testis expressed 38	-5.2272854	down	-4.49347	down
Unknown			-3.2390456	down	-3.5526977	down
DEFB134	NM_001033019	defensin, beta 134	-4.272501	down	-2.3584015	down
RETSAT		retinol saturase (all-trans-retinol 13,14-reductase)	-4.187894	down	-2.0712445	down
C22orf34	NR_026997	chromosome 22 open reading frame 34	-2.2686067	down	-3.4166331	down
FAM163B	NM_001080515	family with sequence similarity 163, member B	-3.0009964	down	-4.526807	down
Unknown			-4.2920394	down	-2.2805216	down
DEFB107A	NM_001037668	defensin, beta 107A	-5.8547006	down	-6.540771	down
EMP2	NM_001424	epithelial membrane protein 2	-4.497152	down	-3.9792604	down
Unknown			-3.6748657	down	-2.4281075	down
KLK13	NM_015596	kallikrein-related peptidase 13	-3.5179138	down	-2.421634	down
MOG	NM_206809	myelin oligodendrocyte glycoprotein	-3.3864996	down	-2.8477802	down
Unknown			-3.1846945	down	-2.42982	down
IQCF2	NM_203424	IQ motif containing F2	-3.8578835	down	-3.2830222	down
PCDHGB1	NM_032095	protocadherin gamma subfamily B, 1	-2.5671983	down	-2.044309	down
LOC100131742	AK130172	uncharacterized LOC100131742	-2.9540162	down	-2.585999	down
Unknown			-4.821494	down	-3.7484388	down
POTEM	NM_001145442	POTE ankyrin domain family, member M	-5.870577	down	-7.468435	down
FOXA3	NM_004497	forkhead box A3	-2.276451	down	-2.5469534	down
LINC00271	NR_026805	long intergenic non-protein coding RNA 271	-3.210521	down	-2.036642	down
Unknown			-2.701382	down	-3.0407228	down
Unknown			-3.7331333	down	-2.9879882	down
MYBPC1	NM_206819	myosin binding protein C, slow type	-4.8794165	down	-2.3334785	down
FLJ45482	AK127393	uncharacterized LOC645566	-2.1131496	down	-2.0237968	down
RAD21-AS1	NR_033886	RAD21 antisense RNA 1	-4.5330076	down	-2.5131156	down
Unknown			-5.920746	down	-3.9413996	down
Unknown			-7.209718	down	-5.029593	down
FAM213A	NM_001243780	family with sequence similarity 213, member A	-2.0932353	down	-2.2744062	down
CBX1	NM_006807	chromobox homolog 1	-5.542864	down	-2.1077194	down
NT5DC4	XM_001716359	5'-nucleotidase domain containing 4	-3.0654423	down	-2.1735709	down
CCNT1	NM_001240	cyclin T1	-4.919279	down	-2.7882442	down
KLK8	NM_144505	kallikrein-related peptidase 8	-3.3221169	down	-3.1755412	down
XLOC_014512			-3.3489199	down	-2.2669382	down
Unknown			-2.9625103	down	-3.81752	down
MAGIX	XM_005278064	MAGI family member, X-linked	-4.040417	down	-3.233026	down
Unknown			-5.8650217	down	-4.2233114	down
EDA	NM_001005610	ectodysplasin A	-3.2992418	down	-2.1785462	down

NAT8	NM_003960	N-acetyltransferase 8 (GCN5-related, putative)	-2.053412	down	-2.8716145	down
PTGER4P2-CDK2AP2P2	NR_024496	PTGER4P2-CDK2AP2P2 readthrough transcribed pseudogene	-3.012088	down	-2.1895514	down
SERPINI2	NM_006217	serpin peptidase inhibitor, clade I (pancpin), member 2	-2.15828	down	-2.0928302	down
HYKK	NM_001083612	hydroxylysine kinase	-2.6601794	down	-3.9076874	down
ZBED3-AS1	NR_024398	ZBED3 antisense RNA 1	-7.885624	down	-3.2932596	down
OR13A1	NM_001004297	olfactory receptor, family 13, subfamily A, member 1	-3.9673913	down	-2.099428	down
ADAMTS15	NM_139055	ADAM metallopeptidase with thrombospondin type 1 motif, 15	-4.1341434	down	-2.1439404	down
ZNF813	NM_001004301	zinc finger protein 813	-4.167519	down	-2.342992	down
ADARB1	NR_027673	adenosine deaminase, RNA-specific, B1	-2.712197	down	-2.1330729	down
LOC100233156	AK126241	tektin 4 pseudogene	-6.2541156	down	-3.8529954	down
	BC040680		-2.854412	down	-2.1774023	down
LINC00689		long intergenic non-protein coding RNA 689	-4.6481786	down	-2.2514052	down
PRKD1	NM_002742	protein kinase D1	-4.0513344	down	-3.865807	down
RAX2	NM_032753	retina and anterior neural fold homeobox 2	-4.2330184	down	-4.128754	down
ZNF169	NM_194320	zinc finger protein 169	-2.066752	down	-2.108995	down
A1CF	NM_138933	APOBEC1 complementation factor	-3.839784	down	-3.7475994	down
GIPC3	NM_133261	GIPC PDZ domain containing family, member 3	-5.3851786	down	-2.9935844	down
LOC642620	AK057320	uncharacterized LOC642620	-3.2882762	down	-3.0802457	down
LINC00618	NR_104113	long intergenic non-protein coding RNA 618	-2.7428224	down	-2.9055536	down
SLCO5A1	NM_030958	solute carrier organic anion transporter family, member 5A1	-4.9413896	down	-2.3006828	down
GPC1	NM_002081	glypican 1	-2.2144046	down	-2.2077737	down
HHIPL1	NM_032425	HHIP-like 1	-3.4155917	down	-2.093702	down
HECTD2	NM_182765	HECT domain containing E3 ubiquitin protein ligase 2	-4.4645815	down	-4.0942264	down
Unknown			-4.001242	down	-2.8668354	down
CSHL1	NM_022579	chorionic somatomammotropin hormone-like 1	-2.5057847	down	-2.6213083	down
FAM106A	NR_026809	family with sequence similarity 106, member A	-4.532388	down	-3.5205219	down
Unknown			-2.0774088	down	-2.9638157	down
	AK127893		-7.202477	down	-4.8544517	down
VTCN1	NR_045603	V-set domain containing T cell activation inhibitor 1	-3.9781182	down	-2.031538	down
LOC100132529	NR_103851	uncharacterized LOC100132529	-3.5951004	down	-2.9600005	down
LOC100127904	BC065739	uncharacterized LOC100127904	-3.549728	down	-5.4386525	down
LINC00544		long intergenic non-protein coding RNA 544	-4.11095	down	-2.5443115	down
Unknown	XR_243769		-2.3676298	down	-2.8649302	down
LOC728903	AK093722	uncharacterized LOC728903	-2.816711	down	-2.5341508	down
ZDHHC23	AK127260	zinc finger, DHHC-type containing 23	-6.642432	down	-6.0998616	down
Unknown	XM_005263792		-3.3495297	down	-3.1391382	down
TNFRSF6B	NM_003823	tumor necrosis factor receptor superfamily, member 6b, decoy	-2.6274188	down	-3.1570582	down
Unknown			-2.2161825	down	-2.4181159	down
PRSS16	NM_005865	protease, serine, 16 (thymus)	-9.22594	down	-11.002375	down
ABCG4	NM_022169	ATP-binding cassette, sub-family G (WHITE), member	-4.1571283	down	-2.0086153	down
PCED1B-AS1	NR_026544	PCED1B antisense RNA 1	-3.536466	down	-2.3936365	down
OR7E5P	NR_027688	olfactory receptor, family 7, subfamily E, member 5 pseudogene	-3.319051	down	-3.4216955	down
Unknown			-4.762558	down	-3.1926172	down
Unknown			-3.5872154	down	-2.2247903	down
VSIG10L	NM_001163922	V-set and immunoglobulin domain containing 10 like	-4.7419457	down	-2.73466	down
FAM57B	NM_031478	family with sequence similarity 57, member B	-2.1994233	down	-2.1494594	down
PHYHIP	NM_014759	phytanoyl-CoA 2-hydroxylase interacting protein	-5.250907	down	-3.9368682	down
FLJ42022	AK124016	uncharacterized LOC646748	-3.167998	down	-2.330798	down
C1QTNF1-AS1	NR_040018	C1QTNF1 antisense RNA 1	-3.9835076	down	-2.4516547	down
GOLGA6L4	NM_001267536	golgin A6 family-like 4	-4.712639	down	-2.619375	down
NACAD	NM_001146334	NAC alpha domain containing	-5.321976	down	-2.9412565	down
POTED	NM_174981	POTE ankyrin domain family, member D	-6.789891	down	-3.989508	down
LOC646652	XR_241774	integral membrane glycoprotein-like	-2.1505013	down	-3.532068	down
MTSS1L	NM_138383	metastasis suppressor 1-like	-3.1351573	down	-3.9568074	down
VAC14-AS1	NR_034083	VAC14 antisense RNA 1	-2.433133	down	-2.3949747	down
LGALS14	NM_203471	lectin, galactoside-binding, soluble, 14	-4.251584	down	-2.9478471	down
KRT34	NM_021013	keratin 34	-2.625701	down	-3.3958266	down
TLE6	NM_001143986	transducin-like enhancer of split 6 (E(sp1) homolog, Drosophila)	-4.5395117	down	-3.1661563	down
Unknown			-5.394148	down	-4.0448604	down

ENGASE	NM_001042573	endo-beta-N-acetylglucosaminidase	-3.6411772	down	-3.5386775	down
Unknown			-2.8715608	down	-2.2317536	down
HSPB3	NM_006308	heat shock 27kDa protein 3	-4.4834275	down	-2.325979	down
PGM5P2		phosphoglucomutase 5 pseudogene 2	-4.1240783	down	-2.2196925	down
RIPK4	NM_020639	receptor-interacting serine-threonine kinase 4	-2.4087222	down	-2.3816807	down
MMP21	NM_147191	matrix metallopeptidase 21	-3.395997	down	-2.1670947	down
NOL3	NM_003946	nucleolar protein 3 (apoptosis repressor with CARD domain)	-2.4400847	down	-2.2851138	down
REM1	NM_014012	RAS (RAD and GEM)-like GTP-binding 1	-4.25901	down	-2.496624	down
CALHM3	NM_001129742	calcium homeostasis modulator 3	-5.316171	down	-2.9816327	down
EPS8L1	NM_133180	EPS8-like 1	-2.4781256	down	-2.1542614	down
Unknown			-2.2233758	down	-2.0284348	down
Unknown			-6.7672424	down	-3.6973152	down
ANKRD20A11P		ankyrin repeat domain 20 family, member A11, pseudogene	-3.5555363	down	-3.0089655	down
NEDD4L	NM_001144967	neural precursor cell expressed, developmentally down-regulated 4-like, E3 ubiquitin protein ligase	-3.1037552	down	-2.0620732	down
	XR_247053		-2.2172968	down	-2.6088536	down
CHRNA4	NM_000744	cholinergic receptor, nicotinic, alpha 4 (neuronal)	-4.4841604	down	-2.7507045	down
Unknown			-2.018379	down	-2.6687324	down
Unknown			-9.651339	down	-6.2553062	down
	DA571569		-2.917901	down	-2.8202133	down
AMER3	NM_152698	APC membrane recruitment protein 3	-4.2442575	down	-2.7864103	down
PGA3	NM_001079807	pepsinogen 3, group I (pepsinogen A)	-2.0987616	down	-2.4115167	down
GRHL3	NM_198174	grainyhead-like 3 (Drosophila)	-3.3199573	down	-4.2577024	down
LOC100130872	NR_024569	uncharacterized LOC100130872	-2.0307987	down	-2.1362271	down
LINC00935	NR_029448	long intergenic non-protein coding RNA 935	-3.4732542	down	-2.5803335	down
IRX4	NM_016358	iroquois homeobox 4	-2.993381	down	-2.227684	down
SETD5-AS1	BC132680	SETD5 antisense RNA 1	-4.2791686	down	-5.0659294	down
LINC00483		long intergenic non-protein coding RNA 483	-4.7961707	down	-3.1257324	down
EIF4E1B	NM_001099408	eukaryotic translation initiation factor 4E family member 1B	-5.926982	down	-3.3814898	down
Unknown			-6.7825127	down	-3.352346	down
PDE8B	NM_003719	phosphodiesterase 8B	-2.2604287	down	-5.3174696	down
NRXN3	NM_004796	neurexin 3	-4.5228877	down	-2.1737566	down
LRRC73	NM_001012974	leucine rich repeat containing 73	-3.81703	down	-2.7284353	down
OR51A2	NM_001004748	olfactory receptor, family 51, subfamily A, member 2	-2.7020066	down	-2.214204	down
SLC16A10	NM_018593	solute carrier family 16 (aromatic amino acid transporter), member 10	-3.2704742	down	-2.1475823	down
Unknown			-3.946461	down	-2.2522726	down
CT47A11	NM_173571	cancer/testis antigen family 47, member A11	-6.4026427	down	-2.2315848	down
TBX3	NM_016569	T-box 3	-6.177232	down	-3.092287	down
LOC284454	NR_036515	uncharacterized LOC284454	-3.7287028	down	-2.3870957	down
SEC61A2	NM_018144	Sec61 alpha 2 subunit (S. cerevisiae)	-2.2156138	down	-2.6974995	down
COL8A1	NM_001850	collagen, type VIII, alpha 1	-4.2171135	down	-3.1609988	down
SYN3	NM_133633	synapsin III	-3.7954516	down	-2.006292	down
SLC22A25	NM_199352	solute carrier family 22, member 25	-7.330516	down	-2.47531	down
LOC149373	NM_001256615	uncharacterized LOC149373	-4.3129115	down	-2.069769	down
Unknown			-3.4046166	down	-2.5725393	down
Unknown			-2.4059758	down	-3.1031451	down
C19orf69	NM_001130514	chromosome 19 open reading frame 69	-4.7379704	down	-2.3431609	down
NFATC2IP	NM_032815	nuclear factor of activated T-cells, cytoplasmic, calcineurin-dependent 2 interacting protein	-3.1731696	down	-2.4465878	down
BTNL2	NM_019602	butyrophilin-like 2 (MHC class II associated)	-2.5240126	down	-2.7211523	down
HES3	NM_001024598	hes family bHLH transcription factor 3	-2.6068592	down	-2.6749797	down
UBD	NM_006398	ubiquitin D	-8.755354	down	-4.809574	down
OR7D4	NM_001005191	olfactory receptor, family 7, subfamily D, member 4	-5.6084228	down	-3.0526707	down
ENDOV	NM_173627	endonuclease V	-3.1270795	down	-3.2813058	down
Unknown			-2.1880362	down	-3.242217	down
S100B		S100 calcium binding protein B	-3.5035996	down	-2.3076413	down
TRIM46	NM_025058	tripartite motif containing 46	-3.149517	down	-3.551807	down
CACNA1C	NM_199460	calcium channel, voltage-dependent, L type, alpha 1C subunit	-5.3734813	down	-2.6821473	down
PRMT8	NM_019854	protein arginine methyltransferase 8	-2.8533008	down	-2.2988193	down
MGAT4C	NM_013244	mannosyl (alpha-1,3-)-glycoprotein beta-1,4-N-acetylglucosaminyltransferase, isozyme C (putative)	-4.2813425	down	-2.0604637	down

RBFOX2	NM_001031695	RNA binding protein, fox-1 homolog (C. elegans) 2	-9.846267	down	-6.778945	down
TMEM37	XM_005263597	transmembrane protein 37	-2.3031223	down	-2.0115738	down
SLC22A2	NM_003058	solute carrier family 22 (organic cation transporter), member 2	-2.4066129	down	-2.0932717	down
ANKRD20A12P	NR_046228	ankyrin repeat domain 20 family, member A12, pseudogene	-5.7210774	down	-6.1331234	down
XLOC_014422			-2.561916	down	-2.7846134	down
CXorf28	NR_038428	chromosome X open reading frame 28	-2.8776813	down	-2.0084805	down
Unknown			-5.006759	down	-2.9862378	down
LOC100129775	AK094853	uncharacterized LOC100129775	-3.4907112	down	-3.8658996	down
MGC4859		uncharacterized LOC79150	-3.601921	down	-2.8889797	down
SENP1	NM_001267595	SUMO1/sentrin specific peptidase 1	-2.0336473	down	-2.44317	down
CRB2	NM_173689	crumbs homolog 2 (Drosophila)	-3.942032	down	-3.0469072	down
BPIFA3	NM_178466	BPI fold containing family A, member 3	-3.0791116	down	-2.1466963	down
SCN5A	NM_001099404	sodium channel, voltage-gated, type V, alpha subunit	-3.0009327	down	-2.1593676	down
LOC283856		uncharacterized LOC283856	-4.671689	down	-3.9197576	down
PARVB	AK309987	parvin, beta	-3.1674705	down	-2.1484697	down
	AK130794		-4.6924896	down	-2.8214889	down
Unknown			-2.339011	down	-2.4623194	down
KCNJ6	NM_002240	potassium inwardly-rectifying channel, subfamily J, member 6	-3.730596	down	-2.2140284	down
FPGT-TNNI3K	BX640903	FPGT-TNNI3K readthrough	-2.702092	down	-3.0323894	down
AK7	NM_152327	adenylate kinase 7	-2.9305077	down	-2.3729737	down
	AK097143		-3.3003173	down	-2.0021162	down
LOC202181	NR_026921	SUMO-interacting motifs containing 1 pseudogene	-5.4216523	down	-6.883093	down
Unknown			-6.3917685	down	-4.0024467	down
ADAMTSL1	NM_052866	ADAMTS-like 1	-3.6237416	down	-2.2870672	down
VWA5B1	AK125833	von Willebrand factor A domain containing 5B1	-5.025445	down	-4.0214868	down
HAS3	NM_138612	hyaluronan synthase 3	-4.259085	down	-2.525828	down
SNX30	NM_001012994	sorting nexin family member 30	-2.8072846	down	-2.224706	down
Unknown			-2.3061244	down	-3.3599677	down
NKPD1	NM_198478	NTPase, KAP family P-loop domain containing 1	-3.886222	down	-4.0329967	down
P4HA3	NM_182904	prolyl 4-hydroxylase, alpha polypeptide III	-2.8523128	down	-2.103879	down
SLC26A9	NM_052934	solute carrier family 26 (anion exchanger), member 9	-3.2993114	down	-2.0560274	down
DEFB128	NM_001037732	defensin, beta 128	-2.5016863	down	-2.638117	down
SPTSSB	XM_005247164	serine palmitoyltransferase, small subunit B	-5.6634626	down	-2.507794	down
Unknown			-3.7881436	down	-4.0071855	down
	BF106382		-3.0622535	down	-2.3401406	down
TPSD1	NM_012217	tryptase delta 1	-3.3138595	down	-2.5900114	down
ETV3	NM_001145312	ets variant 3	-4.211457	down	-2.7701516	down
LOC101928058	XM_005276314	myosin IC heavy chain-like	-2.5324035	down	-2.776681	down
ZNF774	NM_001004309	zinc finger protein 774	-3.901621	down	-2.1446857	down
METTL21A	NM_001127395	methyltransferase like 21A	-5.2700067	down	-3.6845732	down
	AK123993		-3.710725	down	-2.828009	down
DGCR9		DiGeorge syndrome critical region gene 9	-4.6057434	down	-2.7433918	down
MUC5B	NM_002458	mucin 5B, oligomeric mucus/gel-forming	-10.756107	down	-6.858593	down
HERC2P4		hect domain and RLD 2 pseudogene 4	-6.269283	down	-3.6758735	down
LINC00969	XR_110395	long intergenic non-protein coding RNA 969	-2.264478	down	-2.7566223	down
PRAMEF2	NM_023014	PRAME family member 2	-4.333282	down	-2.755429	down
GCLM	NM_002061	glutamate-cysteine ligase, modifier subunit	-2.7818973	down	-2.3291495	down
Unknown			-7.0010934	down	-5.762676	down
SLC35A4	AF445025	solute carrier family 35, member A4	-3.9913979	down	-2.3749065	down
TBCD	AL096745	tubulin folding cofactor D	-2.2289119	down	-3.2921207	down
	AK000470		-2.7995682	down	-2.1033428	down
Unknown			-3.496711	down	-2.5059135	down
RAB7B	NM_177403	RAB7B, member RAS oncogene family	-2.3200176	down	-2.7598479	down
Unknown			-2.8396058	down	-2.2084846	down
XRCC2	NM_005431	X-ray repair complementing defective repair in Chinese hamster cells 2	-4.578571	down	-3.9412048	down
SP6	NM_001258248	Sp6 transcription factor	-4.552489	down	-2.0697722	down
ALX1	NM_006982	ALX homeobox 1	-4.8283577	down	-2.365797	down
C10orf71	NM_001135196	chromosome 10 open reading frame 71	-4.1339903	down	-2.0601904	down
EMC1		ER membrane protein complex subunit 1	-3.1584673	down	-4.023962	down
GGT6	NM_153338	gamma-glutamyltransferase 6	-10.755072	down	-5.5791426	down

NSAP11	AY176665	nervous system abundant protein 11	-4.7356954	down	-2.004151	down
MASP2	NM_006610	mannan-binding lectin serine peptidase 2	-3.6281624	down	-3.7483637	down
	BX108667		-3.3677077	down	-2.2276397	down
KIAA0087	NR_022006	KIAA0087	-4.918724	down	-2.3426142	down
LOC283693	NR_036446	actin, alpha 2, smooth muscle, aorta pseudogene	-2.600257	down	-2.2174149	down
Unknown			-5.801206	down	-3.9314988	down
TTC7A	NM_020458	tetratricopeptide repeat domain 7A	-2.2556286	down	-2.294366	down
Unknown			-5.349309	down	-2.5119946	down
BGN	NM_001711	biglycan	-7.392654	down	-5.2434115	down
PADI3	NM_016233	peptidyl arginine deiminase, type III	-4.1503663	down	-2.3341827	down
C16orf95	AK125749	chromosome 16 open reading frame 95	-4.60003	down	-2.8895736	down
SIGLEC6	NM_001245	sialic acid binding Ig-like lectin 6	-3.5347624	down	-5.1416054	down
FAM92A1P2	NR_003612	family with sequence similarity 92, member A3	-3.867607	down	-5.862625	down
Unknown			-5.5634627	down	-3.0058002	down
LINC00277	NR_026949	long intergenic non-protein coding RNA 277	-4.986363	down	-2.0005004	down
CACNA1G	NM_018896	calcium channel, voltage-dependent, T type, alpha 1G subunit	-3.597668	down	-2.7417939	down
C1orf21	NM_030806	chromosome 1 open reading frame 21	-2.2572765	down	-3.111706	down
	AF116661		-4.683552	down	-2.8661096	down
KIAA1161	NM_020702	KIAA1161	-2.454754	down	-2.7705297	down
Unknown			-3.0757117	down	-2.3838866	down
Unknown			-3.3601573	down	-2.8170528	down
C1QL2	NM_182528	complement component 1, q subcomponent-like 2	-4.7173605	down	-4.247638	down
LOC100996379	XR_248076	uncharacterized LOC100996379	-2.7734199	down	-2.542685	down
Unknown			-5.515265	down	-3.347469	down
Unknown			-2.7698796	down	-2.7305565	down
ILDR1	NM_175924	immunoglobulin-like domain containing receptor 1	-3.5082798	down	-2.6286054	down
GAFA1	AF190748	FGF2-associated protein GAFA1	-3.319671	down	-2.0848186	down
LOC100499221	AK127052	uncharacterized LOC100499221	-5.324082	down	-4.90141	down
HERC2	NM_004667	HECT and RLD domain containing E3 ubiquitin protein ligase 2	-2.75267	down	-2.2911088	down
VHL	NM_000551	von Hippel-Lindau tumor suppressor, E3 ubiquitin protein ligase	-4.829094	down	-3.0923693	down
GRIA1	NM_000827	glutamate receptor, ionotropic, AMPA 1	-4.5990267	down	-2.0847723	down
Unknown			-4.894907	down	-2.5602794	down
RGL1	NM_015149	ral guanine nucleotide dissociation stimulator-like 1	-6.9861145	down	-5.1065435	down
MPV17L	NM_173803	MPV17 mitochondrial membrane protein-like	-4.1107717	down	-3.6402934	down
	BC014063		-4.8932796	down	-3.6603844	down
NRIP3	NM_020645	nuclear receptor interacting protein 3	-3.287184	down	-2.0406232	down
Unknown			-3.3846116	down	-2.2177672	down
Unknown			-4.2624636	down	-3.3741927	down
LOC100996465	XM_003846784	putative lung carcinoma-associated protein 10-like	-4.5345583	down	-2.4019191	down
NTF3	NM_002527	neurotrophin 3	-4.462657	down	-2.3882217	down
OR10A6	NM_001004461	olfactory receptor, family 10, subfamily A, member 6	-5.114049	down	-3.0233197	down
LOC284219	AK094436	uncharacterized LOC284219	-10.057078	down	-6.937428	down
CCDC9	NM_015603	coiled-coil domain containing 9	-2.0740407	down	-2.431223	down
OR4N4	NM_001005241	olfactory receptor, family 4, subfamily N, member 4	-6.6908693	down	-4.0046897	down
DNM1P46	NR_003260	DNM1 pseudogene 46	-4.1590767	down	-2.9753864	down
PDCL2	NM_152401	phosducin-like 2	-2.151772	down	-2.1179345	down
KIAA1751	NM_001080484	KIAA1751	-3.5649388	down	-2.0209827	down
XLOC_014512			-2.6693015	down	-5.814292	down
E2F7	NM_203394	E2F transcription factor 7	-3.502249	down	-2.8482833	down
FAM181B	NM_175885	family with sequence similarity 181, member B	-2.8712947	down	-2.804772	down
AZFP	AY371499	AML-associated zinc finger protein	-4.4405746	down	-2.305171	down
MDH1B	NR_104261	malate dehydrogenase 1B, NAD (soluble)	-3.5608828	down	-4.026212	down
PGAM5	NM_138575	phosphoglycerate mutase family member 5	-2.3490632	down	-2.82386	down
XLOC_014512			-8.03557	down	-4.8995767	down
LOC729609		uncharacterized LOC729609	-3.7811809	down	-2.7213964	down
DHRS4-AS1	NR_023921	DHRS4 antisense RNA 1	-3.5561388	down	-3.4821274	down
GNG12	NM_018841	guanine nucleotide binding protein (G protein), gamma 12	-3.0714355	down	-2.5622551	down
FAM86B3P		family with sequence similarity 86, member A pseudogene	-3.6985886	down	-3.8406982	down
ERVW-1	NM_014590	endogenous retrovirus group W, member 1	-5.1962247	down	-4.2838273	down
FAM201B	XR_158980	family with sequence similarity 201, member B	-4.2108583	down	-4.605173	down

Unknown			-6.3695407	down	-5.1520066	down
Unknown			-4.2780185	down	-3.4537048	down
ARHGAP42	NM_152432	Rho GTPase activating protein 42	-3.8138444	down	-2.7685244	down
LINC01101	NR_027181	long intergenic non-protein coding RNA 1101	-2.2022698	down	-2.1107287	down
C8orf74	NM_001040032	chromosome 8 open reading frame 74	-7.1475515	down	-3.8976905	down
LOC100507547	NR_037169	uncharacterized LOC100507547	-2.9369998	down	-3.1023297	down
CPLX2	NM_006650	complexin 2	-5.9213643	down	-3.2364979	down
PRODH2	NM_021232	proline dehydrogenase (oxidase) 2	-5.200948	down	-2.0751872	down
CD86	NM_006889	CD86 molecule	-5.824963	down	-6.0708394	down
SCGB1B2P		secretoglobin, family 1B, member 2, pseudogene	-3.4354215	down	-2.312933	down
TBX4	NM_018488	T-box 4	-3.6303208	down	-2.0030367	down
FAM19A4	NM_182522	family with sequence similarity 19 (chemokine (C-C motif)-like), member A4	-4.174579	down	-2.1570458	down
POU4F3	NM_002700	POU class 4 homeobox 3	-3.7735963	down	-2.1781178	down
	BC007749		-6.975558	down	-4.605416	down
	XM_005249927		-3.7251582	down	-4.0899434	down
AMZ1	NM_133463	archaelysin family metallopeptidase 1	-3.3645036	down	-3.0072765	down
PWAR4	NR_022010	Prader Willi/Angelman region RNA 4	-9.453847	down	-7.4043956	down
ALDH1L2	NM_001034173	aldehyde dehydrogenase 1 family, member L2	-2.764571	down	-2.5476327	down
Unknown			-9.498351	down	-21.25616	down
ADAMTS4	BC030812	ADAM metallopeptidase with thrombospondin type 1 motif, 4	-4.3887296	down	-2.774159	down
VPS18	NM_020857	vacuolar protein sorting 18 homolog (S. cerevisiae)	-2.131899	down	-2.7445157	down
GRIA4	NM_001077243	glutamate receptor, ionotropic, AMPA 4	-2.7662363	down	-2.414727	down
FER1L5	NM_001113382	fer-1-like 5 (C. elegans)	-2.756342	down	-2.0715592	down
ZNF852	XM_001717544	zinc finger protein 852	-2.3718178	down	-2.7356296	down
RND1	NM_014470	Rho family GTPase 1	-3.1681838	down	-2.352077	down
TCEB3B	NM_016427	transcription elongation factor B polypeptide 3B (elongin A2)	-5.5670867	down	-3.5261405	down
C7orf10	NM_024728	chromosome 7 open reading frame 10	-3.4909778	down	-2.821668	down
PROSER2	NM_153256	proline and serine-rich protein 2	-2.7859175	down	-2.5315373	down
Unknown			-4.8965826	down	-3.031054	down
BNIP3L	AF370457	BCL2/adenovirus E1B 19kDa interacting protein 3-like	-3.117538	down	-2.8183804	down
FAM222A-AS1	NR_026661	FAM222A antisense RNA 1	-3.2355976	down	-2.1342783	down
	AK122833		-4.0080695	down	-2.378262	down
MCC	NM_001085377	mutated in colorectal cancers	-6.3913565	down	-2.6274388	down
C12orf80	NM_001242696	chromosome 12 open reading frame 80	-2.9636812	down	-2.4312103	down
IFFO1	NR_036467	intermediate filament family orphan 1	-3.801478	down	-2.5381324	down
Unknown			-6.240865	down	-2.6310046	down
	BX537909		-2.7614732	down	-2.0076368	down
KCNB2	NM_004770	potassium voltage-gated channel, Shab-related subfamily, member 2	-5.579362	down	-2.4696224	down
BMPER	NM_133468	BMP binding endothelial regulator	-4.3178067	down	-2.4310462	down
Unknown			-3.7011566	down	-3.5182805	down
LOC400553	XR_111612	uncharacterized LOC400553	-5.2414927	down	-5.910486	down
NUTM2F	NM_017561	NUT family member 2F	-3.4455528	down	-2.226387	down
			-2.7774398	down	-2.2071064	down
DNMT3A	NM_175629	DNA (cytosine-5-)-methyltransferase 3 alpha	-2.0813985	down	-2.0584514	down
SPAG17	NM_206996	sperm associated antigen 17	-3.2783108	down	-2.1135762	down
ARHGAP21	BX537570	Rho GTPase activating protein 21	-3.1821902	down	-2.3107944	down
Unknown			-2.4048257	down	-2.2358017	down
LOC100131066	AK126015	uncharacterized LOC100131066	-5.14673	down	-2.6286132	down
	XR_248227		-4.2758546	down	-2.2441561	down
SERPINB7	NM_001040147	serpin peptidase inhibitor, clade B (ovalbumin), member 7	-3.9667935	down	-2.9745972	down
GABRB1	AK296023	gamma-aminobutyric acid (GABA) A receptor, beta 1	-2.4437363	down	-2.3036993	down
	AK127846		-2.835314	down	-3.3377197	down
	XR_247266		-3.9648254	down	-2.3169346	down
LAMC2	NM_005562	laminin, gamma 2	-2.598634	down	-2.3459158	down
YWHAE	AK296555	tyrosine 3-monooxygenase/tryptophan 5-monooxygenase activation protein, epsilon polypeptide	-4.3042426	down	-2.908293	down
LOC100507547	NR_037169	uncharacterized LOC100507547	-3.0497594	down	-2.4733112	down
MAP3K6	NM_004672	mitogen-activated protein kinase kinase kinase 6	-2.1583643	down	-2.601848	down
LOC728158	XR_246201	hCG2044975	-4.0380673	down	-2.263453	down
C20orf85	NM_178456	chromosome 20 open reading frame 85	-3.6901999	down	-2.8147066	down

Unknown			-3.8055658	down	-3.5980184	down
PER2	NM_022817	period circadian clock 2	-2.111167	down	-2.9666555	down
CDH4	NM_001794	cadherin 4, type 1, R-cadherin (retinal)	-5.5603046	down	-6.0878124	down
02-mar	NM_017898	mitochondrial amidoxime reducing component 2	-2.674826	down	-2.0407164	down
CDH2	NM_001792	cadherin 2, type 1, N-cadherin (neuronal)	-4.583503	down	-3.714384	down
C9orf131	NM_203299	chromosome 9 open reading frame 131	-2.0848014	down	-2.3778877	down
	BC010535		-2.0297146	down	-2.4203024	down
GH2	NM_022558	growth hormone 2	-3.3913367	down	-2.3368669	down
MYO1C	NM_033375	myosin IC	-2.2141192	down	-2.173801	down
GTF2IRD2B	NM_001003795	GTF2I repeat domain containing 2B	-2.1344588	down	-2.7550595	down
LOC101059906	XM_003959933	collagen alpha-1(XXVII) chain-like	-2.3530664	down	-2.4644034	down
BICC1	NM_001080512	bicaudal C homolog 1 (Drosophila)	-4.6057963	down	-3.3942635	down
HFE2	NM_213653	hemochromatosis type 2 (juvenile)	-3.113686	down	-2.420331	down
PRDM15	NM_001282934	PR domain containing 15	-2.801667	down	-2.3053489	down
ENTPD8	NM_001033113	ectonucleoside triphosphate diphosphohydrolase 8	-6.5482965	down	-3.736031	down
TECPR2	NM_001172631	tectonin beta-propeller repeat containing 2	-4.199432	down	-2.1858718	down
Unknown			-8.630097	down	-3.782386	down
Unknown			-4.741531	down	-4.886796	down
LOC284581	AK094426	uncharacterized LOC284581	-14.301185	down	-9.800617	down
THNSL2	NM_001244676	threonine synthase-like 2 (S. cerevisiae)	-4.398632	down	-3.135761	down
SPACA1	NM_030960	sperm acrosome associated 1	-3.7234159	down	-3.5610242	down
MAD2L2		MAD2 mitotic arrest deficient-like 2 (yeast)	-3.8341029	down	-3.2875857	down
FAM27A	NR_024060	family with sequence similarity 27, member A	-2.543885	down	-2.4772813	down
FUT11	NM_001284194	fucosyltransferase 11 (alpha (1,3) fucosyltransferase)	-2.0877993	down	-2.0068507	down
Unknown			-2.967473	down	-2.789328	down
Unknown			-5.7256427	down	-2.1246498	down
	AK021797		-4.1011076	down	-2.5752614	down
FAM3A	NM_001171134	family with sequence similarity 3, member A	-2.1458092	down	-2.1456265	down
ARHGEF35	NM_001003702	Rho guanine nucleotide exchange factor (GEF) 35	-5.7877855	down	-2.4864452	down
NS3BP	NR_046316	NS3BP	-2.9254448	down	-2.2801344	down
HELT	NM_001029887	helt bHLH transcription factor	-3.549902	down	-2.0148091	down
C4orf19	NM_018302	chromosome 4 open reading frame 19	-2.7179587	down	-2.4065623	down
POU6F2	NM_007252	POU class 6 homeobox 2	-3.8951123	down	-2.414026	down
FAM95B1		family with sequence similarity 95, member B1	-3.8343315	down	-2.5860279	down
LOC63930		uncharacterized LOC63930	-3.9411638	down	-2.8614426	down
BMP8A	NM_181809	bone morphogenetic protein 8a	-4.3716955	down	-2.676179	down
LINC00301	NR_026946	long intergenic non-protein coding RNA 301	-8.623594	down	-4.916239	down
HIST3H2A	NM_033445	histone cluster 3, H2a	-2.01784	down	-2.0041134	down
COL6A5	NM_153264	collagen, type VI, alpha 5	-2.608723	down	-2.7317326	down
ZNF704	NM_001033723	zinc finger protein 704	-6.9241543	down	-3.5323913	down
LOC727982	NR_034134	uncharacterized LOC727982	-2.338986	down	-2.262424	down
			-4.768012	down	-3.0192158	down
			-3.514974	down	-3.394756	down
MAGEE2	NM_138703	melanoma antigen family E, 2	-3.6469004	down	-2.00022	down
EFCAB10	NR_027068	EF-hand calcium binding domain 10	-3.6122298	down	-2.418079	down
FAM41C	NR_027055	family with sequence similarity 41, member C	-5.4968333	down	-3.758045	down
XLOC_014512			-4.5733705	down	-3.4781518	down
CLDN14	NM_144492	claudin 14	-8.394292	down	-3.1466837	down
TMEM179	NM_001286390	transmembrane protein 179	-4.006581	down	-2.9599962	down
KCTD3	NM_016121	potassium channel tetramerization domain containing 3	-3.1514747	down	-2.715921	down
PROKR1	NM_138964	prokineticin receptor 1	-4.421828	down	-2.236533	down
			-7.5295234	down	-2.791482	down
			-3.4022899	down	-2.2949421	down
FLJ22763	NR_033977	uncharacterized LOC401081	-4.0255485	down	-2.2647824	down
			-4.2093396	down	-2.5410364	down
TMEM184A	NM_001097620	transmembrane protein 184A	-3.654196	down	-3.2429647	down
FERMT1	NM_017671	fermitin family member 1	-3.6510782	down	-2.7994456	down
C14orf64		chromosome 14 open reading frame 64	-3.591492	down	-2.30505	down
PRKAR1B	NM_001164761	protein kinase, cAMP-dependent, regulatory, type I, beta	-2.594742	down	-2.5395684	down
			-2.0164742	down	-3.3423624	down
TNFRSF19	BC035796	tumor necrosis factor receptor superfamily, member 19	-5.3742495	down	-6.1958547	down
SYT2	NM_001136504	synaptotagmin II	-3.6701791	down	-3.063535	down

SLC15A1	AB001328	solute carrier family 15 (oligopeptide transporter), member 1	-4.0305743	down	-2.1222937	down
HIST1H2BE	NM_003523	histone cluster 1, H2be	-2.5931947	down	-2.0254223	down
MANEA	NM_024641	mannosidase, endo-alpha	-3.0877209	down	-2.4143198	down
LOC100128591	AK128705	uncharacterized LOC100128591	-3.759605	down	-3.0677636	down
VRTN	NM_018228	vertebrae development associated	-5.439992	down	-3.349385	down
FAM149A	NM_015398	family with sequence similarity 149, member A	-3.5005147	down	-2.9712298	down
FUBP3	NM_003934	far upstream element (FUSE) binding protein 3	-5.2330184	down	-2.7376785	down
HSD11B2	NM_000196	hydroxysteroid (11-beta) dehydrogenase 2	-3.596185	down	-2.1148684	down
NLGN4X	NM_001282145	neuroligin 4, X-linked	-3.998283	down	-2.2987318	down
TLCD2	NM_001164407	TLC domain containing 2	-5.3781314	down	-2.198005	down
LOC284950	NR_038888	uncharacterized LOC284950	-6.351751	down	-2.9387305	down
SCRT2	NM_033129	scratch family zinc finger 2	-4.027731	down	-2.2713118	down
			-2.1838965	down	-4.360089	down
ANGPTL2	NM_012098	angiopoietin-like 2	-4.095893	down	-2.3287864	down
	XM_001716650		-2.5670235	down	-2.132765	down
KCNK4	NM_033310	potassium channel, subfamily K, member 4	-2.068595	down	-2.363637	down
LOC389831	NM_001242480	uncharacterized LOC389831	-3.2388964	down	-3.4519672	down
	AL833920		-3.602652	down	-2.6940622	down
C14orf178	NM_174943	chromosome 14 open reading frame 178	-4.598357	down	-2.2258563	down
PDE4DIP	NM_022359	phosphodiesterase 4D interacting protein	-5.818242	down	-3.0068479	down
KRTAP10-5	NM_198694	keratin associated protein 10-5	-4.0719576	down	-3.916554	down
MGP	NM_000900	matrix Gla protein	-2.911317	down	-2.1918366	down
KRTAP3-3	NM_033185	keratin associated protein 3-3	-4.4346056	down	-2.7511568	down
LOC100129785	AK125285	uncharacterized LOC100129785	-8.948574	down	-7.7528396	down
SUV420H2	AK304562	suppressor of variegation 4-20 homolog 2 (Drosophila)	-4.5760546	down	-2.475215	down
ZC3H12D	NM_207360	zinc finger CCCH-type containing 12D	-5.7286477	down	-3.0985065	down
			-5.549519	down	-2.541297	down
			-2.9380908	down	-2.0397563	down
ST3GAL4	AK021929	ST3 beta-galactoside alpha-2,3-sialyltransferase 4	-4.5439243	down	-7.044811	down
			-2.9376445	down	-2.4711325	down
			-2.830846	down	-2.2931592	down
			-2.7486212	down	-3.1073565	down
			-2.8975022	down	-2.364695	down
SLC30A2	NM_001004434	solute carrier family 30 (zinc transporter), member 2	-2.344598	down	-2.9452925	down
SLC24A1	NM_004727	solute carrier family 24 (sodium/potassium/calcium exchanger), member 1	-3.9151382	down	-2.394274	down
ZNF468	NM_001277120	zinc finger protein 468	-2.827076	down	-2.8107257	down
CATSPERD	AK128088	catsper channel auxiliary subunit delta	-4.623141	down	-3.2647536	down
ARHGEF17	NM_014786	Rho guanine nucleotide exchange factor (GEF) 17	-2.4432623	down	-2.1165524	down
			-4.3227043	down	-2.2147217	down
HMGCS2	NM_005518	3-hydroxy-3-methylglutaryl-CoA synthase 2 (mitochondrial)	-2.4938753	down	-2.0311007	down
GRXCR2	NM_001080516	glutaredoxin, cysteine rich 2	-4.0122957	down	-2.2865386	down
ONECUT1	NM_004498	one cut homeobox 1	-4.9073195	down	-2.0443244	down
OR13D1	NM_001004484	olfactory receptor, family 13, subfamily D, member 1	-2.4869776	down	-2.1143165	down
OR51I1	NM_001005288	olfactory receptor, family 51, subfamily I, member 1	-5.992391	down	-3.1008372	down
KCNE4	NM_080671	potassium voltage-gated channel, Isk-related family, member 4	-3.4151103	down	-5.6199284	down
	U66047		-7.5220237	down	-4.487957	down
FGFR3	NM_000142	fibroblast growth factor receptor 3	-2.0702164	down	-3.825862	down
AFAP1-AS1	NR_026892	AFAP1 antisense RNA 1	-3.780862	down	-2.1451848	down
ACTRT3	NM_032487	actin-related protein T3	-4.7366433	down	-3.146077	down
			-3.2747273	down	-2.0443058	down
			-2.1157718	down	-2.325933	down
FLJ46066		uncharacterized LOC401103	-4.485975	down	-2.317953	down
			-2.4015107	down	-2.5467656	down
DHRS2	NM_182908	dehydrogenase/reductase (SDR family) member 2	-6.8590326	down	-3.0704036	down
REEP6	NM_138393	receptor accessory protein 6	-2.575719	down	-2.875117	down
RCOR2	NM_173587	REST corepressor 2	-4.64733	down	-3.462535	down
	AK025061		-8.363573	down	-4.825887	down
FAT3	NM_001008781	FAT atypical cadherin 3	-4.0956755	down	-2.8852012	down
KRT222	NM_152349	keratin 222	-7.3488154	down	-5.3459196	down
			-4.4342446	down	-3.6658337	down

SCN9A	NM_002977	sodium channel, voltage-gated, type IX, alpha subunit	-5.770431	down	-3.8044028	down
PROM2	NM_001165978	prominin 2	-9.506657	down	-7.005659	down
SRCIN1	NM_025248	SRC kinase signaling inhibitor 1	-3.8380826	down	-4.1044054	down
PIP	NM_002652	prolactin-induced protein	-10.857858	down	-7.557286	down
KRTAP9-1	NM_001190460	keratin associated protein 9-1	-3.4712448	down	-2.895403	down
EVA1A	NM_032181	eva-1 homolog A (C. elegans)	-2.7754946	down	-2.3179898	down
PCDHGA3	NM_032011	protocadherin gamma subfamily A, 3	-3.5279963	down	-2.0073564	down
SHANK1	NM_016148	SH3 and multiple ankyrin repeat domains 1	-5.3425636	down	-4.383421	down
STGC3	AY078383	uncharacterized STGC3	-3.1621144	down	-2.004961	down
LOC101928841	XM_005275740	collagen alpha-1(II) chain-like	-3.6128876	down	-3.0595014	down
LOC100130452	NR_034036	uncharacterized LOC100130452	-3.787305	down	-2.2544987	down
NR2F1	NM_005654	nuclear receptor subfamily 2, group F, member 1	-3.382628	down	-2.361522	down
ARHGAP39	NM_025251	Rho GTPase activating protein 39	-5.1775594	down	-2.9829621	down
C3orf84	NM_001080528	chromosome 3 open reading frame 84	-5.4438753	down	-3.8746393	down
C10orf115	NR_103721	chromosome 10 open reading frame 115	-3.7585018	down	-2.6245832	down
SNORA70B	BQ029156	small nucleolar RNA, H/ACA box 70B	-2.2360396	down	-2.2352493	down
	AK128215		-2.3471217	down	-2.1363661	down
	BX093647		-3.8331578	down	-2.2324064	down
OR2T4	NM_001004696	olfactory receptor, family 2, subfamily T, member 4	-4.949648	down	-2.253095	down
TBC1D28	NM_001039397	TBC1 domain family, member 28	-3.4437764	down	-2.87844	down
FAM212A	NM_203370	family with sequence similarity 212, member A	-2.2688856	down	-2.4272845	down
LOC100129048	AK128074	uncharacterized LOC100129048	-4.081966	down	-2.5689895	down
SSX4B	NM_001034832	synovial sarcoma, X breakpoint 4B	-4.847469	down	-4.047795	down
RNF150	NM_020724	ring finger protein 150	-8.132386	down	-5.1244707	down
LOC647983	AK126828	uncharacterized LOC647983	-3.9270349	down	-4.8871355	down
GOLGA2P2Y	NR_001555	golgin A2 pseudogene 2, Y-linked	-3.5396156	down	-2.5211184	down
LINC00314	NR_027246	long intergenic non-protein coding RNA 314	-3.850291	down	-2.2244236	down
LRRC46	NM_033413	leucine rich repeat containing 46	-2.1864002	down	-2.0385394	down
DGCR10	NR_026651	DiGeorge syndrome critical region gene 10 (non-protein coding)	-7.111806	down	-3.6058676	down
	AK094155		-4.5506506	down	-5.0520654	down
LENG9	NM_198988	leukocyte receptor cluster (LRC) member 9	-3.007068	down	-2.007832	down
C16orf3	NM_001214	chromosome 16 open reading frame 3	-7.0938406	down	-5.961076	down
UMODL1	NM_001199528	uromodulin-like 1	-3.8491638	down	-4.0061846	down
PCDHB8	NM_019120	protocadherin beta 8	-4.534865	down	-2.515205	down
SIX3	NM_005413	SIX homeobox 3	-4.537231	down	-3.9609032	down
AGFG2	NM_006076	ArfGAP with FG repeats 2	-2.2483902	down	-2.0649567	down
OR2AT4	NM_001005285	olfactory receptor, family 2, subfamily AT, member 4	-5.2183795	down	-3.5410466	down
SEC24A	NM_001252231	SEC24 family member A	-2.5152516	down	-2.012962	down
LRRC19	NM_022901	leucine rich repeat containing 19	-6.2985168	down	-3.5081592	down
TSSK1B	NM_032028	testis-specific serine kinase 1B	-5.448901	down	-2.1378126	down
LOC101930210	XR_246852	protein capicua homolog	-5.429978	down	-2.7558503	down
ARMS2	NM_001099667	age-related maculopathy susceptibility 2	-3.8944674	down	-2.514969	down
PAX2	NM_003990	paired box 2	-5.113308	down	-4.706431	down
LOC284014	AK095567	uncharacterized LOC284014	-5.84957	down	-3.6832788	down
			-3.2407732	down	-3.682595	down
			-4.3227715	down	-3.0128133	down
INMT	NM_001199219	indolethylamine N-methyltransferase	-5.000981	down	-4.5414267	down
STRC	NM_153700	stereocilin	-3.7172263	down	-2.3872378	down
XLOC_014512			-5.1569023	down	-2.4237964	down
			-2.342658	down	-2.1300905	down
C7orf61	NM_001004323	chromosome 7 open reading frame 61	-3.8604336	down	-4.073289	down
RNASET2	AK124363	ribonuclease T2	-5.3448896	down	-5.269457	down
HELZ2	BC104974	helicase with zinc finger 2, transcriptional coactivator	-2.06229	down	-2.3447983	down
GSN-AS1	NR_103560	GSN antisense RNA 1	-6.0970016	down	-2.933544	down
LOC339539		uncharacterized LOC339539	-5.9084177	down	-2.9785721	down
			-3.372966	down	-2.9898376	down
OR7G2	NM_001005193	olfactory receptor, family 7, subfamily G, member 2	-3.580863	down	-2.5025396	down
PAPPA	NM_002581	pregnancy-associated plasma protein A, pappalysin 1	-5.3291507	down	-2.2928572	down
KRTAP19-1	NM_181607	keratin associated protein 19-1	-6.35791	down	-6.9071665	down
	BQ719082		-2.2931893	down	-3.9675622	down
			-3.1420896	down	-2.0686378	down
			-4.8877363	down	-2.0024207	down

MAGEC2	NM_016249	melanoma antigen family C, 2	-2.90552	down	-3.9414883	down
ZNF248	BC021819	zinc finger protein 248	-3.8065662	down	-2.340402	down
SULT4A1	NM_014351	sulfotransferase family 4A, member 1	-4.77159	down	-3.5427861	down
DCUN1D2	NM_001014283	DCN1, defective in cullin neddylation 1, domain containing 2	-4.7389073	down	-3.6211264	down
LINC00551		long intergenic non-protein coding RNA 551	-6.7207165	down	-2.9179118	down
			-6.762854	down	-2.3440614	down
LOC100506191	AF289610	uncharacterized LOC100506191	-5.593989	down	-2.304706	down
PRDX4	XM_005274438	peroxiredoxin 4	-2.4713347	down	-2.0758712	down
			-4.241283	down	-2.5028021	down
GPSM1	NM_015597	G-protein signaling modulator 1	-7.4389806	down	-3.4216862	down
C2CD4D	NM_001136003	C2 calcium-dependent domain containing 4D	-2.365709	down	-2.1858833	down
HIST1H4E	NM_003545	histone cluster 1, H4e	-2.2334175	down	-2.8773859	down
HCAR1	NM_032554	hydroxycarboxylic acid receptor 1	-5.485897	down	-2.687955	down
	BG957402		-7.5299973	down	-2.1933568	down
ZDHHC8P1	NR_003950	zinc finger, DHHC-type containing 8 pseudogene 1	-6.426266	down	-3.9944594	down
IKBKB	AK309052	inhibitor of kappa light polypeptide gene enhancer in B-cells, kinase beta	-2.5327141	down	-2.0707302	down
CASC8	NR_024393	cancer susceptibility candidate 8 (non-protein coding)	-4.968937	down	-2.752713	down
			-3.1215482	down	-3.951643	down
CDHR5	NM_031264	cadherin-related family member 5	-3.7579374	down	-2.1542735	down
TNFSF11	NM_003701	tumor necrosis factor (ligand) superfamily, member 11	-4.0673733	down	-3.0199182	down
	AB529275		-5.685839	down	-2.7506073	down
	BC069659		-5.8605723	down	-3.5412123	down
LOC100133091	NR_029411	uncharacterized LOC100133091	-3.669263	down	-3.6314611	down
	XR_241347		-4.2305098	down	-2.3596847	down
HCP5B	BC035659	HLA complex P5B (non-protein coding)	-2.7624404	down	-2.2379448	down
MBL1P		mannose-binding lectin (protein A) 1, pseudogene	-4.385063	down	-2.250458	down
OR2D3	NM_001004684	olfactory receptor, family 2, subfamily D, member 3	-6.4077725	down	-2.0092852	down
OR52W1	NM_001005178	olfactory receptor, family 52, subfamily W, member 1	-5.795704	down	-2.0155756	down
HIST1H4L	NM_003546	histone cluster 1, H4l	-3.6117704	down	-2.3230183	down
POLR3G	NM_006467	polymerase (RNA) III (DNA directed) polypeptide G (32kD)	-4.5163455	down	-4.865662	down
	AK128523		-2.950879	down	-3.2098026	down
			-4.7508554	down	-2.2627273	down
SHROOM4	NM_020717	shroom family member 4	-3.1549098	down	-2.698823	down
KIAA1598	NM_001258299	KIAA1598	-3.210234	down	-2.2871034	down
			-4.5786552	down	-3.6364934	down
ODF2L	NM_001007022	outer dense fiber of sperm tails 2-like	-2.8438332	down	-2.6571915	down
	XR_158963		-3.9666255	down	-3.4660625	down
LOC100131581	AK092544	uncharacterized LOC100131581	-4.9146338	down	-3.0167692	down
GDF10	NM_004962	growth differentiation factor 10	-2.9069853	down	-3.432062	down
SLC35F2	NM_017515	solute carrier family 35, member F2	-3.9931924	down	-4.473814	down
ORAOV1	XM_005274423	oral cancer overexpressed 1	-2.6955535	down	-2.090473	down
LOC729080	NR_033244	glycine cleavage system protein H (aminomethyl carrier) pseudogene	-2.50403	down	-4.1445174	down
TSPEAR	NM_144991	thrombospondin-type laminin G domain and EAR repeats	-3.7105336	down	-2.1994097	down
GPR157	NM_024980	G protein-coupled receptor 157	-4.7851667	down	-2.2411687	down
GOLGA6L10	NM_001164465	golgin A6 family-like 10	-11.504027	down	-6.690217	down
			-4.657573	down	-3.3173008	down
TIAL1	AB209260	TIA1 cytotoxic granule-associated RNA binding protein-like 1	-3.5871084	down	-2.5260284	down
LINC00205	AF426264	long intergenic non-protein coding RNA 205	-3.2771027	down	-2.068929	down
LOC100128653	XR_108727	uncharacterized LOC100128653	-4.25126	down	-2.6321714	down
GUCA2B	NM_007102	guanylate cyclase activator 2B (uroguanylin)	-2.9126515	down	-2.9069526	down
LOC440461	NR_027283	Rho GTPase activating protein 27 pseudogene	-3.0236762	down	-2.007196	down
	AL833395		-4.758976	down	-4.232102	down
			-4.334282	down	-2.4203575	down
ADAM12	NM_003474	ADAM metallopeptidase domain 12	-6.5722375	down	-5.1799927	down
PCBP3-OT1	AK126016	PCBP3 overlapping transcript (non-protein coding)	-4.3989706	down	-4.1296663	down
XLOC_014512			-3.846768	down	-3.3598876	down
KRTAP4-7	NM_033061	keratin associated protein 4-7	-2.7487543	down	-2.7129662	down
ZNF577		zinc finger protein 577	-4.8343444	down	-2.2719307	down
XLOC_014512			-3.0384092	down	-6.1747327	down

UNC5B	NM_170744	unc-5 homolog B (C. elegans)	-4.787286	down	-2.8932836	down
			-6.249618	down	-3.1547675	down
DCAF8L1	NM_001017930	DDB1 and CUL4 associated factor 8-like 1	-2.110095	down	-2.2685955	down
GRIK4	NM_001282470	glutamate receptor, ionotropic, kainate 4	-7.2889075	down	-5.004351	down
MYO3A	NM_017433	myosin IIIA	-7.0374346	down	-6.171576	down
VSX2	NM_182894	visual system homeobox 2	-3.971455	down	-2.4018989	down
LIPH	NM_139248	lipase, member H	-4.1432076	down	-2.4428647	down
			-2.7833745	down	-2.495093	down
MARK4	NM_031417	MAP/microtubule affinity-regulating kinase 4	-3.4606154	down	-2.6387324	down
TSPAN16	NM_012466	tetraspanin 16	-3.8295674	down	-5.547237	down
LOC283112	AK096599	uncharacterized LOC283112	-3.0908506	down	-2.2130513	down
HSD52		uncharacterized LOC729467	-4.1009865	down	-2.3608165	down
TMEM229B	XR_245666	transmembrane protein 229B	-2.950043	down	-2.2172356	down
TRIM49D1	NM_001206627	tripartite motif containing 49D1	-6.2758837	down	-4.8642063	down
LOC729444	NR_038388	uncharacterized LOC729444	-2.953629	down	-2.3946958	down
AKAP14	NM_001008535	A kinase (PRKA) anchor protein 14	-9.706893	down	-3.7234235	down
PPP6R1	NM_014931	protein phosphatase 6, regulatory subunit 1	-2.7125459	down	-3.7880533	down
LRCOL1	NM_001195520	leucine rich colipase-like 1	-7.937017	down	-2.3316307	down
LRRC2	NM_024512	leucine rich repeat containing 2	-8.663694	down	-4.936049	down
	XR_253722		-5.3861666	down	-4.1076794	down
ABCC6	NM_001079528	ATP-binding cassette, sub-family C (CFTR/MRP), member 6	-4.704009	down	-2.3465993	down
COL23A1	NM_173465	collagen, type XXIII, alpha 1	-2.6393318	down	-2.4980407	down
DSCR10	NR_027695	Down syndrome critical region gene 10 (non-protein coding)	-2.3509786	down	-4.523501	down
ARNT2	NM_014862	aryl-hydrocarbon receptor nuclear translocator 2	-2.1457686	down	-3.672476	down
WNT9A	NM_003395	wingless-type MMTV integration site family, member 9A	-4.6638374	down	-2.4781842	down
CALY	XM_005252686	calcyon neuron-specific vesicular protein	-2.646121	down	-3.2764416	down
TAS2R60	NM_177437	taste receptor, type 2, member 60	-3.5869987	down	-7.17155	down
	AK091409		-3.8394096	down	-2.1723459	down
CSPG4P8	NR_033579	chondroitin sulfate proteoglycan 4 pseudogene 8	-2.690664	down	-4.108693	down
FLJ34223		uncharacterized LOC440479	-2.80412	down	-3.636388	down
			-3.1214278	down	-2.0357	down
NXPH3	NM_007225	neurexophilin 3	-7.387283	down	-5.9469247	down
FOSL1	NM_005438	FOS-like antigen 1	-3.677499	down	-2.878679	down
ODAM	NM_017855	odontogenic, ameloblast asssociated	-5.745308	down	-2.316448	down
RPA4	NM_013347	replication protein A4, 30kDa	-6.4615455	down	-5.7699113	down
	XR_249135		-2.4342716	down	-2.6885736	down
LOC151475		uncharacterized LOC151475	-4.511114	down	-2.0918717	down
BEND4	NM_207406	BEN domain containing 4	-5.315073	down	-2.7898242	down
	M15530		-7.6152816	down	-3.8116748	down
B4GALNT1	NM_001478	beta-1,4-N-acetyl-galactosaminyl transferase 1	-6.0181375	down	-4.1517744	down
LOC729159	NM_001282301	UPF0607 protein ENSP00000381418-like	-3.909766	down	-2.6409407	down
INTU	NM_015693	inturned planar cell polarity protein	-2.1973627	down	-2.0360618	down
GPT	NM_005309	glutamic-pyruvate transaminase (alanine aminotransferase)	-17.095665	down	-12.025615	down
POF1B	NM_024921	premature ovarian failure, 1B	-4.3078823	down	-3.9651456	down
LOC441728	XR_109175	golgin-like	-10.269858	down	-11.882804	down
FGF22	NM_020637	fibroblast growth factor 22	-3.7246077	down	-2.0708148	down
HEATR4	NM_203309	HEAT repeat containing 4	-3.8288264	down	-2.7644792	down
OR10R3P	AK122667	olfactory receptor, family 10, subfamily R, member 3 pseudogene	-4.039973	down	-2.766414	down
OR2M7	NM_001004691	olfactory receptor, family 2, subfamily M, member 7	-4.7752323	down	-3.0194101	down
EMX1	BC037242	empty spiracles homeobox 1	-2.2458196	down	-2.7129996	down
MST1L	NM_001271733	macrophage stimulating 1-like	-5.965646	down	-3.6798	down
RGMA	NM_020211	repulsive guidance molecule family member a	-2.3790376	down	-2.797557	down
LHX1	NM_005568	LIM homeobox 1	-11.5837	down	-16.453512	down
			-10.089806	down	-3.8611047	down
CSDC2	NM_014460	cold shock domain containing C2, RNA binding	-3.5730286	down	-2.2499437	down
GMNC	NM_001146686	geminin coiled-coil domain containing	-3.3997037	down	-2.2894156	down
LINC00941		long intergenic non-protein coding RNA 941	-4.341432	down	-2.8990226	down
GNN	NR_027249	Grp94 neighboring nucleotidase pseudogene	-4.8369975	down	-3.0765047	down
	AK130724		-3.016074	down	-3.0144577	down
ZSCAN30	AK131291	zinc finger and SCAN domain containing 30	-2.652367	down	-2.432667	down

	AK130932		-3.6162224	down	-2.50488	down
	DA666023		-3.3337607	down	-5.5206895	down
PAX8	NM_003466	paired box 8	-6.5135703	down	-2.3970957	down
IGFN1	NM_001164586	immunoglobulin-like and fibronectin type III domain containing 1	-3.8319924	down	-2.936139	down
SNORA60	BF304636	small nucleolar RNA, H/ACA box 60	-14.885139	down	-24.101852	down
TAB3	AL832071	TGF-beta activated kinase 1/MAP3K7 binding protein 3	-3.1210954	down	-2.0841086	down
DNAI1	NM_012144	dynein, axonemal, intermediate chain 1	-5.2658434	down	-2.885911	down
DMTF1	NR_024549	cyclin D binding myb-like transcription factor 1	-2.2022564	down	-2.3297913	down
OR2T6	NM_001005471	olfactory receptor, family 2, subfamily T, member 6	-5.5887094	down	-2.9359596	down
			-2.3561962	down	-2.2367468	down
PPIEL	AK093659	peptidylprolyl isomerase E-like pseudogene	-2.6236956	down	-2.3107414	down
EYA2	NM_005244	eyes absent homolog 2 (Drosophila)	-4.438241	down	-3.1323988	down
APOA1	NM_000039	apolipoprotein A-I	-3.2232969	down	-2.5555198	down
RNU105B	NR_004386	RNA, U105B small nucleolar	-2.6134226	down	-2.052073	down
GOLGA8A	NM_181077	golgin A8 family, member A	-2.3954964	down	-3.1745539	down
			-4.5105433	down	-2.0300515	down
GOLT1A	NM_198447	golgi transport 1A	-2.2132843	down	-2.2550058	down
LOC402160		uncharacterized LOC402160	-5.117429	down	-2.94199	down
ZBTB8B	NM_001145720	zinc finger and BTB domain containing 8B	-3.9387116	down	-2.5661407	down
MAGI2	NM_012301	membrane associated guanylate kinase, WW and PDZ domain containing 2	-3.9937086	down	-2.0878015	down
MIR17HG	NR_027350	miR-17-92 cluster host gene (non-protein coding)	-5.763578	down	-4.278801	down
FAM166A	XM_005266087	family with sequence similarity 166, member A	-5.8831716	down	-3.26692	down
NLRP7	NM_139176	NLR family, pyrin domain containing 7	-2.160418	down	-2.6873498	down
C1orf61	XM_005244829	chromosome 1 open reading frame 61	-5.2116704	down	-4.1522026	down
ARHGAP23	NM_001199417	Rho GTPase activating protein 23	-3.52002	down	-2.0313954	down
CDHR3	NM_152750	cadherin-related family member 3	-5.453435	down	-2.9512844	down
OR1J1	NM_001004451	olfactory receptor, family 1, subfamily J, member 1	-4.8351145	down	-2.8675408	down
PSG11	NM_002785	pregnancy specific beta-1-glycoprotein 11	-2.7547078	down	-2.0961835	down
RREB1	NM_001003699	ras responsive element binding protein 1	-2.8816469	down	-2.9915798	down
			-3.475632	down	-4.0586104	down
			-2.9290357	down	-3.7115092	down
MACROD2	BC035876	MACRO domain containing 2	-2.7689078	down	-3.7093298	down
LOC442132		golgin A6 family-like 1 pseudogene	-3.2626016	down	-2.6582966	down
			-3.6953633	down	-4.7340984	down
PITX2	NM_153426	paired-like homeodomain 2	-4.612477	down	-3.0472758	down
			-5.3786416	down	-3.4793699	down
			-7.823745	down	-4.4878373	down
LHX9	NM_020204	LIM homeobox 9	-4.391915	down	-3.2342193	down
GPR179	NM_001004334	G protein-coupled receptor 179	-8.6036825	down	-3.5796883	down
TPH2	NM_173353	tryptophan hydroxylase 2	-11.120205	down	-7.6923847	down
MAPK15	NM_139021	mitogen-activated protein kinase 15	-2.143195	down	-2.5821118	down
MOB3B	NM_024761	MOB kinase activator 3B	-2.8915176	down	-3.4244726	down
GPR98	NM_032119	G protein-coupled receptor 98	-6.1065216	down	-4.3317847	down
AQP4	NM_001650	aquaporin 4	-5.1011415	down	-3.0286992	down
MPL	NM_005373	myeloproliferative leukemia virus oncogene	-3.005372	down	-2.378023	down
FBLN1	NM_001996	fibulin 1	-5.5976233	down	-6.499015	down
			-3.9432645	down	-2.2055886	down
PPP1R27	NM_001007533	protein phosphatase 1, regulatory subunit 27	-5.734787	down	-5.269405	down
			-4.3869987	down	-2.184193	down
LOC286442		uncharacterized LOC286442	-4.339916	down	-2.328914	down
SPEN	NM_015001	spen family transcriptional repressor	-8.814157	down	-5.6074247	down
			-6.3649473	down	-2.3358502	down
SPATA31A3	NM_001083124	SPATA31 subfamily A, member 3	-6.6184096	down	-3.3604574	down
OR4C6	NM_001004704	olfactory receptor, family 4, subfamily C, member 6	-19.2936	down	-11.45397	down
CDRT1	NM_001282540	CMT1A duplicated region transcript 1	-6.194238	down	-4.3554344	down
			-2.4670947	down	-4.1436296	down
CACNA1B	NM_000718	calcium channel, voltage-dependent, N type, alpha 1B subunit	-4.147942	down	-4.9678426	down
RNASE12	NM_001024822	ribonuclease, RNase A family, 12 (non-active)	-4.9715176	down	-2.7973037	down
APC	NM_001127511	adenomatous polyposis coli	-2.448398	down	-2.182364	down
			-4.7872157	down	-4.1678476	down
SPON2	AK124606	spondin 2, extracellular matrix protein	-3.202906	down	-2.3445394	down

			-5.53947	down	-3.3159459	down
	BC046635		-4.5571737	down	-3.8089678	down
RGS8	NM_033345	regulator of G-protein signaling 8	-5.530544	down	-2.525994	down
	XR_248969		-4.0392647	down	-2.3164892	down
	BC015677		-5.105051	down	-2.8813436	down
RAP1GAP	NM_002885	RAP1 GTPase activating protein	-2.9104455	down	-3.1807668	down
LOC100130539	NM_001258000	uncharacterized LOC100130539	-2.5246282	down	-2.0935957	down
			-4.4528317	down	-2.2228973	down
KCNJ14	NM_013348	potassium inwardly-rectifying channel, subfamily J, member 14	-2.850899	down	-2.4246068	down
ENTPD1	NM_001776	ectonucleoside triphosphate diphosphohydrolase 1	-2.5639234	down	-2.13871	down
LOC286177	NR_038874	uncharacterized LOC286177	-3.6278076	down	-2.1548452	down
			-2.3879719	down	-2.7265215	down
FSHR	NM_000145	follicle stimulating hormone receptor	-3.3127756	down	-2.267113	down
GPM6B	NM_001001995	glycoprotein M6B	-3.0634086	down	-2.3533	down
ARHGAP23	NM_001199417	Rho GTPase activating protein 23	-4.858368	down	-3.3287895	down
			-4.8783894	down	-3.0666175	down
			-5.2587976	down	-3.3255405	down
PGM5	NM_021965	phosphoglucomutase 5	-2.2973363	down	-2.2664988	down
KRT19P2	NR_036685	keratin 19 pseudogene 2	-3.348448	down	-2.1444669	down
KAAG1	NM_181337	kidney associated antigen 1	-4.3520412	down	-2.9201388	down
FBXL7	NM_012304	F-box and leucine-rich repeat protein 7	-5.4775863	down	-2.2919905	down
LINC00910		long intergenic non-protein coding RNA 910	-7.4299035	down	-2.4430544	down
ULBP2	NM_025217	UL16 binding protein 2	-6.8728576	down	-3.1869807	down
SERPINA4	NM_006215	serpin peptidase inhibitor, clade A (alpha-1 antiproteinase, antitrypsin), member 4	-3.1868882	down	-3.599843	down
ELK1	NM_001257168	ELK1, member of ETS oncogene family	-8.585914	down	-8.91259	down
ANKRD20A8P	NR_003366	ankyrin repeat domain 20 family, member A8, pseudogene	-6.176295	down	-4.333573	down
TMEM178B		transmembrane protein 178B	-3.5479217	down	-2.201137	down
PIP5K1A	NM_003557	phosphatidylinositol-4-phosphate 5-kinase, type I, alpha	-3.5278819	down	-4.3902464	down
SPTBN4	NM_020971	spectrin, beta, non-erythrocytic 4	-3.0292487	down	-3.9215827	down
			-5.932906	down	-3.4587607	down
DCD	NM_053283	dermcidin	-3.9843438	down	-2.4074175	down
HHIP	NM_022475	hedgehog interacting protein	-2.439893	down	-3.1273031	down
SHC3	NM_016848	SHC (Src homology 2 domain containing) transforming protein 3	-2.7798693	down	-2.0452006	down
CCDC108	NM_152389	coiled-coil domain containing 108	-3.7554383	down	-2.321727	down
LOC283731	BC050067	uncharacterized LOC283731	-5.6335015	down	-3.443976	down
TSGA10	NM_025244	testis specific, 10	-4.2336516	down	-2.2358823	down
FAM230C	NR_027278	family with sequence similarity 230, member C	-6.3683257	down	-3.6202443	down
EXTL1	NM_004455	exostosin-like glycosyltransferase 1	-2.0882576	down	-4.033939	down
SOX3	NM_005634	SRY (sex determining region Y)-box 3	-7.3945627	down	-3.300588	down
PHLDB3	AK097512	pleckstrin homology-like domain, family B, member 3	-3.8866968	down	-2.2092724	down
			-3.2939706	down	-3.443294	down
USH1C	NM_005709	Usher syndrome 1C (autosomal recessive, severe)	-5.6109343	down	-2.880904	down
	XR_171112		-4.5336623	down	-2.611336	down
	AK123118		-5.9080386	down	-6.6710963	down
ZNF331		zinc finger protein 331	-5.116043	down	-3.0107036	down
LRRC3	NM_030891	leucine rich repeat containing 3	-5.487424	down	-2.853224	down
PDGFRA	NM_006206	platelet-derived growth factor receptor, alpha polypeptide	-2.4526863	down	-2.2322545	down
DPF3	AK024141	D4, zinc and double PHD fingers, family 3	-2.124125	down	-2.080736	down
FAM27A	NR_024060	family with sequence similarity 27, member A	-3.115372	down	-4.4144225	down
			-2.6201854	down	-2.9267063	down
			-2.047749	down	-2.0754888	down
	BC041650		-4.693455	down	-3.5388305	down
PRAMEF22	NM_001100631	PRAME family member 22	-3.2595613	down	-2.1726177	down
NFASC	NM_001005388	neurofascin	-5.391501	down	-6.7971063	down
LOC101927910	XM_005256332	keratin-associated protein 5-5-like	-2.2112243	down	-2.005248	down
			-5.157066	down	-2.634412	down
			-3.0468583	down	-2.4412186	down
	AK123308		-3.7871592	down	-2.1073499	down
			-3.4931276	down	-2.2421494	down
	CU678159		-4.846243	down	-3.3637216	down

LOC441204		uncharacterized LOC441204	-5.868776	down	-3.853323	down
ABCB9	NM_019625	ATP-binding cassette, sub-family B (MDR/TAP), member 9	-3.5484471	down	-2.612492	down
EML5	NM_183387	echinoderm microtubule associated protein like 5	-4.5585327	down	-9.253154	down
LOC200726	NM_001102659	hCG1657980	-6.687719	down	-5.9616265	down
			-3.3375595	down	-2.2873392	down
BSX	NM_001098169	brain-specific homeobox	-6.2262125	down	-2.4991012	down
			-2.0069022	down	-3.1697426	down
CASKIN1	NM_020764	CASK interacting protein 1	-2.8927066	down	-2.277006	down
DGCR5		DiGeorge syndrome critical region gene 5 (non-protein coding)	-4.28018	down	-2.143334	down
NEUROG1	NM_006161	neurogenin 1	-5.3619804	down	-3.1397324	down
KIRREL3-AS3	NR_040078	KIRREL3 antisense RNA 3	-3.6323845	down	-5.0667768	down
NR2F2-AS1	XR_248180	NR2F2 antisense RNA 1	-3.7656596	down	-2.6659617	down
DLK1	NM_003836	delta-like 1 homolog (Drosophila)	-3.1148267	down	-2.7227533	down
RAPH1	NM_213589	Ras association (RalGDS/AF-6) and pleckstrin homology domains 1	-3.9197226	down	-2.3589814	down
PHLDA3	NM_012396	pleckstrin homology-like domain, family A, member 3	-3.2136095	down	-2.6204505	down
CIITA	NM_001286403	class II, major histocompatibility complex, transactivator	-5.077524	down	-2.8290663	down
			-5.3983345	down	-4.9868937	down
	XM_005245645		-3.9965096	down	-3.0538406	down
PAX5	NM_016734	paired box 5	-4.005408	down	-2.3719497	down
LOC116437	NR_026670	uncharacterized LOC116437	-5.4227715	down	-4.1206765	down
GLRA1	NM_000171	glycine receptor, alpha 1	-4.4588695	down	-2.484067	down
RNU105C	NR_004385	RNA, U105C small nucleolar	-11.19681	down	-7.9807844	down
KIF6	NM_145027	kinesin family member 6	-3.9143476	down	-2.2739236	down
AZGP1	NM_001185	alpha-2-glycoprotein 1, zinc-binding	-9.540843	down	-2.3737552	down
IKZF2	NM_001079526	IKAROS family zinc finger 2 (Helios)	-2.601485	down	-2.5392673	down
			-5.3467007	down	-3.2232668	down
			-4.261249	down	-2.107883	down
C1orf95	NM_001003665	chromosome 1 open reading frame 95	-5.0441523	down	-2.5306077	down
CHEK2	NM_145862	checkpoint kinase 2	-2.3252156	down	-2.01323	down
			-2.1989734	down	-3.9305325	down
SCN8A	NM_014191	sodium channel, voltage gated, type VIII, alpha subunit	-4.553953	down	-2.4663725	down
LINC00452	XR_246655	long intergenic non-protein coding RNA 452	-3.9686918	down	-2.4761572	down
PTCHD4	NM_001013732	patched domain containing 4	-4.50329	down	-2.5933256	down
	AF130075		-7.4900355	down	-4.5568705	down
PDE4DIP	NM_001198832	phosphodiesterase 4D interacting protein	-3.7096884	down	-5.6383185	down
LOC401188	AK091013	uncharacterized LOC401188	-4.323573	down	-2.4214504	down
GOLGA6L4	NM_001267536	golgin A6 family-like 4	-11.619715	down	-7.4029164	down
CEP55	NM_018131	centrosomal protein 55kDa	-3.6055336	down	-2.0650635	down
SH3GL1P2	NR_033420	SH3-domain GRB2-like 1 pseudogene 2	-2.1710477	down	-4.760231	down
MYO18B	NM_032608	myosin XVIIIB	-2.7982197	down	-2.263577	down
SLC25A30-AS1	XR_172004	SLC25A30 antisense RNA 1	-5.4230833	down	-2.3404934	down
			-4.927114	down	-3.1189828	down
AACSP1		acetoacetyl-CoA synthetase pseudogene 1	-6.807344	down	-8.845006	down
			-3.1793828	down	-2.2812047	down
POM121L9P	NR_003714	POM121 transmembrane nucleoporin-like 9, pseudogene	-4.6989236	down	-3.0508938	down
FLJ16779	NR_024389	uncharacterized LOC100192386	-2.6983058	down	-2.0264542	down
GALNT5	NM_014568	UDP-N-acetyl-alpha-D-galactosamine:polypeptide N-acetylgalactosaminyltransferase 5 (GalNAc-T5)	-3.7609816	down	-3.2798953	down
ANKRD20A3	XM_005276027	ankyrin repeat domain 20 family, member A3	-7.36758	down	-5.3436694	down
	XR_112948		-5.93326	down	-4.5668626	down
			-5.271536	down	-2.1753078	down
MPP3	NM_001932	membrane protein, palmitoylated 3 (MAGUK p55 subfamily member 3)	-3.6993234	down	-3.2927377	down
SLC38A8	NM_001080442	solute carrier family 38, member 8	-4.513118	down	-2.4864526	down
MYADML2	NM_001145113	myeloid-associated differentiation marker-like 2	-4.480282	down	-2.3721561	down
	AF068294		-4.001837	down	-3.0097098	down
RUNX1	NM_001122607	runt-related transcription factor 1	-3.6770244	down	-4.8424187	down
			-5.349376	down	-3.8398585	down
GLB1L3	NM_001080407	galactosidase, beta 1-like 3	-2.864664	down	-2.0269883	down
LOC143286	AL049428	uncharacterized LOC143286	-5.528928	down	-3.7336493	down
	AF495723		-7.263195	down	-3.4110782	down

	BC039440		-6.5707564	down	-4.5986185	down
LINC00111		long intergenic non-protein coding RNA 111	-9.909001	down	-6.9616804	down
SLC25A53	NM_001012755	solute carrier family 25, member 53	-2.7965713	down	-2.0241315	down
BSPRY	NM_017688	B-box and SPRY domain containing	-2.0788772	down	-2.912585	down
NFATC2	NM_173091	nuclear factor of activated T-cells, cytoplasmic, calcineurin-dependent 2	-2.7129464	down	-3.1096048	down
	AF116680		-4.3722887	down	-3.4316304	down
MGC15705	BC007304	uncharacterized protein MGC15705	-2.6516984	down	-2.3447604	down
DAZ1	NM_004081	deleted in azoospermia 1	-4.2207193	down	-2.696044	down
BAALC		brain and acute leukemia, cytoplasmic	-3.9340394	down	-2.5718036	down
XLOC_014512			-4.6114936	down	-2.9526112	down
			-3.5228634	down	-2.9310122	down
			-2.2054574	down	-3.3041184	down
			-3.3535411	down	-2.9740458	down
LOC100130278	AK094114	uncharacterized LOC100130278	-4.352934	down	-2.6810272	down
MYLK3	NM_182493	myosin light chain kinase 3	-4.8094454	down	-2.6045861	down
ZEB1-AS1	CD674797	ZEB1 antisense RNA 1	-2.5606425	down	-2.2103498	down
KCNH6	NM_173092	potassium voltage-gated channel, subfamily H (eag-related), member 6	-7.2367606	down	-3.789919	down
C7orf31	NM_138811	chromosome 7 open reading frame 31	-3.9815376	down	-2.5165324	down
ASB15	NM_080928	ankyrin repeat and SOCS box containing 15	-4.1943336	down	-2.018217	down
LOC100128501	AK092192	uncharacterized LOC100128501	-2.1971228	down	-2.9409704	down
TWIST2	NM_001271893	twist family bHLH transcription factor 2	-2.498709	down	-2.13133	down
CHN2	XM_005249602	chimerin 2	-3.31459	down	-2.1265008	down
OPRL1	NM_182647	opiate receptor-like 1	-2.5215628	down	-3.7429655	down
			-4.053519	down	-2.713801	down
FAM83F	NM_138435	family with sequence similarity 83, member F	-2.1103554	down	-2.2759414	down
LOC100129148	NR_033999	uncharacterized LOC100129148	-6.381669	down	-3.8475778	down
VPS37D	NM_001077621	vacuolar protein sorting 37 homolog D (S. cerevisiae)	-2.9814596	down	-2.6878357	down
ZC3H12D	NM_207360	zinc finger CCCH-type containing 12D	-5.7907715	down	-6.3925047	down
GPR133	NM_198827	G protein-coupled receptor 133	-4.3677216	down	-3.547708	down
			-5.0685506	down	-2.664155	down
NCAPG	NM_022346	non-SMC condensin I complex, subunit G	-4.2137847	down	-2.4537015	down
NTNG2	AY358165	netrin G2	-7.3633924	down	-6.8432245	down
TTC40	NM_001200049	tetratricopeptide repeat domain 40	-2.0136626	down	-2.4544106	down
AMER2	NM_152704	APC membrane recruitment protein 2	-3.7883055	down	-2.0918944	down
ADRA1B	NM_000679	adrenoceptor alpha 1B	-2.41941	down	-2.8089519	down
GJD4	BC035898	gap junction protein, delta 4, 40.1kDa	-4.75909	down	-6.991357	down
C22orf42	NM_001010859	chromosome 22 open reading frame 42	-4.2366695	down	-3.8820403	down
LOC100128105		uncharacterized LOC100128105	-5.372041	down	-2.01934	down
RUFY4	NM_198483	RUN and FYVE domain containing 4	-2.5532694	down	-3.057439	down
LOC285300		uncharacterized LOC285300	-4.2367067	down	-3.0888019	down
TSG1	NR_015362	tumor suppressor TSG1	-4.174873	down	-2.380016	down
			-4.5816817	down	-4.532669	down
			-2.6276996	down	-2.3408945	down
	AF130091		-2.744198	down	-2.0109074	down
REG3A	NM_138938	regenerating islet-derived 3 alpha	-3.5031226	down	-2.559145	down
NPLOC4	AB040932	nuclear protein localization 4 homolog (S. cerevisiae)	-3.555991	down	-2.1306121	down
ONECUT3	NM_001080488	one cut homeobox 3	-3.2645967	down	-3.3143384	down
PRSS8	NM_002773	protease, serine, 8	-6.2877145	down	-4.0148997	down
C17orf105	NM_001136483	chromosome 17 open reading frame 105	-3.658769	down	-2.9773674	down
LYZL1	NM_032517	lysozyme-like 1	-6.901953	down	-4.044266	down
	AB305689		-2.1255066	down	-2.2967505	down
ZNF682	NM_033196	zinc finger protein 682	-5.54705	down	-3.902928	down
TTLL6	NM_173623	tubulin tyrosine ligase-like family, member 6	-3.27067	down	-3.057029	down
	AK124325		-4.2009473	down	-2.5298648	down
C2orf53	NM_178553	chromosome 2 open reading frame 53	-2.501564	down	-2.0435889	down
TSPY26P	NR_002781	testis specific protein, Y-linked 26, pseudogene	-3.6118186	down	-2.7128167	down
			-2.5757632	down	-2.9023128	down
POTEB	NM_207355	POTE ankyrin domain family, member B	-5.3219733	down	-4.1299067	down
LARP4B	NM_015155	La ribonucleoprotein domain family, member 4B	-3.7670898	down	-2.2183692	down
PROX2	NM_001243007	prospero homeobox 2	-5.8180184	down	-5.0145664	down
	AF390550		-3.377641	down	-2.703168	down
FAM188B	AK090826	family with sequence similarity 188, member B	-3.2656837	down	-2.0660553	down

LINC00548		long intergenic non-protein coding RNA 548	-3.4382236	down	-2.267029	down
HMGB3P1	NR_002165	high mobility group box 3 pseudogene 1	-3.4374135	down	-3.4973514	down
SPC24	AK075287	SPC24, NDC80 kinetochore complex component	-4.6533856	down	-2.3197775	down
MEGF11	NM_032445	multiple EGF-like-domains 11	-2.6790116	down	-4.04248	down
ZP4	NM_021186	zona pellucida glycoprotein 4	-5.587552	down	-2.7163396	down
MAT1A	NM_000429	methionine adenosyltransferase I, alpha	-3.8006558	down	-3.4326124	down
SH2D4B	NM_207372	SH2 domain containing 4B	-3.73974	down	-3.135691	down
GH1	NM_000515	growth hormone 1	-2.8961325	down	-2.542353	down
ERO1LB		ERO1-like beta (S. cerevisiae)	-3.8247004	down	-2.0460145	down
CYCSP52	NR_001560	cytochrome c, somatic pseudogene 52	-5.9181232	down	-2.7928193	down
			-4.169155	down	-3.0524366	down
LOC729739	BC013681	peptidylprolyl isomerase A (cyclophilin A) pseudogene	-5.952292	down	-3.0480518	down
ARHGEF4	NM_015320	Rho guanine nucleotide exchange factor (GEF) 4	-2.9063163	down	-2.0614185	down
MAGEC1	NM_005462	melanoma antigen family C, 1	-5.0645366	down	-3.344572	down
			-2.954408	down	-3.5126047	down
LOC100130372	AK127532	uncharacterized LOC100130372	-5.8386774	down	-2.0686522	down
LOC402779	NR_038372	uncharacterized LOC402779	-2.578671	down	-2.2035089	down
STAG2	NM_001042751	stromal antigen 2	-3.316222	down	-2.3683817	down
CALCA	NM_001033952	calcitonin-related polypeptide alpha	-2.9686708	down	-2.9556026	down
AATK	XM_005257848	apoptosis-associated tyrosine kinase	-6.053745	down	-3.704955	down
S100A7A	AY189119	S100 calcium binding protein A7A	-2.3899438	down	-3.0437043	down
TMEM99	NM_001195386	transmembrane protein 99	-3.0904317	down	-2.2920058	down
SATB2	NM_015265	SATB homeobox 2	-3.6700046	down	-2.0754857	down
			-4.7635064	down	-2.615217	down
VSTM2L	NM_080607	V-set and transmembrane domain containing 2 like	-6.645723	down	-6.580755	down
LOC100128176	NR_038408	uncharacterized LOC100128176	-4.1481166	down	-2.3471353	down
C4orf50	XM_003118524	chromosome 4 open reading frame 50	-4.803954	down	-3.3959832	down
			-4.2472863	down	-3.8274066	down
			-9.365239	down	-8.7195015	down
MYH7B	NM_020884	myosin, heavy chain 7B, cardiac muscle, beta	-4.8613653	down	-2.909175	down
			-3.5452375	down	-3.4789088	down
TMEM132C	NM_001136103	transmembrane protein 132C	-4.38772	down	-2.8326926	down
GPC6	NM_005708	glypican 6	-5.3728676	down	-2.7232473	down
LOC100128950	AK124198	uncharacterized LOC100128950	-5.8576503	down	-3.310135	down
			-7.9491067	down	-8.349956	down
LOC729911	NR_038997	uncharacterized LOC729911	-5.487533	down	-3.3559012	down
C2orf16	NM_032266	chromosome 2 open reading frame 16	-3.6598673	down	-7.9584894	down
STON2	NM_033104	stonin 2	-3.67418	down	-2.5440195	down
CEP72	NM_018140	centrosomal protein 72kDa	-2.8053527	down	-2.883138	down
PLA2G2D	BC025706	phospholipase A2, group IID	-5.292874	down	-2.4678793	down
MAGEA11	NM_001011544	melanoma antigen family A, 11	-4.0140657	down	-2.2949226	down
	AJ276252		-3.4094586	down	-2.196858	down
TRPM3	NM_206948	transient receptor potential cation channel, subfamily M, member 3	-3.452296	down	-4.561398	down
LOC100132356	NR_034127	uncharacterized LOC100132356	-2.7319398	down	-2.5004866	down
HTRA3	NM_053044	HtrA serine peptidase 3	-3.8360732	down	-2.380205	down
			-6.7110906	down	-4.2568326	down
BTBD19	NM_001136537	BTB (POZ) domain containing 19	-4.391678	down	-3.4949558	down
XLOC_014512			-3.4835758	down	-2.3657763	down
PRAMEF4	NM_001009611	PRAME family member 4	-4.5565324	down	-5.2520337	down
			-3.2805517	down	-2.0368047	down
FCER1G		Fc fragment of IgE, high affinity I, receptor for; gamma polypeptide	-2.0793395	down	-2.0450554	down
OPRM1	NM_001008504	opioid receptor, mu 1	-2.0698018	down	-2.3947942	down
FLJ13224	NR_026806	uncharacterized LOC79857	-3.4342465	down	-2.504551	down
UBE2I	XM_005255542	ubiquitin-conjugating enzyme E2I	-3.2711504	down	-3.1578462	down
FLJ44674	AK128747	FLJ44674 protein	-4.437907	down	-2.497909	down
XLOC_014512			-3.7397025	down	-2.2224689	down
OR52E2	NM_001005164	olfactory receptor, family 52, subfamily E, member 2	-11.298313	down	-12.850661	down
	AK128779		-4.7531257	down	-3.1691606	down
			-6.413644	down	-2.450457	down
ANKRD30BL	NR_027019	ankyrin repeat domain 30B-like	-4.696919	down	-3.9171348	down
SND1-IT1	NR_027330	SND1 intronic transcript 1 (non-protein coding)	-2.7406774	down	-5.7121572	down
ZNF295-AS1	NR_027273	ZNF295 antisense RNA 1	-5.074827	down	-2.1244316	down

LINC00908		long intergenic non-protein coding RNA 908	-3.0775535	down	-2.149846	down
C9orf170	NM_001001709	chromosome 9 open reading frame 170	-4.1848717	down	-2.194299	down
KRT18	NM_000224	keratin 18	-2.6492693	down	-2.544059	down
LOC100133077	XR_110101	uncharacterized LOC100133077	-5.1168437	down	-2.663046	down
KRT24	NM_019016	keratin 24	-5.66145	down	-3.9864826	down
LOC101060578	AK097639	putative COBW domain-containing protein 7-like	-2.5687132	down	-2.050406	down
			-5.182995	down	-3.2997575	down
LYPD1	NM_144586	LY6/PLAUR domain containing 1	-5.2903824	down	-3.1973336	down
LOC100131257	NR_034022	zinc finger protein 655 pseudogene	-2.8151958	down	-2.0795376	down
ZP3	NM_007155	zona pellucida glycoprotein 3 (sperm receptor)	-2.2300262	down	-3.2205937	down
ELMOD3	NM_001135021	ELMO/CED-12 domain containing 3	-5.04395	down	-6.2250795	down
BBOX1	NM_003986	butyrobetaine (gamma), 2-oxoglutarate dioxygenase (gamma-butyrobetaine hydroxylase) 1	-3.7182384	down	-2.249721	down
			-3.3010452	down	-3.4104838	down
NRIP2	NM_031474	nuclear receptor interacting protein 2	-3.6821842	down	-3.1240985	down
PLEKHH3	NM_024927	pleckstrin homology domain containing, family H (with MyTH4 domain) member 3	-3.8838005	down	-6.09917	down
SPEG	NM_005876	SPEG complex locus	-4.8856554	down	-4.6148615	down
ELOVL3	NM_152310	ELOVL fatty acid elongase 3	-4.179019	down	-2.1939065	down
CYP11B1	NM_000497	cytochrome P450, family 11, subfamily B, polypeptide 1	-5.6661777	down	-4.0354156	down
			-5.2011156	down	-2.5169075	down
			-2.8676488	down	-2.4875305	down
			-3.9133577	down	-2.1222732	down
PRKCG	NM_002739	protein kinase C, gamma	-5.734045	down	-2.5764086	down
NEU2	NM_005383	sialidase 2 (cytosolic sialidase)	-2.9798942	down	-2.152409	down
			-3.2337716	down	-2.5985951	down
PSG1	NM_006905	pregnancy specific beta-1-glycoprotein 1	-4.9921613	down	-2.1910615	down
XLOC_010534			-4.276244	down	-3.053713	down
LINC00302	CU449054	long intergenic non-protein coding RNA 302	-3.6860738	down	-2.149412	down
DKKL1	NM_014419	dickkopf-like 1	-3.9595037	down	-2.145298	down
ACACB	NM_001093	acetyl-CoA carboxylase beta	-2.8339155	down	-2.1251402	down
C1orf140	NR_024236	uncharacterized LOC400804	-3.5248063	down	-5.214493	down
PPIAL4G	AK123006	peptidylprolyl isomerase A (cyclophilin A)-like 4G	-4.986513	down	-3.2630737	down
CLDN24	NM_001185149	claudin 24	-3.2661986	down	-2.027146	down
EFHC2	NM_025184	EF-hand domain (C-terminal) containing 2	-2.5841026	down	-2.1857426	down
FLJ45256	XR_172139	uncharacterized LOC400511	-5.4824586	down	-3.1493418	down
NUMB	AK055876	numb homolog (Drosophila)	-5.916553	down	-4.465653	down
ESYT3	NM_031913	extended synaptotagmin-like protein 3	-3.2355413	down	-2.027996	down
ANKRD1	NM_014391	ankyrin repeat domain 1 (cardiac muscle)	-3.6157227	down	-2.058246	down
LOC650157	XM_001727011	peptidyl-prolyl cis-trans isomerase A-like	-2.1183245	down	-2.1015105	down
GJA8	NM_005267	gap junction protein, alpha 8, 50kDa	-2.6307201	down	-2.649914	down
			-2.5484412	down	-2.8283353	down
OR5AS1	NM_001001921	olfactory receptor, family 5, subfamily AS, member 1	-9.57515	down	-6.8833327	down
FOXD2-AS1	NR_026878	FOXD2 antisense RNA 1 (head to head)	-2.1654174	down	-2.1788409	down
PRAMEF15	NM_001098376	PRAME family member 15	-5.546904	down	-3.3328235	down
	DA797466		-4.1117473	down	-3.3040235	down
EGFL6	NM_001167890	EGF-like-domain, multiple 6	-4.1612563	down	-2.9807382	down
KALRN	AK131379	kalirin, RhoGEF kinase	-8.337575	down	-7.722789	down
HSD17B2	NM_002153	hydroxysteroid (17-beta) dehydrogenase 2	-2.771075	down	-2.275334	down
RS1	NM_000330	retinoschisin 1	-5.631471	down	-2.5032353	down
TTLL13	NR_104604	tubulin tyrosine ligase-like family, member 13	-4.3395324	down	-2.9083288	down
SERPING1	NM_000062	serpin peptidase inhibitor, clade G (C1 inhibitor), member 1	-3.6206262	down	-4.2205234	down
NXN	BC104634	nucleoredoxin	-3.0354202	down	-2.1891823	down
C2orf27B	NM_214461	chromosome 2 open reading frame 27B	-6.6304526	down	-3.303043	down
LOC284561	XR_110828	uncharacterized LOC284561	-4.8970804	down	-4.23857	down
AOX1	NM_001159	aldehyde oxidase 1	-2.7608504	down	-2.0797276	down
GPRIN1	NM_052899	G protein regulated inducer of neurite outgrowth 1	-2.2356331	down	-2.8827758	down
			-5.0693965	down	-2.142824	down
	XM_001717040		-2.4956841	down	-2.1503267	down
KNCN	NM_001097611	kinocilin	-4.165843	down	-2.725823	down
LOC100128682	NR_040046	uncharacterized LOC100128682	-5.0672064	down	-2.454104	down
BTC	NM_001729	betacellulin	-4.2291036	down	-2.8819747	down
MLNR	NM_001507	motilin receptor	-2.1119905	down	-2.2022471	down

LINC00265	NR_026999	long intergenic non-protein coding RNA 265	-3.0890703	down	-10.263503	down
FAM46B	NM_052943	family with sequence similarity 46, member B	-2.7220135	down	-2.3069835	down
LOC100131242	AK124483	uncharacterized LOC100131242	-6.7647185	down	-3.5512571	down
KCNMA1	NM_001271520	potassium large conductance calcium-activated channel, subfamily M, alpha member 1	-4.62325	down	-2.2860222	down
C22orf24	NM_015372	chromosome 22 open reading frame 24	-5.1430025	down	-3.5211573	down
SLC2A12	NM_145176	solute carrier family 2 (facilitated glucose transporter), member 12	-2.7787275	down	-3.3910832	down
LONRF2	NM_198461	LON peptidase N-terminal domain and ring finger 2	-6.4311395	down	-3.2625039	down
XAGE1A	NM_001097592	X antigen family, member 1A	-5.9906077	down	-3.8157604	down
			-5.939781	down	-3.477825	down
KISS1	NM_002256	KiSS-1 metastasis-suppressor	-2.2105904	down	-2.3801632	down
LOC728613		programmed cell death 6 pseudogene	-4.78385	down	-4.9963565	down
SPATA13	AK092754	spermatogenesis associated 13	-3.8933823	down	-3.1779885	down
LIPG	NM_006033	lipase, endothelial	-2.362314	down	-2.2022915	down
MATN1	NM_002379	matrilin 1, cartilage matrix protein	-3.2768407	down	-2.150094	down
LINC00640	NR_038358	long intergenic non-protein coding RNA 640	-3.1292577	down	-2.2213464	down
LINC00537	XR_246700	long intergenic non-protein coding RNA 537	-2.5830874	down	-2.9399838	down
			-3.5642037	down	-2.74192	down
TNFAIP8L1	NM_001167942	tumor necrosis factor, alpha-induced protein 8-like 1	-6.1978087	down	-2.8801103	down
ATOH7	NM_145178	atonal homolog 7 (Drosophila)	-2.5730178	down	-3.0680246	down
IGFL4		IGF-like family member 4	-4.258593	down	-2.6725647	down
KRBA2	NM_213597	KRAB-A domain containing 2	-3.341646	down	-2.636628	down
			-6.3981156	down	-3.1327965	down
LOC654780	AK095331	SFPQ	-3.0009508	down	-2.1665819	down
LOC285758		uncharacterized LOC285758	-7.021153	down	-6.7411222	down
BEGAIN	NM_001159531	brain-enriched guanylate kinase-associated	-2.6326988	down	-2.8482418	down
IFITM5	NM_001025295	interferon induced transmembrane protein 5	-4.1292477	down	-3.7229087	down
			-2.8375366	down	-4.615257	down
SRPX	NM_006307	sushi-repeat containing protein, X-linked	-4.989093	down	-2.3670316	down
			-3.950959	down	-2.0965304	down
WWC2-AS2	NR_024008	WWC2 antisense RNA 2	-4.723164	down	-2.3429992	down
HMBOX1	XM_005273634	homeobox containing 1	-6.382482	down	-4.5809073	down
CDH24	NM_022478	cadherin 24, type 2	-5.361275	down	-8.422184	down
SIGLEC16	NR_002825	sialic acid binding Ig-like lectin 16 (gene/pseudogene)	-2.0913475	down	-2.8121698	down
ICMT	BC017037	isoprenylcysteine carboxyl methyltransferase	-2.5117805	down	-2.7361953	down
PRSS50	NM_013270	protease, serine, 50	-3.8746665	down	-2.275281	down
			-4.589399	down	-2.977569	down
			-4.056352	down	-2.0973783	down
FRMD4A	AK001072	FERM domain containing 4A	-5.396286	down	-5.5562153	down
RYR2	NM_001035	ryanodine receptor 2 (cardiac)	-3.1924555	down	-2.3227167	down
ANKMY2	NM_020319	ankyrin repeat and MYND domain containing 2	-2.4281106	down	-2.540638	down
			-2.1460063	down	-2.3498874	down
LOC101930210	XR_246852	protein capicua homolog	-3.987064	down	-3.8640091	down
PGC	NM_002630	progastricsin (pepsinogen C)	-3.061083	down	-3.787712	down
ANKRD33B		ankyrin repeat domain 33B	-4.4069405	down	-2.1035476	down
LOC392364	NR_040117	nuclear pore associated protein 1 pseudogene	-8.982032	down	-4.0242825	down
			-4.7029734	down	-4.641126	down
C19orf80	NM_018687	chromosome 19 open reading frame 80	-4.1604776	down	-2.9006255	down
PRELID2	NM_138492	PRELI domain containing 2	-2.774592	down	-2.567545	down
	AF130090		-2.1519656	down	-2.12819	down
	AK002210		-3.0624077	down	-2.419726	down
PARP10	NM_032789	poly (ADP-ribose) polymerase family, member 10	-2.3700228	down	-3.0117638	down
ENTPD1-AS1	NR_038444	ENTPD1 antisense RNA 1	-4.6143446	down	-2.0298798	down
	DA967691		-6.460211	down	-3.8911455	down
C1orf95	NM_001003665	chromosome 1 open reading frame 95	-4.16224	down	-2.8651948	down
PLAC4	NM_182832	placenta-specific 4	-4.465535	down	-2.899971	down
MUC22	NM_001198815	mucin 22	-3.7509608	down	-2.2997468	down
ASS1	NM_000050	argininosuccinate synthase 1	-7.2687473	down	-2.3587267	down
HSPA12B	NM_052970	heat shock 70kD protein 12B	-8.000544	down	-6.8582325	down
MREG	NM_018000	melanoregulin	-3.8753052	down	-2.5230293	down
AIM1L	NM_001039775	absent in melanoma 1-like	-2.3814204	down	-2.0896115	down
CLDN9	NM_020982	claudin 9	-2.284755	down	-2.3201125	down
	AK055281		-3.8784013	down	-6.856401	down

			-3.5241735	down	-3.6521168	down
RARB	NM_000965	retinoic acid receptor, beta	-2.4316323	down	-2.0805387	down
HOXA2	NM_006735	homeobox A2	-9.283564	down	-7.2102246	down
SCNN1D	NM_001130413	sodium channel, non-voltage-gated 1, delta subunit	-4.4984703	down	-2.8304255	down
	AK126599		-2.272024	down	-2.2630346	down
			-3.466794	down	-2.512548	down
ADH4	NM_000670	alcohol dehydrogenase 4 (class II), pi polypeptide	-5.3756394	down	-5.839934	down
LOC153811	AK021734	uncharacterized LOC153811	-2.1625905	down	-2.077881	down
ZNF750	NM_024702	zinc finger protein 750	-2.105438	down	-2.2620246	down
GDA	NM_004293	guanine deaminase	-5.3868537	down	-2.3238697	down
DKFZP547J0410	AL050263	DKFZP547J0410 protein	-6.3094077	down	-2.1034982	down
ANKRD20A5P	NR_040113	ankyrin repeat domain 20 family, member A5, pseudogene	-3.8878298	down	-3.301266	down
LOC100132474	AK125160	uncharacterized LOC100132474	-5.5846457	down	-4.3550954	down
LOC101928426	XM_005262504	basic salivary proline-rich protein 2-like	-2.4671354	down	-2.1240373	down
TEKT5	NM_144674	tektin 5	-3.534046	down	-2.1575022	down
RPL23AP7		ribosomal protein L23a pseudogene 7	-2.0044103	down	-4.298498	down
CYTH1	AK123894	cytohesin 1	-8.135434	down	-3.669139	down
C14orf23	NR_026731	chromosome 14 open reading frame 23	-3.1571982	down	-2.0129092	down
			-4.117132	down	-3.1985557	down
PLCE1	NM_016341	phospholipase C, epsilon 1	-5.704092	down	-4.449349	down
			-4.1643095	down	-2.488993	down
			-2.06069	down	-3.2373679	down
HOXD3	NM_006898	homeobox D3	-3.6895819	down	-4.82493	down
C16orf78	NM_144602	chromosome 16 open reading frame 78	-2.2827077	down	-2.8015893	down
			-3.9657881	down	-3.0303025	down
FAM95B1		family with sequence similarity 95, member B1	-6.9745016	down	-6.628003	down
LOC100996405	XM_003846604	uncharacterized LOC100996405	-4.227307	down	-3.8447871	down
TRIM49	NM_020358	tripartite motif containing 49	-4.4292817	down	-2.952903	down
CARD10	NM_014550	caspase recruitment domain family, member 10	-3.1858025	down	-2.5354111	down
CDK20	NM_001039803	cyclin-dependent kinase 20	-2.0132074	down	-2.9887044	down
ITGB1BP2	NM_012278	integrin beta 1 binding protein (melusin) 2	-5.0998955	down	-3.6831837	down
HILS1	NR_024193	histone linker H1 domain, spermatid-specific 1, pseudogene	-6.504197	down	-2.1705287	down
MAFIP	NR_046439	MAFF interacting protein (pseudogene)	-2.3858893	down	-4.0018263	down
FIGNL2	NM_001013690	fidgetin-like 2	-6.216129	down	-7.0376697	down
FLJ16126	BC130506	uncharacterized LOC645010	-3.0474324	down	-2.6852047	down
	AK094078		-3.32062	down	-2.536136	down
			-2.4806254	down	-2.242781	down
			-2.2267606	down	-2.1346881	down
			-5.5596623	down	-3.0330968	down
FLJ45079	NR_028337	FLJ45079 protein	-3.5942397	down	-2.9329846	down
FAM188B	NM_032222	family with sequence similarity 188, member B	-2.208694	down	-2.7789786	down
			-3.7954915	down	-2.2357159	down
TTC9B	NM_152479	tetratricopeptide repeat domain 9B	-2.3041391	down	-5.811311	down
	XM_005275749		-4.0413184	down	-2.5914798	down
			-3.9764905	down	-3.4983406	down
GJB1	NM_000166	gap junction protein, beta 1, 32kDa	-5.241464	down	-2.311607	down
C2CD2	AK129520	C2 calcium-dependent domain containing 2	-3.0305173	down	-3.1529033	down
	XR_243167		-4.9343033	down	-5.7813587	down
CACNG7	NM_031896	calcium channel, voltage-dependent, gamma subunit 7	-2.3234606	down	-2.189293	down
SEC14L3	NM_174975	SEC14-like 3 (S. cerevisiae)	-3.0333176	down	-2.3337786	down
RNF44	XM_005265842	ring finger protein 44	-2.6866064	down	-2.272156	down
AQP7P1		aquaporin 7 pseudogene 1	-3.6866508	down	-5.191415	down
			-4.472773	down	-2.1274254	down
EVPLL	NM_001145127	envoplakin-like	-2.6809144	down	-2.7900255	down
MYB	XM_005267001	v-myb avian myeloblastosis viral oncogene homolog	-6.1278763	down	-2.402128	down
	AY043127		-3.135213	down	-3.5069249	down
CYP3A7	NM_000765	cytochrome P450, family 3, subfamily A, polypeptide 7	-4.6630445	down	-2.7026408	down
SLC4A11	NM_032034	solute carrier family 4, sodium borate transporter, member 11	-2.8201563	down	-3.8291397	down
			-7.3692164	down	-6.0088816	down
TNR	NM_003285	tenascin R	-7.25473	down	-3.688181	down
CDH26	AF169690	cadherin 26	-6.338106	down	-2.5530965	down

WEE2	NM_001105558	WEE1 homolog 2 (S. pombe)	-5.4077764	down	-2.0671835	down
LOC283140	XR_247724	uncharacterized LOC283140	-2.933328	down	-2.5002172	down
STARD13	AY082592	StAR-related lipid transfer (START) domain containing 13	-4.6400976	down	-6.3124475	down
KRTAP22-1	BC101686	keratin associated protein 22-1	-2.7501624	down	-2.193338	down
HIP1	AY358103	huntingtin interacting protein 1	-2.891669	down	-4.580367	down
SPINT3	NM_006652	serine peptidase inhibitor, Kunitz type, 3	-2.2559807	down	-2.1773279	down
TRAIP	NM_005879	TRAF interacting protein	-3.5846562	down	-2.61517	down
			-2.6725955	down	-2.0892658	down
			-3.5203722	down	-2.3094718	down
			-4.4513683	down	-4.216011	down
			-6.562516	down	-3.1852612	down
RSG1	NM_030907	REM2 and RAB-like small GTPase 1	-3.4372394	down	-2.7162266	down
KATNAL2	NM_031303	katanin p60 subunit A-like 2	-7.3391366	down	-3.7840579	down
LRRC27	NM_001143757	leucine rich repeat containing 27	-2.0544841	down	-2.0007784	down
			-5.568514	down	-3.4921243	down
CASC5	NM_170589	cancer susceptibility candidate 5	-8.423703	down	-2.6278677	down
	XR_241567		-3.3718548	down	-3.7753386	down
LOC284628		uncharacterized LOC284628	-2.3500805	down	-2.0210927	down
			-3.7812335	down	-2.4951174	down
ZBTB46	NM_025224	zinc finger and BTB domain containing 46	-3.1657815	down	-2.4187608	down
OR4P4	NM_001004124	olfactory receptor, family 4, subfamily P, member 4	-3.9142623	down	-2.0618207	down
BPIFA1	NM_130852	BPI fold containing family A, member 1	-3.3350966	down	-2.1941106	down
			-4.8065295	down	-2.262546	down
ARHGAP33	AY044864	Rho GTPase activating protein 33	-8.100466	down	-3.8457866	down
			-3.883319	down	-2.1764355	down
JSRP1	NM_144616	junctional sarcoplasmic reticulum protein 1	-2.617098	down	-2.1416838	down
KRTAP21-1	NM_181619	keratin associated protein 21-1	-2.7263892	down	-2.2723818	down
DCDC2B	NM_001099434	doublecortin domain containing 2B	-2.467398	down	-3.0515325	down
			-8.121333	down	-2.458312	down
LANCL3	NM_198511	LanC lantibiotic synthetase component C-like 3 (bacterial)	-3.333469	down	-3.9324112	down
			-2.4194882	down	-2.147342	down
POTEA	NM_001005365	POTE ankyrin domain family, member A	-4.14527	down	-2.153182	down
FXYD5	AF177940	FXYD domain containing ion transport regulator 5	-2.6237252	down	-3.351646	down
ZNF833P	NR_028594	zinc finger protein 833, pseudogene	-3.313393	down	-3.2071152	down
			-2.7475557	down	-3.576794	down
CD70	NM_001252	CD70 molecule	-4.5155106	down	-2.6214747	down
NIFK-AS1	NR_037857	NIFK antisense RNA 1	-3.7612462	down	-2.4693296	down
FUT5	NM_002034	fucosyltransferase 5 (alpha (1,3) fucosyltransferase)	-3.9949164	down	-5.0115747	down
LOC440910	NR_030728	uncharacterized LOC440910	-2.980566	down	-2.178331	down
LINC00910	AK127427	long intergenic non-protein coding RNA 910	-5.5718756	down	-2.6120749	down
	AK092728		-5.614942	down	-3.9423654	down
RDH8	NM_015725	retinol dehydrogenase 8 (all-trans)	-2.50793	down	-2.9215565	down
RBP5	NM_031491	retinol binding protein 5, cellular	-4.0440464	down	-3.0047588	down
LOC729800		ZP domain-containing protein LOC729800-like	-5.677417	down	-3.5357096	down
ASPN	NM_001193335	asporin	-4.8149104	down	-3.1003358	down
OSBPL1A	NM_080597	oxysterol binding protein-like 1A	-3.3080153	down	-2.3265214	down
FILIP1	NM_015687	filamin A interacting protein 1	-4.325328	down	-4.686489	down
CLCN2	NM_004366	chloride channel, voltage-sensitive 2	-2.1723602	down	-3.5596416	down
TUBB3	NM_006086	tubulin, beta 3 class III	-2.5607321	down	-2.9997253	down
PNPLA5	NM_138814	patatin-like phospholipase domain containing 5	-2.759226	down	-2.1336887	down
			-2.06931	down	-2.929605	down
SSC5D	NM_001144950	scavenger receptor cysteine rich domain containing (5 domains)	-2.9397655	down	-4.202606	down
			-3.31682	down	-2.1304648	down
PDE1C	NM_005020	phosphodiesterase 1C, calmodulin-dependent 70kDa	-3.563801	down	-2.0257459	down
TMEM254	NM_025125	transmembrane protein 254	-2.029224	down	-2.027727	down
STAR	NM_000349	steroidogenic acute regulatory protein	-2.4905252	down	-2.6234725	down
ZNF496	NM_032752	zinc finger protein 496	-2.0337863	down	-2.1253622	down
KCNK17	NM_031460	potassium channel, subfamily K, member 17	-2.0733569	down	-2.324354	down
RASGEF1C	NM_175062	RasGEF domain family, member 1C	-4.494135	down	-2.5676343	down
LINC00479	NR_027272	long intergenic non-protein coding RNA 479	-5.7776327	down	-4.0778666	down
LOC100506688	NR_104614	uncharacterized LOC100506688	-4.2683935	down	-2.596568	down

KRTAP2-3	NM_001165252	keratin associated protein 2-3	-2.3841586	down	-2.2337527	down
AKR1B15	NM_001080538	aldo-keto reductase family 1, member B15	-4.6705494	down	-2.1337974	down
ADAMTS16	NM_139056	ADAM metallopeptidase with thrombospondin type 1 motif, 16	-3.0361693	down	-3.2559235	down
RABEPK	AL832249	Rab9 effector protein with kelch motifs	-2.301904	down	-2.7702084	down
SLC4A10	NM_022058	solute carrier family 4, sodium bicarbonate transporter, member 10	-2.200645	down	-2.6917078	down
OXCT2	NM_022120	3-oxoacid CoA transferase 2	-2.7740047	down	-2.0726116	down
FAM101A	NM_181709	family with sequence similarity 101, member A	-6.9708815	down	-5.0124717	down
LOC101929847		golgin subfamily A member 2-like protein 3-like	-4.146473	down	-2.4807687	down
TRIP13	NM_004237	thyroid hormone receptor interactor 13	-3.2175422	down	-2.0441926	down
FAM184A	BX640728	family with sequence similarity 184, member A	-6.394074	down	-2.818335	down
			-7.104944	down	-6.231032	down
MCF2L	NM_024979	MCF.2 cell line derived transforming sequence-like	-3.2454245	down	-2.4111705	down
USP2-AS1	AK130019	USP2 antisense RNA 1 (head to head)	-3.144454	down	-2.4094837	down
			-4.0224037	down	-2.2229578	down
OBSCN	NM_052843	obscurin, cytoskeletal calmodulin and titin-interacting RhoGEF	-2.8314023	down	-2.9657788	down
			-4.04259	down	-2.9328835	down
			-6.907095	down	-7.210703	down
			-3.9782977	down	-2.40387	down
PRSS38	NM_183062	protease, serine, 38	-7.0407524	down	-4.308838	down
ZDHHC20	BC034944	zinc finger, DHHC-type containing 20	-5.1861024	down	-2.653478	down
INE1	NR_024616	inactivation escape 1 (non-protein coding)	-4.8058147	down	-2.4841604	down
DRD2	NM_000795	dopamine receptor D2	-4.1984577	down	-2.098226	down
LOC643327	AK096549	uncharacterized LOC643327	-3.9665527	down	-2.3396132	down
AQP4-AS1	NR_026908	AQP4 antisense RNA 1	-2.1082878	down	-2.2112362	down
HOXB13	NM_006361	homeobox B13	-5.8375278	down	-3.7711306	down
	AK126601		-2.9459722	down	-3.1251743	down
LOC400752	NR_024270	uncharacterized LOC400752	-3.2062156	down	-2.4061265	down
LOC100131043	AK124222	uncharacterized LOC100131043	-2.9896443	down	-3.286456	down
IMMT	NM_001100170	inner membrane protein, mitochondrial	-2.81435	down	-2.6078925	down
SCNN1G	NM_001039	sodium channel, non-voltage-gated 1, gamma subunit	-3.7129946	down	-2.1228693	down
			-5.527194	down	-3.530172	down
GFM1	AK022724	G elongation factor, mitochondrial 1	-2.9672923	down	-3.4136693	down
LINC00243	XR_159139	long intergenic non-protein coding RNA 243	-3.0639231	down	-2.547695	down
CNGA4	NM_001037329	cyclic nucleotide gated channel alpha 4	-5.126623	down	-2.4353645	down
TTTY5		testis-specific transcript, Y-linked 5 (non-protein coding)	-5.2447047	down	-3.821448	down
HSP90AB4P	NR_073415	heat shock protein 90kDa alpha (cytosolic), class B member 4, pseudogene	-2.23193	down	-2.056503	down
IL26	NM_018402	interleukin 26	-5.910685	down	-5.722428	down
FLJ41455	AK123449	uncharacterized LOC441441	-3.5164332	down	-3.128099	down
DIO2	NM_013989	deiodinase, iodothyronine, type II	-3.1742823	down	-2.5414531	down
AVPR1B	NM_000707	arginine vasopressin receptor 1B	-2.363095	down	-3.216929	down
			-2.5052896	down	-2.1526978	down
	DB053889		-3.6354737	down	-3.9369462	down
EBPL	NR_103802	emopamil binding protein-like	-3.90579	down	-3.1389692	down
LOC100128001	AK125720	uncharacterized LOC100128001	-6.692714	down	-5.13357	down
	AK095132		-6.0531936	down	-2.3890495	down
OR10G2	NM_001005466	olfactory receptor, family 10, subfamily G, member 2	-2.3035967	down	-3.315201	down
KRT8P41	NR_027713	keratin 8 pseudogene 41	-4.6306205	down	-2.871	down
MGC16275	NR_026914	uncharacterized protein MGC16275	-2.0273113	down	-2.3972409	down
HES2	BC012091	hes family bHLH transcription factor 2	-2.453654	down	-2.0149393	down
NPPC	NM_024409	natriuretic peptide C	-2.9862602	down	-2.2403631	down
LOC389831	NM_001242480	uncharacterized LOC389831	-6.8795314	down	-5.603441	down
XLOC_014512			-4.342132	down	-2.6453834	down
LINC00478	NR_027790	long intergenic non-protein coding RNA 478	-2.500025	down	-2.1834679	down
TMEM177	AK311734	transmembrane protein 177	-3.8832707	down	-2.816161	down
GOLGA6L9	NM_198181	golgin A6 family-like 9	-4.7038183	down	-2.6304502	down
OR2L2	NM_001004686	olfactory receptor, family 2, subfamily L, member 2	-4.306786	down	-2.3532882	down
POFUT1	NM_172236	protein O-fucosyltransferase 1	-2.7953544	down	-2.8667874	down
LINC00661		long intergenic non-protein coding RNA 661	-3.4698396	down	-2.3260567	down
			-2.2811232	down	-2.3419116	down
PARP1		poly (ADP-ribose) polymerase 1	-4.216585	down	-2.5167377	down

REXO1L2P	NR_003594	REX1, RNA exonuclease 1 homolog (S. cerevisiae)-like 2 (pseudogene)	-8.747213	down	-6.7877603	down
			-2.7141654	down	-3.81782	down
			-2.2623057	down	-3.6396644	down
LINC00634	NR_024355	long intergenic non-protein coding RNA 634	-4.4256353	down	-2.6002984	down
ITPK1-AS1	NR_002808	ITPK1 antisense RNA 1	-2.1100764	down	-3.1966882	down
CHN2		chimerin 2	-2.1069708	down	-3.3470228	down
KRTAP26-1	NM_203405	keratin associated protein 26-1	-2.9844346	down	-2.1406884	down
			-3.0447924	down	-2.2138312	down
			-3.0453627	down	-2.482147	down
STXBP5L	NM_014980	syntaxin binding protein 5-like	-4.401448	down	-2.0890753	down
FAM230C	XR_246982	family with sequence similarity 230, member C	-3.9510932	down	-5.4164205	down
MED15P9	BC036597	mediator complex subunit 15 pseudogene 9	-3.685006	down	-3.2048664	down
ZNF503-AS2	NR_024421	ZNF503 antisense RNA 2	-4.7052536	down	-2.3035274	down
LOC650293	NM_001040071	seven transmembrane helix receptor	-3.7780097	down	-6.540019	down
	BC040619		-6.669156	down	-5.4872065	down
TAF5L		TAF5-like RNA polymerase II, p300/CBP-associated factor (PCAF)-associated factor, 65kDa	-3.4269083	down	-2.3891914	down
MGC39545	XR_110535	uncharacterized LOC403312	-3.1313677	down	-2.1363657	down
B4GALNT2	NM_153446	beta-1,4-N-acetyl-galactosaminyl transferase 2	-3.1871336	down	-2.0449708	down
			-2.2826583	down	-2.1958797	down
POM121L8P	NR_024583	POM121 transmembrane nucleoporin-like 8 pseudogene	-5.051601	down	-7.818869	down
			-2.7276943	down	-2.0610745	down
CTPS2	NM_001144002	CTP synthase 2	-3.576919	down	-2.0952806	down
MROH2A		maestro heat-like repeat family member 2A	-2.824729	down	-2.594823	down
AKAP1	NM_003488	A kinase (PRKA) anchor protein 1	-6.781774	down	-6.650966	down
			-3.3637526	down	-3.730161	down
PAK3	NM_002578	p21 protein (Cdc42/Rac)-activated kinase 3	-7.558883	down	-3.365554	down
FBLN1	NM_006486	fibulin 1	-3.014256	down	-3.6865642	down
SPRR2E	NM_001024209	small proline-rich protein 2E	-3.9378178	down	-2.9355419	down
			-3.032016	down	-2.0585797	down
C2CD4B	NM_001007595	C2 calcium-dependent domain containing 4B	-2.3726068	down	-3.2585046	down
CHRNA1	NM_001039523	cholinergic receptor, nicotinic, alpha 1 (muscle)	-3.457899	down	-4.53592	down
CRHR1	NM_001145148	corticotropin releasing hormone receptor 1	-2.697257	down	-2.5290244	down
			-2.8683896	down	-2.354647	down
TAS2R30	NM_001097643	taste receptor, type 2, member 30	-4.41865	down	-2.4881923	down
VAX1	NM_001112704	ventral anterior homeobox 1	-4.890126	down	-3.3857303	down
			-5.7741895	down	-5.241445	down
			-3.9164226	down	-2.1355317	down
C6orf164	NR_026784	chromosome 6 open reading frame 164	-2.8960931	down	-2.4579763	down
ACAN	NM_001135	aggrecan	-3.3048954	down	-3.8159482	down
BCAS1	NM_003657	breast carcinoma amplified sequence 1	-3.4770348	down	-2.7943482	down
LOC101930210	XR_246852	protein capicua homolog	-5.241599	down	-6.458739	down
LRRTM4	NM_024993	leucine rich repeat transmembrane neuronal 4	-2.4008393	down	-2.0386956	down
	BC050023		-8.402515	down	-4.641401	down
LOC100128402	AK124574	uncharacterized LOC100128402	-2.4591472	down	-3.4809797	down
KRTAP5-5	NM_001001480	keratin associated protein 5-5	-3.1588116	down	-2.3037202	down
MAP3K10	NM_002446	mitogen-activated protein kinase kinase kinase 10	-2.6512883	down	-3.0484006	down
MAGEB10	NM_182506	melanoma antigen family B, 10	-5.0158195	down	-2.435996	down
	XM_005263847		-3.0371017	down	-2.6299403	down
DRGX		dorsal root ganglia homeobox	-2.583394	down	-2.05292	down
SHOX	NM_006883	short stature homeobox	-4.9061747	down	-4.3361726	down
	XR_243792		-4.3647003	down	-3.4306297	down
CXorf64	NM_001122716	chromosome X open reading frame 64	-2.4640534	down	-2.1719856	down
KRT26	NM_181539	keratin 26	-2.13939	down	-2.0922265	down
MUCL1	NM_058173	mucin-like 1	-3.6118934	down	-3.1079416	down
			-2.0161433	down	-2.6670797	down
PTN	NM_002825	pleiotrophin	-5.1150107	down	-2.4462154	down
PTPRH	NM_002842	protein tyrosine phosphatase, receptor type, H	-4.100824	down	-2.4729233	down
C17orf82	NM_203425	chromosome 17 open reading frame 82	-4.369473	down	-3.0048323	down
CSH1	NM_001317	chorionic somatomammotropin hormone 1 (placental lactogen)	-2.5124705	down	-3.4446065	down
OR6P1	NM_001160325	olfactory receptor, family 6, subfamily P, member 1	-5.5802674	down	-2.0816424	down
SERINC2	NM_178865	serine incorporator 2	-2.2564945	down	-2.1549928	down

PPP1R1C	NM_001080545	protein phosphatase 1, regulatory (inhibitor) subunit 1C	-2.3891208	down	-2.2610533	down
MAP3K13	NM_004721	mitogen-activated protein kinase kinase kinase 13	-3.7432306	down	-2.3992116	down
STAU2-AS1	NR_038406	STAU2 antisense RNA 1	-2.7688327	down	-2.489578	down
DEFB1	NM_005218	defensin, beta 1	-7.353411	down	-2.3869355	down
PDILT	NM_174924	protein disulfide isomerase-like, testis expressed	-7.540905	down	-4.672183	down
	XM_005276061		-2.3770661	down	-2.4421797	down
LOC729020	NM_001143909	rcRPE	-4.013231	down	-2.6608815	down
OTC	NM_000531	ornithine carbamoyltransferase	-2.5970876	down	-3.4451053	down
CAPN13	NM_144575	calpain 13	-2.9244206	down	-3.1888301	down
	BX648501		-2.4813714	down	-2.9855356	down
TMEM132E	NM_207313	transmembrane protein 132E	-3.091431	down	-4.284696	down
TRIM17	NM_016102	tripartite motif containing 17	-4.3472695	down	-3.620156	down
MBD3L1	NM_145208	methyl-CpG binding domain protein 3-like 1	-4.395825	down	-3.1835573	down
TP53TG3	NM_016212	TP53 target 3	-2.395882	down	-2.0361729	down
	AK024906		-4.0546193	down	-2.464693	down
AP1S2	XM_005274612	adaptor-related protein complex 1, sigma 2 subunit	-3.997316	down	-2.379591	down
ODF3B	NM_001014440	outer dense fiber of sperm tails 3B	-2.1032474	down	-3.0685577	down
	XR_245823		-3.6191506	down	-2.9917042	down
CYP46A1	AB209749	cytochrome P450, family 46, subfamily A, polypeptide 1	-4.347425	down	-2.2400594	down
			-2.9855435	down	-2.6587827	down
CT45A5	NM_001007551	cancer/testis antigen family 45, member A5	-4.472325	down	-8.094015	down
C6orf118	NM_144980	chromosome 6 open reading frame 118	-5.746527	down	-2.1295283	down
			-2.306612	down	-3.1050115	down
EMR4P	AY181245	egf-like module containing, mucin-like, hormone receptor-like 4 pseudogene	-2.3178992	down	-2.3522377	down
LOC100131303	NM_001282442	uncharacterized LOC100131303	-4.5085297	down	-3.6261485	down
LINC00328	AF172850	long intergenic non-protein coding RNA 328	-4.8891616	down	-3.3823345	down
C18orf64	NM_001243517	chromosome 18 open reading frame 64	-8.573052	down	-5.265971	down
OR4M1	NM_001005500	olfactory receptor, family 4, subfamily M, member 1	-3.0773025	down	-2.6218333	down
EPX	NM_000502	eosinophil peroxidase	-2.1059031	down	-2.1721497	down
			-3.5672867	down	-2.6959102	down
PLK1	NM_005030	polo-like kinase 1	-3.6753976	down	-2.1234255	down
IGSF21	NM_032880	immunoglobin superfamily, member 21	-2.9224963	down	-2.7440553	down
LOC152286	AL117431	uncharacterized LOC152286	-3.1030061	down	-2.150858	down
SELV	NM_182704	selenoprotein V	-3.1896558	down	-4.503737	down
			-2.7777936	down	-2.3124392	down
LRRC48	NM_031294	leucine rich repeat containing 48	-5.1789722	down	-3.5324757	down
CADPS	NM_183393	Ca++-dependent secretion activator	-2.484442	down	-2.1044981	down
			-3.1737974	down	-2.3382058	down
			-3.2957397	down	-4.4931135	down
	AK000950		-7.552501	down	-4.49499	down
OR51L1	NM_001004755	olfactory receptor, family 51, subfamily L, member 1	-2.1734262	down	-2.3236735	down
IAPP	NM_000415	islet amyloid polypeptide	-2.0420742	down	-2.7572558	down
TPO	NM_175721	thyroid peroxidase	-2.3773062	down	-2.2086618	down
ILDR1	NM_001199799	immunoglobulin-like domain containing receptor 1	-8.349081	down	-3.69501	down
PPP1R12B	NM_001167858	protein phosphatase 1, regulatory subunit 12B	-5.0467706	down	-2.428456	down
POM121L4P	NR_024592	POM121 transmembrane nucleoporin-like 4 pseudogene	-4.1517305	down	-6.7370973	down
			-7.83493	down	-3.391712	down
ALPK3	NM_020778	alpha-kinase 3	-2.720958	down	-3.1536317	down
HOXD12	NM_021193	homeobox D12	-4.8989153	down	-3.730177	down
SLC22A24	NM_001136506	solute carrier family 22, member 24	-3.0037184	down	-2.218759	down
CLEC4M	NM_001144911	C-type lectin domain family 4, member M	-7.802538	down	-3.3323767	down
PLXDC1	NM_020405	plexin domain containing 1	-2.7237802	down	-2.8136203	down
MMP2	NM_004530	matrix metallopeptidase 2 (gelatinase A, 72kDa gelatinase, 72kDa type IV collagenase)	-3.1940796	down	-3.0168788	down
NUMBL	AK096566	numb homolog (Drosophila)-like	-4.5448823	down	-2.4770176	down
RHO	NM_000539	rhodopsin	-2.9424348	down	-2.0070555	down
			-2.2462947	down	-2.1946433	down
LOC100128851	AK127423	uncharacterized LOC100128851	-2.8426902	down	-3.5658264	down
LYPD4	XM_005278383	LY6/PLAUR domain containing 4	-3.141229	down	-2.368074	down
XLOC_013472			-2.740107	down	-3.4043262	down
SIM1	NM_005068	single-minded family bHLH transcription factor 1	-4.73623	down	-3.516522	down
ERBB4	NM_005235	v-erb-b2 avian erythroblastic leukemia viral oncogene homolog 4	-2.403767	down	-3.0228953	down

TTC39A	AB007921	tetratricopeptide repeat domain 39A	-5.586702	down	-4.0860405	down
SSC5D	NM_001144950	scavenger receptor cysteine rich domain containing (5 domains)	-3.5083632	down	-3.9298322	down
TPTEP1		transmembrane phosphatase with tensin homology pseudogene 1	-2.0719025	down	-2.7495475	down
			-5.5728498	down	-2.1346862	down
C21orf62	NM_019596	chromosome 21 open reading frame 62	-2.4237745	down	-2.2702794	down
YY2	NM_206923	YY2 transcription factor	-3.6573117	down	-2.745018	down
SH2D4B	NM_207372	SH2 domain containing 4B	-3.4170883	down	-2.143373	down
KLK3		kallikrein-related peptidase 3	-3.3449035	down	-3.9587147	down
FAM95B1		family with sequence similarity 95, member B1	-3.175988	down	-2.1441925	down
SPATA31C2	NM_001166137	SPATA31 subfamily C, member 2	-4.9029975	down	-2.105384	down
WDR6	NM_018031	WD repeat domain 6	-3.4563015	down	-3.2026353	down
VTCN1	NM_024626	V-set domain containing T cell activation inhibitor 1	-3.4809265	down	-2.519322	down
	DA380926		-5.650798	down	-2.8633425	down
FZD10	NM_007197	frizzled family receptor 10	-3.1928272	down	-2.1467643	down
RSPO1	NM_001038633	R-spondin 1	-2.5024793	down	-2.2965157	down
PBOV1	NM_021635	prostate and breast cancer overexpressed 1	-3.575623	down	-2.8565507	down
DLL4	NM_019074	delta-like 4 (Drosophila)	-2.9784076	down	-2.6120486	down
TBX15	XM_005271161	T-box 15	-3.6806834	down	-2.572243	down
			-3.340373	down	-2.323868	down
			-2.8262095	down	-2.0393913	down
STXBP1	NM_003165	syntaxin binding protein 1	-5.8065495	down	-11.575024	down
PRSS35	NM_153362	protease, serine, 35	-4.6289682	down	-3.7475495	down
ARMC4	NM_018076	armadillo repeat containing 4	-3.9101305	down	-2.4722443	down
GPHA2	NM_130769	glycoprotein hormone alpha 2	-2.4099991	down	-2.9351585	down
	DQ323997		-2.2424254	down	-3.025131	down
XLOC_014512			-2.581114	down	-2.9747903	down
C11orf39	AK127362	chromosome 11 open reading frame 39	-2.8149347	down	-2.5082736	down
C9orf53	NR_024274	chromosome 9 open reading frame 53	-2.0028186	down	-2.208523	down
LOC390705		protein phosphatase 2, regulatory subunit B'', beta pseudogene	-5.679186	down	-3.3711836	down
FAM115A	NM_014719	family with sequence similarity 115, member A	-2.7608933	down	-2.575283	down
LOC100130027	XR_250425	uncharacterized LOC100130027	-3.599427	down	-2.3434985	down
GPR143	NM_000273	G protein-coupled receptor 143	-2.0673742	down	-2.0001326	down
			-2.1234512	down	-2.7141237	down
FFAR4	NM_181745	free fatty acid receptor 4	-2.6581173	down	-3.5376074	down
CCL20	NM_004591	chemokine (C-C motif) ligand 20	-3.3799648	down	-5.3729663	down
ARHGAP29	NM_004815	Rho GTPase activating protein 29	-5.012964	down	-2.3029828	down
LCE3A	NM_178431	late cornified envelope 3A	-4.960309	down	-4.215301	down
XLOC_005985			-4.1495547	down	-2.4659622	down
LINC00469	NR_027146	long intergenic non-protein coding RNA 469	-2.3934133	down	-3.5327144	down
PDZD2	XM_005248269	PDZ domain containing 2	-4.299657	down	-2.0075896	down
	AX721312		-2.8196726	down	-2.0176792	down
GHRH	NM_021081	growth hormone releasing hormone	-2.2639303	down	-2.4451466	down
FLJ44881	BC119774	FLJ44881	-2.2183788	down	-2.81897	down
	AK026312		-2.1538436	down	-3.3416808	down
			-4.838314	down	-3.2798095	down
			-4.710228	down	-2.6721013	down
			-4.021721	down	-3.3218708	down
FLJ42102		uncharacterized LOC399923	-3.9866443	down	-3.0024512	down
			-4.7367983	down	-2.5129466	down
	BE468260		-2.217405	down	-2.3224392	down
GDF5	NM_000557	growth differentiation factor 5	-2.519642	down	-2.5101643	down
			-3.1182075	down	-5.8776093	down
SARDH	NM_007101	sarcosine dehydrogenase	-2.5900073	down	-2.4933617	down
			-2.8365214	down	-2.2153018	down
KIF21B	NM_001252102	kinesin family member 21B	-2.734255	down	-2.2452161	down
TRPV1	NM_080706	transient receptor potential cation channel, subfamily V, member 1	-3.4843838	down	-4.1230664	down
			-3.2270882	down	-3.051634	down
POLM	AK092801	polymerase (DNA directed), mu	-4.4273906	down	-6.1459208	down
PGF	NM_002632	placental growth factor	-2.7537487	down	-4.1989174	down
HOGA1	AK094791	4-hydroxy-2-oxoglutarate aldolase 1	-4.9284415	down	-2.9133787	down

	BX350880		-5.1892653	down	-3.5348084	down
FARP2	NM_014808	FERM, RhoGEF and pleckstrin domain protein 2	-2.578871	down	-3.1892345	down
OR4C13	NM_001001955	olfactory receptor, family 4, subfamily C, member 13	-4.78674	down	-2.7805126	down
RPRM	NM_019845	reprimo, TP53 dependent G2 arrest mediator candidate	-4.356147	down	-3.009193	down
C17orf104	NM_001145080	chromosome 17 open reading frame 104	-3.279685	down	-2.2853482	down
C1orf110	XM_005245126	chromosome 1 open reading frame 110	-6.8540196	down	-2.212149	down
C11orf91	NM_001166692	chromosome 11 open reading frame 91	-3.4042659	down	-3.2158985	down
LINC00112		long intergenic non-protein coding RNA 112	-6.3439503	down	-3.696973	down
RAB3C	NM_138453	RAB3C, member RAS oncogene family	-2.401721	down	-2.6397803	down
SLC26A1	NM_022042	solute carrier family 26 (anion exchanger), member 1	-4.0565376	down	-2.140201	down
			-4.2524214	down	-4.1089587	down
PRRG3		proline rich Gla (G-carboxyglutamic acid) 3 (transmembrane)	-4.379238	down	-4.0721474	down
LOC440518	NR_033899	golgin A2 pseudogene	-2.174021	down	-2.8164687	down
RAET1E	NM_139165	retinoic acid early transcript 1E	-3.5835888	down	-2.6243067	down
			-2.762051	down	-2.1660411	down
			-6.106518	down	-3.950393	down
CLIC5	NM_016929	chloride intracellular channel 5	-2.3120713	down	-2.813209	down
PCDH8	NM_002590	protocadherin 8	-4.5068326	down	-2.3611255	down
	XR_252185		-4.242264	down	-5.463425	down
CCDC13-AS1	XR_245087	CCDC13 antisense RNA 1	-4.3199215	down	-2.1194508	down
	BC069683		-3.6916153	down	-3.0403626	down
			-5.2074895	down	-2.120838	down
SLC19A3		solute carrier family 19 (thiamine transporter), member 3	-4.854747	down	-2.89024	down
MYCT1	NM_025107	myc target 1	-2.9153717	down	-3.1453035	down
LINC00656		long intergenic non-protein coding RNA 656	-2.3716185	down	-2.534903	down
AK4	NM_001005353	adenylate kinase 4	-4.4906936	down	-4.0319743	down
XLOC_014512			-3.80788	down	-2.2034023	down
RTN4RL1	NM_178568	reticulon 4 receptor-like 1	-2.713394	down	-2.4262722	down
SEPN1	NM_020451	selenoprotein N, 1	-2.4997265	down	-2.6878133	down
RBMY1B	NM_001006121	RNA binding motif protein, Y-linked, family 1, member B	-2.3554819	down	-2.2463882	down
			-7.0104017	down	-5.8662295	down
			-2.2990892	down	-2.0807428	down
			-3.154178	down	-3.1178367	down
	XM_005276061		-3.4237015	down	-3.155046	down
LOC100130442	AK131364	uncharacterized LOC100130442	-5.190087	down	-4.7179313	down
L2HGDH	NM_024884	L-2-hydroxyglutarate dehydrogenase	-4.540999	down	-3.6938467	down
			-3.6504707	down	-3.3330872	down
MUC12	NM_001164462	mucin 12, cell surface associated	-4.1237965	down	-2.31573	down
LUZP4	NM_016383	leucine zipper protein 4	-3.0300114	down	-2.3685749	down
	DA825750		-4.3914485	down	-2.1884382	down
ABHD12B	NM_001206673	abhydrolase domain containing 12B	-2.6606398	down	-2.2948968	down
C7	NM_000587	complement component 7	-4.9730897	down	-4.3760676	down
			-3.6345408	down	-2.1417725	down
			-3.1546032	down	-2.1340694	down
MAGEB16	NM_001099921	melanoma antigen family B, 16	-10.212704	down	-3.1281996	down
LINC00298		long intergenic non-protein coding RNA 298	-3.4533699	down	-3.0976439	down
TERF1	AK128828	telomeric repeat binding factor (NIMA-interacting) 1	-3.042686	down	-2.939499	down
CRHR1	AK124894	corticotropin releasing hormone receptor 1	-4.5187407	down	-2.3067143	down
FLJ32955		uncharacterized protein FLJ32955	-2.799315	down	-2.436399	down
	AK127494		-2.3571315	down	-2.2153118	down
C8orf66	AL834492	chromosome 8 open reading frame 66	-5.2120147	down	-5.335049	down
GGNBP1	NR_028361	gametogenetin binding protein 1 (pseudogene)	-2.5650973	down	-2.296233	down
FLJ45482	AK127393	uncharacterized LOC645566	-7.6402583	down	-5.4473696	down
ZNF221	NM_013359	zinc finger protein 221	-3.153823	down	-2.2103717	down
RIBC1	NM_144968	RIB43A domain with coiled-coils 1	-3.9644072	down	-2.9950383	down
CNTN6	NM_014461	contactin 6	-3.1018984	down	-2.5210204	down
			-2.4810603	down	-2.5062912	down
CALML6	NM_138705	calmodulin-like 6	-3.336499	down	-2.3114624	down
APOC4	NM_001646	apolipoprotein C-IV	-2.4776707	down	-2.2767167	down
OR13H1	NM_001004486	olfactory receptor, family 13, subfamily H, member 1	-2.3443534	down	-2.2315154	down
	XR_242443		-3.4162261	down	-2.558823	down
LRRC3B	NM_052953	leucine rich repeat containing 3B	-2.6797447	down	-2.304273	down

IGSF11	NM_152538	immunoglobulin superfamily, member 11	-2.2304337	down	-2.044415	down
LOC442122	AK128759	uncharacterized LOC442122	-2.4087815	down	-3.912592	down
ZFHX2	NM_033400	zinc finger homeobox 2	-3.499969	down	-2.0057359	down
SNX29	BC029857	sorting nexin 29	-3.4163837	down	-2.550967	down
TAF7L	NM_024885	TAF7-like RNA polymerase II, TATA box binding protein (TBP)-associated factor, 50kDa	-3.3501267	down	-4.237088	down
LOC100130219	BC039168	uncharacterized LOC100130219	-3.1230786	down	-2.995904	down
C11orf94	NM_001080446	chromosome 11 open reading frame 94	-2.98685	down	-2.2475994	down
LOC100128644	AY358240	LMNE6487	-2.9948626	down	-2.3389635	down
LINC01020	NR_026994	long intergenic non-protein coding RNA 1020	-4.014717	down	-2.321115	down
SLC22A7	NM_153320	solute carrier family 22 (organic anion transporter), member 7	-4.0024796	down	-2.5640233	down
DCN	NM_001920	decorin	-2.5660083	down	-2.601472	down
XLOC_004837			-5.287984	down	-2.8931408	down
LOC400682	XM_003846486	zinc finger protein 100-like	-3.8529625	down	-3.1319497	down
	AK057937		-5.594234	down	-3.1844053	down
LAMA3	BC093406	laminin, alpha 3	-3.9153767	down	-2.5552838	down
RLIM	NM_183353	ring finger protein, LIM domain interacting	-3.060859	down	-2.0225472	down
TBXA2R	NM_001060	thromboxane A2 receptor	-3.645117	down	-2.2187736	down
ZNRF3	NM_001206998	zinc and ring finger 3	-6.217153	down	-4.799175	down
	BC094703		-2.2877982	down	-2.0489757	down
ST8SIA6-AS1	AK127982	ST8SIA6 antisense RNA 1	-3.586174	down	-2.0305517	down
MYOZ3	NM_133371	myozenin 3	-3.4981747	down	-3.1188323	down
			-2.7736974	down	-2.9971247	down
			-3.0250106	down	-2.0324469	down
EPM2A	NM_001018041	epilepsy, progressive myoclonus type 2A, Lafora disease (laforin)	-2.6596923	down	-2.4355779	down
ZNF365	NM_199451	zinc finger protein 365	-9.490078	down	-3.8610733	down
			-5.0509114	down	-2.2933345	down
PSG10P	L14723	pregnancy specific beta-1-glycoprotein 10, pseudogene	-4.9226646	down	-5.8618336	down
PCDH10	NM_032961	protocadherin 10	-3.8108032	down	-2.4410305	down
OTOG		otogelin	-2.6265247	down	-2.0625606	down
BCOR		BCL6 corepressor	-3.6131258	down	-2.0180728	down
LOC100130433	AK096255	uncharacterized LOC100130433	-6.477724	down	-2.5512693	down
			-11.2422495	down	-7.8933845	down
DUOXA2	BX537581	dual oxidase maturation factor 2	-3.2095342	down	-2.217342	down
			-7.351093	down	-3.0303755	down
PKD2L1	NM_001253837	polycystic kidney disease 2-like 1	-2.2718992	down	-2.9406118	down
OR2A5	NM_012365	olfactory receptor, family 2, subfamily A, member 5	-4.145408	down	-3.9019973	down
IVL	NM_005547	involucrin	-3.2350159	down	-3.6366699	down
C18orf15	AK055900	chromosome 18 open reading frame 15	-2.8002374	down	-4.244676	down
ATP2A1	NM_173201	ATPase, Ca++ transporting, cardiac muscle, fast twitch 1	-2.146978	down	-2.6509368	down
C10orf67	NM_153714	chromosome 10 open reading frame 67	-2.6981645	down	-2.49525	down
	DA292495		-3.3290203	down	-4.4575987	down
KLF17	NM_173484	Kruppel-like factor 17	-5.9575186	down	-2.9071062	down
			-2.7443027	down	-2.0764837	down
LINC00471	NR_024079	long intergenic non-protein coding RNA 471	-2.6475132	down	-2.558882	down
SRRM2	NM_016333	serine/arginine repetitive matrix 2	-2.7734127	down	-2.4846544	down
HIC2	NM_015094	hypermethylated in cancer 2	-2.6893122	down	-3.6411602	down
ARHGEF25	NM_182947	Rho guanine nucleotide exchange factor (GEF) 25	-4.45104	down	-2.2068572	down
ACVR2B-AS1	NR_028389	ACVR2B antisense RNA 1	-2.1485991	down	-2.6750498	down
CENPM	NM_001002876	centromere protein M	-2.6285555	down	-3.0034392	down
	AK126822		-3.216857	down	-2.144103	down
LOC100127974	AK096395	uncharacterized LOC100127974	-2.5793755	down	-2.3793402	down
SNAR-A3	NR_024214	small ILF3/NF90-associated RNA A3	-3.394051	down	-2.610968	down
ARTN	NM_057090	artemin	-3.0175884	down	-2.9977477	down
DRAXIN	NM_198545	dorsal inhibitory axon guidance protein	-5.2025223	down	-4.297741	down
GOLGA8I	NR_024074	golgin A8 family, member I	-2.3765383	down	-2.3570118	down
KERA	NM_007035	keratocan	-6.805399	down	-2.166617	down
CCDC18	NM_206886	coiled-coil domain containing 18	-2.667565	down	-2.0145316	down
			-5.041713	down	-5.5404215	down
TREM2	NM_018965	triggering receptor expressed on myeloid cells 2	-3.880465	down	-2.5101337	down
OR51V1	NM_001004760	olfactory receptor, family 51, subfamily V, member 1	-5.2598615	down	-3.1856377	down

CSPG4P8	NR_033579	chondroitin sulfate proteoglycan 4 pseudogene 8	-3.605421	down	-2.7088027	down
SRGAP1	NM_020762	SLIT-ROBO Rho GTPase activating protein 1	-3.2719216	down	-5.9805984	down
LOC100128184	AK128032	uncharacterized LOC100128184	-2.6046724	down	-3.726653	down
SPATA31E1	NM_178828	SPATA31 subfamily E, member 1	-2.5855255	down	-3.0858095	down
PAQR9	NM_198504	progestin and adipoQ receptor family member IX	-4.246932	down	-2.3362849	down
	BC137370		-2.8928747	down	-3.0560966	down
			-3.5755098	down	-3.1110375	down
C12orf42	NR_103526	chromosome 12 open reading frame 42	-2.9332204	down	-2.0324252	down
OR2Z1	NM_001004699	olfactory receptor, family 2, subfamily Z, member 1	-7.3849187	down	-4.651697	down
KRTAP5-7	NM_001012503	keratin associated protein 5-7	-4.1390243	down	-3.689695	down
AKR1C1		aldo-keto reductase family 1, member C1	-2.3489442	down	-2.9251955	down
KIAA1804	NM_032435	mixed lineage kinase 4	-2.1933222	down	-3.1814885	down
			-3.1517398	down	-3.0949423	down
PRSS37	NM_001008270	protease, serine, 37	-3.6511247	down	-2.7350087	down
IL19	NM_153758	interleukin 19	-2.3829544	down	-2.0626311	down
MS4A4E	XM_005274415	membrane-spanning 4-domains, subfamily A, member 4E	-4.7889643	down	-2.944113	down
HNF1A	X71347	HNF1 homeobox A	-4.2920446	down	-9.948551	down
PRDM5	NM_018699	PR domain containing 5	-2.4535525	down	-2.291478	down
FGL1	NM_201553	fibrinogen-like 1	-3.2281563	down	-3.7432191	down
ACBD7	NM_001039844	acyl-CoA binding domain containing 7	-4.7633204	down	-3.5007224	down
OR7E91P		olfactory receptor, family 7, subfamily E, member 91 pseudogene	-2.2479303	down	-2.177752	down
LOC101930277	XM_005276001	putative speedy protein-like protein LOC442572-like	-2.3543677	down	-3.9875796	down
			-4.580545	down	-3.7671514	down
	DW451783		-2.31998	down	-3.93076	down
			-3.0771558	down	-2.5405533	down
SLC12A1	NM_000338	solute carrier family 12 (sodium/potassium/chloride transporter), member 1	-2.9304106	down	-2.1636744	down
			-2.7537467	down	-4.634769	down
			-11.223335	down	-5.431114	down
			-3.9416976	down	-4.826088	down
SYTL4	AL832596	synaptotagmin-like 4	-7.2147017	down	-3.0938442	down
TRIM66	NM_014818	tripartite motif containing 66	-2.8489277	down	-2.0310817	down
TSSK6	NM_032037	testis-specific serine kinase 6	-2.6091082	down	-3.1816342	down
MGC45800	NR_027107	uncharacterized LOC90768	-3.9954557	down	-2.265468	down
GPX3	NM_002084	glutathione peroxidase 3 (plasma)	-8.080547	down	-26.24971	down
			-3.0391965	down	-2.26418	down
	XM_005262351		-3.0273724	down	-2.187194	down
PGF	AK023843	placental growth factor	-2.0445035	down	-3.2633703	down
GNG4	NM_001098722	guanine nucleotide binding protein (G protein), gamma 4	-4.072524	down	-2.2099357	down
WT1-AS	NR_023920	WT1 antisense RNA	-4.723758	down	-2.638942	down
	AK095662		-4.8174334	down	-3.7249205	down
THRSP	NM_003251	thyroid hormone responsive	-9.411306	down	-2.5471523	down
NPY5R	NM_006174	neuropeptide Y receptor Y5	-2.752537	down	-2.4194074	down
LINC00994		long intergenic non-protein coding RNA 994	-2.2048903	down	-2.8703406	down
			-3.8704274	down	-2.5611365	down
KCNG1	BC006367	potassium voltage-gated channel, subfamily G, member 1	-3.8978465	down	-2.251751	down
ADRA2B	NM_000682	adrenoceptor alpha 2B	-3.3958566	down	-2.0081847	down
ANKRD36B	NM_025190	ankyrin repeat domain 36B	-4.0414047	down	-2.42802	down
	AK097428		-2.9957068	down	-2.2840698	down
			-8.298018	down	-8.08274	down
DDC	NM_001242890	dopa decarboxylase (aromatic L-amino acid decarboxylase)	-4.1380014	down	-2.8398564	down
LINC00862	NR_040064	long intergenic non-protein coding RNA 862	-3.6854162	down	-3.9664595	down
			-8.106635	down	-4.1982193	down
			-3.607722	down	-5.6128917	down
FAM222A	NM_032829	family with sequence similarity 222, member A	-2.377456	down	-2.464742	down
MGC24103	BC020879	uncharacterized MGC24103	-3.3975537	down	-3.5006068	down
GIGYF2		GRB10 interacting GYF protein 2	-6.8904552	down	-2.1976812	down
CNTFR-AS1	NR_024369	CNTFR antisense RNA 1	-4.012094	down	-2.0007253	down
			-3.3043	down	-2.2938197	down
			-7.126149	down	-3.697126	down
CRHR1	NM_001256299	corticotropin releasing hormone receptor 1	-2.9646633	down	-3.3979523	down

ADCK3	XM_005273202	aarF domain containing kinase 3	-2.765031	down	-2.3748455	down
M1AP	NM_001281295	meiosis 1 associated protein	-4.565583	down	-5.0430565	down
SMIM17		small integral membrane protein 17	-7.0270543	down	-3.9596071	down
GRM4	NM_000841	glutamate receptor, metabotropic 4	-4.9132304	down	-3.4541683	down
CNBD1	NM_173538	cyclic nucleotide binding domain containing 1	-2.362943	down	-2.9723847	down
MFSD2B	NM_001080473	major facilitator superfamily domain containing 2B	-2.8333333	down	-2.5988963	down
			-5.6871257	down	-3.6310484	down
FAM95B1	NR_026759	family with sequence similarity 95, member B1	-2.4547598	down	-6.888975	down
FOXL1	NM_005250	forkhead box L1	-2.908986	down	-2.4489553	down
ASS1	NM_000050	argininosuccinate synthase 1	-2.0016015	down	-4.2798576	down
MSTO1	BC070067	misato 1, mitochondrial distribution and morphology regulator	-4.7585	down	-3.1386647	down
			-6.852509	down	-8.059803	down
PLAC9		placenta-specific 9	-2.2458434	down	-2.4713573	down
SMARCA1	NM_003069	SWI/SNF related, matrix associated, actin dependent regulator of chromatin, subfamily a, member 1	-2.820417	down	-2.0854108	down
			-3.4437118	down	-2.085765	down
SPACA6P	NR_024330	sperm acrosome associated 6, pseudogene	-2.147981	down	-2.171319	down
GABRB3	NR_103801	gamma-aminobutyric acid (GABA) A receptor, beta 3	-2.6119237	down	-2.5747182	down
LOC340335	AK074459	uncharacterized LOC340335	-2.2451448	down	-2.6379824	down
BRD7		bromodomain containing 7	-5.857923	down	-8.3824215	down
			-4.160587	down	-5.0996275	down
CETN1	NM_004066	centrin, EF-hand protein, 1	-3.3830988	down	-2.6012702	down
	AY358259		-3.4036396	down	-2.1031651	down
LINC00612	AK123808	long intergenic non-protein coding RNA 612	-3.347819	down	-2.3952613	down
CREG2	NM_153836	cellular repressor of E1A-stimulated genes 2	-2.2474577	down	-3.1792367	down
CPS1-IT1	NR_002763	CPS1 intronic transcript 1 (non-protein coding)	-4.651172	down	-2.132304	down
			-2.8417528	down	-3.6120808	down
			-4.688848	down	-4.3502536	down
CROCCP2		ciliary rootlet coiled-coil, rootletin pseudogene 2	-2.5046911	down	-2.0799756	down
LOC648691	NR_027426	uncharacterized LOC648691	-3.170918	down	-2.7376802	down
LOC100129213	NR_038419	uncharacterized LOC100129213	-5.7021146	down	-4.172857	down
			-2.2922685	down	-2.5002108	down
GCNT7	NM_080615	glucosaminyl (N-acetyl) transferase family member 7	-4.09372	down	-2.0400364	down
			-4.4009466	down	-2.563053	down
FAM230C	XR_246982	family with sequence similarity 230, member C	-5.1188145	down	-3.6988819	down
LRRC37A3	NM_199340	leucine rich repeat containing 37, member A3	-2.4184704	down	-3.8995996	down
TEX40	NM_001039496	testis expressed 40	-2.0131922	down	-2.8640788	down
			-2.3689635	down	-2.521992	down
CCNB3	NM_033031	cyclin B3	-2.3457255	down	-2.4409332	down
			-7.226682	down	-6.421004	down
NPAP1	NM_018958	nuclear pore associated protein 1	-3.0788836	down	-5.384669	down
LOC284395	NR_040029	uncharacterized LOC284395	-6.3845477	down	-3.5200908	down
MOBP	NM_182935	myelin-associated oligodendrocyte basic protein	-2.612194	down	-2.4987159	down
RNF151	NM_174903	ring finger protein 151	-10.789865	down	-6.9431434	down
	DB321672		-3.9226203	down	-5.995757	down
XIRP1	NM_194293	xin actin-binding repeat containing 1	-2.5284197	down	-2.0768394	down
TRIM15	NM_033229	tripartite motif containing 15	-2.9695067	down	-2.6229405	down
	DB452078		-8.392802	down	-5.68079	down
ATMIN		ATM interactor	-2.5728643	down	-3.0260386	down
CHRDL1	NM_145234	chordin-like 1	-7.965218	down	-4.456384	down
CIB4	NM_001029881	calcium and integrin binding family member 4	-2.6833458	down	-2.0584834	down
	AF176921		-3.7699955	down	-2.4161296	down
CAND1	NM_018448	cullin-associated and neddylation-dissociated 1	-2.1629581	down	-2.76554	down
LOC728208	AK055890	uncharacterized LOC728208	-2.373086	down	-2.0081418	down
NWD1	NM_001007525	NACHT and WD repeat domain containing 1	-4.7681046	down	-2.9914217	down
	XR_246457		-3.7747784	down	-3.6855512	down
BMP8B	NM_001720	bone morphogenetic protein 8b	-4.392157	down	-3.4113386	down
			-3.600906	down	-2.8334656	down
C6orf132	NM_001164446	chromosome 6 open reading frame 132	-2.3015475	down	-2.0614069	down
IGF2-AS	NR_028044	IGF2 antisense RNA	-2.4300961	down	-2.2078357	down
			-3.8739939	down	-2.2797542	down
DLGAP1	BC040718	discs, large (Drosophila) homolog-associated protein 1	-4.467252	down	-2.4041307	down
IGLON5	NM_001101372	IgLON family member 5	-4.6648974	down	-4.5871277	down

CXorf36	NM_176819	chromosome X open reading frame 36	-4.2681413	down	-3.6356258	down
			-6.3634233	down	-3.9116843	down
			-3.2045245	down	-4.4178777	down
LINC01060		long intergenic non-protein coding RNA 1060	-2.7237144	down	-2.3773453	down
ECM2	NM_001197296	extracellular matrix protein 2, female organ and adipocyte specific	-3.7958162	down	-2.1066232	down
TMED7-TICAM2	NM_001164469	TMED7-TICAM2 readthrough	-2.6871846	down	-4.454804	down
CCL13	NM_005408	chemokine (C-C motif) ligand 13	-2.5201604	down	-2.7365208	down
CNTN1	NM_001256064	contactin 1	-3.4734068	down	-2.6254642	down
TMEM136	NM_174926	transmembrane protein 136	-5.311514	down	-3.2309813	down
HES2	NM_019089	hes family bHLH transcription factor 2	-3.4782894	down	-2.1838522	down
OR5M1	NM_001004740	olfactory receptor, family 5, subfamily M, member 1	-3.393951	down	-2.0258088	down
LOC643669	NM_001243212	uncharacterized LOC643669	-4.8111906	down	-2.9784377	down
			-2.0588372	down	-2.2057874	down
NTM	NM_001144058	neurotrimin	-2.9576633	down	-3.3258417	down
	AK090395		-3.5862362	down	-2.8501523	down
ZNF735	NM_001159524	zinc finger protein 735	-2.1558645	down	-2.6174467	down
			-7.364551	down	-3.1889184	down
CD276	NM_001024736	CD276 molecule	-3.558426	down	-3.9181426	down
KIR3DL3	NM_153443	killer cell immunoglobulin-like receptor, three domains, long cytoplasmic tail, 3	-12.905526	down	-5.4592304	down
SLC52A1	NM_017986	solute carrier family 52 (riboflavin transporter), member	-2.6768174	down	-3.277146	down
KCNK10	NM_021161	potassium channel, subfamily K, member 10	-2.8726737	down	-3.266088	down
LOC101059918	NM_001282484	golgin subfamily A member 8R-like	-3.9089081	down	-6.7563615	down
DNAH10OS	AK127211	dynein, axonemal, heavy chain 10 opposite strand	-2.8059027	down	-2.3963795	down
HPN-AS1	NR_024561	HPN antisense RNA 1	-4.2889986	down	-3.4931052	down
FAM180B	NM_001164379	family with sequence similarity 180, member B	-3.263672	down	-3.0560126	down
SNTG1		syntrophin, gamma 1	-3.6184735	down	-4.626544	down
LOC400043	NR_026656	uncharacterized LOC400043	-2.574002	down	-2.0180123	down
LOC100128437	AK126077	uncharacterized LOC100128437	-3.787349	down	-3.705672	down
ARHGAP42	NM_152432	Rho GTPase activating protein 42	-4.6884327	down	-5.0928216	down
TAF1B		TATA box binding protein (TBP)-associated factor, RNA polymerase I, B, 63kDa	-3.7659035	down	-2.3717206	down
LOC440047	CN273640	uncharacterized LOC440047	-5.813673	down	-3.7307885	down
HIST2H3A	NM_001005464	histone cluster 2, H3a	-5.252526	down	-4.079533	down
			-3.1482422	down	-4.8730435	down
			-4.9207687	down	-3.8382444	down
	AA489744		-4.0394135	down	-3.2260072	down
VAPB	NM_004738	VAMP (vesicle-associated membrane protein)-associated protein B and C	-3.3189611	down	-2.6637015	down
C10orf112	XM_003403619	chromosome 10 open reading frame 112	-9.1042795	down	-5.223857	down
	XR_243598		-2.7269614	down	-3.6086152	down
PDLIM2	NM_176871	PDZ and LIM domain 2 (mystique)	-2.8918436	down	-2.5667872	down
TACC2	NM_206862	transforming, acidic coiled-coil containing protein 2	-3.9553227	down	-2.6267865	down
SCXA	NM_001008271	scleraxis homolog A (mouse)	-2.3026268	down	-2.4852164	down
DLK2	NM_206539	delta-like 2 homolog (Drosophila)	-2.6934922	down	-2.4141169	down
RGMB	NM_001012761	repulsive guidance molecule family member b	-2.54233	down	-2.0258934	down
CCBL2	NM_001008661	cysteine conjugate-beta lyase 2	-3.892778	down	-4.073024	down
LOC645321	XR_245489	uncharacterized LOC645321	-6.129499	down	-7.913347	down
			-4.215071	down	-2.8316476	down
ZNF556	NM_024967	zinc finger protein 556	-4.1390405	down	-4.8221555	down
			-7.0757327	down	-3.5852954	down
LOC400927	NR_002821	TPTE and PTEN homologous inositol lipid phosphatase pseudogene	-3.1335623	down	-2.7839723	down
			-4.7952833	down	-3.0954583	down
KRTAP4-8	NM_031960	keratin associated protein 4-8	-2.1826358	down	-2.6519606	down
LINC00371	NR_102432	long intergenic non-protein coding RNA 371	-3.407904	down	-2.1707833	down
LINC00671	NR_027254	long intergenic non-protein coding RNA 671	-2.2122674	down	-2.6735916	down
DNAJC5B	NM_033105	DnaJ (Hsp40) homolog, subfamily C, member 5 beta	-4.672535	down	-2.5006452	down
BAGE	NM_001187	B melanoma antigen	-2.5002224	down	-2.2087772	down
			-2.190112	down	-2.5467663	down
SPINK14	NM_001001325	serine peptidase inhibitor, Kazal type 14 (putative)	-2.410096	down	-2.6004455	down
LOC442122	AK128759	uncharacterized LOC442122	-2.7732742	down	-3.5038638	down
OR5H1	NM_001005338	olfactory receptor, family 5, subfamily H, member 1	-2.4639056	down	-3.2964878	down
TBC1D26		TBC1 domain family, member 26	-4.6548347	down	-2.5070555	down

OR51M1	NM_001004756	olfactory receptor, family 51, subfamily M, member 1	-6.921062	down	-2.374214	down
PTGR2	NM_152444	prostaglandin reductase 2	-6.3909965	down	-8.441116	down
LOC100506514	AK124637	uncharacterized LOC100506514	-10.0932045	down	-5.316796	down
			-2.2071943	down	-4.2262087	down
EFR3B	AB023170	EFR3 homolog B (S. cerevisiae)	-2.170626	down	-5.4527555	down
			-2.93619	down	-2.6616418	down
ANKRD19P	AK292218	ankyrin repeat domain 19, pseudogene	-4.0869055	down	-2.4562414	down
	BX404796		-8.677917	down	-6.1796365	down
EBF2	NM_022659	early B-cell factor 2	-3.2187676	down	-4.952757	down
			-2.0403483	down	-2.0120137	down
			-5.1649723	down	-4.454665	down
LINC00312	NR_024065	long intergenic non-protein coding RNA 312	-2.5148447	down	-2.5898385	down
			-2.3364172	down	-3.0176022	down
	AK124642		-2.5255454	down	-2.088488	down
KRT18P55	NR_028334	keratin 18 pseudogene 55	-2.5229087	down	-3.00817	down
ZNF503-AS1	NR_038223	ZNF503 antisense RNA 1	-5.666863	down	-3.3589785	down
LOC100130849		phosphorylase kinase, gamma 1 (muscle) pseudogene	-2.5456324	down	-2.6875086	down
ZDHHC11	NM_024786	zinc finger, DHHC-type containing 11	-2.9321234	down	-2.5308278	down
RABL2B	BC020495	RAB, member of RAS oncogene family-like 2B	-5.261373	down	-3.8184621	down
	DB153536		-2.2824883	down	-2.5775142	down
ADAMTSL4-AS1	AK127688	ADAMTSL4 antisense RNA 1	-4.0250177	down	-4.0490417	down
AADACL3	NM_001103170	arylacetamide deacetylase-like 3	-7.033656	down	-4.7020364	down
REXO1L2P	NR_003594	REX1, RNA exonuclease 1 homolog (S. cerevisiae)-like 2 (pseudogene)	-3.4867666	down	-3.3646264	down
TRIM61	NM_001012414	tripartite motif containing 61	-3.1146066	down	-2.6063843	down
	XR_243187		-4.0085664	down	-2.9335191	down
RBM43	NM_198557	RNA binding motif protein 43	-2.7332804	down	-2.1906834	down
LOC284379		solute carrier family 7 (cationic amino acid transporter, y+ system), member 3 pseudogene	-2.574183	down	-2.6562598	down
	XM_005244923		-3.68567	down	-2.043585	down
LOC100996291	NR_073178	uncharacterized LOC100996291	-2.192945	down	-2.3191981	down
			-4.8397064	down	-9.558157	down
CCL25	NM_005624	chemokine (C-C motif) ligand 25	-4.4605064	down	-2.8125806	down
STK32A	NM_145001	serine/threonine kinase 32A	-3.9841325	down	-2.125329	down
LOC100128988	AK023614	uncharacterized LOC100128988	-5.826838	down	-2.5201375	down
OR3A4P	NR_024128	olfactory receptor, family 3, subfamily A, member 4 pseudogene	-2.4522014	down	-2.0444016	down
LOC100132005	AK092662	uncharacterized LOC100132005	-3.9472644	down	-2.2618313	down
MIR143HG	NR_027180	MIR143 host gene (non-protein coding)	-2.0151367	down	-2.3195164	down
SLC39A5	NM_001135195	solute carrier family 39 (zinc transporter), member 5	-4.9817257	down	-2.177772	down
OR51D1	NM_001004751	olfactory receptor, family 51, subfamily D, member 1	-2.6888237	down	-2.7234256	down
ZSCAN5D	XM_003403711	zinc finger and SCAN domain containing 5D	-4.5405416	down	-2.763367	down
AANAT	NM_001166579	aralkylamine N-acetyltransferase	-3.5853052	down	-4.4548736	down
SPATA4	NM_144644	spermatogenesis associated 4	-3.9177687	down	-2.0024173	down
UPK1A	NM_007000	uroplakin 1A	-2.2895222	down	-3.35515	down
			-3.8948188	down	-2.079903	down
SPPL2B	NM_152988	signal peptide peptidase like 2B	-2.820456	down	-2.3410263	down
OPA1	NM_130831	optic atrophy 1 (autosomal dominant)	-3.345311	down	-2.2762234	down
C20orf166-AS1	NR_033263	C20orf166 antisense RNA 1	-3.3013394	down	-3.677217	down
			-2.2027879	down	-2.2614367	down
HTR6	NM_000871	5-hydroxytryptamine (serotonin) receptor 6, G protein-coupled	-3.94895	down	-4.801688	down
LOC729732	XR_247131	uncharacterized LOC729732	-3.3206744	down	-5.959142	down
GOLGA6L1	NM_001001413	golgin A6 family-like 1	-5.487814	down	-3.3031168	down
OR5C1	NM_001001923	olfactory receptor, family 5, subfamily C, member 1	-2.1553092	down	-2.0499384	down
CD300LG	NM_145273	CD300 molecule-like family member g	-2.7944899	down	-4.8576713	down
CD37	XR_243973	CD37 molecule	-4.721688	down	-2.8741121	down
XLOC_014512			-2.4976802	down	-2.7772305	down
HOXB3	NM_002146	homeobox B3	-2.8945665	down	-3.075062	down
			-2.676337	down	-2.4572413	down
TCTE1	NM_182539	t-complex-associated-testis-expressed 1	-2.521798	down	-2.600335	down
RUSC1-AS1	NM_001039517	RUSC1 antisense RNA 1	-2.4845698	down	-4.7683234	down
LOC344887		NmrA-like family domain containing 1 pseudogene	-2.2412922	down	-2.1797147	down
CEND1	NM_016564	cell cycle exit and neuronal differentiation 1	-2.2966251	down	-2.1468523	down

SYCN	NM_001080468	syncollin	-2.8738527	down	-4.126801	down
			-5.363066	down	-2.6321282	down
TCP10L	NM_144659	t-complex 10-like	-3.8079617	down	-2.1274908	down
LOC285084		uncharacterized LOC285084	-6.156386	down	-3.2387424	down
GATA4	XM_005272387	GATA binding protein 4	-3.3302603	down	-3.4079113	down
L2HGDH	NM_024884	L-2-hydroxyglutarate dehydrogenase	-3.8875659	down	-7.3986907	down
IKZF2	NM_001079526	IKAROS family zinc finger 2 (Helios)	-2.4134467	down	-2.096539	down
FOXB2	NM_001013735	forkhead box B2	-3.9257894	down	-2.2824314	down
LINC00162		long intergenic non-protein coding RNA 162	-4.4838343	down	-2.2422283	down
LOC400891		chromosome 14 open reading frame 166B pseudogene	-2.215716	down	-3.673029	down
DMBT1	NM_007329	deleted in malignant brain tumors 1	-5.848115	down	-5.0211787	down
TTBK1	NM_032538	tau tubulin kinase 1	-8.653743	down	-3.3152597	down
			-2.904501	down	-4.1136	down
CA10	NM_020178	carbonic anhydrase X	-4.0328526	down	-2.1008806	down
PTCH1	AB214500	patched 1	-3.7899678	down	-5.1449695	down
KLK13	AY923173	kallikrein-related peptidase 13	-4.9105315	down	-2.6436265	down
TNFRSF13C	NM_052945	tumor necrosis factor receptor superfamily, member 13C	-2.1515656	down	-2.2379334	down
SLC22A7	NM_153320	solute carrier family 22 (organic anion transporter), member 7	-4.192265	down	-3.2391326	down
			-2.8490677	down	-3.094894	down
LINC00337	NR_103534	long intergenic non-protein coding RNA 337	-4.958702	down	-2.6796117	down
			-2.231278	down	-2.2449286	down
OTOP3	NM_178233	otopetrin 3	-3.9969406	down	-2.1958504	down
	AY461701		-4.7512646	down	-3.6513202	down
PLEKHN1	NM_032129	pleckstrin homology domain containing, family N member 1	-3.2920194	down	-4.4371614	down
TNP1	NM_003284	transition protein 1 (during histone to protamine replacement)	-7.639208	down	-3.239564	down
LINC00521	NR_024182	long intergenic non-protein coding RNA 521	-6.8597307	down	-13.302271	down
PREX2	NM_025170	phosphatidylinositol-3,4,5-trisphosphate-dependent Rac exchange factor 2	-2.065082	down	-2.5224254	down
NTRK3	XR_253443	neurotrophic tyrosine kinase, receptor, type 3	-4.179228	down	-3.1561337	down
			-8.691348	down	-6.5622582	down
PDPN	NM_198389	podoplanin	-3.0143769	down	-2.4606566	down
GOLGA8O	NM_001277308	golgin A8 family, member O	-4.9697156	down	-6.2678037	down
PDE4D	NM_001197218	phosphodiesterase 4D, cAMP-specific	-2.9013636	down	-3.2914772	down
			-4.2725863	down	-2.5822186	down
SALL4	NM_020436	spalt-like transcription factor 4	-5.617824	down	-7.585921	down
	AK128018		-3.326546	down	-2.0155284	down
PCDH10	NM_020815	protocadherin 10	-10.423427	down	-3.8829024	down
			-7.27371	down	-4.033067	down
			-2.78899	down	-2.1923654	down
MUC2	NM_002457	mucin 2, oligomeric mucus/gel-forming	-2.0494304	down	-2.0623684	down
NME9	NM_178130	NME/NM23 family member 9	-2.343076	down	-2.1936576	down
LOC283038		uncharacterized LOC283038	-5.1055355	down	-3.2461724	down
YIPF7	NM_182592	Yip1 domain family, member 7	-4.312422	down	-2.478259	down
HMCN1	NM_031935	hemicentin 1	-2.1529458	down	-4.2775693	down
H2AFB1	NM_001017990	H2A histone family, member B1	-2.5806627	down	-2.1886191	down
OR8H2	NM_001005200	olfactory receptor, family 8, subfamily H, member 2	-4.1839037	down	-6.4763327	down
C2orf27A	NM_013310	chromosome 2 open reading frame 27A	-2.3645375	down	-2.1131563	down
SLC25A16	BC001407	solute carrier family 25 (mitochondrial carrier; Graves disease autoantigen), member 16	-3.2903755	down	-4.07135	down
FLG	NM_002016	filaggrin	-6.6835027	down	-4.5268593	down
CREB3L3	NM_032607	cAMP responsive element binding protein 3-like 3	-7.3778715	down	-5.5571895	down
LOC283335	NR_033854	uncharacterized LOC283335	-3.906171	down	-2.1808505	down
ERBB3	NM_001005915	v-erb-b2 avian erythroblastic leukemia viral oncogene homolog 3	-3.073997	down	-2.7754562	down
GPR123	NM_001083909	G protein-coupled receptor 123	-2.199137	down	-2.4919996	down
HIST1H2APS1		histone cluster 1, H2a, pseudogene 1	-2.4418077	down	-2.6281953	down
TPTE2P3	NR_002793	transmembrane phosphoinositide 3-phosphatase and tensin homolog 2 pseudogene 3	-6.196592	down	-2.5212119	down
SNCAIP	NM_005460	synuclein, alpha interacting protein	-2.5305371	down	-2.3968954	down
			-10.0941515	down	-12.246122	down
FOXA1		forkhead box A1	-5.672315	down	-3.9074488	down
OR51E1	NM_152430	olfactory receptor, family 51, subfamily E, member 1	-4.7580957	down	-2.1475463	down
			-3.8761833	down	-3.3969452	down

ANKRD20A12P	NR_046228	ankyrin repeat domain 20 family, member A12, pseudogene	-4.617865	down	-3.2308414	down
SLC5A6	NM_021095	solute carrier family 5 (sodium/multivitamin and iodide cotransporter), member 6	-2.6141648	down	-2.4534147	down
PDXK	XM_005261201	pyridoxal (pyridoxine, vitamin B6) kinase	-2.4855683	down	-2.0311298	down
FGF17	NM_003867	fibroblast growth factor 17	-4.12194	down	-4.1033063	down
CFLAR	NM_001202519	CASP8 and FADD-like apoptosis regulator	-4.2781434	down	-4.6703625	down
			-3.527747	down	-2.7066426	down
RP1L1	AK127545	retinitis pigmentosa 1-like 1	-3.0141788	down	-2.7269533	down
OR1F1	NM_012360	olfactory receptor, family 1, subfamily F, member 1	-5.768367	down	-4.514842	down
MRGPRD	NM_198923	MAS-related GPR, member D	-6.7391796	down	-3.5890746	down
LRRN4	NM_152611	leucine rich repeat neuronal 4	-2.4337735	down	-2.4662676	down
C1QTNF7	NM_031911	C1q and tumor necrosis factor related protein 7	-2.0753133	down	-2.151715	down
ZNF812	NM_001199814	zinc finger protein 812	-2.3612852	down	-2.6769469	down
	XR_109680		-3.2096179	down	-2.4871452	down
LOC399829		uncharacterized LOC399829	-3.523806	down	-3.4007292	down
NRN1	NM_016588	neuritin 1	-4.3750463	down	-2.3697023	down
NDP	NM_000266	Norrie disease (pseudoglioma)	-2.4819515	down	-2.5220702	down
XLOC_014512			-4.9803357	down	-6.0992327	down
NRG1	NM_004495	neuregulin 1	-3.4265642	down	-3.5246947	down
TTLL9	NM_001008409	tubulin tyrosine ligase-like family, member 9	-3.3337328	down	-3.0298066	down
			-2.87328	down	-3.7075272	down
BEST3	NM_001282614	bestrophin 3	-3.2675202	down	-4.591873	down
GSG1	NM_001080554	germ cell associated 1	-3.887381	down	-4.001791	down
			-4.4213104	down	-3.6939983	down
			-5.0280814	down	-2.902639	down
MLPH	NM_024101	melanophilin	-3.6302767	down	-2.6889625	down
COL5A1	AK057231	collagen, type V, alpha 1	-3.1718087	down	-4.067668	down
			-5.963965	down	-3.7325935	down
GRAMD2	NM_001012642	GRAM domain containing 2	-2.6142683	down	-2.04428	down
DMC1	NM_007068	DNA meiotic recombinase 1	-3.1878057	down	-2.5993614	down
ANKMY1	NM_017844	ankyrin repeat and MYND domain containing 1	-2.2135456	down	-3.5635881	down
GNRHR	NM_000406	gonadotropin-releasing hormone receptor	-2.5192702	down	-2.2511566	down
DNM1P46	NR_003260	DNM1 pseudogene 46	-2.924564	down	-3.9727767	down
			-2.277879	down	-2.5086474	down
PLSCR4	NM_020353	phospholipid scramblase 4	-3.4526606	down	-2.0200844	down
YEATS2	AK090720	YEATS domain containing 2	-3.4195857	down	-2.1613255	down
PTGR1	NM_012212	prostaglandin reductase 1	-3.185148	down	-2.6007707	down
			-3.5763938	down	-3.0213652	down
ATE1	NM_001001976	arginyltransferase 1	-2.4992554	down	-2.19486	down
TMEM174	NM_153217	transmembrane protein 174	-5.639762	down	-5.9497805	down
FLJ25363	NM_001145553	uncharacterized LOC401082	-2.058923	down	-2.0607967	down
			-9.519212	down	-4.476083	down
			-2.1686904	down	-3.219986	down
ZNF3	NM_017715	zinc finger protein 3	-2.2520313	down	-4.0247526	down
BRINP1	BC021560	bone morphogenetic protein/retinoic acid inducible neural-specific 1	-4.0490446	down	-2.245867	down
C8orf44-SGK3	NM_001204173	C8orf44-SGK3 readthrough	-2.279959	down	-2.4135082	down
			-2.1664078	down	-2.2181242	down
CRMP1	NM_001014809	collapsin response mediator protein 1	-3.3279607	down	-4.3283453	down
XLOC_014512			-3.7703242	down	-2.093263	down
LOC728208	AK055890	uncharacterized LOC728208	-3.6769822	down	-4.2462087	down
	XR_241667		-3.223834	down	-2.7122898	down
SUPT20HL2	NM_001136233	suppressor of Ty 20 homolog (S. cerevisiae)-like 2	-4.5437694	down	-3.7483592	down
NPAS1	NM_002517	neuronal PAS domain protein 1	-2.8871007	down	-2.5409536	down
PODNL1	NM_024825	podocan-like 1	-6.30966	down	-5.9561896	down
GPR115	NM_153838	G protein-coupled receptor 115	-2.6663744	down	-2.7746212	down
DND1	NM_194249	DND microRNA-mediated repression inhibitor 1	-2.372499	down	-2.0638418	down
	AK125712		-2.8168895	down	-3.7789106	down
CDH16	NM_004062	cadherin 16, KSP-cadherin	-6.998267	down	-2.9937773	down
LOC100132014	AK092445	uncharacterized LOC100132014	-4.0977426	down	-3.343256	down
LOC644093	XR_133298	hCG2040054	-2.9958692	down	-2.7517478	down
ABCA9	NM_080283	ATP-binding cassette, sub-family A (ABC1), member 9	-4.275162	down	-2.8953872	down
			-2.0994916	down	-2.2386808	down

TCL6	NR_028288	T-cell leukemia/lymphoma 6 (non-protein coding)	-8.836517	down	-3.5812922	down
FARP1	NM_005766	FERM, RhoGEF (ARHGEF) and pleckstrin domain protein 1 (chondrocyte-derived)	-3.7099974	down	-2.2835436	down
			-4.882639	down	-3.2970088	down
RCL1		RNA terminal phosphate cyclase-like 1	-3.1823862	down	-3.0049343	down
DPP6	NM_001039350	dipeptidyl-peptidase 6	-4.87151	down	-4.514639	down
FOXP3	NM_014009	forkhead box P3	-2.1174169	down	-2.0533419	down
LOC100270679	NR_038394	uncharacterized LOC100270679	-3.4841127	down	-2.6754577	down
TMEM89	NM_001008269	transmembrane protein 89	-3.989943	down	-2.730447	down
GALNT9	NM_021808	UDP-N-acetyl-alpha-D-galactosamine:polypeptide N-acetylgalactosaminyltransferase 9 (GalNAc-T9)	-3.5364873	down	-2.3163168	down
SLC19A3	NM_025243	solute carrier family 19 (thiamine transporter), member 3	-3.0739653	down	-2.0669942	down
TMEM235	NM_001204210	transmembrane protein 235	-5.142528	down	-10.200586	down
			-3.6666253	down	-4.1268883	down
PRODH	BC036534	proline dehydrogenase (oxidase) 1	-3.3388712	down	-4.231664	down
VPS37A	AL834189	vacuolar protein sorting 37 homolog A (S. cerevisiae)	-2.1635778	down	-2.7135425	down
TRHDE-AS1	NR_026836	TRHDE antisense RNA 1	-3.8931842	down	-2.1587815	down
	AW593215		-2.0717487	down	-2.9488413	down
GHRLOS	NR_073566	ghrelin opposite strand/antisense RNA	-2.3212717	down	-2.9478412	down
LOC100144602	NR_034138	uncharacterized LOC100144602	-4.896772	down	-3.4381387	down
	DA797466		-2.9358544	down	-4.107947	down
RNF165	NM_152470	ring finger protein 165	-3.496437	down	-2.897544	down
MORN1	NM_024848	MORN repeat containing 1	-2.4638755	down	-4.779135	down
USHBP1	NM_031941	Usher syndrome 1C binding protein 1	-7.139853	down	-5.4821634	down
GOLGA6A	NM_001038640	golgin A6 family, member A	-5.4780416	down	-3.289856	down
SCARA5	NM_173833	scavenger receptor class A, member 5 (putative)	-2.2436392	down	-3.1757426	down
ASIP	NM_001672	agouti signaling protein	-3.0855982	down	-3.1825237	down
			-3.2393532	down	-2.0611525	down
SPRR2B	NM_001017418	small proline-rich protein 2B	-3.6214142	down	-2.376864	down
LINC00087	NR_024493	long intergenic non-protein coding RNA 87	-3.4455636	down	-2.7784214	down
FRG2	NM_001005217	FSHD region gene 2	-2.1598742	down	-3.2779982	down
LYZL2	NM_183058	lysozyme-like 2	-3.7891939	down	-2.972997	down
			-3.6120558	down	-3.2739453	down
PNMA6A	NM_032882	paraneoplastic Ma antigen family member 6A	-2.8800113	down	-2.3811274	down
C8orf37	NM_177965	chromosome 8 open reading frame 37	-2.3259041	down	-2.026593	down
TSPEAR-AS1	NR_103707	TSPEAR antisense RNA 1	-3.1262767	down	-3.9372993	down
SLC26A7	NM_052832	solute carrier family 26 (anion exchanger), member 7	-6.679923	down	-3.6869466	down
			-4.819006	down	-5.557467	down
SLCO4C1	AF119865	solute carrier organic anion transporter family, member 4C1	-2.5227711	down	-2.5298479	down
MPPED1	NM_001044370	metallophosphoesterase domain containing 1	-9.362119	down	-5.874022	down
DBIL5P2	NR_036635	diazepam binding inhibitor-like 5 pseudogene 2	-3.343624	down	-3.0367873	down
MED1	NM_004774	mediator complex subunit 1	-3.148989	down	-4.8572793	down
GUCY2D	NM_000180	guanylate cyclase 2D, membrane (retina-specific)	-3.742484	down	-2.2980378	down
	AK026667		-5.012495	down	-3.575991	down
	CD694834		-3.5655062	down	-6.289534	down
CASS4	NM_001164115	Cas scaffolding protein family member 4	-2.152909	down	-2.1462932	down
SLC2A1-AS1	AK056786	SLC2A1 antisense RNA 1	-2.7799091	down	-3.794238	down
BLACE	NR_103545	B-cell acute lymphoblastic leukemia expressed	-2.6555748	down	-2.6677935	down
SLC9A7	NM_032591	solute carrier family 9, subfamily A (NHE7, cation proton antiporter 7), member 7	-2.1925597	down	-2.1704664	down
CCBE1	NM_133459	collagen and calcium binding EGF domains 1	-2.2427454	down	-2.804313	down
OPN5	NM_181744	opsin 5	-3.1091917	down	-3.137884	down
INADL	AJ001306	InaD-like (Drosophila)	-3.5576234	down	-2.2827427	down
CCDC40	NM_001243342	coiled-coil domain containing 40	-2.184633	down	-2.201744	down
LOC389602	AK124321	uncharacterized LOC389602	-4.4012833	down	-2.602459	down
	BX648392		-3.1091304	down	-2.781433	down
CSPG4P8	NR_033579	chondroitin sulfate proteoglycan 4 pseudogene 8	-4.6197977	down	-3.6204724	down
ZNF717	NM_001128223	zinc finger protein 717	-3.7600822	down	-2.0979207	down
TSGA10	NM_025244	testis specific, 10	-3.5671353	down	-2.0500958	down
LOC101929256	XR_242748	uncharacterized LOC101929256	-10.446433	down	-4.113459	down
DEFB121	NM_001171832	defensin, beta 121	-10.808161	down	-5.5644264	down
TRIM36	NM_001017397	tripartite motif containing 36	-4.4509544	down	-2.3733952	down
LRIT2	NM_001284223	leucine-rich repeat, immunoglobulin-like and transmembrane domains 2	-2.6727815	down	-2.9736469	down

	BC029571		-2.869104	down	-3.9679463	down
TRIM53AP	NR_028346	tripartite motif containing 53A, pseudogene	-6.196078	down	-2.2950032	down
ARHGEF33	NM_001145451	Rho guanine nucleotide exchange factor (GEF) 33	-4.192055	down	-4.3038073	down
BPIFC	NM_174932	BPI fold containing family C	-4.952355	down	-2.704431	down
URAHP	NR_027335	urate (hydroxyiso-) hydrolase, pseudogene	-3.1641784	down	-2.9379911	down
PEX26	NM_001199319	peroxisomal biogenesis factor 26	-2.643714	down	-2.410133	down
HYAL4	NM_012269	hyaluronoglucosaminidase 4	-4.994242	down	-2.448535	down
ANGPTL4	NM_139314	angiopoietin-like 4	-4.4138093	down	-2.9992814	down
SLC38A4	NM_018018	solute carrier family 38, member 4	-3.1320074	down	-3.050788	down
RAB19	NM_001008749	RAB19, member RAS oncogene family	-4.1937647	down	-5.7299867	down
ZNF320	AK307375	zinc finger protein 320	-3.2322986	down	-4.9440103	down
LY6K	NM_017527	lymphocyte antigen 6 complex, locus K	-4.3846498	down	-3.219032	down
	XR_253355		-2.8110096	down	-2.912551	down
FAT2	NM_001447	FAT atypical cadherin 2	-2.0005474	down	-2.0715022	down
			-3.2356954	down	-2.9836261	down
FLJ40039	AK097358	uncharacterized LOC647662	-7.326995	down	-7.3407874	down
ASRGL1	NM_001083926	asparaginase like 1	-5.146012	down	-2.5058482	down
LOC100130207	XR_245244	uncharacterized LOC100130207	-3.2457929	down	-2.7351577	down
	A25493		-2.0449417	down	-4.0344954	down
LOC100128402	AK124574	uncharacterized LOC100128402	-3.227346	down	-2.0488076	down
SIX1	NM_005982	SIX homeobox 1	-5.4981327	down	-6.201456	down
XLOC_014512			-7.9634647	down	-6.688405	down
TICRR	NM_152259	TOPBP1-interacting checkpoint and replication regulator	-3.413	down	-2.2031682	down
ANP32A	AK127498	acidic (leucine-rich) nuclear phosphoprotein 32 family, member A	-2.9449587	down	-2.457489	down
RDX	NM_001260492	radixin	-2.7580304	down	-3.0268786	down
HTATSF1P2	NR_033884	HIV-1 Tat specific factor 1 pseudogene 2	-4.959301	down	-2.7865615	down
IL21R-AS1	NR_037158	IL21R antisense RNA 1	-2.1568296	down	-2.1284125	down
			-3.0915384	down	-3.9829996	down
C15orf59	NM_001039614	chromosome 15 open reading frame 59	-5.095286	down	-3.7594361	down
TNC	NM_002160	tenascin C	-2.8688912	down	-3.2533302	down
MAP1LC3C	NM_001004343	microtubule-associated protein 1 light chain 3 gamma	-4.9354134	down	-2.7137017	down
SDCBP2	NM_080489	syndecan binding protein (syntenin) 2	-3.2117202	down	-4.48174	down
			-2.073658	down	-2.434551	down
			-3.0981166	down	-2.793236	down
ETV3	NM_001145312	ets variant 3	-5.5510178	down	-3.0406468	down
LPA	NM_005577	lipoprotein, Lp(a)	-2.8777912	down	-2.0264592	down
ERC2	NM_015576	ELKS/RAB6-interacting/CAST family member 2	-3.2305107	down	-2.3619015	down
TMEM52	NM_178545	transmembrane protein 52	-2.8768456	down	-5.5612707	down
TEX35		testis expressed 35	-5.3534317	down	-2.3776698	down
	BC025775		-3.1246405	down	-3.3646216	down
			-2.6537619	down	-4.065298	down
PTPRQ	NM_001145026	protein tyrosine phosphatase, receptor type, Q	-4.2192388	down	-3.1092496	down
SEMA6C	NM_030913	sema domain, transmembrane domain (TM), and cytoplasmic domain, (semaphorin) 6C	-4.878546	down	-5.4617624	down
LOC100130954	NR_034016	uncharacterized LOC100130954	-5.480152	down	-6.72411	down
			-4.794201	down	-3.1852295	down
DIO3	NM_001362	deiodinase, iodothyronine, type III	-5.387029	down	-4.2080483	down
LOC729159	NM_001282301	UPF0607 protein ENSP00000381418-like	-2.4853585	down	-2.254708	down
IFNA4	NM_021068	interferon, alpha 4	-3.0964239	down	-2.1906059	down
			-3.3436778	down	-3.3933635	down
PRAMEF5	NM_001013407	PRAME family member 5	-3.6232092	down	-2.5051455	down
ANO7	NM_001001891	anoctamin 7	-2.8737407	down	-3.3846624	down
			-3.64133	down	-5.701493	down
	BC085019		-3.6552308	down	-4.041795	down
LOC100130278	AK094114	uncharacterized LOC100130278	-2.7314095	down	-2.1353686	down
LOC728196	BC021736	uncharacterized LOC728196	-3.7549574	down	-3.0604086	down
CDH23	NM_052836	cadherin-related 23	-3.1131163	down	-3.5766797	down
TP53AIP1	NM_001195195	tumor protein p53 regulated apoptosis inducing protein 1	-6.3529067	down	-5.6300135	down
ZNF521	NM_015461	zinc finger protein 521	-5.4888496	down	-4.9204555	down
LOC100652869	XM_005254101	basic proline-rich protein-like	-3.5021257	down	-2.2566817	down
TMEM54	NM_033504	transmembrane protein 54	-3.1583796	down	-3.4771028	down
JAZF1-AS1	NR_034097	JAZF1 antisense RNA 1	-2.0210278	down	-2.2433896	down
			-3.9052703	down	-2.7800899	down

FBLL1	NR_024356	fibrillarin-like 1	-2.1588242	down	-2.3722076	down
	M27336		-5.0882497	down	-3.7871387	down
			-3.0497742	down	-2.549752	down
LINC00693		long intergenic non-protein coding RNA 693	-2.5811281	down	-2.0339217	down
CELF6	NM_001172684	CUGBP, Elav-like family member 6	-2.3446755	down	-2.6516817	down
LOC100131048	AK125976	uncharacterized LOC100131048	-2.395401	down	-2.0402286	down
FAM183B	NR_028347	acyloxyacyl hydrolase (neutrophil)	-2.4139957	down	-2.2025957	down
OR2G2	NM_001001915	olfactory receptor, family 2, subfamily G, member 2	-5.1925764	down	-2.0096073	down
POTEI	NM_001277406	POTE ankyrin domain family, member I	-3.1637805	down	-4.067189	down
			-2.0912874	down	-2.7782009	down
CCDC144A	NM_014695	coiled-coil domain containing 144A	-2.8149688	down	-2.0681257	down
GRID1	NM_017551	glutamate receptor, ionotropic, delta 1	-5.60282	down	-3.1634269	down
HNRNPA3	NM_194247	heterogeneous nuclear ribonucleoprotein A3	-3.1076338	down	-2.4045832	down
INSC	NM_001031853	inscuteable homolog (Drosophila)	-2.363814	down	-2.2724304	down
APOB	BC051278	apolipoprotein B	-2.3353279	down	-2.2254267	down
			-3.0941298	down	-5.587338	down
XLOC_014512			-5.463075	down	-3.195272	down
	XR_243752		-5.4829135	down	-5.6406894	down
C9orf84	NM_173521	chromosome 9 open reading frame 84	-5.6855555	down	-2.2580492	down
STARD9		StAR-related lipid transfer (START) domain containing 9	-3.0600054	down	-4.066877	down
GAMT	NM_138924	guanidinoacetate N-methyltransferase	-2.1603847	down	-3.1134667	down
XLOC_012299			-3.5615017	down	-3.5790865	down
PYCR1	NM_006907	pyrroline-5-carboxylate reductase 1	-2.239936	down	-2.8095202	down
	AY461701		-5.131562	down	-4.0095925	down
CLDN3	NM_001306	claudin 3	-2.3323839	down	-2.3020046	down
PDZRN3	BC014432	PDZ domain containing ring finger 3	-3.8516345	down	-2.4092207	down
FFAR1	NM_005303	free fatty acid receptor 1	-2.783463	down	-2.0572886	down
			-4.4301763	down	-3.677437	down
			-2.484814	down	-2.5446966	down
IL24	NM_001185156	interleukin 24	-7.163365	down	-14.694345	down
AQP12A	NM_198998	aquaporin 12A	-2.710312	down	-2.184617	down
FLJ41327	AK123321	FLJ41327 protein	-4.333203	down	-2.5390434	down
TTLL10-AS1	XR_132470	TTLL10 antisense RNA 1	-2.3095708	down	-2.3826046	down
PRAMEF13	NM_001024661	PRAME family member 13	-2.5607765	down	-3.3176877	down
			-3.0229836	down	-2.4075794	down
C21orf88	NR_026543	chromosome 21 open reading frame 88	-3.1558237	down	-3.1465907	down
ZNF502	NM_033210	zinc finger protein 502	-2.2319617	down	-2.3460853	down
	AF116680		-3.204686	down	-2.1041095	down
XLOC_014512			-4.199353	down	-2.1084313	down
ZNF7	NM_001282796	zinc finger protein 7	-2.7727046	down	-2.479293	down
KCNE1	NM_000219	potassium voltage-gated channel, Isk-related family, member 1	-2.320797	down	-2.353031	down
			-3.609129	down	-2.3282945	down
GAFA1	AF190748	FGF2-associated protein GAFA1	-4.1789336	down	-3.424499	down
CHSY3	NM_175856	chondroitin sulfate synthase 3	-2.85819	down	-2.0585327	down
LOC339874	NR_038976	uncharacterized LOC339874	-2.7612095	down	-3.359486	down
IGFL2	AK125754	IGF-like family member 2	-2.6383815	down	-2.1914747	down
			-2.5658398	down	-2.1889653	down
			-3.9712124	down	-2.1629236	down
MSLN	NM_005823	mesothelin	-2.5596788	down	-2.3855743	down
SYNGAP1	NM_006772	synaptic Ras GTPase activating protein 1	-2.423149	down	-2.4489183	down
DEFB109P1	NR_024044	defensin, beta 109, pseudogene 1	-3.2719843	down	-2.6331418	down
WDR96	NM_025145	WD repeat domain 96	-4.8922844	down	-3.6573246	down
USP49	NM_001286554	ubiquitin specific peptidase 49	-2.6954446	down	-2.0703204	down
FGD5	NM_152536	FYVE, RhoGEF and PH domain containing 5	-4.523561	down	-3.1576493	down
SGSM2	AK124883	small G protein signaling modulator 2	-3.5707347	down	-4.649883	down
FLT4	AB209637	fms-related tyrosine kinase 4	-2.0052538	down	-2.8877766	down
RNF216	AK128551	ring finger protein 216	-2.2064335	down	-3.6984475	down
VSX1	NM_001256272	visual system homeobox 1	-2.4522958	down	-3.3461142	down
NRG3	NM_001010848	neuregulin 3	-3.7313676	down	-2.1122186	down
STK16	NM_001008910	serine/threonine kinase 16	-4.1459317	down	-5.1239147	down
LINC00410		long intergenic non-protein coding RNA 410	-2.0326953	down	-3.2167509	down
TMCO5B		transmembrane and coiled-coil domains 5B, pseudogene	-2.8259728	down	-2.0207627	down

CD209	NM_021155	CD209 molecule	-3.5167131	down	-4.00072	down
NADK2	NM_001085411	NAD kinase 2, mitochondrial	-2.4109905	down	-4.36624	down
	AK309505		-3.0657148	down	-3.4554362	down
LY6G5C	NM_025262	lymphocyte antigen 6 complex, locus G5C	-3.8617296	down	-9.029468	down
PSMB11	NM_001099780	proteasome (prosome, macropain) subunit, beta type, 11	-5.6147547	down	-2.034291	down
LOC100240734	NR_026657	uncharacterized LOC100240734	-2.2160983	down	-2.6423094	down
TAF1L	NM_153809	TAF1 RNA polymerase II, TATA box binding protein (TBP)-associated factor, 210kDa-like	-5.080518	down	-5.5881977	down
	AF289570		-4.5587263	down	-4.904358	down
C1orf210	NM_182517	chromosome 1 open reading frame 210	-4.7883873	down	-3.2689347	down
KLB	NM_175737	klotho beta	-2.282705	down	-2.7674081	down
LINC00273	NR_038368	long intergenic non-protein coding RNA 273	-5.749421	down	-11.5935135	down
ANKRD18DP	NR_003291	ankyrin repeat domain 18D, pseudogene	-3.2685976	down	-2.1356416	down
TRIM3	NM_006458	tripartite motif containing 3	-2.0928247	down	-2.016826	down
XKR6	NM_173683	XK, Kell blood group complex subunit-related family, member 6	-3.098491	down	-2.8963418	down
KALRN	NM_003947	kalirin, RhoGEF kinase	-4.3364773	down	-4.946721	down
NEBL		nebulette	-3.5314546	down	-2.6540837	down
ERC2-IT1	NR_024615	ERC2 intronic transcript 1 (non-protein coding)	-4.1177244	down	-3.9506292	down
MYLPF	NM_013292	myosin light chain, phosphorylatable, fast skeletal muscle	-2.7917573	down	-2.8975496	down
MAGEA2B	NM_153488	melanoma antigen family A, 2B	-6.062839	down	-3.042379	down
FAM110D	NM_024869	family with sequence similarity 110, member D	-2.3871846	down	-2.0276973	down
SCGB2B3P	BC114619	secretoglobin, family 2B, member 3, pseudogene	-3.2321026	down	-2.5438118	down
DEFB130	NM_001037804	defensin, beta 130	-3.6249826	down	-2.711161	down
HSPG2	NM_005529	heparan sulfate proteoglycan 2	-3.78184	down	-3.7379262	down
TRPM3	NM_001007471	transient receptor potential cation channel, subfamily M, member 3	-2.3731909	down	-2.509547	down
ACKR2	NM_001296	atypical chemokine receptor 2	-5.3267617	down	-2.5091977	down
MTHFD2P1		methylenetetrahydrofolate dehydrogenase (NADP+ dependent) 2, methenyltetrahydrofolate cyclohydrolase pseudogene 1	-3.1037102	down	-2.1858025	down
	AK054980		-2.38821	down	-2.2069337	down
MAGEA4	NM_002362	melanoma antigen family A, 4	-4.6131377	down	-2.1924	down
MYOD1	NM_002478	myogenic differentiation 1	-4.8791566	down	-7.1367836	down
RBM10	AK098115	RNA binding motif protein 10	-3.2416704	down	-2.5255873	down
SLC28A1	NM_201651	solute carrier family 28 (concentrative nucleoside transporter), member 1	-4.0050144	down	-2.9468334	down
			-2.3254492	down	-4.1608076	down
ANKRD30BP2	NR_026916	ankyrin repeat domain 30B pseudogene 2	-2.039451	down	-2.7637517	down
KDM2B	AB031230	lysine (K)-specific demethylase 2B	-3.9652858	down	-2.3524652	down
			-4.221551	down	-2.8000383	down
			-3.4848704	down	-2.2860525	down
UBE2Q2P3	NR_024474	ubiquitin-conjugating enzyme E2Q family member 2 pseudogene 3	-4.638243	down	-2.3396132	down
	AY927536		-3.1960142	down	-2.0427396	down
DMRT3	NM_021240	doublesex and mab-3 related transcription factor 3	-6.351967	down	-3.0700583	down
			-4.0810723	down	-3.3692966	down
GALP	NM_033106	galanin-like peptide	-2.1908662	down	-2.3076105	down
FBXO44	NM_001014765	F-box protein 44	-2.466258	down	-3.7377772	down
SDK2	NM_001144952	sidekick cell adhesion molecule 2	-2.7065814	down	-2.5141318	down
			-2.8158572	down	-2.526869	down
			-3.9913695	down	-3.1192315	down
LRRC3C	NM_001195545	leucine rich repeat containing 3C	-2.8349178	down	-2.158264	down
C9orf117	BC133027	chromosome 9 open reading frame 117	-3.4086435	down	-3.2173495	down
PTGER4P2-CDK2AP2P2	NR_024496	PTGER4P2-CDK2AP2P2 readthrough transcribed pseudogene	-2.4928596	down	-3.0705323	down
KRTAP29-1	NM_001257309	keratin associated protein 29-1	-2.6373148	down	-2.3721492	down
			-4.324224	down	-2.6186666	down
			-2.6373143	down	-2.5506198	down
	CU677870		-3.519375	down	-2.3017318	down
UCA1	NR_015379	urothelial cancer associated 1 (non-protein coding)	-2.7921085	down	-2.8168876	down
			-4.126632	down	-4.000587	down
AKNA	NM_030767	AT-hook transcription factor	-3.446541	down	-5.454889	down
ATP4A	NM_000704	ATPase, H+/K+ exchanging, alpha polypeptide	-3.3158548	down	-2.286891	down
LOC100128402	AK124574	uncharacterized LOC100128402	-2.382179	down	-2.8967838	down

DEFB132	NM_207469	defensin, beta 132	-4.181409	down	-2.4618776	down
SNX31	NM_152628	sorting nexin 31	-4.377163	down	-2.1604326	down
LOC283501	AK093292	uncharacterized LOC283501	-5.249557	down	-3.187564	down
PTCHD2	NM_020780	patched domain containing 2	-2.156919	down	-2.1074114	down
XLOC_005165			-4.537724	down	-5.123204	down
PTPRF	BC048416	protein tyrosine phosphatase, receptor type, F	-2.859115	down	-6.87335	down
ZNF550	NM_001277092	zinc finger protein 550	-2.166701	down	-2.3173168	down
HYALP1		hyaluronoglucosaminidase pseudogene 1	-8.469493	down	-4.87453	down
PASD1	NM_173493	PAS domain containing 1	-3.7638307	down	-3.107486	down
	AK096239		-2.143118	down	-2.659365	down
	AK310283		-2.2995627	down	-2.2905316	down
	BC022023		-5.1547475	down	-3.3703258	down
			-2.6670568	down	-2.3039758	down
KCNQ2	NM_004518	potassium voltage-gated channel, KQT-like subfamily, member 2	-2.07185	down	-2.6802337	down
			-5.377821	down	-4.353743	down
OR56A4	NM_001005179	olfactory receptor, family 56, subfamily A, member 4	-2.6277683	down	-3.442417	down
COL9A1	NM_001851	collagen, type IX, alpha 1	-2.2062743	down	-2.5184052	down
MYCBPAP	NM_032133	MYCBP associated protein	-2.0877862	down	-2.5466025	down
KCNV2	NM_133497	potassium channel, subfamily V, member 2	-2.68557	down	-3.276973	down
PRAMEF3	NM_001013692	PRAME family member 3	-2.673795	down	-2.3842094	down
ARHGAP36	NM_144967	Rho GTPase activating protein 36	-3.3971918	down	-2.4338784	down
POU5F1	NM_002701	POU class 5 homeobox 1	-3.039562	down	-2.5848484	down
IPO7	NM_006391	importin 7	-2.3144677	down	-2.1834571	down
LOC100132071	AK128253	uncharacterized LOC100132071	-2.5642095	down	-2.3124185	down
FLJ37786	XR_108343	uncharacterized LOC642691	-4.030967	down	-4.214188	down
FLJ43903		uncharacterized LOC401471	-13.164824	down	-17.184885	down
			-4.0000906	down	-4.050142	down
OR2Y1	NM_001001657	olfactory receptor, family 2, subfamily Y, member 1	-2.5017567	down	-2.3281872	down
			-2.2851682	down	-4.3974977	down
SLC30A3	NM_003459	solute carrier family 30 (zinc transporter), member 3	-2.101825	down	-3.165658	down
BDNF-AS	NR_002832	BDNF antisense RNA	-3.6844656	down	-3.3081436	down
CDH18	NM_004934	cadherin 18, type 2	-3.7692652	down	-3.1158633	down
SGCD	NM_000337	sarcoglycan, delta (35kDa dystrophin-associated glycoprotein)	-5.0472403	down	-5.716335	down
ABCB11	NM_003742	ATP-binding cassette, sub-family B (MDR/TAP), member 11	-2.037655	down	-2.027744	down
FAM71F1	NM_032599	family with sequence similarity 71, member F1	-2.6675453	down	-2.8818603	down
PRDM15	NM_022115	PR domain containing 15	-2.1434133	down	-2.5705073	down
NUGGC	NM_001010906	nuclear GTPase, germinal center associated	-3.313941	down	-2.86587	down
CYP24A1	NM_000782	cytochrome P450, family 24, subfamily A, polypeptide 1	-6.168156	down	-2.2484956	down
MUM1	NM_032853	melanoma associated antigen (mutated) 1	-3.3921714	down	-2.1086583	down
HELZ2	NM_001037335	helicase with zinc finger 2, transcriptional coactivator	-2.6748044	down	-6.1510987	down
XLOC_011569			-3.9999313	down	-3.532509	down
			-6.1922946	down	-3.6665282	down
SYNDIG1L	NM_001105579	synapse differentiation inducing 1-like	-3.2151628	down	-4.2930007	down
VSX1	NM_014588	visual system homeobox 1	-2.0182288	down	-2.0050478	down
CECR2	AF411609	cat eye syndrome chromosome region, candidate 2	-6.1551948	down	-4.5544186	down
LOC286154	AK096739	uncharacterized LOC286154	-3.5007787	down	-2.5067275	down
	AK172763		-3.6620286	down	-3.33453	down
SRPX2	NM_014467	sushi-repeat containing protein, X-linked 2	-3.4255567	down	-2.1659663	down
TRIM43	NM_138800	tripartite motif containing 43	-2.4109194	down	-2.4103746	down
LOC147646	NM_001193623	uncharacterized LOC147646	-2.1772976	down	-4.1407604	down
			-3.50995	down	-4.5689235	down
CPNE7	NM_014427	copine VII	-2.1578808	down	-2.0932019	down
OR4F6	NM_001005326	olfactory receptor, family 4, subfamily F, member 6	-4.400692	down	-2.498925	down
XLOC_014512			-3.2084534	down	-2.6798487	down
NTRK3-AS1	NR_038229	NTRK3 antisense RNA 1	-6.0013185	down	-3.1906116	down
HIP1R	XM_005253629	huntingtin interacting protein 1 related	-3.8472257	down	-3.9575086	down
LOC283270	AK091052	uncharacterized LOC283270	-3.8437066	down	-6.2427974	down
LOC101930441	XM_005276062	mucin-3A-like	-2.2318156	down	-3.0007043	down
GRIK2	NM_001166247	glutamate receptor, ionotropic, kainate 2	-3.3479972	down	-3.7389605	down
ADAMDEC1	NM_001145271	ADAM-like, decysin 1	-4.589097	down	-2.8370197	down
			-2.898056	down	-4.2263117	down

RNF157	NM_052916	ring finger protein 157	-4.5362597	down	-5.696053	down
C10orf129	NM_207321	chromosome 10 open reading frame 129	-2.8480814	down	-3.9808633	down
			-2.021194	down	-2.2515256	down
POM121L1P		POM121 transmembrane nucleoporin-like 1, pseudogene	-3.749679	down	-2.0600715	down
			-2.0854013	down	-4.2592173	down
OR2G3	NM_001001914	olfactory receptor, family 2, subfamily G, member 3	-2.0783615	down	-2.050878	down
SYT8	NM_138567	synaptotagmin VIII	-3.7881665	down	-2.3531735	down
OR7E37P	NR_002163	olfactory receptor, family 7, subfamily E, member 37 pseudogene	-7.010088	down	-5.2708173	down
LOC100130741	AK127222	uncharacterized LOC100130741	-2.6621487	down	-3.072275	down
FAM64A	NM_001195228	family with sequence similarity 64, member A	-3.9991457	down	-4.513279	down
LOC100130285	XR_243328	uncharacterized LOC100130285	-2.588497	down	-3.3794758	down
SEMA6C	NM_001178061	sema domain, transmembrane domain (TM), and cytoplasmic domain, (semaphorin) 6C	-3.974376	down	-3.451388	down
GOLGA8EP	NR_033350	golgin A8 family, member E, pseudogene	-2.9001524	down	-2.189271	down
NALCN	AK094390	sodium leak channel, non-selective	-2.681519	down	-3.0156343	down
C12orf50	NM_152589	chromosome 12 open reading frame 50	-3.4775212	down	-3.3261697	down
			-2.0930085	down	-2.0394537	down
			-4.577794	down	-2.3084831	down
LOC284412	NR_029390	uncharacterized LOC284412	-2.5856466	down	-2.5562716	down
	DA613845		-2.2043633	down	-3.3073323	down
OR11H6	NM_001004480	olfactory receptor, family 11, subfamily H, member 6	-2.7578084	down	-2.2350492	down
TRIM51	NM_032681	tripartite motif-containing 51	-3.021771	down	-2.4483905	down
GLP2R	NM_004246	glucagon-like peptide 2 receptor	-3.5124466	down	-2.654922	down
AMMECR1	NM_001171689	Alport syndrome, mental retardation, midface hypoplasia and elliptocytosis chromosomal region gene 1	-2.861841	down	-3.9276004	down
KIAA0087	NR_022006	KIAA0087	-7.8302794	down	-7.228069	down
LOC100131581	AK092544	uncharacterized LOC100131581	-5.5654273	down	-2.8613105	down
KRTAP12-2	NM_181684	keratin associated protein 12-2	-2.8004386	down	-2.7637467	down
SLC34A2	NM_006424	solute carrier family 34 (type II sodium/phosphate contransporter), member 2	-2.90975	down	-2.3184993	down
			-4.152239	down	-2.6399198	down
			-2.1294622	down	-2.5514452	down
FLJ44790	XR_109635	uncharacterized FLJ44790	-3.3191893	down	-2.7173836	down
	AB529268		-2.4113262	down	-2.2521598	down
	AK090448		-2.6160192	down	-2.1621234	down
PTH1R	NM_000316	parathyroid hormone 1 receptor	-2.1189172	down	-2.3096282	down
			-2.3642833	down	-2.1962855	down
	BC028053		-2.4468186	down	-3.2160168	down
MYH14	NM_001077186	myosin, heavy chain 14, non-muscle	-4.300755	down	-3.9846447	down
ZNF621	NM_198484	zinc finger protein 621	-2.906025	down	-3.3787754	down
SNAR-C3	BF570763	small ILF3/NF90-associated RNA C3	-6.8305964	down	-5.299424	down
XLOC_014512			-5.5697865	down	-4.396531	down
WDR64	NM_144625	WD repeat domain 64	-5.2395377	down	-3.1141214	down
SSTR4	NM_001052	somatostatin receptor 4	-2.3040757	down	-2.2098005	down
CHRM2	NM_001006630	cholinergic receptor, muscarinic 2	-2.2591429	down	-2.6866453	down
WFIKKN2	NM_175575	WAP, follistatin/kazal, immunoglobulin, kunitz and netrin domain containing 2	-3.320794	down	-4.5092516	down
			-2.8171134	down	-2.4341285	down
C17orf104	BC035159	chromosome 17 open reading frame 104	-2.8501248	down	-2.8974319	down
SUSD2	NM_019601	sushi domain containing 2	-2.470507	down	-2.9208345	down
AKAP12	NM_144497	A kinase (PRKA) anchor protein 12	-3.1571686	down	-2.039172	down
			-2.6902053	down	-2.8659291	down
CXCL14	NM_004887	chemokine (C-X-C motif) ligand 14	-4.384196	down	-2.5581894	down
			-4.000027	down	-2.7704477	down
MEGF8	NM_001271938	multiple EGF-like-domains 8	-2.224648	down	-3.1479897	down
EXD1	NM_152596	exonuclease 3'-5' domain containing 1	-2.7454815	down	-4.3647523	down
			-2.4035234	down	-2.8587263	down
MAML3	NM_018717	mastermind-like 3 (Drosophila)	-6.9032307	down	-15.053548	down
LAMA4	NM_001105207	laminin, alpha 4	-2.6171181	down	-4.858686	down
FLJ13773	AK023835	uncharacterized LOC246318	-3.1289072	down	-2.103998	down
NLGN2	NM_020795	neuroligin 2	-2.3548706	down	-2.5072322	down
			-4.491011	down	-2.7703223	down
MUC6	AK092533	mucin 6, oligomeric mucus/gel-forming	-3.4253206	down	-2.2798593	down

STH	NM_001007532	saitohin	-6.309312	down	-4.316842	down
			-3.9920168	down	-2.496098	down
HAAO		3-hydroxyanthranilate 3,4-dioxygenase	-5.3837333	down	-5.9729013	down
	AF068294		-4.2279806	down	-4.3153763	down
GNAL	NM_182978	guanine nucleotide binding protein (G protein), alpha activating activity polypeptide, olfactory type	-3.1100879	down	-2.8628473	down
PPP6R2	AK024148	protein phosphatase 6, regulatory subunit 2	-4.283714	down	-5.953941	down
UGT2B7	NM_001074	UDP glucuronosyltransferase 2 family, polypeptide B7	-3.3240469	down	-4.2031784	down
DBX1	NM_001029865	developing brain homeobox 1	-3.3425362	down	-2.674685	down
BUB1B	NM_001211	BUB1 mitotic checkpoint serine/threonine kinase B	-3.9039443	down	-4.010965	down
DDB1		damage-specific DNA binding protein 1, 127kDa	-3.287064	down	-2.6218011	down
LZTS1	NM_021020	leucine zipper, putative tumor suppressor 1	-2.017927	down	-2.11839	down
			-4.0435286	down	-3.8491814	down
SPPL2C	NM_175882	signal peptide peptidase like 2C	-3.5815568	down	-3.3487146	down
CLEC6A	NM_001007033	C-type lectin domain family 6, member A	-4.035783	down	-3.0251372	down
PARPBP	NM_017915	PARP1 binding protein	-5.487618	down	-7.67942	down
TRIM61	NM_001012414	tripartite motif containing 61	-2.4182298	down	-2.1925528	down
XLOC_008300			-2.6192105	down	-2.2568557	down
PRM2	NM_001286359	protamine 2	-2.3511887	down	-2.4658096	down
			-2.8285642	down	-2.1807523	down
TWIST1	NM_000474	twist family bHLH transcription factor 1	-2.9219046	down	-2.3576634	down
CELA3A	NM_005747	chymotrypsin-like elastase family, member 3A	-3.4059	down	-2.2357085	down
TCEB3C	NM_145653	transcription elongation factor B polypeptide 3C (elongin A3)	-9.024723	down	-8.311952	down
NRG2	AK124504	neuregulin 2	-2.7005093	down	-2.7202995	down
C6orf201	NM_001085401	chromosome 6 open reading frame 201	-2.4032972	down	-2.1229281	down
CIDEA	NM_001279	cell death-inducing DFFA-like effector a	-2.5417988	down	-5.287442	down
CHD5	NM_015557	chromodomain helicase DNA binding protein 5	-4.255513	down	-2.7657988	down
KRTAP10-4	NM_198687	keratin associated protein 10-4	-2.3867939	down	-4.6243157	down
NPL	BC034966	N-acetylneuraminate pyruvate lyase (dihydrodipicolinate synthase)	-2.9103768	down	-2.38428	down
OVOL2	NM_021220	ovo-like zinc finger 2	-3.847198	down	-3.4602869	down
TMEM257	NM_004709	transmembrane protein 257	-4.6702285	down	-2.5168185	down
			-3.7606943	down	-3.6310387	down
DEFB136	NM_001033018	defensin, beta 136	-2.0803308	down	-2.5463066	down
MRP63	NM_024026	mitochondrial ribosomal protein 63	-4.125595	down	-2.5852232	down
TPT1-AS1	NR_024458	TPT1 antisense RNA 1	-2.1273007	down	-2.055494	down
NTF4	NM_006179	neurotrophin 4	-4.8414006	down	-2.2685297	down
FADS6	NM_178128	fatty acid desaturase 6	-3.189713	down	-5.106219	down
			-2.7428076	down	-2.7674096	down
	AK074630		-2.150738	down	-3.5783901	down
ZBTB12	NM_181842	zinc finger and BTB domain containing 12	-2.7510123	down	-2.2155778	down
CRYBB2P1		crystallin, beta B2 pseudogene 1	-3.8412104	down	-2.578034	down
AGMO	NM_001004320	alkylglycerol monooxygenase	-2.0152147	down	-2.9283755	down
BRSK1	NM_032430	BR serine/threonine kinase 1	-4.4639883	down	-3.3366559	down
	CU677925		-3.4826844	down	-2.1556482	down
NPW	NM_001099456	neuropeptide W	-3.3776999	down	-2.8928888	down
LMX1A	NM_177398	LIM homeobox transcription factor 1, alpha	-3.3338974	down	-2.7074177	down
GJA5	NM_005266	gap junction protein, alpha 5, 40kDa	-3.6569626	down	-2.4397182	down
			-2.6388934	down	-3.1679533	down
			-2.7665997	down	-2.2375312	down
OR4F4	NM_001004195	olfactory receptor, family 4, subfamily F, member 4	-2.8472764	down	-2.2719133	down
NUTM1	NM_175741	NUT midline carcinoma, family member 1	-4.030192	down	-3.0860043	down
LRP11	XM_005267168	low density lipoprotein receptor-related protein 11	-3.7708538	down	-2.8726544	down
			-2.2673814	down	-3.8446653	down
DNAAF2	NM_001083908	dynein, axonemal, assembly factor 2	-2.21681	down	-2.1570506	down
LOC100287225	NR_040075	uncharacterized LOC100287225	-4.758138	down	-2.439124	down
GVINP1	NR_003945	GTPase, very large interferon inducible pseudogene 1	-2.0340738	down	-2.1592197	down
HOMER1		homer homolog 1 (Drosophila)	-5.7913218	down	-3.095869	down
ROBO2	NM_002942	roundabout, axon guidance receptor, homolog 2 (Drosophila)	-4.7293706	down	-4.242307	down
GLI2	NM_005270	GLI family zinc finger 2	-2.6989348	down	-2.0886974	down
TGM7	NM_052955	transglutaminase 7	-2.994587	down	-2.6584141	down
ZNF749	NM_001023561	zinc finger protein 749	-3.815186	down	-2.2963426	down

FGF7		fibroblast growth factor 7	-5.7991605	down	-3.1798081	down
LOC100129518	NR_037166	uncharacterized LOC100129518	-4.2684283	down	-3.0518596	down
VWA5B2	NM_138345	von Willebrand factor A domain containing 5B2	-2.6401281	down	-2.3341541	down
MUC22	NM_001198815	mucin 22	-4.3170433	down	-2.1166081	down
NAG20	AF210649	NAG20	-9.043121	down	-10.061569	down
	AK129565		-4.1287208	down	-4.287017	down
MPP2	NM_001278381	membrane protein, palmitoylated 2 (MAGUK p55 subfamily member 2)	-2.1509416	down	-2.9756818	down
SOX10	NM_006941	SRY (sex determining region Y)-box 10	-2.0350943	down	-2.019094	down
LACTBL1	XM_003846622	lactamase, beta-like 1	-2.6398363	down	-2.4899201	down
FLJ46361		deleted in malignant brain tumors 1 pseudogene	-3.599743	down	-2.379761	down
LOC650293	NM_001040071	seven transmembrane helix receptor	-5.6999173	down	-3.1572661	down
ACSS3	NM_024560	acyl-CoA synthetase short-chain family member 3	-2.5697956	down	-2.8812094	down
	AF384996		-3.3046668	down	-3.1008523	down
P4HA2	NM_001017973	prolyl 4-hydroxylase, alpha polypeptide II	-2.3412817	down	-2.0589087	down
XLOC_014512			-2.1736803	down	-3.4896889	down
RHBDL2	NM_017821	rhomboid, veinlet-like 2 (Drosophila)	-3.181914	down	-2.0552807	down
BCL2L10	NM_020396	BCL2-like 10 (apoptosis facilitator)	-8.181561	down	-5.0572643	down
LINC00235	NR_024121	long intergenic non-protein coding RNA 235	-4.40941	down	-2.086108	down
LOC100128498	AK126742	uncharacterized LOC100128498	-2.9709055	down	-2.821145	down
	XM_001714385		-2.2547572	down	-3.4506288	down
RBM33	NM_053043	RNA binding motif protein 33	-2.0423963	down	-2.5258825	down
			-3.2855985	down	-3.0081782	down
CDX1	NM_001804	caudal type homeobox 1	-3.1783936	down	-2.900254	down
			-2.5416565	down	-3.316415	down
TXLNB		taxilin beta	-6.5075784	down	-2.9084172	down
	BC004968		-11.480335	down	-14.663246	down
CATSPER2		cation channel, sperm associated 2	-3.9929798	down	-5.412927	down
FAM9B	NM_205849	family with sequence similarity 9, member B	-2.8020952	down	-2.0680704	down
PHKG1	NM_001258459	phosphorylase kinase, gamma 1 (muscle)	-3.249507	down	-3.820593	down
VSX1	NM_199425	visual system homeobox 1	-2.022524	down	-2.3420432	down
	BC015443		-2.2768896	down	-3.1130645	down
SYPL2	NM_001040709	synaptophysin-like 2	-5.377401	down	-5.0311785	down
PTPRC	NM_002838	protein tyrosine phosphatase, receptor type, C	-2.037058	down	-2.4676294	down
			-2.864905	down	-3.6696165	down
FLJ13224	NR_026806	uncharacterized LOC79857	-2.764445	down	-3.3484845	down
GRIP1	NM_021150	glutamate receptor interacting protein 1	-3.5788162	down	-3.6491559	down
FGF3	NM_005247	fibroblast growth factor 3	-11.292002	down	-13.7881565	down
BCL2	NM_000657	B-cell CLL/lymphoma 2	-2.2831893	down	-2.037091	down
ADCY1	NM_021116	adenylate cyclase 1 (brain)	-4.2855544	down	-3.6959312	down
MRPS16	NM_016065	mitochondrial ribosomal protein S16	-3.8599627	down	-2.248775	down
			-3.9068456	down	-2.9753563	down
CPN2	NM_001080513	carboxypeptidase N, polypeptide 2	-2.4232342	down	-3.211302	down
			-3.1515028	down	-3.5383708	down
HOXA7	NM_006896	homeobox A7	-4.500766	down	-3.1903841	down
SUPT20HL1	NM_001136234	suppressor of Ty 20 homolog (S. cerevisiae)-like 1	-3.2862632	down	-4.0409393	down
			-5.885245	down	-4.13544	down
			-2.0515213	down	-2.275369	down
EML2	NM_012155	echinoderm microtubule associated protein like 2	-2.2590485	down	-2.432665	down
LOC100272216		uncharacterized LOC100272216	-3.3476305	down	-3.7766654	down
			-2.5488825	down	-2.5882592	down
PPAPDC1A	NM_001030059	phosphatidic acid phosphatase type 2 domain containing 1A	-3.092788	down	-2.0184798	down
MYL10	NM_138403	myosin, light chain 10, regulatory	-4.2693377	down	-2.9560215	down
DPM3	NM_018973	dolichyl-phosphate mannosyltransferase polypeptide 3	-2.650582	down	-2.4301288	down
SYTL5	NM_138780	synaptotagmin-like 5	-2.0972455	down	-2.0588367	down
AWAT2	NM_001002254	acyl-CoA wax alcohol acyltransferase 2	-2.1516817	down	-2.237796	down
			-2.5261834	down	-6.600277	down
ANP32A-IT1	NR_026808	ANP32A intronic transcript 1 (non-protein coding)	-4.6453705	down	-2.7897952	down
DST	NM_001723	dystonin	-2.146557	down	-3.4785645	down
CFHR4	NM_006684	complement factor H-related 4	-4.3196845	down	-2.4945016	down
NYAP2	NM_020864	neuronal tyrosine-phosphorylated phosphoinositide-3-kinase adaptor 2	-2.5273674	down	-2.1306896	down

SLC13A3	NM_001193339	solute carrier family 13 (sodium-dependent dicarboxylate transporter), member 3	-3.0832705	down	-2.5012276	down
CNTN4	NM_175613	contactin 4	-3.6705587	down	-2.3370209	down
LINC00839	NR_026827	long intergenic non-protein coding RNA 839	-3.2451005	down	-2.203354	down
			-4.947109	down	-2.8130136	down
RFPL3S	NR_001450	RFPL3 antisense	-2.3393514	down	-2.4599001	down
PYY	NM_004160	peptide YY	-2.381993	down	-2.8745625	down
XLOC_014512			-7.3024993	down	-4.6487794	down
CCDC64B	NM_001103175	coiled-coil domain containing 64B	-7.086797	down	-8.239313	down
PDZD7	NM_001195263	PDZ domain containing 7	-3.483815	down	-2.757152	down
SORCS3	NM_014978	sortilin-related VPS10 domain containing receptor 3	-7.4999743	down	-4.1854196	down
LOC100129027	NR_038876	uncharacterized LOC100129027	-2.9324067	down	-2.3324537	down
CDC27	NM_001114091	cell division cycle 27	-3.3529873	down	-3.5207305	down
PSMD10		proteasome (prosome, macropain) 26S subunit, non-ATPase, 10	-11.659073	down	-4.215822	down
ZNF160	XM_005259381	zinc finger protein 160	-4.386037	down	-2.535495	down
KCNK13	NM_022054	potassium channel, subfamily K, member 13	-3.3013327	down	-4.140283	down
STAM	NM_003473	signal transducing adaptor molecule (SH3 domain and ITAM motif) 1	-2.2100203	down	-3.2446482	down
MPZ	NM_000530	myelin protein zero	-3.2613645	down	-2.3164196	down
ARMCX4		armadillo repeat containing, X-linked 4	-4.0474944	down	-4.506873	down
IL1RAPL1	NM_014271	interleukin 1 receptor accessory protein-like 1	-2.798176	down	-4.7195106	down
AK9	AK124171	adenylate kinase 9	-2.8478622	down	-2.4501972	down
PCNXL4	AK095489	pecanex-like 4 (Drosophila)	-8.512695	down	-3.2413363	down
FLNC	NM_001458	filamin C, gamma	-4.6592126	down	-2.333547	down
GRK1	NM_002929	G protein-coupled receptor kinase 1	-3.3471928	down	-2.9543786	down
C17orf72	NM_001164257	chromosome 17 open reading frame 72	-3.8624935	down	-4.932738	down
LOC286238	NM_001100111	uncharacterized LOC286238	-7.003478	down	-3.359222	down
CFL1P1		cofilin 1 (non-muscle) pseudogene 1	-5.7603097	down	-4.1626306	down
	BC131768		-6.988259	down	-14.568469	down
ST8SIA3	NM_015879	ST8 alpha-N-acetyl-neuraminide alpha-2,8-sialyltransferase 3	-2.3533812	down	-2.969064	down
C7orf13	NR_026865	chromosome 7 open reading frame 13	-4.130074	down	-3.7078924	down
SUSD2	NM_019601	sushi domain containing 2	-4.8122654	down	-3.070956	down
			-2.4343324	down	-4.772648	down
XLOC_014512			-4.8459706	down	-6.020332	down
SNAI2	NM_003068	snail family zinc finger 2	-3.4664352	down	-3.1396892	down
TSSK3	NM_052841	testis-specific serine kinase 3	-2.5004082	down	-2.4604442	down
KCNQ1DN		KCNQ1 downstream neighbor (non-protein coding)	-2.4163675	down	-2.7120717	down
DAAM2	BC078153	dishevelled associated activator of morphogenesis 2	-2.64459	down	-3.612623	down
SLC51B	NM_178859	solute carrier family 51, beta subunit	-4.014045	down	-3.0954852	down
UPK3A	NM_006953	uroplakin 3A	-3.8951068	down	-4.2959294	down
HNRNPU-AS1	NR_026778	HNRNPU antisense RNA 1	-2.090377	down	-2.758113	down
			-4.898429	down	-4.649454	down
KRTAP5-3	NM_001012708	keratin associated protein 5-3	-4.750036	down	-3.4099417	down
			-4.8345633	down	-3.400303	down
C2orf61	NM_173649	chromosome 2 open reading frame 61	-2.4952595	down	-4.651796	down
KCNT1	AK123276	potassium channel, subfamily T, member 1	-5.199707	down	-3.4384925	down
	XR_245740		-3.2351213	down	-3.3209505	down
CEACAM5	NM_004363	carcinoembryonic antigen-related cell adhesion molecule 5	-3.2936344	down	-2.1425428	down
			-3.8236752	down	-3.2922227	down
			-2.5795133	down	-2.3078423	down
TEX41		testis expressed 41 (non-protein coding)	-2.1533556	down	-2.6219852	down
CD164L2		CD164 sialomucin-like 2	-3.2204216	down	-2.5628273	down
			-4.3016853	down	-4.1499066	down
LOC100130193	AK096102	uncharacterized LOC100130193	-2.3975291	down	-4.886785	down
ADAMTSL1	NM_001040272	ADAMTS-like 1	-2.007045	down	-2.7540135	down
SLC35F1	NM_001029858	solute carrier family 35, member F1	-2.1552486	down	-2.722175	down
MMP24	NM_006690	matrix metallopeptidase 24 (membrane-inserted)	-3.4404671	down	-2.19764	down
NFIB	NM_005596	nuclear factor I/B	-3.01854	down	-2.010619	down
BTBD9	NM_052893	BTB (POZ) domain containing 9	-5.525792	down	-3.1878254	down
	AY203961		-4.81801	down	-2.5608068	down
C1orf86	AK054818	chromosome 1 open reading frame 86	-2.7995405	down	-4.3577604	down
			-4.967905	down	-4.784889	down

MGC34800	XR_110135	uncharacterized protein MGC34800	-2.4075873	down	-2.3042676	down
ZNF385C	AK128007	zinc finger protein 385C	-2.3752508	down	-4.192814	down
			-3.560824	down	-2.6761942	down
USP2	NM_004205	ubiquitin specific peptidase 2	-4.0459924	down	-2.1656077	down
	XR_244450		-4.0777574	down	-2.3239396	down
TUBG2	NM_016437	tubulin, gamma 2	-2.3899364	down	-3.9754875	down
XLOC_014512			-3.7147028	down	-2.9490306	down
MEP1A	NM_005588	meprin A, alpha (PABA peptide hydrolase)	-2.7293026	down	-4.245633	down
SDC3	NM_014654	syndecan 3	-2.6710043	down	-2.348735	down
PTPRK	NM_002844	protein tyrosine phosphatase, receptor type, K	-2.1162932	down	-2.455539	down
TRAPPC3L	AK002042	trafficking protein particle complex 3-like	-3.0332775	down	-2.6196525	down
FCRL5	NM_001195388	Fc receptor-like 5	-2.545056	down	-5.0250344	down
	AK026419		-5.6002483	down	-2.4700818	down
LOC100131195	AK097743	uncharacterized LOC100131195	-2.0920508	down	-4.797982	down
			-2.7286146	down	-3.0460346	down
LEPROTL1	NM_001128208	leptin receptor overlapping transcript-like 1	-2.7332027	down	-4.4890656	down
RAB7A	AF119891	RAB7A, member RAS oncogene family	-3.1113956	down	-2.522849	down
			-5.0724645	down	-5.2356024	down
			-3.3808103	down	-2.2561421	down
			-2.9529443	down	-4.5816	down
DOK7	XM_005247956	docking protein 7	-2.4975963	down	-4.1738076	down
BIRC7	NM_022161	baculoviral IAP repeat containing 7	-2.1600926	down	-3.8651798	down
CHRNA2	NM_000742	cholinergic receptor, nicotinic, alpha 2 (neuronal)	-2.3879519	down	-4.3793488	down
IGFALS	NM_004970	insulin-like growth factor binding protein, acid labile subunit	-3.5575476	down	-3.3129983	down
			-2.8758028	down	-3.6051116	down
LOC388882	XR_109751	uncharacterized LOC388882	-2.563767	down	-3.3496366	down
HPX-2	X74861	homeobox HPX-2	-2.4647527	down	-3.8764646	down
			-2.8099236	down	-4.3805804	down
GPR52	NM_005684	G protein-coupled receptor 52	-3.045984	down	-2.719857	down
LOC401176	BC043001	uncharacterized LOC401176	-2.0356565	down	-2.3629441	down
	XR_132593		-5.3166018	down	-2.2071903	down
FAM215A	NR_026770	family with sequence similarity 215, member A (non-protein coding)	-2.2663	down	-2.3708618	down
TNRC6C	NM_001142640	trinucleotide repeat containing 6C	-2.366664	down	-3.187599	down
CDRT1	NM_006382	CMT1A duplicated region transcript 1	-3.6224349	down	-2.9550984	down
			-2.2238503	down	-2.8204217	down
PRDM15	NM_022115	PR domain containing 15	-3.539146	down	-2.4915326	down
LOC728586	XR_159059	hCG1981531	-4.7047973	down	-2.68227	down
DDX31	NM_138620	DEAD (Asp-Glu-Ala-Asp) box polypeptide 31	-2.4094048	down	-2.0697954	down
			-4.824043	down	-3.0591376	down
NANOS2	NM_001029861	nanos homolog 2 (Drosophila)	-2.2167985	down	-3.2480094	down
LOC100130417		uncharacterized LOC100130417	-2.2563155	down	-2.8546896	down
TRIM48	NM_024114	tripartite motif containing 48	-4.441204	down	-3.0246205	down
SERHL2	NM_014509	serine hydrolase-like 2	-3.8752592	down	-2.3131166	down
	CU692621		-2.2269025	down	-2.4917176	down
HMGB3	NM_005342	high mobility group box 3	-2.2475595	down	-2.2783196	down
	AK127494		-4.1838593	down	-3.993354	down
FBXL22	NM_203373	F-box and leucine-rich repeat protein 22	-2.4989083	down	-3.2706625	down
			-4.155539	down	-2.1899219	down
PHGR1	NM_001145643	proline/histidine/glycine-rich 1	-2.2858489	down	-2.3261058	down
LOC729159	NM_001282301	UPF0607 protein ENSP00000381418-like	-4.5937133	down	-4.1811337	down
LOC100134868	NR_004846	uncharacterized LOC100134868	-3.5268638	down	-2.9018774	down
ADARB2	AK289895	adenosine deaminase, RNA-specific, B2 (non-functional)	-2.7499673	down	-2.1572196	down
SLC22A13	NM_004256	solute carrier family 22 (organic anion/urate transporter), member 13	-4.99975	down	-8.378844	down
KLHL14	XM_005258322	kelch-like family member 14	-4.626692	down	-2.8605113	down
MAP4	NM_030885	microtubule-associated protein 4	-2.4157965	down	-3.695181	down
SNORA26	AI792523	small nucleolar RNA, H/ACA box 26	-2.7077954	down	-3.5881493	down
DNASE1	NM_005223	deoxyribonuclease I	-2.2552814	down	-2.3602126	down
GATA4	NM_002052	GATA binding protein 4	-3.0824058	down	-2.032713	down
FRG2C	NM_001124759	FSHD region gene 2 family, member C	-3.4860063	down	-4.7455025	down
ASIC2		acid-sensing (proton-gated) ion channel 2	-6.6445665	down	-3.7661602	down
XLOC_006444			-2.554289	down	-2.5732157	down

SNORA80	DB304787	small nucleolar RNA, H/ACA box 80	-3.5858228	down	-4.2207484	down
ZNF720	XM_005255752	zinc finger protein 720	-3.6915834	down	-2.0350087	down
XLOC_014512			-3.233398	down	-3.0234857	down
PDLIM4	NM_003687	PDZ and LIM domain 4	-3.2684553	down	-3.443663	down
GLTPD1	NM_001029885	glycolipid transfer protein domain containing 1	-3.2156906	down	-4.349214	down
			-2.7814844	down	-3.0453532	down
GALNTL6	NM_001034845	UDP-N-acetyl-alpha-D-galactosamine:polypeptide N-acetylgalactosaminyltransferase-like 6	-3.1167448	down	-2.4416559	down
LOC100128946	NR_038944	uncharacterized LOC100128946	-2.2885587	down	-2.120539	down
C10orf91	NM_173541	chromosome 10 open reading frame 91	-2.6343257	down	-2.202203	down
			-6.7362247	down	-2.1661677	down
SH3GL3	AF036272	SH3-domain GRB2-like 3	-9.720975	down	-3.3240488	down
GABRE	NM_004961	gamma-aminobutyric acid (GABA) A receptor, epsilon	-5.865793	down	-4.76355	down
BRD7P3	NR_002730	bromodomain containing 7 pseudogene 3	-3.7400136	down	-3.635799	down
	XR_112948		-4.5553465	down	-2.3863375	down
			-5.201958	down	-2.5900695	down
FZD4	NM_012193	frizzled family receptor 4	-2.4081843	down	-2.6502554	down
OLFML3	NM_020190	olfactomedin-like 3	-3.3830307	down	-2.7830617	down
SH3BP5L	AK125138	SH3-binding domain protein 5-like	-3.603822	down	-4.087898	down
TECRL	NM_001010874	trans-2,3-enoyl-CoA reductase-like	-5.822932	down	-3.893835	down
MAGIX	NM_024859	MAGI family member, X-linked	-3.5742652	down	-2.5316963	down
LOC541473	NR_003602	FK506 binding protein 6, 36kDa pseudogene	-3.3182943	down	-2.1551108	down
SLC2A6	NM_017585	solute carrier family 2 (facilitated glucose transporter), member 6	-2.7849984	down	-2.6380157	down
SLC18A1	NM_001142325	solute carrier family 18 (vesicular monoamine transporter), member 1	-2.673114	down	-3.7324808	down
DNAJB3	NM_001001394	DnaJ (Hsp40) homolog, subfamily B, member 3	-6.209302	down	-4.1020045	down
PTGER1	NM_000955	prostaglandin E receptor 1 (subtype EP1), 42kDa	-5.4906716	down	-5.4921627	down
CAPN5	NM_004055	calpain 5	-2.9290805	down	-2.5296283	down
			-2.4156694	down	-2.2140472	down
			-2.5095646	down	-3.1748285	down
			-3.7195191	down	-2.0138168	down
			-4.727455	down	-4.1788774	down
ACTL7B	NM_006686	actin-like 7B	-3.5372226	down	-2.1480823	down
LOC441493	XR_246724	uncharacterized LOC441493	-4.1309505	down	-2.3391201	down
KRTAP4-5	NM_033188	keratin associated protein 4-5	-3.2040782	down	-2.0711207	down
CHIC1	NM_001039840	cysteine-rich hydrophobic domain 1	-2.3631287	down	-2.7092931	down
DNAJC14	NM_032364	DnaJ (Hsp40) homolog, subfamily C, member 14	-2.2589595	down	-2.3243752	down
	BC035844		-4.4034514	down	-5.0251207	down
PTGER4P2-CDK2AP2P2	NR_024496	PTGER4P2-CDK2AP2P2 readthrough transcribed pseudogene	-3.3345542	down	-3.8294752	down
SCN4B	NM_174934	sodium channel, voltage-gated, type IV, beta subunit	-2.3026211	down	-4.41341	down
HECW1-IT1	AK093262	HECW1 intronic transcript 1 (non-protein coding)	-4.673794	down	-2.8677733	down
ANKRD20A2	NM_001012421	ankyrin repeat domain 20 family, member A2	-4.2440553	down	-3.1776767	down
DPYSL3	NM_001387	dihydropyrimidinase-like 3	-5.1374197	down	-7.8794856	down
LINC00893		long intergenic non-protein coding RNA 893	-3.073363	down	-2.4553752	down
	AK091705		-3.2365527	down	-2.938854	down
NPAS3	NM_022123	neuronal PAS domain protein 3	-2.8375876	down	-2.5736332	down
RAP1GAP		RAP1 GTPase activating protein	-3.0960302	down	-3.8229792	down
FOXD4L2	NM_001099279	forkhead box D4-like 2	-3.7543197	down	-4.9400506	down
ROR1	NM_001083592	receptor tyrosine kinase-like orphan receptor 1	-2.3358982	down	-3.349082	down
JPH2	NM_020433	junctophilin 2	-2.3735423	down	-2.6826003	down
LOC100134368	NR_024453	uncharacterized LOC100134368	-5.7505503	down	-3.5742295	down
SERP1	AK125413	stress-associated endoplasmic reticulum protein 1	-2.510614	down	-2.0096798	down
ZNF667	NM_022103	zinc finger protein 667	-3.8506627	down	-3.8127968	down
			-2.5004954	down	-2.629157	down
			-5.3656635	down	-3.5300465	down
ASCL1	NM_004316	achaete-scute family bHLH transcription factor 1	-2.1384103	down	-2.3816438	down
KIAA1984	NM_001039374	KIAA1984	-3.7728004	down	-4.78533	down
UROC1	NM_144639	urocanate hydratase 1	-2.3174648	down	-2.5256565	down
DUOX2	NM_014080	dual oxidase 2	-2.4195597	down	-2.9561653	down
CDK13	NM_031267	cyclin-dependent kinase 13	-2.9897325	down	-2.6361322	down
KY	NM_178554	kyphoscoliosis peptidase	-3.153499	down	-2.494125	down
MMD2	NM_198403	monocyte to macrophage differentiation-associated 2	-3.5563962	down	-2.3638217	down

RNASE7	NM_032572	ribonuclease, RNase A family, 7	-3.441989	down	-3.0744746	down
FAM83G	NM_001039999	family with sequence similarity 83, member G	-3.7324202	down	-3.3051016	down
NR4A3	NM_173200	nuclear receptor subfamily 4, group A, member 3	-4.1451697	down	-2.4256346	down
SPACA4	NM_133498	sperm acrosome associated 4	-2.8797195	down	-2.8658245	down
FLJ16171	NR_046113	FLJ16171 protein	-4.456786	down	-3.077039	down
WDFY4	NM_020945	WDFY family member 4	-4.1682367	down	-6.073584	down
NRXN1	NM_004801	neurexin 1	-5.6087255	down	-6.327814	down
MAS1L	NM_052967	MAS1 oncogene-like	-2.1188357	down	-2.6141255	down
			-3.4498158	down	-2.8989537	down
C1orf94	NM_032884	chromosome 1 open reading frame 94	-4.2618175	down	-3.756665	down
EVC	NM_153717	Ellis van Creveld syndrome	-2.0444205	down	-2.7697384	down
PLA2G2F	NM_022819	phospholipase A2, group IIF	-4.5901465	down	-3.403952	down
MAST4	NM_198828	microtubule associated serine/threonine kinase family member 4	-2.270318	down	-3.4127192	down
MGC10814	BC004943	uncharacterized protein MGC10814	-2.8463356	down	-2.439593	down
CHKA	NM_001277	choline kinase alpha	-2.8922307	down	-3.3115492	down
			-2.3634236	down	-3.0070133	down
LOC729770	XR_112442	uncharacterized LOC729770	-4.0857825	down	-6.183721	down
			-4.771054	down	-2.9896858	down
	AK130290		-2.223409	down	-3.0822678	down
GDNF	NM_000514	glial cell derived neurotrophic factor	-3.3872845	down	-2.1684034	down
DYNC1I1	NM_004411	dynein, cytoplasmic 1, intermediate chain 1	-4.5076895	down	-3.1733766	down
POLQ	NM_199420	polymerase (DNA directed), theta	-3.473344	down	-3.7286987	down
ZDHHC14	NM_153746	zinc finger, DHHC-type containing 14	-2.2533472	down	-2.8085394	down
HSD3B1	NM_000862	hydroxy-delta-5-steroid dehydrogenase, 3 beta- and steroid delta-isomerase 1	-4.3433223	down	-2.3151848	down
TSPAN15	NM_012339	tetraspanin 15	-2.7452376	down	-3.7497544	down
GOLGA6L5P		golgin A6 family-like 5, pseudogene	-6.9764524	down	-3.574765	down
LOC254099	NR_038869	uncharacterized LOC254099	-2.1583831	down	-2.1128137	down
			-4.833741	down	-3.0458848	down
LOC440570	BC036435	uncharacterized LOC440570	-3.3676214	down	-2.5759373	down
PTK2B	AK128371	protein tyrosine kinase 2 beta	-3.2689044	down	-5.003921	down
			-3.6835198	down	-2.0228636	down
TMEM213	NM_001085429	transmembrane protein 213	-3.141155	down	-2.7899535	down
ATP6V0A4	NM_020632	ATPase, H+ transporting, lysosomal V0 subunit a4	-3.362149	down	-2.221564	down
ZNF236	AF085243	zinc finger protein 236	-3.3924828	down	-3.1913104	down
LOC100128770	NR_047572	uncharacterized LOC100128770	-4.8236938	down	-2.5847692	down
PAQR3	BC031256	progestin and adipoQ receptor family member III	-3.0193427	down	-4.154528	down
AP3B2	NM_004644	adaptor-related protein complex 3, beta 2 subunit	-2.860026	down	-3.4030423	down
PLA2G4F	NM_213600	phospholipase A2, group IVF	-3.1767044	down	-2.6701288	down
PAX3	NM_013942	paired box 3	-2.1487	down	-3.6595964	down
LOC100133306	AK125136	uncharacterized LOC100133306	-3.275926	down	-2.4206388	down
LRRTM2	NM_015564	leucine rich repeat transmembrane neuronal 2	-4.2407784	down	-2.1928859	down
GLOD5	NM_001080489	glyoxalase domain containing 5	-2.820592	down	-3.307993	down
ENTPD3	NM_001248	ectonucleoside triphosphate diphosphohydrolase 3	-2.487411	down	-3.190444	down
HOXD10	NM_002148	homeobox D10	-2.5739188	down	-2.7860587	down
GPR151	NM_194251	G protein-coupled receptor 151	-2.025946	down	-2.0616794	down
KRTAP7-1	NM_181606	keratin associated protein 7-1 (gene/pseudogene)	-4.4194274	down	-2.0595503	down
FHL2	CR936682	four and a half LIM domains 2	-4.855638	down	-3.5324337	down
PEG3-AS1	NR_023847	PEG3 antisense RNA 1	-5.20535	down	-2.824169	down
C7orf60	NM_152556	chromosome 7 open reading frame 60	-2.0350688	down	-2.3955445	down
KRTAP9-4	NM_033191	keratin associated protein 9-4	-3.8046248	down	-2.282601	down
MS4A2	NM_000139	membrane-spanning 4-domains, subfamily A, member 2	-2.1466374	down	-2.3528442	down
OTOG	XM_005252894	otogelin	-3.856835	down	-2.0336635	down
MOGAT1	NM_058165	monoacylglycerol O-acyltransferase 1	-3.0788498	down	-2.0775344	down
DCDC5	NM_020869	doublecortin domain containing 5	-5.717283	down	-2.1005309	down
SGSM1	NM_001039948	small G protein signaling modulator 1	-4.089707	down	-4.0712523	down
H1FOO	NM_153833	H1 histone family, member O, oocyte-specific	-2.4701095	down	-2.195511	down
HHIPL1	NM_001127258	HHIP-like 1	-4.3807864	down	-2.9411142	down
PRSS27	NM_031948	protease, serine 27	-2.9303668	down	-2.892147	down
ADAD2	NM_139174	adenosine deaminase domain containing 2	-3.2303932	down	-2.6538084	down
EGFLAM	NM_152403	EGF-like, fibronectin type III and laminin G domains	-2.069753	down	-2.8712275	down
IL21	NM_021803	interleukin 21	-3.9880693	down	-2.505085	down
			-6.566463	down	-3.3438714	down

C12orf77	NM_001101339	chromosome 12 open reading frame 77	-3.431409	down	-2.3369946	down
PRPF40B	NM_001031698	PRP40 pre-mRNA processing factor 40 homolog B (S. cerevisiae)	-2.0940976	down	-2.1412349	down
KRTAP13-3	NM_181622	keratin associated protein 13-3	-3.7251003	down	-2.4094756	down
SHANK2	NM_012309	SH3 and multiple ankyrin repeat domains 2	-2.6133018	down	-3.990878	down
LOC100129840	AK126633	uncharacterized LOC100129840	-2.0743992	down	-2.2003484	down
SAMM50	AK124895	SAMM50 sorting and assembly machinery component	-2.8685935	down	-2.794607	down
			-3.4017203	down	-4.5553555	down
GRIK1-AS1	NR_027021	GRIK1 antisense RNA 1	-4.528228	down	-4.8278446	down
IGF1	NM_000618	insulin-like growth factor 1 (somatomedin C)	-4.2389855	down	-2.6714737	down
SMPDL3B	NM_001009568	sphingomyelin phosphodiesterase, acid-like 3B	-2.100032	down	-2.5115447	down
DEFB115	NM_001037730	defensin, beta 115	-4.099375	down	-2.3854241	down
MIR503HG	NR_024607	MIR503 host gene (non-protein coding)	-4.262186	down	-2.8313148	down
			-2.6794598	down	-3.501977	down
TAS2R13	NM_023920	taste receptor, type 2, member 13	-2.197716	down	-6.0611205	down
C9orf92	NM_001271829	chromosome 9 open reading frame 92	-2.1804469	down	-2.2791998	down
C1QTNF1	NM_198594	C1q and tumor necrosis factor related protein 1	-3.4386747	down	-3.01527	down
RNF212	NM_001131034	ring finger protein 212	-3.345049	down	-2.3526158	down
SOGA1	NM_199181	suppressor of glucose, autophagy associated 1	-2.5017445	down	-3.131229	down
FAM134A	NM_024293	family with sequence similarity 134, member A	-2.3377404	down	-2.2635784	down
			-2.5455604	down	-3.0650012	down
SOX21	NM_007084	SRY (sex determining region Y)-box 21	-4.509207	down	-3.1146472	down
OR4F29	NM_001005221	olfactory receptor, family 4, subfamily F, member 29	-5.0110016	down	-2.3857095	down
TREH	NM_007180	trehalase (brush-border membrane glycoprotein)	-2.1237082	down	-3.3082974	down
LOC440040	NR_027044	glutamate receptor, metabotropic 5 pseudogene	-4.0887885	down	-2.1251934	down
DMBX1	NM_147192	diencephalon/mesencephalon homeobox 1	-2.1984198	down	-2.8457117	down
C20orf166-AS1	AK054875	C20orf166 antisense RNA 1	-6.636039	down	-3.8827298	down
CASR	NM_001178065	calcium-sensing receptor	-3.831848	down	-2.2855563	down
EGFR	NM_201282	epidermal growth factor receptor	-2.252608	down	-3.1577954	down
LOC653712	NR_034179	intraflagellar transport 122 homolog (Chlamydomonas) pseudogene	-3.9754863	down	-4.171725	down
AGBL1	NM_152336	ATP/GTP binding protein-like 1	-2.53444	down	-2.045469	down
	XR_244723		-5.293987	down	-2.8013902	down
DEFB123	NM_153324	defensin, beta 123	-2.2521536	down	-2.784746	down
FN1	NM_054034	fibronectin 1	-4.186497	down	-2.2919369	down
CTSL3P	NR_027917	cathepsin L family member 3, pseudogene	-3.8634944	down	-3.0742848	down
LOC100128079	XR_243370	uncharacterized LOC100128079	-2.8896449	down	-3.0410738	down
			-4.901936	down	-2.6380951	down
			-2.478083	down	-3.6973255	down
BACE1	NM_012104	beta-site APP-cleaving enzyme 1	-3.6266146	down	-4.215377	down
KCNIP2	NM_173197	Kv channel interacting protein 2	-3.4221997	down	-2.0455768	down
TBC1D24	NM_020705	TBC1 domain family, member 24	-3.6651707	down	-2.0904098	down
PCA3	NR_015342	prostate cancer antigen 3 (non-protein coding)	-7.6312537	down	-3.2699623	down
PLA2G4D	NM_178034	phospholipase A2, group IVD (cytosolic)	-4.1246085	down	-2.7543018	down
			-4.4280047	down	-4.814678	down
GJC3	NM_181538	gap junction protein, gamma 3, 30.2kDa	-4.5795345	down	-7.2663884	down
			-2.112227	down	-2.6411629	down
WFDC5	NM_145652	WAP four-disulfide core domain 5	-4.577946	down	-5.2364945	down
LRRC14B	NM_001080478	leucine rich repeat containing 14B	-5.5541577	down	-4.1470437	down
LOC388849	NM_001243537	uncharacterized LOC388849	-3.8001928	down	-2.9330778	down
			-4.2258434	down	-3.5745382	down
BAD	AK309150	BCL2-associated agonist of cell death	-2.9449153	down	-2.5891025	down
			-2.0221806	down	-3.2103443	down
SPRR2C	NR_003062	small proline-rich protein 2C (pseudogene)	-4.56502	down	-5.268663	down
	BC029043		-4.4780912	down	-2.434501	down
LOC646652	XR_241774	integral membrane glycoprotein-like	-2.987425	down	-4.879596	down
LOC100128320	AK125438	uncharacterized LOC100128320	-5.8658676	down	-5.204045	down
MCCD1	NM_001011700	mitochondrial coiled-coil domain 1	-2.3733726	down	-2.6316607	down
SLC22A12	NM_144585	solute carrier family 22 (organic anion/urate transporter), member 12	-3.0122786	down	-2.382098	down
GPRC6A	NM_148963	G protein-coupled receptor, family C, group 6, member	-3.2741032	down	-2.0084155	down
LOC257152	AK001439	uncharacterized LOC257152	-3.951049	down	-4.5231023	down
LOC644083	XR_112044	uncharacterized LOC644083	-3.2993798	down	-2.031323	down
KIF18B	NM_001265577	kinesin family member 18B	-4.905325	down	-2.4329178	down

	AK127601		-8.0969925	down	-7.013296	down
FAM182B	NR_027061	family with sequence similarity 182, member B	-7.0808654	down	-4.446895	down
			-2.0131373	down	-2.805535	down
LOC645427	AK094407	uncharacterized LOC645427	-5.1247487	down	-2.940977	down
LOC646034	AK125175	uncharacterized LOC646034	-2.9871988	down	-2.8623116	down
SLC22A6	NM_153277	solute carrier family 22 (organic anion transporter), member 6	-2.112785	down	-3.6926575	down
OR2L3	NM_001004687	olfactory receptor, family 2, subfamily L, member 3	-2.5190835	down	-3.0904346	down
LOC284379		solute carrier family 7 (cationic amino acid transporter, y+ system), member 3 pseudogene	-3.352311	down	-2.5896008	down
LOC728093	XR_133401	putative POM121-like protein 1-like	-2.4229887	down	-3.1270144	down
LOC339240	NR_001443	keratin 17 pseudogene	-3.3057435	down	-2.251825	down
DNM1	AB209124	dynamin 1	-4.179987	down	-5.9209957	down
OR1K1	NM_080859	olfactory receptor, family 1, subfamily K, member 1	-2.5619006	down	-2.29827	down
TMEM239	NM_001167670	transmembrane protein 239	-3.2283669	down	-2.354593	down
LOC646743	NR_033930	uncharacterized LOC646743	-7.533138	down	-6.9335494	down
TNNT2	NM_000364	troponin T type 2 (cardiac)	-6.3595967	down	-3.6965022	down
KLK15	NM_017509	kallikrein-related peptidase 15	-4.519163	down	-2.576642	down
C9orf152	NM_001012993	chromosome 9 open reading frame 152	-3.7276387	down	-2.067756	down
SCGB1D2	NM_006551	secretoglobin, family 1D, member 2	-2.8465605	down	-2.367468	down
PRKG2	NM_006259	protein kinase, cGMP-dependent, type II	-3.4355297	down	-2.108568	down
ANKRD20A12P	NR_046228	ankyrin repeat domain 20 family, member A12, pseudogene	-4.8926783	down	-6.3074183	down
BBIP1	AK125829	BBSome interacting protein 1	-3.6637754	down	-2.659356	down
			-3.9294705	down	-4.5740514	down
			-3.3030953	down	-4.9121447	down
FLJ30403	NR_034159	uncharacterized LOC729975	-2.3147686	down	-5.3173976	down
PRR20B	NM_001130404	proline rich 20B	-3.0119767	down	-2.647472	down
	AI218952		-4.497109	down	-3.341208	down
			-2.5083578	down	-2.7077096	down
			-2.198738	down	-2.3534725	down
MUC13	NM_033049	mucin 13, cell surface associated	-13.06234	down	-6.926307	down
TTTY14	NR_001543	testis-specific transcript, Y-linked 14 (non-protein coding)	-3.7363346	down	-2.7651021	down
NT5E	NM_002526	5'-nucleotidase, ecto (CD73)	-3.0897279	down	-2.1316285	down
C2orf66	NM_213608	chromosome 2 open reading frame 66	-3.142606	down	-2.7817643	down
RFPL4AL1	NM_001277397	ret finger protein-like 4A-like 1	-3.8857641	down	-2.625456	down
GUCA1A	AK125780	guanylate cyclase activator 1A (retina)	-2.5478992	down	-2.2565725	down
CELA3A	AK308514	chymotrypsin-like elastase family, member 3A	-3.7255614	down	-5.3999424	down
	XM_005253817		-3.7453048	down	-3.0245314	down
DKK3	NM_015881	dickkopf WNT signaling pathway inhibitor 3	-3.3695397	down	-5.5479383	down
MYL3	NM_000258	myosin, light chain 3, alkali; ventricular, skeletal, slow	-5.2481933	down	-3.9522247	down
LOC101928125		major seminal plasma glycoprotein PSP-I-like	-3.309607	down	-3.6487744	down
SLC8A2	NM_015063	solute carrier family 8 (sodium/calcium exchanger), member 2	-3.363458	down	-3.599214	down
			-4.144339	down	-2.4550724	down
			-2.9484534	down	-2.266612	down
ASTL	NM_001002036	astacin-like metallo-endopeptidase (M12 family)	-4.6552873	down	-5.6348124	down
FAM47A	NM_203408	family with sequence similarity 47, member A	-2.3044775	down	-2.873728	down
OR2T1	NM_030904	olfactory receptor, family 2, subfamily T, member 1	-4.5176363	down	-2.457143	down
KCNJ13	NM_002242	potassium inwardly-rectifying channel, subfamily J, member 13	-6.919588	down	-3.466033	down
LINC00544		long intergenic non-protein coding RNA 544	-5.5293097	down	-3.2540586	down
	AK123300		-2.1097949	down	-2.0487065	down
LAMA4	NM_001105206	laminin, alpha 4	-6.2213125	down	-5.1228747	down
BCL2L15	NM_001010922	BCL2-like 15	-4.6121244	down	-3.1335475	down
SIGLEC17P	NR_047529	sialic acid binding Ig-like lectin 17, pseudogene	-3.6782072	down	-5.5102215	down
DOC2B	NM_003585	double C2-like domains, beta	-2.4733732	down	-2.101427	down
VKORC1	AK125618	vitamin K epoxide reductase complex, subunit 1	-2.8217468	down	-3.1096578	down
			-5.2779803	down	-5.4172783	down
LOC100652824	NM_001277372	uncharacterized protein KIAA2012	-3.4607103	down	-2.028815	down
ACSM5		acyl-CoA synthetase medium-chain family member 5	-5.2238708	down	-3.1996808	down
LINC00893	NR_027455	long intergenic non-protein coding RNA 893	-4.117779	down	-3.4121733	down
			-2.243266	down	-4.2110543	down
			-2.0180762	down	-3.2585142	down

			-3.647513	down	-2.1952505	down
SLC9A5	NM_004594	solute carrier family 9, subfamily A (NHE5, cation proton antiporter 5), member 5	-3.5455575	down	-2.5307686	down
			-2.1661193	down	-3.3831615	down
SPANXA1	NM_013453	sperm protein associated with the nucleus, X-linked, family member A1	-5.5813317	down	-3.1174965	down
MYH15	NM_014981	myosin, heavy chain 15	-3.7967823	down	-2.0230348	down
ATP4B	NM_000705	ATPase, H+/K+ exchanging, beta polypeptide	-2.11136	down	-3.2286546	down
			-8.568361	down	-5.982001	down
C9orf96	NM_153710	chromosome 9 open reading frame 96	-2.8538926	down	-2.5803359	down
FHAD1	NM_052929	forkhead-associated (FHA) phosphopeptide binding domain 1	-2.3698392	down	-2.1333544	down
ZNRD1-AS1	NR_026751	ZNRD1 antisense RNA 1	-3.3367116	down	-5.032544	down
			-2.6221402	down	-3.5694723	down
BMP10	NM_014482	bone morphogenetic protein 10	-2.3684022	down	-2.414793	down
PPAN-P2RY11	NM_001040664	PPAN-P2RY11 readthrough	-2.059526	down	-4.0220838	down
SPRY4	NM_030964	sprouty homolog 4 (Drosophila)	-3.192514	down	-2.0099323	down
HAPLN1	NM_001884	hyaluronan and proteoglycan link protein 1	-3.4948018	down	-2.2465894	down
			-3.942514	down	-2.542598	down
LYPD6	NM_001195685	LY6/PLAUR domain containing 6	-5.629027	down	-3.7093213	down
			-4.394614	down	-2.8566647	down
CST1	NM_001898	cystatin SN	-2.9084961	down	-2.5339808	down
SYNGAP1	NM_006772	synaptic Ras GTPase activating protein 1	-3.5952303	down	-2.971704	down
LOC647983	AK126828	uncharacterized LOC647983	-2.9117491	down	-4.001189	down
SMPX	NM_014332	small muscle protein, X-linked	-4.408218	down	-2.355111	down
TMEM191B	NM_001242313	transmembrane protein 191B	-3.9663272	down	-2.428717	down
TRIM31	NM_007028	tripartite motif containing 31	-4.3925486	down	-2.069862	down
	BC066548		-3.7657835	down	-2.1639297	down
GCM2	NM_004752	glial cells missing homolog 2 (Drosophila)	-8.538396	down	-4.5928836	down
SPDYE3	NM_001004351	speedy/RINGO cell cycle regulator family member E3	-9.048894	down	-5.2403007	down
C17orf47	NM_001038704	chromosome 17 open reading frame 47	-5.22768	down	-4.134124	down
C20orf112	NM_001256798	chromosome 20 open reading frame 112	-4.7333417	down	-3.826094	down
OLIG3	NM_175747	oligodendrocyte transcription factor 3	-4.4394217	down	-3.3683784	down
CELF5	NM_021938	CUGBP, Elav-like family member 5	-2.000706	down	-2.1728947	down
LOC100131831	AK129685	uncharacterized LOC100131831	-3.3401163	down	-2.1375268	down
			-3.3350067	down	-7.518144	down
OR2M5	NM_001004690	olfactory receptor, family 2, subfamily M, member 5	-6.7175207	down	-5.429307	down
FGF18	NM_003862	fibroblast growth factor 18	-3.16828	down	-2.57209	down
HOXB5	NM_002147	homeobox B5	-3.5866268	down	-3.2419593	down
LOC731656	NR_027454	uncharacterized LOC731656	-3.5993297	down	-2.717006	down
MS4A15	NM_001098835	membrane-spanning 4-domains, subfamily A, member 15	-5.9790115	down	-2.134461	down
CNTN2	NM_005076	contactin 2 (axonal)	-2.3980975	down	-4.524108	down
GPR133	NM_198827	G protein-coupled receptor 133	-2.1164494	down	-3.0372365	down
SLC7A14	NM_020949	solute carrier family 7, member 14	-4.6224732	down	-2.4758465	down
	CU687439		-2.6724308	down	-2.1815228	down
COL4A1	NM_001845	collagen, type IV, alpha 1	-5.886313	down	-4.864076	down
			-3.6251414	down	-2.1866405	down
ZC3H10	NM_032786	zinc finger CCCH-type containing 10	-2.0510392	down	-2.6616583	down
TGFB2	NM_003238	transforming growth factor, beta 2	-3.055837	down	-2.0163758	down
LOC644662		uncharacterized LOC644662	-4.680299	down	-2.0723808	down
C3orf20	NM_032137	chromosome 3 open reading frame 20	-4.7395577	down	-3.0207899	down
SVOPL	NM_174959	SVOP-like	-4.290916	down	-2.2200868	down
PSMB7		proteasome (prosome, macropain) subunit, beta type, 7	-2.6602936	down	-2.0117671	down
	AF001893		-4.0939503	down	-2.8376615	down
	XR_109656		-5.778005	down	-3.221395	down
AHNAK2	NM_138420	AHNAK nucleoprotein 2	-4.3639317	down	-3.2539978	down
SELV	NM_182704	selenoprotein V	-5.2477098	down	-3.2637463	down
GYG2	NM_001184703	glycogenin 2	-2.709407	down	-2.4951386	down
C11orf95	NM_001144936	chromosome 11 open reading frame 95	-2.1004827	down	-2.8758984	down
SSX4B	NM_001034832	synovial sarcoma, X breakpoint 4B	-9.031811	down	-5.78702	down
TDRD3	BC020604	tudor domain containing 3	-5.1527405	down	-3.1269019	down
TOX2	NM_032883	TOX high mobility group box family member 2	-3.155693	down	-2.1438344	down
	XM_005254829		-3.8228116	down	-2.3832526	down

MGC20647	BC008289	uncharacterized protein MGC20647	-3.8994372	down	-3.140553	down
			-2.648222	down	-2.009179	down
			-3.6317687	down	-3.9271653	down
MIXL1	NM_001282402	Mix paired-like homeobox	-3.0936568	down	-2.4086452	down
CES3	NM_024922	carboxylesterase 3	-3.923915	down	-3.7508008	down
CDRT15P2	CF272591	CMT1A duplicated region transcript 15 pseudogene 2	-8.116303	down	-5.4234653	down
CXorf38	AL832829	chromosome X open reading frame 38	-6.3632436	down	-2.774094	down
TSPAN8	NM_004616	tetraspanin 8	-3.9676056	down	-2.351582	down
LOC100289650	NR_036584	uncharacterized LOC100289650	-5.4919457	down	-5.134286	down
HULC	NR_004855	hepatocellular carcinoma up-regulated long non-coding RNA	-3.245721	down	-2.8277068	down
CP	NM_000096	ceruloplasmin (ferroxidase)	-3.8290896	down	-2.0804539	down
TTC28-AS1	NR_026963	TTC28 antisense RNA 1	-4.7788754	down	-5.1564555	down
			-4.681456	down	-3.246623	down
MYO5B	NM_001080467	myosin VB	-2.1622176	down	-2.735534	down
			-6.7268376	down	-4.5198603	down
GORAB	NM_001146039	golgin, RAB6-interacting	-4.460955	down	-2.1386724	down
CHIAP2		chitinase, acidic pseudogene 2	-2.6081417	down	-2.6871252	down
LOC100129223	XR_111243	uncharacterized LOC100129223	-4.7283764	down	-3.0323548	down
	BC064144		-3.631553	down	-2.941225	down
TRIM51	NM_032681	tripartite motif-containing 51	-6.7421284	down	-3.7017303	down
PRSS54	NM_001080492	protease, serine, 54	-2.3186297	down	-2.1586802	down
			-3.4967318	down	-3.143149	down
TMEM44	NM_001011655	transmembrane protein 44	-4.202736	down	-2.4158256	down
			-2.485159	down	-2.0613482	down
			-8.800126	down	-8.081863	down
SGK494	NM_001174103	uncharacterized serine/threonine-protein kinase SgK494	-3.9401453	down	-4.712771	down
UGT1A6	NM_001072	UDP glucuronosyltransferase 1 family, polypeptide A6	-3.616997	down	-2.7758641	down
LOC100130453	AK123920	uncharacterized LOC100130453	-2.8773363	down	-3.266371	down
RUFY4	NM_198483	RUN and FYVE domain containing 4	-3.350211	down	-4.517506	down
ARHGAP35	NM_004491	Rho GTPase activating protein 35	-2.086419	down	-2.017066	down
DEFT1P	NR_036686	defensin, theta 1 pseudogene	-5.412975	down	-2.2326682	down
TGIF1	NM_173209	TGFB-induced factor homeobox 1	-4.559707	down	-6.2166343	down
CCDC144A	NM_014695	coiled-coil domain containing 144A	-3.904522	down	-2.1359017	down
FLJ37786		uncharacterized LOC642691	-2.5663917	down	-2.85832	down
FTCD	NM_206965	formimidoyltransferase cyclodeaminase	-4.1456485	down	-2.718331	down
ADAT2	NM_001286259	adenosine deaminase, tRNA-specific 2	-2.19801	down	-2.2314584	down
TMEM25	NM_032780	transmembrane protein 25	-2.3513644	down	-2.5767536	down
			-4.0503774	down	-4.5056696	down
			-3.075554	down	-2.5572865	down
GSTM5	NM_000851	glutathione S-transferase mu 5	-2.0333214	down	-2.880986	down
	XR_110857		-6.3977985	down	-4.881606	down
CCDC11	NM_145020	coiled-coil domain containing 11	-3.2385538	down	-2.6664994	down
SPACA5	NM_205856	sperm acrosome associated 5	-2.7097774	down	-3.5503838	down
PTPRG	NM_002841	protein tyrosine phosphatase, receptor type, G	-2.5296814	down	-2.7297883	down
LOC100131894	AK092825	uncharacterized LOC100131894	-3.493357	down	-3.298152	down
HTR4	NM_001040173	5-hydroxytryptamine (serotonin) receptor 4, G protein-coupled	-4.4692106	down	-2.7669418	down
			-3.1720343	down	-2.4836795	down
ZNF763	NM_001012753	zinc finger protein 763	-2.7272363	down	-2.4495506	down
			-2.4312892	down	-2.4366586	down
LINC00222	NR_033376	long intergenic non-protein coding RNA 222	-3.6635182	down	-2.7653391	down
HIBCH	NM_198047	3-hydroxyisobutyryl-CoA hydrolase	-3.251146	down	-2.5109205	down
TMEM262	AK124141	transmembrane protein 262	-2.954637	down	-2.9576893	down
KIAA1244	NM_020340	KIAA1244	-4.133689	down	-2.0615048	down
LINC00263		long intergenic non-protein coding RNA 263	-2.277511	down	-2.3385224	down
			-2.7621043	down	-2.3800116	down
LOC101928356	XM_005260632	uncharacterized LOC101928356	-2.8883715	down	-2.077758	down
ACTA2-AS1	XR_110433	ACTA2 antisense RNA 1	-4.297376	down	-3.4432583	down
ADAM21	NM_003813	ADAM metallopeptidase domain 21	-2.22801	down	-2.1352837	down
SHISA9	NM_001145205	shisa family member 9	-3.1103396	down	-2.3256302	down
NR1D2	XM_005265639	nuclear receptor subfamily 1, group D, member 2	-4.5979977	down	-2.9418225	down
ZNF551	AK126625	zinc finger protein 551	-3.694569	down	-4.3313055	down
CHRNA5	NM_000745	cholinergic receptor, nicotinic, alpha 5 (neuronal)	-3.5252004	down	-3.8311088	down

GOLGA6A	NM_001038640	golgin A6 family, member A	-5.2765617	down	-4.515959	down
KRTAP4-1	NM_033060	keratin associated protein 4-1	-5.6619554	down	-7.800764	down
SLFN5	NM_144975	schlafen family member 5	-2.9985046	down	-2.7624593	down
XLOC_014512			-2.7244267	down	-2.930762	down
NKAIN4	NM_152864	Na+/K+ transporting ATPase interacting 4	-2.932312	down	-2.099535	down
SLC3A1	DQ023513	solute carrier family 3 (amino acid transporter heavy chain), member 1	-3.5304673	down	-2.5089746	down
PRRC2B	NM_013318	proline-rich coiled-coil 2B	-2.2755969	down	-3.4010828	down
LOC646719		uncharacterized LOC646719	-2.586085	down	-2.1726642	down
CCDC104	XM_005264120	coiled-coil domain containing 104	-4.388496	down	-4.960236	down
NUPL2		nucleoporin like 2	-4.1397505	down	-2.258889	down
IFNW1	NM_002177	interferon, omega 1	-3.816654	down	-2.479682	down
DEDD	XM_005245598	death effector domain containing	-3.7361922	down	-3.5773826	down
ZNF662	NM_207404	zinc finger protein 662	-2.2450626	down	-3.2179415	down
C1orf227	NM_001024601	chromosome 1 open reading frame 227	-3.972156	down	-2.218295	down
C21orf88	NR_026542	chromosome 21 open reading frame 88	-2.5638278	down	-2.2156951	down
			-2.8380506	down	-2.4456036	down
RPRML	NM_203400	reprimo-like	-3.238193	down	-2.5461533	down
PLA2G4F	NM_213600	phospholipase A2, group IVF	-2.3714466	down	-3.569584	down
TAS2R41	NM_176883	taste receptor, type 2, member 41	-2.146868	down	-2.5078769	down
CACNA2D1	NM_000722	calcium channel, voltage-dependent, alpha 2/delta subunit 1	-2.2712677	down	-2.2805147	down
H2AFB2	NM_001017991	H2A histone family, member B2	-2.6558301	down	-3.7219415	down
MC3R	NM_019888	melanocortin 3 receptor	-2.5054488	down	-2.5158591	down
CSPG4P8	NR_033579	chondroitin sulfate proteoglycan 4 pseudogene 8	-2.6950886	down	-2.8473406	down
LBP	NM_004139	lipopolysaccharide binding protein	-2.8356764	down	-2.1001062	down
CYP1A2	NM_000761	cytochrome P450, family 1, subfamily A, polypeptide 2	-3.0114543	down	-2.5994556	down
LOC286272	AK093004	uncharacterized LOC286272	-2.0728724	down	-2.0242498	down
			-2.900329	down	-3.5111523	down
SPRED3	NR_073032	sprouty-related, EVH1 domain containing 3	-5.852899	down	-3.9820526	down
	BC130558		-2.3949933	down	-2.5987318	down
TARM1	NM_001135686	T cell-interacting, activating receptor on myeloid cells 1	-3.3468943	down	-2.3951137	down
RAI1	AJ271790	retinoic acid induced 1	-2.5672915	down	-2.4362583	down
VCX2	NM_016378	variable charge, X-linked 2	-7.5590267	down	-4.6333914	down
			-4.5514884	down	-3.5649886	down
FLG	NM_002016	filaggrin	-2.2354674	down	-3.173141	down
SLC17A6	NM_020346	solute carrier family 17 (vesicular glutamate transporter), member 6	-4.472577	down	-6.0544634	down
LACRT	NM_033277	lacritin	-3.4390855	down	-2.9059248	down
LOC100126784	NR_015384	uncharacterized LOC100126784	-2.4430256	down	-3.4552524	down
TRIP13	NM_001166260	thyroid hormone receptor interactor 13	-2.0666785	down	-2.2269318	down
LOC100127909	AK130422	uncharacterized LOC100127909	-3.7166522	down	-3.1288	down
DUSP8	NM_004420	dual specificity phosphatase 8	-4.888023	down	-2.5769916	down
LOC400548		uncharacterized LOC400548	-2.3815918	down	-2.40036	down
			-2.557638	down	-4.2483263	down
CSNK1A1P1	NR_027320	casein kinase 1, alpha 1 pseudogene 1	-2.293013	down	-2.1933153	down
OR2L8	NM_001001963	olfactory receptor, family 2, subfamily L, member 8	-2.740557	down	-2.1067717	down
KRTAP1-5	NM_031957	keratin associated protein 1-5	-2.711638	down	-2.1074543	down
TMEM132A	NM_017870	transmembrane protein 132A	-2.877574	down	-2.3369367	down
LOC100131860	AK097109	uncharacterized LOC100131860	-3.108029	down	-2.086315	down
SPRR2F	NM_001014450	small proline-rich protein 2F	-2.3785138	down	-2.3916743	down
SLC3A1	NM_000341	solute carrier family 3 (amino acid transporter heavy chain), member 1	-13.755996	down	-9.613231	down
BEST3	NM_001282614	bestrophin 3	-2.1513548	down	-3.3493996	down
TMEM35	NM_021637	transmembrane protein 35	-8.613536	down	-4.691395	down
OPN1MW	NM_000513	opsin 1 (cone pigments), medium-wave-sensitive	-4.2184377	down	-3.7852883	down
KLK2	NM_001002231	kallikrein-related peptidase 2	-3.3831027	down	-3.8603814	down
NCKAP5L	BC110599	NCK-associated protein 5-like	-3.9752944	down	-3.3476906	down
CAV1	NM_001753	caveolin 1, caveolae protein, 22kDa	-3.3506534	down	-2.6581335	down
LOC285095	XM_005275940	uncharacterized LOC285095	-3.403231	down	-2.771608	down
IQCH	NM_001031715	IQ motif containing H	-3.459624	down	-2.1537576	down
FMO2	NM_001460	flavin containing monooxygenase 2 (non-functional)	-3.2999504	down	-2.3110301	down
CRYGN	NM_144727	crystallin, gamma N	-2.5662577	down	-2.383322	down
TBX2	NM_005994	T-box 2	-5.112472	down	-2.4387703	down

ALDOAP2	M21191	aldolase A, fructose-bisphosphate pseudogene 2	-3.914163	down	-2.0807843	down
			-2.0556438	down	-2.3149915	down
NRAP	NM_198060	nebulin-related anchoring protein	-4.2681665	down	-2.4597619	down
C6orf132	NM_001164446	chromosome 6 open reading frame 132	-7.083013	down	-9.727346	down
DNAH12	NM_178504	dynein, axonemal, heavy chain 12	-2.7406645	down	-2.3318284	down
ABCG8	NM_022437	ATP-binding cassette, sub-family G (WHITE), member	-8.683227	down	-4.009081	down
	XR_245014		-3.322607	down	-3.3536286	down
ANKRD20A9P	NR_027995	ankyrin repeat domain 20 family, member A9, pseudogene	-4.0775495	down	-2.7862835	down
MRO	NM_031939	maestro	-3.7212763	down	-2.1969876	down
RHBDD1	XM_005246899	rhomboid domain containing 1	-4.405751	down	-4.3730392	down
			-7.0380025	down	-10.683768	down
FAM25A	NM_001146157	family with sequence similarity 25, member A	-4.29874	down	-5.3189464	down
DPYSL5	NM_020134	dihydropyrimidinase-like 5	-6.0256314	down	-3.9440286	down
LOC100509091	XM_003403877	extracellular matrix protein 2-like	-4.0398355	down	-2.1500516	down
SPACA5	NM_205856	sperm acrosome associated 5	-3.7635021	down	-2.5995908	down
LOC644285	AK126853	uncharacterized LOC644285	-2.5874307	down	-2.3458006	down
LDHAL6A	NM_144972	lactate dehydrogenase A-like 6A	-3.1628232	down	-2.3466198	down
LOC100131000	AK124361	uncharacterized LOC100131000	-2.672186	down	-3.218077	down
DHH	NM_021044	desert hedgehog	-4.5250783	down	-2.851316	down
FLJ12825	NR_026655	uncharacterized LOC440101	-2.9325914	down	-2.1450946	down
ZAR1	NM_175619	zygote arrest 1	-2.529602	down	-4.3487625	down
Unknown			-2.681653	down	-2.0454698	down
KRT6B	NM_005555	keratin 6B	-2.886168	down	-2.2352533	down
PRSS55	NM_198464	protease, serine, 55	-2.013933	down	-3.3137004	down
SEC14L6		SEC14-like 6 (S. cerevisiae)	-6.6565104	down	-3.011952	down
LOC728061	AK025151	hCG2003663	-2.101864	down	-2.5644073	down
FAM198A	AK091001	family with sequence similarity 198, member A	-4.235054	down	-3.992717	down
LOC100130849		phosphorylase kinase, gamma 1 (muscle) pseudogene	-3.8368144	down	-3.8647861	down
Unknown			-4.50916	down	-4.554587	down
	AK096324		-3.5536737	down	-2.1652827	down
OR14A16	NM_001001966	olfactory receptor, family 14, subfamily A, member 16	-4.8684325	down	-2.6017656	down
Unknown			-2.7589223	down	-2.4328039	down
FLJ46906	NR_033896	uncharacterized LOC441172	-2.6454606	down	-2.533102	down
C20orf78	CR738909	chromosome 20 open reading frame 78	-2.5147624	down	-2.1220982	down
	DB066855		-5.3450966	down	-5.1644583	down
INMT	NM_001199219	indolethylamine N-methyltransferase	-2.7871006	down	-2.257948	down
PKDCC	AK311155	protein kinase domain containing, cytoplasmic	-8.488906	down	-2.9054477	down
TAS2R19	NM_176888	taste receptor, type 2, member 19	-4.191179	down	-2.273835	down
Unknown			-2.8093636	down	-3.831292	down
ZNF81	NM_007137	zinc finger protein 81	-5.50009	down	-3.3331683	down
Unknown			-2.9515224	down	-2.6774642	down
XLOC_004729			-4.5539103	down	-5.732493	down
	XR_252542		-3.7279403	down	-4.7672544	down
Unknown			-2.1732457	down	-2.010095	down
RAB43	BM917410	RAB43, member RAS oncogene family	-4.005407	down	-4.0891805	down
Unknown			-3.6262307	down	-9.106207	down
ABI3BP	NM_015429	ABI family, member 3 (NESH) binding protein	-4.010035	down	-2.7335498	down
	AK127417		-2.2932725	down	-2.0540762	down
Unknown			-2.2990296	down	-2.3209527	down
LINC00917		long intergenic non-protein coding RNA 917	-3.3895245	down	-3.1207476	down
FLJ11710	AK021772	uncharacterized protein FLJ11710	-4.07118	down	-3.912566	down
LOC100129098	XM_001714893	WAS/WASL-interacting protein family member 1-like	-4.818137	down	-2.7728262	down
CPXM2	NM_198148	carboxypeptidase X (M14 family), member 2	-2.9014094	down	-3.7511039	down
CCDC103	NM_001258397	coiled-coil domain containing 103	-2.0293207	down	-2.7786374	down
INO80	NM_017553	INO80 complex subunit	-2.0089707	down	-2.2209868	down
AQP7P3		aquaporin 7 pseudogene 3	-2.9169405	down	-2.6258645	down
PRRT2	NM_145239	proline-rich transmembrane protein 2	-2.8965902	down	-2.5783308	down
LCNL1	NM_207510	lipocalin-like 1	-2.3413348	down	-3.0202014	down
PRAMEF7	NM_001012277	PRAME family member 7	-4.934108	down	-2.3781807	down
CNTNAP4	NM_138994	contactin associated protein-like 4	-5.6010222	down	-2.8021007	down
OR5T3	NM_001004747	olfactory receptor, family 5, subfamily T, member 3	-4.4067535	down	-2.5306466	down
Unknown			-2.2567105	down	-2.3137233	down

OR2H2	NM_007160	olfactory receptor, family 2, subfamily H, member 2	-2.355751	down	-3.0151265	down
SOD3	NM_003102	superoxide dismutase 3, extracellular	-4.3885098	down	-2.8318462	down
MFSD6L	NM_152599	major facilitator superfamily domain containing 6-like	-7.2669406	down	-3.7301908	down
	XR_242003		-2.6000671	down	-2.7394753	down
	AK090928		-2.3281045	down	-2.9178474	down
	XR_111551		-6.3857136	down	-6.3807073	down
Unknown			-2.4589067	down	-2.7157795	down
SLC5A4	NM_014227	solute carrier family 5 (glucose activated ion channel), member 4	-3.3165627	down	-2.34644	down
	XR_247160		-3.223451	down	-2.5894132	down
CREB3L4	NM_130898	cAMP responsive element binding protein 3-like 4	-3.1609852	down	-2.0981402	down
PTPRG	NM_002841	protein tyrosine phosphatase, receptor type, G	-2.362611	down	-2.2563598	down
	XM_005276096		-2.4808502	down	-3.227337	down
TEX19	NM_207459	testis expressed 19	-4.444819	down	-2.0959647	down
SOX13	BC040649	SRY (sex determining region Y)-box 13	-2.7995327	down	-2.0764039	down
SPC24	NM_182513	SPC24, NDC80 kinetochore complex component	-3.5928822	down	-2.0852225	down
LINC00114	AY204748	long intergenic non-protein coding RNA 114	-5.0722156	down	-2.791678	down
RARB	BC069735	retinoic acid receptor, beta	-5.4694147	down	-3.9027586	down
PSG5	BG354573	pregnancy specific beta-1-glycoprotein 5	-5.4630227	down	-3.4516673	down
Unknown			-5.184938	down	-2.7488794	down
FAM86B3P		family with sequence similarity 86, member A pseudogene	-2.1150413	down	-3.0564413	down
Unknown			-11.828863	down	-6.469771	down
Unknown			-4.2464	down	-5.801462	down
TMCO4	NM_181719	transmembrane and coiled-coil domains 4	-2.593795	down	-2.375089	down
LOC100129917	NR_036511	uncharacterized LOC100129917	-5.6742225	down	-2.1845236	down
IL20RA	AK098312	interleukin 20 receptor, alpha	-2.7197917	down	-2.0692644	down
S100A5	NM_002962	S100 calcium binding protein A5	-4.0946097	down	-3.9728982	down
GLIS3	DQ438879	GLIS family zinc finger 3	-4.8215327	down	-3.84525	down
LOC100131581	AK092544	uncharacterized LOC100131581	-4.264229	down	-6.2694902	down
NEK2	NM_002497	NIMA-related kinase 2	-5.203363	down	-2.6656015	down
GPM6B	NM_001001996	glycoprotein M6B	-2.152969	down	-3.0386808	down
	AF289562		-2.324719	down	-2.5417893	down
ZNF316		zinc finger protein 316	-2.2313414	down	-2.1793094	down
Unknown			-3.3035352	down	-2.4414911	down
PTGER3	NM_198715	prostaglandin E receptor 3 (subtype EP3)	-2.3494437	down	-2.3230703	down
	XR_112958		-7.2895436	down	-3.5592332	down
Unknown			-10.18425	down	-4.4264274	down
CPNE9	NM_153635	copine family member IX	-3.292837	down	-2.8515382	down
DCLK1	NM_004734	doublecortin-like kinase 1	-4.5513983	down	-4.0360823	down
WFIKKN2	NM_175575	WAP, follistatin/kazal, immunoglobulin, kunitz and netrin domain containing 2	-6.0906954	down	-2.9805276	down
MUC3	AF007191	intestinal mucin-like	-2.6964486	down	-2.2538464	down
DNM3	NM_001278252	dynamin 3	-3.4509933	down	-2.1572034	down
WNK4	NM_032387	WNK lysine deficient protein kinase 4	-2.8895853	down	-2.4359827	down
FAM189A2	NM_004816	family with sequence similarity 189, member A2	-2.698642	down	-2.1782188	down
KRBOX1	NM_001205272	KRAB box domain containing 1	-2.6187804	down	-2.0611854	down
08-sep	NM_001098811	septin 8	-2.5213604	down	-2.7487297	down
Unknown			-3.9527194	down	-3.4421222	down
LOC285740	NR_027114	uncharacterized LOC285740	-7.3191624	down	-3.6962438	down
BMP3	NM_001201	bone morphogenetic protein 3	-4.0776105	down	-4.1398873	down
SZT2	BC051343	seizure threshold 2 homolog (mouse)	-3.7157001	down	-2.9513226	down
	AK125954		-2.4387221	down	-3.079703	down
FAM138E	NR_026819	family with sequence similarity 138, member E	-2.3055542	down	-2.0997684	down
L3MBTL4	NM_173464	l(3)mbt-like 4 (Drosophila)	-3.7521877	down	-2.481974	down
Unknown			-4.3694143	down	-3.3554232	down
TBCB		tubulin folding cofactor B	-2.3452904	down	-2.6268542	down
MAGEC3	NM_138702	melanoma antigen family C, 3	-5.0959487	down	-3.0491858	down
FRAS1	AK092082	Fraser syndrome 1	-3.6836667	down	-2.0997248	down
FBXO27	NM_178820	F-box protein 27	-3.9830894	down	-3.283798	down
GPR101	NM_054021	G protein-coupled receptor 101	-3.0422552	down	-2.0174198	down
LOC100131581	AK092544	uncharacterized LOC100131581	-3.0183983	down	-4.8780975	down
