# Supplementary material for: Transcriptional signature associated with early rheumatoid arthritis and healthy individuals at high risk to develop the disease
Source: PLoS One. 2018 Mar 27;13(3):e0194205. doi: 10.1371/journal.pone.0194205 (PMC5870959; doi:10.1371/journal.pone.0194205)
Supplement: S8 Table — (PDF) [file pone.0194205.s008.pdf]

Supplementary table 8. Down regulated genes in AR and up regulated genes in ACCP+ groups acording Venn diagram

Gene Symbol	Genbank Accession	Gene Name	ACCP+		RA	
			Fold Change	Regulation	Fold Change	Regulation
SCARF2	NM_153334	scavenger receptor class F, member 2	2.360706	up	-2.2257302	down
LOC389834	NR_027420	ankyrin repeat domain 57 pseudogene	2.7860982	up	-2.426158	down
DDX3Y	NM_001122665	DEAD (Asp-Glu-Ala-Asp) box helicase 3, Y-linked	2.0400486	up	-2.8573813	down
ACTN4	NM_004924	actinin, alpha 4	2.3923786	up	-2.0808754	down
USP21	NM_001014443	ubiquitin specific peptidase 21	2.651004	up	-2.2606707	down
HMGA2	NM_003484	high mobility group AT-hook 2	3.1459346	up	-2.9009023	down
TMEM151B	NM_001137560	transmembrane protein 151B	2.4972787	up	-2.982902	down
LOC100130238	NR_024563	uncharacterized LOC100130238	2.1249104	up	-2.4025908	down
ZNF205	NM_003456	zinc finger protein 205	3.350588	up	-3.5070689	down
CLPTM1	NM_001294	cleft lip and palate associated transmembrane protein 1	2.178986	up	-2.5246377	down
KCTD19	NM_001100915	potassium channel tetramerization domain containing 19	3.0291438	up	-2.0930295	down
COL6A2	NM_058174	collagen, type VI, alpha 2	2.97253	up	-2.2731335	down
NPB	NM_148896	neuropeptide B	2.888444	up	-2.6334124	down
LOC729732	XR_159504	uncharacterized LOC729732	2.6223218	up	-2.2632632	down
PCSK1N	NM_013271	proprotein convertase subtilisin/kexin type 1 inhibitor	3.5376828	up	-4.017537	down
ZNF771	NM_016643	zinc finger protein 771	2.0332878	up	-2.7068307	down
SPRY1	NM_001258038	sprouty homolog 1, antagonist of FGF signaling (Drosophila)	2.126156	up	-3.9324708	down
PFKL	NM_002626	phosphofructokinase, liver	3.2058015	up	-4.5982337	down
CHI3L1	NM_001276	chitinase 3-like 1 (cartilage glycoprotein-39)	2.4524612	up	-2.1526206	down
SPSB4	NM_080862	splA/ryanodine receptor domain and SOCS box containing 4	2.1544807	up	-2.0252662	down
BHLHE23	NM_080606	basic helix-loop-helix family, member e23	2.8559263	up	-2.953399	down
SUSD4		sushi domain containing 4	2.3678844	up	-2.1846018	down
LOC100130557	NR_024567	uncharacterized LOC100130557	2.0982842	up	-2.0409	down
TPGS1	NM_033513	tubulin polyglutamylase complex subunit 1	2.06661	up	-2.420084	down
	S60780		2.1208236	up	-2.0055678	down
A4GALT	NM_017436	alpha 1,4-galactosyltransferase	2.2207155	up	-4.01797	down
ZNF329	XR_243958	zinc finger protein 329	2.294157	up	-2.483955	down
PYY2		peptide YY, 2 (pseudogene)	2.2559474	up	-2.9844809	down
USP21	NM_001014443	ubiquitin specific peptidase 21	2.0056686	up	-2.4280882	down
CMIP	NM_198390	c-Maf inducing protein	2.1697936	up	-2.3066244	down
SLC11A1	NM_000578	solute carrier family 11 (proton-coupled divalent metal ion transporter), member 1	2.160759	up	-2.4163134	down
KCNQ2	NM_172109	potassium voltage-gated channel, KQT-like subfamily, member 2	2.3352866	up	-2.9891136	down
