# Supplementary material for: Transcriptional signature associated with early rheumatoid arthritis and healthy individuals at high risk to develop the disease
Source: PLoS One. 2018 Mar 27;13(3):e0194205. doi: 10.1371/journal.pone.0194205 (PMC5870959; doi:10.1371/journal.pone.0194205)
Supplement: S9 Table — (PDF) [file pone.0194205.s009.pdf]

Supplementary table 9. Up regulated genes in AR and down regulated genes in ACCP+ groups acording Venn diagram

Gene Symbol	Genbank Accession	Gene Name	ACCP+		RA	
			Fold Change	Regulation	Fold Change	Regulation
CCDC175		coiled-coil domain containing 175	-2.5091715	down	2.0528383	up
ETV4	NM_001079675	ets variant 4	-2.0932825	down	2.078938	up
LOC100131138	NR_036513	uncharacterized LOC100131138	-2.0074818	down	2.896989	up
PBX1	NM_002585	pre-B-cell leukemia homeobox 1	-2.457484	down	2.5033636	up
KCNK17	NM_031460	potassium channel, subfamily K, member 17	-2.125552	down	2.0954254	up
	XR_248979		-2.9701798	down	2.2098558	up
ERBB3	NM_001982	v-erb-b2 avian erythroblastic leukemia viral oncogene homolog 3	-2.0647767	down	2.1594281	up
ANP32E	NM_030920	acidic (leucine-rich) nuclear phosphoprotein 32 family, member E	-2.1112173	down	2.136219	up
PPCS		phosphopantothenoylcysteine synthetase	-2.3279622	down	2.4874487	up
TAS2R50	NM_176890	taste receptor, type 2, member 50	-2.0667639	down	2.1007218	up
DIXDC1	NM_001037954	DIX domain containing 1	-2.3925838	down	2.7631285	up
	XR_109175		-3.8947074	down	2.015282	up
GEMIN2	NM_003616	gem (nuclear organelle) associated protein 2	-2.5096903	down	2.307731	up
Unknown			-2.619015	down	2.6360404	up
NPPA	NM_006172	natriuretic peptide A	-2.1464565	down	2.326938	up
LRTM1	NM_020678	leucine-rich repeats and transmembrane domains 1	-2.4161856	down	2.12895	up
TAF1	NM_001286074	TAF1 RNA polymerase II, TATA box binding protein (TBP)-associated factor, 250kDa	-2.0174625	down	2.2413568	up
Unknown			-2.630963	down	2.2269216	up
PCDH9	BC150296	protocadherin 9	-3.0894706	down	2.9098659	up
ERV18-1	AK126787	endogenous retrovirus group 18, member 1	-2.1419477	down	2.1447368	up
RCAN2	NM_005822	regulator of calcineurin 2	-2.2815917	down	2.8625188	up
KCND3	NM_004980	potassium voltage-gated channel, Shal-related subfamily, member 3	-2.090463	down	2.0554535	up
TPD52L3	NM_001001874	tumor protein D52-like 3	-2.4482148	down	3.119155	up
IGFL2	NM_001002915	IGF-like family member 2	-2.614197	down	2.4721038	up
ARGLU1	XR_243043	arginine and glutamate rich 1	-2.4347777	down	2.158685	up
TFPI	NM_006287	tissue factor pathway inhibitor (lipoprotein-associated coagulation inhibitor)	-2.2367547	down	2.8298364	up
Unknown			-2.1864674	down	2.0243075	up
Unknown			-2.4927843	down	2.062969	up
TRPA1	NM_007332	transient receptor potential cation channel, subfamily A, member 1	-2.0856848	down	2.3513865	up
CCL11	NM_002986	chemokine (C-C motif) ligand 11	-2.5958261	down	2.0526867	up
XG	NM_175569	Xg blood group	-2.021248	down	2.3177845	up
Unknown			-2.5485678	down	2.5991683	up
COL4A2-AS2	XR_158875	COL4A2 antisense RNA 2	-3.0004354	down	2.1227462	up
GJA10	NM_032602	gap junction protein, alpha 10, 62kDa	-2.3321197	down	2.4125774	up
NCS1	NM_014286	neuronal calcium sensor 1	-2.7745311	down	2.0359647	up
CDADC1	NM_030911	cytidine and dCMP deaminase domain containing 1	-3.2822497	down	2.978757	up
FABP4	NM_001442	fatty acid binding protein 4, adipocyte	-2.3255286	down	2.1605802	up
