# Supplementary material for: Transcriptional signature associated with early rheumatoid arthritis and healthy individuals at high risk to develop the disease
Source: PLoS One. 2018 Mar 27;13(3):e0194205. doi: 10.1371/journal.pone.0194205 (PMC5870959; doi:10.1371/journal.pone.0194205)
Supplement: S10 Table — (PDF) [file pone.0194205.s010.pdf]

Suplementary table 10. Biological function of the 2 fold change Up-regulated genes according GO analisis in relatives with ACCP+

Biological function	Gene Symbol	Gene Name	Genebank Accession
Innate immune response	CORO1A	coronin, actin binding protein, 1A	NM_007074
	APOBEC3C	apolipoprotein B mRNA editing enzyme, catalytic polypeptide-like 3C	NM_014508
	LGALS3	lectin, galactoside-binding, soluble, 3	NM_002306
	NRROS	negative regulator of reactive oxygen species	NM_198565
	DUSP4	dual specificity phosphatase 4	NM_001394
	GAPDH	glyceraldehyde-3-phosphate dehydrogenase	NM_002046
	FCER1G	Fc fragment of IgE, high affinity I, receptor for; gamma polypeptide	NM_004106
	MAPKAPK2	mitogen-activated protein kinase-activated protein kinase 2	NM_004759
	OAS2	2'-5'-oligoadenylate synthetase 2, 69/71kDa	NM_016817
	HSP90B1	heat shock protein 90kDa beta (Grp94), member 1	NM_003299
	NLRC5	NLR family, CARD domain containing 5	NM_032206
	MYD88	myeloid differentiation primary response 88	NM_002468
	CACTIN	cactin, spliceosome C complex subunit	NM_021231
	CXCL16	chemokine (C-X-C motif) ligand 16	NM_022059
	ITGB2	integrin, beta 2 (complement component 3 receptor 3 and 4 subunit)	L78790
	CAMK2G	calcium/calmodulin-dependent protein kinase II gamma	NM_172171
	OAS1	2'-5'-oligoadenylate synthetase 1, 40/46kDa	NM_002534
	UBE2L6	ubiquitin-conjugating enzyme E2L 6	NM_198183
	IFITM3	interferon induced transmembrane protein 3	NM_021034
	PHLPP1	PH domain and leucine rich repeat protein phosphatase 1	NM_194449
	UNC93B1	unc-93 homolog B1 (C. elegans)	NM_030930
	CD4	CD4 molecule	NM_000616
	IRF7	interferon regulatory factor 7	NM_004031
	WASF2	WAS protein family, member 2	AK075231
	AIF1	allograft inflammatory factor 1	NM_004847
	SLC11A1	solute carrier family 11 (proton-coupled divalent metal ion transporter), member 1	NM_000578
	POLR1C	polymerase (RNA) I polypeptide C, 30kDa	CA416988
	CSF1	colony stimulating factor 1 (macrophage)	NM_172212
	PDPK1	3-phosphoinositide dependent protein kinase 1	AF111845
	IFNAR2	interferon (alpha, beta and omega) receptor 2	NM_000874
	HLA-DRB3	major histocompatibility complex, class II, DR beta 3	NM_022555
	UNC93B1	unc-93 homolog B1 (C. elegans)	NM_030930
	SAMHD1	SAM domain and HD domain 1	NM_015474
Interferon type 1 response	OAS2	2'-5'-oligoadenylate synthetase 2, 69/71kDa	NM_016817
	OAS1	2'-5'-oligoadenylate synthetase 1, 40/46kDa	NM_002534
	IFITM3	interferon induced transmembrane protein 3	NM_021034
	IRF7	interferon regulatory factor 7	NM_004031
	IFNAR2	interferon (alpha, beta and omega) receptor 2	NM_000874
Interferon gamma response	GAPDH	glyceraldehyde-3-phosphate dehydrogenase	NM_002046
	OAS2	2'-5'-oligoadenylate synthetase 2, 69/71kDa	NM_016817
	CXCL16	chemokine (C-X-C motif) ligand 16	NM_022059
	CAMK2G	calcium/calmodulin-dependent protein kinase II gamma	NM_172171
	OAS1	2'-5'-oligoadenylate synthetase 1, 40/46kDa	NM_002534
	IFITM3	interferon induced transmembrane protein 3	NM_021034
	IRF7	interferon regulatory factor 7	NM_004031
	AIF1	allograft inflammatory factor 1	NM_004847
	SLC11A1	solute carrier family 11 (proton-coupled divalent metal ion transporter), member 1	NM_000578
	HLA-DRB3	major histocompatibility complex, class II, DR beta 3	NM_022555
Leucocyte activation	FCER1G	Fc fragment of IgE, high affinity I, receptor for; gamma polypeptide	NM_004106
	ITGAL	integrin, alpha L (antigen CD11A (p180), lymphocyte function-associated antigen 1; alpha poly	NM_002209
	CCND3	cyclin D3	NM_001760
	CD3E	CD3e molecule, epsilon (CD3-TCR complex)	NM_000733
	HDAC5	histone deacetylase 5	NM_001015053
	CD4	CD4 molecule	NM_000616
	NKX2-3	NK2 homeobox 3	NM_145285
	AIF1	allograft inflammatory factor 1	NM_004847

	LYL1	lymphoblastic leukemia associated hematopoiesis regulator 1	NM_005583
	SELPLG	selectin P ligand	NM_001206609
	TNFRSF4	tumor necrosis factor receptor superfamily, member 4	NM_003327
	CPLX2	complexin 2	
	ZBTB32	zinc finger and BTB domain containing 32	NM_014383
	SLC11A1	solute carrier family 11 (proton-coupled divalent metal ion transporter), member 1	NM_000578
	CSF1	colony stimulating factor 1 (macrophage)	NM_172212
	Unknown	Homo sapiens cDNA FLJ39779 fis, clone SPLEN2001945. [AK097098]	AK097098
	FKBP1A	FK506 binding protein 1A, 12kDa	NM_000801
**Leucocyte migration**	CORO1A	coronin, actin binding protein, 1A	NM_007074
	CAV1	caveolin 1, caveolae protein, 22kDa	NM_001753
	C5AR1	complement component 5a receptor 1	NM_001736
	FCER1G	Fc fragment of IgE, high affinity I, receptor for; gamma polypeptide	NM_004106
	ITGAL	integrin, alpha L (antigen CD11A (p180), lymphocyte function-associated antigen 1; alpha poly	NM_002209
	CXCL16	chemokine (C-X-C motif) ligand 16	NM_022059
	ITGB2	integrin, beta 2 (complement component 3 receptor 3 and 4 subunit)	L78790
	MYO9B	myosin IXB	NM_004145
	SLC7A5	solute carrier family 7 (amino acid transporter light chain, L system), member 5	NM_003486
	NKX2-3	NK2 homeobox 3	NM_145285
	SELPLG	selectin P ligand	NM_001206609
	RET	ret proto-oncogene	NM_020630
**Myeloid lineage differentiation**	MFAP2	microfibrillar-associated protein 2	NM_017459
	RASGRP4	RAS guanyl releasing protein 4	NM_170604
	MAEA	macrophage erythroblast attacher	NM_001017405
	NBEAL2	neurobeachin-like 2	NM_015175
	JUNB	jun B proto-oncogene	NM_002229
	GATA1	GATA binding protein 1 (globin transcription factor 1)	NM_002049
	NKX2-3	NK2 homeobox 3	NM_145285
	ERCC2	excision repair cross-complementation group 2	NM_000400
	WASF2	WAS protein family, member 2	AK075231
	CSF1	colony stimulating factor 1 (macrophage)	NM_172212
	OCSTAMP	osteoclast stimulatory transmembrane protein	NM_080721
	HBZ	hemoglobin, zeta	NM_005332
**Leucocyte differentiation**	CD3E	CD3e molecule, epsilon (CD3-TCR complex)	NM_000733
	HDAC5	histone deacetylase 5	NM_001015053
	JUNB	jun B proto-oncogene	NM_002229
	CD4	CD4 molecule	NM_000616
	GATA1	GATA binding protein 1 (globin transcription factor 1)	NM_002049
	NKX2-3	NK2 homeobox 3	NM_145285
	LYL1	lymphoblastic leukemia associated hematopoiesis regulator 1	NM_005583
	CSF1	colony stimulating factor 1 (macrophage)	NM_172212
	Unknown	Homo sapiens cDNA FLJ39779 fis, clone SPLEN2001945. [AK097098]	AK097098
	OCSTAMP	osteoclast stimulatory transmembrane protein	NM_080721
**e-containing compound catabolic process**	MOV10L1	Mov10 RISC complex RNA helicase like 1	NM_018995
	GNB1	guanine nucleotide binding protein (G protein), beta polypeptide 1	NM_002074
	ASNA1	arsA arsenite transporter, ATP-binding, homolog 1 (bacterial)	NM_004317
	RNF213	ring finger protein 213	NM_001256071
	TAPBP	TAP binding protein (tapasin)	NM_003190
	ATP6V0D1	ATPase, H+ transporting, lysosomal 38kDa, V0 subunit d1	NM_004691
	TUBA3D	tubulin, alpha 3d	NM_080386
	NOA1	nitric oxide associated 1	NM_032313
	MYH14	myosin, heavy chain 14, non-muscle	NM_001077186
	SRPR	signal recognition particle receptor (docking protein)	NM_003139
	TUFM	Tu translation elongation factor, mitochondrial	NM_003321
	MYO9B	myosin IXB	NM_004145
	ERCC2	excision repair cross-complementation group 2	NM_000400
	GNG8	guanine nucleotide binding protein (G protein), gamma 8	NM_033258
	MRAS	muscle RAS oncogene homolog	NM_012219
	PSMC4	proteasome (prosome, macropain) 26S subunit, ATPase, 4	NM_006503

Purir	DDX3Y	DEAD (Asp-Glu-Ala-Asp) box helicase 3, Y-linked	NM_004660
	GPX1	glutathione peroxidase 1	NM_201397
	ARFRP1	ADP-ribosylation factor related protein 1	
	ATP1A3	ATPase, Na+/K+ transporting, alpha 3 polypeptide	NM_001256214
	CHD1L	chromodomain helicase DNA binding protein 1-like	NM_004284
	RHOG	ras homolog family member G	NM_001665
	NKIRAS2	NFKB inhibitor interacting Ras-like 2	NM_001144927
	PDE4A	phosphodiesterase 4A, cAMP-specific	NM_001111307
Cellular response to cytokines stimulus	CORO1A	coronin, actin binding protein, 1A	NM_007074
	ACSL1	acyl-CoA synthetase long-chain family member 1	NM_001995
	CHI3L1	chitinase 3-like 1 (cartilage glycoprotein-39)	NM_001276
	GAPDH	glyceraldehyde-3-phosphate dehydrogenase	NM_002046
	IL2RG	interleukin 2 receptor, gamma	NM_000206
	OAS2	2'-5'-oligoadenylate synthetase 2, 69/71kDa	NM_016817
	EIF4G1	eukaryotic translation initiation factor 4 gamma, 1	NM_182917
	RPS6KA4	ribosomal protein S6 kinase, 90kDa, polypeptide 4	NM_003942
	CACTIN	cactin, spliceosome C complex subunit	NM_021231
	CAMK2G	calcium/calmodulin-dependent protein kinase II gamma	NM_172171
	OAS1	2'-5'-oligoadenylate synthetase 1, 40/46kDa	NM_002534
	UBE2L6	ubiquitin-conjugating enzyme E2L 6	NM_198183
	IFITM3	interferon induced transmembrane protein 3	NM_021034
	STAT3	signal transducer and activator of transcription 3 (acute-phase response factor)	NM_213662
	IRF7	interferon regulatory factor 7	NM_004031
	IL1R2	interleukin 1 receptor, type II	NM_004633
	AIF1	allograft inflammatory factor 1	NM_004847
	CSF2RA	colony stimulating factor 2 receptor, alpha, low-affinity (granulocyte-macrophage) [Source:HGNC Symbol;Acc:HGNC:2435] [ENST00000381524]	HGNC:2435
	SELPLG	selectin P ligand	NM_001206609
	IFNLR1	interferon, lambda receptor 1	NM_170743
	TNFRSF4	tumor necrosis factor receptor superfamily, member 4	NM_003327
	IFNAR2	interferon (alpha, beta and omega) receptor 2	NM_000874
	HLA-DRB3	major histocompatibility complex, class II, DR beta 3	NM_022555
	OCSTAMP	osteoclast stimulatory transmembrane protein	NM_080721
	RAE1	ribonucleic acid export 1	NM_001015885
Cellular response to organonitrogen compounds	ATP6V0B	ATPase, H+ transporting, lysosomal 21kDa, V0 subunit b	NM_004047
	AKT2	v-akt murine thymoma viral oncogene homolog 2	NM_001626
	EIF4G1	eukaryotic translation initiation factor 4 gamma, 1	NM_182917
	GNB1	guanine nucleotide binding protein (G protein), beta polypeptide 1	NM_002074
	HSP90B1	heat shock protein 90kDa beta (Grp94), member 1	NM_003299
	RAPGEF1	Rap guanine nucleotide exchange factor (GEF) 1	NM_198679
	UCP2	uncoupling protein 2 (mitochondrial, proton carrier)	NM_003355
	ATP6V0D1	ATPase, H+ transporting, lysosomal 38kDa, V0 subunit d1	NM_004691
	STAT3	signal transducer and activator of transcription 3 (acute-phase response factor)	NM_213662
	HDAC5	histone deacetylase 5	NM_001015053
	SSH1	slingshot protein phosphatase 1	NM_001161331
	GNG8	guanine nucleotide binding protein (G protein), gamma 8	NM_033258
	SREBF1	sterol regulatory element binding transcription factor 1	NM_001005291
	UROS	uroporphyrinogen III synthase	NM_000375
	PRKAG3	protein kinase, AMP-activated, gamma 3 non-catalytic subunit	NM_017431
	SH3BP4	SH3-domain binding protein 4	NM_014521
	AKT1	v-akt murine thymoma viral oncogene homolog 1	NM_005163
	PDPK1	3-phosphoinositide dependent protein kinase 1	AF111845
esponse to hormones	ACSL1	acyl-CoA synthetase long-chain family member 1	NM_001995
	ATP6V0B	ATPase, H+ transporting, lysosomal 21kDa, V0 subunit b	NM_004047
	MYOG	myogenin (myogenic factor 4)	NM_002479
	AKT2	v-akt murine thymoma viral oncogene homolog 2	NM_001626
	EIF4G1	eukaryotic translation initiation factor 4 gamma, 1	NM_182917
	GNB1	guanine nucleotide binding protein (G protein), beta polypeptide 1	NM_002074
	RARG	retinoic acid receptor, gamma	NM_000966
	UCP2	uncoupling protein 2 (mitochondrial, proton carrier)	NM_003355

Cellular re	ATP6V0D1	ATPase, H+ transporting, lysosomal 38kDa, V0 subunit d1	NM_004691
	NR1H2	nuclear receptor subfamily 1, group H, member 2	NM_007121
	STAT3	signal transducer and activator of transcription 3 (acute-phase response factor)	NM_213662
	HDAC5	histone deacetylase 5	NM_001015053
	JUNB	jun B proto-oncogene	NM_002229
	GATA1	GATA binding protein 1 (globin transcription factor 1)	NM_002049
	GNG8	guanine nucleotide binding protein (G protein), gamma 8	NM_033258
	SREBF1	sterol regulatory element binding transcription factor 1	NM_001005291
	PRKAG3	protein kinase, AMP-activated, gamma 3 non-catalytic subunit	NM_017431
	AKT1	v-akt murine thymoma viral oncogene homolog 1	NM_005163
	PDPK1	3-phosphoinositide dependent protein kinase 1	AF111845
	OCSTAMP	osteoclast stimulatory transmembrane protein	NM_080721
Chemotaxis	CORO1A	coronin, actin binding protein, 1A	NM_007074
	ARHGEF16	Rho guanine nucleotide exchange factor (GEF) 16	NM_014448
	C5AR1	complement component 5a receptor 1	NM_001736
	FCER1G	Fc fragment of IgE, high affinity I, receptor for; gamma polypeptide	NM_004106
	SPTAN1	spectrin, alpha, non-erythrocytic 1	NM_003127
	POU4F2	POU class 4 homeobox 2	NM_004575
	AGRN	agrin	NM_198576
	DVL1	dishevelled segment polarity protein 1	NM_004421
	RPS6KA4	ribosomal protein S6 kinase, 90kDa, polypeptide 4	NM_003942
	CXCL16	chemokine (C-X-C motif) ligand 16	NM_022059
	ITGB2	integrin, beta 2 (complement component 3 receptor 3 and 4 subunit)	L78790
	MYH14	myosin, heavy chain 14, non-muscle	NM_001077186
	ROBO3	roundabout, axon guidance receptor, homolog 3 (Drosophila)	NM_022370
	MYO9B	myosin IXB	NM_004145
	AP2B1	adaptor-related protein complex 2, beta 1 subunit	NM_001030006
	COL6A2	collagen, type VI, alpha 2	NM_058174
	PLEKHG5	pleckstrin homology domain containing, family G (with RhoGef domain) member 5	NM_001265594
	RHOG	ras homolog family member G	NM_001665
	LAMB1	laminin, beta 1	
	CMTM7	CKLF-like MARVEL transmembrane domain containing 7	NM_138410
	KCNQ2	potassium channel, voltage gated KQT-like subfamily Q, member 2	NM_172109
	NFASC	neurofascin	NM_001005389
	DPYSL4	dihydropyrimidinase-like 4	NM_006426
Cellular response to insulin stimulus	ATP6V0B	ATPase, H+ transporting, lysosomal 21kDa, V0 subunit b	NM_004047
	AKT2	v-akt murine thymoma viral oncogene homolog 2	NM_001626
	EIF4G1	eukaryotic translation initiation factor 4 gamma, 1	NM_182917
	UCP2	uncoupling protein 2 (mitochondrial, proton carrier)	NM_003355
	ATP6V0D1	ATPase, H+ transporting, lysosomal 38kDa, V0 subunit d1	NM_004691
	HDAC5	histone deacetylase 5	NM_001015053
	SREBF1	sterol regulatory element binding transcription factor 1	NM_001005291
	PRKAG3	protein kinase, AMP-activated, gamma 3 non-catalytic subunit	NM_017431
	AKT1	v-akt murine thymoma viral oncogene homolog 1	NM_005163
	PDPK1	3-phosphoinositide dependent protein kinase 1	AF111845
Cellular response to chemical stimulus	WNT10A	wingless-type MMTV integration site family, member 10A	NM_025216
	CORO1A	coronin, actin binding protein, 1A	NM_007074
	ACSL1	acyl-CoA synthetase long-chain family member 1	NM_001995
	ARHGEF16	Rho guanine nucleotide exchange factor (GEF) 16	NM_014448
	PRDX5	peroxiredoxin 5	NM_012094
	DUSP4	dual specificity phosphatase 4	NM_001394
	CHI3L1	chitinase 3-like 1 (cartilage glycoprotein-39)	NM_001276
	ATP6V0B	ATPase, H+ transporting, lysosomal 21kDa, V0 subunit b	NM_004047
	CYP26A1	cytochrome P450, family 26, subfamily A, polypeptide 1	NM_057157
	GAPDH	glyceraldehyde-3-phosphate dehydrogenase	NM_002046
	IL2RG	interleukin 2 receptor, gamma	NM_000206
	C5AR1	complement component 5a receptor 1	NM_001736
	MYOG	myogenin (myogenic factor 4)	NM_002479
	FCER1G	Fc fragment of IgE, high affinity I, receptor for; gamma polypeptide	NM_004106
	CTGF	connective tissue growth factor	NM_001901

MKNK1	MAP kinase interacting serine/threonine kinase 1	NM_003684
MAPKAPK2	mitogen-activated protein kinase-activated protein kinase 2	NM_004759
GSTO2	glutathione S-transferase omega 2	NM_183239
OAS2	2'-5'-oligoadenylate synthetase 2, 69/71kDa	NM_016817
ACADVL	acyl-CoA dehydrogenase, very long chain	NM_000018
AKT2	v-akt murine thymoma viral oncogene homolog 2	NM_001626
ACSS2	acyl-CoA synthetase short-chain family member 2	NM_018677
EIF4G1	eukaryotic translation initiation factor 4 gamma, 1	NM_182917
GNB1	guanine nucleotide binding protein (G protein), beta polypeptide 1	NM_002074
HSP90B1	heat shock protein 90kDa beta (Grp94), member 1	NM_003299
RAPGEF1	Rap guanine nucleotide exchange factor (GEF) 1	NM_198679
RPS6KA4	ribosomal protein S6 kinase, 90kDa, polypeptide 4	NM_003942
MYD88	myeloid differentiation primary response 88	NM_002468
CACTIN	cactin, spliceosome C complex subunit	NM_021231
CXCL16	chemokine (C-X-C motif) ligand 16	NM_022059
RARG	retinoic acid receptor, gamma	NM_000966
ACER1	alkaline ceramidase 1	NM_133492
ITGB2	integrin, beta 2 (complement component 3 receptor 3 and 4 subunit)	L78790
CAMK2G	calcium/calmodulin-dependent protein kinase II gamma	NM_172171
UCP2	uncoupling protein 2 (mitochondrial, proton carrier)	NM_003355
PLK3	polo-like kinase 3	NM_004073
DGAT2	diacylglycerol O-acyltransferase 2	NM_032564
ATP6V0D1	ATPase, H+ transporting, lysosomal 38kDa, V0 subunit d1	NM_004691
NR1H2	nuclear receptor subfamily 1, group H, member 2	NM_007121
OAS1	2'-5'-oligoadenylate synthetase 1, 40/46kDa	NM_002534
UBE2L6	ubiquitin-conjugating enzyme E2L 6	NM_198183
SRPR	signal recognition particle receptor (docking protein)	NM_003139
IFITM3	interferon induced transmembrane protein 3	NM_021034
PHLPP1	PH domain and leucine rich repeat protein phosphatase 1	NM_194449
STAT3	signal transducer and activator of transcription 3 (acute-phase response factor)	NM_213662
HDAC5	histone deacetylase 5	NM_001015053
PRDX2	peroxiredoxin 2	NM_005809
JUNB	jun B proto-oncogene	NM_002229
MYO9B	myosin IXB	NM_004145
AP2B1	adaptor-related protein complex 2, beta 1 subunit	NM_001030006
SSH1	slingshot protein phosphatase 1	NM_001161331
HTRA2	HtrA serine peptidase 2	NM_145074
GATA1	GATA binding protein 1 (globin transcription factor 1)	NM_002049
IRF7	interferon regulatory factor 7	NM_004031
TPP1	tripeptidyl peptidase I	NM_000391
IL1R2	interleukin 1 receptor, type II	NM_004633
GNG8	guanine nucleotide binding protein (G protein), gamma 8	NM_033258
AIF1	allograft inflammatory factor 1	NM_004847
SREBF1	sterol regulatory element binding transcription factor 1	NM_001005291
CSF2RA	colony stimulating factor 2 receptor, alpha, low-affinity (granulocyte-macrophage)	
UROS	uroporphyrinogen III synthase	NM_000375
GPX1	glutathione peroxidase 1	NM_201397
PRKAG3	protein kinase, AMP-activated, gamma 3 non-catalytic subunit	NM_017431
SELPLG	selectin P ligand	NM_001206609
GSTA3	glutathione S-transferase alpha 3	NM_000847
CMA1	chymase 1, mast cell	NM_001836
IFNLR1	interferon, lambda receptor 1	NM_170743
PLEKHG5	pleckstrin homology domain containing, family G (with RhoGef domain) member 5	NM_001265594
SH3BP4	SH3-domain binding protein 4	NM_014521
AKT1	v-akt murine thymoma viral oncogene homolog 1	NM_005163
TNFRSF4	tumor necrosis factor receptor superfamily, member 4	NM_003327
MTRR	5-methyltetrahydrofolate-homocysteine methyltransferase reductase	NM_002454
NOS3	nitric oxide synthase 3 (endothelial cell)	NM_000603
LMNA	lamin A/C	NM_170707
RHOG	ras homolog family member G	NM_001665
HIF3A	hypoxia inducible factor 3, alpha subunit	XR_243952
PDPK1	3-phosphoinositide dependent protein kinase 1	AF111845
IFNAR2	interferon (alpha, beta and omega) receptor 2	NM_000874

ALDH3B1	aldehyde dehydrogenase 3 family, member B1	NM_001161473
LTK	leukocyte receptor tyrosine kinase	NM_002344
HLA-DRB3	major histocompatibility complex, class II, DR beta 3	NM_022555
TFDP2	transcription factor Dp-2 (E2F dimerization partner 2)	NM_001178138
PDE4A	phosphodiesterase 4A, cAMP-specific	NM_001111307
FKBP1A	FK506 binding protein 1A, 12kDa	NM_000801
OCSTAMP	osteoclast stimulatory transmembrane protein	NM_080721
MPV17	MpV17 mitochondrial inner membrane protein	NM_002437
RAE1	ribonucleic acid export 1	NM_001015885
RET	ret proto-oncogene	NM_020630
