# Supplementary material for: Transcriptional signature associated with early rheumatoid arthritis and healthy individuals at high risk to develop the disease
Source: PLoS One. 2018 Mar 27;13(3):e0194205. doi: 10.1371/journal.pone.0194205 (PMC5870959; doi:10.1371/journal.pone.0194205)
Supplement: S11 Table — (PDF) [file pone.0194205.s011.pdf]

Suplementary table 11. Biological function of the 2 fold change Up-regulated genes according GO analisis in RA

Biological function	Gene Symbol	Gene Name	Genebank Accession
Inflammatory response	KLKB1	kallikrein B, plasma (Fletcher factor) 1	NM_000892
	HRH4	histamine receptor H4	NM_021624
	ANO6	anoctamin 6	NM_001025356
	CCL11	chemokine (C-C motif) ligand 11	NM_002986
	ADORA1	adenosine A1 receptor	NM_000674
	ANXA1	annexin A1	NM_000700
	CLEC7A	C-type lectin domain family 7, member A	NM_197947
	VNN1	vanin 1	NM_004666
Cell projection organization	ACTR3	ARP3 actin-related protein 3 homolog (yeast)	NM_005721
	RB1	retinoblastoma 1	NM_000321
	BBS7	Bardet-Biedl syndrome 7	NM_018190
	ERBB3	v-erb-b2 erythroblastic leukemia viral oncogene homolog 3 (avian)	NM_001982
	BCL2	B-cell CLL/lymphoma 2	NM_000633
	DNM3	dynamin 3	NM_015569
	EGR2	early growth response 2	NM_000399
	SPTA1	spectrin, alpha, erythrocytic 1 (elliptocytosis 2)	NM_003126
	ROBO3	roundabout, axon guidance receptor, homolog 3 (Drosophila)	NM_022370
	SLITRK5	SLIT and NTRK-like family, member 5	NM_015567
	ETV4	ets variant 4	NM_001079675
	LHFPL5	lipoma HMGIC fusion partner-like 5	NM_182548
	NCK2	NCK adaptor protein 2	NM_003581
	WNT5A	wingless-type MMTV integration site family, member 5A	NM_003392
	MEF2A	myocyte enhancer factor 2A	NM_001171894
	CNTNAP2	contactin associated protein-like 2	NM_014141
	COL9A2	collagen, type IX, alpha 2	BC065715
	DNAAF1	dynein, axonemal, assembly factor 1	NM_178452
	EPHB2	EPH receptor B2	NM_004442
	FZD6	frizzled family receptor 6	NM_003506
Signal transduction	CXCL12	chemokine (C-X-C motif) ligand 12	NM_001033886
	ATP6V1E1	ATPase, H+ transporting, lysosomal 31kDa, V1 subunit E1	NM_001696
	AGTR1	angiotensin II receptor, type 1	NM_031850
	SHOC2	soc-2 suppressor of clear homolog (C. elegans)	NM_007373
	RB1	retinoblastoma 1	NM_000321
	TRAT1	T cell receptor associated transmembrane adaptor 1	NM_016388
	FGF1	fibroblast growth factor 1 (acidic)	NM_000800
	ATP6V0E1	ATPase, H+ transporting, lysosomal 9kDa, V0 subunit e1	NM_003945
	EGR1	early growth response 1	NM_001964
	WSB2	WD repeat and SOCS box containing 2	NM_018639
	LPAR6	lysophosphatidic acid receptor 6	NM_005767
	CTNNB1	catenin (cadherin-associated protein), beta 1, 88kDa	NM_001904
	STC1	stanniocalcin 1	NM_003155
	INADL	InaD-like (Drosophila)	NM_176877
	HCAR2	hydroxycarboxylic acid receptor 2	NM_177551
	TAS2R50	taste receptor, type 2, member 50	NM_176890
	ERBB3	v-erb-b2 erythroblastic leukemia viral oncogene homolog 3 (avian)	NM_001982
	BCL2	B-cell CLL/lymphoma 2	NM_000633
	RGS12	regulator of G-protein signaling 12	NM_002926
	AHRR	aryl-hydrocarbon receptor repressor	NM_020731
	ITPKB	inositol-trisphosphate 3-kinase B	NM_002221
	HRH4	histamine receptor H4	NM_021624
	RAB2B	RAB2B, member RAS oncogene family	NM_032846
	DACT1	dapper, antagonist of beta-catenin, homolog 1 (Xenopus laevis)	NM_016651
	CCL11	chemokine (C-C motif) ligand 11	NM_002986

NPPA	natriuretic peptide A	NM_006172
ADORA1	adenosine A1 receptor	NM_000674
EVI2A	ecotropic viral integration site 2A	NM_001003927
PLCL2	phospholipase C-like 2	NM_015184
LRP12	low density lipoprotein receptor-related protein 12	NM_013437
CNTNAP3	contactin associated protein-like 3	NM_033655
ANXA1	annexin A1	NM_000700
UBE3A	ubiquitin protein ligase E3A	NM_130839
CLEC7A	C-type lectin domain family 7, member A	NM_197947
TANK	TRAF family member-associated NFKB activator	NM_133484
NDFIP2	Nedd4 family interacting protein 2	NM_019080
PIK3R1	phosphoinositide-3-kinase, regulatory subunit 1 (alpha)	NM_181523
RIN2	Ras and Rab interactor 2	NM_018993
XPR1	xenotropic and polytropic retrovirus receptor 1	NM_004736
CX3CL1	chemokine (C-X3-C motif) ligand 1	NM_002996
RAB39B	RAB39B, member RAS oncogene family	NM_171998
ARHGAP28	Rho GTPase activating protein 28	NM_001010000
STK17B	serine/threonine kinase 17b	NM_004226
BMPR2	bone morphogenetic protein receptor, type II (serine/threonine kinase)	NM_001204
WNK1	WNK lysine deficient protein kinase 1	NM_001184985
PTEN	phosphatase and tensin homolog	NM_000314
ASB7	ankyrin repeat and SOCS box containing 7	NM_198243
TGM2	transglutaminase 2 (C polypeptide, protein-glutamine-gamma-glutamyltransferase)	NM_198951
NR1D2	nuclear receptor subfamily 1, group D, member 2	NM_005126
HSPA5	heat shock 70kDa protein 5 (glucose-regulated protein, 78kDa)	NM_005347
RCAN2	regulator of calcineurin 2	NM_005822
ARHGAP18	Rho GTPase activating protein 18	NM_033515
ARID5B	AT rich interactive domain 5B (MRF1-like)	NM_032199
RGSL1	regulator of G-protein signaling like 1	NM_001137669
CLEC2D	C-type lectin domain family 2, member D	NM_001004419
PTPRK	protein tyrosine phosphatase, receptor type, K	NM_002844
TXNDC17	thioredoxin domain containing 17	NM_032731
NR3C1	nuclear receptor subfamily 3, group C, member 1 (glucocorticoid receptor)	NM_001018077
EPHB4	EPH receptor B4	NM_004444
INSIG1	insulin induced gene 1	NM_005542
GPR78	G protein-coupled receptor 78	NM_080819
LIMD1	LIM domains containing 1	NM_014240
ARHGAP40	Rho GTPase activating protein 40	NM_001164431
CD69	CD69 molecule	AK303383
PRKAA1	protein kinase, AMP-activated, alpha 1 catalytic subunit	NM_206907
NCK2	NCK adaptor protein 2	NM_003581
NCS1	neuronal calcium sensor 1	NM_014286
ATF1	activating transcription factor 1	NM_005171
GCG	glucagon	NM_002054
NR2C1	nuclear receptor subfamily 2, group C, member 1	NM_001032287
PTPRJ	protein tyrosine phosphatase, receptor type, J	NM_001098503
SUMO1	SMT3 suppressor of mif two 3 homolog 1 (S. cerevisiae)	NM_003352
WNT5A	wingless-type MMTV integration site family, member 5A	NM_003392
MEF2A	myocyte enhancer factor 2A	NM_001171894
GPX1	glutathione peroxidase 1	NM_201397
OR5AN1	olfactory receptor, family 5, subfamily AN, member 1	NM_001004729
STAP1	signal transducing adaptor family member 1	NM_012108
CNTNAP2	contactin associated protein-like 2	NM_014141
EPHB2	EPH receptor B2	NM_004442
STK17B	serine/threonine kinase 17b	BC052561
SKAP2	src kinase associated phosphoprotein 2	NM_003930
WNT4	wingless-type MMTV integration site family, member 4	NM_030761
DIXDC1	DIX domain containing 1	NM_001037954

	FZD6	frizzled family receptor 6	NM_003506
	MRGPRX2	MAS-related GPR, member X2	NM_054030
	LRRD1	leucine-rich repeats and death domain containing 1	NM_001161528
	OR4D1	olfactory receptor, family 4, subfamily D, member 1	NM_012374
	OR51E1	olfactory receptor, family 51, subfamily E, member 1	NM_152430
**Cellular response to chemical stimulus**	CXCL12	chemokine (C-X-C motif) ligand 12	NM_001033886
	NR3C1	nuclear receptor subfamily 3, group C, member 1 (glucocorticoid receptor)	NM_001018077
	CYP39A1	cytochrome P450, family 39, subfamily A, polypeptide 1	NM_016593
	CLIC4	chloride intracellular channel 4	NM_013943
	ATP6V1E1	ATPase, H+ transporting, lysosomal 31kDa, V1 subunit E1	NM_001696
	AGTR1	angiotensin II receptor, type 1	NM_031850
	SHOC2	soc-2 suppressor of clear homolog (C. elegans)	NM_007373
	FGF1	fibroblast growth factor 1 (acidic)	NM_000800
	ATP6V0E1	ATPase, H+ transporting, lysosomal 9kDa, V0 subunit e1	NM_003945
	EGR1	early growth response 1	NM_001964
	SNCA	synuclein, alpha (non A4 component of amyloid precursor)	NM_007308
	STC1	stanniocalcin 1	NM_003155
	ERBB3	v-erb-b2 erythroblastic leukemia viral oncogene homolog 3 (avian)	NM_001982
	PRDX3	peroxiredoxin 3	NM_006793
	CCL11	chemokine (C-C motif) ligand 11	NM_002986
	FABP4	fatty acid binding protein 4, adipocyte	NM_001442
	ANXA1	annexin A1	NM_000700
	CLEC7A	C-type lectin domain family 7, member A	NM_197947
	PIK3R1	phosphoinositide-3-kinase, regulatory subunit 1 (alpha)	NM_181523
	SERPINB9	serpin peptidase inhibitor, clade B (ovalbumin), member 9	NM_004155
	CYP2B6	cytochrome P450, family 2, subfamily B, polypeptide 6	NM_000767
	CX3CL1	chemokine (C-X3-C motif) ligand 1	NM_002996
	HMGCS1	3-hydroxy-3-methylglutaryl-CoA synthase 1 (soluble)	NM_002130
	ETS1	v-ets erythroblastosis virus E26 oncogene homolog 1 (avian)	NM_005238
	PTEN	phosphatase and tensin homolog	NM_000314
	NR1D2	nuclear receptor subfamily 1, group D, member 2	NM_005126
	HSPA5	heat shock 70kDa protein 5 (glucose-regulated protein, 78kDa)	NM_005347
	PTPRK	protein tyrosine phosphatase, receptor type, K	NM_002844
	TXNDC17	thioredoxin domain containing 17	NM_032731
	NR3C1	nuclear receptor subfamily 3, group C, member 1 (glucocorticoid receptor)	NM_001018077
	CD69	CD69 molecule	AK303383
	PRKAA1	protein kinase, AMP-activated, alpha 1 catalytic subunit	NM_206907
	ATF1	activating transcription factor 1	NM_005171
	GCG	glucagon	NM_002054
	NR2C1	nuclear receptor subfamily 2, group C, member 1	NM_001032287
	SUMO1	SMT3 suppressor of mif two 3 homolog 1 (S. cerevisiae)	NM_003352
	MEF2A	myocyte enhancer factor 2A	NM_001171894
	RBM3	RNA binding motif (RNP1, RRM) protein 3	NM_006743
	GPX1	glutathione peroxidase 1	NM_201397
	UGDH	UDP-glucose 6-dehydrogenase	NM_003359
	WNT4	wingless-type MMTV integration site family, member 4	NM_030761
**Defence response**	CXCL12	chemokine (C-X-C motif) ligand 12	NM_001033886
	NR3C1	nuclear receptor subfamily 3, group C, member 1 (glucocorticoid receptor)	NM_001018077
	BNIP3L	BCL2/adenovirus E1B 19kDa interacting protein 3-like	NM_004331
	HP	haptoglobin	NM_005143
	TRAT1	T cell receptor associated transmembrane adaptor 1	NM_016388
	EGR1	early growth response 1	NM_001964
	C1S	complement component 1, s subcomponent	NM_001734
	KLKB1	kallikrein B, plasma (Fletcher factor) 1	NM_000892
	SNCA	synuclein, alpha (non A4 component of amyloid precursor)	NM_007308
	DEFA4	defensin, alpha 4, corticostatin	NM_001925

	BCL2	B-cell CLL/lymphoma 2	NM_000633
	HRH4	histamine receptor H4	NM_021624
	ANO6	anoctamin 6	NM_001025356
	TFF3	trefoil factor 3 (intestinal)	NM_003226
	DEFB126	defensin, beta 126	NM_030931
	CCL11	chemokine (C-C motif) ligand 11	NM_002986
	ADORA1	adenosine A1 receptor	NM_000674
	ANXA1	annexin A1	NM_000700
	CLEC7A	C-type lectin domain family 7, member A	NM_197947
	TANK	TRAF family member-associated NFKB activator	NM_133484
	CX3CL1	chemokine (C-X3-C motif) ligand 1	NM_002996
	ATF1	activating transcription factor 1	NM_005171
	SUMO1	SMT3 suppressor of mif two 3 homolog 1 (S. cerevisiae)	NM_003352
	MEF2A	myocyte enhancer factor 2A	NM_001171894
	HTN1	histatin 1	NM_002159
	KRT1	keratin 1	NM_006121
	BNIP3L	BCL2/adenovirus E1B 19kDa interacting protein 3-like	NM_004331
**Immune response**	VNN1	vanin 1	NM_004666
	SERPING1	serpin peptidase inhibitor, clade G (C1 inhibitor), member 1	
	EGR1	early growth response 1	NM_001964
	C1S	complement component 1, s subcomponent	NM_001734
	SNCA	synuclein, alpha (non A4 component of amyloid precursor)	NM_007308
	POU2AF1	POU class 2 associating factor 1	NM_006235
	BCL2	B-cell CLL/lymphoma 2	NM_000633
	CCL11	chemokine (C-C motif) ligand 11	NM_002986
	CLEC7A	C-type lectin domain family 7, member A	NM_197947
	TANK	TRAF family member-associated NFKB activator	NM_133484
	SERPINB9	serpin peptidase inhibitor, clade B (ovalbumin), member 9	NM_004155
	CX3CL1	chemokine (C-X3-C motif) ligand 1	NM_002996
	ETS1	v-ets erythroblastosis virus E26 oncogene homolog 1 (avian)	NM_005238
	ATF1	activating transcription factor 1	NM_005171
	SUMO1	SMT3 suppressor of mif two 3 homolog 1 (S. cerevisiae)	NM_003352
	MEF2A	myocyte enhancer factor 2A	NM_001171894
	KRT1	keratin 1	NM_006121
	VNN1	vanin 1	NM_004666
	MS4A1	membrane-spanning 4-domains, subfamily A, member 1	NM_152866
	SERPING1	serpin peptidase inhibitor, clade G (C1 inhibitor), member 1	
	CXCL12	chemokine (C-X-C motif) ligand 12	NM_001033886
**Regulation of metabolic process**	USP1	ubiquitin specific peptidase 1	NM_003368
	TCEA1	transcription elongation factor A (SII), 1	NM_006756
	AKAP6	A kinase (PRKA) anchor protein 6	NM_004274
	FEM1B	fem-1 homolog b (C. elegans)	NM_015322
	TAF1B	TATA box binding protein (TBP)-associated factor, RNA polymerase I, B, 63kDa	NM_005680
	AGTR1	angiotensin II receptor, type 1	NM_031850
	SHOC2	soc-2 suppressor of clear homolog (C. elegans)	NM_007373
	RB1	retinoblastoma 1	NM_000321
	HP	haptoglobin	NM_005143
	ZNF217	zinc finger protein 217	NM_006526
	TRAT1	T cell receptor associated transmembrane adaptor 1	NM_016388
	HNRPDL	heterogeneous nuclear ribonucleoprotein D-like	NM_031372
	FGF1	fibroblast growth factor 1 (acidic)	NM_000800
	CAST	calpastatin	NM_001042440
	EGR1	early growth response 1	NM_001964
	KLKB1	kallikrein B, plasma (Fletcher factor) 1	NM_000892
	CTNNB1	catenin (cadherin-associated protein), beta 1, 88kDa	NM_001904
	SNCA	synuclein, alpha (non A4 component of amyloid precursor)	NM_007308

L3MBTL3	l(3)mbt-like 3 (Drosophila)	NM_032438
POU2AF1	POU class 2 associating factor 1	NM_006235
MORC3	MORC family CW-type zinc finger 3	NM_015358
HCAR2	hydroxycarboxylic acid receptor 2	NM_177551
C17orf42	chromosome 17 open reading frame 42	NM_024683
ERBB3	v-erb-b2 erythroblastic leukemia viral oncogene homolog 3 (avian)	NM_001982
BCL2	B-cell CLL/lymphoma 2	NM_000633
RGS12	regulator of G-protein signaling 12	NM_002926
AHRR	aryl-hydrocarbon receptor repressor	NM_020731
ZNF664	zinc finger protein 664	NM_152437
ACER1	alkaline ceramidase 1	NM_133492
E2F2	E2F transcription factor 2	NM_004091
TBC1D9	TBC1 domain family, member 9 (with GRAM domain)	NM_015130
SBF2	SET binding factor 2	NM_030962
ZFHX4	zinc finger homeobox 4	NM_024721
EGR2	early growth response 2	NM_000399
MKL2	MKL/myocardin-like 2	NM_014048
PRDX3	peroxiredoxin 3	NM_006793
CCL11	chemokine (C-C motif) ligand 11	NM_002986
MOSPD1	motile sperm domain containing 1	NM_019556
ADORA1	adenosine A1 receptor	NM_000674
ZBTB6	zinc finger and BTB domain containing 6	NM_006626
SOX13	SRY (sex determining region Y)-box 13	NM_005686
YEATS4	YEATS domain containing 4	NM_006530
FABP4	fatty acid binding protein 4, adipocyte	NM_001442
ZNF334	zinc finger protein 334	NM_199441
TSC22D2	TSC22 domain family, member 2	NM_014779
ANXA1	annexin A1	NM_000700
WFDC2	WAP four-disulfide core domain 2	NM_006103
PBX1	pre-B-cell leukemia homeobox 1	NM_002585
UBE3A	ubiquitin protein ligase E3A	NM_130839
ANP32E	acidic (leucine-rich) nuclear phosphoprotein 32 family, member E	NM_030920
NDFIP2	Nedd4 family interacting protein 2	NM_019080
MEAF6	MYST/Esa1-associated factor 6	NM_022756
PIK3R1	phosphoinositide-3-kinase, regulatory subunit 1 (alpha)	NM_181523
SERPINB9	serpin peptidase inhibitor, clade B (ovalbumin), member 9	NM_004155
RIN2	Ras and Rab interactor 2	NM_018993
HNRNPH1	heterogeneous nuclear ribonucleoprotein H1 (H)	NM_005520
KNDC1	kinase non-catalytic C-lobe domain (KIND) containing 1	NM_152643
FLI1	Friend leukemia virus integration 1	NM_002017
PPP1CB	protein phosphatase 1, catalytic subunit, beta isozyme	NM_002709
EIF5	eukaryotic translation initiation factor 5	NM_001969
ETV4	ets variant 4	NM_001079675
IRF2BP2	interferon regulatory factor 2 binding protein 2	NM_182972
ARHGAP28	Rho GTPase activating protein 28	NM_001010000
ETS1	v-ets erythroblastosis virus E26 oncogene homolog 1 (avian)	NM_005238
BMPR2	bone morphogenetic protein receptor, type II (serine/threonine kinase)	NM_001204
WNK1	WNK lysine deficient protein kinase 1	NM_001184985
PTEN	phosphatase and tensin homolog	NM_000314
NR1D2	nuclear receptor subfamily 1, group D, member 2	NM_005126
HSPA5	heat shock 70kDa protein 5 (glucose-regulated protein, 78kDa)	NM_005347
ARHGAP18	Rho GTPase activating protein 18	NM_033515
ZNF367	zinc finger protein 367	NM_153695
ARID5B	AT rich interactive domain 5B (MRF1-like)	NM_032199
TAF1	TAF1 RNA polymerase II, TATA box binding protein (TBP)-associated factor, 250kDa	NM_004606
CDC5L	CDC5 cell division cycle 5-like (S. pombe)	NM_001253
SCAI	suppressor of cancer cell invasion	NM_173690
ZIC1	Zic family member 1	NM_003412

	PTPRK	protein tyrosine phosphatase, receptor type, K	NM_002844
	ZNF673	zinc finger family member 673	AK097159
	CLK1	CDC-like kinase 1	NM_001162407
	TFPI	tissue factor pathway inhibitor (lipoprotein-associated coagulation inhibitor)	NM_006287
	ANKRD62	ankyrin repeat domain 62	XM_003118756
	NR3C1	nuclear receptor subfamily 3, group C, member 1 (glucocorticoid receptor)	NM_001018077
	JMY	junction mediating and regulatory protein, p53 cofactor	NM_152405
	INSIG1	insulin induced gene 1	NM_005542
	LIMD1	LIM domains containing 1	NM_014240
	ARHGAP40	Rho GTPase activating protein 40	NM_001164431
	CHTOP	chromatin target of PRMT1	AK097870
	SETD7	SET domain containing (lysine methyltransferase) 7	BC066361
	PRKAA1	protein kinase, AMP-activated, alpha 1 catalytic subunit	NM_206907
	NCBP1	nuclear cap binding protein subunit 1, 80kDa	
	NCK2	NCK adaptor protein 2	NM_003581
	ATF1	activating transcription factor 1	NM_005171
	GCG	glucagon	NM_002054
	NR2C1	nuclear receptor subfamily 2, group C, member 1	NM_001032287
	PTPRJ	protein tyrosine phosphatase, receptor type, J	NM_001098503
	SUMO1	SMT3 suppressor of mif two 3 homolog 1 (S. cerevisiae)	NM_003352
	POLR2F	polymerase (RNA) II (DNA directed) polypeptide F	
	MAMSTR	MEF2 activating motif and SAP domain containing transcriptional regulator	NM_182574
	CBX1	chromobox homolog 1	NM_006807
	WNT5A	wingless-type MMTV integration site family, member 5A	NM_003392
	MEF2A	myocyte enhancer factor 2A	NM_001171894
	SP4	Sp4 transcription factor	NM_003112
	ARGLU1	arginine and glutamate rich 1	AK304751
	RBM3	RNA binding motif (RNP1, RRM) protein 3	NM_006743
	GPX1	glutathione peroxidase 1	NM_201397
	TAF4B	TAF4b RNA polymerase II, TATA box binding protein (TBP)-associated factor, 105kDa	NM_005640
	RASGEF1B	RasGEF domain family, member 1B	NM_152545
	LCN1	lipocalin 1	NM_002297
	UHRF1	ubiquitin-like with PHD and ring finger domains 1	NM_013282
	WNT4	wingless-type MMTV integration site family, member 4	NM_030761
	DIXDC1	DIX domain containing 1	NM_001037954
	HLF	hepatic leukemia factor	NM_002126
	EIF2S1	eukaryotic translation initiation factor 2, subunit 1 alpha, 35kDa	NM_004094
	TMF1	TATA element modulatory factor 1	NM_007114
	ATXN1	ataxin 1	NM_000332
	FZD6	frizzled family receptor 6	NM_003506
	SERPING1	serpin peptidase inhibitor, clade G (C1 inhibitor), member 1	
	ZNF235	zinc finger protein 235	NM_004234
	SUV420H1	suppressor of variegation 4-20 homolog 1 (Drosophila)	NM_017635
	ZBTB8A	zinc finger and BTB domain containing 8A	NM_001040441
	A2ML1	alpha-2-macroglobulin-like 1	
	NR3C1	nuclear receptor subfamily 3, group C, member 1 (glucocorticoid receptor)	NM_144670<br>NM_001018077
**lation of immune system process**	TRAT1	T cell receptor associated transmembrane adaptor 1	NM_016388
	C1S	complement component 1, s subcomponent	NM_001734
	BCL2	B-cell CLL/lymphoma 2	NM_000633
	ITPKB	inositol-trisphosphate 3-kinase B	NM_002221
	CLEC7A	C-type lectin domain family 7, member A	NM_197947
	PIK3R1	phosphoinositide-3-kinase, regulatory subunit 1 (alpha)	NM_181523
	CX3CL1	chemokine (C-X3-C motif) ligand 1	NM_002996
	PVR	poliovirus receptor	NM_006505
	PTEN	phosphatase and tensin homolog	NM_000314
	NCK2	NCK adaptor protein 2	NM_003581
	ATF1	activating transcription factor 1	NM_005171

Positive regu	WNT5A	wingless-type MMTV integration site family, member 5A	NM_003392
	MEF2A	myocyte enhancer factor 2A	NM_001171894
	KRT1	keratin 1	NM_006121
	VNN1	vanin 1	NM_004666
	SERPING1	serpin peptidase inhibitor, clade G (C1 inhibitor), member 1	
	CXCL12	chemokine (C-X-C motif) ligand 12	NM_001033886
Cell surface receptor signalling pathway	ATP6V1E1	ATPase, H+ transporting, lysosomal 31kDa, V1 subunit E1	NM_001696
	AGTR1	angiotensin II receptor, type 1	NM_031850
	SHOC2	soc-2 suppressor of clear homolog (C. elegans)	NM_007373
	TRAT1	T cell receptor associated transmembrane adaptor 1	NM_016388
	FGF1	fibroblast growth factor 1 (acidic)	NM_000800
	ATP6V0E1	ATPase, H+ transporting, lysosomal 9kDa, V0 subunit e1	NM_003945
	EGR1	early growth response 1	NM_001964
	LPAR6	lysophosphatidic acid receptor 6	NM_005767
	CTNNB1	catenin (cadherin-associated protein), beta 1, 88kDa	NM_001904
	STC1	stanniocalcin 1	NM_003155
	HCAR2	hydroxycarboxylic acid receptor 2	NM_177551
	TAS2R50	taste receptor, type 2, member 50	NM_176890
	ERBB3	erb-b2 receptor tyrosine kinase 3	NM_001982
	ITPKB	inositol-trisphosphate 3-kinase B	NM_002221
	HRH4	histamine receptor H4	NM_021624
	DACT1	dishevelled-binding antagonist of beta-catenin 1	NM_016651
	NPPA	natriuretic peptide A	NM_006172
	ADORA1	adenosine A1 receptor	NM_000674
	ANXA1	annexin A1	NM_000700
	PIK3R1	phosphoinositide-3-kinase, regulatory subunit 1 (alpha)	NM_181523
	XPR1	xenotropic and polytropic retrovirus receptor 1	NM_004736
	CX3CL1	chemokine (C-X3-C motif) ligand 1	AB209037
	BMPR2	bone morphogenetic protein receptor, type II (serine/threonine kinase)	NM_001204
	PTEN	phosphatase and tensin homolog	NM_000314
	TGM2	transglutaminase 2	NM_198951
	ARID5B	AT rich interactive domain 5B (MRF1-like)	NM_032199
	CLEC2D	C-type lectin domain family 2, member D	NM_001004419
	PTPRK	protein tyrosine phosphatase, receptor type, K	NM_002844
	TXNDC17	thioredoxin domain containing 17	NM_032731
	EPHB4	EPH receptor B4	NM_004444
	GPR78	G protein-coupled receptor 78	NM_080819
	PRKAA1	protein kinase, AMP-activated, alpha 1 catalytic subunit	NM_206907
	NCK2	NCK adaptor protein 2	NM_003581
	ATF1	activating transcription factor 1	NM_005171
	GCG	glucagon	NM_002054
	PTPRJ	protein tyrosine phosphatase, receptor type, J	NM_001098503
	SUMO1	small ubiquitin-like modifier 1	NM_003352
	WNT5A	wingless-type MMTV integration site family, member 5A	NM_003392
	MEF2A	myocyte enhancer factor 2A	NM_001171894
	OR5AN1	olfactory receptor, family 5, subfamily AN, member 1	NM_001004729
	STAP1	signal transducing adaptor family member 1	NM_012108
	EPHB2	EPH receptor B2	NM_004442
	WNT4	wingless-type MMTV integration site family, member 4	NM_030761
	DIXDC1	DIX domain containing 1	NM_001037954
	FZD6	frizzled class receptor 6	NM_003506
	MRGPRX2	MAS-related GPR, member X2	BC063450
	OR4D1	olfactory receptor, family 4, subfamily D, member 1	NM_012374
	OR51E1	olfactory receptor, family 51, subfamily E, member 1	NM_152430
	CXCL12	chemokine (C-X-C motif) ligand 12	NM_001033886
ess	USP1	ubiquitin specific peptidase 1	NM_003368

Cellular protein modification proc	SRPK3	SRSF protein kinase 3	NM_014370
	MSL2	male-specific lethal 2 homolog (Drosophila)	NM_018133
	CBLL1	Cbl proto-oncogene-like 1, E3 ubiquitin protein ligase	NM_024814
	TRIP12	thyroid hormone receptor interactor 12	NM_004238
	FEM1B	fem-1 homolog b (C. elegans)	NM_015322
	BRD1	bromodomain containing 1	NM_014577
	PGM3	phosphoglucomutase 3	NM_015599
	FGF1	fibroblast growth factor 1 (acidic)	NM_000800
	HACE1	HECT domain and ankyrin repeat containing E3 ubiquitin protein ligase 1	NM_020771
	MORC3	MORC family CW-type zinc finger 3	NM_015358
	ERBB3	erb-b2 receptor tyrosine kinase 3	NM_001982
	BCL2	B-cell CLL/lymphoma 2	NM_000633
	PPP6C	protein phosphatase 6, catalytic subunit	NM_002721
	TGM4	transglutaminase 4	NM_003241
	CCL11	chemokine (C-C motif) ligand 11	NM_002986
	YEATS4	YEATS domain containing 4	NM_006530
	ANXA1	annexin A1	NM_000700
	METAP2	methionyl aminopeptidase 2	NM_006838
	UBE3A	ubiquitin protein ligase E3A	NM_130839
	MEAF6	MYST/Esa1-associated factor 6	NM_022756
	KNDC1	kinase non-catalytic C-lobe domain (KIND) containing 1	NM_152643
	PPP1CB	protein phosphatase 1, catalytic subunit, beta isozyme	NM_002709
	PGGT1B	protein geranylgeranyltransferase type I, beta subunit	NM_005023
	STK17B	serine/threonine kinase 17b	NM_004226
	BMPR2	bone morphogenetic protein receptor, type II (serine/threonine kinase)	NM_001204
	WNK1	WNK lysine deficient protein kinase 1	NM_001184985
	PTEN	phosphatase and tensin homolog	NM_000314
	TGM2	transglutaminase 2	NM_198951
	TAF1	TAF1 RNA polymerase II, TATA box binding protein (TBP)-associated factor, 250kDa	NM_001286074
	RSRC1	arginine/serine-rich coiled-coil 1	NM_016625
	PTPRK	protein tyrosine phosphatase, receptor type, K	NM_002844
	CLK1	CDC-like kinase 1	NM_001162407
	EPHB4	EPH receptor B4	NM_004444
	DNAJC6	DnaJ (Hsp40) homolog, subfamily C, member 6	NM_001256864
	ANAPC16	anaphase promoting complex subunit 16	NM_001242546
	FBXO24	F-box protein 24	NM_033506
	SETD7	SET domain containing (lysine methyltransferase) 7	BC066361
	PRKAA1	protein kinase, AMP-activated, alpha 1 catalytic subunit	NM_206907
	ZDHHC2	zinc finger, DHHC-type containing 2	NM_016353
	GCNT1	glucosaminyl (N-acetyl) transferase 1, core 2	NM_001097634
	DCAF16	DDB1 and CUL4 associated factor 16	NM_017741
	PTPRJ	protein tyrosine phosphatase, receptor type, J	NM_001098503
	SUMO1	small ubiquitin-like modifier 1	NM_003352
	lnc-POLR2F-1	lnc-POLR2F-1:1	
	TRIOBP	TRIO and F-actin binding protein	NM_138632
	BMP2K	BMP2 inducible kinase	NM_017593
	MYO3A	myosin IIIA	BC036079
	GPX1	glutathione peroxidase 1	NM_201397
	EPHB2	EPH receptor B2	NM_004442
	STK17B	serine/threonine kinase 17b	BC052561
	SENP6	SUMO1/sentrin specific peptidase 6	NM_015571
	WNT4	wingless-type MMTV integration site family, member 4	NM_030761
	EIF2S1	eukaryotic translation initiation factor 2, subunit 1 alpha, 35kDa	NM_004094
	F13A1	coagulation factor XIII, A1 polypeptide	NM_000129
	SUV420H1	suppressor of variegation 4-20 homolog 1 (Drosophila)	NM_001300907
›ment	BCL2	B-cell CLL/lymphoma 2	NM_000633
	EGR2	early growth response 2	NM_000399

**Brain develop**	DCT	dopachrome tautomerase	NM_001922
	OTP	orthopedia homeobox	NM_032109
	TWSG1	twisted gastrulation BMP signaling modulator 1	NM_020648
	UBE3A	ubiquitin protein ligase E3A	NM_130839
	KNDC1	kinase non-catalytic C-lobe domain (KIND) containing 1	NM_152643
	HMGCS1	3-hydroxy-3-methylglutaryl-CoA synthase 1 (soluble)	NM_002130
	ETS1	v-ets avian erythroblastosis virus E26 oncogene homolog 1	NM_005238
	BMPR2	bone morphogenetic protein receptor, type II (serine/threonine kinase)	NM_001204
	HSPA5	heat shock 70kDa protein 5 (glucose-regulated protein, 78kDa)	NM_005347
	ZIC1	Zic family member 1	NM_003412
	NR3C1	nuclear receptor subfamily 3, group C, member 1 (glucocorticoid receptor)	NM_001018077
	WNT5A	wingless-type MMTV integration site family, member 5A	NM_003392
	CNTNAP2	contactin associated protein-like 2	NM_014141
	EPHB2	EPH receptor B2	NM_004442
	WNT4	wingless-type MMTV integration site family, member 4	NM_030761
	DIXDC1	DIX domain containing 1	NM_001037954
	FZD6	frizzled class receptor 6	NM_003506
	CXCL12	chemokine (C-X-C motif) ligand 12	NM_001033886
	NR3C1	nuclear receptor subfamily 3, group C, member 1 (glucocorticoid receptor)	NM_001018077
**Cellular nitrogen compound metabolic process**	USP1	ubiquitin specific peptidase 1	NM_003368
	VNN2	vanin 2	NM_004665
	RPIA	ribose 5-phosphate isomerase A	NM_144563
	TCEA1	transcription elongation factor A (SII), 1	NM_006756
	ATP6V1E1	ATPase, H+ transporting, lysosomal 31kDa, V1 subunit E1	NM_001696
	DDX24	DEAD (Asp-Glu-Ala-Asp) box helicase 24	NM_020414
	SSB	Sjogren syndrome antigen B (autoantigen La)	NM_003142
	TAF1B	TATA box binding protein (TBP)-associated factor, RNA polymerase I, B, 63kDa	NM_005680
	PGM3	phosphoglucomutase 3	NM_015599
	ZNF217	zinc finger protein 217	NM_006526
	HNRNPDL	heterogeneous nuclear ribonucleoprotein D-like	NM_031372
	ATP6V0E1	ATPase, H+ transporting, lysosomal 9kDa, V0 subunit e1	NM_003945
	EGR1	early growth response 1	NM_001964
	POU2AF1	POU class 2 associating factor 1	NM_006235
	TEFM	transcription elongation factor, mitochondrial	NM_024683
	AK9	adenylate kinase 9	NM_145025
	DNM3	dynamin 3	NM_015569
	ABCC13	ATP-binding cassette, sub-family C (CFTR/MRP), member 13, pseudogene	NR_003088
	E2F2	E2F transcription factor 2	NM_004091
	EGR2	early growth response 2	NM_000399
	NAP1L1	nucleosome assembly protein 1-like 1	NM_139207
	SPTA1	spectrin, alpha, erythrocytic 1	NM_003126
	TRUB1	TruB pseudouridine (psi) synthase family member 1	NM_139169
	NPPA	natriuretic peptide A	NM_006172
	TSPYL6	TSPY-like 6	NM_001003937
	NT5C1A	5'-nucleotidase, cytosolic IA	NM_032526
	MYH7	myosin, heavy chain 7, cardiac muscle, beta	NM_000257
	HNRNPH1	heterogeneous nuclear ribonucleoprotein H1 (H)	NM_005520
	HNRNPH3	heterogeneous nuclear ribonucleoprotein H3 (2H9)	NM_012207
	AMD1	adenosylmethionine decarboxylase 1	NM_001634
	HNRNPA2B1	heterogeneous nuclear ribonucleoprotein A2/B1	NM_002137
	EIF5	eukaryotic translation initiation factor 5	NM_001969
	QRSL1	glutaminyl-tRNA synthase (glutamine-hydrolyzing)-like 1	NM_018292
	ETS1	v-ets avian erythroblastosis virus E26 oncogene homolog 1	NM_005238
	BMPR2	bone morphogenetic protein receptor, type II (serine/threonine kinase)	NM_001204
	SRSF1	serine/arginine-rich splicing factor 1	NM_001078166
	BCAT1	branched chain amino-acid transaminase 1, cytosolic	NM_005504
	NR1D2	nuclear receptor subfamily 1, group D, member 2	NM_005126

HSPA5	heat shock 70kDa protein 5 (glucose-regulated protein, 78kDa)	NM_005347
SRSF8	serine/arginine-rich splicing factor 8	NM_032102
TAF1	TAF1 RNA polymerase II, TATA box binding protein (TBP)-associated factor, 250kDa	NM_001286074
RSRC1	arginine/serine-rich coiled-coil 1	NM_016625
CDC5L	cell division cycle 5-like	NM_001253
PANK3	pantothenate kinase 3	BX648891
FECH	ferrochelatase	NM_001012515
NR3C1	nuclear receptor subfamily 3, group C, member 1 (glucocorticoid receptor)	NM_001018077
JMY	junction mediating and regulatory protein, p53 cofactor	NM_152405
ZC3H12B	zinc finger CCCH-type containing 12B	NM_001010888
HIST2H2BF	histone cluster 2, H2bf	NM_001024599
DDX21	DEAD (Asp-Glu-Ala-Asp) box helicase 21	NM_001256910
ATF1	activating transcription factor 1	NM_005171
GEMIN2	gem (nuclear organelle) associated protein 2	NM_003616
SUMO1	small ubiquitin-like modifier 1	NM_003352
lnc-POLR2F-1	lnc-POLR2F-1:1	
MAMSTR	MEF2 activating motif and SAP domain containing transcriptional regulator	NM_182574
APOBEC4	apolipoprotein B mRNA editing enzyme, catalytic polypeptide-like 4 (putative)	NM_203454
PPCS	phosphopantothenoylcysteine synthetase	NM_001287507
MEF2A	myocyte enhancer factor 2A	NM_001171894
RBM3	RNA binding motif (RNP1, RRM) protein 3	NM_006743
MYO3A	myosin IIIA	BC036079
GPX1	glutathione peroxidase 1	NM_201397
TAF4B	TAF4b RNA polymerase II, TATA box binding protein (TBP)-associated factor, 105kDa	NM_001293725
MMAB	methylmalonic aciduria (cobalamin deficiency) cblB type	NM_052845
UHRF1	ubiquitin-like with PHD and ring finger domains 1	NM_001290050
UGDH	UDP-glucose 6-dehydrogenase	NM_003359
VNN1	vanin 1	NM_004666
HLF	hepatic leukemia factor	NM_002126
TMF1	TATA element modulatory factor 1	NM_007114
ATXN1	ataxin 1	NM_000332
TUBBP5	tubulin, beta pseudogene 5	NR_027156
NR3C1	nuclear receptor subfamily 3, group C, member 1 (glucocorticoid receptor)	NM_001018077

Cellular response to stress

USP1	ubiquitin specific peptidase 1	NM_003368
TCEA1	transcription elongation factor A (SII), 1	NM_006756
CTSV	cathepsin V	NM_001333
FGF1	fibroblast growth factor 1 (acidic)	NM_000800
SNCA	synuclein, alpha (non A4 component of amyloid precursor)	NM_007308
STC1	stanniocalcin 1	NM_003155
ARPP21	cAMP-regulated phosphoprotein, 21kDa	NM_016300
BCL2	B-cell CLL/lymphoma 2	NM_000633
SPATA18	spermatogenesis associated 18	NM_145263
SLC52A3	solute carrier family 52 (riboflavin transporter), member 3	NM_033409
PRDX3	peroxiredoxin 3	NM_006793
ANXA1	annexin A1	NM_000700
ETS1	v-ets avian erythroblastosis virus E26 oncogene homolog 1	NM_005238
BMPR2	bone morphogenetic protein receptor, type II (serine/threonine kinase)	NM_001204
HSPA5	heat shock 70kDa protein 5 (glucose-regulated protein, 78kDa)	NM_005347
TAF1	TAF1 RNA polymerase II, TATA box binding protein (TBP)-associated factor, 250kDa	NM_001286074
PTPRK	protein tyrosine phosphatase, receptor type, K	NM_002844
JMY	junction mediating and regulatory protein, p53 cofactor	NM_152405
INSIG1	insulin induced gene 1	NM_005542
PRKAA1	protein kinase, AMP-activated, alpha 1 catalytic subunit	NM_206907
ATF1	activating transcription factor 1	NM_005171
SUMO1	small ubiquitin-like modifier 1	NM_003352
lnc-POLR2F-1	lnc-POLR2F-1:1	
WNT5A	wingless-type MMTV integration site family, member 5A	NM_003392

MEF2A	myocyte enhancer factor 2A	NM_001171894
GPX1	glutathione peroxidase 1	NM_201397
UHRF1	ubiquitin-like with PHD and ring finger domains 1	NM_001290050
WNT4	wingless-type MMTV integration site family, member 4	NM_030761
EIF2S1	eukaryotic translation initiation factor 2, subunit 1 alpha, 35kDa	NM_004094
