# Supplementary material for: Transcriptional signature associated with early rheumatoid arthritis and healthy individuals at high risk to develop the disease
Source: PLoS One. 2018 Mar 27;13(3):e0194205. doi: 10.1371/journal.pone.0194205 (PMC5870959; doi:10.1371/journal.pone.0194205)
Supplement: S12 Table — (PDF) [file pone.0194205.s012.pdf]

Supplementary table 12. Specific induced pathways in relatives with ACCP+ compared to relatives ACCP-

Pathway	p-value	Matched Entities	Pathway Entities of Experiment Type
Hs_MHC_class_II_antigen_presentation_WP2679_76872	3.03E-05	10	90
Hs_Costimulation_by_the_CD28_family_WP1799_77064	4.11E-05	8	71
Hs_Interferon_gamma_signaling_WP1836_77096	1.28E-04	6	43
Hs_Fatty_Acid_Beta_Oxidation_WP143_71172	2.18E-04	6	34
Hs_TFs_Regulate_miRNAs_related_to_cardiac_hypertrophy_WP1559_68890	8.88E-04	3	15
Hs_Effects_of_Nitric_Oxide_WP1995_69910	0.00640001	2	8
Hs_Neural_Crest_Differentiation_WP2064_68974	0.0094352	2	101
Hs_Acetylcholine_Synthesis_WP528_76269	0.01298301	2	7
Hs_Trafficking_and_processing_of_endosomal_TLR_WP2709_76922	0.0170148	2	8
Hs_Triacylglyceride_Synthesis_WP325_71223	0.02356568	3	24
Hs_Endothelin_Pathways_WP2197_74852	0.02598208	1	33
Hs_Amino_acid_conjugation_of_benzoic_acid_WP521_63156	0.02598208	1	4
Hs_Nanoparticle_triggered_regulated_necrosis_WP2513_74182	0.02598208	1	11
Hs_alpha-linolenic_(omega3)_and_linoleic_(omega6)_acid_metabolism_WP2724_76941	0.02642153	2	11
Hs_AGE-RAGE_pathway_WP2324_71701	0.02848138	5	66
Hs_Activation_of_Chaperone_Genes_by_XBP1(S)_WP2667_76852	0.029068	4	46
Hs_mRNA_Capping_WP1861_76815	0.03535084	3	28
Hs_Fatty_acid,_triacylglycerol,_and_ketone_body_metabolism_WP1817_77087	0.03814839	4	49
Hs_Generic_Transcription_Pathway_WP1822_77033	0.04218413	3	30
