# Supplementary material for: Transcriptional signature associated with early rheumatoid arthritis and healthy individuals at high risk to develop the disease
Source: PLoS One. 2018 Mar 27;13(3):e0194205. doi: 10.1371/journal.pone.0194205 (PMC5870959; doi:10.1371/journal.pone.0194205)
Supplement: S13 Table — (PDF) [file pone.0194205.s013.pdf]

Supplementary table 13. Induced pathways in preclnical and clinical AR represented by relatives with ACCP+ and AR patients compared to relatives ACCP-

Pathway	Relatives ACCP+			RA patients		
	Matched Entities	Pathway Entities of Experiment Type	p-value	Matched Entities	Pathway Entities of Experiment Type	p-value
Hs_Complement_and_Coagulation_Cascades_WP558_67786	2	64	0.3743229	6	64	1.40E-04
Hs_BDNF_signaling_pathway_WP2380_71549	5	141	0.29964626	8	141	0.00188149
Hs_Wnt_Signaling_Pathway_WP428_71365	4	61	0.07054249	5	61	0.00267845
Hs_Androgen_receptor_signaling_pathway_WP138_72130	4	88	0.19576426	6	88	0.00286521
Hs_Serotonin_Receptor_4-6-7_and_NR3C_Signaling_WP734_74438	1	19	0.3936679	3	19	0.00322424
AndrogenReceptor	3	94	0.44297302	6	94	0.00397564
Wnt	5	104	0.13066246	6	104	0.00619828
Hs_DNA_Damage_Response_(only_ATM_dependent)_WP710_70109	6	97	0.00146364	4	97	0.00713114
Hs_Formation_of_Fibrin_Clot_(Clotting_Cascade)_WP1818_76965	1	30	0.546213	3	30	0.01187087
Hs_Integrated_Cancer_pathway_WP1971_71249	1	36	0.58070517	3	36	0.01540314
Hs_Wnt_Signaling_Pathway_and_Pluripotency_WP399_74897	5	100	0.09829516	5	100	0.01738259
TGFBR	6	134	0.13350268	6	134	0.02004555
Hs_PIP3_activates_AKT_signaling_WP2653_76821	2	91	0.54615563	4	91	0.02560972
Hs_Interleukin-11_Signaling_Pathway_WP2332_71360	3	40	0.08510803	3	40	0.02571158
Hs_Aryl_Hydrocarbon_Receptor_WP2586_76318	1	47	0.6513437	3	47	0.02571158
Hs_Signaling_by_Insulin_receptor_WP1913_77046	5	75	0.0393615	4	75	0.02803931
Hs_Prostate_Cancer_WP2263_73838	4	116	0.30288115	5	116	0.02853272
Hs_Integrated_Breast_Cancer_Pathway_WP1984_72732	7	164	0.02485127	5	164	0.03162374
Hs_Translation_Factors_WP107_69767	2	50	0.35555542	3	50	0.04103661
Hs_IL-3_Signaling_Pathway_WP286_72139	3	49	0.13441081	3	49	0.04321275
Hs_MicroRNAs_in_cardiomyocyte_hypertrophy_WP1544_75258	9	104	3.05E-04	4	104	0.04379221
Hs_Translocation_of_GLUT4_to_the_Plasma_Membrane_WP2777_77058	4	51	0.04323081	3	51	0.04773436
Hs_TCR_signaling_WP1927_76950	8	74	8.31E-05	3	74	0.05744057
Hs_Latent_infection_of_Homo_sapiens_with_Mycobacterium_tuberculosis_WP2700_76907	3	62	0.03869027	2	62	0.07760624

Hs_TSH_signaling_pathway_WP2032_75384	5	66	0.02848138	3	66	0.08840181
Hs_Insulin_Signaling_WP481_72080	9	161	0.02437216	5	161	0.11346993
Hs_EGF-EGFR_Signaling_Pathway_WP437_72106	10	162	0.00964363	5	162	0.11567275
Hs_Energy_Metabolism_WP1541_68947	5	47	0.00728563	2	47	0.17221336
Hs_Corticotropin-releasing_hormone_WP2355_71393	6	90	0.02993017	3	90	0.17352201
IL9	2	12	0.03744847	1	12	0.17536251
Hs_Cardiac_Hypertrophic_Response_WP2795_76316	5	54	0.01295823	2	54	0.21266016
Hs_Electron_Transport_Chain_WP111_71266	10	104	3.56E-04	3	104	0.22649026
Hs_Mitochondrial_LC-Fatty_Acid_Beta-Oxidation_WP368_71383	3	16	0.0076009	1	16	0.22670752
Hs_Metabolism_of_nitric_oxide_WP1850_77097	3	17	0.00905353	1	17	0.23903722
Hs_Oxidative_phosphorylation_WP623_68894	5	63	0.01972986	2	63	0.24797079
Hs_Visual_phototransduction_WP2776_77056	5	67	0.02687827	2	67	0.27754122
Hs_G1_to_S_cell_cycle_control_WP45_71377	5	68	0.03186591	2	68	0.29525807
Hs_Parkin-Ubiquitin_ Proteasomal_ System_ pathway_ WP2359_72121	5	73	0.03364835	2	73	0.30115107
Hs_Transcriptional_Regulation_of_White_Adipocyte_Differentiation_WP2751_76992	5	74	0.04138897	2	74	0.3246263
Hs_IL-7_Signaling_Pathway_WP205_70018	3	27	0.02627396	1	27	0.33086675
Hs_Gastric_cancer_network_1_WP2361_71382	3	28	0.03535084	1	28	0.36237556
Hs_Respiratory_electron_transport,_ATP_synthesis_by_chemiosmotic_coupling,_and_heat_production_by_uncoupling_proteins._WP1902_77091	11	94	3.32E-05	2	94	0.44309214
Hs_Lipid_digestion,_mobilization,_and_transport_WP2764_77026	4	41	0.01514362	1	41	0.44829082
Hs_Binding_and_Uptake_of_Ligands_by_Scavenger_Receptors_WP2784_77068	6	195	0.00130905	1	195	0.5302673
Hs_Glycolysis_and_Gluconeogenesis_WP534_74524	4	49	0.03574297	1	49	0.5377641
Hs_Nucleotide_Excision_Repair_WP1980_76859	4	49	0.03814839	1	49	0.5451415
Hs_Wnt_Signaling_Pathway_Netpath_WP363_70630	4	51	0.04323081	1	51	0.55954564
Hs_Focal_Adhesion_WP306_71714	11	188	0.00897285	3	188	0.56379765
Hs_Asparagine_N-linked_glycosylation_WP1785_76985	4	53	0.04867506	1	53	0.57349443
Hs_Integrin-mediated_Cell_Adhesion_WP185_71391	8	99	0.00431048	1	99	0.796657
Hs_MAPK_Signaling_Pathway_WP382_72103	13	168	4.73E-04	1	168	0.9331941
